# Taxonomic revision of *Stigmatomma* Roger (Hymenoptera: Formicidae) in the Malagasy region

**DOI:** 10.3897/BDJ.4.e8032

**Published:** 2016-06-13

**Authors:** Flavia A. Esteves, Brian L. Fisher

**Affiliations:** ‡ California Academy of Sciences, San Francisco, United States of America

**Keywords:** Malagasy bioregion, taxonomy, ants, Amblyoponinae, Madagascar, Seychelles

## Abstract

In this study we present the first taxonomic revision of the ant genus *Stigmatomma* in the Malagasy biogeographic region, re­describe the previously known *S. besucheti* Baroni-Urbani, and describe seven new species to science (*S. bolabola* **sp. n.**, *S. irayhady* **sp. n.**, *S. janovitsika* **sp. n.**, *S. liebe* **sp. n.**, *S. roahady* **sp. n.**, *S. sakalava* **sp. n.**, and *S. tsyhady* **sp. n.**). The revision is based on the worker caste, but we provide brief descriptions of gynes and males for some species. Species descriptions, diagnosis, character discussion, identiﬁcation key, and glossary are illustrated with 360 high-quality montage and SEM images. The distribution of *Stigmatomma* species in Madagascar are mapped and discussed within the context of the island’s biomes and ecoregions. We also discuss how some morphometric variables describe the diﬀerences among the species in the bioregion. Open science is supported by providing access to R scripts, raw measurement data, and all specimen data used. All specimens used in this study were given unique identifies, and holotypes were imaged. Specimens and images are made accessible on AntWeb.org.

## Introduction

*Stigmatomma* Roger (1859), the largest genus of the ant subfamily Amblyoponinae, currently contains more than 40 species among extant and fossil taxa (AntCat 2016). Its systematics has recently received attention. The genus was revived as a valid after many years as a synonym of *Amblyopone* (Yoshimura and Fisher 2012b), and is considered to be closely related to the Amblyoponinae genera *Adetomyrma*, *Myopopone*, *Mystrium*, and *Xymmer* (a group also known as XMMAS clade; Ward and Fisher 2016, Yoshimura and Fisher 2012b, Brady et al. 2006, Ouellette et al. 2006, Saux et al. 2004). The most recent phylogenetic hypothesis on Amblyoponinae divided the genus into two groups (Ward and Fisher 2016). *Fulakora*, resurrected from its synonymy under *Stigmatomma* and elevated to generic status, is a predominantly Neotropical lineage that contains some Australasian species. The remainder species continue to be assigned to *Stigmatomma*. However, the phylogeny was inconclusive regarding the monophyly of the newly delimited *Stigmatomma*, and its relationship with the other XMMAS lineages remains debatable. We believe that a more uniform and exhaustive taxon sampling in future phylogenetic studies may help to clarify these uncertainties.

*Stigmatomma* is distributed globally, but very little is known about the genus in the Malagasy region, apart from the description of *S. besucheti* (Baroni Urbani 1978) from the Seychelles.

Madagascar and its surrounding islands are extremely biodiverse, have a high rate of animal and plant endemism, and possess exceptional rates of habitat loss due to human activity (Goodman and Benstead 2004, Myers et al. 2000). Ninety percent of the original forest cover is estimated to have disappeared from the island since the arrival of humans around 2000 years ago (Du Puy and Moat 1996, Myers et al. 2000, Goodman and Benstead 2004). The region is likely home to 1,300 ant species, of which about 60% are undescribed (Fisher 2005, Fisher 2004); furthermore, 95% of described ant species in the region are found nowhere else in the world (Fisher 2004).

Ants play a large role in a terrestrial ecosystem (Alonso 2000, Andersen 1990, Hoffmann et al. 2000), and basic knowledge of their taxonomy and distribution may provide a baseline for all subsequent research and conservation efforts including them. Brian Fisher and members of the Malagasy Arthropod Team, based at the Madagascar Biodiversity Center in Madagascar, have conducted arthropod inventories in the Malagasy region for the last twenty years in an attempt to unravel the diversity of ants in that area. They have contributed to twenty-seven taxonomic revisions, which have added more than 300 new ant species to the Malagasy ant fauna list to date. From the material they have collected, more than 900 *Stigmatomma* specimens have been databased, and more than 150 images of these ants are available online on AntWeb.

This study presents the first taxonomic revision for the genus *Stigmatomma* in the Malagasy region and recognizes eight species, of which seven are newly described. It focuses on the worker caste, but images and a brief description of gynes and males are provided when possible.

### Biology

Our understanding of the biology of the species assigned to *Stigmatomma* is far from comprehensive as it is based on generalizations from limited observations of a few species. One of the major culprits for our lack of observations is the cryptobiotic lifestyle of these ants, which hampers access to their colonies and studies on their behavior (Ward and Fisher 2016).

Predominantly, *Stigmatomma* species nests in the soil or in rotten logs of humid forest habitats (Brown 1960). Workers are usually solitary hunters (Masuko 1993, Traniello 1978), but *S. reclinatum* (Mayr 1879), from Indomalaya region, has been found to recruit aid to recover prey (Billen et al. 2005, Ito 1993a). *Stigmatomma* prey upon other arthropods, especially geophilomorph centipedes (Gotwald and Lévieux 1972, Brown 1960)—observations indicate that up to 80% of the diet of *S. silvestrii* Wheeler 1928 (Palearctic region) is composed of such centipedes (Masuko 1993).

Larvae feed directly on prey when positioned on this food source (Brown 1960). We are not aware of any report of trophallaxis between larvae and adults of *Stigmatomma*. Instead, studies indicate that female adults perform nondestructive cannibalism on their own larvae. This practice, also known as Larval Hemolymph Feeding (LHF), consists of ingestion of hemolymph dripping from punctures made by adults in the larval integument. It was described for the Nearctic *S. pallipes* (Haldeman 1844) and for *S. silvestrii* (Haskins 1928, Masuko 1986, respectively). In the latter species, queens of mature colonies seem to feed exclusively on larval hemolymph. LHF was also reported for species of other Amblyoponinae genera (*e.g*., *Amblyopone*, *Myopopone*, *Mystrium*, and *Prionopelta*; Ito 2010, Ito and Billen 1998, Wheeler and Wheeler 1988).

The majority of the species produces winged gynes. However, some members of the *Stigmatomma reclinatum* species-group does not present a morphologicaclly distinct queen, and reproduction is performed by gamergates (Ito 1993b, Ito 1991). Within these colonies, dominance is established through chemical and aggressive interactions (Ito 1993b).

## Materials and methods

### ​Species delimitation

The biological species concept guides species delimitation in this study, implying that species represent reproductively isolated entities, enclosing one or many populations connected by gene flow (Coyne and Orr 2004). We thus used morphological discontinuities as evidence for species separation preferentially maintained in sympatry among closely related forms.

### Species names

In this study, new species names formed from a personal name are nouns in apposition and thus invariant. All other new species names presented are not latinized words, and thus also indeclinable (ICZN article 31.2.3, ICZN 1999).

### Specimen records

Every specimen we examined bears a specimen code label (e.g., CASENT0797614, ANTWEB1008502). Each code is a registered unique identifier, which aggregates several information regarding a given specimen on AntWeb (e.g., collection record, images, identification). Specimen data can be accessed on AntWeb through the persistent URL of its specimen code: www.antweb.org/specimen/“specimen code" (e.g., www.antweb.org/specimen/CASENT0797614).

### Terminology

Morphological terminology used follows Keller (2011), unless otherwise stated:

- Aedeagal apodemes (Snodgrass 1935): pair of anterior apodemes of the aedeagus that are connected to the majority of the aedeagal muscles (Fig. 1b, d).
- Aedeagus (Snodgrass 1935): the intromittent organ of the male genitalia. It projects from between the anterior portion of the parameres, and contains the aedeagal apodemes and the penisvalvae (Fig. 1a, d).
- Anepisternum (as in Bolton 1994): dorsal subdivision of the mesepisternum, separated from the katepisternum by the mesepisternal sulcus (Fig. 2a).
- Arolium: adhesive pretarsal organ (Fig. 3d).
- Basal ring (Snodgrass 1957): anterior annular sclerite of the male genitalia. It is connected anteriorly to the muscles from the abdominal segment IX, and posteriorly to the muscles of the aedeagus, volsellae, and parameres (Fig. 1a, b).
- Basimere (Snodgrass 1957): anterior portion of each paramere (Fig. 1b, c).
- Basivolsella (Peck 1937): anteroventral plate of the volsella; supports the cuspis and the digitus (Fig. 1b, c).
- Calcar of strigil: protibial spur, which together with the comb of strigil forms the antennal cleaning organ, which is also known as strigil (Fig. 3a).
- Comb of strigil: comb-­like structure on probasitarsus, which together with the calcar forms an antennal cleaning organ (Fig. 3a).
- Cuspis (Snodgrass 1941): lobe of the volsella continuous to the basivolsella; located between the paramere and the digitus (Fig. 1a, b, c).
- Digitus (Snodgrass 1941): movable lobe of the volsella; located between the cuspis and the aedeagus (Fig. 1a, b, c).
- Epistomal sulcus: sulcus that divides the clypeus posteriorly and laterally from the remainder of the head (Richards 1977; Fig. 4a).
- Frontal lobes: in this study, we use this term to name the trait formed by: (1) the median arch projection of the torulus (torular lobe in Keller 2011); or (2) the many degrees of fusion between the anterior dorso­lateral expansion of the frontal carina (posttorular flange in Keller 2011) and the median arch projection of the torulus (Fig. 4a).
- Fronto­clypeal sulcus: medial section of the epistomal sulcus running between the anterior tentorial pits (Fig. 4a).
- Gaster (as in Bolton 1990): formed by the third, fourth, fifth, sixth, and seventh abdominal segments in Stigmatomma females. When mentioned separately, the Roman numeral of the corresponding homologous true segment labels abdominal segments (i.e., III– VII; Fig. 4b).
- Genal tooth (Brown 1960): lateral cuticular projection on the anterior genal angle (Fig. 4a).
- Hypopygium: sternite of abdominal segment VII in adult females (Fig. 4b).
- Katepisternum (as in Bolton 1994): ventral subdivision of the mesepisternum, separated from the anepisternum by the mesepisternal sulcus (Fig. 3d).
- Lower and upper metapleuron (Snodgrass 1910): secondary division of the metapleuron in a dorsal wing bearing part and a ventral leg-bearing part (Fig. 5b​).
- Median area of the clypeus: clypeal area between and below the frontal carinae (Fig. 4a).
- Mesepimeral lobe (as epimeral lobe in Yoshimura and Fisher 2012b): posterodorsal lobe of the mesepimeron, which covers the metathoracic spiracle (Fig. 5b).
- Mesepimeron (as in Gibson et al. 1998): posterodorsal portion of the mesopleuron, which is differentiated from the mesepisternum by the mesopleural suture (Fig. 5b).
- Mesepisternum (as in Huber and Sharkey 1993): anterior subdivision of the mesopleuron, usually comprising most of the mesopleuron (Figs 2b, 5b).
- Mesobasitarsal sulcus: longitudinal impression situated on the antero­dorsal face of the mesobasitarsus (Fig. 3b).
- Mesopleural suture (Snodgrass 1910): vertical or oblique suture dividing the mesopleuron into an anterior/ventral mesepisternum and a posterior/dorsal mesepimeron. It extends from the base of the wing process to the coxal process.
- Mesopleuron (as in Huber and Sharkey 1993): lateral and ventral part of the mesothorax, which is the second and largest of the three primary subdivisions of the thorax, bearing the middle pair of legs and, when present, the forewings.
- Mesoscutellar-axillar complex (as in Gibson et al. 1998): region of the mesonotum posterior the transscutal articulation; often simply referred to as the scutellum, but composed of the scutellum and axillae (Fig. 5a).
- Mesoscutum (as in Gibson et al. 1998): region of the mesonotum anterior to the transscutal articulation and scutellar-axillar complex (Fig. 5a).
- Mesosoma (as in Bolton 1990): formed by three thoracic segments plus the propodeum (abdominal segment I; Fig. 4b).
- Metabasitarsal sulcus: longitudinal impression situated on the anterior face of the metabasitarsus (Fig. 3c).
- Metapleuron (as in Gibson et al. 1998): pleuron of the metathorax (Fig. 5b).
- Microtrichia: setae­-like cuticular projections.
- Notaulus (pl. notauli; as in Gibson et al. 1998): paired lines or grooves on the mesoscutum that subdivide the sclerite into a median midlobe and lateral lobes (Fig. 5a).
- Parameres (Snodgrass 1957): elongated pair of lateral lobes of the male genitalia; subdivided into the anterior basimere and the posterior telomere (Fig. 1a, b, c).
- Penisvalvae (Snodgrass 1941): pair of well-developed, dorsoventrally and anteroposteriorly elongated, sclerotized lateral plates of the aedeagus (Fig. 1b, d).
- Petiolar laterotergite: paired long, narrow, strip-­like area of cuticle parallel to the ventral margin of the petiolar tergite (Fig. 6a).
- Petiolar proprioceptor zone: a depression sharply delineated anteriorly and bearing numerous sensilla on the anterior-most part of the petiolar sternite (Fig. 6a).
- Petiole: abdominal segment II (Fig. 4b).
- Poststernite: posterior remaining portion of sternite not concealed by an articulation (Fig. 6b).
- Pygostyles (as in Yoshimura and Fisher 2007; referred as cerci in Gibson et al. 1998): paired sensory finger-like projections that articulate with the tenth abdominal tergite of male ants.
- Scuto-scutellar suture (as in Gibson et al. 1998): groove or line that separates the axillae from the scutellum (Fig. 5a).
- Subpetiolar process (as in Ward 1994): anteroventral projection of the petiolar poststernite.
- Supraclypeal area (frontal triangle in Bolton 1994): well ­delineated and unpaired area lying immediately posterior to the median part of the clypeus, between the frontal carinae (Fig. 4a).
- Suture and sulcus: the fusion of two sclerites forms a suture, while a depression formed by an invagination of the cuticle corresponds to a sulcus.
- Telomere (Snodgrass 1957): hollow posterior portion of each paramere (Fig. 1b, c).
- Transscutal articulation (as in Gibson et al. 1998): transverse line across the mesonotum at the level of the forewings that differentiates an anterior mesoscutum and posterior scutellar-axillar complex, and that permits flexion of the mesonotum for flight.
- Volsellae (Snodgrass 1957): pincer-like organ located between each paramere and aedeagus. Formed by the cuspis, digitus, and basivolsella (Fig. 1a, b, c).

Sculpture terminology follows Harris (1979) as below. In order to describe additive sculpture, we employ a dash between terms (e.g., foveate-costate means numerous pits among longitudinal costae).

- Alveolate: honeycombed, with regular, deep, angular cavities separated by thin partitions; furnished with cells or alveoli.
- Areolate: divided into a number of small, irregular, nonparallel spaces.
- Carinate: keeled, with one, or several, but usually few longitudinal narrow raised ridges.
- Catenate: with longitudinal, connected elevations like links in a chain.
- Confused: indefinite outlines.
- Costate: with longitudinal raised ridges (costae); coarser than carinate.
- Costulate: less prominent than costate.
- Dispersed: scattered markings or small sculptures.
- Foveate: pitted, with numerous, regular depressions or pits (foveae).
- Foveolate: with small, deep pits; finely pitted.
- Imbricate: partly overlapping, like shingles on a roof or scales on a fish.
- Nodulate: with small knots or swellings.
- Plicate: folded; with folds.
- Puncticulate: dispersed points or punctures, with very fine, widely spaced punctures.
- Smooth: devoid of any sculpturing.
- Strigate: with narrow, transverse raised ridges or impressed lines.
- Rugose: wrinkled.
- Rugulose: minutely wrinkled.
- Taeniate: with broad, longitudinal ribbon-­like markings; shaped like a tapeworm.
- Tuberculate: furnished with rounded, projecting lobes.

We describe setae and cuticular projections with the following terminology:

- Acuminate: tapering to a point (Fig. 7a).
- Antler-like: branched, shaped like an antler (Fig. 7b).
- Blunt: not sharp, a worn-down apex (Fig. 7c).
- Conic: shaped like a cone (Fig. 7d).
- Dentiform: shaped like a tooth (Fig. 7c, d).
- Digitiform: shaped like a finger (Fig. 7e).
- Filiform: shaped like a thread; filamentous. Here used to describe setae having a regular, hair-like shape (Fig. 8a).
- Flattened­-apex: flattened apically, not round (Fig. 7a).
- Glabrous: devoid of hair or cuticular projections.
- Lanceolate: shaped like the head of a lance (Fig. 7e).
- Mucronate: ending abruptly in a sharp point (Fig. 7c).
- Paddle-like: shaped like a paddle (Fig. 7f).
- Scrobiculate: uniformly covered with short, oblong or trench-like hollows.
- Spatular: shaped like a spatula (Fig. 7a, c).
- Spiniform: shaped like a spine (Fig. 8a).
- Squamiform: shaped like a scale (Fig. 7e).
- Stout: heavily built (Fig. 8a).
- Strap­-like: shaped like a long and narrow strip (Fig. 7f).
- Tongue-like: shaped like a tongue (Fig. 8b).
- Truncated: having the apex cut off transversally; lacking the apex (Fig. 7b).
- Tubiform: shaped like a tube (Fig. 8c).

The terminology used to describe pilosity inclination, in regards to cuticle surface, follows Wilson (1955).

**Figure 9.**
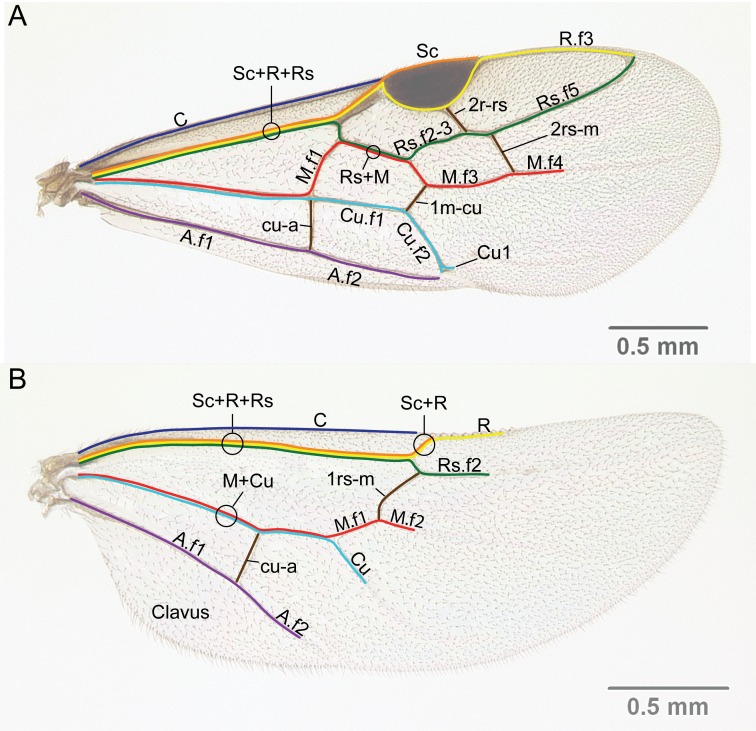
Diagram of the *Stigmatomma* generalized wing venation: A, forewing; B, hindwing. Abbreviations: C, costa; Sc, subcosta; R, radius; Rs, radial sector; M, media; C, cubitus; A, anal. The position of vein's free abscissas are indicated by the letter f followed by a cardinal number. Images by Masashi Yoshimura, available at AntWeb.org (specimen CASENT0083104). llustration by F. A. Esteves.

Wing venation (Fig. 9) follows Archibald et al. (2006).

### Measurements and indices

We used indices and measurements to quantify size, and as means of comparison among *Stigmatomma* species. Measurements were taken on a Leica MZ APO stereomicroscope, rounded to the nearest 0.01 mm. They are expressed in mm, and presented as minimum and maximum values with holotype measurements within parentheses. Indices are rounded to the nearest integer value, and expressed as minimum and maximum values with holotype index within parentheses. The raw data are presented in Suppl. material 1.

**Figure 10.**
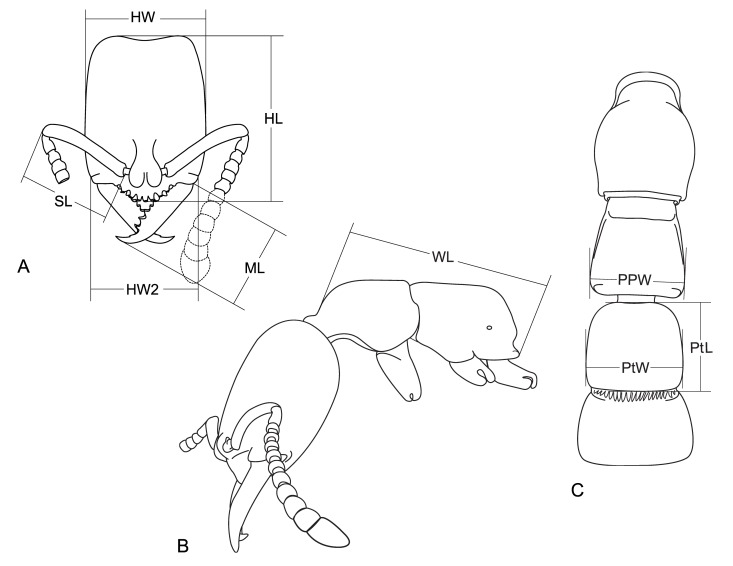
Measurements taken from *Stigmatomma* worker caste. A: fullface view; B: lateral view of head and mesosoma; C: dorsal view of mesosoma and anterior abdominal tergites. Abbreviations: HL, head length; HW, head width; HW2, head width 2; ML, mandibular length; PPW, propodeal posterior width; PtL, petiolar length; PtW, petiolar width; SL, scape length; WL, Weber's length of mesosoma. Illustrations by F. A. Esteves.

- Head length (HL): in fullface view, straight line from the anterior clypeal margin to the midpoint of a straight imaginary line connecting posterior corners of the head (Fig. 10A).
- Head width (HW): maximum width of the head, including eyes when present (Fig. 10A).
- Head width 2 (HW2): width of the head immediately posterior to the posterolateral margin of the clypeus (as in Taylor 1978; Fig. 10A).
- Scape length (SL): length of the scape (first antennal segment), excluding its basal constriction or neck and condyle (Fig. 10A).
- Mandibular length (ML): outer length of the mandible (as in Taylor 1978; Fig. 10A).
- Weber’s length of mesosoma (WL): diagonal length of the mesosoma in profile, from base of anterior slope of pronotum to metapleural lobe (Fig. 10B).
- Propodeal posterior width (PPW): width of posterior margin of propodeal dorsal face, in dorsal view (Fig. 10C).
- Petiolar length (PtL): maximum length of petiole in dorsal view (Fig. 10C).
- Petiolar width (PtW): maximum width of petiole in dorsal view (Fig. 10C).
- Cephalic index (CI): HW/HL ×100.
- Scape index (SI): SL/HL ×100.
- Mandibular index (MI): ML/HL ×100.
- Petiolar index (PtI): PtL/PtW ×100.

We employed a UPGMA hierarchical cluster analysis to visualize how specimens are grouped based on the differences in their linear morphometry. We also compared the clustering result with our species hypothesis to see how well they reflect each other.

In the UPGMA analysis, specimens clustered together are morphometrically more similar than specimens grouped into different clusters (Legendre and Legendre 1998). All of the following steps were performed on the R platform (R Core Team 2015; see complete script in the Suppl. material 2).

Cluster analysis uses a dissimilarity matrix as input. As a measure of dissimilarity, we used Euclidean distances, defined as the squared differences of measurement values between each pair of specimens (function *dist*, method “euclidean”; stats package, R Core Team 2015):

\begin{equation*}
            d_{x,y} = { \sqrt{ \displaystyle\sum_{j=1}^{J}(x_{j}-y_{j})^2}}
        \end{equation*}

where *d* is the distance between specimens *x* and *y*, *J* is the total number of measurements taken from each specimen, and *j* is a given measurement.

Data normalization is imperative for unbiased Euclidean distances. It balances the contribution of each measurement to the distance matrix, neutralizing the weight of the absolute differences of larger variables (see more about data normalization in Gelman and Hill 2007). The impact of data normalization on Euclidean distances can be seen in this example of a simple model of two specimens, A and B, and three measurements (WL, PtL, and PtW):

\begin{equation*}
            d_{A,B} = { \sqrt{ (A_{WL}-B_{WL})^2+(A_{PtL}-B_{PtL})^2+(A_{PtW}-B_{PtW})^2}}
        \end{equation*}

\begin{equation*}
            d_{A,B} = { \sqrt{ (1.508-1.396)^2+(0.692-0.674)^2+(0.758-0.704)^2}}
        \end{equation*}

\begin{equation*}
            d_{A,B} = { \sqrt{ 0.0125+0.0003+0.0029}}
        \end{equation*}

\begin{equation*}
            d_{A,B} =0.125
        \end{equation*}

If the same measurement values were normalized to the log scale, the Euclidean distance between specimens A and B would be only 0.0022. Thus, before data normalization, the absolute difference between Weber’s length values dominates the equation’s result. To counteract this effect, we normalize original measurement values to the natural-log scale using function *log* (base package, R Core Team 2015) before calculating the dissimilarity distances between specimens.

UPGMA starts by combining the couple of most similar specimens into a group, then, it adds other specimens, or combines groups to groups, until all specimens are united by a common root. We used the method "average" as clustering strategy. It combines similar clusters together using the distance among cluster centroids as a dissimilarity measurement. For clustering, we used function *hclust* (stats package).

We use the cophenetic correlation coefficient to measure how well the cluster represents the distances between specimens. This provides a linear correlation coefficient between the cophenetic distances obtained from the cluster, and the original dissimilarity matrix that was used to build the cluster. The output value must be close to 1 for a high-informative cluster (Oksanen 2014, Oksanen 2015). To calculate the cophenetic correlation coefficient, we use the functions *cophenetic* and *cor* (stats package), and to plot the cluster, the function *plot* (graphics package, R Core Team 2015).

Cluster analysis captures the multivariate structure of a dataset, but it does not unveil the patterns of variation behind the clusters it builds. We used Principal Component Analysis (PCA) to visualize and interpret patterns of morphometric variation among specimens. As seen below, PCA offers solutions for two elements that hamper the detection of patterns underlying morphometric variation among specimens: variable correlation and multidimensionality.

Morphological measurements are biologically linked to each other as they describe traits of an organism, and therefore, they are generally correlated (Zelditch et al. 2012). PCA creates new variables, or components, to eliminate such correlations. PCA components combine linearly the original measurements, are independent from each other, and will act as new dimensions/axes in the ordination space (Zelditch et al. 2012, Marhold 2011).

In the morphometric space (*i.e*., space defined by the measurements), PCA draws its first component along the line that comprises the highest proportion of variation among specimens. Consecutively, it derives the remaining components to encompass the highest variation after derivation of the previous components. This process continues until the number of components equals the number of original measurements (Zelditch et al. 2012, Marhold 2011).

The newly computed components are uncorrelated (*i.e*., orthogonal in the space), and ideally, the first components will capture most of the variation among specimens (Zelditch et al. 2012, Marhold 2011). Hence, the first components may be used to produce a graphical representation of the dataset in a lower dimensional space. The analysis provides: (1) A matrix with eigenvectors values, which are the location of original measurements on each component axis. It indicates the contribution of each original variable to the component: the larger the absolute value, the more important the variable. (2) The proportion of the total variance encompassed by each component.

Eventually, PCA projects the position of each specimen onto the components (Zelditch et al. 2012, Marhold 2011). In other words, it fits the specimens into the ordination space. The coordinate, or location, of each specimen on a given component is the PCA score (Marhold 2011).

We executed all the steps of the Principal Component Analysis on the R platform (R Core Team 2015), as follows (see complete script in the Suppl. material 3).

First, we checked the original measurements for the presence of correlations (function *cor*, stats package; R Core Team 2015). PCA components are based on variable correlations, which means that high correlations between variables increase the success of the analysis (Zelditch et al. 2012, Marhold 2011).

Function *prcomp* (scale set to TRUE; stats package) executed PCA analysis on the original measurement matrix. It also standardized our dataset to zero mean and unit variance, which prevents dominance of variables with higher variance in the analysis (Manly 2004). Function *ggscreeplot* (ggbiplot package; Vu 2011) produced a screeplot of the proportional variation explained by each PCA component. Function *ggbiplot* (ggbiplot package) mapped species scores along the components that encompassed the majority of the variation exhibited by the dataset.

The data and R scripts underpinning the analysis presented above are deposited in the Dryad Data Repository at http://dx.doi.org/10.5061/dryad.m7340.

### Images

Extended focus montage images were created with a Leica DFC 425 camera and LEICA APPLICATION SUITE software (version 3.8; Leica Microsystems, Switzerland), and are available online at AntWeb. In addition, scanning electron microscopy (SEM) was used for observations of smaller characters. We prepared specimens for SEM adapting the procedure used by Keller (2011):

Workers kept in ethanol were washed in water and gently brushed to remove dirt particles, before being placed in 90% ethanol for 20 minutes. Specimens were then: (1) point mounted in a copper conductive triangle (TED PELLA, INC.) below the median and hind right coxae, and fixed in an SEM aluminum Zeiss stub (TED PELLA, INC.) via a double-­sided adhesive conducting PELCO tab (TED PELLA, INC.); and (2) left to air dry for at least 12 hours before scanning.

Point­-mounted dry specimens were submerged in warm water to dissolve the mounting glue before being placed in 90% ethanol, after which the same treatment described above was applied.

Specimens mounted on stubs were coated with gold­-palladium—this procedure was not applied to rare taxa (i.e., poorly represented in collections). Images were taken using a LEO/Zeiss 1450 VP SEM field emission scanning electron microscope at CASC, using the high voltage mode (HV) at a voltage of 10 kV. Images of uncoated ants were taken using the SEM at a variable pressure secondary electron mode (VPSE) with the following configuration: VP target pressure around 20Pa, spot size around between 500 and 600, VPSE collector bias at 390V, and voltage at 20kV. At least three specimens of each species were imaged when permitted by the available number of specimens.

### Maps

For Madagascar, species distributions were mapped over a shaded relief of the island, overlaid by an elevation layer and the outlines of five simplified ecoregion zones of the country (Burgess et al. 2004): humid forests, subhumid forests, dry deciduous forests, succulent woodlands, and spiny thickets. Mangroves were merged with the adjacent ecoregion since they are not biologically informative for *Stigmatomma*. The ecoregion classification used here only reflects the original primary vegetation of Madagascar. Nowadays, more than 82% of the island's original vegetation has been modified by human activities (Du Puy and Moat 1998).

For Seychelles, species distributions were mapped over a shaded-relief of the islands, overlaid by an elevation layer.

All of the following steps, unless otherwise noted, were performed on the R platform (R Core Team 2015; see Suppl. material 4 for script, which is also deposited in the Dryad Data Repository at http://dx.doi.org/10.5061/dryad.m7340).

1. Obtaining elevation raster layer for Madagascar: Function *getData* (raster package, Hijmans 2015) acquired elevation data for Madagascar, aggregating SRTM 90 m resolution data.
2. Obtaining elevation raster layer for Seychelles: Although function *getData* worked perfectly for Madagascar, it did not return any data for Seychelles. Thus, we downloaded elevation data directly from the International Centre for Tropical Agriculture (CIAT; Jarvis et al. 2008, available at srtm.csi.cgiar.org), and used function *readGDAL* (rgdal package, Bivand et al. 2015) to enter the data into R. Function *raster* (raster package) rasterized the elevation layer for Seychelles.
3. Obtaining and modifying ecoregions vector layers for Madagascar: In this study, ecoregion outlines of Madagascar are based on the vector data disclosed by the Terrestrial Ecoregions of the World (Olson et al. 2001, available at the WWF website). However, the original outlines were slightly mismatching the relief of Madagascar. To solve this, we combined the original ecoregion data with data from the Remaining Primary Vegetation of Madagascar (Du Puy and Moat 1996, available at the Kew Royal Botanic Gardens website), which has more natural outlines. QUANTUM GIS 1.8.0 (QGIS Development Team 2013) provided the tools to geoprocess these layers (i.e., Clip, Union, and Difference). Function *readOGR* (rgdal package) read the processed ecoregions files into R.
4. Reading distribution points for each *Stigmatomma* species: Function *read.csv* (utils package, R Core Team 2015) loaded into R a file containing the geographic coordinates of collection points for each specimen examined in this study.
5. Standardizing projections of raster and vector layers: Function *proj4string* (raster package) retrieved the vector data projection, and used it to set the projection of the elevation data. Function *CRS* (rgdal package) assigned that value to an R object, which was used as a liaison between retrieving and setting projections.
6. Obtaining the shaded relief of Madagascar: Function *terrain* (raster package) computed slope and terrain from the elevation data, which were used by function *hillShade* (raster package) to compute the shaded-relief layer.
7. Plottting maps: Function *plot* (raster package) drew the shaded relief of Madagascar and Seychelles, and overlaid them with the raw elevation layer. For Madagascar, function *plot* also overlaid the resuting image with the modified ecoregion layers. Function *grey* (grDevices package, R Core Team 2015) provided the different levels of gray for shaded-relief and elevation layers; function *alpha* (scales package, Wickham 2015) modified color transparency. Function *points* (graphics package, R Core Team 2015) drew species distributions over the map at their specified geographic coordinates.

Note that extensive myrmecological exploration of Madagascar is ongoing; we encourage readers to consult detailed and regularly updated distribution data available on AntWeb.org, where existing and future distributions can be mapped interactively and at higher resolution then the maps presented here.

### Depository acronyms

ANIC: Australian National Insect Collection, Canberra, Australia.

BMNH: The Natural History Museum, London, U.K.

CASC: California Academy of Sciences, San Francisco, California, U.S.A.

MCZC: Museum of Comparative Zoology, Harvard University, Cambridge, U.S.A.

NHMB: Naturhistorisches Museum, Basel, Switzerland.

NHMW: Naturhistorisches Museum, Vienna, Austria.

MHNG: Muséum d’Histoire Naturelle, Geneva, Switzerland.

MZSP: Museu de Zoologia da Universidade de Sao Paulo, Sao Paulo, Brazil.

USNM: National Museum of Natural History, Smithsonian Institution, Washington, D.C., U.S.A.

## Taxon treatments

### Stigmatomma

Roger 1859

Stigmatomma as junior synonym of *Amblyopone*: Emery and Forel 1879: 455; Mayr 1887: 546. Revived from synonymy: Dalla Torre 1893: 14. Subgenus of *Amblyopone*: Forel 1900: 55; Clark 1934: 27; Brown 1949: 87. Revived status as genus: Bingham 1903: 36; Emery 1911: 23; Creighton 1950: 31. Junior synonym of *Amblyopone*: Brown 1960: 155. Revived status as genus: Yoshimura and Fisher 2012b: 17. Senior synonym of *Arotropus*: Yoshimura and Fisher 2012b: 17.
Stigmatomma = *Arotropus*Provancher 1881: 205. Type-species: *Arotropus binodosus* (junior synonym of *Typhlopone pallipes*), by monotypy.
Stigmatomma Stigmatomma denticulatum Roger 1859Bingham 1903: 36. by subsequent designation

#### Diagnosis

**Figure 11.**
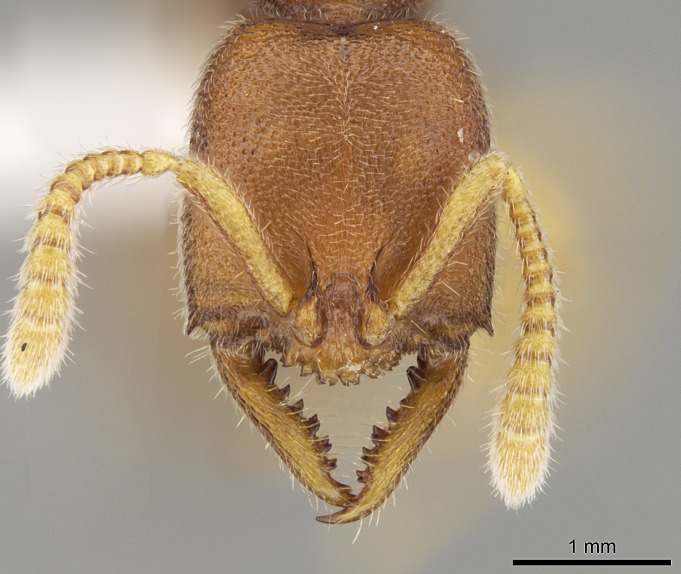
Holotype worker of *Stigmatomma liebe* **sp. n.** (CASENT0318428); dorsal face of the head. Image by F. A. Esteves; available at AntWeb.org

**Figure 13.**
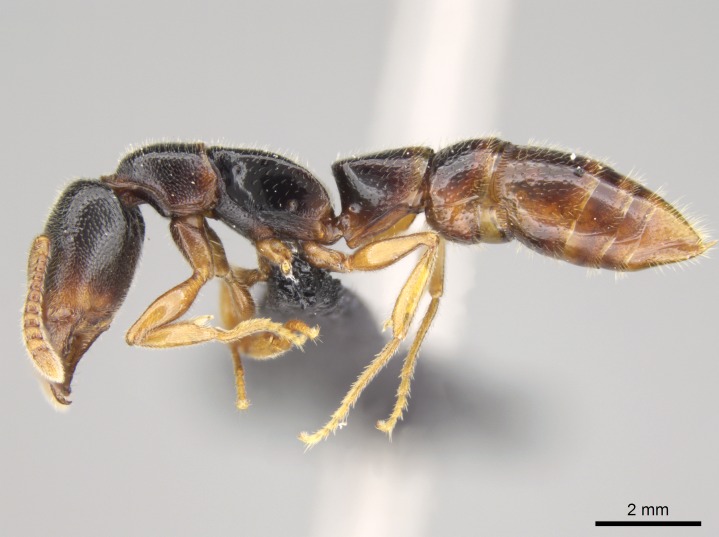
*Stigmatomma tsyhady* **sp. n.** worker; lateral view (CASENT0121332). Image by F. A. Esteves; available at AntWeb.org

**Figure 14.**
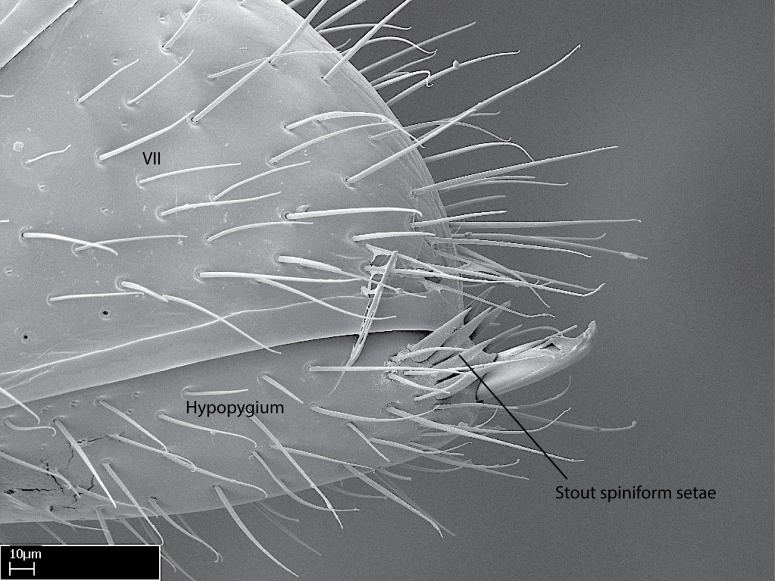
Abdominal segment VII of *Stigmatomma sakalava* worker **sp. n.** (CASENT0022146), lateral view, featuring stout spiniform setae on the apex of its hypopygium. Image by F. A. Esteves; available at AntWeb.org

Workers of *Stigmatomma* in the Malagasy bioregion – characters of the Amblyoponinae as described by Brown (1960) and the following characters:

1. Mandible elongate and linear, not as long as the head, pointed at the apex (Fig. 11). Masticatory and basal margins running parallel to each other along baso­apical axis, resulting in two rows of teeth (Fig. 12). Teeth of the same pair generally basally fused.
2. Median portion of clypeal anterior margin anteriorly projected (generally convex; Fig. 11). Anterior clypeal margin armed with single row of dentiform setae, arising from tubercle-­like cuticular projections or from the flat cuticle (Fig. 12​). Pair of long setae on clypeus, generally arising from its anterior margin.
3. Genal teeth present or absent.
4. Number of antennomeres: 10–12.
5. Under the stereomicroscope, pilosity similar present on all antennomeres (Fig. 11).
6. Palpal formula: 4:3; 4:2; or 2:2 (two maxillary and two labial).
7. Metanotal suture well developed to absent.
8. Mesepisternum generally divided into anepisternum and katepisternum (Fig. 2).
9. Number of mesotibial spurs: 0–2.
10. Anterodorsal face of mesobasitarsus generally with a longitudinal sulcus (Fig. 3b).
11. Number of metatibial spurs: 1–2.
12. Anterior face of metabasitarsus generally without a longitudinal sulcus.
13. Pretarsal claw simple; arolium present on pro-, meso-, and metapretarsi (Fig. 3d).
14. Petiole (abdominal segment II) sessile (Fig. 13). Subpetiolar process present; fenestra present or absent on its lateral face.
15. Constriction, generally scrobiculate, present between pretergite and postergite of abdominal segment III.
16. Prora present.
17. Scrobiculate constriction present between presclerites and postsclerites of abdominal segment IV.
18. Stout spiniform setae on apex of hypopygium present or absent (Fig. 14).

##### Comments on worker characters

The list of characters above forms an inclusive diagnosis of the genus, but no character can currently be pointed as unique for *Stigmatomma*.

**1.** In *Stigmatomma*, the total dental count (including teeth arranged in pairs) recorded for Malagasy species is 11–15, distributed from base to apex as follows: 1–3 single teeth, followed by 3–6 teeth pairs (generally fused at the base), a pre­apical (generally single) tooth, and an apical pointy tooth (Fig. 12). Tooth number and arrangement may be constant within some species, but not for all species we evaluated: it varies within nest series and even between left and right mandibles of the same specimen. Given that, we did not use these characters alone to isolate individual species.

The most basal tooth is enlarged in the majority of species we studied, but not in all (Fig. 12​​). This contradicts the opinion of Yoshimura and Fisher (2012b), which is that all Malagasy *Stigmatomma* species possess an enlarged basal tooth in their mandibles.

**Figure 15.**
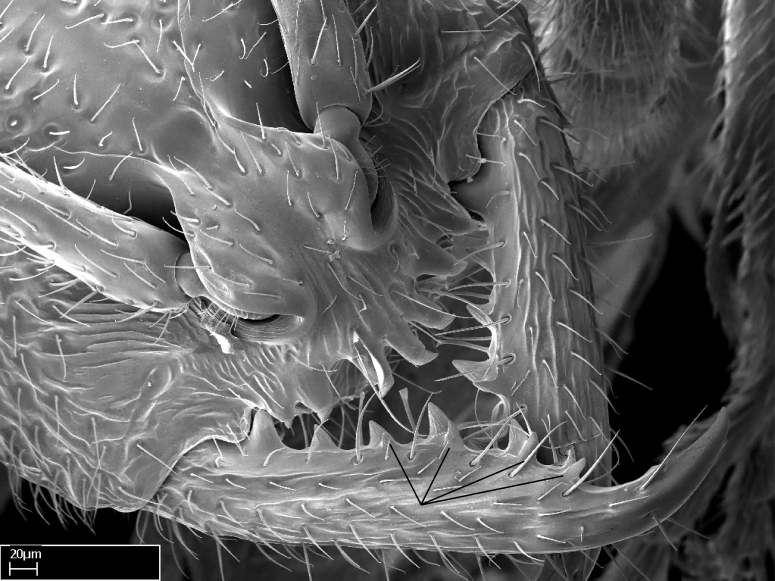
Mandibles of *Stigmatomma sakalava* **sp. n.** worker; dorsal view (CASENT0022146). Arrows point to dorsal tooth couples, which increase in size towards the mandible's apex. Image by F. A. Esteves; available at AntWeb.org

Teeth coupling generally occurs between teeth with similar dimensions (Fig. 12a). However, in two species (*Stigmatomma bolabola* **sp. n.** and *S. sakalava* **sp. n.**), dorsal teeth increase in size towards the mandible’s apex (Fig. 15​). In that case, the dorsal tooth is smaller than the ventral paired tooth, but at the mandible's apex. This also contradicts Yoshimura and Fisher (2014) and Yoshimura and Fisher (2012b), who were of the opinion that dorsal teeth are smaller than ventral teeth in the XMMAS clade genera. In their view, the genus *Amblyopone* would generally present mandibles with no teeth pairs, but if teeth were present, the dorsal tooth would be larger than the respective ventral pair. A species noteworthy in this discussion is *Stigmatomma pluto* (Gotwald and Lévieux 1972) (ANTWEB1008502; Afrotropical region), whose mandible has no basal teeth paired with mandibular teeth, thus resembling the mandible of *Amblyopone* (Fig. 16).

Among the other Amblyoponinae genera distributed in the Malagasy bioregion: *Prionopelta* has short and subtriangular mandibles, which are usually armed with three teeth on the apical half, so that basal and mastigatory margins are distinct (Fig. 17c). The mandibles of *Mystrium* are similar to those of *Stigmatomma* in their indistinct basal and mastigatory margins, but are longer than its head, and have blunt apex (Bolton 1994; Fig. 17b). Also in *Mystrium*, the ventral row of teeth is set far apart from the dorsal row (Yoshimura and Fisher 2014). *Adetomyrma* and *Xymmer*, like *Stigmatomma*, present mandibles that shorter than the head, with indistinct basal and masticatory margins and a pointy apex (Fig. 17a, d). While teeth are not disposed in pairs along the mandibles of *Adetomyrma* (Yoshimura and Fisher 2012b), the mandibles of *Xymmer* do have pairs of teeth.

In addition to the similarities and differences among the shape and configuration of the mandibles, an enlarged mandibular basal tooth is absent in all other Malagasy Amblyoponinae genera (Yoshimura and Fisher 2012a, Yoshimura and Fisher 2012b; Fig. 17).

**2.** Number and configuration of clypeal cuticular processes and associated dentiform setae vary among the evaluated species of *Stigmatomma*. All species present three to ten cuticular processes on the anterior margin of the clypeus. Each medial process bears one dentiform seta.

In half of the species (*tsyhady* species-complex members and *Stigmatomma janovitsika* **sp. n.**), the seta on the lateral-most process is laterodistally followed by a row of dentiform setae. These lateral rows extend laterad on the anterior clypeal margin, where it arises from flat cuticle (Fig. 12a). In few species (*S. bolabola* **sp. n.** and *S. sakalava* **sp. n.**), the lateral-most cuticular process is smaller, and does not bear any dentiform setae (Fig. 15). *S. besucheti* presents three medial cuticular processes that are followed laterodistally by a notch on the anterior clypeal margin. This notch is succeeded by a row of dentiform setae arising from flat cuticle (or from reduced cuticular processes; Fig. 12b). 

However, the number of medial cuticular processes may vary within some species and sometimes within nest series. Thus, we did not use such variations to isolate individual species.

Among the other Amblyoponinae genera present in the Malagasy region, *Mystrium*, like *Stigmatomma*, also presents a single row of cuticular projections bearing dentiform setae on the anterior clypeal margin (Fig. 17b; or see ANTWEB1008554 for high-magnification images). On the other hand, *Xymmer* has neither specialized setae nor cuticular tubercle-­like projections (Fig. 17d; or see ANTWEB1008499 for more images); in *Adetomyrma*, all dentiform clypeal setae arise from flat cuticle (Fig. 17a; or see ANTWEB1008494 for SEM images); and *Prionopelta* seems to have cuticular projections welded onto an anterior clypeal apron (Fig. 17c).

A pair of long setae is present on the anterior margin of the clypeus of all genera in the XMMAS clade in the Malagasy region, however, they are reduced and stouter in *Mystrium* (CASENT0002095).

**3.** The presence or absence of genal teeth is uniform within *Stigmatomma* species, and this character has relative importance to group species with similar morphology. In the Malagasy bioregion, this trait is present in all *Mystrium* species (Fig. 17b) and absent in *Adetomyrma* (Fig. 17a), *Prionopelta* (Fig. 17c), and *Xymmer* (Fig. 17d).

**4.** Despite the variation among species, the number of antennomeres is constant within the *Stigmatomma* species we studied. *Adetomyrma*, *Mystrium*, and *Xymmer* species present no variation for this character, with all having twelve­ antennomeres.

**5.** Under the stereomicroscope, the whole antenna is equally covered by setae in *Adetomyrma*, *Prionopelta*, *Stigmatomma*, and *Xymmer* (Figs 11, 17a, c, d). In *Mystrium*, the four apical-most antennomeres are covered with denser pilosity (​Fig. 17b). SEM images show that the apical antennomeres in *Mystrium* are actually covered by a different type of setae (ANTWEB1008554).

**6.** Without dissection, the maxillary and labial palpomeres are often extremely difficult to count in the species we studied.

Regarding the number of maxillary and labial palpomeres in other Amblyoponinae members in the Malagasy region, the palpal formula is constant within *Mystrium* (4:3) and *Prionopelta* (2:2) (Yoshimura and Fisher 2014), but not in *Adetomyrma* and *Xymmer*.

The palpal formula published for the *Adetomyrma* worker caste is 3:3, but some species are only known by the male caste, which, depending on the species, may present palpomere counts of 2:2 and 2:3 (Yoshimura and Fisher 2012a). The palpal formula for *Xymmer* males is 4:3/3:3/3:2 (Yoshimura and Fisher 2012b). Since published records indicate that the number of palpomeres is generally constant across castes of Amblyponinae species (Brown 1960), we expect the females of *Xymmer* and *Adetomyrma* to reflect a similarly diverse combination.

Finally, Yoshimura and Fisher (2012b) presented 4:3/4:2/3:3 as palpal formula for *Stigmatomma* males in the Malagasy region, differing from the numbers we counted for workers. However, mouthpart dissections on several male specimens of the same morphotypes used by Yoshimura & Fisher revealed that, for *Stigmatomma*, the number of palpomeres is the same in males and females (4:3/4:2; not evaluated for *S. besucheti*, as males are unknown).

**7.** The presence or absence of the metanotal suture, and the degree of its impression, may vary within species, as well as within nest series of *Stigmatomma* in the Malagasy region. Given this, we did not use those variations to isolate individual species.

**9.** The number of mesotibial spur(s) is difficult to determine under stereomicroscopes when the anterior spur is reduced in size, and also because the posterior spur may be “replaced” by an enlarged, stout spiniform seta. SEM images allowed comparisons between the texture of enlarged spinifom processes and surrounding cuticle, thus enabling us to differentiate spur and seta (Fig. 18​). 

In the *Stigmatomma* we studied, the number of mesotibial spurs ranged from zero to two, and were generally constant within species. In one species, *S. liebe* **sp. n.**, the number of mesotibial spurs visible under the stereomicroscope ranges from one to two. The anterior spur may be visible and developed, but it is vestigial in the majority of the specimens we evaluated. This variation was observed in specimens from the same nest series. 

Regarding other members of the XMMAS clade, *Stigmatomma pallipes* (ANTWEB1008501; Nearctic region), *S. pluto* (ANTWEB1008502), *Adetomyrma caputleae* Yoshimura and Fisher 2012a (ANTWEB1008494; Malagasy region), *Fulakora mystriops* (Brown 1960) (ANTWEB1008500; Neotropical region), *Myopopone castanea* (Smith 1860) (ANTWEB1008551; Indomalaya and Australasia regions), and *Xymmer muticus* Santschi 1914 (ANTWEB1008499; Afrotropical region) have two mesotibial spurs. *A. venatrix* Ward 1994 (Malagasy region) possesses one spur (Ward 1994), as well as *F. chilensis* (Mayr 1887) (ANTWEB1008496; Neotropical region) and *Mystrium voeltzkowi* Forel 1897 (ANTWEB1008554; Malagasy region). All *Xymmer* morphospecies from Madagascar evaluated under a stereomicroscope presented one spur/stout seta on the apex of the mesotibia. One species clearly seems to have a spur, while the others apparently present an enlarged stout seta.

Among the Amblyoponinae genera outside the XMMAS clade, ​*Amblyopone australis* Erichson 1842 (ANTWEB1008497; Australasia region), *A. mercovichi* Brown 1960 (ANTWEB1008498; Australasia region), and *Apomyrma stygia* Brown et al. 1971 (ANTWEB1008505) possess two mesotibial spurs. *Onychomyrmex* *doddi* Wheeler 1916 (ANTWEB1008560; Australasia region) possesses two vestigial spurs at the apex of the mesotibia. Each spur is a small, stout, conic seta totally or partially concealed by a fovea. *Prionopelta aethiopica* Arnold 1949 (ANTWEB1008580; Afrotropical region) and *P. antillana* Forel 1909 (ANTWEB1008581; Neotropical region) have one vestigial spur, while *P. concenta* (Brown 1974) (ANTWEB1008513; Afrotropical region) presents no spurs on the mesotibia.

**10.** We confirm the presence of a longitudinal sulcus on the antero­dorsal face of the mesobasitarsus in all species of *Stigmatomma* in the Malagasy region save *S. tsyhady* **sp. n.**

Within the XMMAS clade, this sulcus is present on the mesobasitarsus of *Stigmatomma pallipes* (ANTWEB1008501), *S. pluto* (ANTWEB1008502), *Adetomyrma caputleae* (ANTWEB1008494), *Fulakora chilensis* (ANTWEB1008496), *F. mystriops* (ANTWEB1008500), *Myopopone castanea* (ANTWEB1008551), and *Xymmer muticus* (ANTWEB1008499). We confirm present of this sulcus in only one *Xymmer* species in the Malagasy region. However, this character is difficult to visualize under a stereomicroscope when specimens are too small, as it occurs with *Xymmer* species, and its presence or absence may be better evaluated with an SEM microscope. This sulcus is absent in all *Mystrium* species we evaluated in the Malagasy region (CASENT0429914; CASENT0482698; CASENT0003281; CASENT0429897; CASENT0129838; CASENT0418314; CASENT0318933; CASENT0494274; CASENT0248701; CASENT0001158; ANTWEB1008554).

The sulcus on the anterior face of the mesobasitarsus is absent in *Amblyopone australis* (ANTWEB1008497), *A. mercovichi* (ANTWEB1008498), *Apomyrma stygia* (ANTWEB1008505), *Onychomyrmex doddi* (ANTWEB1008560), *Prionopelta aethiopica* (ANTWEB1008580), *P. antillana* (ANTWEB1008581), and *P. concenta* (ANTWEB1008513).

**11.** All *Stigmatomma* species present in the Malagasy bioregion present two well-developed metatibial spurs save *S. liebe* **sp. n.** In this species, the number of metatibial spurs visible under the stereomicroscope ranges from one to two. The anterior spur is visibly smaller than the posterior spur, and may be vestigial in some specimens. This variation was observed in specimens from the same nest series. A similar condition is found in *Onychomyrmex hedleyi* Emery 1895 (Australasia region). In this species, metatibial spurs are vestigial and may be present or absent in specimens from the same colony (Brown 1960).

In the XMMAS clade, *Stigmatomma pallipes* (ANTWEB1008501), *S. pluto* (ANTWEB1008502), *Adetomyrma caputleae* (ANTWEB1008494), *A. venatrix*, *Fulakora chilensis* (ANTWEB1008496), *F. mystriops* (ANTWEB1008500), *Myopopone castanea* (ANTWEB1008551), *Mystrium voeltzkowi* (ANTWEB1008554), and *Xymmer muticus* (ANTWEB1008499) possess two spurs on the metatibia.

*Amblyopone australis* (ANTWEB1008497), *A. mercovichi* (ANTWEB1008498), and *Apomyrma stygia* (ANTWEB1008505) possess two metatibial spurs. *Onychomyrmex doddi* (ANTWEB1008560) possesses two vestigial spurs at the apex of the metatibia. These spurs are small, stout, conic seta totally or partially concealed by a fovea. *Prionopelta aethiopica* (ANTWEB1008580) and *P. antillana* (ANTWEB1008581) have one spur, while *P. concenta* (ANTWEB1008513) presents no spurs.

**12.** Only one *Stigmatomma* species evaluated in this study (*S. roahady* **sp. n.**) presents a longitudinal sulcus on the anterior face of the metabasitarsus. The metabasitarsus of *S. besucheti*, while not presenting a sulcus on its anterior face, possesses two raised, parallel, not-­well­-developed longitudinal carinae with convergent apexes on its dorsal face.

This sulcus is present on the metabasitarsus of *Myopopone castanea* (ANTWEB1008551), and absent in *Stigmatomma pallipes* (ANTWEB1008501), *S. pluto* (ANTWEB1008502), *Adetomyrma caputleae* (ANTWEB1008494), *A. venatrix*, *Fulakora chilensis* (ANTWEB1008496), *F. mystriops* (ANTWEB1008500), and *Xymmer muticus* (ANTWEB1008499). It seems to be absent on the metabasitarsus of *Xymmer* in the Malagasy region. However, we cautiously affirm that, since this character is difficult to visualize under a stereomicroscope when specimens are too small, like those of *Xymmer*, it would be better evaluated under higher magnification. This sulcus is absent in all *Mystrium* species we evaluated in the Malagasy bioregion (CASENT0429914; CASENT0482698; CASENT0003281; CASENT0429897; CASENT0129838; CASENT0418314; CASENT0318933; CASENT0494274; CASENT0248701; CASENT0001158; ANTWEB1008554).

Among the Amblyoponinae genera that are not part of the XMMAS clade, the sulcus on the metabasitarsus is absent on *Amblyopone australis* (ANTWEB1008497), *A. mercovichi* (ANTWEB1008498), *Apomyrma stygia* (ANTWEB1008505), *Onychomyrmex doddi* (ANTWEB1008560), *Prionopelta aethiopica* (ANTWEB1008580), *P. antillana* (ANTWEB1008581), and *P. concenta* (ANTWEB1008513).

**13.** Arolium present on pro-, meso-, and metapretarsi in all species we studied. The same seems to apply to the other Amblyoponinae genera in the Malagasy region.

**14.** The petiole is sessile to sub­sessile in *Adetomyrma* and *Mystrium*; and subsessile to penduculate in *Xymmer* (Fig. 19). Also, within the XMMAS clade in the Malagasy region, *Xymmer* is the only genus in which the subpetiolar process is absent.

**15.** The constriction between pretergite and postergite of the abdominal segment III is scrobiculate in all *Stigmatomma* species but one. In *Adetomyrma* such a constriction is not visible; in *Xymmer* species the constriction is alveolate; and in *Mystrium* it is scrobiculate.

**16.** A prora is visible under a stereomicroscope in all *Stigmatomma* and *Mystrium* species in the Malagasy region; it seems to be absent in *Adetomyrma* and *Xymmer*.

**17.** *Adetomyrma* does not possess a constriction between the presclerite and postsclerite of abdominal segment IV. The constriction is scrobiculate in *Mystrium* and *Stigmatomma*, and alveolate in *Xymmer*.

**18.** Stout spiniform setae may be located on the apex of the hypopygium, surrounding the sting (Fig. 14). The number of setae varies from six to nine, when present in *Stigmatomma* species in the Malagasy region. This contradicts the opinion of Yoshimura and Fisher (2014), which states that the number of stout setae ranges from three to nine.

In the Malagasy region, all *Mystrium* species present two or four stout setae on the hypopygium (Yoshimura and Fisher 2014), while *Xymmer* and *Adetomyrma* have no such setae. Yoshimura and Fisher (2014) affirmed that two or four stout setae on the apex of the hypopygium are uniquely observed in Mystrium; however, Fulakora mystriops (ANTWEB1008500) also presents four stout setae on the hypopygium.

Stout spiniform setae are also present on the hypopygium of *Stigmatomma pluto* (twelve setae, ANTWEB1008502), *Fulakora chilensis* (eight setae, ANTWEB1008496), and in *F. cleae* (Lacau and Delabie 2002) and *F. agostii* (Lacau and Delabie 2002), which have ten setae each (both from the Neotropical region). These setae are absent in *S. pallipes* (ANTWEB1008501), the Neotropical *F. heraldoi* (Lacau and Delabie 2002), *Myopopone castanea* (ANTWEB1008551), *Xymmer muticus* (ANTWEB1008499), *Amblyopone australis* (ANTWEB1008497), *A. mercovichi* (ANTWEB1008498), *Apomyrma stygia* (ANTWEB1008505), *Onychomyrmex doddi* (ANTWEB1008560), *Prionopelta aethiopica* (ANTWEB1008580), and *P. concenta* (ANTWEB1008513).

##### Malagasy species-group *Stigmatomma*

We introduce a morphological organization system for the species diversity of *Stigmatomma* in the Malagasy bioregion which is based upon the definition of informal species-groups, which may contain species-complexes when necessary. Groups and complexes are named after the most abundant species, and the groups we presently define only reflect what is seen in the Malagasy fauna.

##### Synoptic list of Malagasy species

*besucheti* **group**

***besucheti*** (Baroni Urbani 1978) (Seychelles; Singapore?)

*tsyhady* **group**

*sakalava* **complex**

***bolabola*** Esteves & Fisher **sp. n.** (Madagascar)

***janovitsika*** Esteves & Fisher **sp. n.** (Seychelles)

***sakalava*** Esteves & Fisher **sp. n.** (Madagascar)

*tsyhady* **complex**

***irayhady*** Esteves & Fisher **sp. n.** (Madagascar)

***liebe*** Esteves & Fisher **sp. n.** (Madagascar)

***roahady*** Esteves & Fisher **sp. n.** (Madagascar)

***tsyhady*** Esteves & Fisher **sp. n.** (Madagascar)

##### *besucheti* species-group

*Stigmatomma besucheti* (Baroni Urbani 1978)

The morphology of *S. besucheti* isolates the species from other *Stigmatomma* in the Malagasy bioregion, and we place it in its own group based on the following worker characters (asterisks flag unique characters within the genus in the Malagasy bioregion):

1. * Ten antennomeres;

2. * Two maxillary palpomeres (palpal formula: 2:2);

3. * Calcar of strigil completely pectinate;

4. * Anterior face of the calcar of strigil with squamiform microtrichia basally;

5. * Posterior face of the calcar of strigil glabrous;

6. * Weakly raised longitudinal parallel carinae present on the dorsal face of metabasitarsus, with convergent apexes;

7. * Petiolar proprioceptor zone reduced to a small concavity.

##### *tsyhady* species-group

Workers with the following combination of characters (asterisks flag unique characters within the genus in the Malagasy bioregion):

1. * Twelve antennomeres;

2. * Four maxillary palpomeres (palpal formula: 4:3 or 4:2);

3. * Calcar of strigil not completely pectinate; baso­ventral lamella generally visible (in one species the lamella is reduced to a basal bud);

4. Anterior face of the calcar of strigil with strap­like or tubiform microtrichia basally;

5. * Posterior face of the calcar of strigil with lanceolate microtrichia;

6. Absence of any longitudinal carina on the dorsal face of metabasitarsus;

7. * Petiolar proprioceptor zone a large, round concavity.

This group can be split into subgroups based on morphological similarities, here called species complexes.

##### *sakalava* species-complex

*Stigmatomma bolabola* Esteves & Fisher, **sp. n.**

*Stigmatomma janovitsika* Esteves & Fisher, **sp. n.**

*Stigmatomma sakalava* Esteves & Fisher, **sp. n.**

Workers with the following combination of characters (character numbers are sequential to the species groups for sake of clarity in the character discussion):

8. Genal teeth present or absent;

9. Two labial palpomeres (palpal formula: 4:2);

10. Antler­-like microtrichia present on posterior face of posterior metatibial spur;

11. Absence of fenestra on the subpetiolar process;

12. Stout spiniform setae present on the apex of hypopygium.

##### *tsyhady* species-complex

*Stigmatomma irayhady* Esteves & Fisher, **sp. n.**

*Stigmatomma liebe* Esteves & Fisher, **sp. n.**

*Stigmatomma roahady* Esteves & Fisher, **sp. n.**

*Stigmatomma tsyhady* Esteves & Fisher, **sp. n.**

Workers with the following combination of characters (character numbers are sequential to the species groups for sake of clarity in the character discussion; asterisks flag unique characters within the genus in the Malagasy bioregion):

8. Genal teeth present;

9. * Three labial palpomeres (palpal formula: 4:3);

10. Posterior face of posterior metatibial spur mostly glabrous;

11. * Fenestra present on the subpetiolar process;

12. * Absence of stout spiniform setae on hypopygium.

##### Comments on species-groups and species-complexes characters

**3.** A reduced lamella on the baso­ventral margin of the calcar of strigil is often difficult to visualize under a stereomicroscope, and the calcar may appear completely pectinate while in reality it has a basal bud on the base of its ventral margin. Nonetheless, the proportion of lamellar tissue on the ventral margin of the calcar is constant within species, and was helpful to delimit certain species.

**4-5.** Presence and shape of microtrichia on anterior and posterior face of the calcar of strigil are not visible under a stereomicroscope. However, those characters are informative to diagnose groups of species.

**6.** The longitudinal parallel carinae on the dorsal face of the *Stigmatomma besucheti* metabasitarsus somewhat converge at their apexes; thus, the region between them appears groove-like in dorsal view. No other species of *Stigmatomma* in the Malagasy region presents such a character. However, one species, *S. roahady*, possesses a longitudinal sulcus on the anterior face of its metabasitarsus, and since its shape and location are different from the carinae on *S. besucheti*, we did not consider them homologous.

**10.** The presence and shape of microtrichia on the posterior face of the metatibial spur are not visible under a steromicroscope; however, it is informative to diagnose groups of species.

### Stigmatomma besucheti

(Baroni Urbani 1978)

Amblyopone besucheti Baroni Urbani 1978: 49, figs. 15, 16. Holotype (worker, CASENT0101816): SEYCHELLES: Ile de la Digue, 28.Jan.1975, P. Schauenberg leg. Paratypes: 9 workers; same data as holotype.
Stigmatomma besucheti Combination in *Stigmatomma*: Yoshimura and Fisher 2012b: 19.

#### Materials

- **Type status:** Holotype. **Occurrence:** catalogNumber: casent0101816; recordedBy: P. Schauenberg; sex: 1w; preparations: pin; associatedMedia: http://www.antweb.org/specimen/casent0101816; **Taxon:** scientificName: Stigmatomma besucheti; genus: Stigmatomma; **Location:** country: Seychelles; locality: Ile de la Digue; decimalLatitude: -4.359097; decimalLongitude: 55.841242; georeferenceRemarks: coordinates obtained from Google Earth; **Event:** eventDate: 01/28/1975; **Record Level:** institutionCode: MHNG
- **Type status:** Paratype. **Occurrence:** catalogNumber: casent0101900; recordedBy: P. Schauenberg; sex: 1w; preparations: pin; associatedMedia: http://www.antweb.org/specimen/casent0101900; **Taxon:** scientificName: Stigmatomma besucheti; genus: Stigmatomma; **Location:** country: Seychelles; locality: Ile de la Digue; decimalLatitude: -4.359097; decimalLongitude: 55.841242; georeferenceRemarks: coordinates obtained from Google Earth; **Event:** eventDate: 01/28/1975; **Record Level:** institutionCode: MHNG
- **Type status:** Paratype. **Occurrence:** catalogNumber: casent0101970; recordedBy: P. Schauenberg; sex: 1w; preparations: pin; associatedMedia: http://www.antweb.org/specimen/casent0101970; **Taxon:** scientificName: Stigmatomma besucheti; genus: Stigmatomma; **Location:** country: Seychelles; locality: Ile de la Digue; decimalLatitude: -4.359097; decimalLongitude: 55.841242; georeferenceRemarks: coordinates obtained from Google Earth; **Event:** eventDate: 01/28/1975; **Record Level:** institutionCode: MHNG
- **Type status:** Paratype. **Occurrence:** catalogNumber: casent0280650; recordedBy: P. Schauenberg; sex: 1w; preparations: pin; otherCatalogNumbers: BMNH(E)1017525; associatedMedia: http://www.antweb.org/specimen/casent0280650; **Taxon:** scientificName: Stigmatomma besucheti; genus: Stigmatomma; **Location:** country: Seychelles; locality: Ile de la Digue; decimalLatitude: -4.359097; decimalLongitude: 55.841242; georeferenceRemarks: coordinates obtained from Google Earth; **Event:** eventDate: 01/28/1975; **Record Level:** institutionCode: BMNH
- **Type status:** Paratype. **Occurrence:** catalogNumber: casent0906833; recordedBy: P. Schauenbert; sex: 1w; preparations: pin; associatedMedia: http://www.antweb.org/specimen/casent0906833; **Taxon:** scientificName: Stigmatomma besucheti; genus: Stigmatomma; **Location:** country: Seychelles; locality: Ile de la Digue; decimalLatitude: -4.359097; decimalLongitude: 55.841242; georeferenceRemarks: coordinates obtained from Google Earth; **Event:** eventDate: 01/28/1975; **Record Level:** institutionCode: NHMB
- **Type status:** Paratype. **Occurrence:** catalogNumber: casent0906834; recordedBy: P. Schauenberg; sex: 1w; preparations: pin; associatedMedia: http://www.antweb.org/specimen/casent0906834; **Taxon:** scientificName: Stigmatomma besucheti; genus: Stigmatomma; **Location:** country: Seychelles; locality: Ile de la Digue; decimalLatitude: -4.359097; decimalLongitude: 55.841242; georeferenceRemarks: coordinates obtained from Google Earth; **Event:** eventDate: 01/28/1975; **Record Level:** institutionCode: NHMB
- **Type status:** Paratype. **Occurrence:** catalogNumber: casent0906835; recordedBy: P. Schauenberg; sex: 1w; preparations: pin; associatedMedia: http://www.antweb.org/specimen/casent0906835; **Taxon:** scientificName: Stigmatomma besucheti; genus: Stigmatomma; **Location:** country: Seychelles; locality: Ile de la Digue; decimalLatitude: -4.359097; decimalLongitude: 55.841242; georeferenceRemarks: coordinates obtained from Google Earth; **Event:** eventDate: 01/28/1975; **Record Level:** institutionCode: NHMB
- **Type status:** Other material. **Occurrence:** catalogNumber: casent0172194; recordedBy: D.H.Murphy; sex: 1w; preparations: pin; otherCatalogNumbers: anic32-016286; associatedMedia: http://www.antweb.org/specimen/CASENT0172194; **Taxon:** scientificName: Stigmatomma cf. besucheti; genus: Stigmatomma; **Location:** country: Singapore; locality: Univ. Campus Singapore; (ANIC32-016286); decimalLatitude: 1.28333; decimalLongitude: 103.767; georeferenceRemarks: 10km; **Identification:** identifiedBy: R.W. Taylor; dateIdentified: 12/06/1978; **Event:** eventDate: 06/01/1964; habitat: Imperata grassland; eventRemarks: soil; **Record Level:** institutionCode: ANIC
- **Type status:** Other material. **Occurrence:** catalogNumber: casent0195513; recordedBy: D.H.Murphy; sex: 1dq; preparations: pin; otherCatalogNumbers: anic32-016287; associatedMedia: http://www.antweb.org/specimen/CASENT0195513; **Taxon:** scientificName: Stigmatomma cf. besucheti; genus: Stigmatomma; **Location:** country: Singapore; locality: Bukit Timah Nature Reserve; decimalLatitude: 1.38333; decimalLongitude: 103.8; georeferenceRemarks: 10km; **Identification:** identifiedBy: R.W. Taylor; dateIdentified: 12/07/1978; **Event:** eventDate: 10/04/1965; habitat: degraded coastal hill forest; eventRemarks: on grantite, berlesate No I24; **Record Level:** institutionCode: ANIC

#### Description

Worker (Fig. 20​): **HL**: 0.38-0.40; **HW**: 0.28-0.29; **HW2**: 0.26-0.27; **SL**: 0.18-0.19; **ML**: 0.20-0.22; **WL**: 0.41-0.43; **PPW**: 0.15-0.16; **PtL**: 0.14-0.15; **PtW**: 0.16-0.16; **CI**: 71.36-73.54; **SI**: 46.04-48.68; **MI**: 52.26-55.45; **PtI**: 90.00-92.50. 

*Head*:

Mandibular baso-masticatory margin skirted dorsally by row of filiform setae; medially, by flexuous tongue-like setae; ventrally, by flexuous filiform setae, grading into flexuous tongue-like setae apically (Fig. 21a, b). Mandibular dentition arrangement, from base to apex: two single teeth (same size of teeth arranged in pairs); four pairs of teeth (each pair with same dimensions, fused basally); single preapical tooth; apical tooth (Fig. 21a). Pair of teeth with similar dimensions along mandible's basoapical axis. Anterior clypeal margin with three cuticular processes arranged in a single row, armed anteriorly with asymmetrical mucronate dentiform seta; followed laterally by a notch on the anterior clypeal margin (Fig. 21a). Most lateral portion of anterior clypeal margin armed with row of conical setae arising from flat cuticle (or from reduced tubercle-like cuticular processes), decreasing in size laterad. Clypeal cuticular processes with approximately same length of associated dentiform setae. Long filiform setae pair on clypeal median area, posterior to central-most pair of cuticular processes on clypeal anterior margin. Shorter filiform pair of setae on clypeal median area, between longer pair of setae and frontal lobes. Median area of clypeus extending posteriorly between antennal sockets as narrow longitudinal strip; frontoclypeal sulcus acute (Fig. 21c). Supraclypeal area as small oblong depression (Fig. 21c). Ten antennomeres. Genal teeth absent. Compound eyes absent. Palpal formula: 2:2 (two maxillary, two labial; Fig. 21d​).

*Mesosoma*:

In dorsal view, mesonotum somewhat expanded laterally (Fig. 22a). Metanotal suture absent. Sulcus dividing mesepisternum into anepisternum and katepisternum; dorsoposterior corner of katepisternum not rounded (Fig. 22b). Metathoracic spiracle slit-like, surrounded by cuticular swell, projected posteriorly, inserted in a concavity. Propodeal spiracle round, surrounded by a cuticular swell. Propodeal declivitous face slightly concave (Fig. 22a).

*Legs*:

Absence of lamella on basoventral margin of calcar of strigil (Fig. 23a). ​Calcar of strigil anterior face with squamiform microtrichia basally (Fig. 23a); posterior face mostly glabrous (Fig. 23b). Multiple paddle-like setae on anteroventral face of protibial apex, next to ​calcar of strigil. Multiple paddle-like setae on anterior face of probasitarsus; row of stout setae along posterior face, next to comb of strigil. Mesotibial spur absent; apex of mesotibial inner face with deep fovea (Fig. 23c). Slit-like longitudinal sulcus on anterodorsal face of mesobasitarsus, with apical end projected laterally (Fig. 23c). Two metatibial spurs; anterior spur simple with lanceolate microtrichia; posterior spur pectinate (Fig. 23d). Anterior face of posterior metatibial spur glabrous (Fig. 23d); posterior face with antler-like microtrichia dorsoapically (Fig. 23e). Dorsal face of metabasitarsus with two parallel carinae with convergent apexes (Fig. 23f). Brush of few stout paddle-like setae on baso-inner face of metabasitarsus (Fig. 23e). Arolium on pro-, meso-, and metapretarsus.

*Metasoma*:

Petiole sessile (Fig. 24a). Ventroanterior margin of petiolar tergite anterior dorso-latero-ventral carina (Ward 1990) 2x the size of anterior margin of subpetiolar process, in lateral view (Fig. 24a). Subpetiolar process with obtuse angle at midpoint of its ventral margin (fin-like; Fig. 24a). Absence of fenestra on lateral face of subpetiolar process. Petiolar proprioceptor zone reduced to small round concavity bearing few sensilla (Fig. 24b​). Prora present (Fig. 24c). Smooth sulcus between pretergite and postergite of abdominal segment III; weakly scrobiculate sulci between presclerites and postsclerites of abdominal segment IV (Fig. 24c). Five to six stout spiniform setae on apex of hypopygium (Fig. 24d).

*Sculpture*:

Mandibular dorsal face costate-slightly catenate basally, grading into costate apically except for smooth apical portion (Fig. 21a). Clypeal median area smooth, grading into costate to rugose laterally (Fig. 21a). Supraclypeal area smooth (Fig. 21c). Head, in dorsal view, taeniate-catenate; area posterior to tentorial pit plicate (Fig. 21c). Labrum weakly imbricate (Fig. 25a). Mesosoma in dorsal view and lateral face of pronotum foveolate (Fig. 22). Anepisternum smooth; katepisternum mostly imbricate; metapleuron costate posteriorly; lateral face of propodeum smooth, grading into imbricate posteroventrally (Fig. 22b); declivitous face of propodeum smooth (Fig. 22a). Petiolar tergite imbricate ventroanteriorly, grading into weakly and scarcely foveate dorsally and posteriorly (Figs 24a, 25b). Petiolar laterotergite imbricate; petiolar poststernite mostly areolate, grading into smooth ventrally (Fig. 24b). Most of gaster weakly foveolate (Fig. 25c).

*Pilosity and color*:

Suberect pilosity on head, dorsal face of mesosoma, lateral face of pronotum, petiolar tergite, and abdominal segments III, IV, V, and VI. Legs densely covered by subdecumbent pilosity. Suberect pilosity on anterior half of petiolar poststernite. Longer pilosity on abdominal segment VII. Body color yellow.

##### Comments on character variation

Character variation among specimens was minimal.

##### Other castes

Unknown for the Malagasy region. However, a gyne of a putative *Stigmatomma besucheti* is known for Singapore, as presented below.

#### Diagnosis

With characters of the *besucheti* species-group as described above and the following characters (asterisks flag unique characters within *Stigmatomma* in the Malagasy bioregion):

1. Integument yellow; small-sized ant (HL: 0.38-0.40, WL: 0.41-0.43; Fig. 20).
2. Pairs of teeth of mandible’s baso-masticatory margin the same size along mandible’s basoapical axis (Fig. 21a).
* Tongue-like setae medially inserted on mandible’s baso-mastigatory margin (Fig. 21b).
* Dorsal face of the head densely taeniate-catenate (Fig. 21c).
5. Genal teeth absent.
* Palpal formula 2:2 (Fig. 21d).
7. Dorsal face of mesosoma and lateral face of propotum foveolate; declivitous face of propodeum smooth; lateral face of propodeum mostly smooth (Fig. 22).
8. Mesepisternum divided by a sulcus into anepisternum and katepisternum (Fig. 22b).
* Mesotibial spur absent (Fig. 23c).
* Apex of mesotibial inner face bearing a deep fovea; absence of any enlarged process ressembling a spur (Fig. 23c).
11. Slit-like sulcus present on the anterior face of mesobasitarsus, with apical end projected laterally (Fig. 23c).
12. Anterior face of posterior metatibial spur glabrous; posterior face with antler-like microtrichia dorsoapically (Fig. 23d, e).
13. Brush of few stout, paddle-like setae present on the baso-inner face of metabasitarsus.
14. Absence of fenestra on lateral face of subpetiolar process (Fig. 24a).
15. Subpetiolar process fin-like: half of its ventral margin obtusely angled.
16. Presence of 5-6 stout spiniform setae on apex of hypopygium (Fig. 24d).

In the Malagasy bioregion, *Stigmatomma besucheti* is unique and easily recognized by: reduced number of antennomeres, palpal formula, head sculpture, absence of any enlarged process ressembling a spur on the apex of the mesotibia, petiolar proprioceptor zone reduced to a small concavity, and small body size. Further, it does not occur in sympatry with any other congener.

#### Distribution

**Figure 26.**
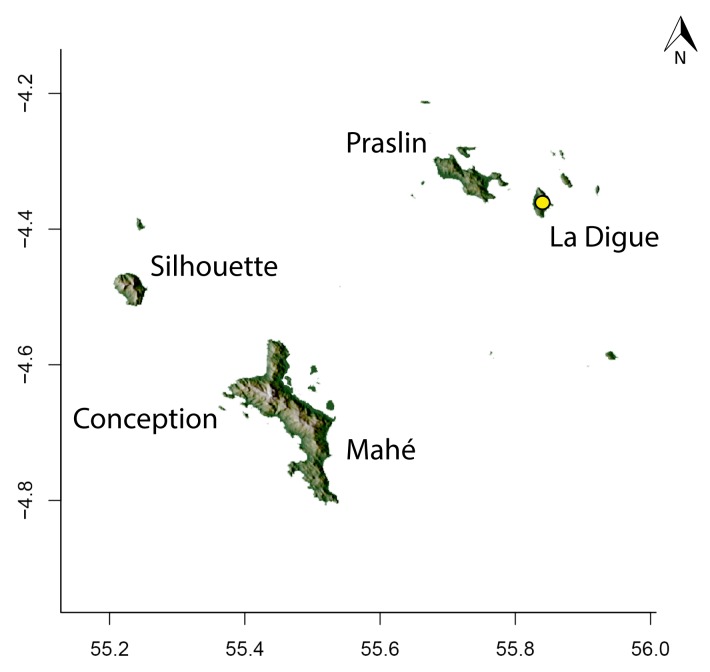
Distribution map of *Stigmatomma besucheti* in the Seychelles.

*Stigmatomma besucheti* is known by its type series, collected in Seychelles by Schauenberg in 1975 (Fig. 26), and two specimens collected in Singapore.

While no direct information about habitat/microhabitat exists, published records of other organisms collected by Schauenberg on La Digue island on 28.Jan.1975 indicate the type series of *S. besucheti* was probably extracted from soil samples submitted to Berlese funnels (Mahunka 1978b, Mahunka 1978a).

The two specimens from Singapore (a worker and a queen; CASENT0172194 and CASENT0195513, respectively) share remarkable similarities with the type specimens (*e.g*., antennomeres, general body shape and size, sculpture on the head, lack of any spiniform process on the mesotibial inner apex). However, those specimens differ in the following characters (corresponding characters of type specimens are presented within parentheses):

1. Three mandibular pairs of teeth (four pairs of teeth) (Fig. 27a).
2. The posterior-most pair of long filiform setae on the clypeus of the Singapore specimens is much longer (Fig. 27a).
3. Supraclypeal area as a longer oblong depression (small oblong depression) (Fig. 27a).
4. Anterior face of posterior metatibial spur glabrous; posterior face glabrous (anterior face glabrous; posterior face with antler-like microtrichia dorsoapically) (Fig. 27b).

Despite these differences, we did not examine enough specimens of each form to evaluate character variation, and therefore cannot affirm they are different species. Also, while it seems improbable that a specialized predator would become an exotic species, it is noteworthy that: (1) the putative prey of *Stigmatomma besucheti* (i.e., geophilomorph centipedes) are widespread around the world and a major component of soil ecosystems (Bonato et al. 2013); and (2) global trade is known to profoundly influence the movement of species around the world (McNeely et al. 2001), and plant seeds and other organisms were inadvertently dispersed in the soil used as ballast in early shipping (McNeely et al. 2001). The Seychelles have historically occupied a strategic position along Indian Ocean trade routes, providing coaling/fuelling stations for ships bringing goods from the East to Europe and North America (Ofcansky 1995).

In addition to the specimens collected in Singapore, there is single specimen collected in Sabah (Borneo; CASENT0235146) that resembles *Stigmatomma besucheti* in the number of antennomeres, head sculpture, mandible and clypeal configuration, size, and color, but differs in some significant characters of the petiole. Compared to *S. besucheti*, the anteroventral margin of petiolar tergite anterior dorso-latero-ventral carina is much shorter, and the shape of the subpetiolar process is different. Unfortunately, specimen CASENT0235146 was previously submitted to DNA extraction (Ward and Fisher 2016), and is too fragile for a thorough examination.

### Stigmatomma bolabola

Esteves & Fisher sp. n.

urn:lsid:zoobank.org:act:05D881B6-3EBA-4D5D-863F-135430794C5D

#### Materials

- **Type status:** Holotype. **Occurrence:** catalogNumber: casent0034580; recordedBy: Fisher, Griswold et al.; sex: 1w; preparations: pin; associatedMedia: http://www.antweb.org/specimen/casent0034580; **Taxon:** scientificName: Stigmatomma bolabola; genus: Stigmatomma; **Location:** country: Madagascar; locality: Montagne d'Akirindro 7.6 km 341° NNW Ambinanitelo; decimalLatitude: -15.28833; decimalLongitude: 49.54833; georeferenceRemarks: coordinates obtained from GPS; **Event:** eventDate: 03/17/2003; habitat: rainforest; fieldNumber: BLF08250; eventRemarks: sifted litter (leaf mold, rotten wood); **Record Level:** institutionCode: CASC
- **Type status:** Other material. **Occurrence:** catalogNumber: casent0034744; recordedBy: Fisher, Griswold et al.; sex: 1w; preparations: pin; associatedMedia: http://www.antweb.org/specimen/casent0034744; **Taxon:** scientificName: Stigmatomma bolabola; genus: Stigmatomma; **Location:** country: Madagascar; locality: Montagne d'Anjanaharibe, 19.5 km 27° NNE Ambinanitelo; decimalLatitude: -15.17833; decimalLongitude: 49.635; georeferenceRemarks: coordinates obtained from GPS; **Event:** eventDate: 03/12/2003; habitat: montane rainforest; fieldNumber: BLF08150; eventRemarks: sifted litter (leaf mold, rotten wood); **Record Level:** institutionCode: CASC

#### Description

Worker (Fig. 28); only the holotype was measured): **HL**: 0.76; **HW**: 0.59; **HW2**: 0.49; **SL**: 0.45; **ML**: 0.41; **WL**: 0.92; **PPW**: 0.34; **PtL**: 0.29; **PtW**: 0.41; **CI**: 78; **SI**: 60; **MI**: 54; **PtI**: 72. 

*Head*:

Mandibular baso-masticatory margin skirted dorsally by row of filiform setae; ventrally, by acuminate flattened-apex setae, and row of longer filiform setae (Fig. 29a). Mandibular dentition arrangement, from base to apex: row of three single teeth (same dimensions of teeth arranged in pairs); three pairs of teeth (each teeth pair fused basally); single preapical tooth; apical tooth (Fig. 29a). Dorsal teeth pairs increasing in length towards mandibular apex. Anterior clypeal margin with nine tubercle-like cuticular processes arranged in a single row. All clypeal cuticular processes, except the most lateral, armed anteriorly with truncated dentiform seta; most lateral processes smaller and unarmed (Fig. 29a). Median clypeal cuticular processes around 3x the length of associated dentiform setae. Pair of long filiform setae on clypeal median area, posterior to tubercle-like cuticular processes on clypeal anterior margin. Pair of shorter filiform setae on clypeal median area posterior to longer setae pair, followed posteriorly by longitudinal row of much shorter filiform setae. Clypeal median area extending posteriorly between antennal sockets as narrow longitudinal strip; frontoclypeal sulcus acute. Supraclypeal area as oval shaped concavity (Fig. 29b). Twelve antennomeres. Genal teeth absent (Fig. 29b). Widest diameter of compound eyes: 2-3 ommatidia (Fig. 29c). Palpal formula: 4:2 (four maxillary, two labial; Fig. 29d).

*Mesosoma*:

In dorsal view, mesonotum narrower than remaining mesosoma (Fig. 30a). Metanotal suture absent (Fig. 30a). Sulcus divinding mesepisternum into anepisternum and katepisternum; posterodorsal corner of katepisternum rounded (Fig. 30b). Metathoracic spiracle slit-like, reduced in size (Fig. 30b). Propodeal spiracle round, surrounded by cuticular swell, followed by sulcus (Fig. 30b). Propodeal declivitous face not concave.

*Legs*:

Basoventral fifth of calcar of strigil lamellar (Fig. 31b). Calcar of strigil anterior face with tubiform microtrichia (Fig. 31a); posterior face with lanceolate microtrichia (Fig. 31b). Multiple paddle-like setae on anteroventral face of protibial apex, next to calcar of strigil (Fig. 31a). Multiple paddle-like setae on anterior face of probasitarsus (Fig. 31a); stout setae on apex of posterior face (Fig. 31b). Single mesotibial spur with lanceolate microtrichia (Fig. 31c). Slit-like longitudinal sulcus on anterodorsal face of mesobasitarsus (Fig. 31d). Row of stout setae along inner face of mesobasitarsus (Fig. 31c). Two metatibial spurs; simple anterior spur with lanceolate microtrichia; posterior spur pectinate (Fig. 31e). Anterior face of posterior metatibial spur glabrous (Fig. 31e); posterior face with antler-like microtrichia. Brush of long, truncated filiform setae on posterior face of metatibial apex, next to posterior metatibial spur. Absence of longitudinal sulcus on anterodorsal face of metabasitarsus (Fig. 31e). Brush of tubiform setae on baso-inner face of metabasitarsus. Stout setae on remaining inner face of metabasitarsus. Arolium on pro-, meso-, and metapretarsus.

*Metasoma*:

Petiole sessile (Fig. 32a). Ventroanterior margin of petiolar tergite anterior dorso-latero-ventral carina (Ward 1990) much shorter than anterior margin of subpetiolar process, in lateral view. Subpetiolar process with obtuse angle at mid-point of its ventral margin (fin-like; Fig. 32a). Absence of fenestra on lateral face of subpetiolar process (Fig. 32a). Petiolar proprioceptor zone a large, round concavity with few sensilla (Fig. 32b). Prora present (Fig. 32a). Scrobiculate sulcus between pretergite and postergite of abdominal segment III and presclerites and postsclerites of abdominal segment IV (Fig. 32c). Eight stout spiniform setae on apex of hypopygium (Fig. 32d).

*Sculpture*:

Mandibular dorsal face rugose-foveate basally, grading into costate-foveolate apically except for smooth apical portion (Fig. 29a). Clypeal median area costate. Supraclypeal area smooth. Head, in dorsal view, mostly foveate-reticulate/densely foveate; area posterior to tentorial pit plicate (Fig. 29b). Labrum rugose (Fig. 33a). Mesosoma foveate dorsally (Fig. 30a). Pronotum rugulose-foveate laterally (Fig. 33b). Anepisternum scarcely costulate; katepisternum costate-rugulose (Fig. 30b). Metapleuron mostly costate (Fig. 30b). Lateral face of propodeum costate-foveolate; declivitous face strigate (Fig. 30). Petiolar tergite, in lateral view, areolate/imbricate ventroanteriorly, grading into strigate dorsoanteriorly, costate laterally, foveate dorsally (Fig. 33c). Petiolar laterotergite imbricate posteriorly (Fig. 32b). Petiolar poststernite mostly alveolate, grading into smooth ventrally (Fig. 32b). Abdominal segment III foveolate; remaining gaster puncticulate (Fig. 32c).

*Pilosity and color*:

Suberect pilosity on head, dorsal face of mesosoma, lateral face of propodeum, petiolar tergite, and abdominal segments III and IV. Petiolar poststernite mostly glabrous. Longer pilosity on abdominal segments V, VI and VII. Body color red-brown; apex of gaster and appendages orange-yellow.

##### Comments on character variation

Character variation on the specimens examined was minimal.

##### Other castes

Unknown.

##### Specimens used in prior studies

This taxon was referenced as *Stigmatomma* MG03 (specimen CASENT0034580) in Ward and Fisher (2016).

#### Diagnosis

*Worker*

With characters of the *tsyhady* species-group and the *sakalava* species-complex as described above, and the following characters (asterisks flag unique characters within the genus in the Malagasy bioregion):

1. Integument red-brown; medium-sized ant (HL: 0.76, WL: 0.92; Fig. 28).
2. Dorsal teeth row of mandible’s pairs of teeth increasing in size towards mandibular apex (Fig. 29a).
3. Row of acuminate flattened-apex setae setae ventrally skirting mandible’s baso-masticatory margin, parallel to row of flexuous, longer setae (Figs 29a, 33a).
4. Dorsal face of the head mostly foveate-reticulate/densely foveate (Figs 28a, 29b).
5. Genal teeth absent (Figs 28a, 29b).
6. Palpal formula 4:2 (Fig. 29d).
7. Dorsal face of mesosoma foveate; lateral face of pronotum rugulose-foveate; lateral face of propodeum costate-foveolate (Figs 30, 33b).
* Declivitous face of propodeum strigate (Fig. 30a).
* Mesepisternum divided into anepisternum and katepisternum; posterodorsal corner of katepisternum rounded (Fig. 30b).
* Basoventral one-fifth of calcar of strigil lamellar (Fig. 31b).
11. Calcar of strigil anterior face with tubiform microtrichia (Fig. 31a).
12. Single mesotibial spur covered with lanceolate microtrichia (Fig. 31c).
13. Longitudinal slit-like sulcus present on the anterodorsal face of mesobasitarsus (Fig. 31d).
14. Anterior face of posterior metatibial spur glabrous; posterior face with antler-like microtrichia (Fig. 31e).
15. Brush of long truncated-apex filiform setae present on the posterior face of metatibial apex.
16. Brush of tubiform setae present on the baso-inner face of metabasitarsus.
17. Absence of sulcus on metabasitarsus (Fig. 31e).
18. Subpetiolar process fin-like: half of its ventral margin obtusely angled (Fig. 32a).
19. Eight stout spiniform setae present on the apex of hypopygium (Fig. 32d).

*Stigmatomma bolabola* may be confounded with *S. sakalava* by the following characters: absence of genal teeth, palpal formula, single mesotibial spur, head sculpture, shape of subpetiolar process, and presence of stout spiniform setae on the apex of hypopygium. However, it is easily recognized by the sculpture of its mesosoma lateral face and propodeal declivitous face, katepisternum shape, proportion of lamella on the basoventral margin of calcar of strigil, and distribution (since they do not occur in sympatry).

#### Etymology

Bola-­bola is the name that Malagasy people give to logs of rosewood, a plant of the genus *Dalbergia* (Schuurman 2009). *Stigmatomma bolabola* is named for the resemblance between its color and that of rosewood timber, and because it lives in the region of Madagascar most affected by illegal rosewood logging.

Madagascar is home to 48 species of rosewood, of which 47 are endemic (Barrett et al. 2010). Of the 20 endangered species and 15 vulnerable species of rosewood, about 16 species are unsustainably exploited for their timber and known locally as bois de rose (IUCN 2015).

Due to international demand for rosewood, thousands of loggers have flooded into the national parks of Madagascar. In the process of extraction, new roads are built, and logging camps set up, which increases access to forests, fuels extraction of other resources, and accelerates general deforestation, illegal mining, and poaching (Barrett et al. 2010, Patel 2007). That is why, beyond the reduction or extinction of rare trees, rosewood logging is inextricably linked to reduction of native species diversity, invasion of non­-native species, and landscape aridification (Patel 2007). Unfortunately, due to the low density of rosewood trees in the wild, loggers routinely search for new territories, initiating a new cycle of destruction (Barrett et al. 2010).

The highest rosewood species richness in Madagascar is found in the northeastern rainforest, with seven species native to the SAVA Region and the Makira-­Masoala Landscape (Barrett et al. 2010). There, the protected areas are at high risk of logging from a lack of law enforcement, and the higher quality, size, and density per hectare of the rosewood (Barrett et al. 2010, Schuurman 2009).

The Makira component of the Makira-­Masoala Landscape (Makira Forest Protected Area) is the only place in Madagascar where *Stigmatomma bolabola* has been found, in a collection effort encompassing more than 440 collection sites. Given that the health of the ecosystem is essential to protect *S. bolabola* habitat, here we plead for more effective protection of Malagasy rosewood.

#### Distribution

**Figure 34.**
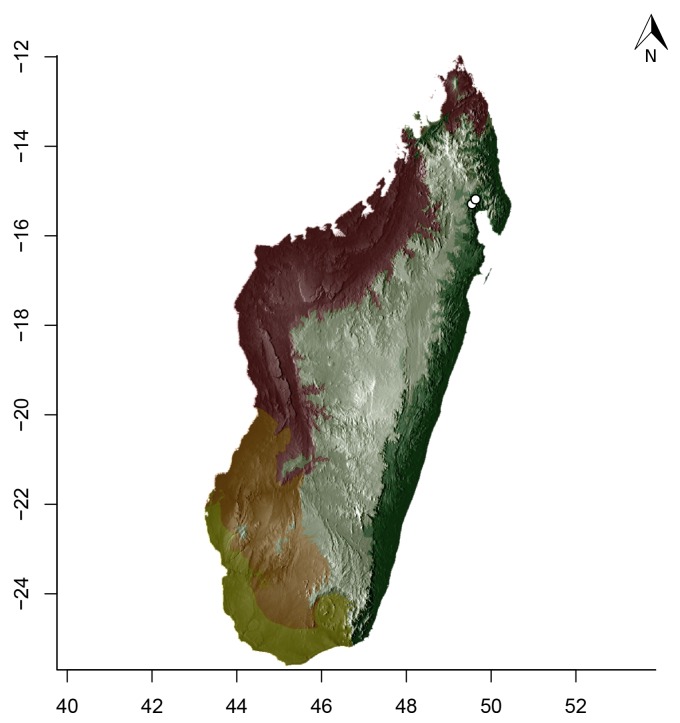
Distribution map of *Stigmatomma bolabola* ​**sp. n.** in the Malagasy bioregion. Collection localities are mapped over the outlines of five simplified ecoregion zones of Madagascar: humid forests (dark green), subhumid forests (light green), dry deciduous forests (brown), succulent woodlands (orange), and spiny thickets (yellow).

*Stigmatomma bolabola* was collected in two localities of the Makira Forest Protected Area, in rainforest and montane rainforest habitats in the humid forests ecoregion of Madagascar (at 600 m and 1100 m respectively; following the classification of Burgess et al. 2004; Fig. 34). Specimens were recorded from sifted leaf mold and rotten wood.

### Stigmatomma irayhady

Esteves & Fisher sp. n.

urn:lsid:zoobank.org:act:89E7025A-101A-4DDB-A65A-A33920E95554

#### Materials

- **Type status:** Holotype. **Occurrence:** catalogNumber: casent0042899; recordedBy: B.L.Fisher; sex: 1w; preparations: pin; associatedMedia: http://www.antweb.org/specimen/casent0042899; **Taxon:** scientificName: Stigmatomma irayhady; genus: Stigmatomma; **Location:** country: Madagascar; stateProvince: Antsiranana; locality: Forêt de Binara, 9.4km 235° SW Daraina; verbatimElevation: 1100; decimalLatitude: -13.26333; decimalLongitude: 49.6; georeferenceRemarks: coordinates obtained from GPS; **Event:** samplingProtocol: MW 25 sample transect, 5m; eventDate: 12/05/2003; habitat: montane rainforest; fieldNumber: BLF09800; eventRemarks: sifted litter (leaf mold, rotten wood); **Record Level:** institutionCode: CASC
- **Type status:** Paratype. **Occurrence:** catalogNumber: casent0042843; recordedBy: B.L.Fisher; sex: 1w; preparations: pin; associatedMedia: http://www.antweb.org/specimen/casent0042843; **Taxon:** scientificName: Stigmatomma irayhady; genus: Stigmatomma; **Location:** country: Madagascar; stateProvince: Antsiranana; locality: Forêt de Binara, 9.4km 235° SW Daraina; verbatimElevation: 1100; decimalLatitude: -13.26333; decimalLongitude: 49.6; georeferenceRemarks: coordinates obtained from GPS; **Event:** samplingProtocol: MW 25 sample transect, 5m; eventDate: 12/05/2003; habitat: montane rainforest; fieldNumber: BLF09800; eventRemarks: sifted litter (leaf mold, rotten wood); **Record Level:** institutionCode: CASC
- **Type status:** Paratype. **Occurrence:** catalogNumber: casent0042845; recordedBy: B.L.Fisher; sex: 1dQ; preparations: pin; associatedMedia: http://www.antweb.org/specimen/casent0042845; **Taxon:** scientificName: Stigmatomma irayhady; genus: Stigmatomma; **Location:** country: Madagascar; stateProvince: Antsiranana; locality: Forêt de Binara, 9.4km 235° SW Daraina; verbatimElevation: 1100; decimalLatitude: -13.26333; decimalLongitude: 49.6; georeferenceRemarks: coordinates obtained from GPS; **Event:** samplingProtocol: MW 25 sample transect, 5m; eventDate: 12/05/2003; habitat: montane rainforest; fieldNumber: BLF09800; eventRemarks: sifted litter (leaf mold, rotten wood); **Record Level:** institutionCode: CASC
- **Type status:** Paratype. **Occurrence:** catalogNumber: casent0042847; recordedBy: B.L.Fisher; sex: 1dQ; preparations: pin; associatedMedia: http://www.antweb.org/specimen/casent0042847; **Taxon:** scientificName: Stigmatomma irayhady; genus: Stigmatomma; **Location:** country: Madagascar; stateProvince: Antsiranana; locality: Forêt de Binara, 9.4km 235° SW Daraina; verbatimElevation: 1100; decimalLatitude: -13.26333; decimalLongitude: 49.6; georeferenceRemarks: coordinates obtained from GPS; **Event:** samplingProtocol: MW 25 sample transect, 5m; eventDate: 12/05/2003; habitat: montane rainforest; fieldNumber: BLF09800; eventRemarks: sifted litter (leaf mold, rotten wood); **Record Level:** institutionCode: CASC
- **Type status:** Paratype. **Occurrence:** catalogNumber: casent0797614; recordedBy: B.L.Fisher; sex: 1w; preparations: pin; associatedMedia: http://www.antweb.org/specimen/casent0797614; **Taxon:** scientificName: Stigmatomma irayhady; genus: Stigmatomma; **Location:** country: Madagascar; stateProvince: Antsiranana; locality: Forêt de Binara, 9.4km 235° SW Daraina; verbatimElevation: 1100; decimalLatitude: -13.26333; decimalLongitude: 49.6; georeferenceRemarks: coordinates obtained from GPS; **Event:** samplingProtocol: MW 25 sample transect, 5m; eventDate: 12/05/2003; habitat: montane rainforest; fieldNumber: BLF09800; eventRemarks: sifted litter (leaf mold, rotten wood); **Record Level:** institutionCode: BMNH
- **Type status:** Paratype. **Occurrence:** catalogNumber: casent0042898; recordedBy: B.L.Fisher; sex: 1w; preparations: pin; associatedMedia: http://www.antweb.org/specimen/casent0042898; **Taxon:** scientificName: Stigmatomma irayhady; genus: Stigmatomma; **Location:** country: Madagascar; stateProvince: Antsiranana; locality: Forêt de Binara, 9.4km 235° SW Daraina; verbatimElevation: 1100; decimalLatitude: -13.26333; decimalLongitude: 49.6; georeferenceRemarks: coordinates obtained from GPS; **Event:** samplingProtocol: MW 25 sample transect, 5m; eventDate: 12/05/2003; habitat: montane rainforest; fieldNumber: BLF09800; eventRemarks: sifted litter (leaf mold, rotten wood); **Record Level:** institutionCode: MHNG
- **Type status:** Paratype. **Occurrence:** catalogNumber: casent0042842; recordedBy: B.L.Fisher; sex: 1w; preparations: pin; associatedMedia: http://www.antweb.org/specimen/casent0042842; **Taxon:** scientificName: Stigmatomma irayhady; genus: Stigmatomma; **Location:** country: Madagascar; stateProvince: Antsiranana; locality: Forêt de Binara, 9.4km 235° SW Daraina; verbatimElevation: 1100; decimalLatitude: -13.26333; decimalLongitude: 49.6; georeferenceRemarks: coordinates obtained from GPS; **Event:** samplingProtocol: MW 25 sample transect, 5m; eventDate: 12/05/2003; habitat: montane rainforest; fieldNumber: BLF09800; eventRemarks: sifted litter (leaf mold, rotten wood); **Record Level:** institutionCode: NHMB
- **Type status:** Other material. **Occurrence:** catalogNumber: casent0369789; recordedBy: B.L.Fisher et al.; sex: 1w; preparations: pin; associatedMedia: http://www.antweb.org/specimen/casent0369789; **Taxon:** scientificName: Stigmatomma irayhady; genus: Stigmatomma; **Location:** country: Madagascar; stateProvince: Antsiranana; locality: Binara Forest; verbatimElevation: 1065; decimalLatitude: -13.26392; decimalLongitude: 49.59919; georeferenceRemarks: ±500m; **Event:** samplingProtocol: 3 MaxiWinks, mixed samples; eventDate: 10/18/2013; habitat: rainforest; fieldNumber: BLF32140; eventRemarks: sifted litter; **Record Level:** institutionCode: CASC
- **Type status:** Other material. **Occurrence:** catalogNumber: casent0370139; recordedBy: B.L.Fisher et al.; sex: 1w; preparations: pin; associatedMedia: http://www.antweb.org/specimen/casent0370139; **Taxon:** scientificName: Stigmatomma irayhady; genus: Stigmatomma; **Location:** country: Madagascar; stateProvince: Antsiranana; locality: Binara Forest; verbatimElevation: 1065; decimalLatitude: -13.26392; decimalLongitude: 49.59919; georeferenceRemarks: ±500m; **Event:** samplingProtocol: General collection; eventDate: 10/18/2013; habitat: rainforest; fieldNumber: BLF32151; eventRemarks: ex soil; **Record Level:** institutionCode: CASC
- **Type status:** Other material. **Occurrence:** catalogNumber: casent0371016; recordedBy: B.L.Fisher et al.; sex: 1w; preparations: pin; associatedMedia: http://www.antweb.org/specimen/casent0371016; **Taxon:** scientificName: Stigmatomma irayhady; genus: Stigmatomma; **Location:** country: Madagascar; stateProvince: Antsiranana; locality: Binara Forest; verbatimElevation: 1065; decimalLatitude: -13.26392; decimalLongitude: 49.59919; georeferenceRemarks: ±500m; **Event:** samplingProtocol: General collection; eventDate: 10/19/2013; habitat: rainforest; fieldNumber: BLF32179; eventRemarks: ex soil; **Record Level:** institutionCode: CASC
- **Type status:** Other material. **Occurrence:** catalogNumber: casent0410412; recordedBy: Fisher, Griswold et al.; sex: 1w; preparations: pin; associatedMedia: http://www.antweb.org/specimen/casent0410412; **Taxon:** scientificName: Stigmatomma irayhady; genus: Stigmatomma; **Location:** country: Madagascar; stateProvince: Antananarivo; locality: 3 km 41° NE Andranomay, 11.5 km 147° SSE Anjozorobe; verbatimElevation: 1300; decimalLatitude: -18.47333; decimalLongitude: 47.96; georeferenceRemarks: coordinates obtained from GPS; **Event:** samplingProtocol: MW 50 sample transect, 5m; eventDate: 12/05/2000; habitat: montane rainforest; fieldNumber: BLF02378; eventRemarks: sifted litter (leaf mold, rotten wood); **Record Level:** institutionCode: CASC
- **Type status:** Other material. **Occurrence:** catalogNumber: casent0410413; recordedBy: Fisher, Griswold et al.; sex: 1w; preparations: pin; associatedMedia: http://www.antweb.org/specimen/casent0410413; **Taxon:** scientificName: Stigmatomma irayhady; genus: Stigmatomma; **Location:** country: Madagascar; stateProvince: Antananarivo; locality: 3 km 41° NE Andranomay, 11.5 km 147° SSE Anjozorobe; verbatimElevation: 1300; decimalLatitude: -18.47333; decimalLongitude: 47.96; georeferenceRemarks: coordinates obtained from GPS; **Event:** samplingProtocol: MW 50 sample transect, 5m; eventDate: 12/05/2000; habitat: montane rainforest; fieldNumber: BLF02378; eventRemarks: sifted litter (leaf mold, rotten wood); **Record Level:** institutionCode: CASC
- **Type status:** Other material. **Occurrence:** catalogNumber: casent0458588; recordedBy: Fisher, Griswold et al.; sex: 1w; preparations: pin; associatedMedia: http://www.antweb.org/specimen/casent0458588; **Taxon:** scientificName: Stigmatomma irayhady; genus: Stigmatomma; **Location:** country: Madagascar; stateProvince: Antananarivo; locality: Réserve Spéciale d'Ambohitantely, Forêt d Ambohitantely, Jardin Botanique, 24.1km 59° NE d Ankazobe; verbatimElevation: 1620; decimalLatitude: -18.17139; decimalLongitude: 47.28182; georeferenceRemarks: coordinates obtained from GPS; **Event:** samplingProtocol: MW 50 sample transect, 5m; eventDate: 04/17/2001; habitat: montane rainforest; fieldNumber: BLF03720; eventRemarks: sifted litter (leaf mold, rotten wood); **Record Level:** institutionCode: CASC
- **Type status:** Other material. **Occurrence:** catalogNumber: casent0458589; recordedBy: Fisher, Griswold et al.; sex: 1dQ; preparations: pin; associatedMedia: http://www.antweb.org/specimen/casent0458589; **Taxon:** scientificName: Stigmatomma irayhady; genus: Stigmatomma; **Location:** country: Madagascar; stateProvince: Antananarivo; locality: Réserve Spéciale d'Ambohitantely, Forêt d Ambohitantely, Jardin Botanique, 24.1km 59° NE d Ankazobe; verbatimElevation: 1620; decimalLatitude: -18.17139; decimalLongitude: 47.28182; georeferenceRemarks: coordinates obtained from GPS; **Event:** samplingProtocol: MW 50 sample transect, 5m; eventDate: 04/17/2001; habitat: montane rainforest; fieldNumber: BLF03720; eventRemarks: sifted litter (leaf mold, rotten wood); **Record Level:** institutionCode: CASC
- **Type status:** Other material. **Occurrence:** catalogNumber: casent0458590; recordedBy: Fisher, Griswold et al.; sex: 1dQ; preparations: pin; associatedMedia: http://www.antweb.org/specimen/casent0458590; **Taxon:** scientificName: Stigmatomma irayhady; genus: Stigmatomma; **Location:** country: Madagascar; stateProvince: Antananarivo; locality: Réserve Spéciale d'Ambohitantely, Forêt d Ambohitantely, Jardin Botanique, 24.1km 59° NE d Ankazobe; verbatimElevation: 1620; decimalLatitude: -18.17139; decimalLongitude: 47.28182; georeferenceRemarks: coordinates obtained from GPS; **Event:** samplingProtocol: MW 50 sample transect, 5m; eventDate: 04/17/2001; habitat: montane rainforest; fieldNumber: BLF03720; eventRemarks: sifted litter (leaf mold, rotten wood); **Record Level:** institutionCode: CASC
- **Type status:** Other material. **Occurrence:** catalogNumber: casent0458591; recordedBy: Fisher, Griswold et al.; sex: 1w; preparations: pin; associatedMedia: http://www.antweb.org/specimen/casent0458591; **Taxon:** scientificName: Stigmatomma irayhady; genus: Stigmatomma; **Location:** country: Madagascar; stateProvince: Antananarivo; locality: Réserve Spéciale d'Ambohitantely, Forêt d Ambohitantely, Jardin Botanique, 24.1km 59° NE d Ankazobe; verbatimElevation: 1620; decimalLatitude: -18.17139; decimalLongitude: 47.28182; georeferenceRemarks: coordinates obtained from GPS; **Event:** samplingProtocol: MW 50 sample transect, 5m; eventDate: 04/17/2001; habitat: montane rainforest; fieldNumber: BLF03720; eventRemarks: sifted litter (leaf mold, rotten wood); **Record Level:** institutionCode: CASC

#### Description

Worker (Fig. 35; holotype values within parentheses): **HL**: 1.07-1.14 (1.14); **HW**: 0.93-0.97 (0.97); **HW2**: 0.82-0.84 (0.84); **SL**: 0.58-0.61 (0.61); **ML**: 0.65-0.72 (0.72); **WL**: 1.37-1.55 (1.55); **PPW**: 0.60-0.69 (0.69); **PtL**: 0.63-0.70 (0.70); **PtW**: 0.68-0.76 (0.76); **CI**: 85-88 (85); **SI**: 51-54 (53); **MI**: 60-63 (63); **PtI**: 91-96 (92). 

*Head*:

Mandibular baso-masticatory margin skirted dorsally by row of filiform setae; ventrally, by truncated filiform setae (Fig. 36a). Mandibular dentition arrangement, from base to apex: single larger tooth; five pairs of teeth (each teeth pair fused basally); somewhat bifid preapical tooth; apical tooth (Fig. 36a). Most-basal tooth of dorsal teeth pairs much smaller; absent in some specimens. Anterior clypeal margin with eight to nine tubercle-like cuticular processes, arranged in a single row, armed anteriorly with asymmetrical mucronate dentiform seta (Fig. 36a). Most-lateral clypeal cuticular process with row of smaller, conic setae anterolaterally (Fig. 36a). Row of clypeal conic setae continues laterally along clypeal anterior margin, arising from flat cuticle. Median clypeal cuticular processes with almost the same length of associated dentiform setae. Long filiform setae pair on anterior clypeal margin, bordering the most central cuticular processes. Median clypeal area extending posteriorly between antennal sockets, frontoclypeal sulcus round (Fig. 36b). Supraclypeal area as shallow oval concavity (Fig. 36b). Twelve antennomeres. Genal teeth present (Fig. 36b). Widest diameter of compound eyes: three ommatidia (Fig. 36c). Palpal formula: 4:3 (four maxillary, three labial; Fig. 36d​).

*Mesosoma*:

In dorsal view, mesonotum lateral margins continuous with posterior remainder of mesosoma (Fig. 37a). Metanotal suture absent or weakly impressed. Sulcus dividing mesepisternum into anepisternum and katepisternum (Fig. 37b). Metathoracic spiracle slit-like with somewhat swollen dorsoposterior margin; preceded anteroventrally by a cuticular swell; surrounded ventroposteriorly by shallow concentric sulcus (Fig. 37b). Propodeal spiracle round, with somewhat swollen margins (Fig. 37b). Propodeal declivitous face slightly concave (Fig. 37a).

*Legs*:

Basoventral three-fourths of calcar of strigil lamellar (Fig. 38a, b). Calcar of strigil anterior face with strap-like microtrichia; posterior face with lanceolate microtrichia (Fig. 38a, b). Multiple paddle-like setae on antero-ventral face of protibial apex, next to calcar of strigil (Fig. 38a). Multiple paddle-like setae on anterior face of probasitarsus; row of stout setae along posterior face, parallel to comb of strigil. Two mesotibial spurs; simple anterior spur with lanceolate microtrichia; posterior spur somewhat falcate (with rounded baso-ventral projection), bearing lanceolate microtrichia (Fig. 38c). Ventral margin of posterior mesotibial spur with few digitiform cuticular projections, restricted to the most basal region or distributed along ventral margin (Fig. 38c). Slit-like longitudinal sulcus on anterodorsal face of mesobasitarsus (Fig. 38d). Stout setae along inner face of mesobasitarsus. Two metatibial spurs; simple anterior spur with lanceolate microtrichia; posterior spur pectinate (Fig. 38e). Anterior face of posterior metatibial spur with sparse lanceolate microtrichia; posterior face glabrous (Fig. 38e, f). Absence of longitudinal sulcus on antero-dorsal face of metabasitarsus. Sparse, blunt, stout setae on baso-inner face of metabasitarsus; stout setae along remainder inner face. Arolium on pro-, meso-, and metapretarsus.

*Metasoma*:

Petiole sessile (Fig. 39a). Ventroanterior margin of petiolar tergite anterior dorso-latero-ventral carina (Ward 1990) much shorter than anterior margin of subpetiolar process, in lateral view (Fig. 39a). Subpetiolar process with midpoint of ventral margin angled obtusely (fin-like; Fig. 39a). Presence of fenestra on lateral face of subpetiolar process (Fig. 39a). Petiolar proprioceptor zone as large round concavity with numerous sensilla (Fig. 39b). Prora present (Fig. 39a). Scrobiculate sulcus between pretergite and postergite of abdominal segment III and presclerites and postsclerites of abdominal segment IV. Absence of stout setae on hypopygium (Fig. 39c).

**Figure 40.**
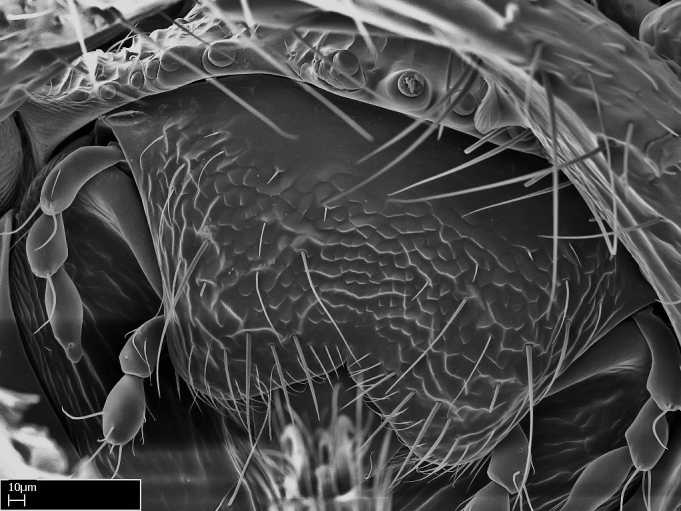
Mouthparts of *Stigmatomma irayhady* ​**sp. n.** worker (CASENT0458591), ventral view. Image by F. A. Esteves; available at AntWeb.org.

*Sculpture*:

Mandibular dorsal face areolate-rugose basally, grading into costate apically except for smooth apical portion (Fig. 36a). Clypeal median area costulate, grading into costate to rugose laterally (Fig. 36a). Supraclypeal area costulate (Fig. 36b). Anterior three-fourths of the head, in dorsal view, areolate-rugose, grading into foveolate/foveate posteriorly (Fig. 36b). Area posterior to tentorial pit tuberculate concentrically (Fig. 36b). Labrum imbricate (Fig. 40). Pronotum and dorsal face of remainder mesosoma foveolate (Fig. 37). Anepisternun mostly smooth; katepisternum foveolate-slightly rugulose dorsally, foveate-rugose ventrally (Fig. 37b). Metapleuron costulate-rugulose dorsally, costulate ventrally (Fig. 37b). Lateral face of propodeum scarsely foveolate, grading into rugose ventrally (Fig. 37b). Propodeal declivitous face foveolate. Petiolar tergite mostly foveolate/foveate (Fig. 39a). Petiolar laterotergite weakly imbricate anteriorly. Petiolar poststernite mostly alveolate (Fig. 39a). Abdominal segments III and IV foveolate; segments V, VI, and VII imbricate (Fig. 39c).

*Pilosity and color*:

Erect to subdecumbent pilosity on head. Erect to suberect pilosity on dorsal face of mesosoma, petiolar tergite, and abdominal segments III and IV. Erect pilosity on anterior half of petiolar poststernite. Longer pilosity on abdominal segments V, VI, and VII. Body color dark-brown to blackish; apex of the gaster orange; yellow-brown appendages.

##### Comments on character variation

No geographic pattern is seen in the variation on *Stigmatomma irayhady*, and characters such as body size, the presence of most basal masticatory tooth, number of dentiform setae on the anterior margin of the clypeus, presence and degree of development of metanotal suture, and presence and amount of digitiform cuticular projections on the ventral margin of the posterior mesotibial spur fluctuate even among specimens collected at the same locality. 

##### Other castes

Gyne (Fig. 41); alate when virgin: similar to the worker caste but for the greater body length, larger compound eyes, presence of ocelli, and differences on the mesosoma due to the presence of wings. Parapsidal lines on the mesoscutum; scuto-scutellar suture narrow, without apparent sculpture on its mid-section, but scrobiculate on its apexes (Fig. 42a). Mesepisternum divided into anepisternum and katepisternum; mesepimeral lobe distinct, but not well developed; metapleuron divided into upper and lower sections; upper metapleuron separated from propodeum by a carina followed dorsally by a scrobiculate sulcus; lower metapleuron separated from propodeum by strongly scrobiculate sulcus (Fig. 42b).

Males: Unknown.

#### Diagnosis

Worker

With characters of the *tsyhady* species-group and the *tsyhady* species-complex as described above, and the following characters:

1. Integument dark-brown; large-sized ant (HL: 1.07-1.14, WL: 1.37-1.55; Fig. 35).
2. Pairs of teeth along mandible’s baso-masticatory margin have the same length along mandible’s basoapical axis, except for smaller basal teeth of dorsal row (Fig. 36a).
3. Truncated filiform setae ventrally skirting baso-masticatory margin of mandible (Fig. 36a).
4. Dorsal face of the head areolate-rugose, grading to foveolate/foveate posteriorly (Figs 35a, 36b).
5. Palpal formula 4:3 (Fig. 36d).
6. Pronotum and the dorsal face of remainder of mesosoma foveolate; propodeal lateral face scarcely foveolate; propodeal declivitous face foveolate (Fig. 37).
7. Mesepisternum divided into anepisternum and katepisternum (Fig. 37b).
8. Basoventral three-fourths of calcar of strigil lamellar (Fig. 38a, b).
9. Anterior face of calcar of strigil with strap-like microtrichia (Fig. 38a).
10. Two well-developed mesotibial spurs (Fig. 38c).
11. Slit-like longitudinal sulcus present on the anterodorsal face of mesobasitarsus (Fig. 38d).
12. Two well-developed metatibial spurs (Fig. 38e).
13. Anterior face of posterior metatibial spur with sparse, small, lanceolate microtrichia; posterior face glabrous (Fig. 38e, f).
14. Absence of a longitudinal sulcus on metabasitarsus.
15. Sparse, blunt, stout setae present on the baso-inner face of metabasitarsus; stout setae present along the remainder inner face.
16. Subpetiolar process fin-like: half of its ventral margin obtusely angled (Fig. 39a).

Size, color, presence of genal teeth, palpal formula, presence of fenestra on the subpetiolar process, two mesotibial spurs, shape of microtrichia on posterior face of posterior metatibial spur, and absence of stout setae on the apex of hypopygium make it difficult to separate *Stigmatomma irayhady* from *S. roahady* and *S. tsyhady*.

However, *S. irayhady* possesses a sulcus on the anterodorsal face of the mesobasitarsus, and *S. tsyhady* does not; and it lacks a sulcus on the anterodorsal face of the metabasitarsus, which *S. roahady* has. Also, the ventral margin of the subpetiolar process decreases continuously posterad in *S. roahady* and *S. tsyhady*, while it has an obtuse angle at its midpoint in *S. irayhady*.

*S. irayhady* is sympatric with *S. roahady* in three localities: nearby Andranomay, close to Anjozorobe, and at the Binara Forest. It co-occurs with *S. tsyhady* at the Binara Forest.

#### Etymology

The name is a compound of the Malagasy cardinal number *iray*, meaning one, and the Malagasy noun *hady*, meaning sulcus, ditch, or trench. It refers to the presence of a longitudinal sulcus on the anterior face of the mesobasitarsus, and the absence of a longitudinal sulcus on the anterior face of the metabasitarsus of that species. This is not unique among *Stigmatomma* species in the Malagasy bioregion, but distinguishes *S. irayhady* from the other two species most similar to it, *S. tsyhady* and *S. roahady*.

#### Distribution

**Figure 43.**
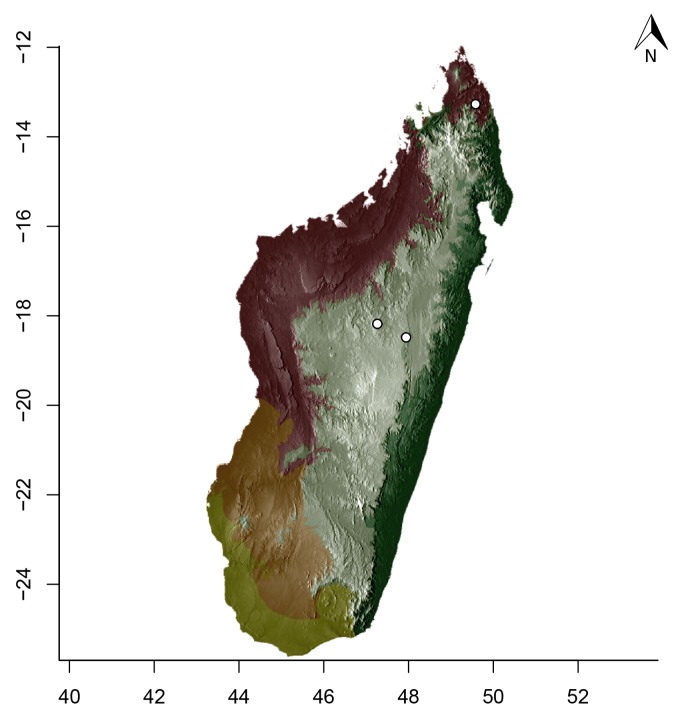
Distribution map of *Stigmatomma irayhady* ​**sp. n.** in the Malagasy bioregion. Collection localities are mapped over the outlines of five simplified ecoregion zones of Madagascar: humid forests (dark green), subhumid forests (light green), dry deciduous forests (brown), succulent woodlands (orange), and spiny thickets (yellow).

*Stigmatomma irayhady* was collected in montane rainforest habitats, above 1000 m, at the central to northern portions of the subhumid forests ecoregion of Madagascar (following the classification of Burgess et al. 2004; Fig. 43). Specimens were recorded from sifted leaf mold, rotten wood, and in soil.

### Stigmatomma janovitsika

Esteves & Fisher sp. n.

urn:lsid:zoobank.org:act:18B3E45E-C9B0-424A-AB57-8B6AC85FF525

#### Materials

- **Type status:** Holotype. **Occurrence:** catalogNumber: casent0161533; recordedBy: B.L.Fisher et al.; sex: 1w; preparations: pin; associatedMedia: http://www.antweb.org/specimen/casent0161533; **Taxon:** scientificName: Stigmatomma janovitsika; genus: Stigmatomma; **Location:** locationID: Mahé Blanc 660; country: Seychelles; locality: Mahé Island, Morne Blanc; verbatimElevation: 660; decimalLatitude: -4.6574; decimalLongitude: 55.43325; georeferenceRemarks: coordinates obtained from GPS; **Event:** samplingProtocol: 9 MaxiWinks, mixed samples; eventDate: 02/10/2010; habitat: mixed forest near glacis; fieldNumber: BLF24151; eventRemarks: sifted litter (leaf mold, rotten wood); **Record Level:** institutionCode: CASC
- **Type status:** Paratype. **Occurrence:** catalogNumber: casent0156023; recordedBy: B.L.Fisher et al.; sex: 1w; preparations: pin; associatedMedia: http://www.antweb.org/specimen/casent0156023; **Taxon:** scientificName: Stigmatomma janovitsika; genus: Stigmatomma; **Location:** locationID: Mahé Blanc 660; country: Seychelles; locality: Mahé Island, Morne Blanc; verbatimElevation: 660; decimalLatitude: -4.6574; decimalLongitude: 55.43325; georeferenceRemarks: coordinates obtained from GPS; **Event:** samplingProtocol: 9 MaxiWinks, mixed samples; eventDate: 02/10/2010; habitat: mixed forest near glacis; fieldNumber: BLF24151; eventRemarks: sifted litter (leaf mold, rotten wood); **Record Level:** institutionCode: CASC
- **Type status:** Paratype. **Occurrence:** catalogNumber: casent0161532; recordedBy: B.L.Fisher et al.; sex: 1dQ; preparations: pin; associatedMedia: http://www.antweb.org/specimen/casent0161532; **Taxon:** scientificName: Stigmatomma janovitsika; genus: Stigmatomma; **Location:** locationID: Mahé Blanc 660; country: Seychelles; locality: Mahé Island, Morne Blanc; verbatimElevation: 660; decimalLatitude: -4.6574; decimalLongitude: 55.43325; georeferenceRemarks: coordinates obtained from GPS; **Event:** samplingProtocol: 9 MaxiWinks, mixed samples; eventDate: 02/10/2010; habitat: mixed forest near glacis; fieldNumber: BLF24151; eventRemarks: sifted litter (leaf mold, rotten wood); **Record Level:** institutionCode: CASC
- **Type status:** Paratype. **Occurrence:** catalogNumber: casent0156022; recordedBy: B.L.Fisher et al.; sex: 1w; preparations: pin; associatedMedia: http://www.antweb.org/specimen/casent0156022; **Taxon:** scientificName: Stigmatomma janovitsika; genus: Stigmatomma; **Location:** locationID: Mahé Blanc 660; country: Seychelles; locality: Mahé Island, Morne Blanc; verbatimElevation: 660; decimalLatitude: -4.6574; decimalLongitude: 55.43325; georeferenceRemarks: coordinates obtained from GPS; **Event:** samplingProtocol: 9 MaxiWinks, mixed samples; eventDate: 02/10/2010; habitat: mixed forest near glacis; fieldNumber: BLF24151; eventRemarks: sifted litter (leaf mold, rotten wood); **Record Level:** institutionCode: MHNG
- **Type status:** Other material. **Occurrence:** catalogNumber: casent0145426; recordedBy: B.L.Fisher et al.; sex: 1w; preparations: SEM mount; associatedMedia: http://www.antweb.org/specimen/casent0145426; **Taxon:** scientificName: Stigmatomma janovitsika; genus: Stigmatomma; **Location:** locationID: Mahé Blanc 660; country: Seychelles; locality: Mahé Island, Morne Blanc; verbatimElevation: 660; decimalLatitude: -4.6574; decimalLongitude: 55.43325; georeferenceRemarks: coordinates obtained from GPS; **Event:** samplingProtocol: 9 MaxiWinks, mixed samples; eventDate: 02/10/2010; habitat: mixed forest near glacis; fieldNumber: BLF24151; eventRemarks: sifted litter (leaf mold, rotten wood); **Record Level:** institutionCode: CASC
- **Type status:** Other material. **Occurrence:** catalogNumber: casent0159676; recordedBy: B.L.Fisher et al.; sex: 1w; preparations: pin; associatedMedia: http://www.antweb.org/specimen/casent0159676; **Taxon:** scientificName: Stigmatomma janovitsika; genus: Stigmatomma; **Location:** locationID: Conception 65; country: Seychelles; locality: Conception Island; verbatimElevation: 65; decimalLatitude: -4.66311; decimalLongitude: 55.36821; georeferenceRemarks: coordinates obtained from GPS; **Event:** samplingProtocol: General collecting; eventDate: 02/12/2010; habitat: mixed forest; fieldNumber: BLF24276; eventRemarks: under rootmat, litter on rock; **Record Level:** institutionCode: CASC
- **Type status:** Other material. **Occurrence:** catalogNumber: casent0159677; recordedBy: B.L.Fisher et al.; sex: 1w; preparations: pin; associatedMedia: http://www.antweb.org/specimen/casent0159677; **Taxon:** scientificName: Stigmatomma janovitsika; genus: Stigmatomma; **Location:** locationID: Silhouette 520; country: Seychelles; locality: Silhouette Island, above Jardin Marron on crest to Mont Plaisir and Pot à Eau; verbatimElevation: 520; decimalLatitude: -4.4867; decimalLongitude: 55.2341; georeferenceRemarks: coordinates obtained from GPS; **Event:** samplingProtocol: General collecting; eventDate: 01/20/2010; habitat: forest; fieldNumber: BLF23168; eventRemarks: ex rotten log; **Record Level:** institutionCode: CASC
- **Type status:** Other material. **Occurrence:** catalogNumber: casent0159679; recordedBy: B.L.Fisher et al.; sex: 1w; preparations: pin; associatedMedia: http://www.antweb.org/specimen/casent0159679; **Taxon:** scientificName: Stigmatomma janovitsika; genus: Stigmatomma; **Location:** locationID: Conception 65; country: Seychelles; locality: Conception Island; verbatimElevation: 65; decimalLatitude: -4.66311; decimalLongitude: 55.36821; georeferenceRemarks: coordinates obtained from GPS; **Event:** samplingProtocol: General collecting; eventDate: 02/12/2010; habitat: mixed forest; fieldNumber: BLF24244; eventRemarks: under rootmat, litter on rock; **Record Level:** institutionCode: CASC
- **Type status:** Other material. **Occurrence:** catalogNumber: casent0159680; recordedBy: B.L.Fisher et al.; sex: 1w; preparations: pin; associatedMedia: http://www.antweb.org/specimen/casent0159680; **Taxon:** scientificName: Stigmatomma janovitsika; genus: Stigmatomma; **Location:** locationID: Gratte Fesse 410; country: Seychelles; locality: Silhouette Island, Gratte Fesse; verbatimElevation: 410; decimalLatitude: -4.49169; decimalLongitude: 55.23886; georeferenceRemarks: coordinates obtained from GPS; **Event:** samplingProtocol: General collecting; eventDate: 01/25/2010; habitat: forest; fieldNumber: BLF23396; eventRemarks: ex rotten log; **Record Level:** institutionCode: CASC
- **Type status:** Other material. **Occurrence:** catalogNumber: casent0160355; recordedBy: B.L.Fisher et al.; sex: 1w; preparations: pin; associatedMedia: http://www.antweb.org/specimen/casent0160355; **Taxon:** scientificName: Stigmatomma janovitsika; genus: Stigmatomma; **Location:** locationID: Conception 65; country: Seychelles; locality: Conception Island; verbatimElevation: 65; decimalLatitude: -4.66311; decimalLongitude: 55.36821; georeferenceRemarks: coordinates obtained from GPS; **Event:** samplingProtocol: 9 MaxiWinks, mixed samples; eventDate: 02/12/2010; habitat: mixed forest; fieldNumber: BLF24286; eventRemarks: sifted litter (leaf mold, rotten wood); **Record Level:** institutionCode: CASC
- **Type status:** Other material. **Occurrence:** catalogNumber: casent0160379; recordedBy: B.L.Fisher et al.; sex: 1w; preparations: pin; associatedMedia: http://www.antweb.org/specimen/casent0160379; **Taxon:** scientificName: Stigmatomma janovitsika; genus: Stigmatomma; **Location:** locationID: Conception 65; country: Seychelles; locality: Conception Island; verbatimElevation: 65; decimalLatitude: -4.66311; decimalLongitude: 55.36821; georeferenceRemarks: coordinates obtained from GPS; **Event:** samplingProtocol: 9 MaxiWinks, mixed samples; eventDate: 02/12/2010; habitat: mixed forest; fieldNumber: BLF24286; eventRemarks: sifted litter (leaf mold, rotten wood); **Record Level:** institutionCode: CASC
- **Type status:** Other material. **Occurrence:** catalogNumber: casent0160792; recordedBy: B.L.Fisher et al.; sex: 1m; preparations: pin, slide; associatedMedia: http://www.antweb.org/specimen/casent0160792; **Taxon:** scientificName: Stigmatomma janovitsika; genus: Stigmatomma; **Location:** locationID: Silhouette 520; country: Seychelles; locality: Silhouette Island, above Jardin Marron on crest to Mont Plaisir and Pot à Eau; verbatimElevation: 520; decimalLatitude: -4.4867; decimalLongitude: 55.2341; georeferenceRemarks: coordinates obtained from GPS; **Event:** samplingProtocol: Malaise trap; eventDate: 01/20/2010; habitat: forest; fieldNumber: BLF23134; **Record Level:** institutionCode: CASC
- **Type status:** Other material. **Occurrence:** catalogNumber: casent0318444; recordedBy: B.L.Fisher et al.; sex: 1w; preparations: pin; associatedMedia: http://www.antweb.org/specimen/casent0318444; **Taxon:** scientificName: Stigmatomma janovitsika; genus: Stigmatomma; **Location:** locationID: Gratte Fesse 410; country: Seychelles; locality: Silhouette Island, Gratte Fesse; verbatimElevation: 410; decimalLatitude: -4.49169; decimalLongitude: 55.23886; georeferenceRemarks: coordinates obtained from GPS; **Event:** samplingProtocol: General collecting; eventDate: 01/25/2010; habitat: forest; fieldNumber: BLF23396; eventRemarks: ex rotten log; **Record Level:** institutionCode: CASC
- **Type status:** Other material. **Occurrence:** catalogNumber: casent0318418; recordedBy: B.L.Fisher et al.; sex: 1w; preparations: SEM mount; associatedMedia: http://www.antweb.org/specimen/casent0318418; **Taxon:** scientificName: Stigmatomma janovitsika; genus: Stigmatomma; **Location:** locationID: Conception 65; country: Seychelles; locality: Conception Island; verbatimElevation: 65; decimalLatitude: -4.66311; decimalLongitude: 55.36821; georeferenceRemarks: coordinates obtained from GPS; **Event:** samplingProtocol: General collecting; eventDate: 02/12/2010; habitat: mixed forest; fieldNumber: BLF24276; eventRemarks: under rootmat, litter on rock; **Record Level:** institutionCode: CASC
- **Type status:** Other material. **Occurrence:** catalogNumber: casent0318446; recordedBy: B.L.Fisher et al.; sex: 1m; preparations: pin, slide; associatedMedia: http://www.antweb.org/specimen/casent0318446; **Taxon:** scientificName: Stigmatomma janovitsika; genus: Stigmatomma; **Location:** locationID: Silhouette 520; country: Seychelles; locality: Silhouette Island, above Jardin Marron on crest to Mont Plaisir and Pot à Eau; verbatimElevation: 520; decimalLatitude: -4.4867; decimalLongitude: 55.2341; georeferenceRemarks: coordinates obtained from GPS; **Event:** samplingProtocol: Malaise trap; eventDate: 01/20/2010; habitat: forest; fieldNumber: BLF23134; **Record Level:** institutionCode: CASC
- **Type status:** Other material. **Occurrence:** catalogNumber: casent0318447; recordedBy: B.L.Fisher et al.; sex: 1m; preparations: pin, slide; associatedMedia: http://www.antweb.org/specimen/casent0318447; **Taxon:** scientificName: Stigmatomma janovitsika; genus: Stigmatomma; **Location:** locationID: Silhouette 520; country: Seychelles; locality: Silhouette Island, above Jardin Marron on crest to Mont Plaisir and Pot à Eau; verbatimElevation: 520; decimalLatitude: -4.4867; decimalLongitude: 55.2341; georeferenceRemarks: coordinates obtained from GPS; **Event:** samplingProtocol: Malaise trap; eventDate: 01/20/2010; habitat: forest; fieldNumber: BLF23134; **Record Level:** institutionCode: CASC
- **Type status:** Other material. **Occurrence:** catalogNumber: casent0318448; recordedBy: B.L.Fisher et al.; sex: 1m; preparations: pin, slide; associatedMedia: http://www.antweb.org/specimen/casent0318448; **Taxon:** scientificName: Stigmatomma janovitsika; genus: Stigmatomma; **Location:** locationID: Silhouette 520; country: Seychelles; locality: Silhouette Island, above Jardin Marron on crest to Mont Plaisir and Pot à Eau; verbatimElevation: 520; decimalLatitude: -4.4867; decimalLongitude: 55.2341; georeferenceRemarks: coordinates obtained from GPS; **Event:** samplingProtocol: Malaise trap; eventDate: 01/20/2010; habitat: forest; fieldNumber: BLF23134; **Record Level:** institutionCode: CASC
- **Type status:** Other material. **Occurrence:** catalogNumber: casent0318448; recordedBy: B.L.Fisher et al.; sex: 1m; preparations: slide; associatedMedia: http://www.antweb.org/specimen/casent0318448; **Taxon:** scientificName: Stigmatomma janovitsika; genus: Stigmatomma; **Location:** locationID: Silhouette 520; country: Seychelles; locality: Silhouette Island, above Jardin Marron on crest to Mont Plaisir and Pot à Eau; verbatimElevation: 520; decimalLatitude: -4.4867; decimalLongitude: 55.2341; georeferenceRemarks: coordinates obtained from GPS; **Event:** samplingProtocol: Malaise trap; eventDate: 01/20/2010; habitat: forest; fieldNumber: BLF23134; **Record Level:** institutionCode: CASC

#### Description

Worker (Fig. 44; holotype values within parentheses): **HL**: 0.74-0.79 (0.79); **HW**: 0.63-0.67 (0.67); **HW2**: 0.58-0.62 (0.61); **SL**: 0.44-0.46 (0.46); **ML**: 0.54-0.57 (0.56); **WL**: 0.87-0.93 (0.93); **PPW**: 0.34-0.37 (0.37); **PtL**: 0.36-0.38 (0.38); **PtW**: 0.39-0.42 (0.42); **CI**: 84-88 (84); **SI**: 58-61 (58); **MI**: 71-75 (71); **PtI**: 90-93 (91). 

*Head*:

Mandibular baso-masticatory margin skirted dorsally by row of filiform setae; medially, by spatular setae; ventrally, by longer acuminate flattened-apex setae (Fig. 45a). Dentition arrangement, from base to apex: single larger tooth; single smaller tooth; four pairs of teeth; bicuspid pre-apical tooth; apical tooth (Fig. 45a). Tooth couples with same dimensions; teeth basally fused. Pairs of teeth with similar dimensions along mandible's basoapical axis. Anterior clypeal margin with six tubercle-like cuticular processes arranged in a single row; armed anteriorly with asymmetrical mucronate dentiform setae (Fig. 45a). Most lateral clypeal cuticular process armed anterolaterally with row of numerous smaller, blunt, dentiform setae, continuing laterad on clypeal anterior margin, arising from flat cuticle (Fig. 45a). Clypeal cuticular processes approximately the same length as associated dentiform setae. Pair of long, filiform setae on clypeal median area, posterior to central-most pair of cuticular processes on anterior clypeal margin. Clypeal corners with brush of filiform setae; if absent, numerous punctations instead (Fig. 45a). Median clypeal area extending posteriorly between antennal sockets as a narrow longitudinal strip; frontoclypeal sulcus acute (Fig. 45b). Supraclypeal area as small oblong depression (Fig. 45b​). Twelve antennomeres (Fig. 45b​). Small genal teeth present. Compound eyes absent. Palpal formula: 4:2 (four maxillary, two labial; ​Fig. 45c).

*Mesosoma*:

In dorsal view, mesonotum narrower than remaining mesosoma (Fig. 46a). Metanotal suture absent (Fig. 46a​). Mesepisternum not divided into anepisternum and kaptepisternum (Fig. 46b). Sulcus separating mesepisternum from posterior remainder of mesosoma, running from metathoracic spiracle to endoapodemal pit of mesopleural arm (Fig. 46b​). Metathoracic spiracle round, pinched inside its opening, and surrounded by cuticular swell (Fig. 46b​). Propodeal spiracle round, surrounded by cuticular swell (Fig. 46b​). Propodeal declivitous face with raised lateral margins (Fig. 46a​).

*Legs*:

Basoventral lamella of calcar of strigil reduced to a basal bud. Anterior face of calcar of strigil with tubiform microtrichia (Fig. 47a); posterior face with lanceolate microtrichia. Multiple paddle-like setae on antero-ventral face of protibial apex, next to calcar of strigil (Fig. 47a​). Multiple paddle-like setae on anterior face of probasitarsus (Fig. 47a​); row of stout setae along posterior face of probasitarsus, next to comb of strigil. Mesotibial spur absent. Apex of mesotibial inner face with long, stout, spiniform seta resembling a spur under the optical microscope, followed apically by a deep fovea concealing a small, stout, truncated seta (Fig. 47b, c, d). Slit-like longitudinal sulcus on anterodorsal face of mesobasitarsus, with apical end projected laterally (Fig. 47e). Two metatibial spurs; simple anterior spur with lanceolate microtrichia; posterior spur pectinate (Fig. 48a, b). Anterior face of posterior metatibial spur glabrous; posterior face with numerous antler-like microtrichia. Brush of truncated-apex long filiform setae on posterior face of metatibial apex, next to posterior metatibial spur (Fig. 48b). Absence of longitudinal sulcus on antero-dorsal face of metabasitarsus. Base of the inner face of metabasitarsus swollen anteriorly; swollen posterior face with longitudinal row of truncated, flattened-apex stout setae, followed by brush of filiform setae apically (Fig. 48a, b, c). Somewhat stout setae along inner face of remaining metabasitarsus. Arolium on pro-, meso-, and metapretarsus.

*Metasoma*:

Petiole sessile (Fig. 49a). Ventroanterior margin of petiolar tergite anterior dorso-latero-ventral carina (Ward 1990) much shorter than anterior margin of subpetiolar process, in lateral view (Fig. 49a​). Subpetiolar process fin-like: obtuse angle on mid-length of its ventral margin (Fig. 49a​). Absence of fenestra on lateral face of subpetiolar process (Fig. 49a​). Petiolar proprioceptor zone a large, round concavity with few sensilla (Fig. 49b). Prora present (Fig. 49a​). Scrobiculate sulcus between pretergite and postergite of abdominal segment III and presclerites and postsclerites of abdominal segment IV. Eight stout spiniform setae on apex of hypopygium (Fig. 49c).

**Figure 50.**
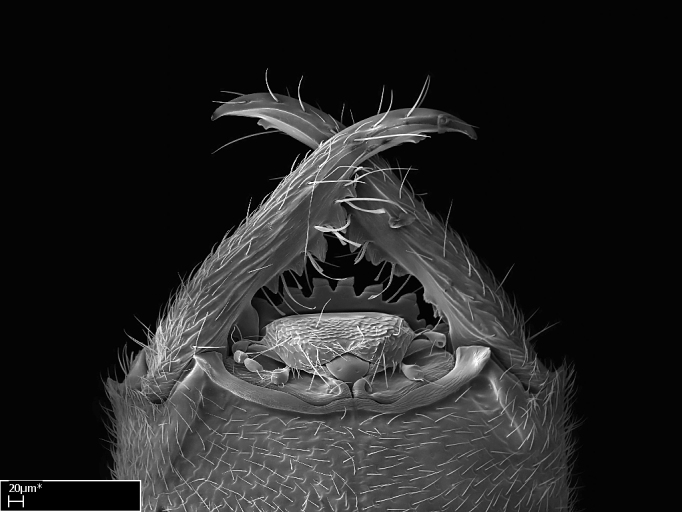
*Stigmatomma janovitsika* ​**sp. n.** worker (CASENT0145426): ventral view of the mandibles, mouthparts, and anterior part of the head. Image by F. A. Esteves; available at AntWeb.org.

*Sculpture*:

Mandibular dorsal face rugose-foveolate basally, grading into costate apically, except for smooth apical portion (Fig. 45a). Clypeal median area smooth, grading to costulate laterally (Fig. 45a). Supraclypeal area smooth (Fig. 45b). Head in dorsal view, areolate; area posterior to tentorial pit plicate (Fig. 45b​). Labrum imbricate (Fig. 50). Mesosoma foveolate dorsally (Fig. 46a). Pronotum rugose-foveolate laterally; remainder of lateral face of mesosoma mostly costate (Fig. 46b). Propodeal declivitous face smooth (Fig. 46a). Petiolar tergite alveolate ventroanteriorly, grading to smooth anteriorly, imbricate laterally, and foveolate dorsally (Fig. 49a). Petiolar laterotergite smooth anteriorly, grading to alveolate posteriorly and imbricate lateroposteriorly (Fig. 49b). Petiolar poststernite imbricate anteriorly, grading to alveolate to smooth posteriorly (Fig. 49b​). Abdominal segment III foveolate; segment IV punctate; segments V, VI, and VII imbricate (Fig. 49c).

*Pilosity and color*:

Erect to subdecumbent pilosity on head, dorsal face of mesosoma, petiolar tergite, and abdominal segments III and IV. Petiolar poststernite mostly glabrous, with row of setae along lateral margins. Longer pilosity on abdominal segments V, VI, and VII. Body color orange-brown; light-orange appendages.

##### Comments on character variation

Under the stereomicroscope, there is no observable character variation on the specimens examined.

##### Other castes

Gyne (Fig. 51); alate when virgin: Very similar to the worker caste but for the greater body length, presence of compound eyes and ocelli, and differences on the mesosoma due to the presence of wings. Parapsidal lines on the mesoscutum; scuto-scutellar suture narrow, without apparent sculpture (Fig. 52a​). Mesepisternum not divided into anepisternum and katepisternum by a sulcus, but the upper mesepisternum is clearly smoother than its lower section; mesepimeral lobe not distinct; metapleuron not divided into upper and lower sections but for a short and narrow longitudinal sulcus located around the mid-length of the suture separating mesopleuron from metapleuron; metapleuron not clearly distinct from the propodeum (Fig. 52b​).

Male (Fig. 53); alate: Mandibles falcate, with sharp, single apical tooth (Fig. 53a). Anterior margin of the clypeus with dentiform setae (Fig. 53a). Compound eyes with long setae among ommatidia (Fig. 54a). Palpal formula 4:2 (Fig. 55a). Notauli distinct; parapsidal lines present; scuto-scutellar suture narrow, not sculptured (Fig. 54b). Mesepisternum not divided into anepisternum and katepisternum; posterior oblique sulcus short, not well developed; mesepimeral lobe not distinct (Fig. 54c). Metapleuron divided into upper and lower sections by a sulcus; costate sulcus separating upper metapleuron from propodeum; lower metapleuron not completey distinct from the propodeum (Fig. 54c​). Forewing (Figs 53d, 56a, b, 57a, b): pterostigma well developed; Rs.f2-3 may be indistinct; Rs.f5 present and reaching R.f3; 1r-rs absent; 2r-rs present; M.f2 present, but may be just slightly distinct; Rs+M complete or not well-developed; M.f3-4 present; 2rs-m absent; Cu.f2 present; 1m-cu present or just slightly distinct; A.f2 present; cu-a intercepting M+Cu anteriorly to the separation point between M.f1 and Cu.f1. Hindwing (Figs 56c, d, 57c, d): C slightly distinct; Sc+R, R, Rs.f1, and Rs.f2 absent; M+Cu just slightly distinct; 1rs-m, M.f1, M.f2, Cu, and cu-a absent; A present. Pygostyles present (Fig. 54d). Posterior margin of abdominal sternum IX convex (Fig. 55d). Visible division of the paramere into telomere and basimere. Digitus mushroom-shaped; presence of a short projection at the base of the digitus (Fig. 55c). Anterior half of the ventral margin of penisvalva clearly serrate; ventral portion of the penisvalva extremely reduced if compared with other *Stigmatomma* species in the Malagasy bioregion; dorsal portion somewhat sclerotized (Fig. 55b).

##### Specimens used in prior studies

This taxon was referenced as *Stigmatomma* SC01 (specimen CASENT0159676-D01) in Ward and Fisher (2016).

#### Diagnosis

Worker

With characters of the *tsyhady* species-group and the *sakalava* species-complex as described above, and the following characters (asterisks flag unique characters within the genus in the Malagasy bioregion):

1. Integument orange-brown (Fig. 44); medium-sized ant (HL: 0.74-0.79, WL: 0.87-0.93).
2. Pairs of teeth along baso-masticatory margin of mandible have the same length along the baso-apical axis (Fig. 45a, b).
3. Bicuspid pre-apical tooth (Fig. 45a, b).
4. Long acuminate flattened-apex setae ventrally skirting baso-masticatory margin of mandible (Fig. 45b).
* Most lateral area of clypeus bearing a brush of filiform setae (when setae are not present, the region presents numerous punctuations; Figs 44a, 45a).
6. Dorsal face of the head areolate (Figs 44a, 45b).
7. Genal teeth present (Fig. 44a).
8. Palpal formula 4:2 (Fig. 45c).
9. Dorsal face of mesosoma foveolate; lateral face of propotum rugose-foveolate; remainder lateral face of mesosoma mostly costate; declivitous face of propodeum smooth (Fig. 46).
* Mesepisternum not divided into anepisternum and katepisternum (Fig. 46b).
* Basoventral lamella of calcar of strigil reduced to a basal bud.
12. Anterior face of calcar of strigil with tubiform microtrichia (Fig. 47a).
13. Mesotibial spur absent (Fig. 47b, c).
* Apex of mesotibial inner face bearing a long, stout, spiniform seta, resembling a spur under optical microscope (Fig. 47b, c).
15. Mesotibial apical stout seta apically followed by a deep fovea concealing small, stout, truncated seta (Fig. 47c, d).
16. Slit-like sulcus present on the anterodorsal face of mesobasitarsus, with apical end projected laterally (Fig. 47e).
17. Anterior face of posterior metatibial spur glabrous (Fig. 48a).
18. Brush of truncated filiform setae present on the posterior face of the apex of metatibia (Fig. 48b).
* Base of the inner face of metabasitarsus swollen anteriorly. Posterior face of basal swollen area bearing a row of truncated, flattened-apex stout setae, followed apically by a brush of filiform setae (Fig. 48c).
20. Absence of a longitudinal sulcus on metabasitarsus.
21. Subpetiolar process fin-like: half of its ventral margin obtusely angled (Fig. 49a).
22. Eight stout spiniform setae present on the apex of hypopygium (Fig. 49c).

*Stigmatomma janovitsika* is somewhat similar to *S. bolabola* and *S. sakalava* in palpal formula, shape of microtrichia on the posterior face of posterior metatibial spur, and absence of fenestra on the subpetiolar process.

However, it may be distinguished from them by: brush of filiform setae present on the corners of the clypeus (if the setae are removed, the region will be densely punctuate); presence of genal teeth; the mesepisternum is not divided into anepisternum and katepisternum; head sculpture; proportion of lamella on the baso-ventral margin of the calcar of strigil; long, stout, spiniform seta on the mesotibial inner face apex (resembling a spur under the stereomicroscope), followed apically by a cuticular deep fovea concealing a small, stout, truncated seta; and distribution, since it does not occur in sympatry with any of its congeners.

#### Etymology

The name janovitsika is a portmanteau of Janovitz and vitsika (Malagasy name for ants), meaning the ant of Janovitz. Dr. Tyler W. Janovitz is a medical scientist interested in myrmecology, and generously supported this study.

#### Distribution

**Figure 58.**
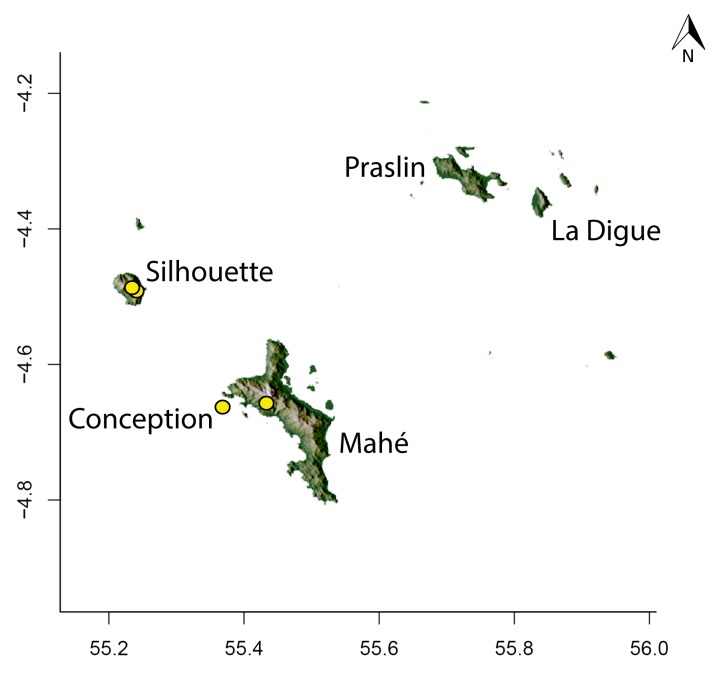
Distribution map of *Stigmatomma janovitsika* **sp. n.** in the Seychelles islands.

*Stigmatomma janovitsika* specimens were collected in forest, mixed forest, and mixed forest near glacis (rocky outcrop) habitats, from 60 to around 700 m above sea level, on three granitic islands of the Seychelles (Conception, Mahé, and Silhouette; Fig. 58). Specimens were recorded: (1) manually under rootmat and litter on rocks, and in rotten logs; (2) from sifted leaf mold and rotten wood; and (3) in a Malaise trap.

### Stigmatomma liebe

Esteves & Fisher sp. n.

urn:lsid:zoobank.org:act:31615B2C-561A-44F8-AFC7-ADAE5A369B67

#### Materials

- **Type status:** Holotype. **Occurrence:** catalogNumber: casent0318428; recordedBy: B.L.Fisher (Sylvain); sex: 1w; preparations: pin; associatedMedia: http://www.antweb.org/specimen/casent0318428; **Taxon:** scientificName: Stigmatomma liebe; genus: Stigmatomma; **Location:** country: Madagascar; stateProvince: Fianarantsoa; locality: 8.0 km NE Ivohibe; verbatimElevation: 1200; decimalLatitude: -22.42167; decimalLongitude: 46.89833; **Event:** samplingProtocol: MW 50 sample transect, 5m; eventDate: 11/03/1997; habitat: montane rainforest; fieldNumber: BLF01753; eventRemarks: sifted litter (leaf mold, rotten wood); **Record Level:** institutionCode: CASC
- **Type status:** Paratype. **Occurrence:** catalogNumber: casent0746700; recordedBy: B.L.Fisher (Sylvain); sex: 3w; preparations: pin; associatedMedia: http://www.antweb.org/specimen/casent0746700; **Taxon:** scientificName: Stigmatomma liebe; genus: Stigmatomma; **Location:** country: Madagascar; stateProvince: Fianarantsoa; locality: 8.0 km NE Ivohibe; verbatimElevation: 1200; decimalLatitude: -22.42167; decimalLongitude: 46.89833; **Event:** samplingProtocol: MW 50 sample transect, 5m; eventDate: 11/03/1997; habitat: montane rainforest; fieldNumber: BLF01753; eventRemarks: sifted litter (leaf mold, rotten wood); **Record Level:** institutionCode: CASC
- **Type status:** Paratype. **Occurrence:** catalogNumber: casent0746702; recordedBy: B.L.Fisher (Sylvain); sex: 1dQ; preparations: pin; associatedMedia: http://www.antweb.org/specimen/casent0746702; **Taxon:** scientificName: Stigmatomma liebe; genus: Stigmatomma; **Location:** country: Madagascar; stateProvince: Fianarantsoa; locality: 8.0 km NE Ivohibe; verbatimElevation: 1200; decimalLatitude: -22.42167; decimalLongitude: 46.89833; **Event:** samplingProtocol: MW 50 sample transect, 5m; eventDate: 11/03/1997; habitat: montane rainforest; fieldNumber: BLF01753; eventRemarks: sifted litter (leaf mold, rotten wood); **Record Level:** institutionCode: CASC
- **Type status:** Paratype. **Occurrence:** catalogNumber: casent0746699; recordedBy: B.L.Fisher (Sylvain); sex: 1w; preparations: pin; associatedMedia: http://www.antweb.org/specimen/casent0746699; **Taxon:** scientificName: Stigmatomma liebe; genus: Stigmatomma; **Location:** country: Madagascar; stateProvince: Fianarantsoa; locality: 8.0 km NE Ivohibe; verbatimElevation: 1200; decimalLatitude: -22.42167; decimalLongitude: 46.89833; **Event:** samplingProtocol: MW 50 sample transect, 5m; eventDate: 11/03/1997; habitat: montane rainforest; fieldNumber: BLF01753; eventRemarks: sifted litter (leaf mold, rotten wood); **Record Level:** institutionCode: BMNH
- **Type status:** Paratype. **Occurrence:** catalogNumber: casent0746701; recordedBy: B.L.Fisher (Sylvain); sex: 1w; preparations: pin; associatedMedia: http://www.antweb.org/specimen/casent0746701; **Taxon:** scientificName: Stigmatomma liebe; genus: Stigmatomma; **Location:** country: Madagascar; stateProvince: Fianarantsoa; locality: 8.0 km NE Ivohibe; verbatimElevation: 1200; decimalLatitude: -22.42167; decimalLongitude: 46.89833; **Event:** samplingProtocol: MW 50 sample transect, 5m; eventDate: 11/03/1997; habitat: montane rainforest; fieldNumber: BLF01753; eventRemarks: sifted litter (leaf mold, rotten wood); **Record Level:** institutionCode: MHNG
- **Type status:** Other material. **Occurrence:** catalogNumber: blf0561(l.o.)-03; recordedBy: B.L.Fisher; sex: 1w, wet; preparations: pin; associatedMedia: http://www.antweb.org/specimen/blf0561(l.o.)-03; **Taxon:** scientificName: Stigmatomma liebe; genus: Stigmatomma; **Location:** country: Madagascar; stateProvince: Toliara; locality: 13 km NW Enakara, Rés. Andohahela; verbatimElevation: 1250; decimalLatitude: -24.55; decimalLongitude: 46.8; **Event:** samplingProtocol: MW 50 sample transect, 5m; eventDate: 11/30/1992; habitat: montane rainforest; fieldNumber: BLF00561; eventRemarks: sifted litter (leaf mold, rotten wood); **Record Level:** institutionCode: CASC
- **Type status:** Other material. **Occurrence:** catalogNumber: casent0009101; recordedBy: B.L.Fisher; sex: 2w; preparations: pin; associatedMedia: http://www.antweb.org/specimen/casent0009101; **Taxon:** scientificName: Stigmatomma liebe; genus: Stigmatomma; **Location:** country: Madagascar; stateProvince: Toliara; locality: 13 km NW Enakara, Rés. Andohahela; verbatimElevation: 1250; decimalLatitude: -24.55; decimalLongitude: 46.8; **Event:** samplingProtocol: MW 50 sample transect, 5m; eventDate: 11/30/1992; habitat: montane rainforest; fieldNumber: BLF00561; eventRemarks: sifted litter (leaf mold, rotten wood); **Record Level:** institutionCode: CASC
- **Type status:** Other material. **Occurrence:** catalogNumber: casent0009102; recordedBy: B.L.Fisher; sex: 2w; preparations: pin, SEM mount; associatedMedia: http://www.antweb.org/specimen/casent0009102; **Taxon:** scientificName: Stigmatomma liebe; genus: Stigmatomma; **Location:** country: Madagascar; stateProvince: Toliara; locality: 13 km NW Enakara, Rés. Andohahela; verbatimElevation: 1250; decimalLatitude: -24.55; decimalLongitude: 46.8; **Event:** samplingProtocol: MW 50 sample transect, 5m; eventDate: 11/30/1992; habitat: montane rainforest; fieldNumber: BLF00561; eventRemarks: sifted litter (leaf mold, rotten wood); **Record Level:** institutionCode: CASC
- **Type status:** Other material. **Occurrence:** catalogNumber: casent0227587; recordedBy: B.L.Fisher; sex: 1w; preparations: SEM mount; associatedMedia: http://www.antweb.org/specimen/casent0227587; **Taxon:** scientificName: Stigmatomma liebe; genus: Stigmatomma; **Location:** country: Madagascar; stateProvince: Toliara; locality: 13 km NW Enakara, Rés. Andohahela; verbatimElevation: 1250; decimalLatitude: -24.55; decimalLongitude: 46.8; **Event:** samplingProtocol: MW 50 sample transect, 5m; eventDate: 11/30/1992; habitat: montane rainforest; fieldNumber: BLF00561; eventRemarks: sifted litter (leaf mold, rotten wood); **Record Level:** institutionCode: CASC
- **Type status:** Other material. **Occurrence:** catalogNumber: casent0318413; recordedBy: B.L.Fisher; sex: 1w; preparations: pin; associatedMedia: http://www.antweb.org/specimen/casent0318413; **Taxon:** scientificName: Stigmatomma liebe; genus: Stigmatomma; **Location:** country: Madagascar; stateProvince: Toliara; locality: 13 km NW Enakara, Rés. Andohahela; verbatimElevation: 1250; decimalLatitude: -24.55; decimalLongitude: 46.8; **Event:** samplingProtocol: MW 50 sample transect, 5m; eventDate: 11/30/1992; habitat: montane rainforest; fieldNumber: BLF00561; eventRemarks: sifted litter (leaf mold, rotten wood); **Record Level:** institutionCode: CASC
- **Type status:** Other material. **Occurrence:** catalogNumber: casent0318414; recordedBy: B.L.Fisher (Sylvain); sex: 1w; preparations: SEM mount; associatedMedia: http://www.antweb.org/specimen/casent0318414; **Taxon:** scientificName: Stigmatomma liebe; genus: Stigmatomma; **Location:** country: Madagascar; stateProvince: Fianarantsoa; locality: R.S. Ivohibe 8.0 km E Ivohibe; verbatimElevation: 1200; decimalLatitude: -22.48333; decimalLongitude: 46.96833; **Event:** samplingProtocol: MW 50 sample transect, 5m; eventDate: 10/15/1997; habitat: montane rainforest; fieldNumber: BLF01747; eventRemarks: sifted litter (leaf mold, rotten wood); **Record Level:** institutionCode: CASC
- **Type status:** Other material. **Occurrence:** catalogNumber: casent0746694; recordedBy: B.L.Fisher; sex: 1w; preparations: pin; associatedMedia: http://www.antweb.org/specimen/casent0746694; **Taxon:** scientificName: Stigmatomma liebe; genus: Stigmatomma; **Location:** country: Madagascar; stateProvince: Fianarantsoa; locality: 40 km S Ambalavao, Rés. Andringitra; verbatimElevation: 1275; decimalLatitude: -22.21667; decimalLongitude: 46.96667; **Event:** samplingProtocol: MW 50 sample transect, 5m; eventDate: 10/15/1993; habitat: montane rainforest; fieldNumber: BLF00793; eventRemarks: sifted litter (leaf mold, rotten wood); **Record Level:** institutionCode: CASC
- **Type status:** Other material. **Occurrence:** catalogNumber: casent0746695; recordedBy: B.L.Fisher; sex: 1w; preparations: pin; associatedMedia: http://www.antweb.org/specimen/casent0746695; **Taxon:** scientificName: Stigmatomma liebe; genus: Stigmatomma; **Location:** country: Madagascar; stateProvince: Toliara; locality: 13 km NW Enakara, Rés. Andohahela; verbatimElevation: 1250; decimalLatitude: -24.55; decimalLongitude: 46.8; **Event:** samplingProtocol: MW 50 sample transect, 5m; eventDate: 11/30/1992; habitat: montane rainforest; fieldNumber: BLF00561; eventRemarks: sifted litter (leaf mold, rotten wood); **Record Level:** institutionCode: CASC
- **Type status:** Other material. **Occurrence:** catalogNumber: casent0746696; recordedBy: B.L.Fisher; sex: 1w; preparations: pin; associatedMedia: http://www.antweb.org/specimen/casent0746696; **Taxon:** scientificName: Stigmatomma liebe; genus: Stigmatomma; **Location:** country: Madagascar; stateProvince: Toliara; locality: 13 km NW Enakara, Rés. Andohahela; verbatimElevation: 1250; decimalLatitude: -24.55; decimalLongitude: 46.8; **Event:** samplingProtocol: MW 50 sample transect, 5m; eventDate: 11/30/1992; habitat: montane rainforest; fieldNumber: BLF00561; eventRemarks: sifted litter (leaf mold, rotten wood); **Record Level:** institutionCode: CASC
- **Type status:** Other material. **Occurrence:** catalogNumber: casent0746697; recordedBy: B.L.Fisher (Sylvain); sex: 1w; preparations: pin; associatedMedia: http://www.antweb.org/specimen/casent0746697; **Taxon:** scientificName: Stigmatomma liebe; genus: Stigmatomma; **Location:** country: Madagascar; stateProvince: Fianarantsoa; locality: R.S. Ivohibe 8.0 km E Ivohibe; verbatimElevation: 1200; decimalLatitude: -22.48333; decimalLongitude: 46.96833; **Event:** samplingProtocol: MW 50 sample transect, 5m; eventDate: 10/15/1997; habitat: montane rainforest; fieldNumber: BLF01747; eventRemarks: sifted litter (leaf mold, rotten wood); **Record Level:** institutionCode: CASC
- **Type status:** Other material. **Occurrence:** catalogNumber: casent0746698; recordedBy: B.L.Fisher (Sylvain); sex: 1w; preparations: pin; associatedMedia: http://www.antweb.org/specimen/casent0746698; **Taxon:** scientificName: Stigmatomma liebe; genus: Stigmatomma; **Location:** country: Madagascar; stateProvince: Fianarantsoa; locality: R.S. Ivohibe 8.0 km E Ivohibe; verbatimElevation: 1200; decimalLatitude: -22.48333; decimalLongitude: 46.96833; **Event:** samplingProtocol: MW 50 sample transect, 5m; eventDate: 10/15/1997; habitat: montane rainforest; fieldNumber: BLF01747; eventRemarks: sifted litter (leaf mold, rotten wood); **Record Level:** institutionCode: CASC
- **Type status:** Other material. **Occurrence:** catalogNumber: casent0724179; recordedBy: B.L.Fisher, F.A.Esteves et al.; sex: 1w; preparations: pin; associatedMedia: http://www.antweb.org/specimen/casent0724179; **Taxon:** scientificName: Stigmatomma liebe; genus: Stigmatomma; **Location:** country: Madagascar; stateProvince: Toliara; locality: Anosy Region, Anosyenne Mts, 31.2 km NW Manantenina; verbatimElevation: 1125; decimalLatitude: -24.13894; decimalLongitude: 47.06804; **Event:** samplingProtocol: general collection; eventDate: 02/26/2015; habitat: montane rainforest; fieldNumber: BLF36518; eventRemarks: under root mat on rock; **Record Level:** institutionCode: CASC
- **Type status:** Other material. **Occurrence:** catalogNumber: casent0724177; recordedBy: B.L.Fisher, F.A.Esteves et al.; sex: 1w.1aq.; preparations: pin; associatedMedia: http://www.antweb.org/specimen/casent0724177; **Taxon:** scientificName: Stigmatomma liebe; genus: Stigmatomma; **Location:** country: Madagascar; stateProvince: Toliara; locality: Anosy Region, Anosyenne Mts, 31.2 km NW Manantenina; verbatimElevation: 1125; decimalLatitude: -24.13894; decimalLongitude: 47.06804; **Event:** samplingProtocol: general collection; eventDate: 02/26/2015; habitat: montane rainforest; fieldNumber: BLF36518; eventRemarks: under root mat on rock; **Record Level:** institutionCode: CASC
- **Type status:** Other material. **Occurrence:** catalogNumber: casent0724178; recordedBy: B.L.Fisher, F.A.Esteves et al.; sex: 1w; preparations: pin; associatedMedia: http://www.antweb.org/specimen/casent0724178; **Taxon:** scientificName: Stigmatomma liebe; genus: Stigmatomma; **Location:** country: Madagascar; stateProvince: Toliara; locality: Anosy Region, Anosyenne Mts, 31.2 km NW Manantenina; verbatimElevation: 1125; decimalLatitude: -24.13894; decimalLongitude: 47.06804; **Event:** samplingProtocol: general collection; eventDate: 02/26/2015; habitat: montane rainforest; fieldNumber: BLF36518; eventRemarks: under root mat on rock; **Record Level:** institutionCode: CASC
- **Type status:** Other material. **Occurrence:** catalogNumber: casent0723207; recordedBy: B.L.Fisher, F.A.Esteves et al.; sex: 1w; preparations: pin; associatedMedia: http://www.antweb.org/specimen/casent0723207; **Taxon:** scientificName: Stigmatomma liebe; genus: Stigmatomma; **Location:** country: Madagascar; stateProvince: Toliara; locality: Anosy Region, Anosyenne Mts, 31.2 km NW Manantenina; verbatimElevation: 1315; decimalLatitude: -24.13632; decimalLongitude: 47.05485; **Event:** samplingProtocol: general collection; eventDate: 02/27/2015; habitat: montane rainforest; fieldNumber: BLF36612; eventRemarks: ex root mat; **Record Level:** institutionCode: CASC
- **Type status:** Other material. **Occurrence:** catalogNumber: casent0723242; recordedBy: B.L.Fisher, F.A.Esteves et al.; sex: 1w; preparations: pin; associatedMedia: http://www.antweb.org/specimen/casent0723242; **Taxon:** scientificName: Stigmatomma liebe; genus: Stigmatomma; **Location:** country: Madagascar; stateProvince: Toliara; locality: Anosy Region, Anosyenne Mts, 31.2 km NW Manantenina; verbatimElevation: 1315; decimalLatitude: -24.13632; decimalLongitude: 47.05485; **Event:** samplingProtocol: general collection; eventDate: 02/27/2015; habitat: montane rainforest; fieldNumber: BLF36612; eventRemarks: ex root mat; **Record Level:** institutionCode: CASC
- **Type status:** Other material. **Occurrence:** catalogNumber: casent0723243; recordedBy: B.L.Fisher, F.A.Esteves et al.; sex: 1w; preparations: pin; associatedMedia: http://www.antweb.org/specimen/casent0723243; **Taxon:** scientificName: Stigmatomma liebe; genus: Stigmatomma; **Location:** country: Madagascar; stateProvince: Toliara; locality: Anosy Region, Anosyenne Mts, 31.2 km NW Manantenina; verbatimElevation: 1315; decimalLatitude: -24.13632; decimalLongitude: 47.05485; **Event:** samplingProtocol: general collection; eventDate: 02/27/2015; habitat: montane rainforest; fieldNumber: BLF36612; eventRemarks: ex root mat; **Record Level:** institutionCode: CASC
- **Type status:** Other material. **Occurrence:** catalogNumber: casent0723244; recordedBy: B.L.Fisher, F.A.Esteves et al.; sex: 1w; preparations: pin; associatedMedia: http://www.antweb.org/specimen/casent0723244; **Taxon:** scientificName: Stigmatomma liebe; genus: Stigmatomma; **Location:** country: Madagascar; stateProvince: Toliara; locality: Anosy Region, Anosyenne Mts, 31.2 km NW Manantenina; verbatimElevation: 1315; decimalLatitude: -24.13632; decimalLongitude: 47.05485; **Event:** samplingProtocol: general collection; eventDate: 02/27/2015; habitat: montane rainforest; fieldNumber: BLF36612; eventRemarks: ex root mat; **Record Level:** institutionCode: CASC
- **Type status:** Other material. **Occurrence:** catalogNumber: casent0723245; recordedBy: B.L.Fisher, F.A.Esteves et al.; sex: 1w; preparations: pin; associatedMedia: http://www.antweb.org/specimen/casent0723245; **Taxon:** scientificName: Stigmatomma liebe; genus: Stigmatomma; **Location:** country: Madagascar; stateProvince: Toliara; locality: Anosy Region, Anosyenne Mts, 31.2 km NW Manantenina; verbatimElevation: 1315; decimalLatitude: -24.13632; decimalLongitude: 47.05485; **Event:** samplingProtocol: general collection; eventDate: 02/27/2015; habitat: montane rainforest; fieldNumber: BLF36612; eventRemarks: ex root mat; **Record Level:** institutionCode: CASC
- **Type status:** Other material. **Occurrence:** catalogNumber: casent0723297; recordedBy: B.L.Fisher, F.A.Esteves et al.; sex: 1w; preparations: pin; associatedMedia: http://www.antweb.org/specimen/casent0723297; **Taxon:** scientificName: Stigmatomma liebe; genus: Stigmatomma; **Location:** country: Madagascar; stateProvince: Toliara; locality: Anosy Region, Anosyenne Mts, 31.2 km NW Manantenina; verbatimElevation: 1315; decimalLatitude: -24.13632; decimalLongitude: 47.05485; **Event:** samplingProtocol: general collection; eventDate: 02/27/2015; habitat: montane rainforest; fieldNumber: BLF36602; eventRemarks: under root mat on rock; **Record Level:** institutionCode: CASC
- **Type status:** Other material. **Occurrence:** catalogNumber: casent0723298; recordedBy: B.L.Fisher, F.A.Esteves et al.; sex: 1w; preparations: pin; associatedMedia: http://www.antweb.org/specimen/casent0723298; **Taxon:** scientificName: Stigmatomma liebe; genus: Stigmatomma; **Location:** country: Madagascar; stateProvince: Toliara; locality: Anosy Region, Anosyenne Mts, 31.2 km NW Manantenina; verbatimElevation: 1315; decimalLatitude: -24.13632; decimalLongitude: 47.05485; **Event:** samplingProtocol: general collection; eventDate: 02/27/2015; habitat: montane rainforest; fieldNumber: BLF36602; eventRemarks: under root mat on rock; **Record Level:** institutionCode: CASC
- **Type status:** Other material. **Occurrence:** catalogNumber: casent0723299; recordedBy: B.L.Fisher, F.A.Esteves et al.; sex: 1w; preparations: pin; associatedMedia: http://www.antweb.org/specimen/casent0723299; **Taxon:** scientificName: Stigmatomma liebe; genus: Stigmatomma; **Location:** country: Madagascar; stateProvince: Toliara; locality: Anosy Region, Anosyenne Mts, 31.2 km NW Manantenina; verbatimElevation: 1315; decimalLatitude: -24.13632; decimalLongitude: 47.05485; **Event:** samplingProtocol: general collection; eventDate: 02/27/2015; habitat: montane rainforest; fieldNumber: BLF36602; eventRemarks: under root mat on rock; **Record Level:** institutionCode: CASC
- **Type status:** Other material. **Occurrence:** catalogNumber: casent0723300; recordedBy: B.L.Fisher, F.A.Esteves et al.; sex: 1w; preparations: pin; associatedMedia: http://www.antweb.org/specimen/casent0723300; **Taxon:** scientificName: Stigmatomma liebe; genus: Stigmatomma; **Location:** country: Madagascar; stateProvince: Toliara; locality: Anosy Region, Anosyenne Mts, 31.2 km NW Manantenina; verbatimElevation: 1315; decimalLatitude: -24.13632; decimalLongitude: 47.05485; **Event:** samplingProtocol: general collection; eventDate: 02/27/2015; habitat: montane rainforest; fieldNumber: BLF36602; eventRemarks: under root mat on rock; **Record Level:** institutionCode: CASC
- **Type status:** Other material. **Occurrence:** catalogNumber: casent0723301; recordedBy: B.L.Fisher, F.A.Esteves et al.; sex: 1w; preparations: pin; associatedMedia: http://www.antweb.org/specimen/casent0723301; **Taxon:** scientificName: Stigmatomma liebe; genus: Stigmatomma; **Location:** country: Madagascar; stateProvince: Toliara; locality: Anosy Region, Anosyenne Mts, 31.2 km NW Manantenina; verbatimElevation: 1315; decimalLatitude: -24.13632; decimalLongitude: 47.05485; **Event:** samplingProtocol: general collection; eventDate: 02/27/2015; habitat: montane rainforest; fieldNumber: BLF36602; eventRemarks: under root mat on rock; **Record Level:** institutionCode: CASC
- **Type status:** Other material. **Occurrence:** catalogNumber: casent0723302; recordedBy: B.L.Fisher, F.A.Esteves et al.; sex: 1w; preparations: pin; associatedMedia: http://www.antweb.org/specimen/casent0723302; **Taxon:** scientificName: Stigmatomma liebe; genus: Stigmatomma; **Location:** country: Madagascar; stateProvince: Toliara; locality: Anosy Region, Anosyenne Mts, 31.2 km NW Manantenina; verbatimElevation: 1315; decimalLatitude: -24.13632; decimalLongitude: 47.05485; **Event:** samplingProtocol: general collection; eventDate: 02/27/2015; habitat: montane rainforest; fieldNumber: BLF36602; eventRemarks: under root mat on rock; **Record Level:** institutionCode: CASC
- **Type status:** Other material. **Occurrence:** catalogNumber: casent0723227; recordedBy: B.L.Fisher, F.A.Esteves et al.; sex: 1w; preparations: pin; associatedMedia: http://www.antweb.org/specimen/casent0723227; **Taxon:** scientificName: Stigmatomma liebe; genus: Stigmatomma; **Location:** country: Madagascar; stateProvince: Toliara; locality: Anosy Region, Anosyenne Mts, 31.2 km NW Manantenina; verbatimElevation: 1125; decimalLatitude: -24.13894; decimalLongitude: 47.06804; **Event:** samplingProtocol: general collection; eventDate: 02/26/2015; habitat: montane rainforest; fieldNumber: BLF36491; eventRemarks: ex root mat; **Record Level:** institutionCode: CASC
- **Type status:** Other material. **Occurrence:** catalogNumber: casent0723228; recordedBy: B.L.Fisher, F.A.Esteves et al.; sex: 1w; preparations: pin; associatedMedia: http://www.antweb.org/specimen/casent0723228; **Taxon:** scientificName: Stigmatomma liebe; genus: Stigmatomma; **Location:** country: Madagascar; stateProvince: Toliara; locality: Anosy Region, Anosyenne Mts, 31.2 km NW Manantenina; verbatimElevation: 1125; decimalLatitude: -24.13894; decimalLongitude: 47.06804; **Event:** samplingProtocol: general collection; eventDate: 02/26/2015; habitat: montane rainforest; fieldNumber: BLF36491; eventRemarks: ex root mat; **Record Level:** institutionCode: CASC
- **Type status:** Other material. **Occurrence:** catalogNumber: casent0723229; recordedBy: B.L.Fisher, F.A.Esteves et al.; sex: 1w; preparations: pin; associatedMedia: http://www.antweb.org/specimen/casent0723229; **Taxon:** scientificName: Stigmatomma liebe; genus: Stigmatomma; **Location:** country: Madagascar; stateProvince: Toliara; locality: Anosy Region, Anosyenne Mts, 31.2 km NW Manantenina; verbatimElevation: 1125; decimalLatitude: -24.13894; decimalLongitude: 47.06804; **Event:** samplingProtocol: general collection; eventDate: 02/26/2015; habitat: montane rainforest; fieldNumber: BLF36491; eventRemarks: ex root mat; **Record Level:** institutionCode: CASC
- **Type status:** Other material. **Occurrence:** catalogNumber: casent0723230; recordedBy: B.L.Fisher, F.A.Esteves et al.; sex: 1w.1m.; preparations: pin; associatedMedia: http://www.antweb.org/specimen/casent0723230; **Taxon:** scientificName: Stigmatomma liebe; genus: Stigmatomma; **Location:** country: Madagascar; stateProvince: Toliara; locality: Anosy Region, Anosyenne Mts, 31.2 km NW Manantenina; verbatimElevation: 1125; decimalLatitude: -24.13894; decimalLongitude: 47.06804; **Event:** samplingProtocol: general collection; eventDate: 02/26/2015; habitat: montane rainforest; fieldNumber: BLF36491; eventRemarks: ex root mat; **Record Level:** institutionCode: CASC
- **Type status:** Other material. **Occurrence:** catalogNumber: casent0723231; recordedBy: B.L.Fisher, F.A.Esteves et al.; sex: 1w.1m.; preparations: pin; associatedMedia: http://www.antweb.org/specimen/casent0723231; **Taxon:** scientificName: Stigmatomma liebe; genus: Stigmatomma; **Location:** country: Madagascar; stateProvince: Toliara; locality: Anosy Region, Anosyenne Mts, 31.2 km NW Manantenina; verbatimElevation: 1125; decimalLatitude: -24.13894; decimalLongitude: 47.06804; **Event:** samplingProtocol: general collection; eventDate: 02/26/2015; habitat: montane rainforest; fieldNumber: BLF36491; eventRemarks: ex root mat; **Record Level:** institutionCode: CASC
- **Type status:** Other material. **Occurrence:** catalogNumber: casent0723232; recordedBy: B.L.Fisher, F.A.Esteves et al.; sex: 1w; preparations: pin; associatedMedia: http://www.antweb.org/specimen/casent0723232; **Taxon:** scientificName: Stigmatomma liebe; genus: Stigmatomma; **Location:** country: Madagascar; stateProvince: Toliara; locality: Anosy Region, Anosyenne Mts, 31.2 km NW Manantenina; verbatimElevation: 1125; decimalLatitude: -24.13894; decimalLongitude: 47.06804; **Event:** samplingProtocol: general collection; eventDate: 02/26/2015; habitat: montane rainforest; fieldNumber: BLF36491; eventRemarks: ex root mat; **Record Level:** institutionCode: CASC
- **Type status:** Other material. **Occurrence:** catalogNumber: casent0724171; recordedBy: B.L.Fisher, F.A.Esteves et al.; sex: 1w.1m.; preparations: pin, slide; associatedMedia: http://www.antweb.org/specimen/casent0724171; **Taxon:** scientificName: Stigmatomma liebe; genus: Stigmatomma; **Location:** country: Madagascar; stateProvince: Toliara; locality: Anosy Region, Anosyenne Mts, 31.2 km NW Manantenina; verbatimElevation: 1125; decimalLatitude: -24.13894; decimalLongitude: 47.06804; **Event:** samplingProtocol: general collection; eventDate: 02/26/2015; habitat: montane rainforest; fieldNumber: BLF36453; eventRemarks: under root mat on rock; **Record Level:** institutionCode: CASC
- **Type status:** Other material. **Occurrence:** catalogNumber: casent0724172; recordedBy: B.L.Fisher, F.A.Esteves et al.; sex: 1w; preparations: SEM mount; associatedMedia: http://www.antweb.org/specimen/casent0724172; **Taxon:** scientificName: Stigmatomma liebe; genus: Stigmatomma; **Location:** country: Madagascar; stateProvince: Toliara; locality: Anosy Region, Anosyenne Mts, 31.2 km NW Manantenina; verbatimElevation: 1125; decimalLatitude: -24.13894; decimalLongitude: 47.06804; **Event:** samplingProtocol: general collection; eventDate: 02/26/2015; habitat: montane rainforest; fieldNumber: BLF36453; eventRemarks: under root mat on rock; **Record Level:** institutionCode: CASC
- **Type status:** Other material. **Occurrence:** catalogNumber: casent0724173; recordedBy: B.L.Fisher, F.A.Esteves et al.; sex: 1w; preparations: pin; associatedMedia: http://www.antweb.org/specimen/casent0724173; **Taxon:** scientificName: Stigmatomma liebe; genus: Stigmatomma; **Location:** country: Madagascar; stateProvince: Toliara; locality: Anosy Region, Anosyenne Mts, 31.2 km NW Manantenina; verbatimElevation: 1125; decimalLatitude: -24.13894; decimalLongitude: 47.06804; **Event:** samplingProtocol: general collection; eventDate: 02/26/2015; habitat: montane rainforest; fieldNumber: BLF36453; eventRemarks: under root mat on rock; **Record Level:** institutionCode: CASC
- **Type status:** Other material. **Occurrence:** catalogNumber: casent0724174; recordedBy: B.L.Fisher, F.A.Esteves et al.; sex: 1w; preparations: pin; associatedMedia: http://www.antweb.org/specimen/casent0724174; **Taxon:** scientificName: Stigmatomma liebe; genus: Stigmatomma; **Location:** country: Madagascar; stateProvince: Toliara; locality: Anosy Region, Anosyenne Mts, 31.2 km NW Manantenina; verbatimElevation: 1125; decimalLatitude: -24.13894; decimalLongitude: 47.06804; **Event:** samplingProtocol: general collection; eventDate: 02/26/2015; habitat: montane rainforest; fieldNumber: BLF36453; eventRemarks: under root mat on rock; **Record Level:** institutionCode: CASC
- **Type status:** Other material. **Occurrence:** catalogNumber: casent0724175; recordedBy: B.L.Fisher, F.A.Esteves et al.; sex: 1w; preparations: pin; associatedMedia: http://www.antweb.org/specimen/casent0724175; **Taxon:** scientificName: Stigmatomma liebe; genus: Stigmatomma; **Location:** country: Madagascar; stateProvince: Toliara; locality: Anosy Region, Anosyenne Mts, 31.2 km NW Manantenina; verbatimElevation: 1125; decimalLatitude: -24.13894; decimalLongitude: 47.06804; **Event:** samplingProtocol: general collection; eventDate: 02/26/2015; habitat: montane rainforest; fieldNumber: BLF36453; eventRemarks: under root mat on rock; **Record Level:** institutionCode: CASC
- **Type status:** Other material. **Occurrence:** catalogNumber: casent0724176; recordedBy: B.L.Fisher, F.A.Esteves et al.; sex: 1w; preparations: pin; associatedMedia: http://www.antweb.org/specimen/casent0724176; **Taxon:** scientificName: Stigmatomma liebe; genus: Stigmatomma; **Location:** country: Madagascar; stateProvince: Toliara; locality: Anosy Region, Anosyenne Mts, 31.2 km NW Manantenina; verbatimElevation: 1125; decimalLatitude: -24.13894; decimalLongitude: 47.06804; **Event:** samplingProtocol: general collection; eventDate: 02/26/2015; habitat: montane rainforest; fieldNumber: BLF36453; eventRemarks: under root mat on rock; **Record Level:** institutionCode: CASC
- **Type status:** Other material. **Occurrence:** catalogNumber: casent0721030; recordedBy: B.L.Fisher, F.A.Esteves et al.; sex: 1w; preparations: pin; associatedMedia: http://www.antweb.org/specimen/casent0721030; **Taxon:** scientificName: Stigmatomma liebe; genus: Stigmatomma; **Location:** country: Madagascar; stateProvince: Toliara; locality: Anosy Region, Anosyenne Mts, 31.2 km NW Manantenina; verbatimElevation: 1125; decimalLatitude: -24.13401; decimalLongitude: 47.05675; **Event:** samplingProtocol: 10 maxi winks; eventDate: 02/25/2015; habitat: montane rainforest; fieldNumber: BLF36450; eventRemarks: sifted litter (leaf mold, rotten wood); **Record Level:** institutionCode: CASC
- **Type status:** Other material. **Occurrence:** catalogNumber: casent0721032; recordedBy: B.L.Fisher, F.A.Esteves et al.; sex: 1w; preparations: pin; associatedMedia: http://www.antweb.org/specimen/casent0721032; **Taxon:** scientificName: Stigmatomma liebe; genus: Stigmatomma; **Location:** country: Madagascar; stateProvince: Toliara; locality: Anosy Region, Anosyenne Mts, 31.2 km NW Manantenina; verbatimElevation: 1125; decimalLatitude: -24.13401; decimalLongitude: 47.05675; **Event:** samplingProtocol: 10 maxi winks; eventDate: 02/25/2015; habitat: montane rainforest; fieldNumber: BLF36450; eventRemarks: sifted litter (leaf mold, rotten wood); **Record Level:** institutionCode: CASC
- **Type status:** Other material. **Occurrence:** catalogNumber: casent0704855; recordedBy: B.L.Fisher, F.A.Esteves et al.; sex: 1w; preparations: pin; associatedMedia: http://www.antweb.org/specimen/casent0704855; **Taxon:** scientificName: Stigmatomma liebe; genus: Stigmatomma; **Location:** country: Madagascar; stateProvince: Toliara; locality: Anosy Region, Anosyenne Mts, 31.2 km NW Manantenina; verbatimElevation: 1125; decimalLatitude: -24.13401; decimalLongitude: 47.05675; **Event:** samplingProtocol: 10 maxi winks; eventDate: 02/25/2015; habitat: montane rainforest; fieldNumber: BLF36450; eventRemarks: sifted litter (leaf mold, rotten wood); **Record Level:** institutionCode: CASC

#### Description

Worker (Fig. 59; holotype values within parentheses): **HL**: 0.90-0.96 (0.96); **HW**: 0.76-0.83 (0.83); **HW2**: 0.69-0.73 (0.73); **SL**: 0.47-0.52 (0.52); **ML**: 0.54-0.60 (0.60); **WL**: 1.16-1.34 (1.25); **PPW**: 0.45-0.53 (0.49); **PtL**: 0.50-0.59 (0.53); **PtW**: 0.51-0.60 (0.57); **CI**: 84-87 (86); **SI**: 52-55 (54); **MI**: 60-65 (63); **PtI**: 94-98 (94). 

*Head*:

Mandibular baso-masticatory margin skirted dorsally by row of filiform setae; medially, by spatular setae; ventrally, by filiform setae (Fig. 60a). Mandibular dentition arrangement, from base to apex: single larger tooth; much smaller single tooth (same size of teeth arranged in pairs; CASENT0318413 lacks this tooth); four pairs of teeth (each tooth pair with same dimensions, fused basally, or most-basal tooth of dorsal tooth pairs much smaller); single preapical tooth; apical tooth (Fig. 60a​). Pairs of teeth similar in length along mandible's basoapical axis (Fig. 60a​). Anterior clypeal margin with seven to nine tubercle-like cuticular processes, arranged in a single row, anteriorly armed with asymmetrical, mucronate, dentiform setae (Fig. 60a​). Lateral-most cuticular process with row of smaller conical setae anterolaterally, continuing laterally along clypeal anterior margin, arising from flat cuticle (Fig. 60a​). Median clypeal cuticular processes with nearly same length of associated dentiform setae (Fig. 60a​). Pair of long, filiform setae on clypeal anterior margin, bordering the central-most cuticular processes. Median area of clypeus extending posteriorly between antennal sockets; frontoclypeal sulcus round (Fig. 60b). Supraclypeal area as shallow oval concavity (Fig. 60b). Twelve antennomeres. Genal teeth present (Fig. 60b). Compound eyes present or absent; widest diameter of compound eyes if present one to three ommatidia (Fig. 60c). Palpal formula: 4:3 (four maxillary, three labial; ​Fig. 60d).

*Mesosoma*:

In dorsal view, lateral margins of mesonotum continuous with posterior remainder of mesosoma, or expanded laterally (Fig. 61a). Metanotal suture well developed or absent (Fig. 61a). Sulcus divinding mesepisternum into anepisternum and katepisternum (Fig. 61b). Metathoracic spiracle slit-like, posterior margin swollen, surrounded ventroposteriorly by concentric sulcus (Fig. 61b). Propodeal spiracle round, slightly tilted posteriorly (Fig. 61b). Propodeal declivitous face slightly concave (Fig. 61a).

*Legs*:

Basoventral two-thirds to three-fourths of calcar of strigil lamellar (Fig. 62a, b). Anterior face of calcar of strigil with strap-like microtrichia (Fig. 62a); posterior face with lanceolate microtrichia (Fig. 62b). Multiple paddle-like setae on anteroventral face of protibia, next to calcar of strigil (Fig. 62a). Multiple paddle-like setae on anterior face of probasitarsus (Fig. 62a); stout setae on posterior face, parallel to comb of strigil (Fig. 62b). Apex of mesotibial inner face with one or two spurs [anterior spur may be present, but in the majority of specimens is reduced to a short, bud-like cuticular projection concealed by a fovea] (Fig. 62c). Slit-like sulcus on anterior face of mesobasitarsus (Fig. 62d). Stout filiform setae along inner face of mesobasitarsus. Apex of metatibial inner face with one or two visible metatibial spurs; when present, the anterior spur is simple and much smaller than posterior spur (less than 1/3 the length of the posterior spur), glabrous or mostly glabrous; when the anterior spur is not visible, a short bud-like cuticular projection concealed by a fovea is seen under higher magnification; posterior spur pectinate (Fig. 63a). Anterior face of posterior metatibial spur mostly glabrous, with few lanceolate microtrichia (Fig. 63a​); posterior face glabrous (Fig. 63b). Absence of longitudinal sulcus on anterodorsal face of metabasitarsus (Fig. 63c). Few blunt, stout setae on the base of inner face of metabasitarsus (Fig. 63c​). Stout setae along remainder of inner face of metabasitarsus (Fig. 63c​). Arolium on pro-, meso-, and metapretarsus.

*Metasoma*:

Petiole sessile (Fig. 64a). Ventroanterior margin of petiolar tergite anterior dorso-latero-ventral carina (Ward 1990) much shorter than anterior margin of subpetiolar process, in lateral view (Fig. 64a​). Ventral margin of subpetiolar process running posteriorly in a continuous line, or angled obtusely at midpoint (Fig. 64a​). Presence of fenestra on lateral face of subpetiolar process (Fig. 64a​). Petiolar proprioceptor zone a large, round concavity with numerous sensilla (Fig. 64b). Prora present (Fig. 64a, c). Scrobiculate sulcus between pretergite and postergite of abdominal segment III and presclerites and postsclerites of abdominal segment IV (Fig. 64c). Absence of stout setae on hypopygium (Fig. 64d).

**Figure 65.**
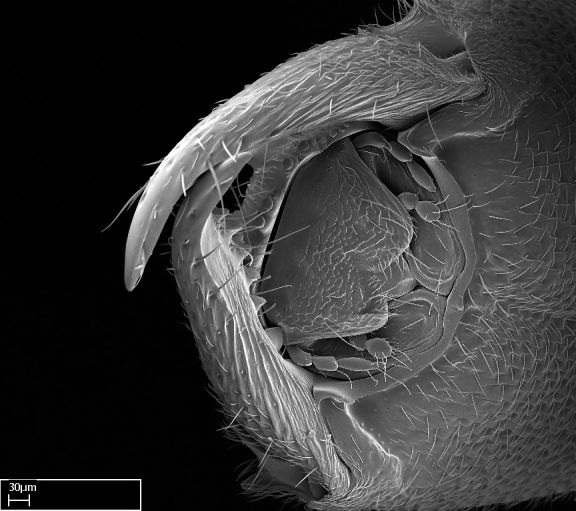
*Stigmatomma liebe* ​**sp. n.** worker (CASENT0009102): ventral view of the mandibles, mouthparts, and anterior part of the head. Image by F. A. Esteves; available at AntWeb.org.

*Sculpture*:

Mandibular dorsal face mostly costate-foveolate, except for smooth apical portion (Fig. 60a). Clypeal median area costate-dispersed foveolate (Fig. 60a). Supraclypeal area rugulose (Fig. 60b). Anterior three-fourths of the head, in dorsal view, costate-slightly catenate-foveolate, grading into foveolate posteriorly and laterally (Fig. 60b). Area posterior to tentorial pit tuberculate concentrically (Fig. 60b). Labrum imbricate (Fig. 65). Dorsal face of mesosoma densely foveolate (Fig. 61). Lateral face of pronotum densely foveolate-rugulose; anepisternum mostly smooth dorsally, grading into costate ventrally; katepisternum mostly confused costate-dispersed foveolate; metapleuron mostly costate (Fig. 61b). Lateral face of propodeum costate anteroventrally, grading into foveolate-rugulose posteriorly and dorsally; declivitous face foveolate-rugulose (Fig. 65). Anterior face of petiolar tergite smooth; lateral face imbricate anteriorly, grading into foveolate-rugulose laterally to foveolate dorsally; laterotergite mostly smooth or slightly imbricate; poststernite imbricate anteriorly, grading into alveolate posteriorly (Fig. 64a). Abdominal segments III foveolate; IV and V, punctate; VI and VII, weakly imbricate.

*Pilosity and color*:

Erect to subdecumbent pilosity on head, dorsal face of mesosoma, petiolar tergite, and abdominal segments III and IV. Erect to suberect pilosity on anterior half and along lateral margins of petiolar poststernite. Longer pilosity on abdominal segments V, VI, and VII. Body color dark-yellow to orange; yellow appendages.

##### Comments on character variation

The great majority of specimens examined present just one visible meso- and metatibial spur under the stereomicroscope; however, variation in number of meso- and metatibial spurs is seen in specimens of the same nest series. When just one spur is present on the meso- or metatibia, it is always the posterior spur; in such cases, a bud-like cuticular projection is seen concealed by a fovea at the place where the anterior spur would be located. It seems to us that such a projection is a sensillum and not the basal portion of a broken spur, given the developmental plasticity exhibited by the anterior mesotibial spur, when it is present: anterior and posterior spurs may have the same length in some specimens, or anterior spur may be much shorter (in one specimen, it corresponds to one-quarter of the size of the posterior spur). However, we do not discard the possibility that the anterior meso- and metatibial spur may be easily broken, but we could not infer that based on the SEM images we possess.

No geographic pattern is seen in the variation of characters of *Stigmatomma liebe*, and body size, the presence of the most-basal masticatory tooth, number of dentiform setae on clypeal anterior margin, degree of mesonotum expansion, number of meso- and metatibial spur, and color fluctuates even on specimens collected in the same locality.

##### Other castes

Gyne (Fig. 66a, b, c); alate when virgin: similar to the worker caste but for the greater body length, larger compound eyes, presence of ocelli, and differences on the mesosoma due to the presence of wings. Parapsidal lines on the mesoscutum; scuto-scutellar suture narrow, but scrobiculate (Fig. 67). Mesepisternum divided into anepisternum and katepisternum; mesepimeral lobe distinct; metapleuron divided into upper and lower sections; upper metapleuron separated from propodeum by a wide scrobiculate sulcus; lower metapleuron separated from propodeum by a carina, followed dorsally by a narrow, somewhat smooth sulcus (Fig. 67). Forewing (Fig. 66d): pterostigma well developed; Rs.f2 present as short stubs; Rs.f3 present; Rs.f5 present and reaching the R.f3; 1r-rs present, but incomplete; 2r-rs present; M.f4 present; 2rs-m present, but incomplete; Cu.f2, 1m-cu, and A.f2 present; cu-a intercepting M+Cu anteriorly to the separation point between M.f1 and Cu.f1. Hindwing (Fig. 66e): C indistinct; R slightly distinct; Rs.f2 and 1rs-m present; M.f2 present as a stub; Cu, cu-a, and A.f2 present.

Male (Fig. 68); alate: Mandibles falcate, with sharp, single apical tooth (Fig. 68a). Anterior clypeal margin armed with dentiform setae (Fig. 68a). Compound eyes with short setae among each ommatidium; sparse, longer setae present (Fig. 69). Palpal formula 4:3 (Fig. 70a). Notauli distinct; parapsidal lines present; scuto-scutellar suture scrobiculate (Fig. 69). Mesepisternum not divided into anepisternum and katepisternum; posterior oblique sulcus short, not well developed; mesepimeral lobe well developed; ventral third of the mesopleural suture scrobiculate; metapleuron divided into upper and lower sections by a sulcus; scrobiculate sulcus separating upper metapleuron from propodeum; lower metapleuron separated from the propodeum by a carina, followed dorsally by a slightly scrobiculate sulcus that decreases in width posteriorly (Fig. 69). Forewing (Fig. 71a​): pterostigma well developed; Rs.f2-3 present; Rs.f5 present and reaching R.f3; 1r-rs absent; 2r-rs, M.f4, 2rs-m, Cu.f2, 1m-cu, and A.f2 present; cu-a intercepting M+Cu at the separation point between M.f1 and Cu.f1. Hindwing (Fig. 71b): C indistinct; R slightly distinct; Rs.f2 as a indistinct stub; 1rs-m present; M.f2 absent; Cu, cu-a, and A.f2 present. Pygostyles present (Fig. 69d). Posterior margin of abdominal segment IX convex (Fig. 70d). Division of the paremere into telomere and basimere not visible. Digitus tongue-plier-shaped: presence of a comparatively enlarged projection at the base of the digitus; cuspis shorter than digitus (Fig. 70c). Entire ventral margin of the penisvalva strongly serrate; dorsal portion of the penisvalva somewhat sclerotized (Fig. 70b).

##### Specimens used in prior studies

*Stigmatomma liebe* was referenced as *Amblyopone* sp.2 (specimen CASENT0500013) in Saux et al. (2004).

#### Diagnosis

Worker

With characters of the *tsyhady* species-group and the *tsyhady* species-complex as described above, and the following characters (asterisks flag unique characters within the genus in the Malagasy bioregion):

1. Integument yellow to dark-yellow; medium-sized ant (HL: 0.90-0.96, WL: 1.16-1.34; Fig. 59).
2. Pairs of teeth along baso-masticatory margin of mandible are the same length along basoapical axis (Fig. 60a).
3. Spatular setae ventrally skirting baso-mastigatory margin of mandible (Fig. 60a).
4. Dorsal face of the head mostly costate-slightly catenate-foveolate (Fig. 60b).
5. Palpal formula 4:3 (Fig. 60d).
6. Dorsal face of mesosoma foveolate; lateral face of pronotum densely foveolate-rugulose; lateral face of propodeum costate anteroventrally, grading into foveolate-rugulose posteriorly and dorsally; propodeal declivitous face foveolate-rugulose (Fig. 61).
7. Mesepisternum divided into anepisternum and katepisternum (Fig. 61b).
8. Basoventral two-thirds of calcar of strigil lamellar (Fig. 62a, b).
9. Anterior face of calcar of strigil with strap-like microtrichia (Fig. 62a).
*Anterior mesotibial spur generally reduced to a bud-like cuticular projection concealed by a fovea. When the anterior mesotibial spur is developed, its length is extremely variable, ranging from the length of the posterior spur to one-quarter of its length.
11. Slit-like longitudinal sulcus present on the anterior face of mesobasitarsus (Fig. 62d).
*Anterior metatibial spur generally reduced to a bud-like cuticular projection concealed by a fovea. When developed, it is reduced in length (less than one-third of the length of the posterior spur), glabrous or mostly glabrous (Fig. 63a).
13. Anterior face of posterior metatibial spur mostly glabrous, posterior face glabrous (Fig. 63a).
14. Few blunt setae present on the baso-inner area of metabasitarsus (Fig. 63a, b, c).
15. Absence of a longitudinal sulcus on the anterior face of the metabasitarsus (Fig. 63c).
16. Ventral margin of the subpetiolar process generally runs continuously posteriorly, without forming a fin, but may be slightly obtusely angled at its mid-length (Fig. 64a).

Presence of genal teeth, palpal formula, presence of fenestra on the subpetiolar process, shape of microtrichia on the posterior face of posterior metatibial spur, and absence of stout setae on the apex of the hypopygium make *Stigmatomma liebe* similar to *S. irayhady, S. roahady*, and *S. tsyhady*.

However, the yellow color and smaller size differentiate it from the rest. Also, it possesses a sulcus on the anterodorsal face of the mesobasitarsus, while *S. tsyhady* does not; it does not have a sulcus on the anterodorsal face of the metabasitarsus, which is present in *S. roahady*; and the anterior metatibial spur is greatly reduced in size, meaning that in the great majority of specimens it is not visible under the stereomicroscope (when it is visible, its length corresponds to less than one-third of the length of the posterior metatial spur), while in *S. irayhady* it is always visible and much longer than half the length of the posterior metatibial spur.

*Stigmatomma liebe* is sympatric with *S. roahady* and *S. tsyhady* in four localities: at the Andohahela National Park, the Anosyenne Mountains, Andringitra Reserve, and the Ivohibe Special Reserve. It was not recorded at the localities *S. irayhady* was collected.

#### Etymology

The name liebe is homage to Elizabeth (Liebe) R. Patterson, for all the support she and her husband (*in memoriam*) have given to the myrmecological work being done in Madagascar.

#### Distribution

**Figure 72.**
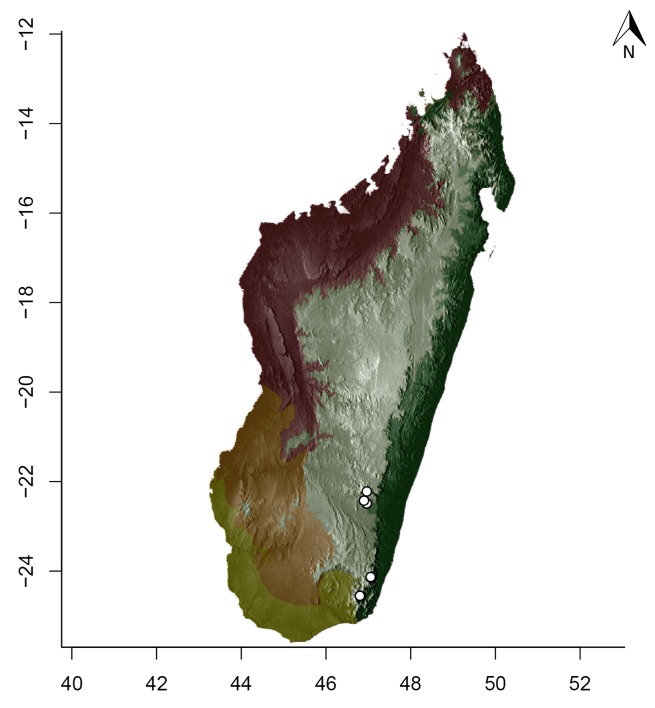
Distribution map of *Stigmatomma liebe* ​**sp. n.** in the Malagasy bioregion. Collection localities are mapped over the outlines of five simplified ecoregion zones of Madagascar: humid forests (dark green), subhumid forests (light green), dry deciduous forests (brown), succulent woodlands (orange), and spiny thickets (yellow).

*Stigmatomma liebe* was collected in montane rainforest habitats, above 1100 m, at the southern portion of the humid forests ecoregion of Madagascar (following the classification of Burgess et al. 2004; Fig. 72). Specimens were recorded from sifted leaf mold and rotten wood, and nesting in the root mat on rock and on soil.

### Stigmatomma roahady

Esteves & Fisher sp. n.

urn:lsid:zoobank.org:act:899EC29E-9400-4A32-9FE4-D6DA02784016

#### Materials

- **Type status:** Holotype. **Occurrence:** catalogNumber: casent0318421; recordedBy: B.L.Fisher; sex: 1w; preparations: pin; associatedMedia: http://www.antweb.org/specimen/casent0318421; **Taxon:** scientificName: Stigmatomma roahady; genus: Stigmatomma; **Location:** country: Madagascar; stateProvince: Toamasina; locality: Forêt Ambatovy, 14.3 km 57° Moramanga; verbatimElevation: 1075; decimalLatitude: -18.85083; decimalLongitude: 48.32; georeferenceRemarks: coordinates obtained from GPS; **Event:** samplingProtocol: General collecting; eventDate: 04/12/2005; habitat: montane rainforest; fieldNumber: BLF11961; eventRemarks: ex rotten log; **Record Level:** institutionCode: CASC
- **Type status:** Paratype. **Occurrence:** catalogNumber: casent0318422; recordedBy: B.L.Fisher; sex: 1w; preparations: pin; associatedMedia: http://www.antweb.org/specimen/casent0318422; **Taxon:** scientificName: Stigmatomma roahady; genus: Stigmatomma; **Location:** country: Madagascar; stateProvince: Toamasina; locality: Forêt Ambatovy, 14.3 km 57° Moramanga; verbatimElevation: 1075; decimalLatitude: -18.85083; decimalLongitude: 48.32; georeferenceRemarks: coordinates obtained from GPS; **Event:** samplingProtocol: General collecting; eventDate: 04/12/2005; habitat: montane rainforest; fieldNumber: BLF11961; eventRemarks: ex rotten log; **Record Level:** institutionCode: NHMW
- **Type status:** Paratype. **Occurrence:** catalogNumber: casent0318424; recordedBy: B.L.Fisher; sex: 1w; preparations: pin; associatedMedia: http://www.antweb.org/specimen/casent0318424; **Taxon:** scientificName: Stigmatomma roahady; genus: Stigmatomma; **Location:** country: Madagascar; stateProvince: Toamasina; locality: Forêt Ambatovy, 14.3 km 57° Moramanga; verbatimElevation: 1075; decimalLatitude: -18.85083; decimalLongitude: 48.32; georeferenceRemarks: coordinates obtained from GPS; **Event:** samplingProtocol: General collecting; eventDate: 04/12/2005; habitat: montane rainforest; fieldNumber: BLF11961; eventRemarks: ex rotten log; **Record Level:** institutionCode: USNM
- **Type status:** Paratype. **Occurrence:** catalogNumber: casent0318423; recordedBy: B.L.Fisher; sex: 1w; preparations: pin; associatedMedia: http://www.antweb.org/specimen/casent0318423; **Taxon:** scientificName: Stigmatomma roahady; genus: Stigmatomma; **Location:** country: Madagascar; stateProvince: Toamasina; locality: Forêt Ambatovy, 14.3 km 57° Moramanga; verbatimElevation: 1075; decimalLatitude: -18.85083; decimalLongitude: 48.32; georeferenceRemarks: coordinates obtained from GPS; **Event:** samplingProtocol: General collecting; eventDate: 04/12/2005; habitat: montane rainforest; fieldNumber: BLF11961; eventRemarks: ex rotten log; **Record Level:** institutionCode: MHNG
- **Type status:** Paratype. **Occurrence:** catalogNumber: casent0227519; recordedBy: B.L.Fisher; sex: 1w; preparations: pin; associatedMedia: http://www.antweb.org/specimen/casent0227519; **Taxon:** scientificName: Stigmatomma roahady; genus: Stigmatomma; **Location:** country: Madagascar; stateProvince: Toamasina; locality: Forêt Ambatovy, 14.3 km 57¡ Moramanga; verbatimElevation: 1075; decimalLatitude: -18.85083; decimalLongitude: 48.32; georeferenceRemarks: coordinates obtained from GPS; **Event:** samplingProtocol: General collecting; eventDate: 04/12/2005; habitat: montane rainforest; fieldNumber: BLF11961; eventRemarks: ex rotten log; **Record Level:** institutionCode: NHMB
- **Type status:** Other material. **Occurrence:** catalogNumber: casent0746592; recordedBy: B.L.Fisher; sex: 1w; preparations: pin; associatedMedia: http://www.antweb.org/specimen/casent0746592; **Taxon:** scientificName: Stigmatomma roahady; genus: Stigmatomma; **Location:** country: Madagascar; stateProvince: Antsiranana; locality: R.S. Manongarivo, 10.8 km 229° SW Antanambao; verbatimElevation: 400; decimalLatitude: -13.96167; decimalLongitude: 48.43333; **Event:** samplingProtocol: MW 75 sample transect, 5,10m; eventDate: 11/08/1998; habitat: rainforest; fieldNumber: BLF01996; eventRemarks: sifted litter (leaf mold, rotten wood); **Record Level:** institutionCode: CASC
- **Type status:** Other material. **Occurrence:** catalogNumber: casent0044932; recordedBy: B.L.Fisher et al.; sex: 1w; preparations: pin; associatedMedia: http://www.antweb.org/specimen/casent0044932; **Taxon:** scientificName: Stigmatomma roahady; genus: Stigmatomma; **Location:** country: Madagascar; stateProvince: Antsiranana; locality: Parc National de Marojejy, Manantenina River, 28.0 km 38° NE Andapa, 8.2 km 333° NNW Manantenina; verbatimElevation: 450; decimalLatitude: -14.43667; decimalLongitude: 49.775; georeferenceRemarks: coordinates obtained from GPS; **Event:** samplingProtocol: MW 25 sample transect, 5m; eventDate: 11/12/2003; habitat: rainforest; fieldNumber: BLF08722; eventRemarks: sifted litter (leaf mold, rotten wood); **Record Level:** institutionCode: CASC
- **Type status:** Other material. **Occurrence:** catalogNumber: casent0374094; recordedBy: B.L.Fisher et al.; sex: 1w; preparations: pin; associatedMedia: http://www.antweb.org/specimen/casent0374094; **Taxon:** scientificName: Stigmatomma roahady; genus: Stigmatomma; **Location:** country: Madagascar; stateProvince: Toamasina; locality: RNI Betampona; verbatimElevation: 489; decimalLatitude: -17.91015; decimalLongitude: 49.20318; georeferenceRemarks: ±200 m; **Event:** samplingProtocol: General collecting; eventDate: 09/23/2013; habitat: rainforest; fieldNumber: BLF31659; eventRemarks: ex soil; **Record Level:** institutionCode: CASC
- **Type status:** Other material. **Occurrence:** catalogNumber: casent0723206; recordedBy: B.L.Fisher, F.A.Esteves et al.; sex: 1w; preparations: pin; associatedMedia: http://www.antweb.org/specimen/casent0723206; **Taxon:** scientificName: Stigmatomma roahady; genus: Stigmatomma; **Location:** country: Madagascar; stateProvince: Toaliara; locality: Anosy Region, Anosyenne Mts, 29.33 km NW Manantenina; verbatimElevation: 540; decimalLatitude: -24.13993; decimalLongitude: 47.07418; georeferenceRemarks: ±50 m; **Event:** samplingProtocol: General collecting; eventDate: 02/23/2015; habitat: rainforest; fieldNumber: BLF36379; eventRemarks: ex soil; **Record Level:** institutionCode: CASC
- **Type status:** Other material. **Occurrence:** catalogNumber: casent0723241; recordedBy: B.L.Fisher, F.A.Esteves et al.; sex: 1w; preparations: pin; associatedMedia: http://www.antweb.org/specimen/casent0723241; **Taxon:** scientificName: Stigmatomma roahady; genus: Stigmatomma; **Location:** country: Madagascar; stateProvince: Toaliara; locality: Anosy Region, Anosyenne Mts, 29.33 km NW Manantenina; verbatimElevation: 540; decimalLatitude: -24.13993; decimalLongitude: 47.07418; georeferenceRemarks: ±50 m; **Event:** samplingProtocol: General collecting; eventDate: 02/23/2015; habitat: rainforest; fieldNumber: BLF36379; eventRemarks: ex soil; **Record Level:** institutionCode: CASC
- **Type status:** Other material. **Occurrence:** catalogNumber: casent0067259; recordedBy: B.L. Fisher et al.; sex: 1w; preparations: pin; associatedMedia: http://www.antweb.org/specimen/casent0067259; **Taxon:** scientificName: Stigmatomma roahady; genus: Stigmatomma; **Location:** country: Madagascar; stateProvince: Fianarantsoa; locality: Forêt de Vevembe, 66.6 km 293° Farafangana; verbatimElevation: 600; decimalLatitude: -22.791; decimalLongitude: 47.18183; georeferenceRemarks: coordinates obtained from GPS; **Event:** samplingProtocol: 9 Maxi winklers; eventDate: 04/23/2006; habitat: rainforest, transition to montane forest; fieldNumber: BLF14120; **Record Level:** institutionCode: CASC
- **Type status:** Other material. **Occurrence:** catalogNumber: casent0067261; recordedBy: B.L. Fisher et al.; sex: 1w; preparations: SEM mount; associatedMedia: http://www.antweb.org/specimen/casent0067261; **Taxon:** scientificName: Stigmatomma roahady; genus: Stigmatomma; **Location:** country: Madagascar; stateProvince: Fianarantsoa; locality: Forêt de Vevembe, 66.6 km 293° Farafangana; verbatimElevation: 600; decimalLatitude: -22.791; decimalLongitude: 47.18183; georeferenceRemarks: coordinates obtained from GPS; **Event:** samplingProtocol: 9 Maxi winklers; eventDate: 04/23/2006; habitat: rainforest, transition to montane forest; fieldNumber: BLF14120; **Record Level:** institutionCode: CASC
- **Type status:** Other material. **Occurrence:** catalogNumber: casent0454523; recordedBy: Fisher, Griswold et al.; sex: 1w; preparations: pin; associatedMedia: http://www.antweb.org/specimen/casent0454523; **Taxon:** scientificName: Stigmatomma roahady; genus: Stigmatomma; **Location:** country: Madagascar; stateProvince: Antsiranana; locality: Ampasindava, Forêt d'Ambilanivy, 3.9 km 181° S Ambaliha; verbatimElevation: 600; decimalLatitude: -13.79861; decimalLongitude: 48.16167; georeferenceRemarks: coordinates obtained from GPS; **Event:** samplingProtocol: MW 50 sample transect, 5m; eventDate: 03/04/2001; habitat: rainforest; fieldNumber: BLF03252; eventRemarks: sifted litter (leaf mold, rotten wood); **Record Level:** institutionCode: CASC
- **Type status:** Other material. **Occurrence:** catalogNumber: casent0454524; recordedBy: Fisher, Griswold et al.; sex: 1w; preparations: pin; associatedMedia: http://www.antweb.org/specimen/casent0454524; **Taxon:** scientificName: Stigmatomma roahady; genus: Stigmatomma; **Location:** country: Madagascar; stateProvince: Antsiranana; locality: Ampasindava, Forêt d'Ambilanivy, 3.9 km 181° S Ambaliha; verbatimElevation: 600; decimalLatitude: -13.79861; decimalLongitude: 48.16167; georeferenceRemarks: coordinates obtained from GPS; **Event:** samplingProtocol: MW 50 sample transect, 5m; eventDate: 03/04/2001; habitat: rainforest; fieldNumber: BLF03252; eventRemarks: sifted litter (leaf mold, rotten wood); **Record Level:** institutionCode: CASC
- **Type status:** Other material. **Occurrence:** catalogNumber: casent0275421; recordedBy: B.L.Fisher et al.; sex: 1w; preparations: pin; associatedMedia: http://www.antweb.org/specimen/casent0275421; **Taxon:** scientificName: Stigmatomma roahady; genus: Stigmatomma; **Location:** country: Madagascar; stateProvince: Toamasina; locality: Ankerana; verbatimElevation: 750; decimalLatitude: -18.40829; decimalLongitude: 48.82107; georeferenceRemarks: ±200 m; **Event:** samplingProtocol: 03 MaxiWinks, mixed samples; eventDate: 01/21/2012; habitat: rainforest; fieldNumber: BLF27931; eventRemarks: sifted litter; **Record Level:** institutionCode: CASC
- **Type status:** Other material. **Occurrence:** catalogNumber: casent0151725; recordedBy: B.L.Fisher et al.; sex: 1w; preparations: pin; associatedMedia: http://www.antweb.org/specimen/casent0151725; **Taxon:** scientificName: Stigmatomma roahady; genus: Stigmatomma; **Location:** country: Madagascar; stateProvince: Toamasina; locality: Parc National de Zahamena, Onibe River; verbatimElevation: 780; decimalLatitude: -17.75908; decimalLongitude: 48.85468; georeferenceRemarks: coordinates obtained from GPS; **Event:** samplingProtocol: 10 maxi winks; eventDate: 02/21/2009; habitat: rainforest; fieldNumber: BLF22214; eventRemarks: sifted litter (leaf mold, rotten wood); **Record Level:** institutionCode: CASC
- **Type status:** Other material. **Occurrence:** catalogNumber: casent0151727; recordedBy: B.L.Fisher et al.; sex: 1w; preparations: pin; associatedMedia: http://www.antweb.org/specimen/casent0151727; **Taxon:** scientificName: Stigmatomma roahady; genus: Stigmatomma; **Location:** country: Madagascar; stateProvince: Toamasina; locality: Parc National de Zahamena, Onibe River; verbatimElevation: 780; decimalLatitude: -17.75908; decimalLongitude: 48.85468; georeferenceRemarks: coordinates obtained from GPS; **Event:** samplingProtocol: 10 maxi winks; eventDate: 02/21/2009; habitat: rainforest; fieldNumber: BLF22214; eventRemarks: sifted litter (leaf mold, rotten wood); **Record Level:** institutionCode: CASC
- **Type status:** Other material. **Occurrence:** catalogNumber: casent0151728; recordedBy: B.L.Fisher et al.; sex: 1w; preparations: pin; associatedMedia: http://www.antweb.org/specimen/casent0151728; **Taxon:** scientificName: Stigmatomma roahady; genus: Stigmatomma; **Location:** country: Madagascar; stateProvince: Toamasina; locality: Parc National de Zahamena, Onibe River; verbatimElevation: 780; decimalLatitude: -17.75908; decimalLongitude: 48.85468; georeferenceRemarks: coordinates obtained from GPS; **Event:** samplingProtocol: 10 maxi winks; eventDate: 02/21/2009; habitat: rainforest; fieldNumber: BLF22214; eventRemarks: sifted litter (leaf mold, rotten wood); **Record Level:** institutionCode: CASC
- **Type status:** Other material. **Occurrence:** catalogNumber: casent0152150; recordedBy: B.L.Fisher et al.; sex: 1w; preparations: pin; associatedMedia: http://www.antweb.org/specimen/casent0152150; **Taxon:** scientificName: Stigmatomma roahady; genus: Stigmatomma; **Location:** country: Madagascar; stateProvince: Toamasina; locality: Parc National de Zahamena, Onibe River; verbatimElevation: 780; decimalLatitude: -17.75908; decimalLongitude: 48.85468; georeferenceRemarks: coordinates obtained from GPS; **Event:** samplingProtocol: General collecting; eventDate: 02/21/2009; habitat: rainforest; fieldNumber: BLF22184; eventRemarks: under rotten log; **Record Level:** institutionCode: CASC
- **Type status:** Other material. **Occurrence:** catalogNumber: casent0746587; recordedBy: B.L.Fisher; sex: 1w; preparations: pin; associatedMedia: http://www.antweb.org/specimen/casent0746587; **Taxon:** scientificName: Stigmatomma roahady; genus: Stigmatomma; **Location:** country: Madagascar; stateProvince: Fianarantsoa; locality: 43 km S Ambalavao, Rés. Andringitra; verbatimElevation: 825; decimalLatitude: -22.23333; decimalLongitude: 47; **Event:** samplingProtocol: MW 50 sample transect, 5m; eventDate: 10/05/1993; habitat: rainforest; fieldNumber: BLF00747; eventRemarks: sifted litter (leaf mold, rotten wood); **Record Level:** institutionCode: CASC
- **Type status:** Other material. **Occurrence:** catalogNumber: casent0150904; recordedBy: B.L.Fisher et al.; sex: 1w; preparations: pin; associatedMedia: http://www.antweb.org/specimen/casent0150904; **Taxon:** scientificName: Stigmatomma roahady; genus: Stigmatomma; **Location:** country: Madagascar; stateProvince: Toamasina; locality: Parc National de Zahamena, Tetezambatana forest, near junction of Nosivola and Manakambahiny Rivers; verbatimElevation: 860; decimalLatitude: -17.74298; decimalLongitude: 48.72936; georeferenceRemarks: coordinates obtained from GPS; **Event:** samplingProtocol: 10 maxi winks; eventDate: 02/18/2009; habitat: rainforest; fieldNumber: BLF21974; eventRemarks: sifted litter (leaf mold, rotten wood); **Record Level:** institutionCode: CASC
- **Type status:** Other material. **Occurrence:** catalogNumber: casent0746594; recordedBy: B.L.Fisher; sex: 3w; preparations: pin; associatedMedia: http://www.antweb.org/specimen/casent0746594; **Taxon:** scientificName: Stigmatomma roahady; genus: Stigmatomma; **Location:** country: Madagascar; stateProvince: Antsiranana; locality: 6.5 km SSW Befingotra, Rés. Anjanaharibe-Sud; verbatimElevation: 875; decimalLatitude: -14.75; decimalLongitude: 49.5; **Event:** samplingProtocol: MW 50 sample transect, 5m; eventDate: 10/19/1994; habitat: rainforest; fieldNumber: BLF01070; eventRemarks: sifted litter (leaf mold, rotten wood); **Record Level:** institutionCode: CASC
- **Type status:** Other material. **Occurrence:** catalogNumber: casent0009069; recordedBy: H.J.Ratsirarson; sex: 1w; preparations: pin; associatedMedia: http://www.antweb.org/specimen/casent0009069; **Taxon:** scientificName: Stigmatomma roahady; genus: Stigmatomma; **Location:** country: Madagascar; stateProvince: Toamasina; locality: P.N. Mantadia; verbatimElevation: 895; decimalLatitude: -18.79167; decimalLongitude: 48.42667; **Event:** samplingProtocol: MW 25 sample transect, 5m; eventDate: 11/25/1998; habitat: rainforest; fieldNumber: HJR111; eventRemarks: sifted litter (leaf mold, rotten wood); **Record Level:** institutionCode: CASC
- **Type status:** Other material. **Occurrence:** catalogNumber: casent0009070; recordedBy: H.J.Ratsirarson; sex: 1w; preparations: pin; associatedMedia: http://www.antweb.org/specimen/casent0009070; **Taxon:** scientificName: Stigmatomma roahady; genus: Stigmatomma; **Location:** country: Madagascar; stateProvince: Toamasina; locality: P.N. Mantadia; verbatimElevation: 895; decimalLatitude: -18.79167; decimalLongitude: 48.42667; **Event:** samplingProtocol: MW 25 sample transect, 5m; eventDate: 11/25/1998; habitat: rainforest; fieldNumber: HJR111; eventRemarks: sifted litter (leaf mold, rotten wood); **Record Level:** institutionCode: CASC
- **Type status:** Other material. **Occurrence:** catalogNumber: casent0746575; recordedBy: H.J.Ratsirarson; sex: 1w; preparations: pin; associatedMedia: http://www.antweb.org/specimen/casent0746575; **Taxon:** scientificName: Stigmatomma roahady; genus: Stigmatomma; **Location:** country: Madagascar; stateProvince: Toamasina; locality: P.N. Mantadia; verbatimElevation: 895; decimalLatitude: -18.79167; decimalLongitude: 48.42667; **Event:** samplingProtocol: MW 25 sample transect, 5m; eventDate: 11/25/1998; habitat: rainforest; fieldNumber: HJR111; eventRemarks: sifted litter (leaf mold, rotten wood); **Record Level:** institutionCode: CASC
- **Type status:** Other material. **Occurrence:** catalogNumber: casent0746576; recordedBy: H.J.Ratsirarson; sex: 1w; preparations: pin; associatedMedia: http://www.antweb.org/specimen/casent0746576; **Taxon:** scientificName: Stigmatomma roahady; genus: Stigmatomma; **Location:** country: Madagascar; stateProvince: Toamasina; locality: P.N. Mantadia; verbatimElevation: 895; decimalLatitude: -18.79167; decimalLongitude: 48.42667; **Event:** samplingProtocol: MW 25 sample transect, 5m; eventDate: 11/25/1998; habitat: rainforest; fieldNumber: HJR111; eventRemarks: sifted litter (leaf mold, rotten wood); **Record Level:** institutionCode: CASC
- **Type status:** Other material. **Occurrence:** catalogNumber: casent0746577; recordedBy: H.J.Ratsirarson; sex: 1w; preparations: pin; associatedMedia: http://www.antweb.org/specimen/casent0746577; **Taxon:** scientificName: Stigmatomma roahady; genus: Stigmatomma; **Location:** country: Madagascar; stateProvince: Toamasina; locality: P.N. Mantadia; verbatimElevation: 895; decimalLatitude: -18.79167; decimalLongitude: 48.42667; **Event:** samplingProtocol: MW 25 sample transect, 5m; eventDate: 11/25/1998; habitat: rainforest; fieldNumber: HJR111; eventRemarks: sifted litter (leaf mold, rotten wood); **Record Level:** institutionCode: CASC
- **Type status:** Other material. **Occurrence:** catalogNumber: hjr111(04)-7; recordedBy: H.J.Ratsirarson; sex: 1w; preparations: pin; associatedMedia: http://www.antweb.org/specimen/hjr111(04)-7; **Taxon:** scientificName: Stigmatomma roahady; genus: Stigmatomma; **Location:** country: Madagascar; stateProvince: Toamasina; locality: P.N. Mantadia; verbatimElevation: 895; decimalLatitude: -18.79167; decimalLongitude: 48.42667; **Event:** samplingProtocol: MW 25 sample transect, 5m; eventDate: 11/25/1998; habitat: rainforest; fieldNumber: HJR111; eventRemarks: sifted litter (leaf mold, rotten wood); **Record Level:** institutionCode: CASC
- **Type status:** Other material. **Occurrence:** catalogNumber: blf1745(19)-7; recordedBy: B.L.Fisher (Sylvain); sex: 1dQ; preparations: pin; associatedMedia: http://www.antweb.org/specimen/blf1745(19)-7; **Taxon:** scientificName: Stigmatomma roahady; genus: Stigmatomma; **Location:** country: Madagascar; stateProvince: Fianarantsoa; locality: R.S. Ivohibe, 7.5 km ENE Ivohibe; verbatimElevation: 900; decimalLatitude: -22.47; decimalLongitude: 46.96; **Event:** samplingProtocol: MW 50 sample transect, 5m; eventDate: 10/07/1997; habitat: rainforest; fieldNumber: BLF01745; eventRemarks: sifted litter (leaf mold, rotten wood); **Record Level:** institutionCode: CASC
- **Type status:** Other material. **Occurrence:** catalogNumber: blf1745(l.o.)-2; recordedBy: B.L.Fisher (Sylvain); sex: 1w; preparations: pin; associatedMedia: http://www.antweb.org/specimen/blf1745(l.o.)-2; **Taxon:** scientificName: Stigmatomma roahady; genus: Stigmatomma; **Location:** country: Madagascar; stateProvince: Fianarantsoa; locality: R.S. Ivohibe, 7.5 km ENE Ivohibe; verbatimElevation: 900; decimalLatitude: -22.47; decimalLongitude: 46.96; **Event:** samplingProtocol: MW 50 sample transect, 5m; eventDate: 10/07/1997; habitat: rainforest; fieldNumber: BLF01745; eventRemarks: sifted litter (leaf mold, rotten wood); **Record Level:** institutionCode: CASC
- **Type status:** Other material. **Occurrence:** catalogNumber: casent0001308; recordedBy: Fisher-Griswold Arthropod Team; sex: 1w; preparations: pin; associatedMedia: http://www.antweb.org/specimen/casent0001308; **Taxon:** scientificName: Stigmatomma roahady; genus: Stigmatomma; **Location:** country: Madagascar; stateProvince: Toliara; locality: Parc National d'Andohahela, Col du Sedro, 3.8 km 113° ESE Mahamavo, 37.6 km 341° NNW Tolagnaro; verbatimElevation: 900; decimalLatitude: -24.76389; decimalLongitude: 46.75167; georeferenceRemarks: coordinates obtained from GPS; **Event:** samplingProtocol: General collecting; eventDate: 01/21/2002; habitat: montane rainforest; fieldNumber: BLF05118; eventRemarks: ex root mat, ground layer; **Record Level:** institutionCode: CASC
- **Type status:** Other material. **Occurrence:** catalogNumber: casent0001309; recordedBy: Fisher-Griswold Arthropod Team; sex: 1w; preparations: pin; associatedMedia: http://www.antweb.org/specimen/casent0001309; **Taxon:** scientificName: Stigmatomma roahady; genus: Stigmatomma; **Location:** country: Madagascar; stateProvince: Toliara; locality: Parc National d'Andohahela, Col du Sedro, 3.8 km 113° ESE Mahamavo, 37.6 km 341° NNW Tolagnaro; verbatimElevation: 900; decimalLatitude: -24.76389; decimalLongitude: 46.75167; georeferenceRemarks: coordinates obtained from GPS; **Event:** samplingProtocol: General collecting; eventDate: 01/21/2002; habitat: montane rainforest; fieldNumber: BLF05118; eventRemarks: ex root mat, ground layer; **Record Level:** institutionCode: CASC
- **Type status:** Other material. **Occurrence:** catalogNumber: casent0008703; recordedBy: B.L.Fisher (Sylvain); sex: 1w; preparations: pin; associatedMedia: http://www.antweb.org/specimen/casent0008703; **Taxon:** scientificName: Stigmatomma roahady; genus: Stigmatomma; **Location:** country: Madagascar; stateProvince: Fianarantsoa; locality: R.S. Ivohibe, 7.5 km ENE Ivohibe; verbatimElevation: 900; decimalLatitude: -22.47; decimalLongitude: 46.96; **Event:** samplingProtocol: MW 50 sample transect, 5m; eventDate: 10/07/1997; habitat: rainforest; fieldNumber: BLF01745; eventRemarks: sifted litter (leaf mold, rotten wood); **Record Level:** institutionCode: CASC
- **Type status:** Other material. **Occurrence:** catalogNumber: casent0170205; recordedBy: W.E.Steiner; sex: 1aQ; preparations: pin; associatedMedia: http://www.antweb.org/specimen/casent0170205; **Taxon:** scientificName: Stigmatomma roahady; genus: Stigmatomma; **Location:** country: Madagascar; stateProvince: Fianarantsoa; locality: Ranomafana Nat. Park, 7km W; verbatimElevation: 900; **Event:** eventDate: 03/20/1990; fieldNumber: MCZ.2040; **Record Level:** institutionCode: CASC
- **Type status:** Other material. **Occurrence:** catalogNumber: casent0443017; recordedBy: Fisher-Griswold Arthropod Team; sex: 1w; preparations: pin; associatedMedia: http://www.antweb.org/specimen/casent0443017; **Taxon:** scientificName: Stigmatomma roahady; genus: Stigmatomma; **Location:** country: Madagascar; stateProvince: Toliara; locality: Parc National d'Andohahela, Col du Sedro, 3.8 km 113° ESE Mahamavo, 37.6 km 341° NNW Tolagnaro; verbatimElevation: 900; decimalLatitude: -24.76389; decimalLongitude: 46.75167; georeferenceRemarks: coordinates obtained from GPS; **Event:** samplingProtocol: General collecting; eventDate: 01/21/2002; habitat: montane rainforest; fieldNumber: BLF05117; eventRemarks: ex root mat, ground layer; **Record Level:** institutionCode: CASC
- **Type status:** Other material. **Occurrence:** catalogNumber: casent0443018; recordedBy: Fisher-Griswold Arthropod Team; sex: 1w; preparations: pin; associatedMedia: http://www.antweb.org/specimen/casent0443018; **Taxon:** scientificName: Stigmatomma roahady; genus: Stigmatomma; **Location:** country: Madagascar; stateProvince: Toliara; locality: Parc National d'Andohahela, Col du Sedro, 3.8 km 113° ESE Mahamavo, 37.6 km 341° NNW Tolagnaro; verbatimElevation: 900; decimalLatitude: -24.76389; decimalLongitude: 46.75167; georeferenceRemarks: coordinates obtained from GPS; **Event:** samplingProtocol: General collecting; eventDate: 01/21/2002; habitat: montane rainforest; fieldNumber: BLF05117; eventRemarks: ex root mat, ground layer; **Record Level:** institutionCode: CASC
- **Type status:** Other material. **Occurrence:** catalogNumber: casent0443019; recordedBy: Fisher-Griswold Arthropod Team; sex: 1w; preparations: pin; associatedMedia: http://www.antweb.org/specimen/casent0443019; **Taxon:** scientificName: Stigmatomma roahady; genus: Stigmatomma; **Location:** country: Madagascar; stateProvince: Toliara; locality: Parc National d'Andohahela, Col du Sedro, 3.8 km 113° ESE Mahamavo, 37.6 km 341° NNW Tolagnaro; verbatimElevation: 900; decimalLatitude: -24.76389; decimalLongitude: 46.75167; georeferenceRemarks: coordinates obtained from GPS; **Event:** samplingProtocol: General collecting; eventDate: 01/21/2002; habitat: montane rainforest; fieldNumber: BLF05117; eventRemarks: ex root mat, ground layer; **Record Level:** institutionCode: CASC
- **Type status:** Other material. **Occurrence:** catalogNumber: casent0443020; recordedBy: Fisher-Griswold Arthropod Team; sex: 1w (head detached); preparations: pin; associatedMedia: http://www.antweb.org/specimen/casent0443020; **Taxon:** scientificName: Stigmatomma roahady; genus: Stigmatomma; **Location:** country: Madagascar; stateProvince: Toliara; locality: Parc National d'Andohahela, Col du Sedro, 3.8 km 113° ESE Mahamavo, 37.6 km 341° NNW Tolagnaro; verbatimElevation: 900; decimalLatitude: -24.76389; decimalLongitude: 46.75167; georeferenceRemarks: coordinates obtained from GPS; **Event:** samplingProtocol: General collecting; eventDate: 01/21/2002; habitat: montane rainforest; fieldNumber: BLF05117; eventRemarks: ex root mat, ground layer; **Record Level:** institutionCode: CASC
- **Type status:** Other material. **Occurrence:** catalogNumber: casent0443021; recordedBy: Fisher-Griswold Arthropod Team; sex: 2w; preparations: pin; associatedMedia: http://www.antweb.org/specimen/casent0443021; **Taxon:** scientificName: Stigmatomma roahady; genus: Stigmatomma; **Location:** country: Madagascar; stateProvince: Toliara; locality: Parc National d'Andohahela, Col du Sedro, 3.8 km 113° ESE Mahamavo, 37.6 km 341° NNW Tolagnaro; verbatimElevation: 900; decimalLatitude: -24.76389; decimalLongitude: 46.75167; georeferenceRemarks: coordinates obtained from GPS; **Event:** samplingProtocol: General collecting; eventDate: 01/21/2002; habitat: montane rainforest; fieldNumber: BLF05117; eventRemarks: ex root mat, ground layer; **Record Level:** institutionCode: CASC
- **Type status:** Other material. **Occurrence:** catalogNumber: casent0443022; recordedBy: Fisher-Griswold Arthropod Team; sex: 1w; preparations: pin; associatedMedia: http://www.antweb.org/specimen/casent0443022; **Taxon:** scientificName: Stigmatomma roahady; genus: Stigmatomma; **Location:** country: Madagascar; stateProvince: Toliara; locality: Parc National d'Andohahela, Col du Sedro, 3.8 km 113° ESE Mahamavo, 37.6 km 341° NNW Tolagnaro; verbatimElevation: 900; decimalLatitude: -24.76389; decimalLongitude: 46.75167; georeferenceRemarks: coordinates obtained from GPS; **Event:** samplingProtocol: General collecting; eventDate: 01/21/2002; habitat: montane rainforest; fieldNumber: BLF05117; eventRemarks: ex root mat, ground layer; **Record Level:** institutionCode: CASC
- **Type status:** Other material. **Occurrence:** catalogNumber: casent0443023; recordedBy: Fisher-Griswold Arthropod Team; sex: 1w; preparations: pin; associatedMedia: http://www.antweb.org/specimen/casent0443023; **Taxon:** scientificName: Stigmatomma roahady; genus: Stigmatomma; **Location:** country: Madagascar; stateProvince: Toliara; locality: Parc National d'Andohahela, Col du Sedro, 3.8 km 113° ESE Mahamavo, 37.6 km 341° NNW Tolagnaro; verbatimElevation: 900; decimalLatitude: -24.76389; decimalLongitude: 46.75167; georeferenceRemarks: coordinates obtained from GPS; **Event:** samplingProtocol: General collecting; eventDate: 01/21/2002; habitat: montane rainforest; fieldNumber: BLF05117; eventRemarks: ex root mat, ground layer; **Record Level:** institutionCode: CASC
- **Type status:** Other material. **Occurrence:** catalogNumber: casent0478546; recordedBy: Fisher-Griswold Arthropod Team; sex: 1w; preparations: pin; associatedMedia: http://www.antweb.org/specimen/casent0478546; **Taxon:** scientificName: Stigmatomma roahady; genus: Stigmatomma; **Location:** country: Madagascar; stateProvince: Toliara; locality: Parc National d'Andohahela, Col du Sedro, 3.8 km 113° ESE Mahamavo, 37.6 km 341° NNW Tolagnaro; verbatimElevation: 900; decimalLatitude: -24.76389; decimalLongitude: 46.75167; georeferenceRemarks: coordinates obtained from GPS; **Event:** samplingProtocol: MW 50 sample transect, 5m; eventDate: 01/21/2002; habitat: montane rainforest; fieldNumber: BLF05010; eventRemarks: sifted litter (leaf mold, rotten wood); **Record Level:** institutionCode: CASC
- **Type status:** Other material. **Occurrence:** catalogNumber: casent0478547; recordedBy: Fisher-Griswold Arthropod Team; sex: 1w; preparations: pin; associatedMedia: http://www.antweb.org/specimen/casent0478547; **Taxon:** scientificName: Stigmatomma roahady; genus: Stigmatomma; **Location:** country: Madagascar; stateProvince: Toliara; locality: Parc National d'Andohahela, Col du Sedro, 3.8 km 113° ESE Mahamavo, 37.6 km 341° NNW Tolagnaro; verbatimElevation: 900; decimalLatitude: -24.76389; decimalLongitude: 46.75167; georeferenceRemarks: coordinates obtained from GPS; **Event:** samplingProtocol: MW 50 sample transect, 5m; eventDate: 01/21/2002; habitat: montane rainforest; fieldNumber: BLF05010; eventRemarks: sifted litter (leaf mold, rotten wood); **Record Level:** institutionCode: CASC
- **Type status:** Other material. **Occurrence:** catalogNumber: casent0478548; recordedBy: Fisher-Griswold Arthropod Team; sex: 1w; preparations: pin; associatedMedia: http://www.antweb.org/specimen/casent0478548; **Taxon:** scientificName: Stigmatomma roahady; genus: Stigmatomma; **Location:** country: Madagascar; stateProvince: Toliara; locality: Parc National d'Andohahela, Col du Sedro, 3.8 km 113° ESE Mahamavo, 37.6 km 341° NNW Tolagnaro; verbatimElevation: 900; decimalLatitude: -24.76389; decimalLongitude: 46.75167; georeferenceRemarks: coordinates obtained from GPS; **Event:** samplingProtocol: MW 50 sample transect, 5m; eventDate: 01/21/2002; habitat: montane rainforest; fieldNumber: BLF05010; eventRemarks: sifted litter (leaf mold, rotten wood); **Record Level:** institutionCode: CASC
- **Type status:** Other material. **Occurrence:** catalogNumber: casent0478549; recordedBy: Fisher-Griswold Arthropod Team; sex: 1w; preparations: pin; associatedMedia: http://www.antweb.org/specimen/casent0478549; **Taxon:** scientificName: Stigmatomma roahady; genus: Stigmatomma; **Location:** country: Madagascar; stateProvince: Toliara; locality: Parc National d'Andohahela, Col du Sedro, 3.8 km 113° ESE Mahamavo, 37.6 km 341° NNW Tolagnaro; verbatimElevation: 900; decimalLatitude: -24.76389; decimalLongitude: 46.75167; georeferenceRemarks: coordinates obtained from GPS; **Event:** samplingProtocol: MW 50 sample transect, 5m; eventDate: 01/21/2002; habitat: montane rainforest; fieldNumber: BLF05010; eventRemarks: sifted litter (leaf mold, rotten wood); **Record Level:** institutionCode: CASC
- **Type status:** Other material. **Occurrence:** catalogNumber: casent0484557; recordedBy: Fisher-Griswold Arthropod Team; sex: 1w; preparations: pin; associatedMedia: http://www.antweb.org/specimen/casent0484557; **Taxon:** scientificName: Stigmatomma roahady; genus: Stigmatomma; **Location:** country: Madagascar; stateProvince: Toliara; locality: Parc National d'Andohahela, Col du Sedro, 3.8 km 113° ESE Mahamavo, 37.6 km 341° NNW Tolagnaro; verbatimElevation: 900; decimalLatitude: -24.76389; decimalLongitude: 46.75167; georeferenceRemarks: coordinates obtained from GPS; **Event:** samplingProtocol: MW 50 sample transect, 5m; eventDate: 01/21/2002; habitat: montane rainforest; fieldNumber: BLF05010; eventRemarks: sifted litter (leaf mold, rotten wood); **Record Level:** institutionCode: CASC
- **Type status:** Other material. **Occurrence:** catalogNumber: casent0746581; recordedBy: B.L.Fisher (Sylvain); sex: 1w; preparations: pin; associatedMedia: http://www.antweb.org/specimen/casent0746581; **Taxon:** scientificName: Stigmatomma roahady; genus: Stigmatomma; **Location:** country: Madagascar; stateProvince: Fianarantsoa; locality: R.S. Ivohibe, 7.5 km ENE Ivohibe; verbatimElevation: 900; decimalLatitude: -22.47; decimalLongitude: 46.96; **Event:** samplingProtocol: MW 50 sample transect, 5m; eventDate: 10/07/1997; habitat: rainforest; fieldNumber: BLF01745; eventRemarks: sifted litter (leaf mold, rotten wood); **Record Level:** institutionCode: CASC
- **Type status:** Other material. **Occurrence:** catalogNumber: casent0746584; recordedBy: B.L.Fisher (Sylvain); sex: 1w; preparations: pin; associatedMedia: http://www.antweb.org/specimen/casent0746584; **Taxon:** scientificName: Stigmatomma roahady; genus: Stigmatomma; **Location:** country: Madagascar; stateProvince: Fianarantsoa; locality: R.S. Ivohibe, 7.5 km ENE Ivohibe; verbatimElevation: 900; decimalLatitude: -22.47; decimalLongitude: 46.96; **Event:** samplingProtocol: MW 50 sample transect, 5m; eventDate: 10/07/1997; habitat: rainforest; fieldNumber: BLF01745; eventRemarks: sifted litter (leaf mold, rotten wood); **Record Level:** institutionCode: CASC
- **Type status:** Other material. **Occurrence:** catalogNumber: casent0746586; recordedBy: B.L.Fisher (Sylvain); sex: 1w; preparations: pin; associatedMedia: http://www.antweb.org/specimen/casent0746586; **Taxon:** scientificName: Stigmatomma roahady; genus: Stigmatomma; **Location:** country: Madagascar; stateProvince: Fianarantsoa; locality: R.S. Ivohibe, 7.5 km ENE Ivohibe; verbatimElevation: 900; decimalLatitude: -22.47; decimalLongitude: 46.96; **Event:** samplingProtocol: MW 50 sample transect, 5m; eventDate: 10/07/1997; habitat: rainforest; fieldNumber: BLF01745; eventRemarks: sifted litter (leaf mold, rotten wood); **Record Level:** institutionCode: CASC
- **Type status:** Other material. **Occurrence:** catalogNumber: casent0746589; recordedBy: B.L.Fisher (Sylvain); sex: 1w; preparations: pin; associatedMedia: http://www.antweb.org/specimen/casent0746589; **Taxon:** scientificName: Stigmatomma roahady; genus: Stigmatomma; **Location:** country: Madagascar; stateProvince: Fianarantsoa; locality: R.S. Ivohibe, 7.5 km ENE Ivohibe; verbatimElevation: 900; decimalLatitude: -22.47; decimalLongitude: 46.96; **Event:** samplingProtocol: MW 50 sample transect, 5m; eventDate: 10/07/1997; habitat: rainforest; fieldNumber: BLF01745; eventRemarks: sifted litter (leaf mold, rotten wood); **Record Level:** institutionCode: CASC
- **Type status:** Other material. **Occurrence:** catalogNumber: casent0746590; recordedBy: B.L.Fisher (Sylvain); sex: 1w; preparations: pin; associatedMedia: http://www.antweb.org/specimen/casent0746590; **Taxon:** scientificName: Stigmatomma roahady; genus: Stigmatomma; **Location:** country: Madagascar; stateProvince: Fianarantsoa; locality: R.S. Ivohibe, 7.5 km ENE Ivohibe; verbatimElevation: 900; decimalLatitude: -22.47; decimalLongitude: 46.96; **Event:** samplingProtocol: MW 50 sample transect, 5m; eventDate: 10/07/1997; habitat: rainforest; fieldNumber: BLF01745; eventRemarks: sifted litter (leaf mold, rotten wood); **Record Level:** institutionCode: CASC
- **Type status:** Other material. **Occurrence:** catalogNumber: casent0410401; recordedBy: Fisher, Griswold et al.; sex: 1w; preparations: pin; associatedMedia: http://www.antweb.org/specimen/casent0410401; **Taxon:** scientificName: Stigmatomma roahady; genus: Stigmatomma; **Location:** country: Madagascar; stateProvince: Antsiranana; locality: Parc National Montagne d'Ambre, 3.6 km 235° SW Joffreville; verbatimElevation: 925; decimalLatitude: -12.53444; decimalLongitude: 49.1795; georeferenceRemarks: coordinates obtained from GPS; **Event:** samplingProtocol: General collecting; eventDate: 01/20/2001; habitat: montane rainforest; fieldNumber: BLF02578; eventRemarks: ex rotten log; **Record Level:** institutionCode: CASC
- **Type status:** Other material. **Occurrence:** catalogNumber: casent0139423; recordedBy: B.L.Fisher et al.; sex: 1w; preparations: pin; associatedMedia: http://www.antweb.org/specimen/casent0139423; **Taxon:** scientificName: Stigmatomma roahady; genus: Stigmatomma; **Location:** country: Madagascar; stateProvince: Toamasina; locality: Station forestière Analamazaotra, Analamazaotra 1.3km S Andasibe; verbatimElevation: 980; decimalLatitude: -18.38466; decimalLongitude: 48.41271; georeferenceRemarks: coordinates obtained from GPS; **Event:** samplingProtocol: 9 MaxiWinks, mixed samples; eventDate: 12/11/2007; habitat: montane rainforest; fieldNumber: BLF19282; eventRemarks: sifted litter (leaf mold, rotten wood); **Record Level:** institutionCode: CASC
- **Type status:** Other material. **Occurrence:** catalogNumber: casent0139424; recordedBy: B.L.Fisher et al.; sex: 1w; preparations: pin; associatedMedia: http://www.antweb.org/specimen/casent0139424; **Taxon:** scientificName: Stigmatomma roahady; genus: Stigmatomma; **Location:** country: Madagascar; stateProvince: Toamasina; locality: Station forestière Analamazaotra, Analamazaotra 1.3km S Andasibe; verbatimElevation: 980; decimalLatitude: -18.38466; decimalLongitude: 48.41271; georeferenceRemarks: coordinates obtained from GPS; **Event:** samplingProtocol: 9 MaxiWinks, mixed samples; eventDate: 12/11/2007; habitat: montane rainforest; fieldNumber: BLF19282; eventRemarks: sifted litter (leaf mold, rotten wood); **Record Level:** institutionCode: CASC
- **Type status:** Other material. **Occurrence:** catalogNumber: casent0139425; recordedBy: B.L.Fisher et al.; sex: 1w; preparations: pin; associatedMedia: http://www.antweb.org/specimen/casent0139425; **Taxon:** scientificName: Stigmatomma roahady; genus: Stigmatomma; **Location:** country: Madagascar; stateProvince: Toamasina; locality: Station forestière Analamazaotra, Analamazaotra 1.3km S Andasibe; verbatimElevation: 980; decimalLatitude: -18.38466; decimalLongitude: 48.41271; georeferenceRemarks: coordinates obtained from GPS; **Event:** samplingProtocol: 9 MaxiWinks, mixed samples; eventDate: 12/11/2007; habitat: montane rainforest; fieldNumber: BLF19282; eventRemarks: sifted litter (leaf mold, rotten wood); **Record Level:** institutionCode: CASC
- **Type status:** Other material. **Occurrence:** catalogNumber: casent0139426; recordedBy: B.L.Fisher et al.; sex: 1w; preparations: pin; associatedMedia: http://www.antweb.org/specimen/casent0139426; **Taxon:** scientificName: Stigmatomma roahady; genus: Stigmatomma; **Location:** country: Madagascar; stateProvince: Toamasina; locality: Station forestière Analamazaotra, Analamazaotra 1.3km S Andasibe; verbatimElevation: 980; decimalLatitude: -18.38466; decimalLongitude: 48.41271; georeferenceRemarks: coordinates obtained from GPS; **Event:** samplingProtocol: 9 MaxiWinks, mixed samples; eventDate: 12/11/2007; habitat: montane rainforest; fieldNumber: BLF19282; eventRemarks: sifted litter (leaf mold, rotten wood); **Record Level:** institutionCode: CASC
- **Type status:** Other material. **Occurrence:** catalogNumber: casent0139427; recordedBy: B.L.Fisher et al.; sex: 1w; preparations: pin; associatedMedia: http://www.antweb.org/specimen/casent0139427; **Taxon:** scientificName: Stigmatomma roahady; genus: Stigmatomma; **Location:** country: Madagascar; stateProvince: Toamasina; locality: Station forestière Analamazaotra, Analamazaotra 1.3km S Andasibe; verbatimElevation: 980; decimalLatitude: -18.38466; decimalLongitude: 48.41271; georeferenceRemarks: coordinates obtained from GPS; **Event:** samplingProtocol: 9 MaxiWinks, mixed samples; eventDate: 12/11/2007; habitat: montane rainforest; fieldNumber: BLF19282; eventRemarks: sifted litter (leaf mold, rotten wood); **Record Level:** institutionCode: CASC
- **Type status:** Other material. **Occurrence:** catalogNumber: casent0139428; recordedBy: B.L.Fisher et al.; sex: 1w; preparations: pin; associatedMedia: http://www.antweb.org/specimen/casent0139428; **Taxon:** scientificName: Stigmatomma roahady; genus: Stigmatomma; **Location:** country: Madagascar; stateProvince: Toamasina; locality: Station forestière Analamazaotra, Analamazaotra 1.3km S Andasibe; verbatimElevation: 980; decimalLatitude: -18.38466; decimalLongitude: 48.41271; georeferenceRemarks: coordinates obtained from GPS; **Event:** samplingProtocol: 9 MaxiWinks, mixed samples; eventDate: 12/11/2007; habitat: montane rainforest; fieldNumber: BLF19282; eventRemarks: sifted litter (leaf mold, rotten wood); **Record Level:** institutionCode: CASC
- **Type status:** Other material. **Occurrence:** catalogNumber: casent0139429; recordedBy: B.L.Fisher et al.; sex: 1w; preparations: pin; associatedMedia: http://www.antweb.org/specimen/casent0139429; **Taxon:** scientificName: Stigmatomma roahady; genus: Stigmatomma; **Location:** country: Madagascar; stateProvince: Toamasina; locality: Station forestière Analamazaotra, Analamazaotra 1.3km S Andasibe; verbatimElevation: 980; decimalLatitude: -18.38466; decimalLongitude: 48.41271; georeferenceRemarks: coordinates obtained from GPS; **Event:** samplingProtocol: 9 MaxiWinks, mixed samples; eventDate: 12/11/2007; habitat: montane rainforest; fieldNumber: BLF19282; eventRemarks: sifted litter (leaf mold, rotten wood); **Record Level:** institutionCode: CASC
- **Type status:** Other material. **Occurrence:** catalogNumber: casent0139430; recordedBy: B.L.Fisher et al.; sex: 1w; preparations: pin; associatedMedia: http://www.antweb.org/specimen/casent0139430; **Taxon:** scientificName: Stigmatomma roahady; genus: Stigmatomma; **Location:** country: Madagascar; stateProvince: Toamasina; locality: Station forestière Analamazaotra, Analamazaotra 1.3km S Andasibe; verbatimElevation: 980; decimalLatitude: -18.38466; decimalLongitude: 48.41271; georeferenceRemarks: coordinates obtained from GPS; **Event:** samplingProtocol: 9 MaxiWinks, mixed samples; eventDate: 12/11/2007; habitat: montane rainforest; fieldNumber: BLF19282; eventRemarks: sifted litter (leaf mold, rotten wood); **Record Level:** institutionCode: CASC
- **Type status:** Other material. **Occurrence:** catalogNumber: casent0134766; recordedBy: B.L.Fisher et al.; sex: 1w; preparations: pin; associatedMedia: http://www.antweb.org/specimen/casent0134766; **Taxon:** scientificName: Stigmatomma roahady; genus: Stigmatomma; **Location:** country: Madagascar; stateProvince: Toamasina; locality: Bevolota 17.1km N Andasibe; verbatimElevation: 995; decimalLatitude: -18.77071; decimalLongitude: 48.43164; georeferenceRemarks: coordinates obtained from GPS; **Event:** samplingProtocol: General collecting; eventDate: 12/12/2007; habitat: montane rainforest; fieldNumber: BLF19308; eventRemarks: ex rotten log; **Record Level:** institutionCode: CASC
- **Type status:** Other material. **Occurrence:** catalogNumber: casent0134768; recordedBy: B.L.Fisher et al.; sex: 1w; preparations: pin; associatedMedia: http://www.antweb.org/specimen/casent0134768; **Taxon:** scientificName: Stigmatomma roahady; genus: Stigmatomma; **Location:** country: Madagascar; stateProvince: Toamasina; locality: Bevolota 17.1km N Andasibe; verbatimElevation: 995; decimalLatitude: -18.77071; decimalLongitude: 48.43164; georeferenceRemarks: coordinates obtained from GPS; **Event:** samplingProtocol: General collecting; eventDate: 12/12/2007; habitat: montane rainforest; fieldNumber: BLF19306; eventRemarks: under moss, above ground, dead tree; **Record Level:** institutionCode: CASC
- **Type status:** Other material. **Occurrence:** catalogNumber: casent0135098; recordedBy: B.L.Fisher et al.; sex: 1w; preparations: pin; associatedMedia: http://www.antweb.org/specimen/casent0135098; **Taxon:** scientificName: Stigmatomma roahady; genus: Stigmatomma; **Location:** country: Madagascar; stateProvince: Toamasina; locality: Bevolota 17.1km N Andasibe; verbatimElevation: 995; decimalLatitude: -18.77071; decimalLongitude: 48.43164; georeferenceRemarks: coordinates obtained from GPS; **Event:** samplingProtocol: General collecting; eventDate: 12/12/2007; habitat: montane rainforest; fieldNumber: BLF19379; eventRemarks: ex rotten log; **Record Level:** institutionCode: CASC
- **Type status:** Other material. **Occurrence:** catalogNumber: casent0275083; recordedBy: B.L.Fisher et al.; sex: 1w; preparations: pin; associatedMedia: http://www.antweb.org/specimen/casent0275083; **Taxon:** scientificName: Stigmatomma roahady; genus: Stigmatomma; **Location:** country: Madagascar; stateProvince: Toamasina; locality: Torotorofotsy; verbatimElevation: 1005; decimalLatitude: -18.77048; decimalLongitude: 48.43043; georeferenceRemarks: ±200 m, coordinates obtained from GPS; **Event:** samplingProtocol: General collecting; eventDate: 03/12/2012; habitat: montane rainforest; fieldNumber: BLF28421; eventRemarks: ex rotten log; **Record Level:** institutionCode: CASC
- **Type status:** Other material. **Occurrence:** catalogNumber: casent0275085; recordedBy: B.L.Fisher et al.; sex: 1w; preparations: pin; associatedMedia: http://www.antweb.org/specimen/casent0275085; **Taxon:** scientificName: Stigmatomma roahady; genus: Stigmatomma; **Location:** country: Madagascar; stateProvince: Toamasina; locality: Torotorofotsy; verbatimElevation: 1005; decimalLatitude: -18.77048; decimalLongitude: 48.43043; georeferenceRemarks: ±200 m, coordinates obtained from GPS; **Event:** samplingProtocol: General collecting; eventDate: 03/12/2012; habitat: montane rainforest; fieldNumber: BLF28441; eventRemarks: ex root mat, ground layer; **Record Level:** institutionCode: CASC
- **Type status:** Other material. **Occurrence:** catalogNumber: casent0275088; recordedBy: B.L.Fisher et al.; sex: 1w; preparations: pin; associatedMedia: http://www.antweb.org/specimen/casent0275088; **Taxon:** scientificName: Stigmatomma roahady; genus: Stigmatomma; **Location:** country: Madagascar; stateProvince: Toamasina; locality: Torotorofotsy; verbatimElevation: 1005; decimalLatitude: -18.77048; decimalLongitude: 48.43043; georeferenceRemarks: ±200 m, coordinates obtained from GPS; **Event:** samplingProtocol: General collecting; eventDate: 03/12/2012; habitat: montane rainforest; fieldNumber: BLF28436; eventRemarks: ex rotten log; **Record Level:** institutionCode: CASC
- **Type status:** Other material. **Occurrence:** catalogNumber: casent0275089; recordedBy: B.L.Fisher et al.; sex: 1w; preparations: pin; associatedMedia: http://www.antweb.org/specimen/casent0275089; **Taxon:** scientificName: Stigmatomma roahady; genus: Stigmatomma; **Location:** country: Madagascar; stateProvince: Toamasina; locality: Torotorofotsy; verbatimElevation: 1005; decimalLatitude: -18.77048; decimalLongitude: 48.43043; georeferenceRemarks: ±200 m, coordinates obtained from GPS; **Event:** samplingProtocol: General collecting; eventDate: 03/12/2012; habitat: montane rainforest; fieldNumber: BLF28436; eventRemarks: ex rotten log; **Record Level:** institutionCode: CASC
- **Type status:** Other material. **Occurrence:** catalogNumber: casent0275091; recordedBy: B.L.Fisher et al.; sex: 1w; preparations: pin; associatedMedia: http://www.antweb.org/specimen/casent0275091; **Taxon:** scientificName: Stigmatomma roahady; genus: Stigmatomma; **Location:** country: Madagascar; stateProvince: Toamasina; locality: Torotorofotsy; verbatimElevation: 1005; decimalLatitude: -18.77048; decimalLongitude: 48.43043; georeferenceRemarks: ±200 m, coordinates obtained from GPS; **Event:** samplingProtocol: General collecting; eventDate: 03/12/2012; habitat: montane rainforest; fieldNumber: BLF28431; eventRemarks: ex rotten log; **Record Level:** institutionCode: CASC
- **Type status:** Other material. **Occurrence:** catalogNumber: casent0275093; recordedBy: B.L.Fisher et al.; sex: 1w; preparations: pin; associatedMedia: http://www.antweb.org/specimen/casent0275093; **Taxon:** scientificName: Stigmatomma roahady; genus: Stigmatomma; **Location:** country: Madagascar; stateProvince: Toamasina; locality: Torotorofotsy; verbatimElevation: 1005; decimalLatitude: -18.77048; decimalLongitude: 48.43043; georeferenceRemarks: ±200 m, coordinates obtained from GPS; **Event:** samplingProtocol: General collecting; eventDate: 03/12/2012; habitat: montane rainforest; fieldNumber: BLF28440; eventRemarks: ex rotten log; **Record Level:** institutionCode: CASC
- **Type status:** Other material. **Occurrence:** catalogNumber: casent0318450; recordedBy: B.L.Fisher et al.; sex: 1m; preparations: pin; associatedMedia: http://www.antweb.org/specimen/casent0318450; **Taxon:** scientificName: Stigmatomma roahady; genus: Stigmatomma; **Location:** country: Madagascar; stateProvince: Toamasina; locality: Torotorofotsy; verbatimElevation: 1006; decimalLatitude: -18.77088; decimalLongitude: 48.43194; georeferenceRemarks: ±200 m, coordinates obtained from GPS; **Event:** samplingProtocol: General collecting; eventDate: 12/12/2014; habitat: montane rainforest; fieldNumber: BLF35430; eventRemarks: ex soil; **Record Level:** institutionCode: CASC
- **Type status:** Other material. **Occurrence:** catalogNumber: casent0701221; recordedBy: B.L.Fisher et al.; sex: 1w; preparations: pin; associatedMedia: http://www.antweb.org/specimen/casent0701221; **Taxon:** scientificName: Stigmatomma roahady; genus: Stigmatomma; **Location:** country: Madagascar; stateProvince: Toamasina; locality: Torotorofotsy; verbatimElevation: 1006; decimalLatitude: -18.77088; decimalLongitude: 48.43194; georeferenceRemarks: ±200 m, coordinates obtained from GPS; **Event:** samplingProtocol: General collecting; eventDate: 12/12/2014; habitat: montane rainforest; fieldNumber: BLF35445; eventRemarks: ex soil; **Record Level:** institutionCode: CASC
- **Type status:** Other material. **Occurrence:** catalogNumber: casent0701534; recordedBy: B.L.Fisher et al.; sex: 1w; preparations: pin; associatedMedia: http://www.antweb.org/specimen/casent0701534; **Taxon:** scientificName: Stigmatomma roahady; genus: Stigmatomma; **Location:** country: Madagascar; stateProvince: Toamasina; locality: Torotorofotsy; verbatimElevation: 1006; decimalLatitude: -18.77088; decimalLongitude: 48.43194; georeferenceRemarks: ±200 m, coordinates obtained from GPS; **Event:** samplingProtocol: General collecting; eventDate: 12/12/2014; habitat: montane rainforest; fieldNumber: BLF35430; eventRemarks: ex soil; **Record Level:** institutionCode: CASC
- **Type status:** Other material. **Occurrence:** catalogNumber: casent0701535; recordedBy: B.L.Fisher et al.; sex: 1w; preparations: pin; associatedMedia: http://www.antweb.org/specimen/casent0701535; **Taxon:** scientificName: Stigmatomma roahady; genus: Stigmatomma; **Location:** country: Madagascar; stateProvince: Toamasina; locality: Torotorofotsy; verbatimElevation: 1006; decimalLatitude: -18.77088; decimalLongitude: 48.43194; georeferenceRemarks: ±200 m, coordinates obtained from GPS; **Event:** samplingProtocol: General collecting; eventDate: 12/12/2014; habitat: montane rainforest; fieldNumber: BLF35430; eventRemarks: ex soil; **Record Level:** institutionCode: CASC
- **Type status:** Other material. **Occurrence:** catalogNumber: casent0701536; recordedBy: B.L.Fisher et al.; sex: 1w; preparations: pin; associatedMedia: http://www.antweb.org/specimen/casent0701536; **Taxon:** scientificName: Stigmatomma roahady; genus: Stigmatomma; **Location:** country: Madagascar; stateProvince: Toamasina; locality: Torotorofotsy; verbatimElevation: 1006; decimalLatitude: -18.77088; decimalLongitude: 48.43194; georeferenceRemarks: ±200 m, coordinates obtained from GPS; **Event:** samplingProtocol: General collecting; eventDate: 12/12/2014; habitat: montane rainforest; fieldNumber: BLF35430; eventRemarks: ex soil; **Record Level:** institutionCode: CASC
- **Type status:** Other material. **Occurrence:** catalogNumber: casent0701537; recordedBy: B.L.Fisher et al.; sex: 1w; preparations: pin; associatedMedia: http://www.antweb.org/specimen/casent0701537; **Taxon:** scientificName: Stigmatomma roahady; genus: Stigmatomma; **Location:** country: Madagascar; stateProvince: Toamasina; locality: Torotorofotsy; verbatimElevation: 1006; decimalLatitude: -18.77088; decimalLongitude: 48.43194; georeferenceRemarks: ±200 m, coordinates obtained from GPS; **Event:** samplingProtocol: General collecting; eventDate: 12/12/2014; habitat: montane rainforest; fieldNumber: BLF35430; eventRemarks: ex soil; **Record Level:** institutionCode: CASC
- **Type status:** Other material. **Occurrence:** catalogNumber: casent0701538; recordedBy: B.L.Fisher et al.; sex: 1w; preparations: pin; associatedMedia: http://www.antweb.org/specimen/casent0701538; **Taxon:** scientificName: Stigmatomma roahady; genus: Stigmatomma; **Location:** country: Madagascar; stateProvince: Toamasina; locality: Torotorofotsy; verbatimElevation: 1006; decimalLatitude: -18.77088; decimalLongitude: 48.43194; georeferenceRemarks: ±200 m, coordinates obtained from GPS; **Event:** samplingProtocol: General collecting; eventDate: 12/12/2014; habitat: montane rainforest; fieldNumber: BLF35430; eventRemarks: ex soil; **Record Level:** institutionCode: CASC
- **Type status:** Other material. **Occurrence:** catalogNumber: casent0701539; recordedBy: B.L.Fisher et al.; sex: 1w; preparations: pin; associatedMedia: http://www.antweb.org/specimen/casent0701539; **Taxon:** scientificName: Stigmatomma roahady; genus: Stigmatomma; **Location:** country: Madagascar; stateProvince: Toamasina; locality: Torotorofotsy; verbatimElevation: 1006; decimalLatitude: -18.77088; decimalLongitude: 48.43194; georeferenceRemarks: ±200 m, coordinates obtained from GPS; **Event:** samplingProtocol: General collecting; eventDate: 12/12/2014; habitat: montane rainforest; fieldNumber: BLF35430; eventRemarks: ex soil; **Record Level:** institutionCode: CASC
- **Type status:** Other material. **Occurrence:** catalogNumber: casent0300365; recordedBy: B.L.Fisher et al.; sex: 1w; preparations: pin; associatedMedia: http://www.antweb.org/specimen/casent0300365; **Taxon:** scientificName: Stigmatomma roahady; genus: Stigmatomma; **Location:** country: Madagascar; stateProvince: Toamasina; locality: Corridor Forestier Analamay-Mantadia, Ambatoharanana; verbatimElevation: 1016; decimalLatitude: -18.79944; decimalLongitude: 48.40375; georeferenceRemarks: ±100 m; **Event:** samplingProtocol: General collecting; eventDate: 12/12/2012; habitat: rainforest; fieldNumber: BLF30485; eventRemarks: under rotten log; **Record Level:** institutionCode: CASC
- **Type status:** Other material. **Occurrence:** catalogNumber: casent0300378; recordedBy: B.L.Fisher et al.; sex: 1w; preparations: pin; associatedMedia: http://www.antweb.org/specimen/casent0300378; **Taxon:** scientificName: Stigmatomma roahady; genus: Stigmatomma; **Location:** country: Madagascar; stateProvince: Toamasina; locality: Corridor Forestier Analamay-Mantadia, Ambatoharanana; verbatimElevation: 1016; decimalLatitude: -18.79944; decimalLongitude: 48.40375; georeferenceRemarks: ±100 m; **Event:** samplingProtocol: General collecting; eventDate: 12/12/2012; habitat: rainforest; fieldNumber: BLF30486; eventRemarks: under rotten log; **Record Level:** institutionCode: CASC
- **Type status:** Other material. **Occurrence:** catalogNumber: casent0007136; recordedBy: R. Harin'Hala; sex: 1aQ; preparations: pin; associatedMedia: http://www.antweb.org/specimen/casent0007136; **Taxon:** scientificName: Stigmatomma roahady; genus: Stigmatomma; **Location:** country: Madagascar; stateProvince: Fianarantsoa; locality: Belle Vue trail, Ranomafana National Park, Fianarantsoa Prov.; verbatimElevation: 1020; decimalLatitude: -21.2665; decimalLongitude: 47.42017; **Event:** samplingProtocol: Malaise trap; eventDate: 02/26/2002; habitat: mixed tropical forest; fieldNumber: MA-02-09C-18; **Record Level:** institutionCode: CASC
- **Type status:** Other material. **Occurrence:** catalogNumber: casent0300576; recordedBy: B.L.Fisher et al.; sex: 1w; preparations: pin; associatedMedia: http://www.antweb.org/specimen/casent0300576; **Taxon:** scientificName: Stigmatomma roahady; genus: Stigmatomma; **Location:** country: Madagascar; stateProvince: Toamasina; locality: Corridor Forestier Analamay-Mantadia, Tsaravoniana; verbatimElevation: 1039; decimalLatitude: -18.76465; decimalLongitude: 48.41938; georeferenceRemarks: ±100 m; **Event:** samplingProtocol: 3 MaxiWinks, mixed samples; eventDate: 12/02/2012; habitat: rainforest; fieldNumber: BLF30065; eventRemarks: sifted litter; **Record Level:** institutionCode: CASC
- **Type status:** Other material. **Occurrence:** catalogNumber: casent0300577; recordedBy: B.L.Fisher et al.; sex: 1w; preparations: pin; associatedMedia: http://www.antweb.org/specimen/casent0300577; **Taxon:** scientificName: Stigmatomma roahady; genus: Stigmatomma; **Location:** country: Madagascar; stateProvince: Toamasina; locality: Corridor Forestier Analamay-Mantadia, Tsaravoniana; verbatimElevation: 1039; decimalLatitude: -18.76465; decimalLongitude: 48.41938; georeferenceRemarks: ±100 m; **Event:** samplingProtocol: 3 MaxiWinks, mixed samples; eventDate: 12/02/2012; habitat: rainforest; fieldNumber: BLF30065; eventRemarks: sifted litter; **Record Level:** institutionCode: CASC
- **Type status:** Other material. **Occurrence:** catalogNumber: casent0300785; recordedBy: B.L.Fisher et al.; sex: 1w; preparations: pin; associatedMedia: http://www.antweb.org/specimen/casent0300785; **Taxon:** scientificName: Stigmatomma roahady; genus: Stigmatomma; **Location:** country: Madagascar; stateProvince: Toamasina; locality: Corridor Forestier Analamay-Mantadia, Tsaravoniana; verbatimElevation: 1039; decimalLatitude: -18.76465; decimalLongitude: 48.41938; georeferenceRemarks: ±100 m; **Event:** samplingProtocol: General collecting; eventDate: 12/04/2012; habitat: rainforest; fieldNumber: BLF30105; eventRemarks: ex rotten log; **Record Level:** institutionCode: CASC
- **Type status:** Other material. **Occurrence:** catalogNumber: casent0279142; recordedBy: B.L.Fisher et al.; sex: 1w; preparations: pin; associatedMedia: http://www.antweb.org/specimen/casent0279142; **Taxon:** scientificName: Stigmatomma roahady; genus: Stigmatomma; **Location:** country: Madagascar; stateProvince: Toamasina; locality: Corridor Forestier Analamay-Mantadia, Ambohibolakely; verbatimElevation: 1044; decimalLatitude: -18.76087; decimalLongitude: 48.37128; georeferenceRemarks: ±100 m; **Event:** samplingProtocol: General collecting; eventDate: 11/29/2012; habitat: rainforest; fieldNumber: BLF29923; eventRemarks: ex rotten log; **Record Level:** institutionCode: CASC
- **Type status:** Other material. **Occurrence:** catalogNumber: casent0279143; recordedBy: B.L.Fisher et al.; sex: 1w; preparations: pin; associatedMedia: http://www.antweb.org/specimen/casent0279143; **Taxon:** scientificName: Stigmatomma roahady; genus: Stigmatomma; **Location:** country: Madagascar; stateProvince: Toamasina; locality: Corridor Forestier Analamay-Mantadia, Ambohibolakely; verbatimElevation: 1044; decimalLatitude: -18.76087; decimalLongitude: 48.37128; georeferenceRemarks: ±100 m; **Event:** samplingProtocol: General collecting; eventDate: 11/29/2012; habitat: rainforest; fieldNumber: BLF29923; eventRemarks: ex rotten log; **Record Level:** institutionCode: CASC
- **Type status:** Other material. **Occurrence:** catalogNumber: casent0300290; recordedBy: B.L.Fisher et al.; sex: 1w; preparations: pin; associatedMedia: http://www.antweb.org/specimen/casent0300290; **Taxon:** scientificName: Stigmatomma roahady; genus: Stigmatomma; **Location:** country: Madagascar; stateProvince: Toamasina; locality: Corridor Forestier Analamay-Mantadia, Ambohibolakely; verbatimElevation: 1044; decimalLatitude: -18.76087; decimalLongitude: 48.37128; georeferenceRemarks: ±100 m; **Event:** samplingProtocol: General collecting; eventDate: 11/29/2012; habitat: rainforest; fieldNumber: BLF29932; eventRemarks: ex soil; **Record Level:** institutionCode: CASC
- **Type status:** Other material. **Occurrence:** catalogNumber: casent0279147; recordedBy: B.L.Fisher et al.; sex: 1w; preparations: pin; associatedMedia: http://www.antweb.org/specimen/casent0279147; **Taxon:** scientificName: Stigmatomma roahady; genus: Stigmatomma; **Location:** country: Madagascar; stateProvince: Toamasina; locality: Corridor Forestier Analamay-Mantadia, Ambohibolakely; verbatimElevation: 1058; decimalLatitude: -18.79956; decimalLongitude: 48.4028; georeferenceRemarks: ±100 m; **Event:** samplingProtocol: General collecting; eventDate: 12/12/2012; habitat: rainforest; fieldNumber: BLF30450; eventRemarks: ex soil; **Record Level:** institutionCode: CASC
- **Type status:** Other material. **Occurrence:** catalogNumber: casent0300443; recordedBy: B.L.Fisher et al.; sex: 1w; preparations: pin; associatedMedia: http://www.antweb.org/specimen/casent0300443; **Taxon:** scientificName: Stigmatomma roahady; genus: Stigmatomma; **Location:** country: Madagascar; stateProvince: Toamasina; locality: Corridor Forestier Analamay-Mantadia, Ambatoharanana; verbatimElevation: 1058; decimalLatitude: -18.79956; decimalLongitude: 48.4028; georeferenceRemarks: ±100 m; **Event:** samplingProtocol: General collecting; eventDate: 12/12/2012; habitat: rainforest; fieldNumber: BLF30450; eventRemarks: ex soil; **Record Level:** institutionCode: CASC
- **Type status:** Other material. **Occurrence:** catalogNumber: casent0300447; recordedBy: B.L.Fisher et al.; sex: 1w; preparations: pin; associatedMedia: http://www.antweb.org/specimen/casent0300447; **Taxon:** scientificName: Stigmatomma roahady; genus: Stigmatomma; **Location:** country: Madagascar; stateProvince: Toamasina; locality: Corridor Forestier Analamay-Mantadia, Ambatoharanana; verbatimElevation: 1058; decimalLatitude: -18.79956; decimalLongitude: 48.4028; georeferenceRemarks: ±100 m; **Event:** samplingProtocol: General collecting; eventDate: 12/12/2012; habitat: rainforest; fieldNumber: BLF30430; eventRemarks: ex soil; **Record Level:** institutionCode: CASC
- **Type status:** Other material. **Occurrence:** catalogNumber: casent0300409; recordedBy: B.L.Fisher et al.; sex: 1w; preparations: pin; associatedMedia: http://www.antweb.org/specimen/casent0300409; **Taxon:** scientificName: Stigmatomma roahady; genus: Stigmatomma; **Location:** country: Madagascar; stateProvince: Toamasina; locality: Corridor Forestier Analamay-Mantadia, Ambatoharanana; verbatimElevation: 1064; decimalLatitude: -18.80398; decimalLongitude: 48.40358; georeferenceRemarks: ±100 m; **Event:** samplingProtocol: General collecting; eventDate: 12/12/2012; habitat: rainforest; fieldNumber: BLF30410; eventRemarks: ex rotten log; **Record Level:** institutionCode: CASC
- **Type status:** Other material. **Occurrence:** catalogNumber: casent0302134; recordedBy: B.L.Fisher et al.; sex: 1w; preparations: pin; associatedMedia: http://www.antweb.org/specimen/casent0302134; **Taxon:** scientificName: Stigmatomma roahady; genus: Stigmatomma; **Location:** country: Madagascar; stateProvince: Toamasina; locality: Corridor Forestier Analamay-Mantadia, Ambatoharanana; verbatimElevation: 1064; decimalLatitude: -18.80398; decimalLongitude: 48.40358; georeferenceRemarks: ±100 m; **Event:** samplingProtocol: 03 MaxiWinks, mixed samples; eventDate: 12/12/2012; habitat: rainforest; fieldNumber: BLF30259; eventRemarks: sifted litter; **Record Level:** institutionCode: CASC
- **Type status:** Other material. **Occurrence:** catalogNumber: casent0302135; recordedBy: B.L.Fisher et al.; sex: 1w; preparations: pin; associatedMedia: http://www.antweb.org/specimen/casent0302135; **Taxon:** scientificName: Stigmatomma roahady; genus: Stigmatomma; **Location:** country: Madagascar; stateProvince: Toamasina; locality: Corridor Forestier Analamay-Mantadia, Ambatoharanana; verbatimElevation: 1064; decimalLatitude: -18.80398; decimalLongitude: 48.40358; georeferenceRemarks: ±100 m; **Event:** samplingProtocol: 03 MaxiWinks, mixed samples; eventDate: 12/12/2012; habitat: rainforest; fieldNumber: BLF30259; eventRemarks: sifted litter; **Record Level:** institutionCode: CASC
- **Type status:** Other material. **Occurrence:** catalogNumber: casent0369790; recordedBy: B.L.Fisher et al.; sex: 1w; preparations: pin; associatedMedia: http://www.antweb.org/specimen/casent0369790; **Taxon:** scientificName: Stigmatomma roahady; genus: Stigmatomma; **Location:** country: Madagascar; stateProvince: Antsiranana; locality: Binara Forest; verbatimElevation: 1065; decimalLatitude: -13.26392; decimalLongitude: 49.59919; georeferenceRemarks: ±500m; **Event:** samplingProtocol: 3 MaxiWinks, mixed samples; eventDate: 10/18/2013; habitat: rainforest; fieldNumber: BLF32140; eventRemarks: sifted litter; **Record Level:** institutionCode: CASC
- **Type status:** Other material. **Occurrence:** catalogNumber: casent0074429; recordedBy: Malagasy ant team; sex: 1w; preparations: pin; associatedMedia: http://www.antweb.org/specimen/casent0074429; **Taxon:** scientificName: Stigmatomma roahady; genus: Stigmatomma; **Location:** country: Madagascar; stateProvince: Toamasina; locality: Analamay; verbatimElevation: 1068; decimalLatitude: -18.80623; decimalLongitude: 48.33707; georeferenceRemarks: coordinates obtained from GPS; **Event:** samplingProtocol: MW, 25 sifted litter; eventDate: 03/21/2004; habitat: montane rainforest; fieldNumber: BLF10502; eventRemarks: sifted litter (leaf mold, rotten wood); **Record Level:** institutionCode: CASC
- **Type status:** Other material. **Occurrence:** catalogNumber: casent0074432; recordedBy: Malagasy ant team; sex: 2w; preparations: pin; associatedMedia: http://www.antweb.org/specimen/casent0074432; **Taxon:** scientificName: Stigmatomma roahady; genus: Stigmatomma; **Location:** country: Madagascar; stateProvince: Toamasina; locality: Analamay; verbatimElevation: 1068; decimalLatitude: -18.80623; decimalLongitude: 48.33707; georeferenceRemarks: coordinates obtained from GPS; **Event:** samplingProtocol: MW, 25 sifted litter; eventDate: 03/21/2004; habitat: montane rainforest; fieldNumber: BLF10502; eventRemarks: sifted litter (leaf mold, rotten wood); **Record Level:** institutionCode: CASC
- **Type status:** Other material. **Occurrence:** catalogNumber: casent0293888; recordedBy: Andrianjaka Ravelomanana; sex: 1w; preparations: pin; associatedMedia: http://www.antweb.org/specimen/casent0293888; **Taxon:** scientificName: Stigmatomma roahady; genus: Stigmatomma; **Location:** country: Madagascar; stateProvince: Fianarantsoa; locality: Ambinanindranomena Non Protected Area, 39.45km SE Ambalavao; verbatimElevation: 1069; decimalLatitude: -21.95386; decimalLongitude: 47.29427; georeferenceRemarks: ±200 m; **Event:** samplingProtocol: PF 20 tube sample transect, 10m; eventDate: 02/01/2012; habitat: montane rainforest; fieldNumber: ARA1271; eventRemarks: pitfall trap; **Record Level:** institutionCode: CASC
- **Type status:** Other material. **Occurrence:** catalogNumber: casent0046989; recordedBy: Malagasy ant team; sex: 1w; preparations: pin; associatedMedia: http://www.antweb.org/specimen/casent0046989; **Taxon:** scientificName: Stigmatomma roahady; genus: Stigmatomma; **Location:** country: Madagascar; stateProvince: Toamasina; locality: Torotorofotsy; verbatimElevation: 1070; decimalLatitude: -18.87082; decimalLongitude: 48.34737; georeferenceRemarks: coordinates obtained from GPS; **Event:** samplingProtocol: MW, 25 sifted litter; eventDate: 03/24/2004; habitat: montane rainforest, marsh edge; fieldNumber: BLF10627; eventRemarks: sifted litter (leaf mold, rotten wood); **Record Level:** institutionCode: CASC
- **Type status:** Other material. **Occurrence:** catalogNumber: casent0046990; recordedBy: Malagasy ant team; sex: 1w; preparations: pin; associatedMedia: http://www.antweb.org/specimen/casent0046990; **Taxon:** scientificName: Stigmatomma roahady; genus: Stigmatomma; **Location:** country: Madagascar; stateProvince: Toamasina; locality: Torotorofotsy; verbatimElevation: 1070; decimalLatitude: -18.87082; decimalLongitude: 48.34737; georeferenceRemarks: coordinates obtained from GPS; **Event:** samplingProtocol: MW, 25 sifted litter; eventDate: 03/24/2004; habitat: montane rainforest, marsh edge; fieldNumber: BLF10627; eventRemarks: sifted litter (leaf mold, rotten wood); **Record Level:** institutionCode: CASC
- **Type status:** Other material. **Occurrence:** catalogNumber: casent0046991; recordedBy: Malagasy ant team; sex: 1w; preparations: pin; associatedMedia: http://www.antweb.org/specimen/casent0046991; **Taxon:** scientificName: Stigmatomma roahady; genus: Stigmatomma; **Location:** country: Madagascar; stateProvince: Toamasina; locality: Torotorofotsy; verbatimElevation: 1070; decimalLatitude: -18.87082; decimalLongitude: 48.34737; georeferenceRemarks: coordinates obtained from GPS; **Event:** samplingProtocol: MW, 25 sifted litter; eventDate: 03/24/2004; habitat: montane rainforest, marsh edge; fieldNumber: BLF10627; eventRemarks: sifted litter (leaf mold, rotten wood); **Record Level:** institutionCode: CASC
- **Type status:** Other material. **Occurrence:** catalogNumber: casent0053729; recordedBy: Malagasy ant team; sex: 1w (missing gaster); preparations: pin; associatedMedia: http://www.antweb.org/specimen/casent0053729; **Taxon:** scientificName: Stigmatomma roahady; genus: Stigmatomma; **Location:** country: Madagascar; stateProvince: Toamasina; locality: Torotorofotsy; verbatimElevation: 1070; decimalLatitude: -18.87082; decimalLongitude: 48.34737; georeferenceRemarks: coordinates obtained from GPS; **Event:** samplingProtocol: MW, 25 sifted litter; eventDate: 03/24/2004; habitat: montane rainforest, marsh edge; fieldNumber: BLF10627; eventRemarks: sifted litter (leaf mold, rotten wood); **Record Level:** institutionCode: CASC
- **Type status:** Other material. **Occurrence:** catalogNumber: casent0050355; recordedBy: Malagasy ant team; sex: 3w; preparations: pin; associatedMedia: http://www.antweb.org/specimen/casent0050355; **Taxon:** scientificName: Stigmatomma roahady; genus: Stigmatomma; **Location:** country: Madagascar; stateProvince: Toamasina; locality: Forêt Ambatovy, 14.3 km 57° Moramanga; verbatimElevation: 1075; decimalLatitude: -18.85083; decimalLongitude: 48.32; georeferenceRemarks: coordinates obtained from GPS; **Event:** samplingProtocol: General collecting; eventDate: 03/22/2004; habitat: montane rainforest; fieldNumber: BLF10546; eventRemarks: ex root mat, ground layer; **Record Level:** institutionCode: CASC
- **Type status:** Other material. **Occurrence:** catalogNumber: casent0050357; recordedBy: Malagasy ant team; sex: 3w; preparations: pin; associatedMedia: http://www.antweb.org/specimen/casent0050357; **Taxon:** scientificName: Stigmatomma roahady; genus: Stigmatomma; **Location:** country: Madagascar; stateProvince: Toamasina; locality: Forêt Ambatovy, 14.3 km 57° Moramanga; verbatimElevation: 1075; decimalLatitude: -18.85083; decimalLongitude: 48.32; georeferenceRemarks: coordinates obtained from GPS; **Event:** samplingProtocol: General collecting; eventDate: 03/22/2004; habitat: montane rainforest; fieldNumber: BLF10567; eventRemarks: ex rotten log; **Record Level:** institutionCode: CASC
- **Type status:** Other material. **Occurrence:** catalogNumber: casent0050358; recordedBy: Malagasy ant team; sex: 2w; preparations: pin; associatedMedia: http://www.antweb.org/specimen/casent0050358; **Taxon:** scientificName: Stigmatomma roahady; genus: Stigmatomma; **Location:** country: Madagascar; stateProvince: Toamasina; locality: Forêt Ambatovy, 14.3 km 57° Moramanga; verbatimElevation: 1075; decimalLatitude: -18.85083; decimalLongitude: 48.32; georeferenceRemarks: coordinates obtained from GPS; **Event:** samplingProtocol: General collecting; eventDate: 03/23/2004; habitat: montane rainforest; fieldNumber: BLF10605; eventRemarks: ex rotten log; **Record Level:** institutionCode: CASC
- **Type status:** Other material. **Occurrence:** catalogNumber: casent0056914; recordedBy: B.L.Fisher; sex: 1w; preparations: pin; associatedMedia: http://www.antweb.org/specimen/casent0056914; **Taxon:** scientificName: Stigmatomma roahady; genus: Stigmatomma; **Location:** country: Madagascar; stateProvince: Toamasina; locality: Forêt Ambatovy, 14.3 km 57° Moramanga; verbatimElevation: 1075; decimalLatitude: -18.85083; decimalLongitude: 48.32; georeferenceRemarks: coordinates obtained from GPS; **Event:** samplingProtocol: General collecting; eventDate: 04/12/2005; habitat: montane rainforest; fieldNumber: BLF11964; eventRemarks: ex rotten log; **Record Level:** institutionCode: CASC
- **Type status:** Other material. **Occurrence:** catalogNumber: casent0056916; recordedBy: B.L.Fisher; sex: 1w; preparations: SEM mount; associatedMedia: http://www.antweb.org/specimen/casent0056916; **Taxon:** scientificName: Stigmatomma roahady; genus: Stigmatomma; **Location:** country: Madagascar; stateProvince: Toamasina; locality: Forêt Ambatovy, 14.3 km 57° Moramanga; verbatimElevation: 1075; decimalLatitude: -18.85083; decimalLongitude: 48.32; georeferenceRemarks: coordinates obtained from GPS; **Event:** samplingProtocol: General collecting; eventDate: 04/12/2005; habitat: montane rainforest; fieldNumber: BLF11961; eventRemarks: ex rotten log; **Record Level:** institutionCode: CASC
- **Type status:** Other material. **Occurrence:** catalogNumber: casent0058814; recordedBy: B.L.Fisher; sex: 1w; preparations: pin; associatedMedia: http://www.antweb.org/specimen/casent0058814; **Taxon:** scientificName: Stigmatomma roahady; genus: Stigmatomma; **Location:** country: Madagascar; stateProvince: Toamasina; locality: Forêt Ambatovy, 14.3 km 57° Moramanga; verbatimElevation: 1075; decimalLatitude: -18.85083; decimalLongitude: 48.32; georeferenceRemarks: coordinates obtained from GPS; **Event:** samplingProtocol: General collecting; eventDate: 04/12/2005; habitat: montane rainforest; fieldNumber: BLF11965; eventRemarks: ex litter sample; **Record Level:** institutionCode: CASC
- **Type status:** Other material. **Occurrence:** catalogNumber: casent0060895; recordedBy: B.L.Fisher et al.; sex: 1w; preparations: pin; associatedMedia: http://www.antweb.org/specimen/casent0060895; **Taxon:** scientificName: Stigmatomma roahady; genus: Stigmatomma; **Location:** country: Madagascar; stateProvince: Fianarantsoa; locality: 2km W Andrambovato, along river Tatamaly; verbatimElevation: 1075; decimalLatitude: -21.51167; decimalLongitude: 47.41; georeferenceRemarks: coordinates obtained from GPS; **Event:** samplingProtocol: General collecting; eventDate: 06/03/2005; habitat: montane rainforest; fieldNumber: BLF12189; eventRemarks: under moss, on rotten log; **Record Level:** institutionCode: CASC
- **Type status:** Other material. **Occurrence:** catalogNumber: casent0060911; recordedBy: B.L.Fisher et al.; sex: 1w; preparations: pin; associatedMedia: http://www.antweb.org/specimen/casent0060911; **Taxon:** scientificName: Stigmatomma roahady; genus: Stigmatomma; **Location:** country: Madagascar; stateProvince: Fianarantsoa; locality: 2km W Andrambovato, along river Tatamaly; verbatimElevation: 1075; decimalLatitude: -21.51167; decimalLongitude: 47.41; georeferenceRemarks: coordinates obtained from GPS; **Event:** samplingProtocol: 7 MaxiWinks; eventDate: 06/03/2005; habitat: montane rainforest; fieldNumber: BLF12164; eventRemarks: sifted litter (leaf mold, rotten wood); **Record Level:** institutionCode: CASC
- **Type status:** Other material. **Occurrence:** catalogNumber: casent0061449; recordedBy: B.L.Fisher et al.; sex: 1w; preparations: pin; associatedMedia: http://www.antweb.org/specimen/casent0061449; **Taxon:** scientificName: Stigmatomma roahady; genus: Stigmatomma; **Location:** country: Madagascar; stateProvince: Fianarantsoa; locality: 2km W Andrambovato, along river Tatamaly; verbatimElevation: 1075; decimalLatitude: -21.51167; decimalLongitude: 47.41; georeferenceRemarks: coordinates obtained from GPS; **Event:** samplingProtocol: 7 MaxiWinks; eventDate: 06/03/2005; habitat: montane rainforest; fieldNumber: BLF12164; eventRemarks: sifted litter (leaf mold, rotten wood); **Record Level:** institutionCode: CASC
- **Type status:** Other material. **Occurrence:** catalogNumber: casent0073990; recordedBy: Malagasy ant team; sex: 1w; preparations: pin; associatedMedia: http://www.antweb.org/specimen/casent0073990; **Taxon:** scientificName: Stigmatomma roahady; genus: Stigmatomma; **Location:** country: Madagascar; stateProvince: Toamasina; locality: Forêt Ambatovy, 14.3 km 57° Moramanga; verbatimElevation: 1075; decimalLatitude: -18.85083; decimalLongitude: 48.32; georeferenceRemarks: coordinates obtained from GPS; **Event:** samplingProtocol: MW, 25 sifted litter; eventDate: 03/21/2004; habitat: montane rainforest; fieldNumber: BLF10501; eventRemarks: sifted litter (leaf mold, rotten wood); **Record Level:** institutionCode: CASC
- **Type status:** Other material. **Occurrence:** catalogNumber: casent0074178; recordedBy: Malagasy ant team; sex: 1w; preparations: pin; associatedMedia: http://www.antweb.org/specimen/casent0074178; **Taxon:** scientificName: Stigmatomma roahady; genus: Stigmatomma; **Location:** country: Madagascar; stateProvince: Toamasina; locality: Forêt Ambatovy, 14.3 km 57° Moramanga; verbatimElevation: 1075; decimalLatitude: -18.85083; decimalLongitude: 48.32; georeferenceRemarks: coordinates obtained from GPS; **Event:** samplingProtocol: MW, 25 sifted litter; eventDate: 03/21/2004; habitat: montane rainforest; fieldNumber: BLF10501; eventRemarks: sifted litter (leaf mold, rotten wood); **Record Level:** institutionCode: CASC
- **Type status:** Other material. **Occurrence:** catalogNumber: casent0107483; recordedBy: B.L.Fisher; sex: 1w, 1m; preparations: pin; associatedMedia: http://www.antweb.org/specimen/casent0107483; **Taxon:** scientificName: Stigmatomma roahady; genus: Stigmatomma; **Location:** country: Madagascar; stateProvince: Toamasina; locality: Forêt Ambatovy, 14.3 km 57° Moramanga; verbatimElevation: 1075; decimalLatitude: -18.85083; decimalLongitude: 48.32; georeferenceRemarks: coordinates obtained from GPS; **Event:** samplingProtocol: General collecting; eventDate: 12/18/2004; habitat: montane rainforest; fieldNumber: BLF11829; eventRemarks: ex rotten log; **Record Level:** institutionCode: CASC
- **Type status:** Other material. **Occurrence:** catalogNumber: casent0318416; recordedBy: B.L.Fisher; sex: 1w; preparations: SEM mount; associatedMedia: http://www.antweb.org/specimen/casent0318416; **Taxon:** scientificName: Stigmatomma janovitsika; genus: Stigmatomma; **Location:** country: Madagascar; stateProvince: Toamasina; locality: Forêt Ambatovy, 14.3 km 57° Moramanga; verbatimElevation: 1075; decimalLatitude: -18.85083; decimalLongitude: 48.32; georeferenceRemarks: coordinates obtained from GPS; **Event:** samplingProtocol: General collecting; eventDate: 12/18/2004; habitat: montane rainforest; fieldNumber: BLF11861; eventRemarks: ex rotten log; **Record Level:** institutionCode: CASC
- **Type status:** Other material. **Occurrence:** catalogNumber: casent0121324; recordedBy: B.L.Fisher et al.; sex: 1w; preparations: pin; associatedMedia: http://www.antweb.org/specimen/casent0121324; **Taxon:** scientificName: Stigmatomma roahady; genus: Stigmatomma; **Location:** country: Madagascar; stateProvince: Toamasina; locality: Ambatovy, 12.4 km NE Moramanga; verbatimElevation: 1080; decimalLatitude: -18.83937; decimalLongitude: 48.30842; georeferenceRemarks: coordinates obtained from GPS; **Event:** samplingProtocol: General collecting; eventDate: 03/08/2007; habitat: montane rainforest; fieldNumber: BLF16935; eventRemarks: ground forager(s); **Record Level:** institutionCode: CASC
- **Type status:** Other material. **Occurrence:** catalogNumber: casent0123506; recordedBy: B.L.Fisher et al.; sex: 1w; preparations: pin; associatedMedia: http://www.antweb.org/specimen/casent0123506; **Taxon:** scientificName: Stigmatomma roahady; genus: Stigmatomma; **Location:** country: Madagascar; stateProvince: Toamasina; locality: Ambatovy, 12.4 km NE Moramanga; verbatimElevation: 1080; decimalLatitude: -18.83937; decimalLongitude: 48.30842; georeferenceRemarks: coordinates obtained from GPS; **Event:** samplingProtocol: MW 25 sample transect, 5m; eventDate: 03/04/2007; habitat: montane rainforest; fieldNumber: BLF16916; eventRemarks: sifted litter (leaf mold, rotten wood); **Record Level:** institutionCode: CASC
- **Type status:** Other material. **Occurrence:** catalogNumber: casent0123508; recordedBy: B.L.Fisher et al.; sex: 1w; preparations: pin; associatedMedia: http://www.antweb.org/specimen/casent0123508; **Taxon:** scientificName: Stigmatomma roahady; genus: Stigmatomma; **Location:** country: Madagascar; stateProvince: Toamasina; locality: Ambatovy, 12.4 km NE Moramanga; verbatimElevation: 1080; decimalLatitude: -18.83937; decimalLongitude: 48.30842; georeferenceRemarks: coordinates obtained from GPS; **Event:** samplingProtocol: MW 25 sample transect, 5m; eventDate: 03/04/2007; habitat: montane rainforest; fieldNumber: BLF16916; eventRemarks: sifted litter (leaf mold, rotten wood); **Record Level:** institutionCode: CASC
- **Type status:** Other material. **Occurrence:** catalogNumber: casent0123509; recordedBy: B.L.Fisher et al.; sex: 1w; preparations: pin; associatedMedia: http://www.antweb.org/specimen/casent0123509; **Taxon:** scientificName: Stigmatomma roahady; genus: Stigmatomma; **Location:** country: Madagascar; stateProvince: Toamasina; locality: Ambatovy, 12.4 km NE Moramanga; verbatimElevation: 1080; decimalLatitude: -18.83937; decimalLongitude: 48.30842; georeferenceRemarks: coordinates obtained from GPS; **Event:** samplingProtocol: MW 25 sample transect, 5m; eventDate: 03/04/2007; habitat: montane rainforest; fieldNumber: BLF16916; eventRemarks: sifted litter (leaf mold, rotten wood); **Record Level:** institutionCode: CASC
- **Type status:** Other material. **Occurrence:** catalogNumber: casent0123510; recordedBy: B.L.Fisher et al.; sex: 1w; preparations: pin; associatedMedia: http://www.antweb.org/specimen/casent0123510; **Taxon:** scientificName: Stigmatomma roahady; genus: Stigmatomma; **Location:** country: Madagascar; stateProvince: Toamasina; locality: Ambatovy, 12.4 km NE Moramanga; verbatimElevation: 1080; decimalLatitude: -18.83937; decimalLongitude: 48.30842; georeferenceRemarks: coordinates obtained from GPS; **Event:** samplingProtocol: MW 25 sample transect, 5m; eventDate: 03/04/2007; habitat: montane rainforest; fieldNumber: BLF16916; eventRemarks: sifted litter (leaf mold, rotten wood); **Record Level:** institutionCode: CASC
- **Type status:** Other material. **Occurrence:** catalogNumber: casent0123511; recordedBy: B.L.Fisher et al.; sex: 1w; preparations: pin; associatedMedia: http://www.antweb.org/specimen/casent0123511; **Taxon:** scientificName: Stigmatomma roahady; genus: Stigmatomma; **Location:** country: Madagascar; stateProvince: Toamasina; locality: Ambatovy, 12.4 km NE Moramanga; verbatimElevation: 1080; decimalLatitude: -18.83937; decimalLongitude: 48.30842; georeferenceRemarks: coordinates obtained from GPS; **Event:** samplingProtocol: MW 25 sample transect, 5m; eventDate: 03/04/2007; habitat: montane rainforest; fieldNumber: BLF16916; eventRemarks: sifted litter (leaf mold, rotten wood); **Record Level:** institutionCode: CASC
- **Type status:** Other material. **Occurrence:** catalogNumber: casent0034838; recordedBy: Fisher, Griswold et al.; sex: 1w; preparations: pin; associatedMedia: http://www.antweb.org/specimen/casent0034838; **Taxon:** scientificName: Stigmatomma roahady; genus: Stigmatomma; **Location:** country: Madagascar; stateProvince: Fianarantsoa; locality: Parc National de Ranomafana, Vatoharanana River, 4.1 km 231° SW Ranomafana; verbatimElevation: 1100; decimalLatitude: -21.29; decimalLongitude: 47.43333; georeferenceRemarks: coordinates obtained from GPS; **Event:** samplingProtocol: MW 50 sample transect, 5m; eventDate: 03/27/2003; habitat: montane rainforest; fieldNumber: BLF08400; eventRemarks: sifted litter (leaf mold, rotten wood); **Record Level:** institutionCode: CASC
- **Type status:** Other material. **Occurrence:** catalogNumber: casent0034841; recordedBy: Fisher, Griswold et al.; sex: 1w; preparations: pin; associatedMedia: http://www.antweb.org/specimen/casent0034841; **Taxon:** scientificName: Stigmatomma roahady; genus: Stigmatomma; **Location:** country: Madagascar; stateProvince: Fianarantsoa; locality: Parc National de Ranomafana, Vatoharanana River, 4.1 km 231° SW Ranomafana; verbatimElevation: 1100; decimalLatitude: -21.29; decimalLongitude: 47.43333; georeferenceRemarks: coordinates obtained from GPS; **Event:** samplingProtocol: MW 50 sample transect, 5m; eventDate: 03/27/2003; habitat: montane rainforest; fieldNumber: BLF08400; eventRemarks: sifted litter (leaf mold, rotten wood); **Record Level:** institutionCode: CASC
- **Type status:** Other material. **Occurrence:** catalogNumber: casent0042844; recordedBy: B.L.Fisher; sex: 1w; preparations: pin; associatedMedia: http://www.antweb.org/specimen/casent0042844; **Taxon:** scientificName: Stigmatomma roahady; genus: Stigmatomma; **Location:** country: Madagascar; stateProvince: Antsiranana; locality: Forêt de Binara, 9.4km 235° SW Daraina; verbatimElevation: 1100; decimalLatitude: -13.26333; decimalLongitude: 49.6; georeferenceRemarks: coordinates obtained from GPS; **Event:** samplingProtocol: MW 25 sample transect, 5m; eventDate: 12/05/2003; habitat: montane rainforest; fieldNumber: BLF09800; eventRemarks: sifted litter (leaf mold, rotten wood); **Record Level:** institutionCode: CASC
- **Type status:** Other material. **Occurrence:** catalogNumber: casent0042846; recordedBy: B.L.Fisher; sex: 1w; preparations: pin; associatedMedia: http://www.antweb.org/specimen/casent0042846; **Taxon:** scientificName: Stigmatomma roahady; genus: Stigmatomma; **Location:** country: Madagascar; stateProvince: Antsiranana; locality: Forêt de Binara, 9.4km 235° SW Daraina; verbatimElevation: 1100; decimalLatitude: -13.26333; decimalLongitude: 49.6; georeferenceRemarks: coordinates obtained from GPS; **Event:** samplingProtocol: MW 25 sample transect, 5m; eventDate: 12/05/2003; habitat: montane rainforest; fieldNumber: BLF09800; eventRemarks: sifted litter (leaf mold, rotten wood); **Record Level:** institutionCode: CASC
- **Type status:** Other material. **Occurrence:** catalogNumber: casent0042895; recordedBy: B.L.Fisher; sex: 1w; preparations: pin; associatedMedia: http://www.antweb.org/specimen/casent0042895; **Taxon:** scientificName: Stigmatomma roahady; genus: Stigmatomma; **Location:** country: Madagascar; stateProvince: Antsiranana; locality: Forêt de Binara, 9.4km 235° SW Daraina; verbatimElevation: 1100; decimalLatitude: -13.26333; decimalLongitude: 49.6; georeferenceRemarks: coordinates obtained from GPS; **Event:** samplingProtocol: MW 25 sample transect, 5m; eventDate: 12/05/2003; habitat: montane rainforest; fieldNumber: BLF09800; eventRemarks: sifted litter (leaf mold, rotten wood); **Record Level:** institutionCode: CASC
- **Type status:** Other material. **Occurrence:** catalogNumber: casent0042896; recordedBy: B.L.Fisher; sex: 1w; preparations: pin; associatedMedia: http://www.antweb.org/specimen/casent0042896; **Taxon:** scientificName: Stigmatomma roahady; genus: Stigmatomma; **Location:** country: Madagascar; stateProvince: Antsiranana; locality: Forêt de Binara, 9.4km 235° SW Daraina; verbatimElevation: 1100; decimalLatitude: -13.26333; decimalLongitude: 49.6; georeferenceRemarks: coordinates obtained from GPS; **Event:** samplingProtocol: MW 25 sample transect, 5m; eventDate: 12/05/2003; habitat: montane rainforest; fieldNumber: BLF09800; eventRemarks: sifted litter (leaf mold, rotten wood); **Record Level:** institutionCode: CASC
- **Type status:** Other material. **Occurrence:** catalogNumber: casent0071992; recordedBy: Val C.; sex: 1w; preparations: pin; associatedMedia: http://www.antweb.org/specimen/casent0071992; **Taxon:** scientificName: Stigmatomma roahady; genus: Stigmatomma; **Location:** country: Madagascar; stateProvince: Fianarantsoa; locality: Vatoharanana; verbatimElevation: 1100; decimalLatitude: -21.29067; decimalLongitude: 47.42617; **Event:** eventDate: 03/31/2003; habitat: old-growth primary forest; fieldNumber: VCR004; **Record Level:** institutionCode: CASC
- **Type status:** Other material. **Occurrence:** catalogNumber: casent0073287; recordedBy: Val C.; sex: 1w; preparations: pin; associatedMedia: http://www.antweb.org/specimen/casent0073287; **Taxon:** scientificName: Stigmatomma roahady; genus: Stigmatomma; **Location:** country: Madagascar; locality: Sanavonarona; verbatimElevation: 1100; decimalLatitude: -21.2575; decimalLongitude: 47.35317; **Event:** eventDate: 03/18/2003; habitat: disturbed roadside forest; fieldNumber: VCR002; **Record Level:** institutionCode: CASC
- **Type status:** Other material. **Occurrence:** catalogNumber: casent0073545; recordedBy: Fisher, Griswold et al.; sex: 1w; preparations: pin; associatedMedia: http://www.antweb.org/specimen/casent0073545; **Taxon:** scientificName: Stigmatomma roahady; genus: Stigmatomma; **Location:** country: Madagascar; stateProvince: Fianarantsoa; locality: Parc National de Ranomafana, Vatoharanana River, 4.1 km 231° SW Ranomafana; verbatimElevation: 1100; decimalLatitude: -21.29; decimalLongitude: 47.43333; georeferenceRemarks: coordinates obtained from GPS; **Event:** samplingProtocol: pitfall trap, PF 50 traps, 11 cm dbh with water, soap, formalin, nonlinear placement; eventDate: 03/27/2003; habitat: montane rainforest; fieldNumber: BLF08402; **Record Level:** institutionCode: CASC
- **Type status:** Other material. **Occurrence:** catalogNumber: casent0497126; recordedBy: Fisher, Griswold et al.; sex: 3w; preparations: pin; associatedMedia: http://www.antweb.org/specimen/casent0497126; **Taxon:** scientificName: Stigmatomma roahady; genus: Stigmatomma; **Location:** country: Madagascar; stateProvince: Fianarantsoa; locality: Parc National de Ranomafana, Vatoharanana River, 4.1 km 231° SW Ranomafana; verbatimElevation: 1100; decimalLatitude: -21.29; decimalLongitude: 47.43333; georeferenceRemarks: coordinates obtained from GPS; **Event:** samplingProtocol: General collecting; eventDate: 03/27/2003; habitat: montane rainforest; fieldNumber: BLF08430; eventRemarks: ex rotten log; **Record Level:** institutionCode: CASC
- **Type status:** Other material. **Occurrence:** catalogNumber: casent0497127; recordedBy: Fisher, Griswold et al.; sex: 3w; preparations: pin; associatedMedia: http://www.antweb.org/specimen/casent0497127; **Taxon:** scientificName: Stigmatomma roahady; genus: Stigmatomma; **Location:** country: Madagascar; stateProvince: Fianarantsoa; locality: Parc National de Ranomafana, Vatoharanana River, 4.1 km 231° SW Ranomafana; verbatimElevation: 1100; decimalLatitude: -21.29; decimalLongitude: 47.43333; georeferenceRemarks: coordinates obtained from GPS; **Event:** samplingProtocol: General collecting; eventDate: 03/27/2003; habitat: montane rainforest; fieldNumber: BLF08430; eventRemarks: ex rotten log; **Record Level:** institutionCode: CASC
- **Type status:** Other material. **Occurrence:** catalogNumber: casent0497128; recordedBy: Fisher, Griswold et al.; sex: 3w; preparations: pin; associatedMedia: http://www.antweb.org/specimen/casent0497128; **Taxon:** scientificName: Stigmatomma roahady; genus: Stigmatomma; **Location:** country: Madagascar; stateProvince: Fianarantsoa; locality: Parc National de Ranomafana, Vatoharanana River, 4.1 km 231° SW Ranomafana; verbatimElevation: 1100; decimalLatitude: -21.29; decimalLongitude: 47.43333; georeferenceRemarks: coordinates obtained from GPS; **Event:** samplingProtocol: General collecting; eventDate: 03/27/2003; habitat: montane rainforest; fieldNumber: BLF08430; eventRemarks: ex rotten log; **Record Level:** institutionCode: CASC
- **Type status:** Other material. **Occurrence:** catalogNumber: casent0497129; recordedBy: Fisher, Griswold et al.; sex: 3w; preparations: pin; associatedMedia: http://www.antweb.org/specimen/casent0497129; **Taxon:** scientificName: Stigmatomma roahady; genus: Stigmatomma; **Location:** country: Madagascar; stateProvince: Fianarantsoa; locality: Parc National de Ranomafana, Vatoharanana River, 4.1 km 231° SW Ranomafana; verbatimElevation: 1100; decimalLatitude: -21.29; decimalLongitude: 47.43333; georeferenceRemarks: coordinates obtained from GPS; **Event:** samplingProtocol: General collecting; eventDate: 03/27/2003; habitat: montane rainforest; fieldNumber: BLF08430; eventRemarks: ex rotten log; **Record Level:** institutionCode: CASC
- **Type status:** Other material. **Occurrence:** catalogNumber: casent0497130; recordedBy: Fisher, Griswold et al.; sex: 3w; preparations: pin; associatedMedia: http://www.antweb.org/specimen/casent0497130; **Taxon:** scientificName: Stigmatomma roahady; genus: Stigmatomma; **Location:** country: Madagascar; stateProvince: Fianarantsoa; locality: Parc National de Ranomafana, Vatoharanana River, 4.1 km 231° SW Ranomafana; verbatimElevation: 1100; decimalLatitude: -21.29; decimalLongitude: 47.43333; georeferenceRemarks: coordinates obtained from GPS; **Event:** samplingProtocol: General collecting; eventDate: 03/27/2003; habitat: montane rainforest; fieldNumber: BLF08430; eventRemarks: ex rotten log; **Record Level:** institutionCode: CASC
- **Type status:** Other material. **Occurrence:** catalogNumber: casent0497131; recordedBy: Fisher, Griswold et al.; sex: 2w; preparations: pin; associatedMedia: http://www.antweb.org/specimen/casent0497131; **Taxon:** scientificName: Stigmatomma roahady; genus: Stigmatomma; **Location:** country: Madagascar; stateProvince: Fianarantsoa; locality: Parc National de Ranomafana, Vatoharanana River, 4.1 km 231° SW Ranomafana; verbatimElevation: 1100; decimalLatitude: -21.29; decimalLongitude: 47.43333; georeferenceRemarks: coordinates obtained from GPS; **Event:** samplingProtocol: General collecting; eventDate: 03/27/2003; habitat: montane rainforest; fieldNumber: BLF08430; eventRemarks: ex rotten log; **Record Level:** institutionCode: CASC
- **Type status:** Other material. **Occurrence:** catalogNumber: casent0497132; recordedBy: Fisher, Griswold et al.; sex: 1w; preparations: pin; associatedMedia: http://www.antweb.org/specimen/casent0497132; **Taxon:** scientificName: Stigmatomma roahady; genus: Stigmatomma; **Location:** country: Madagascar; stateProvince: Fianarantsoa; locality: Parc National de Ranomafana, Vatoharanana River, 4.1 km 231° SW Ranomafana; verbatimElevation: 1100; decimalLatitude: -21.29; decimalLongitude: 47.43333; georeferenceRemarks: coordinates obtained from GPS; **Event:** samplingProtocol: General collecting; eventDate: 03/27/2003; habitat: montane rainforest; fieldNumber: BLF08430; eventRemarks: ex rotten log; **Record Level:** institutionCode: CASC
- **Type status:** Other material. **Occurrence:** catalogNumber: casent0497186; recordedBy: Fisher, Griswold et al.; sex: 1w; preparations: pin; associatedMedia: http://www.antweb.org/specimen/casent0497186; **Taxon:** scientificName: Stigmatomma roahady; genus: Stigmatomma; **Location:** country: Madagascar; stateProvince: Fianarantsoa; locality: Parc National de Ranomafana, Vatoharanana River, 4.1 km 231° SW Ranomafana; verbatimElevation: 1100; decimalLatitude: -21.29; decimalLongitude: 47.43333; georeferenceRemarks: coordinates obtained from GPS; **Event:** samplingProtocol: General collecting; eventDate: 03/27/2003; habitat: montane rainforest; fieldNumber: BLF08546; eventRemarks: ex rotten log; **Record Level:** institutionCode: CASC
- **Type status:** Other material. **Occurrence:** catalogNumber: casent0112603; recordedBy: Rin'ha, Mike; sex: 1aQ; preparations: pin; associatedMedia: http://www.antweb.org/specimen/casent0112603; **Taxon:** scientificName: Stigmatomma roahady; genus: Stigmatomma; **Location:** country: Madagascar; stateProvince: Fianarantsoa; locality: Vatovavy Fitovinany Region,Distric of Ifanadiana, Ranomafana National Park. Vohiparara bridge,17Km W of Ranomafana; verbatimElevation: 1109; decimalLatitude: -21.22617; decimalLongitude: 47.36983; **Event:** samplingProtocol: Malaise; eventDate: 03/12/2002; habitat: rainforest, high altitude; fieldNumber: MG-09A-20; **Record Level:** institutionCode: CASC
- **Type status:** Other material. **Occurrence:** catalogNumber: casent0112177; recordedBy: R. Harin'Hala; sex: 1aQ; preparations: pin; associatedMedia: http://www.antweb.org/specimen/casent0112177; **Taxon:** scientificName: Stigmatomma roahady; genus: Stigmatomma; **Location:** country: Madagascar; stateProvince: Fianarantsoa; locality: Vohiparara broken bridge, Fianarantsoa Prov.; verbatimElevation: 1110; decimalLatitude: -21.22617; decimalLongitude: 47.36983; **Event:** samplingProtocol: Malaise trap; eventDate: 04/29/2002; habitat: high altitude rainforest; fieldNumber: MA-02-09A-27; **Record Level:** institutionCode: CASC
- **Type status:** Other material. **Occurrence:** catalogNumber: casent0206406; recordedBy: Rin'ha, Mike; sex: 1aQ; preparations: pin; associatedMedia: http://www.antweb.org/specimen/casent0206406; **Taxon:** scientificName: Stigmatomma roahady; genus: Stigmatomma; **Location:** country: Madagascar; stateProvince: Fianarantsoa; locality: Fitovavy Fitovinany Region, Distric of Ifanadiana, 12Km W of Ranomafana; verbatimElevation: 1127; decimalLatitude: -21.25083; decimalLongitude: 47.40717; **Event:** samplingProtocol: Malaise, canopy trap; eventDate: 01/20/2006; habitat: forest edge, open area; fieldNumber: MG-09B-137; **Record Level:** institutionCode: CASC
- **Type status:** Other material. **Occurrence:** catalogNumber: casent0476591; recordedBy: B.L.Fisher; sex: 1w; preparations: pin; associatedMedia: http://www.antweb.org/specimen/casent0476591; **Taxon:** scientificName: Stigmatomma roahady; genus: Stigmatomma; **Location:** country: Madagascar; stateProvince: Antsiranana; locality: R.S. Manongarivo, 14.5 km 220° SW Antanambao; verbatimElevation: 1175; decimalLatitude: -13.99833; decimalLongitude: 48.42833; **Event:** samplingProtocol: MW 75 sample transect, 5,10m; eventDate: 10/20/1998; habitat: montane rainforest; fieldNumber: BLF01938; eventRemarks: sifted litter (leaf mold, rotten wood); **Record Level:** institutionCode: CASC
- **Type status:** Other material. **Occurrence:** catalogNumber: blf1747(25)-1; recordedBy: B.L.Fisher (Sylvain); sex: 1w; preparations: pin; associatedMedia: http://www.antweb.org/specimen/blf1747(25)-1; **Taxon:** scientificName: Stigmatomma roahady; genus: Stigmatomma; **Location:** country: Madagascar; stateProvince: Fianarantsoa; locality: R.S. Ivohibe 8.0 km E Ivohibe; verbatimElevation: 1200; decimalLatitude: -22.48333; decimalLongitude: 46.96833; **Event:** samplingProtocol: MW 50 sample transect, 5m; eventDate: 10/15/1997; habitat: montane rainforest; fieldNumber: BLF01747; eventRemarks: sifted litter (leaf mold, rotten wood); **Record Level:** institutionCode: CASC
- **Type status:** Other material. **Occurrence:** catalogNumber: casent0008704; recordedBy: B.L.Fisher (Sylvain); sex: 1w; preparations: pin; associatedMedia: http://www.antweb.org/specimen/casent0008704; **Taxon:** scientificName: Stigmatomma roahady; genus: Stigmatomma; **Location:** country: Madagascar; stateProvince: Fianarantsoa; locality: R.S. Ivohibe 8.0 km E Ivohibe; verbatimElevation: 1200; decimalLatitude: -22.48333; decimalLongitude: 46.96833; **Event:** samplingProtocol: MW 50 sample transect, 5m; eventDate: 10/15/1997; habitat: montane rainforest; fieldNumber: BLF01747; eventRemarks: sifted litter (leaf mold, rotten wood); **Record Level:** institutionCode: CASC
- **Type status:** Other material. **Occurrence:** catalogNumber: casent0746574; recordedBy: B.L.Fisher (Sylvain); sex: 1w; preparations: pin; associatedMedia: http://www.antweb.org/specimen/casent0746574; **Taxon:** scientificName: Stigmatomma roahady; genus: Stigmatomma; **Location:** country: Madagascar; stateProvince: Fianarantsoa; locality: 8.0 km NE Ivohibe; verbatimElevation: 1200; decimalLatitude: -22.42167; decimalLongitude: 46.89833; **Event:** samplingProtocol: MW 50 sample transect, 5m; eventDate: 11/03/1997; habitat: montane rainforest; fieldNumber: BLF01753; eventRemarks: sifted litter (leaf mold, rotten wood); **Record Level:** institutionCode: CASC
- **Type status:** Other material. **Occurrence:** catalogNumber: casent0746582; recordedBy: B.L.Fisher (Sylvain); sex: 1w; preparations: pin; associatedMedia: http://www.antweb.org/specimen/casent0746582; **Taxon:** scientificName: Stigmatomma roahady; genus: Stigmatomma; **Location:** country: Madagascar; stateProvince: Fianarantsoa; locality: 8.0 km NE Ivohibe; verbatimElevation: 1200; decimalLatitude: -22.42167; decimalLongitude: 46.89833; **Event:** samplingProtocol: MW 50 sample transect, 5m; eventDate: 11/03/1997; habitat: montane rainforest; fieldNumber: BLF01753; eventRemarks: sifted litter (leaf mold, rotten wood); **Record Level:** institutionCode: CASC
- **Type status:** Other material. **Occurrence:** catalogNumber: casent0746583; recordedBy: B.L.Fisher (Sylvain); sex: 1w; preparations: pin; associatedMedia: http://www.antweb.org/specimen/casent0746583; **Taxon:** scientificName: Stigmatomma roahady; genus: Stigmatomma; **Location:** country: Madagascar; stateProvince: Fianarantsoa; locality: 8.0 km NE Ivohibe; verbatimElevation: 1200; decimalLatitude: -22.42167; decimalLongitude: 46.89833; **Event:** samplingProtocol: MW 50 sample transect, 5m; eventDate: 11/03/1997; habitat: montane rainforest; fieldNumber: BLF01753; eventRemarks: sifted litter (leaf mold, rotten wood); **Record Level:** institutionCode: CASC
- **Type status:** Other material. **Occurrence:** catalogNumber: casent0746585; recordedBy: B.L.Fisher (Sylvain); sex: 1w; preparations: pin; associatedMedia: http://www.antweb.org/specimen/casent0746585; **Taxon:** scientificName: Stigmatomma roahady; genus: Stigmatomma; **Location:** country: Madagascar; stateProvince: Fianarantsoa; locality: R.S. Ivohibe 8.0 km E Ivohibe; verbatimElevation: 1200; decimalLatitude: -22.48333; decimalLongitude: 46.96833; **Event:** samplingProtocol: MW 50 sample transect, 5m; eventDate: 10/15/1997; habitat: montane rainforest; fieldNumber: BLF01747; eventRemarks: sifted litter (leaf mold, rotten wood); **Record Level:** institutionCode: CASC
- **Type status:** Other material. **Occurrence:** catalogNumber: casent0133735; recordedBy: B.L.Fisher et al.; sex: 1w; preparations: pin; associatedMedia: http://www.antweb.org/specimen/casent0133735; **Taxon:** scientificName: Stigmatomma roahady; genus: Stigmatomma; **Location:** country: Madagascar; stateProvince: Antsiranana; locality: Parc National Montagne d'Ambre, Lac maudit; verbatimElevation: 1250; decimalLatitude: -12.58502; decimalLongitude: 49.15147; georeferenceRemarks: coordinates obtained from GPS; **Event:** samplingProtocol: General collecting; eventDate: 11/13/2007; habitat: montane rainforest; fieldNumber: BLF18080; eventRemarks: ex rotten log; **Record Level:** institutionCode: CASC
- **Type status:** Other material. **Occurrence:** catalogNumber: casent0746578; recordedBy: B.L.Fisher; sex: 1w; preparations: pin; associatedMedia: http://www.antweb.org/specimen/casent0746578; **Taxon:** scientificName: Stigmatomma roahady; genus: Stigmatomma; **Location:** country: Madagascar; stateProvince: Toliara; locality: 13 km NW Enakara, Rés. Andohahela; verbatimElevation: 1250; decimalLatitude: -24.55; decimalLongitude: 46.8; **Event:** samplingProtocol: MW 50 sample transect, 5m; eventDate: 11/30/1992; habitat: montane rainforest; fieldNumber: BLF00561; eventRemarks: sifted litter (leaf mold, rotten wood); **Record Level:** institutionCode: CASC
- **Type status:** Other material. **Occurrence:** catalogNumber: casent0746579; recordedBy: B.L.Fisher; sex: 1w; preparations: pin; associatedMedia: http://www.antweb.org/specimen/casent0746579; **Taxon:** scientificName: Stigmatomma roahady; genus: Stigmatomma; **Location:** country: Madagascar; stateProvince: Toliara; locality: 13 km NW Enakara, Rés. Andohahela; verbatimElevation: 1250; decimalLatitude: -24.55; decimalLongitude: 46.8; **Event:** samplingProtocol: MW 50 sample transect, 5m; eventDate: 11/30/1992; habitat: montane rainforest; fieldNumber: BLF00561; eventRemarks: sifted litter (leaf mold, rotten wood); **Record Level:** institutionCode: CASC
- **Type status:** Other material. **Occurrence:** catalogNumber: casent0746580; recordedBy: B.L.Fisher; sex: 1w; preparations: pin; associatedMedia: http://www.antweb.org/specimen/casent0746580; **Taxon:** scientificName: Stigmatomma roahady; genus: Stigmatomma; **Location:** country: Madagascar; stateProvince: Toliara; locality: 13 km NW Enakara, Rés. Andohahela; verbatimElevation: 1250; decimalLatitude: -24.55; decimalLongitude: 46.8; **Event:** samplingProtocol: MW 50 sample transect, 5m; eventDate: 11/30/1992; habitat: montane rainforest; fieldNumber: BLF00561; eventRemarks: sifted litter (leaf mold, rotten wood); **Record Level:** institutionCode: CASC
- **Type status:** Other material. **Occurrence:** catalogNumber: casent0746588; recordedBy: B.L.Fisher; sex: 2w; preparations: pin; associatedMedia: http://www.antweb.org/specimen/casent0746588; **Taxon:** scientificName: Stigmatomma roahady; genus: Stigmatomma; **Location:** country: Madagascar; stateProvince: Toliara; locality: 13 km NW Enakara, Rés. Andohahela; verbatimElevation: 1250; decimalLatitude: -24.55; decimalLongitude: 46.8; **Event:** samplingProtocol: MW 50 sample transect, 5m; eventDate: 11/30/1992; habitat: montane rainforest; fieldNumber: BLF00561; eventRemarks: sifted litter (leaf mold, rotten wood); **Record Level:** institutionCode: CASC
- **Type status:** Other material. **Occurrence:** catalogNumber: casent0746593; recordedBy: B.L.Fisher; sex: 1w; preparations: pin; associatedMedia: http://www.antweb.org/specimen/casent0746593; **Taxon:** scientificName: Stigmatomma roahady; genus: Stigmatomma; **Location:** country: Madagascar; stateProvince: Antsiranana; locality: 9.2 km WSW Befingotra, Rés. Anjanaharibe-Sud; verbatimElevation: 1280; decimalLatitude: -14.75; decimalLongitude: 49.46667; **Event:** samplingProtocol: MW 26 sample transect, 5m; eventDate: 11/05/1994; habitat: montane rainforest; fieldNumber: BLF01158; eventRemarks: sifted litter (leaf mold, rotten wood); **Record Level:** institutionCode: CASC
- **Type status:** Other material. **Occurrence:** catalogNumber: casent0746595; recordedBy: B.L.Fisher; sex: 1w; preparations: pin; associatedMedia: http://www.antweb.org/specimen/casent0746595; **Taxon:** scientificName: Stigmatomma roahady; genus: Stigmatomma; **Location:** country: Madagascar; stateProvince: Antsiranana; locality: 9.2 km WSW Befingotra, Rés. Anjanaharibe-Sud; verbatimElevation: 1280; decimalLatitude: -14.75; decimalLongitude: 49.46667; **Event:** samplingProtocol: MW 26 sample transect, 5m; eventDate: 11/05/1994; habitat: montane rainforest; fieldNumber: BLF01158; eventRemarks: sifted litter (leaf mold, rotten wood); **Record Level:** institutionCode: CASC
- **Type status:** Other material. **Occurrence:** catalogNumber: casent0746596; recordedBy: B.L.Fisher; sex: 1w; preparations: pin; associatedMedia: http://www.antweb.org/specimen/casent0746596; **Taxon:** scientificName: Stigmatomma roahady; genus: Stigmatomma; **Location:** country: Madagascar; stateProvince: Antsiranana; locality: 9.2 km WSW Befingotra, Rés. Anjanaharibe-Sud; verbatimElevation: 1280; decimalLatitude: -14.75; decimalLongitude: 49.46667; **Event:** samplingProtocol: MW 26 sample transect, 5m; eventDate: 11/05/1994; habitat: montane rainforest; fieldNumber: BLF01158; eventRemarks: sifted litter (leaf mold, rotten wood); **Record Level:** institutionCode: CASC
- **Type status:** Other material. **Occurrence:** catalogNumber: casent0703650; recordedBy: B.L.Fisher, F.A. Esteves et al.; sex: 1w; preparations: pin; associatedMedia: http://www.antweb.org/specimen/casent0703650; **Taxon:** scientificName: Stigmatomma roahady; genus: Stigmatomma; **Location:** country: Madagascar; stateProvince: Antananarivo; locality: Reg. Analamanga, St. Forestière Mandraka; verbatimElevation: 1285; decimalLatitude: -18.9183; decimalLongitude: 47.91687; georeferenceRemarks: ±100 m, coordinates obtained from GPS; **Event:** samplingProtocol: General collecting; eventDate: 03/25/2015; habitat: montane rainforest; fieldNumber: BLF37036; eventRemarks: ex soil; **Record Level:** institutionCode: CASC
- **Type status:** Other material. **Occurrence:** catalogNumber: casent0703651; recordedBy: B.L.Fisher, F.A. Esteves et al.; sex: 1w; preparations: pin; associatedMedia: http://www.antweb.org/specimen/casent0703651; **Taxon:** scientificName: Stigmatomma roahady; genus: Stigmatomma; **Location:** country: Madagascar; stateProvince: Antananarivo; locality: Reg. Analamanga, St. Forestière Mandraka; verbatimElevation: 1285; decimalLatitude: -18.9183; decimalLongitude: 47.91687; georeferenceRemarks: ±100 m, coordinates obtained from GPS; **Event:** samplingProtocol: General collecting; eventDate: 03/25/2015; habitat: montane rainforest; fieldNumber: BLF37036; eventRemarks: ex soil; **Record Level:** institutionCode: CASC
- **Type status:** Other material. **Occurrence:** catalogNumber: casent0703652; recordedBy: B.L.Fisher, F.A. Esteves et al.; sex: 1w; preparations: pin; associatedMedia: http://www.antweb.org/specimen/casent0703652; **Taxon:** scientificName: Stigmatomma roahady; genus: Stigmatomma; **Location:** country: Madagascar; stateProvince: Antananarivo; locality: Reg. Analamanga, St. Forestière Mandraka; verbatimElevation: 1285; decimalLatitude: -18.9183; decimalLongitude: 47.91687; georeferenceRemarks: ±100 m, coordinates obtained from GPS; **Event:** samplingProtocol: General collecting; eventDate: 03/25/2015; habitat: montane rainforest; fieldNumber: BLF37036; eventRemarks: ex soil; **Record Level:** institutionCode: CASC
- **Type status:** Other material. **Occurrence:** catalogNumber: casent0703653; recordedBy: B.L.Fisher, F.A. Esteves et al.; sex: 1w; preparations: pin; associatedMedia: http://www.antweb.org/specimen/casent0703653; **Taxon:** scientificName: Stigmatomma roahady; genus: Stigmatomma; **Location:** country: Madagascar; stateProvince: Antananarivo; locality: Reg. Analamanga, St. Forestière Mandraka; verbatimElevation: 1285; decimalLatitude: -18.9183; decimalLongitude: 47.91687; georeferenceRemarks: ±100 m, coordinates obtained from GPS; **Event:** samplingProtocol: General collecting; eventDate: 03/25/2015; habitat: montane rainforest; fieldNumber: BLF37036; eventRemarks: ex soil; **Record Level:** institutionCode: CASC
- **Type status:** Other material. **Occurrence:** catalogNumber: casent0703654; recordedBy: B.L.Fisher, F.A. Esteves et al.; sex: 1w; preparations: pin; associatedMedia: http://www.antweb.org/specimen/casent0703654; **Taxon:** scientificName: Stigmatomma roahady; genus: Stigmatomma; **Location:** country: Madagascar; stateProvince: Antananarivo; locality: Reg. Analamanga, St. Forestière Mandraka; verbatimElevation: 1285; decimalLatitude: -18.9183; decimalLongitude: 47.91687; georeferenceRemarks: ±100 m, coordinates obtained from GPS; **Event:** samplingProtocol: General collecting; eventDate: 03/25/2015; habitat: montane rainforest; fieldNumber: BLF37036; eventRemarks: ex soil; **Record Level:** institutionCode: CASC
- **Type status:** Other material. **Occurrence:** catalogNumber: casent0703655; recordedBy: B.L.Fisher, F.A. Esteves et al.; sex: 1w; preparations: pin; associatedMedia: http://www.antweb.org/specimen/casent0703655; **Taxon:** scientificName: Stigmatomma roahady; genus: Stigmatomma; **Location:** country: Madagascar; stateProvince: Antananarivo; locality: Reg. Analamanga, St. Forestière Mandraka; verbatimElevation: 1285; decimalLatitude: -18.9183; decimalLongitude: 47.91687; georeferenceRemarks: ±100 m, coordinates obtained from GPS; **Event:** samplingProtocol: General collecting; eventDate: 03/25/2015; habitat: montane rainforest; fieldNumber: BLF37036; eventRemarks: ex soil; **Record Level:** institutionCode: CASC
- **Type status:** Other material. **Occurrence:** catalogNumber: casent0703657; recordedBy: B.L.Fisher, F.A. Esteves et al.; sex: 1q; preparations: pin; associatedMedia: http://www.antweb.org/specimen/casent0703657; **Taxon:** scientificName: Stigmatomma roahady; genus: Stigmatomma; **Location:** country: Madagascar; stateProvince: Antananarivo; locality: Reg. Analamanga, St. Forestière Mandraka; verbatimElevation: 1285; decimalLatitude: -18.9183; decimalLongitude: 47.91687; georeferenceRemarks: ±100 m, coordinates obtained from GPS; **Event:** samplingProtocol: General collecting; eventDate: 03/25/2015; habitat: montane rainforest; fieldNumber: BLF37036; eventRemarks: on low vegetaion; **Record Level:** institutionCode: CASC
- **Type status:** Other material. **Occurrence:** catalogNumber: casent0002030; recordedBy: Fisher, Griswold et al.; sex: 1w; preparations: pin; associatedMedia: http://www.antweb.org/specimen/casent0002030; **Taxon:** scientificName: Stigmatomma roahady; genus: Stigmatomma; **Location:** country: Madagascar; stateProvince: Antsiranana; locality: Parc National Montagne d'Ambre, 12.2 km 211° SSW Joffreville; verbatimElevation: 1300; decimalLatitude: -12.59639; decimalLongitude: 49.1595; georeferenceRemarks: coordinates obtained from GPS; **Event:** samplingProtocol: General collecting; eventDate: 02/02/2001; habitat: montane rainforest; fieldNumber: BLF02849; eventRemarks: ex root mat, ground layer; **Record Level:** institutionCode: CASC
- **Type status:** Other material. **Occurrence:** catalogNumber: casent0002031; recordedBy: Fisher, Griswold et al.; sex: 1w; preparations: SEM mount; associatedMedia: http://www.antweb.org/specimen/casent0002031; **Taxon:** scientificName: Stigmatomma roahady; genus: Stigmatomma; **Location:** country: Madagascar; stateProvince: Antsiranana; locality: Parc National Montagne d'Ambre, 12.2 km 211° SSW Joffreville; verbatimElevation: 1300; decimalLatitude: -12.59639; decimalLongitude: 49.1595; georeferenceRemarks: coordinates obtained from GPS; **Event:** samplingProtocol: General collecting; eventDate: 02/02/2001; habitat: montane rainforest; fieldNumber: BLF02852; eventRemarks: ex root mat, ground layer; **Record Level:** institutionCode: CASC
- **Type status:** Other material. **Occurrence:** catalogNumber: casent0002078; recordedBy: Fisher, Griswold et al.; sex: 1w; preparations: SEM mount; associatedMedia: http://www.antweb.org/specimen/casent0002078; **Taxon:** scientificName: Stigmatomma roahady; genus: Stigmatomma; **Location:** country: Madagascar; stateProvince: Antsiranana; locality: Parc National Montagne d'Ambre, 12.2 km 211° SSW Joffreville; verbatimElevation: 1300; decimalLatitude: -12.59639; decimalLongitude: 49.1595; georeferenceRemarks: coordinates obtained from GPS; **Event:** samplingProtocol: General collecting; eventDate: 02/02/2001; habitat: montane rainforest; fieldNumber: BLF02847; eventRemarks: ex root mat, ground layer; **Record Level:** institutionCode: CASC
- **Type status:** Other material. **Occurrence:** catalogNumber: casent0002104; recordedBy: Fisher, Griswold et al.; sex: 2w; preparations: pin; associatedMedia: http://www.antweb.org/specimen/casent0002104; **Taxon:** scientificName: Stigmatomma roahady; genus: Stigmatomma; **Location:** country: Madagascar; stateProvince: Antsiranana; locality: Parc National Montagne d'Ambre, 12.2 km 211° SSW Joffreville; verbatimElevation: 1300; decimalLatitude: -12.59639; decimalLongitude: 49.1595; georeferenceRemarks: coordinates obtained from GPS; **Event:** samplingProtocol: General collecting; eventDate: 02/02/2001; habitat: montane rainforest; fieldNumber: BLF02848; eventRemarks: ex root mat, ground layer; **Record Level:** institutionCode: CASC
- **Type status:** Other material. **Occurrence:** catalogNumber: casent0002105; recordedBy: Fisher, Griswold et al.; sex: 2w; preparations: pin; associatedMedia: http://www.antweb.org/specimen/casent0002105; **Taxon:** scientificName: Stigmatomma roahady; genus: Stigmatomma; **Location:** country: Madagascar; stateProvince: Antsiranana; locality: Parc National Montagne d'Ambre, 12.2 km 211° SSW Joffreville; verbatimElevation: 1300; decimalLatitude: -12.59639; decimalLongitude: 49.1595; georeferenceRemarks: coordinates obtained from GPS; **Event:** samplingProtocol: General collecting; eventDate: 02/02/2001; habitat: montane rainforest; fieldNumber: BLF02848; eventRemarks: ex root mat, ground layer; **Record Level:** institutionCode: CASC
- **Type status:** Other material. **Occurrence:** catalogNumber: casent0002130; recordedBy: Fisher, Griswold et al.; sex: 3w; preparations: pin; associatedMedia: http://www.antweb.org/specimen/casent0002130; **Taxon:** scientificName: Stigmatomma roahady; genus: Stigmatomma; **Location:** country: Madagascar; stateProvince: Antananarivo; locality: 3 km 41° NE Andranomay, 11.5 km 147° SSE Anjozorobe; verbatimElevation: 1300; decimalLatitude: -18.47333; decimalLongitude: 47.96; georeferenceRemarks: coordinates obtained from GPS; **Event:** samplingProtocol: General collecting; eventDate: 12/05/2000; habitat: montane rainforest; fieldNumber: BLF02526; eventRemarks: ex rotten log; **Record Level:** institutionCode: CASC
- **Type status:** Other material. **Occurrence:** catalogNumber: casent0002131; recordedBy: Fisher, Griswold et al.; sex: 3w; preparations: pin; associatedMedia: http://www.antweb.org/specimen/casent0002131; **Taxon:** scientificName: Stigmatomma roahady; genus: Stigmatomma; **Location:** country: Madagascar; stateProvince: Antananarivo; locality: 3 km 41° NE Andranomay, 11.5 km 147° SSE Anjozorobe; verbatimElevation: 1300; decimalLatitude: -18.47333; decimalLongitude: 47.96; georeferenceRemarks: coordinates obtained from GPS; **Event:** samplingProtocol: General collecting; eventDate: 12/05/2000; habitat: montane rainforest; fieldNumber: BLF02526; eventRemarks: ex rotten log; **Record Level:** institutionCode: CASC
- **Type status:** Other material. **Occurrence:** catalogNumber: casent0002138; recordedBy: Fisher, Griswold et al.; sex: 3w; preparations: pin; associatedMedia: http://www.antweb.org/specimen/casent0002138; **Taxon:** scientificName: Stigmatomma roahady; genus: Stigmatomma; **Location:** country: Madagascar; stateProvince: Antananarivo; locality: 3 km 41° NE Andranomay, 11.5 km 147° SSE Anjozorobe; verbatimElevation: 1300; decimalLatitude: -18.47333; decimalLongitude: 47.96; georeferenceRemarks: coordinates obtained from GPS; **Event:** samplingProtocol: General collecting; eventDate: 12/05/2000; habitat: montane rainforest; fieldNumber: BLF02479; eventRemarks: ex rotten log; **Record Level:** institutionCode: CASC
- **Type status:** Other material. **Occurrence:** catalogNumber: casent0002139; recordedBy: Fisher, Griswold et al.; sex: 3w; preparations: pin; associatedMedia: http://www.antweb.org/specimen/casent0002139; **Taxon:** scientificName: Stigmatomma roahady; genus: Stigmatomma; **Location:** country: Madagascar; stateProvince: Antananarivo; locality: 3 km 41° NE Andranomay, 11.5 km 147° SSE Anjozorobe; verbatimElevation: 1300; decimalLatitude: -18.47333; decimalLongitude: 47.96; georeferenceRemarks: coordinates obtained from GPS; **Event:** samplingProtocol: General collecting; eventDate: 12/05/2000; habitat: montane rainforest; fieldNumber: BLF02479; eventRemarks: ex rotten log; **Record Level:** institutionCode: CASC
- **Type status:** Other material. **Occurrence:** catalogNumber: casent0002140; recordedBy: Fisher, Griswold et al.; sex: 3w; preparations: pin; associatedMedia: http://www.antweb.org/specimen/casent0002140; **Taxon:** scientificName: Stigmatomma roahady; genus: Stigmatomma; **Location:** country: Madagascar; stateProvince: Antananarivo; locality: 3 km 41° NE Andranomay, 11.5 km 147° SSE Anjozorobe; verbatimElevation: 1300; decimalLatitude: -18.47333; decimalLongitude: 47.96; georeferenceRemarks: coordinates obtained from GPS; **Event:** samplingProtocol: General collecting; eventDate: 12/05/2000; habitat: montane rainforest; fieldNumber: BLF02479; eventRemarks: ex rotten log; **Record Level:** institutionCode: CASC
- **Type status:** Other material. **Occurrence:** catalogNumber: casent0004317; recordedBy: Fisher, Griswold et al.; sex: 1w; preparations: pin; associatedMedia: http://www.antweb.org/specimen/casent0004317; **Taxon:** scientificName: Stigmatomma roahady; genus: Stigmatomma; **Location:** country: Madagascar; stateProvince: Antananarivo; locality: 3 km 41° NE Andranomay, 11.5 km 147° SSE Anjozorobe; verbatimElevation: 1300; decimalLatitude: -18.47333; decimalLongitude: 47.96; georeferenceRemarks: coordinates obtained from GPS; **Event:** samplingProtocol: General collecting; eventDate: 12/05/2000; habitat: montane rainforest; fieldNumber: BLF02519; eventRemarks: ex rotten tree stump; **Record Level:** institutionCode: CASC
- **Type status:** Other material. **Occurrence:** catalogNumber: casent0004318; recordedBy: Fisher, Griswold et al.; sex: 1w; preparations: pin; associatedMedia: http://www.antweb.org/specimen/casent0004318; **Taxon:** scientificName: Stigmatomma roahady; genus: Stigmatomma; **Location:** country: Madagascar; stateProvince: Antananarivo; locality: 3 km 41° NE Andranomay, 11.5 km 147° SSE Anjozorobe; verbatimElevation: 1300; decimalLatitude: -18.47333; decimalLongitude: 47.96; georeferenceRemarks: coordinates obtained from GPS; **Event:** samplingProtocol: General collecting; eventDate: 12/05/2000; habitat: montane rainforest; fieldNumber: BLF02519; eventRemarks: ex rotten tree stump; **Record Level:** institutionCode: CASC
- **Type status:** Other material. **Occurrence:** catalogNumber: casent0004319; recordedBy: Fisher, Griswold et al.; sex: 1w; preparations: pin; associatedMedia: http://www.antweb.org/specimen/casent0004319; **Taxon:** scientificName: Stigmatomma roahady; genus: Stigmatomma; **Location:** country: Madagascar; stateProvince: Antananarivo; locality: 3 km 41° NE Andranomay, 11.5 km 147° SSE Anjozorobe; verbatimElevation: 1300; decimalLatitude: -18.47333; decimalLongitude: 47.96; georeferenceRemarks: coordinates obtained from GPS; **Event:** samplingProtocol: General collecting; eventDate: 12/05/2000; habitat: montane rainforest; fieldNumber: BLF02507; eventRemarks: ex rotten log; **Record Level:** institutionCode: CASC
- **Type status:** Other material. **Occurrence:** catalogNumber: casent0004323; recordedBy: Fisher, Griswold et al.; sex: 1w; preparations: pin; associatedMedia: http://www.antweb.org/specimen/casent0004323; **Taxon:** scientificName: Stigmatomma roahady; genus: Stigmatomma; **Location:** country: Madagascar; stateProvince: Antananarivo; locality: 3 km 41° NE Andranomay, 11.5 km 147° SSE Anjozorobe; verbatimElevation: 1300; decimalLatitude: -18.47333; decimalLongitude: 47.96; georeferenceRemarks: coordinates obtained from GPS; **Event:** samplingProtocol: General collecting; eventDate: 12/05/2000; habitat: montane rainforest; fieldNumber: BLF02507; eventRemarks: ex rotten log; **Record Level:** institutionCode: CASC
- **Type status:** Other material. **Occurrence:** catalogNumber: casent0004324; recordedBy: Fisher, Griswold et al.; sex: 1w; preparations: pin; associatedMedia: http://www.antweb.org/specimen/casent0004324; **Taxon:** scientificName: Stigmatomma roahady; genus: Stigmatomma; **Location:** country: Madagascar; stateProvince: Antananarivo; locality: 3 km 41° NE Andranomay, 11.5 km 147° SSE Anjozorobe; verbatimElevation: 1300; decimalLatitude: -18.47333; decimalLongitude: 47.96; georeferenceRemarks: coordinates obtained from GPS; **Event:** samplingProtocol: search by hand; eventDate: 12/05/2000; habitat: montane rainforest; fieldNumber: BLF02425; eventRemarks: sifted litter (leaf mold, rotten wood); **Record Level:** institutionCode: CASC
- **Type status:** Other material. **Occurrence:** catalogNumber: casent0004325; recordedBy: Fisher, Griswold et al.; sex: 1w; preparations: pin; associatedMedia: http://www.antweb.org/specimen/casent0004325; **Taxon:** scientificName: Stigmatomma roahady; genus: Stigmatomma; **Location:** country: Madagascar; stateProvince: Antananarivo; locality: 3 km 41° NE Andranomay, 11.5 km 147° SSE Anjozorobe; verbatimElevation: 1300; decimalLatitude: -18.47333; decimalLongitude: 47.96; georeferenceRemarks: coordinates obtained from GPS; **Event:** samplingProtocol: General collecting; eventDate: 12/05/2000; habitat: montane rainforest; fieldNumber: BLF02526; eventRemarks: ex rotten log; **Record Level:** institutionCode: CASC
- **Type status:** Other material. **Occurrence:** catalogNumber: casent0004326; recordedBy: Fisher, Griswold et al.; sex: 1w; preparations: pin; associatedMedia: http://www.antweb.org/specimen/casent0004326; **Taxon:** scientificName: Stigmatomma roahady; genus: Stigmatomma; **Location:** country: Madagascar; stateProvince: Antananarivo; locality: 3 km 41° NE Andranomay, 11.5 km 147° SSE Anjozorobe; verbatimElevation: 1300; decimalLatitude: -18.47333; decimalLongitude: 47.96; georeferenceRemarks: coordinates obtained from GPS; **Event:** samplingProtocol: General collecting; eventDate: 12/05/2000; habitat: montane rainforest; fieldNumber: BLF02526; eventRemarks: ex rotten log; **Record Level:** institutionCode: CASC
- **Type status:** Other material. **Occurrence:** catalogNumber: casent0004327; recordedBy: Fisher, Griswold et al.; sex: 1w; preparations: pin; associatedMedia: http://www.antweb.org/specimen/casent0004327; **Taxon:** scientificName: Stigmatomma roahady; genus: Stigmatomma; **Location:** country: Madagascar; stateProvince: Antananarivo; locality: 3 km 41° NE Andranomay, 11.5 km 147° SSE Anjozorobe; verbatimElevation: 1300; decimalLatitude: -18.47333; decimalLongitude: 47.96; georeferenceRemarks: coordinates obtained from GPS; **Event:** samplingProtocol: General collecting; eventDate: 12/05/2000; habitat: montane rainforest; fieldNumber: BLF02530; eventRemarks: ex rotten log; **Record Level:** institutionCode: CASC
- **Type status:** Other material. **Occurrence:** catalogNumber: casent0004328; recordedBy: Fisher, Griswold et al.; sex: 1w; preparations: pin; associatedMedia: http://www.antweb.org/specimen/casent0004328; **Taxon:** scientificName: Stigmatomma roahady; genus: Stigmatomma; **Location:** country: Madagascar; stateProvince: Antananarivo; locality: 3 km 41° NE Andranomay, 11.5 km 147° SSE Anjozorobe; verbatimElevation: 1300; decimalLatitude: -18.47333; decimalLongitude: 47.96; georeferenceRemarks: coordinates obtained from GPS; **Event:** samplingProtocol: General collecting; eventDate: 12/05/2000; habitat: montane rainforest; fieldNumber: BLF02530; eventRemarks: ex rotten log; **Record Level:** institutionCode: CASC
- **Type status:** Other material. **Occurrence:** catalogNumber: casent0004332; recordedBy: Fisher, Griswold et al.; sex: 1w; preparations: pin; associatedMedia: http://www.antweb.org/specimen/casent0004332; **Taxon:** scientificName: Stigmatomma roahady; genus: Stigmatomma; **Location:** country: Madagascar; stateProvince: Antananarivo; locality: 3 km 41° NE Andranomay, 11.5 km 147° SSE Anjozorobe; verbatimElevation: 1300; decimalLatitude: -18.47333; decimalLongitude: 47.96; georeferenceRemarks: coordinates obtained from GPS; **Event:** samplingProtocol: General collecting; eventDate: 12/05/2000; habitat: montane rainforest; fieldNumber: BLF02524; eventRemarks: ex rotten log; **Record Level:** institutionCode: CASC
- **Type status:** Other material. **Occurrence:** catalogNumber: casent0004333; recordedBy: Fisher, Griswold et al.; sex: 1w; preparations: pin; associatedMedia: http://www.antweb.org/specimen/casent0004333; **Taxon:** scientificName: Stigmatomma roahady; genus: Stigmatomma; **Location:** country: Madagascar; stateProvince: Antananarivo; locality: 3 km 41° NE Andranomay, 11.5 km 147° SSE Anjozorobe; verbatimElevation: 1300; decimalLatitude: -18.47333; decimalLongitude: 47.96; georeferenceRemarks: coordinates obtained from GPS; **Event:** samplingProtocol: General collecting; eventDate: 12/05/2000; habitat: montane rainforest; fieldNumber: BLF02534; eventRemarks: ex rotten log; **Record Level:** institutionCode: CASC
- **Type status:** Other material. **Occurrence:** catalogNumber: casent0004334; recordedBy: Fisher, Griswold et al.; sex: 1w; preparations: pin; associatedMedia: http://www.antweb.org/specimen/casent0004334; **Taxon:** scientificName: Stigmatomma roahady; genus: Stigmatomma; **Location:** country: Madagascar; stateProvince: Antananarivo; locality: 3 km 41° NE Andranomay, 11.5 km 147° SSE Anjozorobe; verbatimElevation: 1300; decimalLatitude: -18.47333; decimalLongitude: 47.96; georeferenceRemarks: coordinates obtained from GPS; **Event:** samplingProtocol: General collecting; eventDate: 12/05/2000; habitat: montane rainforest; fieldNumber: BLF02534; eventRemarks: ex rotten log; **Record Level:** institutionCode: CASC
- **Type status:** Other material. **Occurrence:** catalogNumber: casent0004338; recordedBy: Fisher, Griswold et al.; sex: 1w; preparations: pin; associatedMedia: http://www.antweb.org/specimen/casent0004338; **Taxon:** scientificName: Stigmatomma roahady; genus: Stigmatomma; **Location:** country: Madagascar; stateProvince: Antananarivo; locality: 3 km 41° NE Andranomay, 11.5 km 147° SSE Anjozorobe; verbatimElevation: 1300; decimalLatitude: -18.47333; decimalLongitude: 47.96; georeferenceRemarks: coordinates obtained from GPS; **Event:** samplingProtocol: General collecting; eventDate: 12/05/2000; habitat: montane rainforest; fieldNumber: BLF02533; eventRemarks: ex rotten log; **Record Level:** institutionCode: CASC
- **Type status:** Other material. **Occurrence:** catalogNumber: casent0004339; recordedBy: Fisher, Griswold et al.; sex: 1w; preparations: SEM mount; associatedMedia: http://www.antweb.org/specimen/casent0004339; **Taxon:** scientificName: Stigmatomma roahady; genus: Stigmatomma; **Location:** country: Madagascar; stateProvince: Antananarivo; locality: 3 km 41° NE Andranomay, 11.5 km 147° SSE Anjozorobe; verbatimElevation: 1300; decimalLatitude: -18.47333; decimalLongitude: 47.96; georeferenceRemarks: coordinates obtained from GPS; **Event:** samplingProtocol: General collecting; eventDate: 12/05/2000; habitat: montane rainforest; fieldNumber: BLF02533; eventRemarks: ex rotten log; **Record Level:** institutionCode: CASC
- **Type status:** Other material. **Occurrence:** catalogNumber: casent0004341; recordedBy: Fisher, Griswold et al.; sex: 1w; preparations: pin; associatedMedia: http://www.antweb.org/specimen/casent0004341; **Taxon:** scientificName: Stigmatomma roahady; genus: Stigmatomma; **Location:** country: Madagascar; stateProvince: Antananarivo; locality: 3 km 41° NE Andranomay, 11.5 km 147° SSE Anjozorobe; verbatimElevation: 1300; decimalLatitude: -18.47333; decimalLongitude: 47.96; georeferenceRemarks: coordinates obtained from GPS; **Event:** samplingProtocol: General collecting; eventDate: 12/05/2000; habitat: montane rainforest; fieldNumber: BLF02535; eventRemarks: ex rotten log; **Record Level:** institutionCode: CASC
- **Type status:** Other material. **Occurrence:** catalogNumber: casent0004342; recordedBy: Fisher, Griswold et al.; sex: 1w; preparations: pin; associatedMedia: http://www.antweb.org/specimen/casent0004342; **Taxon:** scientificName: Stigmatomma roahady; genus: Stigmatomma; **Location:** country: Madagascar; stateProvince: Antananarivo; locality: 3 km 41° NE Andranomay, 11.5 km 147° SSE Anjozorobe; verbatimElevation: 1300; decimalLatitude: -18.47333; decimalLongitude: 47.96; georeferenceRemarks: coordinates obtained from GPS; **Event:** samplingProtocol: General collecting; eventDate: 12/05/2000; habitat: montane rainforest; fieldNumber: BLF02535; eventRemarks: ex rotten log; **Record Level:** institutionCode: CASC
- **Type status:** Other material. **Occurrence:** catalogNumber: casent0004344; recordedBy: Fisher, Griswold et al.; sex: 1w; preparations: pin; associatedMedia: http://www.antweb.org/specimen/casent0004344; **Taxon:** scientificName: Stigmatomma roahady; genus: Stigmatomma; **Location:** country: Madagascar; stateProvince: Antananarivo; locality: 3 km 41° NE Andranomay, 11.5 km 147° SSE Anjozorobe; verbatimElevation: 1300; decimalLatitude: -18.47333; decimalLongitude: 47.96; georeferenceRemarks: coordinates obtained from GPS; **Event:** samplingProtocol: General collecting; eventDate: 12/05/2000; habitat: montane rainforest; fieldNumber: BLF02529; eventRemarks: ex rotten log; **Record Level:** institutionCode: CASC
- **Type status:** Other material. **Occurrence:** catalogNumber: casent0004345; recordedBy: Fisher, Griswold et al.; sex: 1w; preparations: pin; associatedMedia: http://www.antweb.org/specimen/casent0004345; **Taxon:** scientificName: Stigmatomma roahady; genus: Stigmatomma; **Location:** country: Madagascar; stateProvince: Antananarivo; locality: 3 km 41° NE Andranomay, 11.5 km 147° SSE Anjozorobe; verbatimElevation: 1300; decimalLatitude: -18.47333; decimalLongitude: 47.96; georeferenceRemarks: coordinates obtained from GPS; **Event:** samplingProtocol: General collecting; eventDate: 12/05/2000; habitat: montane rainforest; fieldNumber: BLF02529; eventRemarks: ex rotten log; **Record Level:** institutionCode: CASC
- **Type status:** Other material. **Occurrence:** catalogNumber: casent0004349; recordedBy: Fisher, Griswold et al.; sex: 1w; preparations: pin; associatedMedia: http://www.antweb.org/specimen/casent0004349; **Taxon:** scientificName: Stigmatomma roahady; genus: Stigmatomma; **Location:** country: Madagascar; stateProvince: Antananarivo; locality: 3 km 41° NE Andranomay, 11.5 km 147° SSE Anjozorobe; verbatimElevation: 1300; decimalLatitude: -18.47333; decimalLongitude: 47.96; georeferenceRemarks: coordinates obtained from GPS; **Event:** samplingProtocol: General collecting; eventDate: 12/05/2000; habitat: montane rainforest; fieldNumber: BLF02531; eventRemarks: ex rotten log; **Record Level:** institutionCode: CASC
- **Type status:** Other material. **Occurrence:** catalogNumber: casent0004376; recordedBy: Fisher, Griswold et al.; sex: 1w; preparations: pin; associatedMedia: http://www.antweb.org/specimen/casent0004376; **Taxon:** scientificName: Stigmatomma roahady; genus: Stigmatomma; **Location:** country: Madagascar; stateProvince: Antananarivo; locality: 3 km 41° NE Andranomay, 11.5 km 147° SSE Anjozorobe; verbatimElevation: 1300; decimalLatitude: -18.47333; decimalLongitude: 47.96; georeferenceRemarks: coordinates obtained from GPS; **Event:** samplingProtocol: General collecting; eventDate: 12/05/2000; habitat: montane rainforest; fieldNumber: BLF02507; eventRemarks: ex rotten log; **Record Level:** institutionCode: CASC
- **Type status:** Other material. **Occurrence:** catalogNumber: casent0004377; recordedBy: Fisher, Griswold et al.; sex: 1w; preparations: pin; associatedMedia: http://www.antweb.org/specimen/casent0004377; **Taxon:** scientificName: Stigmatomma roahady; genus: Stigmatomma; **Location:** country: Madagascar; stateProvince: Antananarivo; locality: 3 km 41° NE Andranomay, 11.5 km 147° SSE Anjozorobe; verbatimElevation: 1300; decimalLatitude: -18.47333; decimalLongitude: 47.96; georeferenceRemarks: coordinates obtained from GPS; **Event:** samplingProtocol: General collecting; eventDate: 12/05/2000; habitat: montane rainforest; fieldNumber: BLF02534; eventRemarks: ex rotten log; **Record Level:** institutionCode: CASC
- **Type status:** Other material. **Occurrence:** catalogNumber: casent0318445; recordedBy: Fisher, Griswold et al.; sex: 1w; preparations: pin; associatedMedia: http://www.antweb.org/specimen/casent0318445; **Taxon:** scientificName: Stigmatomma roahady; genus: Stigmatomma; **Location:** country: Madagascar; stateProvince: Antsiranana; locality: Parc National Montagne d'Ambre, 12.2 km 211° SSW Joffreville; verbatimElevation: 1300; decimalLatitude: -12.59639; decimalLongitude: 49.1595; georeferenceRemarks: coordinates obtained from GPS; **Event:** samplingProtocol: General collecting; eventDate: 02/02/2001; habitat: montane rainforest; fieldNumber: BLF02848; eventRemarks: ex root mat, ground layer; **Record Level:** institutionCode: CASC
- **Type status:** Other material. **Occurrence:** catalogNumber: casent0410392; recordedBy: Fisher, Griswold et al.; sex: 1w; preparations: pin; associatedMedia: http://www.antweb.org/specimen/casent0410392; **Taxon:** scientificName: Stigmatomma roahady; genus: Stigmatomma; **Location:** country: Madagascar; stateProvince: Antananarivo; locality: 3 km 41° NE Andranomay, 11.5 km 147° SSE Anjozorobe; verbatimElevation: 1300; decimalLatitude: -18.47333; decimalLongitude: 47.96; georeferenceRemarks: coordinates obtained from GPS; **Event:** samplingProtocol: MW 50 sample transect, 5m; eventDate: 12/05/2000; habitat: montane rainforest; fieldNumber: BLF02378; eventRemarks: sifted litter (leaf mold, rotten wood); **Record Level:** institutionCode: CASC
- **Type status:** Other material. **Occurrence:** catalogNumber: casent0410393; recordedBy: Fisher, Griswold et al.; sex: 1w; preparations: pin; associatedMedia: http://www.antweb.org/specimen/casent0410393; **Taxon:** scientificName: Stigmatomma roahady; genus: Stigmatomma; **Location:** country: Madagascar; stateProvince: Antananarivo; locality: 3 km 41° NE Andranomay, 11.5 km 147° SSE Anjozorobe; verbatimElevation: 1300; decimalLatitude: -18.47333; decimalLongitude: 47.96; georeferenceRemarks: coordinates obtained from GPS; **Event:** samplingProtocol: MW 50 sample transect, 5m; eventDate: 12/05/2000; habitat: montane rainforest; fieldNumber: BLF02378; eventRemarks: sifted litter (leaf mold, rotten wood); **Record Level:** institutionCode: CASC
- **Type status:** Other material. **Occurrence:** catalogNumber: casent0410394; recordedBy: Fisher, Griswold et al.; sex: 1w; preparations: pin; associatedMedia: http://www.antweb.org/specimen/casent0410394; **Taxon:** scientificName: Stigmatomma roahady; genus: Stigmatomma; **Location:** country: Madagascar; stateProvince: Antananarivo; locality: 3 km 41° NE Andranomay, 11.5 km 147° SSE Anjozorobe; verbatimElevation: 1300; decimalLatitude: -18.47333; decimalLongitude: 47.96; georeferenceRemarks: coordinates obtained from GPS; **Event:** samplingProtocol: MW 50 sample transect, 5m; eventDate: 12/05/2000; habitat: montane rainforest; fieldNumber: BLF02378; eventRemarks: sifted litter (leaf mold, rotten wood); **Record Level:** institutionCode: CASC
- **Type status:** Other material. **Occurrence:** catalogNumber: casent0410395; recordedBy: Fisher, Griswold et al.; sex: 1w; preparations: pin; associatedMedia: http://www.antweb.org/specimen/casent0410395; **Taxon:** scientificName: Stigmatomma roahady; genus: Stigmatomma; **Location:** country: Madagascar; stateProvince: Antananarivo; locality: 3 km 41° NE Andranomay, 11.5 km 147° SSE Anjozorobe; verbatimElevation: 1300; decimalLatitude: -18.47333; decimalLongitude: 47.96; georeferenceRemarks: coordinates obtained from GPS; **Event:** samplingProtocol: MW 50 sample transect, 5m; eventDate: 12/05/2000; habitat: montane rainforest; fieldNumber: BLF02378; eventRemarks: sifted litter (leaf mold, rotten wood); **Record Level:** institutionCode: CASC
- **Type status:** Other material. **Occurrence:** catalogNumber: casent0410396; recordedBy: Fisher, Griswold et al.; sex: 1w; preparations: pin; associatedMedia: http://www.antweb.org/specimen/casent0410396; **Taxon:** scientificName: Stigmatomma roahady; genus: Stigmatomma; **Location:** country: Madagascar; stateProvince: Antananarivo; locality: 3 km 41° NE Andranomay, 11.5 km 147° SSE Anjozorobe; verbatimElevation: 1300; decimalLatitude: -18.47333; decimalLongitude: 47.96; georeferenceRemarks: coordinates obtained from GPS; **Event:** samplingProtocol: MW 50 sample transect, 5m; eventDate: 12/05/2000; habitat: montane rainforest; fieldNumber: BLF02378; eventRemarks: sifted litter (leaf mold, rotten wood); **Record Level:** institutionCode: CASC
- **Type status:** Other material. **Occurrence:** catalogNumber: casent0410397; recordedBy: Fisher, Griswold et al.; sex: 1w; preparations: pin; associatedMedia: http://www.antweb.org/specimen/casent0410397; **Taxon:** scientificName: Stigmatomma roahady; genus: Stigmatomma; **Location:** country: Madagascar; stateProvince: Antananarivo; locality: 3 km 41° NE Andranomay, 11.5 km 147° SSE Anjozorobe; verbatimElevation: 1300; decimalLatitude: -18.47333; decimalLongitude: 47.96; georeferenceRemarks: coordinates obtained from GPS; **Event:** samplingProtocol: MW 50 sample transect, 5m; eventDate: 12/05/2000; habitat: montane rainforest; fieldNumber: BLF02378; eventRemarks: sifted litter (leaf mold, rotten wood); **Record Level:** institutionCode: CASC
- **Type status:** Other material. **Occurrence:** catalogNumber: casent0410398; recordedBy: Fisher, Griswold et al.; sex: 1w; preparations: pin; associatedMedia: http://www.antweb.org/specimen/casent0410398; **Taxon:** scientificName: Stigmatomma roahady; genus: Stigmatomma; **Location:** country: Madagascar; stateProvince: Antananarivo; locality: 3 km 41° NE Andranomay, 11.5 km 147° SSE Anjozorobe; verbatimElevation: 1300; decimalLatitude: -18.47333; decimalLongitude: 47.96; georeferenceRemarks: coordinates obtained from GPS; **Event:** samplingProtocol: MW 50 sample transect, 5m; eventDate: 12/05/2000; habitat: montane rainforest; fieldNumber: BLF02378; eventRemarks: sifted litter (leaf mold, rotten wood); **Record Level:** institutionCode: CASC
- **Type status:** Other material. **Occurrence:** catalogNumber: casent0410399; recordedBy: Fisher, Griswold et al.; sex: 1w; preparations: pin; associatedMedia: http://www.antweb.org/specimen/casent0410399; **Taxon:** scientificName: Stigmatomma roahady; genus: Stigmatomma; **Location:** country: Madagascar; stateProvince: Antananarivo; locality: 3 km 41° NE Andranomay, 11.5 km 147° SSE Anjozorobe; verbatimElevation: 1300; decimalLatitude: -18.47333; decimalLongitude: 47.96; georeferenceRemarks: coordinates obtained from GPS; **Event:** samplingProtocol: MW 50 sample transect, 5m; eventDate: 12/05/2000; habitat: montane rainforest; fieldNumber: BLF02378; eventRemarks: sifted litter (leaf mold, rotten wood); **Record Level:** institutionCode: CASC
- **Type status:** Other material. **Occurrence:** catalogNumber: casent0410400; recordedBy: Fisher, Griswold et al.; sex: 1w; preparations: pin; associatedMedia: http://www.antweb.org/specimen/casent0410400; **Taxon:** scientificName: Stigmatomma roahady; genus: Stigmatomma; **Location:** country: Madagascar; stateProvince: Antananarivo; locality: 3 km 41° NE Andranomay, 11.5 km 147° SSE Anjozorobe; verbatimElevation: 1300; decimalLatitude: -18.47333; decimalLongitude: 47.96; georeferenceRemarks: coordinates obtained from GPS; **Event:** samplingProtocol: MW 50 sample transect, 5m; eventDate: 12/05/2000; habitat: montane rainforest; fieldNumber: BLF02378; eventRemarks: sifted litter (leaf mold, rotten wood); **Record Level:** institutionCode: CASC
- **Type status:** Other material. **Occurrence:** catalogNumber: casent0410402; recordedBy: Fisher, Griswold et al.; sex: 1w; preparations: pin; associatedMedia: http://www.antweb.org/specimen/casent0410402; **Taxon:** scientificName: Stigmatomma roahady; genus: Stigmatomma; **Location:** country: Madagascar; stateProvince: Antananarivo; locality: 3 km 41° NE Andranomay, 11.5 km 147° SSE Anjozorobe; verbatimElevation: 1300; decimalLatitude: -18.47333; decimalLongitude: 47.96; georeferenceRemarks: coordinates obtained from GPS; **Event:** samplingProtocol: MW 50 sample transect, 5m; eventDate: 12/05/2000; habitat: montane rainforest; fieldNumber: BLF02378; eventRemarks: sifted litter (leaf mold, rotten wood); **Record Level:** institutionCode: CASC
- **Type status:** Other material. **Occurrence:** catalogNumber: casent0410403; recordedBy: Fisher, Griswold et al.; sex: 1w; preparations: pin; associatedMedia: http://www.antweb.org/specimen/casent0410403; **Taxon:** scientificName: Stigmatomma roahady; genus: Stigmatomma; **Location:** country: Madagascar; stateProvince: Antananarivo; locality: 3 km 41° NE Andranomay, 11.5 km 147° SSE Anjozorobe; verbatimElevation: 1300; decimalLatitude: -18.47333; decimalLongitude: 47.96; georeferenceRemarks: coordinates obtained from GPS; **Event:** samplingProtocol: MW 50 sample transect, 5m; eventDate: 12/05/2000; habitat: montane rainforest; fieldNumber: BLF02378; eventRemarks: sifted litter (leaf mold, rotten wood); **Record Level:** institutionCode: CASC
- **Type status:** Other material. **Occurrence:** catalogNumber: casent0410404; recordedBy: Fisher, Griswold et al.; sex: 1w; preparations: pin; associatedMedia: http://www.antweb.org/specimen/casent0410404; **Taxon:** scientificName: Stigmatomma roahady; genus: Stigmatomma; **Location:** country: Madagascar; stateProvince: Antananarivo; locality: 3 km 41° NE Andranomay, 11.5 km 147° SSE Anjozorobe; verbatimElevation: 1300; decimalLatitude: -18.47333; decimalLongitude: 47.96; georeferenceRemarks: coordinates obtained from GPS; **Event:** samplingProtocol: MW 50 sample transect, 5m; eventDate: 12/05/2000; habitat: montane rainforest; fieldNumber: BLF02378; eventRemarks: sifted litter (leaf mold, rotten wood); **Record Level:** institutionCode: CASC
- **Type status:** Other material. **Occurrence:** catalogNumber: casent0410405; recordedBy: Fisher, Griswold et al.; sex: 1w; preparations: pin; associatedMedia: http://www.antweb.org/specimen/casent0410405; **Taxon:** scientificName: Stigmatomma roahady; genus: Stigmatomma; **Location:** country: Madagascar; stateProvince: Antananarivo; locality: 3 km 41° NE Andranomay, 11.5 km 147° SSE Anjozorobe; verbatimElevation: 1300; decimalLatitude: -18.47333; decimalLongitude: 47.96; georeferenceRemarks: coordinates obtained from GPS; **Event:** samplingProtocol: MW 50 sample transect, 5m; eventDate: 12/05/2000; habitat: montane rainforest; fieldNumber: BLF02378; eventRemarks: sifted litter (leaf mold, rotten wood); **Record Level:** institutionCode: CASC
- **Type status:** Other material. **Occurrence:** catalogNumber: casent0410406; recordedBy: Fisher, Griswold et al.; sex: 1w; preparations: pin; associatedMedia: http://www.antweb.org/specimen/casent0410406; **Taxon:** scientificName: Stigmatomma roahady; genus: Stigmatomma; **Location:** country: Madagascar; stateProvince: Antananarivo; locality: 3 km 41° NE Andranomay, 11.5 km 147° SSE Anjozorobe; verbatimElevation: 1300; decimalLatitude: -18.47333; decimalLongitude: 47.96; georeferenceRemarks: coordinates obtained from GPS; **Event:** samplingProtocol: MW 50 sample transect, 5m; eventDate: 12/05/2000; habitat: montane rainforest; fieldNumber: BLF02378; eventRemarks: sifted litter (leaf mold, rotten wood); **Record Level:** institutionCode: CASC
- **Type status:** Other material. **Occurrence:** catalogNumber: casent0410407; recordedBy: Fisher, Griswold et al.; sex: 1w; preparations: pin; associatedMedia: http://www.antweb.org/specimen/casent0410407; **Taxon:** scientificName: Stigmatomma roahady; genus: Stigmatomma; **Location:** country: Madagascar; stateProvince: Antananarivo; locality: 3 km 41° NE Andranomay, 11.5 km 147° SSE Anjozorobe; verbatimElevation: 1300; decimalLatitude: -18.47333; decimalLongitude: 47.96; georeferenceRemarks: coordinates obtained from GPS; **Event:** samplingProtocol: MW 50 sample transect, 5m; eventDate: 12/05/2000; habitat: montane rainforest; fieldNumber: BLF02378; eventRemarks: sifted litter (leaf mold, rotten wood); **Record Level:** institutionCode: CASC
- **Type status:** Other material. **Occurrence:** catalogNumber: casent0410408; recordedBy: Fisher, Griswold et al.; sex: 1w; preparations: pin; associatedMedia: http://www.antweb.org/specimen/casent0410408; **Taxon:** scientificName: Stigmatomma roahady; genus: Stigmatomma; **Location:** country: Madagascar; stateProvince: Antananarivo; locality: 3 km 41° NE Andranomay, 11.5 km 147° SSE Anjozorobe; verbatimElevation: 1300; decimalLatitude: -18.47333; decimalLongitude: 47.96; georeferenceRemarks: coordinates obtained from GPS; **Event:** samplingProtocol: MW 50 sample transect, 5m; eventDate: 12/05/2000; habitat: montane rainforest; fieldNumber: BLF02378; eventRemarks: sifted litter (leaf mold, rotten wood); **Record Level:** institutionCode: CASC
- **Type status:** Other material. **Occurrence:** catalogNumber: casent0410409; recordedBy: Fisher, Griswold et al.; sex: 1w; preparations: pin; associatedMedia: http://www.antweb.org/specimen/casent0410409; **Taxon:** scientificName: Stigmatomma roahady; genus: Stigmatomma; **Location:** country: Madagascar; stateProvince: Antananarivo; locality: 3 km 41° NE Andranomay, 11.5 km 147° SSE Anjozorobe; verbatimElevation: 1300; decimalLatitude: -18.47333; decimalLongitude: 47.96; georeferenceRemarks: coordinates obtained from GPS; **Event:** samplingProtocol: MW 50 sample transect, 5m; eventDate: 12/05/2000; habitat: montane rainforest; fieldNumber: BLF02378; eventRemarks: sifted litter (leaf mold, rotten wood); **Record Level:** institutionCode: CASC
- **Type status:** Other material. **Occurrence:** catalogNumber: casent0410410; recordedBy: Fisher, Griswold et al.; sex: 1w; preparations: pin; associatedMedia: http://www.antweb.org/specimen/casent0410410; **Taxon:** scientificName: Stigmatomma roahady; genus: Stigmatomma; **Location:** country: Madagascar; stateProvince: Antananarivo; locality: 3 km 41° NE Andranomay, 11.5 km 147° SSE Anjozorobe; verbatimElevation: 1300; decimalLatitude: -18.47333; decimalLongitude: 47.96; georeferenceRemarks: coordinates obtained from GPS; **Event:** samplingProtocol: MW 50 sample transect, 5m; eventDate: 12/05/2000; habitat: montane rainforest; fieldNumber: BLF02378; eventRemarks: sifted litter (leaf mold, rotten wood); **Record Level:** institutionCode: CASC
- **Type status:** Other material. **Occurrence:** catalogNumber: casent0410411; recordedBy: Fisher, Griswold et al.; sex: 1w; preparations: pin; associatedMedia: http://www.antweb.org/specimen/casent0410411; **Taxon:** scientificName: Stigmatomma roahady; genus: Stigmatomma; **Location:** country: Madagascar; stateProvince: Antananarivo; locality: 3 km 41° NE Andranomay, 11.5 km 147° SSE Anjozorobe; verbatimElevation: 1300; decimalLatitude: -18.47333; decimalLongitude: 47.96; georeferenceRemarks: coordinates obtained from GPS; **Event:** samplingProtocol: MW 50 sample transect, 5m; eventDate: 12/05/2000; habitat: montane rainforest; fieldNumber: BLF02378; eventRemarks: sifted litter (leaf mold, rotten wood); **Record Level:** institutionCode: CASC
- **Type status:** Other material. **Occurrence:** catalogNumber: casent0410414; recordedBy: Fisher, Griswold et al.; sex: 1w; preparations: pin; associatedMedia: http://www.antweb.org/specimen/casent0410414; **Taxon:** scientificName: Stigmatomma roahady; genus: Stigmatomma; **Location:** country: Madagascar; stateProvince: Antananarivo; locality: 3 km 41° NE Andranomay, 11.5 km 147° SSE Anjozorobe; verbatimElevation: 1300; decimalLatitude: -18.47333; decimalLongitude: 47.96; georeferenceRemarks: coordinates obtained from GPS; **Event:** samplingProtocol: pitfall trap, PF 50 traps, 11 cm dbh with water, soap, formalin, nonlinear placement; eventDate: 12/05/2000; habitat: montane rainforest; fieldNumber: BLF02371; **Record Level:** institutionCode: CASC
- **Type status:** Other material. **Occurrence:** catalogNumber: casent0410415; recordedBy: Fisher, Griswold et al.; sex: 1w (missing gaster); preparations: pin; associatedMedia: http://www.antweb.org/specimen/casent0410415; **Taxon:** scientificName: Stigmatomma roahady; genus: Stigmatomma; **Location:** country: Madagascar; stateProvince: Antananarivo; locality: 3 km 41° NE Andranomay, 11.5 km 147° SSE Anjozorobe; verbatimElevation: 1300; decimalLatitude: -18.47333; decimalLongitude: 47.96; georeferenceRemarks: coordinates obtained from GPS; **Event:** samplingProtocol: General collecting; eventDate: 12/05/2000; habitat: montane rainforest; fieldNumber: BLF02536; eventRemarks: ex rotten log; **Record Level:** institutionCode: CASC
- **Type status:** Other material. **Occurrence:** catalogNumber: casent0410416; recordedBy: Fisher, Griswold et al.; sex: 3w; preparations: pin; associatedMedia: http://www.antweb.org/specimen/casent0410416; **Taxon:** scientificName: Stigmatomma roahady; genus: Stigmatomma; **Location:** country: Madagascar; stateProvince: Antananarivo; locality: 3 km 41° NE Andranomay, 11.5 km 147° SSE Anjozorobe; verbatimElevation: 1300; decimalLatitude: -18.47333; decimalLongitude: 47.96; georeferenceRemarks: coordinates obtained from GPS; **Event:** samplingProtocol: General collecting; eventDate: 12/05/2000; habitat: montane rainforest; fieldNumber: BLF02536; eventRemarks: ex rotten log; **Record Level:** institutionCode: CASC
- **Type status:** Other material. **Occurrence:** catalogNumber: casent0410417; recordedBy: Fisher, Griswold et al.; sex: 3w; preparations: pin; associatedMedia: http://www.antweb.org/specimen/casent0410417; **Taxon:** scientificName: Stigmatomma roahady; genus: Stigmatomma; **Location:** country: Madagascar; stateProvince: Antananarivo; locality: 3 km 41° NE Andranomay, 11.5 km 147° SSE Anjozorobe; verbatimElevation: 1300; decimalLatitude: -18.47333; decimalLongitude: 47.96; georeferenceRemarks: coordinates obtained from GPS; **Event:** samplingProtocol: General collecting; eventDate: 12/05/2000; habitat: montane rainforest; fieldNumber: BLF02536; eventRemarks: ex rotten log; **Record Level:** institutionCode: CASC
- **Type status:** Other material. **Occurrence:** catalogNumber: casent0410418; recordedBy: Fisher, Griswold et al.; sex: 1w; preparations: pin; associatedMedia: http://www.antweb.org/specimen/casent0410418; **Taxon:** scientificName: Stigmatomma roahady; genus: Stigmatomma; **Location:** country: Madagascar; stateProvince: Antananarivo; locality: 3 km 41° NE Andranomay, 11.5 km 147° SSE Anjozorobe; verbatimElevation: 1300; decimalLatitude: -18.47333; decimalLongitude: 47.96; georeferenceRemarks: coordinates obtained from GPS; **Event:** samplingProtocol: MW 25 sample transect, 5m; eventDate: 12/05/2000; habitat: montane rainforest; fieldNumber: BLF02464; eventRemarks: sifted litter (leaf mold, rotten wood); **Record Level:** institutionCode: CASC
- **Type status:** Other material. **Occurrence:** catalogNumber: casent0410419; recordedBy: Fisher, Griswold et al.; sex: 1w; preparations: pin; associatedMedia: http://www.antweb.org/specimen/casent0410419; **Taxon:** scientificName: Stigmatomma roahady; genus: Stigmatomma; **Location:** country: Madagascar; stateProvince: Antananarivo; locality: 3 km 41° NE Andranomay, 11.5 km 147° SSE Anjozorobe; verbatimElevation: 1300; decimalLatitude: -18.47333; decimalLongitude: 47.96; georeferenceRemarks: coordinates obtained from GPS; **Event:** samplingProtocol: MW 25 sample transect, 5m; eventDate: 12/05/2000; habitat: montane rainforest; fieldNumber: BLF02464; eventRemarks: sifted litter (leaf mold, rotten wood); **Record Level:** institutionCode: CASC
- **Type status:** Other material. **Occurrence:** catalogNumber: casent0410420; recordedBy: Fisher, Griswold et al.; sex: 1w; preparations: pin; associatedMedia: http://www.antweb.org/specimen/casent0410420; **Taxon:** scientificName: Stigmatomma roahady; genus: Stigmatomma; **Location:** country: Madagascar; stateProvince: Antananarivo; locality: 3 km 41° NE Andranomay, 11.5 km 147° SSE Anjozorobe; verbatimElevation: 1300; decimalLatitude: -18.47333; decimalLongitude: 47.96; georeferenceRemarks: coordinates obtained from GPS; **Event:** samplingProtocol: MW 25 sample transect, 5m; eventDate: 12/05/2000; habitat: montane rainforest; fieldNumber: BLF02464; eventRemarks: sifted litter (leaf mold, rotten wood); **Record Level:** institutionCode: CASC
- **Type status:** Other material. **Occurrence:** catalogNumber: casent0410421; recordedBy: Fisher, Griswold et al.; sex: 1w; preparations: pin; associatedMedia: http://www.antweb.org/specimen/casent0410421; **Taxon:** scientificName: Stigmatomma roahady; genus: Stigmatomma; **Location:** country: Madagascar; stateProvince: Antananarivo; locality: 3 km 41° NE Andranomay, 11.5 km 147° SSE Anjozorobe; verbatimElevation: 1300; decimalLatitude: -18.47333; decimalLongitude: 47.96; georeferenceRemarks: coordinates obtained from GPS; **Event:** samplingProtocol: MW 25 sample transect, 5m; eventDate: 12/05/2000; habitat: montane rainforest; fieldNumber: BLF02464; eventRemarks: sifted litter (leaf mold, rotten wood); **Record Level:** institutionCode: CASC
- **Type status:** Other material. **Occurrence:** catalogNumber: casent0410422; recordedBy: Fisher, Griswold et al.; sex: 1w; preparations: pin; associatedMedia: http://www.antweb.org/specimen/casent0410422; **Taxon:** scientificName: Stigmatomma roahady; genus: Stigmatomma; **Location:** country: Madagascar; stateProvince: Antananarivo; locality: 3 km 41° NE Andranomay, 11.5 km 147° SSE Anjozorobe; verbatimElevation: 1300; decimalLatitude: -18.47333; decimalLongitude: 47.96; georeferenceRemarks: coordinates obtained from GPS; **Event:** samplingProtocol: MW 25 sample transect, 5m; eventDate: 12/05/2000; habitat: montane rainforest; fieldNumber: BLF02464; eventRemarks: sifted litter (leaf mold, rotten wood); **Record Level:** institutionCode: CASC
- **Type status:** Other material. **Occurrence:** catalogNumber: casent0410423; recordedBy: Fisher, Griswold et al.; sex: 1w; preparations: pin; associatedMedia: http://www.antweb.org/specimen/casent0410423; **Taxon:** scientificName: Stigmatomma roahady; genus: Stigmatomma; **Location:** country: Madagascar; stateProvince: Antananarivo; locality: 3 km 41° NE Andranomay, 11.5 km 147° SSE Anjozorobe; verbatimElevation: 1300; decimalLatitude: -18.47333; decimalLongitude: 47.96; georeferenceRemarks: coordinates obtained from GPS; **Event:** samplingProtocol: MW 25 sample transect, 5m; eventDate: 12/05/2000; habitat: montane rainforest; fieldNumber: BLF02464; eventRemarks: sifted litter (leaf mold, rotten wood); **Record Level:** institutionCode: CASC
- **Type status:** Other material. **Occurrence:** catalogNumber: casent0410424; recordedBy: Fisher, Griswold et al.; sex: 1w; preparations: pin; associatedMedia: http://www.antweb.org/specimen/casent0410424; **Taxon:** scientificName: Stigmatomma roahady; genus: Stigmatomma; **Location:** country: Madagascar; stateProvince: Antananarivo; locality: 3 km 41° NE Andranomay, 11.5 km 147° SSE Anjozorobe; verbatimElevation: 1300; decimalLatitude: -18.47333; decimalLongitude: 47.96; georeferenceRemarks: coordinates obtained from GPS; **Event:** samplingProtocol: MW 25 sample transect, 5m; eventDate: 12/05/2000; habitat: montane rainforest; fieldNumber: BLF02464; eventRemarks: sifted litter (leaf mold, rotten wood); **Record Level:** institutionCode: CASC
- **Type status:** Other material. **Occurrence:** catalogNumber: casent0410425; recordedBy: Fisher, Griswold et al.; sex: 1w; preparations: pin; associatedMedia: http://www.antweb.org/specimen/casent0410425; **Taxon:** scientificName: Stigmatomma roahady; genus: Stigmatomma; **Location:** country: Madagascar; stateProvince: Antananarivo; locality: 3 km 41° NE Andranomay, 11.5 km 147° SSE Anjozorobe; verbatimElevation: 1300; decimalLatitude: -18.47333; decimalLongitude: 47.96; georeferenceRemarks: coordinates obtained from GPS; **Event:** samplingProtocol: MW 25 sample transect, 5m; eventDate: 12/05/2000; habitat: montane rainforest; fieldNumber: BLF02464; eventRemarks: sifted litter (leaf mold, rotten wood); **Record Level:** institutionCode: CASC
- **Type status:** Other material. **Occurrence:** catalogNumber: casent0410426; recordedBy: Fisher, Griswold et al.; sex: 1w; preparations: pin; associatedMedia: http://www.antweb.org/specimen/casent0410426; **Taxon:** scientificName: Stigmatomma roahady; genus: Stigmatomma; **Location:** country: Madagascar; stateProvince: Antananarivo; locality: 3 km 41° NE Andranomay, 11.5 km 147° SSE Anjozorobe; verbatimElevation: 1300; decimalLatitude: -18.47333; decimalLongitude: 47.96; georeferenceRemarks: coordinates obtained from GPS; **Event:** samplingProtocol: MW 25 sample transect, 5m; eventDate: 12/05/2000; habitat: montane rainforest; fieldNumber: BLF02464; eventRemarks: sifted litter (leaf mold, rotten wood); **Record Level:** institutionCode: CASC
- **Type status:** Other material. **Occurrence:** catalogNumber: casent0410427; recordedBy: Fisher, Griswold et al.; sex: 1w; preparations: pin; associatedMedia: http://www.antweb.org/specimen/casent0410427; **Taxon:** scientificName: Stigmatomma roahady; genus: Stigmatomma; **Location:** country: Madagascar; stateProvince: Antananarivo; locality: 3 km 41° NE Andranomay, 11.5 km 147° SSE Anjozorobe; verbatimElevation: 1300; decimalLatitude: -18.47333; decimalLongitude: 47.96; georeferenceRemarks: coordinates obtained from GPS; **Event:** samplingProtocol: MW 25 sample transect, 5m; eventDate: 12/05/2000; habitat: montane rainforest; fieldNumber: BLF02464; eventRemarks: sifted litter (leaf mold, rotten wood); **Record Level:** institutionCode: CASC
- **Type status:** Other material. **Occurrence:** catalogNumber: casent0410428; recordedBy: Fisher, Griswold et al.; sex: 1w; preparations: pin; associatedMedia: http://www.antweb.org/specimen/casent0410428; **Taxon:** scientificName: Stigmatomma roahady; genus: Stigmatomma; **Location:** country: Madagascar; stateProvince: Antananarivo; locality: 3 km 41° NE Andranomay, 11.5 km 147° SSE Anjozorobe; verbatimElevation: 1300; decimalLatitude: -18.47333; decimalLongitude: 47.96; georeferenceRemarks: coordinates obtained from GPS; **Event:** samplingProtocol: MW 25 sample transect, 5m; eventDate: 12/05/2000; habitat: montane rainforest; fieldNumber: BLF02464; eventRemarks: sifted litter (leaf mold, rotten wood); **Record Level:** institutionCode: CASC
- **Type status:** Other material. **Occurrence:** catalogNumber: casent0410429; recordedBy: Fisher, Griswold et al.; sex: 1w; preparations: pin; associatedMedia: http://www.antweb.org/specimen/casent0410429; **Taxon:** scientificName: Stigmatomma roahady; genus: Stigmatomma; **Location:** country: Madagascar; stateProvince: Antananarivo; locality: 3 km 41° NE Andranomay, 11.5 km 147° SSE Anjozorobe; verbatimElevation: 1300; decimalLatitude: -18.47333; decimalLongitude: 47.96; georeferenceRemarks: coordinates obtained from GPS; **Event:** samplingProtocol: MW 25 sample transect, 5m; eventDate: 12/05/2000; habitat: montane rainforest; fieldNumber: BLF02464; eventRemarks: sifted litter (leaf mold, rotten wood); **Record Level:** institutionCode: CASC
- **Type status:** Other material. **Occurrence:** catalogNumber: casent0410430; recordedBy: Fisher, Griswold et al.; sex: 1w; preparations: pin; associatedMedia: http://www.antweb.org/specimen/casent0410430; **Taxon:** scientificName: Stigmatomma roahady; genus: Stigmatomma; **Location:** country: Madagascar; stateProvince: Antananarivo; locality: 3 km 41° NE Andranomay, 11.5 km 147° SSE Anjozorobe; verbatimElevation: 1300; decimalLatitude: -18.47333; decimalLongitude: 47.96; georeferenceRemarks: coordinates obtained from GPS; **Event:** samplingProtocol: MW 25 sample transect, 5m; eventDate: 12/05/2000; habitat: montane rainforest; fieldNumber: BLF02464; eventRemarks: sifted litter (leaf mold, rotten wood); **Record Level:** institutionCode: CASC
- **Type status:** Other material. **Occurrence:** catalogNumber: casent0410431; recordedBy: Fisher, Griswold et al.; sex: 1w; preparations: pin; associatedMedia: http://www.antweb.org/specimen/casent0410431; **Taxon:** scientificName: Stigmatomma roahady; genus: Stigmatomma; **Location:** country: Madagascar; stateProvince: Antananarivo; locality: 3 km 41° NE Andranomay, 11.5 km 147° SSE Anjozorobe; verbatimElevation: 1300; decimalLatitude: -18.47333; decimalLongitude: 47.96; georeferenceRemarks: coordinates obtained from GPS; **Event:** samplingProtocol: MW 25 sample transect, 5m; eventDate: 12/05/2000; habitat: montane rainforest; fieldNumber: BLF02464; eventRemarks: sifted litter (leaf mold, rotten wood); **Record Level:** institutionCode: CASC
- **Type status:** Other material. **Occurrence:** catalogNumber: casent0410432; recordedBy: Fisher, Griswold et al.; sex: 1w; preparations: pin; associatedMedia: http://www.antweb.org/specimen/casent0410432; **Taxon:** scientificName: Stigmatomma roahady; genus: Stigmatomma; **Location:** country: Madagascar; stateProvince: Antananarivo; locality: 3 km 41° NE Andranomay, 11.5 km 147° SSE Anjozorobe; verbatimElevation: 1300; decimalLatitude: -18.47333; decimalLongitude: 47.96; georeferenceRemarks: coordinates obtained from GPS; **Event:** samplingProtocol: MW 25 sample transect, 5m; eventDate: 12/05/2000; habitat: montane rainforest; fieldNumber: BLF02464; eventRemarks: sifted litter (leaf mold, rotten wood); **Record Level:** institutionCode: CASC
- **Type status:** Other material. **Occurrence:** catalogNumber: casent0410433; recordedBy: Fisher, Griswold et al.; sex: 1w; preparations: pin; associatedMedia: http://www.antweb.org/specimen/casent0410433; **Taxon:** scientificName: Stigmatomma roahady; genus: Stigmatomma; **Location:** country: Madagascar; stateProvince: Antananarivo; locality: 3 km 41° NE Andranomay, 11.5 km 147° SSE Anjozorobe; verbatimElevation: 1300; decimalLatitude: -18.47333; decimalLongitude: 47.96; georeferenceRemarks: coordinates obtained from GPS; **Event:** samplingProtocol: MW 25 sample transect, 5m; eventDate: 12/05/2000; habitat: montane rainforest; fieldNumber: BLF02464; eventRemarks: sifted litter (leaf mold, rotten wood); **Record Level:** institutionCode: CASC
- **Type status:** Other material. **Occurrence:** catalogNumber: casent0424112; recordedBy: Fisher, Griswold et al.; sex: 1w; preparations: pin; associatedMedia: http://www.antweb.org/specimen/casent0424112; **Taxon:** scientificName: Stigmatomma roahady; genus: Stigmatomma; **Location:** country: Madagascar; stateProvince: Antsiranana; locality: Parc National Montagne d'Ambre, 12.2 km 211° SSW Joffreville; verbatimElevation: 1300; decimalLatitude: -12.59639; decimalLongitude: 49.1595; georeferenceRemarks: coordinates obtained from GPS; **Event:** samplingProtocol: MW 50 sample transect, 5m; eventDate: 02/02/2001; habitat: montane rainforest; fieldNumber: BLF02808; eventRemarks: sifted litter (leaf mold, rotten wood); **Record Level:** institutionCode: CASC
- **Type status:** Other material. **Occurrence:** catalogNumber: casent0424113; recordedBy: Fisher, Griswold et al.; sex: 1w; preparations: pin; associatedMedia: http://www.antweb.org/specimen/casent0424113; **Taxon:** scientificName: Stigmatomma roahady; genus: Stigmatomma; **Location:** country: Madagascar; stateProvince: Antsiranana; locality: Parc National Montagne d'Ambre, 12.2 km 211° SSW Joffreville; verbatimElevation: 1300; decimalLatitude: -12.59639; decimalLongitude: 49.1595; georeferenceRemarks: coordinates obtained from GPS; **Event:** samplingProtocol: MW 50 sample transect, 5m; eventDate: 02/02/2001; habitat: montane rainforest; fieldNumber: BLF02808; eventRemarks: sifted litter (leaf mold, rotten wood); **Record Level:** institutionCode: CASC
- **Type status:** Other material. **Occurrence:** catalogNumber: casent0424114; recordedBy: Fisher, Griswold et al.; sex: 1w; preparations: pin; associatedMedia: http://www.antweb.org/specimen/casent0424114; **Taxon:** scientificName: Stigmatomma roahady; genus: Stigmatomma; **Location:** country: Madagascar; stateProvince: Antsiranana; locality: Parc National Montagne d'Ambre, 12.2 km 211° SSW Joffreville; verbatimElevation: 1300; decimalLatitude: -12.59639; decimalLongitude: 49.1595; georeferenceRemarks: coordinates obtained from GPS; **Event:** samplingProtocol: MW 50 sample transect, 5m; eventDate: 02/02/2001; habitat: montane rainforest; fieldNumber: BLF02808; eventRemarks: sifted litter (leaf mold, rotten wood); **Record Level:** institutionCode: CASC
- **Type status:** Other material. **Occurrence:** catalogNumber: casent0424115; recordedBy: Fisher, Griswold et al.; sex: 1w; preparations: pin; associatedMedia: http://www.antweb.org/specimen/casent0424115; **Taxon:** scientificName: Stigmatomma roahady; genus: Stigmatomma; **Location:** country: Madagascar; stateProvince: Antsiranana; locality: Parc National Montagne d'Ambre, 12.2 km 211° SSW Joffreville; verbatimElevation: 1300; decimalLatitude: -12.59639; decimalLongitude: 49.1595; georeferenceRemarks: coordinates obtained from GPS; **Event:** samplingProtocol: MW 50 sample transect, 5m; eventDate: 02/02/2001; habitat: montane rainforest; fieldNumber: BLF02808; eventRemarks: sifted litter (leaf mold, rotten wood); **Record Level:** institutionCode: CASC
- **Type status:** Other material. **Occurrence:** catalogNumber: casent0424116; recordedBy: Fisher, Griswold et al.; sex: 1w; preparations: pin; associatedMedia: http://www.antweb.org/specimen/casent0424116; **Taxon:** scientificName: Stigmatomma roahady; genus: Stigmatomma; **Location:** country: Madagascar; stateProvince: Antsiranana; locality: Parc National Montagne d'Ambre, 12.2 km 211° SSW Joffreville; verbatimElevation: 1300; decimalLatitude: -12.59639; decimalLongitude: 49.1595; georeferenceRemarks: coordinates obtained from GPS; **Event:** samplingProtocol: MW 50 sample transect, 5m; eventDate: 02/02/2001; habitat: montane rainforest; fieldNumber: BLF02808; eventRemarks: sifted litter (leaf mold, rotten wood); **Record Level:** institutionCode: CASC
- **Type status:** Other material. **Occurrence:** catalogNumber: casent0275852; recordedBy: B.L.Fisher et al.; sex: 1w; preparations: pin; associatedMedia: http://www.antweb.org/specimen/casent0275852; **Taxon:** scientificName: Stigmatomma roahady; genus: Stigmatomma; **Location:** country: Madagascar; stateProvince: Antananarivo; locality: Mandraka Park; verbatimElevation: 1360; decimalLatitude: -18.9019; decimalLongitude: 47.90786; georeferenceRemarks: ±200 m, coordinates obtained from GPS; **Event:** samplingProtocol: General collecting; eventDate: 03/11/2012; habitat: montane shrubland; fieldNumber: BLF28355; eventRemarks: ex litter; **Record Level:** institutionCode: CASC
- **Type status:** Other material. **Occurrence:** catalogNumber: casent0393035; recordedBy: B.L.Fisher et al.; sex: 1w; preparations: pin; associatedMedia: http://www.antweb.org/specimen/casent0393035; **Taxon:** scientificName: Stigmatomma roahady; genus: Stigmatomma; **Location:** country: Madagascar; stateProvince: Antananarivo; locality: Tsinjoarivo forest, Ankadivory; verbatimElevation: 1385; decimalLatitude: -19.71572; decimalLongitude: 47.82084; georeferenceRemarks: ±100 m; **Event:** eventDate: 01/13/2015; habitat: montane rainforest; fieldNumber: BLF35459; **Record Level:** institutionCode: CASC
- **Type status:** Other material. **Occurrence:** catalogNumber: casent0096761; recordedBy: CE Griswold & D Ubick; sex: 1w; preparations: pin; associatedMedia: http://www.antweb.org/specimen/casent0096761; **Taxon:** scientificName: Stigmatomma roahady; genus: Stigmatomma; **Location:** country: Madagascar; stateProvince: Fianarantsoa; locality: Parc Nationale Ranomafana: Talatakely; decimalLatitude: -21.24833; decimalLongitude: 47.42667; **Event:** eventDate: 04/19/1998; fieldNumber: ANTC2597; eventRemarks: ex leaf litter; **Record Level:** institutionCode: CASC
- **Type status:** Paratype. **Occurrence:** catalogNumber: casent0318918; recordedBy: B.L.Fisher; sex: 1w; preparations: pin; associatedMedia: http://www.antweb.org/specimen/casent0318918; **Taxon:** scientificName: Stigmatomma roahady; genus: Stigmatomma; **Location:** country: Madagascar; stateProvince: Toamasina; locality: Forêt Ambatovy, 14.3 km 57° Moramanga; verbatimElevation: 1075; decimalLatitude: -18.85083; decimalLongitude: 48.32; georeferenceRemarks: coordinates obtained from GPS; **Event:** samplingProtocol: General collecting; eventDate: 04/12/2005; habitat: montane rainforest; fieldNumber: BLF11961; eventRemarks: ex rotten log; **Record Level:** institutionCode: CASC
- **Type status:** Paratype. **Occurrence:** catalogNumber: casent0318422; recordedBy: B.L.Fisher; sex: 1w; preparations: pin; associatedMedia: http://www.antweb.org/specimen/casent0318919; **Taxon:** scientificName: Stigmatomma roahady; genus: Stigmatomma; **Location:** country: Madagascar; stateProvince: Toamasina; locality: Forêt Ambatovy, 14.3 km 57° Moramanga; verbatimElevation: 1075; decimalLatitude: -18.85083; decimalLongitude: 48.32; georeferenceRemarks: coordinates obtained from GPS; **Event:** samplingProtocol: General collecting; eventDate: 04/12/2005; habitat: montane rainforest; fieldNumber: BLF11961; eventRemarks: ex rotten log; **Record Level:** institutionCode: CASC
- **Type status:** Paratype. **Occurrence:** catalogNumber: casent0318917; recordedBy: B.L.Fisher; sex: 1w; preparations: pin; associatedMedia: http://www.antweb.org/specimen/casent0318917; **Taxon:** scientificName: Stigmatomma roahady; genus: Stigmatomma; **Location:** country: Madagascar; stateProvince: Toamasina; locality: Forêt Ambatovy, 14.3 km 57° Moramanga; verbatimElevation: 1075; decimalLatitude: -18.85083; decimalLongitude: 48.32; georeferenceRemarks: coordinates obtained from GPS; **Event:** samplingProtocol: General collecting; eventDate: 04/12/2005; habitat: montane rainforest; fieldNumber: BLF11961; eventRemarks: ex rotten log; **Record Level:** institutionCode: MCZC
- **Type status:** Paratype. **Occurrence:** catalogNumber: casent0318916; recordedBy: B.L.Fisher; sex: 1w; preparations: pin; associatedMedia: http://www.antweb.org/specimen/casent0318916; **Taxon:** scientificName: Stigmatomma roahady; genus: Stigmatomma; **Location:** country: Madagascar; stateProvince: Toamasina; locality: Forêt Ambatovy, 14.3 km 57° Moramanga; verbatimElevation: 1075; decimalLatitude: -18.85083; decimalLongitude: 48.32; georeferenceRemarks: coordinates obtained from GPS; **Event:** samplingProtocol: General collecting; eventDate: 04/12/2005; habitat: montane rainforest; fieldNumber: BLF11961; eventRemarks: ex rotten log; **Record Level:** institutionCode: MZSP
- **Type status:** Other material. **Occurrence:** catalogNumber: casent0042897; recordedBy: B.L.Fisher; sex: 1w; preparations: pin; associatedMedia: http://www.antweb.org/specimen/casent0042897; **Taxon:** scientificName: Stigmatomma roahady; **Location:** country: Madagascar; stateProvince: Antsiranana; locality: Forêt de Binara, 9.4km 235° SW Daraina; verbatimElevation: 1100; decimalLatitude: -13.26333; decimalLongitude: 49.6; georeferenceRemarks: coordinates obtained from GPS; **Event:** samplingProtocol: MW 25 sample transect, 5m; eventDate: 12/05/2003; habitat: montane rainforest; fieldNumber: BLF09800; eventRemarks: sifted litter (leaf mold, rotten wood); **Record Level:** institutionCode: CASC
- **Type status:** Other material. **Occurrence:** catalogNumber: casent0042894; recordedBy: B.L.Fisher; sex: 1w; preparations: pin; associatedMedia: http://www.antweb.org/specimen/casent0042894; **Taxon:** scientificName: Stigmatomma roahady; genus: Stigmatomma; **Location:** country: Madagascar; stateProvince: Antsiranana; locality: Forêt de Binara, 9.4km 235° SW Daraina; verbatimElevation: 1100; decimalLatitude: -13.26333; decimalLongitude: 49.6; georeferenceRemarks: coordinates obtained from GPS; **Event:** samplingProtocol: MW 25 sample transect, 5m; eventDate: 12/05/2003; habitat: montane rainforest; fieldNumber: BLF09800; eventRemarks: sifted litter (leaf mold, rotten wood); **Record Level:** institutionCode: CASC

#### Description

Worker (Fig. 73; holotype values within parentheses): **HL**: 1.32-1.60 (1.57); **HW**: 1.23-1.46 (1.46); **HW2**: 1.05-1.24 (1.24); **SL**: 0.72-0.88 (0.85); **ML**: 0.98-1.12 (1.07); **WL**: 1.68-2.03 (1.95); **PPW**: 0.73-0.89 (0.87); **PtL**: 0.73-0.87 (0.87); **PtW**: 0.76-0.92 (0.92); **CI**: 87-93 (93); **SI**: 48-60 (54); **MI**: 68-74 (68); **PtI**: 92-100 (95). 

*Head*:

Five Mandibular baso-masticatory margin skirted dorsally by row of filiform setae; ventrally, by spatular setae (Fig. 74a). Mandibular dentition arrangement, from base to apex: single larger tooth; much smaller single tooth (absent or much reduced in some specimens); five to six pairs of teeth (each teeth pair with same dimensions, fused basally; pairs of teeth similar in length along mandible's basoapical axis); single preapical tooth; apical tooth (Fig. 74a​). Anterior clypeal margin with seven to ten tubercle-like cuticular processes, arranged in a single row, anteriorly armed with asymmetrical, mucronate, dentiform seta (Fig. 74a​). Lateral-most clypeal cuticular process with row of smaller conical setae anterolaterally, continuing laterally along clypeal anterior margin, arising from flat cuticle (Fig. 74a​). Median clypeal cuticular processes with almost same length of associated dentiform setae. Pair of long, filiform setae on clypeal anterior margin bordering the central-most cuticular processes (Fig. 74a​). Median area of clypeus extending posteriorly between antennal sockets; frontoclypeal sulcus round (Fig. 74b). Supraclypeal area as oval concavity (Fig. 74b​). Twelve antennomeres. Genal teeth present (Fig. 74b​). Widest diameter of compound eyes: four to five ommatidia (Fig. 74c). Palpal formula: 4:3 (four maxillary, three labial; Fig. 74d​).

*Mesosoma*:

In dorsal view, lateral margins of mesonotum continuous with posterior remainder of mesosoma, or expanded laterally (Fig. 75a). Metanotal suture well developed or absent (Fig. 75a). Sulcus dividing mesepisternum into anepisternum and katepisternum (Fig. 75b). Metathoracic spiracle slit-like, anterior margin somewhat swollen (Fig. 75b). Propodeal spiracle round, with swollen margin (Fig. 75b). Propodeal declivitous face slightly concave (Fig. 75a).

*Legs*:

Basoventral half of calcar of strigil lamellar (Fig. 76a, b). Anterior face of calcar of strigil with strap-like microtrichia (Fig. 76a); posterior face with lanceolate microtrichia (Fig. 76b). Multiple paddle-like setae on anteroventral face of protibial apex, next to strigil's calcarcalcar of strigil. Multiple paddle-like setae on anterior face of probasitarsus; stout setae on posterior face, parallel to comb of strigil. Two mesotibial spurs (Fig. 76c): simple anterior spur, with lanceolate microtrichia; posterior spur somewhat falcate (with rounded baso-ventral projection), and with lanceolate microtrichia. Ventral margin of posterior mesotibial spur with digitiform cuticular projections, restricted to the most basal region or along entire ventral margin. Groove-like longitudinal sulcus on anterodorsal face of mesobasitarsus (Fig. 76d). Stout filiform setae along inner face of mesobasitarsus. Two metatibial spurs (Fig. 77a): simple anterior spur, with lanceolate microtrichia; posterior spur pectinate. Anterior face of posterior metatibial spur with sparse lanceolate microtrichia (Fig. 77a​); posterior face mostly glabrous (Fig. 77b). Groove-like longitudinal sulcus on anterior face of metabasitarsus (Fig. 77c). Few stout paddle-like setae on baso-inner face of metabasitarsus; stout filiform setae along remainder of inner face. Arolium on pro-, meso-, and metapretarsus.

*Metasoma*:

Petiole sessile (Fig. 78a). Ventroanterior margin of petiolar tergite anterior dorso-latero-ventral carina (Ward 1990) much shorter than anterior margin of subpetiolar process, in lateral view (Fig. 78a​). Ventral margin of subpetiolar process running posteriorly in a continuous line (Fig. 78a​). Presence of fenestra on lateral face of subpetiolar process (Fig. 78a​). Petiolar proprioceptor zone a large, round concavity with numerous sensilla (Fig. 78b). Prora present (Fig. 78a​). Scrobiculate sulcus between pretergite and postergite of abdominal segment III and presclerites and postsclerites of abdominal segment IV. Absence of stout setae on hypopygium (Fig. 78c).

**Figure 79.**
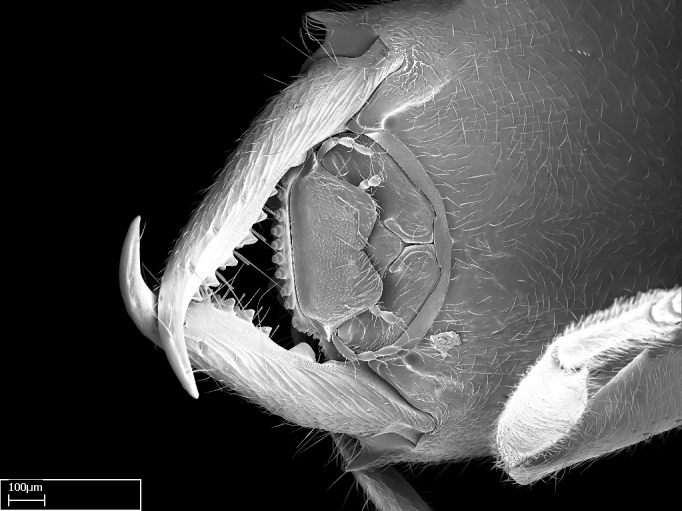
*Stigmatomma roahady* ​**sp. n.** worker (CASENT0004339): ventral view of the mandibles, mouth parts, and anterior part of the head. Image by F. A. Esteves; available at AntWeb.org.

*Sculpture*:

Mandibular dorsal face mostly costate, except for smooth apical portion (Fig. 74a). Median clypeal area costate. Supraclypeal area mostly smooth (Fig. 74b). First third of the head, in dorsal view, costulate-punctate/foveolate, grading to punctate/foveolate posteriorly (Fig. 74b). Area posterior to tentorial pit carinate concentrically (Fig. 74b). Labrum mostly imbricate (Fig. 79). Pronotum and dorsal face of remainder mesosoma scarcely punctate/foveolate (Fig. 75a). Anepisternum mostly smooth; katepisternum costulate dorsally, grading into rugulose to strigate ventrally (Fig. 75b). Metapleuron costate dorsoposteriorly and posteriorly (Fig. 75b). Lateral face propodeum slightly punctate or smooth (Fig. 75b); declivitous face punctate (Fig. 75a). Petiolar tergite mostly punctate/foveolate (Fig. 78a); laterotergite mostly smooth (Fig. 78b); poststernite imbricate/alveolate (Fig. 78b). Gaster mostly smooth; tergites of abdominal segments III and IV slightly punctate/foveolate; tergites of abdominal segments VI and VII slightly imbricate.

*Pilosity and color*:

Suberect to decumbent pilosity on head. Erect to suberect pilosity on dorsal face of mesosoma, petiolar tergite, anterior half of petiolar poststernite, and abdominal segments III, IV, and V. Longer pilosity on abdominal segment VII. Body color orange-brown to black; gaster orange to black with slightly lighter apex; appendages yellow-brown to orange.

##### Comments on character variation

A north-south geographical pattern is apparent considering: (1) number of tooth pairs on the mandible; (2) degree of mesonotum expansion and metanotal suture development; (3) amount of cuticular projections on the ventral margin of posterior mesotibial spur; and (4) head sculpture and body color.

**1**. In *Stigmatomma roahady*, the mandible may bear five to six pairs of teeth. The majority of specimens collected in the north of Madagascar possesses five pairs of teeth on the mandibles, and most of the specimens collected on the rest of the island have six pairs of teeth.

However, there are exceptions to this geographical pattern; some specimens collected in the southeast, center, and northeast of the island exhibit five pairs of teeth in their mandibles (e.g. CASENT0275421, plus all specimens collected at Andohahela National Park, southeast Madagascar). It is noteworthy that one of two specimens collected in the same Winkler sample has five teeth on each mandible, while the other has six (CASENT0227582; specimens from Ranomafana National Park, Fianarantsoa Province). Also, there are specimens presenting different numbers of tooth pairs on each of their mandibles: a specimen from Zahamena National Park, Toamasina Province, has five pairs of teeth on one mandible and six on another (CASENT0150904), and a specimen from Rés. Anjanaharibe-Sud, Antsiranana Province, has five pairs of teeth on one mandible, and four on the other (CASENT0746594).

**2**. Specimens collected in Northern Madagascar present a more expanded mesonotum and a more developed metanotal suture than specimens from the rest of the island, with some exceptions: in a few specimens the mesonotum is not so expanded (CASENT0042897, CASENT0476591, CASENT0746592), and one specimen examined does not have a well-developed metanotal suture (CASENT0746592).

Further, some specimens collected outside that range show an intermediate level of mesonotum expansion (*e.g*., CASENT0454523, CASENT0050358, CASENT0275421, CASENT0746590) and developmentof metanotal suture (*e.g*., CASENT0454523, CASENT0050367, CASENT0050358, CASENT0275421, CASENT0300576).

**3**. Regarding the cuticular projections on the ventral margin of the posterior mesotibial spur, more than half of specimens from the north of Madagascar bear many ventral digitiform cuticular projections; in some specimens, the posterior mesotibial spur appears pectinate (*e.g*., CASENT0002104).

However, in the north of the island, some specimens possess a posterior mesotibial spur bearing few ventral cuticular projections at its basal-most part (*e.g*., CASENT0042844), and has no projections at all on a few specimens (*e.g*., CASENT0042896). This character varies among specimens from the same colony (*e.g*., CASENT0746594), and within the same specimen (on specimen CASENT0133735 the posterior mesotibial spur of one leg bears few cuticular projections on the basoventral margin, while the spur of the other leg might be considered pectinate).

The majority of specimens from the south and central regions of the island present a posterior mesotibial spur bearing few or no cuticular projections on the basoventral margin, with the exception of one specimen from Foret Ambatovy, Toamasina Province (CASENT0067261).

**4**. A north-south geographical pattern is also apparent when head sculpture and body color are considered.

Head sculpture is more impressed and color is darker on specimens collected in the north of Madagascar. But again, there are exceptions to this pattern: in the north, some specimens present a head that is smoother than other specimens from the same region (CASENT0746595; CASENT0746592); in the center-south, specimen CASENT0275421 has a more sculptured head than the other specimens collected in the same area. The color of specimens from central and south Madagascar is extremely variable, ranging from blackish with orange gaster and yellow-brown appendages (*e.g*., CASENT0058814) to orange body with yellow-brown appendages (*e.g*., CASENT0275089).

Other characters differ regardless of sample location, and vary in specimens from the same locality, or even from the same colony: body size, position and size of the smaller basal single tooth of the mandibles, and number of cuticular processes bearing dentiform setae on the clypeal anterior margin.

##### Other castes

Gyne (Fig. 80); alate when virgin: Very similar to the worker caste but for the greater body length, larger compound eyes, presence of ocelli (Fig. 80a), and differences on the mesosoma due to the presence of wings. Parapsidal lines on the mesoscutum; scuto-scutellar suture narrow, without apparent sculpture on its midsection, but scrobiculate on its apexes (Fig. 81a). Mesepisternum divided into anepisternum and katepisternum; mesepimeral lobe distinct, but not well developed; metapleuron divided into upper and lower sections; upper metapleuron separated from propodeum by narrow scrobiculate sulcus; lower metapleuron separated from the propodeum by a carina, followed dorsally by a strigate sulcus (Fig. 81b). Forewing (Fig. 81c): pterostigma well developed; Rs.f2-3 present; Rs.f5 present and reaching R.f3; adventitious vein leaving Rs.f5 apically; 1r-rs absent; 2r-rs, M.f4, 2rs-m, Cu.f2, 1m-cu, and A.f2 present; cu-a intercepting M+Cu anteriorly to the separation point between Cu.f1and M.f1. Hindwing (Fig. 81d): C indistinct; R, Rs.f2, 1rs-m, and M.f2; adventitious crossvein at M+Cu, running towards Sc+R+Rs; Cu, cu-a, and A.f2 present.

Intercaste between gynes and workers (Fig. 82); wingless: Very similar to the worker caste but for the presence of of compound eyes and occelli (Fig. 82a), much larger mesonotum, and more developed metanotal suture (Fig. 82c).

Male (Fig. 83); alate: Mandibles falcate, with sharp, single apical tooth (Fig. 83a). Anterior clypeal margin armed with dentiform setae (Fig. 83a​). Compound eyes with sparse longer setae present among each ommatidium (Fig. 84a). Palpal formula 4:3 (Fig. 85a). Notauli distinct and scrobiculate; parapsidal lines present; scuto-scutellar suture scrobiculate (Fig. 84b). Mesepisternum partially divided or not divided into anepisternum and katepisternum; posterior oblique sulcus short, not well developed; mesepimeral lobe well developed; metapleuron divided into upper and lower sections by a pit; scrobiculate sulcus separating upper metapleuron from propodeum; slightly strigate sulcus separating lower metapleuron from propodeum (Fig. 84c). Forewing (Fig. 86a​): pterostigma well developed; Rs.f2-3 present; Rs.f5 present and reaching R.f3; 1r-rs absent; 2r-rs, M.f4, 2rs-m, Cu.f2, 1m-cu, and A.f2 present; cu-a intercepting M+Cu anteriorly to the separation point between M.f1 and Cu.f1. Hindwing (Fig. 86b): C slightly distinct; R, Rs.f2, 1rs-m, M.f2, Cu, cu-a, and A.f2 present. Pygostyles present (Fig. 84d). Posterior margin of abdominal segment IX convex (Fig. 85d). Paramere not visibly divided into telomere and basimere. Digitus tongue-plier-shaped: presence of a comparatively enlarged, but thin basal projection on the digitus; cuspis almost as long as digitus (Fig. 85c). Entire ventral margin of the penisvalva serrate; dorsal portion of the penisvalva not sclerotized (Fig. 85b).

##### Specimens used in prior studies

*Stigmatomma roahady* was referenced as *Amblyopone* sp. Ma-01 (specimen CASENT0500015) and as *Amblyopone* sp. (CASENT0500385) in Ouellette et al. (2006), as *Stigmatomma* MG01 (CASENT0135098) and as *Stigmatomma* MG05 (CASENT0042894) in Ward and Fisher (2016), and as *Stigmatomma* MG01 (CASENT0227519) in Yoshimura and Fisher (2012b).

#### Diagnosis

Worker

With characters of the *tsyhady* species-group and the *tsyhady* species-complex as described above, and the following characters (asterisks flag unique characters within the genus in the Malagasy bioregion):

1. Integument orange-brown to black; large-sized ant (HL: 1.32-1.60, WL: 1.68-2.03; Fig. 73).
2. Pairs of teeth on baso-masticatory margin of mandible are the same length along the basoapical axis (Fig. 74a).
3. Spatular setae ventrally skirting baso-masticatory margin of mandible (Fig. 74a).
4. Dorsal face of the head costulate-punctate/foveolate anteriorly, grading to punctate/foveolate posteriorly (Fig. 74b).
5. Palpal formula 4:3 (Fig. 74d).
6. Pronotum and the dorsal face of remainder mesosoma punctate/foveolate (Fig. 75a); lateral face of remainder mesosoma mostly slightly punctate or smooth (Fig. 75b); propodeal declivitous face punctate (Fig. 75a).
7. Mesepisternum divided into anepisternum and katepisternum (Fig. 75b).
8. Basoventral half of calcar of strigil lamellar (Fig. 76a, b).
9. Anterior face of calcar of strigil with strap-like microtrichia (Fig. 76a).
10. Two mesotibial spurs (Fig. 76c).
11. Groove-like longitudinal sulcus present on the anterodorsal face of mesobasitarsus (Fig. 76d).
12. Two metatibial spurs (Fig. 77a).
13. Anterior face of posterior metatibial spur with sparsed lanceolate microtrichia (Fig. 77a).
14. Few stout paddle-like setae present on the baso-inner face of metabasitarsus.
* Groove-like longitudinal sulcus present on the anterior face of metabasitarsus (Fig. 77c).
16. Ventral margin of petiolar poststernite runs continuously posteriorly, not forming a fin (Fig. 78a).

*Stigmatomma roahady* ressembles *S. irayhady, S. tsyhady* and *S. liebe* by the presence of genal teeth, palpal formula, two mesotibial spurs, shape of microtrichia on posterior face of posterior metatibial spur, presence of fenestra on the subpetiolar process, and absence of stout setae on the apex of hypopygium. While color and size make it even more similar to *S. irayhady* and *S. tsyhady*, *S. roahady* is unique in having a longitudinal sulcus on the anterior face of its metabasitarsus.

This species occurs in sympatry with *S. irayhady* in two localities (nearby Andranomay, and at the Binara Forest), with *S. tsyhady* in twelve localities (Ambatovy Forest, Analamay Forest, Vevembe Forest, Mantadia National Park, Marojejy National Park, Ranomafana National Park, Zahamena National Park, Andohahela National Park, Andringitra Reserve, Ivohibe special reserve, Anosyenne Mountains, and at the Binara Forest), and with *S. liebe* in four localities (Andohahela National Park, Andringitra Reserve, Ivohibe Special Reserve, and at the Anosyenne Mountains).

#### Etymology

The name is formed by the junction of the Malagasy cardinal number roa, meaning two, and the Malagasy name hady, meaning sulcus, ditch, and trench. The name *roahady* is a reference to the presence of a longitudinal sulcus on the anterior face of the mesobasitarsus and the anterior face of the metabasitarsus. The presence of a groove-like sulcus on the anterior face of the metabasitarsus is unique among *Stigmatomma* species in the Malagasy bioregion.

#### Distribution

**Figure 87.**
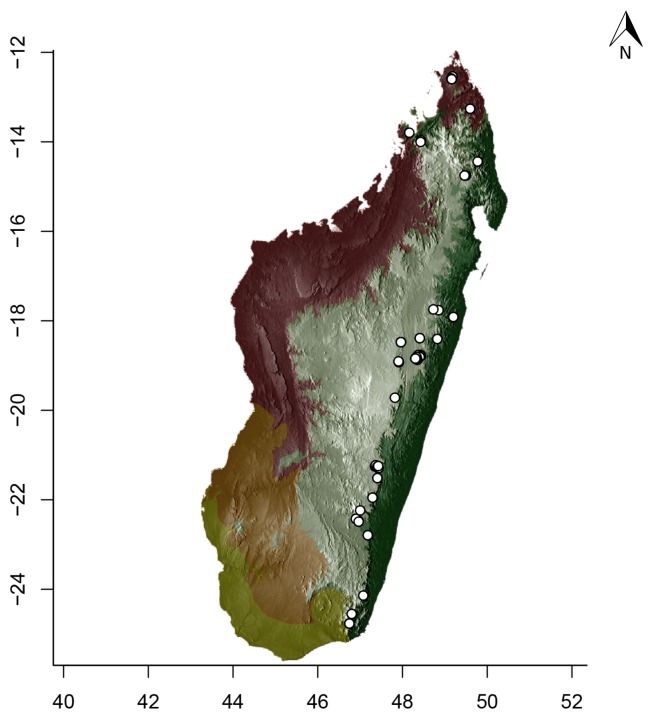
Distribution map of *Stigmatomma roahady* ​**sp. n.** in the Malagasy bioregion. Collection localities are mapped over the outlines of five simplified ecoregion zones of Madagascar: humid forests (dark green), subhumid forests (light green), dry deciduous forests (brown), succulent woodlands (orange), and spiny thickets (yellow).

*Stigmatomma roahady* was collected primarily in rainforest and montane rainforest habitats (just one collection occurred in montane shrubland habitat). All records fall within the limits of the humid forests ecoregions, in the eastern biome of Madagascar (*sensu* Burgess et al. 2004; Fig. 87). Specimens were distributed at elevations ranging from 400 to 1400 m; however, of a total of 99 collection events, just nine were recorded at elevations lower than 800 m.

Specimens were recorded from sifted leaf mold and rotten wood (34 collection records), pitfall traps (3 records), Malaise traps (4 records), ground foraging (1 record), and nesting or foraging in the the root mat on the ground (8 records), under moss on the ground or on rotten logs (2 records), in or under rotten logs (30 and 3 records, respectively), in a rotten tree stump (1 record), and in the soil (8 records).

### Stigmatomma sakalava

Esteves & Fisher sp. n.

urn:lsid:zoobank.org:act:5CD50C18-613E-47D1-86B6-D484A18949F8

#### Materials

- **Type status:** Holotype. **Occurrence:** catalogNumber: casent0366766; recordedBy: B.L.Fisher et al.; sex: 1w; preparations: pin; associatedMedia: http://www.antweb.org/specimen/casent0366766; **Taxon:** scientificName: Stigmatomma sakalava; genus: Stigmatomma; **Location:** locationID: Andranomatavy 275; country: Madagascar; stateProvince: Antsiranana; locality: Ampasindava, Andranomatavy Forest; verbatimElevation: 275; decimalLatitude: -13.6648; decimalLongitude: 47.98702; georeferenceRemarks: ±500m; **Identification:** identifiedBy: F. Esteves; dateIdentified: 2014; **Event:** samplingProtocol: 3 MaxiWinks, mixed samples; eventDate: 10/06/2013; habitat: disturbed dry forest; fieldNumber: BLF31671; eventRemarks: sifted litter; **Record Level:** institutionCode: CASC
- **Type status:** Paratype. **Occurrence:** catalogNumber: casent0366765; recordedBy: B.L.Fisher et al.; sex: 1w; preparations: pin; associatedMedia: http://www.antweb.org/specimen/casent0366765; **Taxon:** scientificName: Stigmatomma sakalava; genus: Stigmatomma; **Location:** locationID: Andranomatavy 275; country: Madagascar; stateProvince: Antsiranana; locality: Ampasindava, Andranomatavy Forest; verbatimElevation: 275; decimalLatitude: -13.6648; decimalLongitude: 47.98702; georeferenceRemarks: ±500m; **Identification:** identifiedBy: F. Esteves; dateIdentified: 2014; **Event:** samplingProtocol: 3 MaxiWinks, mixed samples; eventDate: 10/06/2013; habitat: disturbed dry forest; fieldNumber: BLF31671; eventRemarks: sifted litter; **Record Level:** institutionCode: CASC
- **Type status:** Paratype. **Occurrence:** catalogNumber: casent0318443; recordedBy: B.L.Fisher et al.; sex: 1w; preparations: pin; associatedMedia: http://www.antweb.org/specimen/casent0318443; **Taxon:** scientificName: Stigmatomma sakalava; genus: Stigmatomma; **Location:** locationID: Andranomatavy 275; country: Madagascar; stateProvince: Antsiranana; locality: Ampasindava, Andranomatavy Forest; verbatimElevation: 275; decimalLatitude: -13.6648; decimalLongitude: 47.98702; georeferenceRemarks: ±500m; **Identification:** identifiedBy: F. Esteves; dateIdentified: 2014; **Event:** samplingProtocol: 3 MaxiWinks, mixed samples; eventDate: 10/06/2013; habitat: disturbed dry forest; fieldNumber: BLF31671; eventRemarks: sifted litter; **Record Level:** institutionCode: CASC
- **Type status:** Paratype. **Occurrence:** catalogNumber: casent0366764; recordedBy: B.L.Fisher et al.; sex: 1w; preparations: pin; associatedMedia: http://www.antweb.org/specimen/casent0366764; **Taxon:** scientificName: Stigmatomma sakalava; genus: Stigmatomma; **Location:** locationID: Andranomatavy 275; country: Madagascar; stateProvince: Antsiranana; locality: Ampasindava, Andranomatavy Forest; verbatimElevation: 275; decimalLatitude: -13.6648; decimalLongitude: 47.98702; georeferenceRemarks: ±500m; **Identification:** identifiedBy: F. Esteves; dateIdentified: 2014; **Event:** samplingProtocol: 3 MaxiWinks, mixed samples; eventDate: 10/06/2013; habitat: disturbed dry forest; fieldNumber: BLF31671; eventRemarks: sifted litter; **Record Level:** institutionCode: NHMB
- **Type status:** Paratype. **Occurrence:** catalogNumber: casent0318442; recordedBy: B.L.Fisher et al.; sex: 1w; preparations: pin; associatedMedia: http://www.antweb.org/specimen/casent0318442; **Taxon:** scientificName: Stigmatomma sakalava; genus: Stigmatomma; **Location:** locationID: Andranomatavy 275; country: Madagascar; stateProvince: Antsiranana; locality: Ampasindava, Andranomatavy Forest; verbatimElevation: 275; decimalLatitude: -13.6648; decimalLongitude: 47.98702; georeferenceRemarks: ±500m; **Identification:** identifiedBy: F. Esteves; dateIdentified: 2014; **Event:** samplingProtocol: 3 MaxiWinks, mixed samples; eventDate: 10/06/2013; habitat: disturbed dry forest; fieldNumber: BLF31671; eventRemarks: sifted litter; **Record Level:** institutionCode: BMNH
- **Type status:** Paratype. **Occurrence:** catalogNumber: casent0318441; recordedBy: B.L.Fisher et al.; sex: 1w; preparations: pin; associatedMedia: http://www.antweb.org/specimen/casent0318441; **Taxon:** scientificName: Stigmatomma sakalava; genus: Stigmatomma; **Location:** locationID: Andranomatavy 275; country: Madagascar; stateProvince: Antsiranana; locality: Ampasindava, Andranomatavy Forest; verbatimElevation: 275; decimalLatitude: -13.6648; decimalLongitude: 47.98702; georeferenceRemarks: ±500m; **Identification:** identifiedBy: F. Esteves; dateIdentified: 2014; **Event:** samplingProtocol: 3 MaxiWinks, mixed samples; eventDate: 10/06/2013; habitat: disturbed dry forest; fieldNumber: BLF31671; eventRemarks: sifted litter; **Record Level:** institutionCode: MHNG
- **Type status:** Other material. **Occurrence:** catalogNumber: casent0068192; recordedBy: B.L.Fisher et al.; sex: 1w; preparations: pin; associatedMedia: http://www.antweb.org/specimen/casent0068192; **Taxon:** scientificName: Stigmatomma sakalava; genus: Stigmatomma; **Location:** locationID: Kirindy 100, 15.5 km; country: Madagascar; stateProvince: Toliara; locality: Forêt de Kirindy, 15.5 km 64° ENE Marofandilia; verbatimElevation: 100; decimalLatitude: -20.045; decimalLongitude: 44.66222; georeferenceRemarks: coordinates obtained from GPS; **Identification:** identifiedBy: F. Esteves; dateIdentified: 2014; **Event:** samplingProtocol: MW 50 sample transect, 5m; eventDate: 11/08/2005; habitat: tropical dry forest; fieldNumber: BLF12455; eventRemarks: sifted litter (leaf mold, rotten wood); **Record Level:** institutionCode: CASC
- **Type status:** Other material. **Occurrence:** catalogNumber: casent0022237; recordedBy: Fisher, Griswold et al.; sex: 1w; preparations: pin; associatedMedia: http://www.antweb.org/specimen/casent0022237; **Taxon:** scientificName: Stigmatomma sakalava; genus: Stigmatomma; **Location:** locationID: Androngonibe 30; country: Madagascar; stateProvince: Mahajanga; locality: Réserve Spéciale de Bemarivo, 23.8 km 223° SW Besalampy; verbatimElevation: 30; decimalLatitude: -16.925; decimalLongitude: 44.36833; georeferenceRemarks: coordinates obtained from GPS; **Identification:** identifiedBy: F. Esteves; dateIdentified: 2014; **Event:** samplingProtocol: MW 50 sample transect, 5m; eventDate: 11/19/2002; habitat: tropical dry forest; fieldNumber: BLF06692; eventRemarks: sifted litter (leaf mold, rotten wood); **Record Level:** institutionCode: CASC
- **Type status:** Other material. **Occurrence:** catalogNumber: casent0017557; recordedBy: Fisher-Griswold Arthropod Team; sex: 1w; preparations: pin; associatedMedia: http://www.antweb.org/specimen/casent0017557; **Taxon:** scientificName: Stigmatomma sakalava; genus: Stigmatomma; **Location:** locationID: Andranomite 75; country: Madagascar; stateProvince: Toliara; locality: Forêt de Mite, 20.7 km 29° WNW Tongobory; verbatimElevation: 75; decimalLatitude: -23.52417; decimalLongitude: 44.12133; georeferenceRemarks: coordinates obtained from GPS; **Identification:** identifiedBy: F. Esteves; dateIdentified: 2014; **Event:** samplingProtocol: MW 50 sample transect, 5m; eventDate: 02/27/2002; habitat: gallery forest; fieldNumber: BLF05850; eventRemarks: sifted litter (leaf mold, rotten wood); **Record Level:** institutionCode: CASC
- **Type status:** Other material. **Occurrence:** catalogNumber: casent0015918; recordedBy: Fisher-Griswold Arthropod Team; sex: 1w; preparations: pin; associatedMedia: http://www.antweb.org/specimen/casent0015918; **Taxon:** scientificName: Stigmatomma sakalava; genus: Stigmatomma; **Location:** locationID: Malaza 40; country: Madagascar; stateProvince: Toliara; locality: Réserve Privé Berenty, Forêt de Malaza, Mandraré River, 8.6 km 314° NW Amboasary; verbatimElevation: 40; decimalLatitude: -25.00778; decimalLongitude: 46.306; georeferenceRemarks: coordinates obtained from GPS; **Identification:** identifiedBy: F. Esteves; dateIdentified: 2014; **Event:** samplingProtocol: MW 50 sample transect, 5m; eventDate: 02/06/2002; habitat: gallery forest; fieldNumber: BLF05426; eventRemarks: sifted litter (leaf mold, rotten wood); **Record Level:** institutionCode: CASC
- **Type status:** Other material. **Occurrence:** catalogNumber: casent0438262; recordedBy: Fisher, Griswold et al.; sex: 1w; preparations: SEM mount; associatedMedia: http://www.antweb.org/specimen/casent0438262; **Taxon:** scientificName: Stigmatomma sakalava; genus: Stigmatomma; **Location:** locationID: Ankarana 80; country: Madagascar; stateProvince: Antsiranana; locality: Réserve Spéciale de l'Ankarana, 22.9 km 224° SW Anivorano Nord; verbatimElevation: 80; decimalLatitude: -12.90889; decimalLongitude: 49.10983; georeferenceRemarks: coordinates obtained from GPS; **Identification:** identifiedBy: F. Esteves; dateIdentified: 2014; **Event:** samplingProtocol: MW 50 sample transect, 5m; eventDate: 02/10/2001; habitat: tropical dry forest; fieldNumber: BLF02858; eventRemarks: sifted litter (leaf mold, rotten wood); **Record Level:** institutionCode: CASC
- **Type status:** Other material. **Occurrence:** catalogNumber: casent0022146; recordedBy: Fisher, Griswold et al.; sex: 1w; preparations: SEM mount; associatedMedia: http://www.antweb.org/specimen/casent0022146; **Taxon:** scientificName: Stigmatomma sakalava; genus: Stigmatomma; **Location:** locationID: Mangotoky 100; country: Madagascar; stateProvince: Mahajanga; locality: Parc National de Namoroka, 17.8 km 329° WNW Vilanandro; verbatimElevation: 100; decimalLatitude: -16.37667; decimalLongitude: 45.32667; georeferenceRemarks: coordinates obtained from GPS; **Identification:** identifiedBy: F. Esteves; dateIdentified: 2014; **Event:** samplingProtocol: MW 50 sample transect, 5m; eventDate: 11/08/2002; habitat: tropical dry forest; fieldNumber: BLF06506; eventRemarks: sifted litter (leaf mold, rotten wood); **Record Level:** institutionCode: CASC
- **Type status:** Other material. **Occurrence:** catalogNumber: casent0017556; recordedBy: Fisher-Griswold Arthropod Team; sex: 1w; preparations: SEM mount; associatedMedia: http://www.antweb.org/specimen/casent0017556; **Taxon:** scientificName: Stigmatomma sakalava; genus: Stigmatomma; **Location:** locationID: Andranomite 75; country: Madagascar; stateProvince: Toliara; locality: Forêt de Mite, 20.7 km 29° WNW Tongobory; verbatimElevation: 75; decimalLatitude: -23.52417; decimalLongitude: 44.12133; georeferenceRemarks: coordinates obtained from GPS; **Identification:** identifiedBy: F. Esteves; dateIdentified: 2014; **Event:** samplingProtocol: MW 50 sample transect, 5m; eventDate: 02/27/2002; habitat: gallery forest; fieldNumber: BLF05850; eventRemarks: sifted litter (leaf mold, rotten wood); **Record Level:** institutionCode: CASC
- **Type status:** Other material. **Occurrence:** catalogNumber: casent0015917; recordedBy: Fisher-Griswold Arthropod Team; sex: 1w; preparations: SEM mount; associatedMedia: http://www.antweb.org/specimen/casent0015917; **Taxon:** scientificName: Stigmatomma sakalava; genus: Stigmatomma; **Location:** locationID: Malaza 40; country: Madagascar; stateProvince: Toliara; locality: Réserve Privé Berenty, Forêt de Malaza, Mandraré River, 8.6 km 314° NW Amboasary; verbatimElevation: 40; decimalLatitude: -25.00778; decimalLongitude: 46.306; georeferenceRemarks: coordinates obtained from GPS; **Identification:** identifiedBy: F. Esteves; dateIdentified: 2014; **Event:** samplingProtocol: MW 50 sample transect, 5m; eventDate: 02/06/2002; habitat: gallery forest; fieldNumber: BLF05426; eventRemarks: sifted litter (leaf mold, rotten wood); **Record Level:** institutionCode: CASC

#### Description

Worker (Fig. 88; holotype values within parentheses): **HL**: 0.74-0.76 (0.76); **HW**: 0.58-0.61 (0.61); **HW2**: 0.51-0.57 (0.55); **SL**: 0.42-0.47 (0.45); **ML**: 0.44-0.52 (0.46); **WL**: 0.89-0.94 (0.94); **PPW**: 0.32-0.36 (0.35); **PtL**: 0.31-0.36 (0.35); **PtW**: 0.38-0.44 (0.44); **CI**: 77-83 (80); **SI**: 57-62 (60); **MI**: 58-68 (61); **PtI**: 80-84 (81). 

*Head*:

Mandibular baso-masticatory margin skirted dorsally by row of filiform setae; ventrally, by row of acuminate flattened-apex setae followed by parallel row of longer filiform setae (Fig. 89a). Mandibular dentition arrangement, from base to apex: single larger tooth; smaller single tooth; four pairs of teeth (dorsal tooth row with teeth increasing in length towards mandibular apex; tooth pairs fused basally); single preapical tooth; apical tooth (Fig. 89a, b). Anterior clypeal margin with eight to ten tubercle-like cuticular processes arranged in a single row (Fig. 89a, c). All clypeal cuticular processes save the most lateral ones armed anteriorly with asymmetrical mucronate dentiform seta; most lateral processes unarmed (Fig. 89a, c​). Central-most clypeal cuticular processes around 3x the length of associated dentiform setae. Long, filiform pair of setae on median clypeal area, posterior to the cuticular processes present on anterior margin (Fig. 89a). Pair of shorter, filiform setae on median clypeal area posterior to longer pair of setae (Fig. 89a, c​). Clypeal median area extending between antennal sockets posteriorly as a narrow (or not so narrow) longitudinal strip; frontoclypeal sulcus acute or narrowly rounded (Fig. 89a, c​). Supraclypeal area a round concavity (Fig. 89a, c​). Twelve antennomeres. Genal teeth absent (Fig. 89c). Absence of compound eyes. Palpal formula: 4:2 (four maxillary, two labial; ​Fig. 89d).

*Mesosoma*:

In dorsal view, mesonotum narrower than posterior remainder of mesosoma (Fig. 90a). Metanotal suture absent (Fig. 90a​). Sulcus dividing mesepisternum into anepisternum and katepisternum; katepisternum somewhat rectangular, with dorsoposterior margin angled (Fig. 90b). Metathoracic spiracle slit like, with swollen margin, reduced in size, located in a shallow concavity (Fig. 90b​). Propodeal spiracle round, surrounded by swollen cuticle (Fig. 90b​). Face of propodeal declivity not concave (Fig. 90a​).

*Legs*:

Basoventral half of calcar of strigil lamellar (Fig. 91b). Anterior face of calcar of strigil with tubiform microtrichia (Fig. 91a); posterior face with lanceolate microtrichia (Fig. 91b). Multiple paddle-like setae on anteroventral face of protibial apex, next to calcar of strigil (Fig. 91a). Multiple paddle-like setae on anterior face of probasitarsus (Fig. 91a); row of stout setae on posterior face present or absent (Fig. 91b). Single mesotibial spur with lanceolate microtrichia (Fig. 91c). Slit-like longitudinal sulcus on anterodorsal face of mesobasitarsus (Fig. 91d). Some stout setae on inner face of mesobasitarsus. Two metatibial spurs: simple anterior spur, with lanceolate microtrichia; posterior spur pectinate (Fig. 92a). Anterior face of posterior metatibial spur glabrous (Fig. 92a​); posterior face with antler-like microtrichia (Fig. 92b). Brush of long, truncated, filiform setae on posterior face of metatibial apex, next to posterior metatibial spur (Fig. 92b). Absence of a longitudinal sulcus on the metabasitarsus (Fig. 92a). Brush of setae present on the baso-inner face of the metabasitarsus, formed anteriorly by acuminate, flattened-apex setae, and posteriorly by truncated, filiform setae (Fig. 92). Some stout setae on remainder of inner face of metabasitarsus. Arolium on pro-, meso-, and metapretarsus.

*Metasoma*:

Petiole sessile (Fig. 93a). Ventroanterior margin of petiolar tergite anterior dorso-latero-ventral carina (Ward 1990) much shorter than anterior margin of subpetiolar process, in lateral view (Fig. 93a​). Ventral margin of subpetiolar process with obtuse angle at midpoint (fin-like projection; Fig. 93a​). Absence of fenestra on lateral face of subpetiolar process (Fig. 93a​). Petiolar proprioceptor zone a large, round concavity with few sensilla (Fig. 93b). Scrobiculate sulcus between pretergite and postergite of abdominal segment III and presclerites and postsclerites of abdominal segment IV (Fig. 93c). Presence of eight to ten stout, spiniform setae on the apex of hypopygium (Fig. 93d).

**Figure 94.**
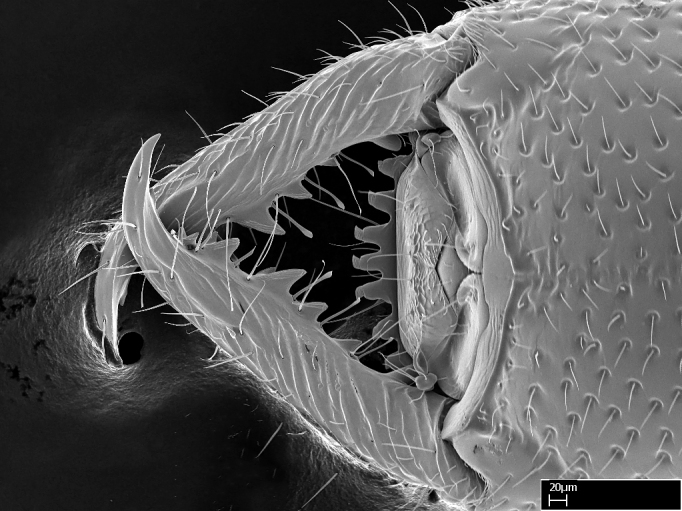
*Stigmatomma sakalava* **sp. n.** worker (CASENT0017556): ventral view of the mandibles, mouth parts, and anterior part of the head. Image by F. A. Esteves; available at AntWeb.org.

*Sculpture*:

Dorsal face of mandible mostly costate-foveolate, except for smooth apical portion (Fig. 89a, b, c). Median clypeal area costate-scarcely foveate (Fig. 89a, c​). Supraclypeal area smooth (Fig. 89a, c​). Head, in dorsal view, foveate; area posterior to tentorial pit plicate (Fig. 89c). Labrum confused alveolate to imbricate apically (Fig. 94). Pronotum foveolate dorsally; remainder of mesosoma foveate dorsally (Fig. 90a). Pronotum mostly dispersed foveolate-weakly alveolate laterally (Fig. 90b). Anepisternum dispersed costulate-weakly alveolate; katepisternum alveolate (Fig. 90b). Metapleuron alveolate-slightly costate (Fig. 90b). Lateral face of propodeum slightly costulate-weakly alveolate or mostly alveolate (Fig. 90b); declivitous face smooth, or weakly alveolate (Fig. 90a). Petiolar tergite alveolate anteroventrally and ventrolaterally, grading into foveolate/foveate dorsally (Fig. 93a); laterotergite confused imbricate/alveolate posteriorly (Fig. 93b); poststernite alveolate (Fig. 93b). Abdominal segments III, IV, V, and VI punctate/foveolate (Fig. 93c); abdominal segment VII mostly smooth (Fig. 93d).

*Pilosity and color*:

Suberect pilosity on head, dorsal face of mesosoma, lateral face of propodeum, petiolar tergite, and abdominal segments III, IV, V, and VI. Petiolar poststernite mostly glabrous. Longer pilosity on abdominal segment VII. Body color orange-brown; appendages yellow to light orange.

##### Comments on character variation

One specimen was very similar to *Stigmatomma sakalava* (CASENT0438262; Fig. 95 ; colleted at Ankarana Special Reserve, at the north limit of *S. sakalava* range). However, that specimen (herein nicknamed morph B) differs from all other *S. sakalava* specimens in the following characters:

**1**. Head: median area of clypeus with longer tubercle-like cuticular processes (approximately 4x the size of the dentiform setae they bear; Figs 95a, 96a). Supraclypeal area is larger (Fig. 96b). Compound eyes are reduced, but present, and possess two ommatidia at their widest diameter (Fig. 96c). The head is round while in *S. sakalava* it is rectangular (Fig. 95a). Dorsal face of the head is foveolate.

**2**. Mesosoma: The mesonotum is longer in dorsal view, and the metanotal suture is present (Fig. 97a). Anepisternum and dorsal portion of katepisternum are visibly wider (Fig. 97b, c). Metathoracic spiracle is oblong, larger, and pinched anteriorly (Fig. 97b, c​). Metapleural carina is well developed and separates the propodeum from the metapleuron (Fig. 97b, c​). The metapleuron is costate, and the lateral face of the propodeum is costate-rugulose-foveolate (Fig. 97b, c​).

**3**. Legs: The simple mesotibial spur bears lanceolate cuticular projections on its ventral margin (Fig. 98a). Brush of setae on the baso-inner face of metabasitarsus is formed by truncated, filiform setae only (Fig. 98b, c).

**4**. Petiole and gaster: Petiole roughly longer than high, in lateral view (Fig. 95b).The tergite of abdominal segment III is divided into pretergite and postergite by a smooth sulcus; in the tergite of the abdominal segment IV, the sulcus is mostly smooth, and in the sternite of the same abdominal segment, the sulcus is scrobiculate, but shallower than the one found in all other specimens of *S. sakalava* (Fig. 99a). The apex of the hypopygium is armed with seven stout spiniform setae (Fig. 99b).

**5**. Body length is much smaller (WL= ; Fig. 95b).

**6.** Body color is yellow (Fig. 95).

Given the morphological variation we found within other *Stigmatomma* species evaluated in this study, it is expected that some of the character variations mentioned above may naturally occur in *S. sakalava*. Examples of taxonomically non-informative characters are: presence/absence and size of compound eyes, sculpture impression on head, length of mesonotum, presence/absence and development of metanotal suture, presence/absence and amount of cuticular projections on the ventral margin of the mesotibial spur, number of spiniform setae on the apex of the hypopygium, and body length.

On the other hand, specimen morph B presents character variations that were not found within other species: head shape, shape and width of anepisternum and katepisternum, metathoracic spiracle shape, development of the metapleural carina, type of constituent setae on the brush present at the baso-inner portion of the metabasitarsus, ratio of petiolar height/length, absence of scrobiculation on the sulci dividing the tergites on abdominal segments III and IV, and development of the sulcus dividing the sternite on abdominal segment IV. Also, personal observations indicate that while recently emerged adults are slightly lighter than others in the same colony, the color difference between morph B and other specimens of *S. sakalava* is striking.

In spite of the differences highlighted above, none of the other characters of the specimen in question differed from those presented in the description of *Stigmatomma sakalava*. Thus, with just one specimen on hand, we decided to delay its description as a new species until more specimens are available for examination. In addition, specimen morph B and the other specimens of *S. sakalava* are allopatric to one another, leaving open the possibility of intraspecific isolation-by-distance and a consequential morphologic variation.

Finally, excluding the specimen morph B, there is no geographic pattern in the variation exhibited by the *Stigmatomma sakalava* specimens we examined regarding body size, the number of dentiform setae on the clypeal anterior margin, amount of clypeus between frontal lobes, number of spiniform setae on the apex of the hypopygium, and scupture.

##### Other castes

Gynes and males unknown.

##### Specimens used in prior studies

This taxon was referenced as *Stigmatomma* MG06 in two previous studies. Yoshimura and Fisher (2012b) used specimen CASENT0022237, and Ward and Fisher (2016) used specimen CASENT0068192-D02.

#### Diagnosis

Worker

With characters of the *tsyhady* species-group and the *sakalava* species-complex as described above, and the following characters (asterisks flag unique characters within the genus in the Malagasy bioregion):

1. Integument orange-brown (Fig. 88); medium-sized ant (HL: 0.74-0.76, WL: 0.89-0.94).
2. Dorsal row of teeth of mandible tooth pairs increasing in size towards apex of mandible (Fig. 89a, b).
3. Flexuous filiform setae ventrally skirting baso-masticatory margin of mandible.
4. Dorsal face of the head foveate (Fig. 89c).
5. Genal teeth absent (Fig. 89c).
6. Palpal formula 4:2 (Fig. 89d).
7. Dorsal face of pronotum foveolate; dorsal face of remainder mesosoma foveate; declivitous face of propodeum smooth, or weakly alveolate (Fig. 90a).
*Katepisternum and metapleuron mostly alveolate; lateral face of propodeum slightly costulate-weakly alveolate, or mostly alveolate (Fig. 90b).
9. Mesepisterum divided into anepisternum and katepisterunm; Katepisternum rectangular, with dorsoposterior margin angled (Fig. 90b).
10. Basoventral half of calcar of strigil lamellar (Fig. 91b).
11. Anterior face of calcar of strigil with tubiform microtrichia (Fig. 91a).
12. Single mesotibial spur covered with lanceolate microtrichia (Fig. 91c).
13. Slit-like longitudinal sulcus present on the anterodorsal face of mesobasitarsus (Fig. 91d).
14. Anterior face of posterior metatibial spur glabrous (Fig. 92a).
15. Brush of long, truncated filiform setae present on the posterior face of metatibial apex (Fig. 92b).
16. Brush of acuminate, flattened-apex setae and truncated, filiform setae present on the baso-inner face of metabasitarsus (Fig. 92).
17. Absence of a longitudinal sulcus on metabasitarsus (Fig. 92a).
18. Subpetiolar process fin-like: ventral margin midpoint obtusely angled (Fig. 93a).
19. Presence of eight to ten stout, spiniform setae on the apex of hypopygium (Fig. 93d).

*Stigmatomma sakalava* and *S. bolabola* share the following characters: absence of genal teeth, palpal formula, single mesotibial spur, head sculpture, shape of the subpetiolar process, and presence of stout, spiniform setae on the apex of hypopygium. However, *S. sakalava* may be distinguished by the smooth or slightly alveolate face of its propodeal declivity, mesosoma lateral face sculpture, katepisternum shape, proportion of lamella on the basoventral margin of calcar of strigil, and distribution (it is not sympatric to any of its congeners).

#### Etymology

The Sakalava people are an ethnic group of Madagascar. They occupy the area along the western coast of the island, from Onilahy River in the south to the island of Nosy Be in the north (Feeley-Harnik 1978), overlapping a large part of the range of *Stigmatomma sakalava*.

#### Distribution

**Figure 100.**
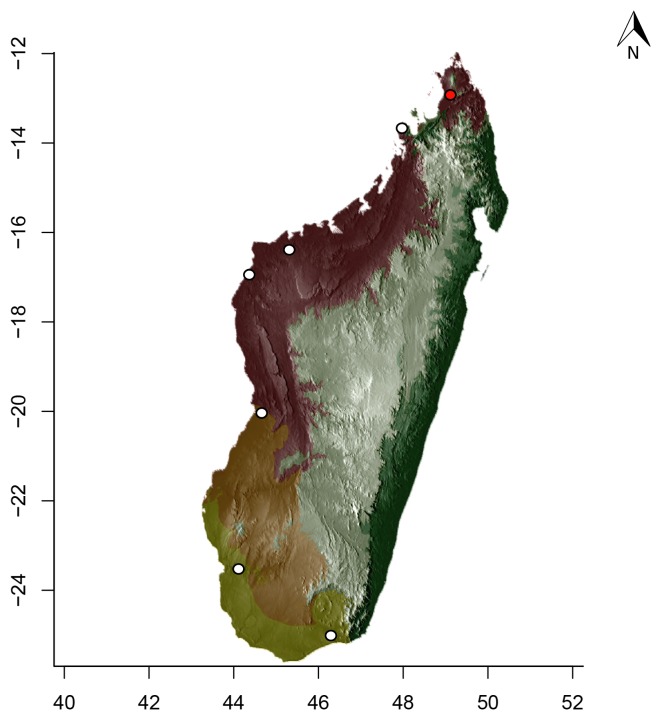
Distribution map of *Stigmatomma sakalava* **sp. n.** in the Malagasy bioregion. Collection localities are mapped over the outlines of five simplified ecoregion zones of Madagascar: humid forests (dark green), subhumid forests (light green), dry deciduous forests (brown), succulent woodlands (orange), and spiny thickets (yellow). The red dot indicates where morph B was collected.

*Stigmatomma sakalava* was collected in gallery forests and in dry deciduous forests. All records fall within the limits of the western and southern biomes of Madagascar (sensu Burgess et al. 2004; Fig. 100). Specimens were distributed at elevations ranging from 30 to 300 m. All specimens were recorded from sifted leaf mold and rotten wood (seven collection records).

### Stigmatomma tsyhady

Esteves & Fisher sp. n.

urn:lsid:zoobank.org:act:34F1B9D3-5C6B-42E2-923D-BC23C92A022D

#### Materials

- **Type status:** Holotype. **Occurrence:** catalogNumber: casent0121332; recordedBy: B.L.Fisher et al.; sex: 1w; preparations: pin; associatedMedia: http://www.antweb.org/specimen/casent0121332; **Taxon:** scientificName: Stigmatomma tsyhady; genus: Stigmatomma; **Location:** country: Madagascar; stateProvince: Toamasina; locality: Ambatovy, 12.4 km NE Moramanga; verbatimElevation: 1080; decimalLatitude: -18.83937; decimalLongitude: 48.30842; georeferenceRemarks: coordinates obtained from GPS; **Event:** samplingProtocol: General collecting; eventDate: 03/08/2007; habitat: montane rainforest; fieldNumber: BLF16936; eventRemarks: ground forager(s); **Record Level:** institutionCode: CASC
- **Type status:** Paratype. **Occurrence:** catalogNumber: casent0318425; recordedBy: B.L.Fisher et al.; sex: 1w; preparations: pin; associatedMedia: http://www.antweb.org/specimen/casent0318425; **Taxon:** scientificName: Stigmatomma tsyhady; genus: Stigmatomma; **Location:** country: Madagascar; stateProvince: Toamasina; locality: Ambatovy, 12.4 km NE Moramanga; verbatimElevation: 1080; decimalLatitude: -18.83937; decimalLongitude: 48.30842; georeferenceRemarks: coordinates obtained from GPS; **Event:** samplingProtocol: General collecting; eventDate: 03/08/2007; habitat: montane rainforest; fieldNumber: BLF16936; eventRemarks: ground forager(s); **Record Level:** institutionCode: CASC
- **Type status:** Paratype. **Occurrence:** catalogNumber: casent0318427; recordedBy: B.L.Fisher et al.; sex: 1w; preparations: pin; associatedMedia: http://www.antweb.org/specimen/casent0318427; **Taxon:** scientificName: Stigmatomma tsyhady; genus: Stigmatomma; **Location:** country: Madagascar; stateProvince: Toamasina; locality: Ambatovy, 12.4 km NE Moramanga; verbatimElevation: 1080; decimalLatitude: -18.83937; decimalLongitude: 48.30842; georeferenceRemarks: coordinates obtained from GPS; **Event:** samplingProtocol: General collecting; eventDate: 03/08/2007; habitat: montane rainforest; fieldNumber: BLF16936; eventRemarks: ground forager(s); **Record Level:** institutionCode: CASC
- **Type status:** Paratype. **Occurrence:** catalogNumber: casent0318426; recordedBy: B.L.Fisher et al.; sex: 1w; preparations: pin; associatedMedia: http://www.antweb.org/specimen/casent0318426; **Taxon:** scientificName: Stigmatomma tsyhady; genus: Stigmatomma; **Location:** country: Madagascar; stateProvince: Toamasina; locality: Ambatovy, 12.4 km NE Moramanga; verbatimElevation: 1080; decimalLatitude: -18.83937; decimalLongitude: 48.30842; georeferenceRemarks: coordinates obtained from GPS; **Event:** samplingProtocol: General collecting; eventDate: 03/08/2007; habitat: montane rainforest; fieldNumber: BLF16936; eventRemarks: ground forager(s); **Record Level:** institutionCode: BMNH
- **Type status:** Paratype. **Occurrence:** catalogNumber: casent0318440; recordedBy: B.L.Fisher et al.; sex: 1w; preparations: pin; associatedMedia: http://www.antweb.org/specimen/casent0318440; **Taxon:** scientificName: Stigmatomma tsyhady; genus: Stigmatomma; **Location:** country: Madagascar; stateProvince: Toamasina; locality: Ambatovy, 12.4 km NE Moramanga; verbatimElevation: 1080; decimalLatitude: -18.83937; decimalLongitude: 48.30842; georeferenceRemarks: coordinates obtained from GPS; **Event:** samplingProtocol: General collecting; eventDate: 03/08/2007; habitat: montane rainforest; fieldNumber: BLF16936; eventRemarks: ground forager(s); **Record Level:** institutionCode: NHMB
- **Type status:** Other material. **Occurrence:** catalogNumber: casent0150901; recordedBy: B.L.Fisher et al.; sex: 1w; preparations: pin; associatedMedia: http://www.antweb.org/specimen/casent0150901; **Taxon:** scientificName: Stigmatomma tsyhady; genus: Stigmatomma; **Location:** country: Madagascar; stateProvince: Toamasina; locality: Parc National de Zahamena, Tetezambatana forest, near junction of Nosivola and Manakambahiny Rivers; verbatimElevation: 860; decimalLatitude: -17.74298; decimalLongitude: 48.72936; georeferenceRemarks: coordinates obtained from GPS; **Event:** samplingProtocol: 10 maxi winks; eventDate: 02/18/2009; habitat: rainforest; fieldNumber: BLF21974; eventRemarks: sifted litter (leaf mold, rotten wood); **Record Level:** institutionCode: CASC
- **Type status:** Other material. **Occurrence:** catalogNumber: casent0150906; recordedBy: B.L.Fisher et al.; sex: 1w; preparations: pin; associatedMedia: http://www.antweb.org/specimen/casent0150906; **Taxon:** scientificName: Stigmatomma tsyhady; genus: Stigmatomma; **Location:** country: Madagascar; stateProvince: Toamasina; locality: Parc National de Zahamena, Tetezambatana forest, near junction of Nosivola and Manakambahiny Rivers; verbatimElevation: 860; decimalLatitude: -17.74298; decimalLongitude: 48.72936; georeferenceRemarks: coordinates obtained from GPS; **Event:** samplingProtocol: 10 maxi winks; eventDate: 02/18/2009; habitat: rainforest; fieldNumber: BLF21974; eventRemarks: sifted litter (leaf mold, rotten wood); **Record Level:** institutionCode: CASC
- **Type status:** Other material. **Occurrence:** catalogNumber: casent0150907; recordedBy: B.L.Fisher et al.; sex: 1w; preparations: pin; associatedMedia: http://www.antweb.org/specimen/casent0150907; **Taxon:** scientificName: Stigmatomma tsyhady; genus: Stigmatomma; **Location:** country: Madagascar; stateProvince: Toamasina; locality: Parc National de Zahamena, Tetezambatana forest, near junction of Nosivola and Manakambahiny Rivers; verbatimElevation: 860; decimalLatitude: -17.74298; decimalLongitude: 48.72936; georeferenceRemarks: coordinates obtained from GPS; **Event:** samplingProtocol: 10 maxi winks; eventDate: 02/18/2009; habitat: rainforest; fieldNumber: BLF21974; eventRemarks: sifted litter (leaf mold, rotten wood); **Record Level:** institutionCode: CASC
- **Type status:** Other material. **Occurrence:** catalogNumber: casent0304824; recordedBy: B.L.Fisher et al.; sex: 1w; preparations: pin; associatedMedia: http://www.antweb.org/specimen/casent0304824; **Taxon:** scientificName: Stigmatomma tsyhady; genus: Stigmatomma; **Location:** country: Madagascar; stateProvince: Antsiranana; locality: Galoko chain, Mont Galoko; verbatimElevation: 980; decimalLatitude: -13.5888; decimalLongitude: 48.72864; georeferenceRemarks: ±200 m; **Event:** samplingProtocol: 10 MaxiWinks, mixed samples; eventDate: 02/22/2013; habitat: montane forest; fieldNumber: BLF30911; eventRemarks: sifted litter; **Record Level:** institutionCode: CASC
- **Type status:** Other material. **Occurrence:** catalogNumber: casent0304825; recordedBy: B.L.Fisher et al.; sex: 1w; preparations: pin; associatedMedia: http://www.antweb.org/specimen/casent0304825; **Taxon:** scientificName: Stigmatomma tsyhady; genus: Stigmatomma; **Location:** country: Madagascar; stateProvince: Antsiranana; locality: Galoko chain, Mont Galoko; verbatimElevation: 980; decimalLatitude: -13.5888; decimalLongitude: 48.72864; georeferenceRemarks: ±200 m; **Event:** samplingProtocol: 10 MaxiWinks, mixed samples; eventDate: 02/22/2013; habitat: montane forest; fieldNumber: BLF30911; eventRemarks: sifted litter; **Record Level:** institutionCode: CASC
- **Type status:** Other material. **Occurrence:** catalogNumber: casent0210643; recordedBy: B.L.Fisher et al.; sex: 1w; preparations: pin; associatedMedia: http://www.antweb.org/specimen/casent0210643; **Taxon:** scientificName: Stigmatomma tsyhady; genus: Stigmatomma; **Location:** country: Madagascar; stateProvince: Toliara; locality: Makay Mts.; verbatimElevation: 510; decimalLatitude: -21.21836; decimalLongitude: 45.3106; georeferenceRemarks: coordinates obtained from GPS; **Event:** samplingProtocol: 10 MaxiWinks, mixed samples; eventDate: 11/24/2010; habitat: Gallery forest on sandy soil; fieldNumber: BLF25204; eventRemarks: sifted litter (leaf mold, rotten wood); **Record Level:** institutionCode: CASC
- **Type status:** Other material. **Occurrence:** catalogNumber: casent0230255; recordedBy: B.L.Fisher et al.; sex: 1w; preparations: pin; associatedMedia: http://www.antweb.org/specimen/casent0230255; **Taxon:** scientificName: Stigmatomma tsyhady; genus: Stigmatomma; **Location:** country: Madagascar; stateProvince: Antsiranana; locality: Makirovana forest; verbatimElevation: 900; decimalLatitude: -14.16506; decimalLongitude: 49.9477; georeferenceRemarks: coordinates obtained from GPS; **Event:** samplingProtocol: 10 MaxiWinks, mixed samples; eventDate: 04/30/2011; habitat: montane rainforest; fieldNumber: BLF26740; eventRemarks: sifted litter (leaf mold, rotten wood); **Record Level:** institutionCode: CASC
- **Type status:** Other material. **Occurrence:** catalogNumber: casent0243169; recordedBy: B.L.Fisher et al.; sex: 1w; preparations: pin; associatedMedia: http://www.antweb.org/specimen/casent0243169; **Taxon:** scientificName: Stigmatomma tsyhady; genus: Stigmatomma; **Location:** country: Madagascar; stateProvince: Antsiranana; locality: Makirovana forest; verbatimElevation: 900; decimalLatitude: -14.16506; decimalLongitude: 49.9477; georeferenceRemarks: coordinates obtained from GPS; **Event:** samplingProtocol: 10 MaxiWinks, mixed samples; eventDate: 04/30/2011; habitat: montane rainforest; fieldNumber: BLF26740; eventRemarks: sifted litter (leaf mold, rotten wood); **Record Level:** institutionCode: CASC
- **Type status:** Other material. **Occurrence:** catalogNumber: casent0245402; recordedBy: B.L.Fisher et al.; sex: 1w; preparations: pin; associatedMedia: http://www.antweb.org/specimen/casent0245402; **Taxon:** scientificName: Stigmatomma tsyhady; genus: Stigmatomma; **Location:** country: Madagascar; stateProvince: Antsiranana; locality: Makirovana forest; verbatimElevation: 415; decimalLatitude: -14.17066; decimalLongitude: 49.95409; georeferenceRemarks: coordinates obtained from GPS; **Event:** samplingProtocol: 10 MaxiWinks, mixed samples; eventDate: 04/28/2011; habitat: rainforest; fieldNumber: BLF26523; eventRemarks: sifted litter (leaf mold, rotten wood); **Record Level:** institutionCode: CASC
- **Type status:** Other material. **Occurrence:** catalogNumber: casent0245406; recordedBy: B.L.Fisher et al.; sex: 1w; preparations: pin; associatedMedia: http://www.antweb.org/specimen/casent0245406; **Taxon:** scientificName: Stigmatomma tsyhady; genus: Stigmatomma; **Location:** country: Madagascar; stateProvince: Antsiranana; locality: Makirovana forest; verbatimElevation: 415; decimalLatitude: -14.17066; decimalLongitude: 49.95409; georeferenceRemarks: coordinates obtained from GPS; **Event:** samplingProtocol: 10 MaxiWinks, mixed samples; eventDate: 04/28/2011; habitat: rainforest; fieldNumber: BLF26523; eventRemarks: sifted litter (leaf mold, rotten wood); **Record Level:** institutionCode: CASC
- **Type status:** Other material. **Occurrence:** catalogNumber: casent0245408; recordedBy: B.L.Fisher et al.; sex: 1w; preparations: pin; associatedMedia: http://www.antweb.org/specimen/casent0245408; **Taxon:** scientificName: Stigmatomma tsyhady; genus: Stigmatomma; **Location:** country: Madagascar; stateProvince: Antsiranana; locality: Makirovana forest; verbatimElevation: 415; decimalLatitude: -14.17066; decimalLongitude: 49.95409; georeferenceRemarks: coordinates obtained from GPS; **Event:** samplingProtocol: 10 MaxiWinks, mixed samples; eventDate: 04/28/2011; habitat: rainforest; fieldNumber: BLF26523; eventRemarks: sifted litter (leaf mold, rotten wood); **Record Level:** institutionCode: CASC
- **Type status:** Other material. **Occurrence:** catalogNumber: casent0245410; recordedBy: B.L.Fisher et al.; sex: 1w; preparations: pin; associatedMedia: http://www.antweb.org/specimen/casent0245410; **Taxon:** scientificName: Stigmatomma tsyhady; genus: Stigmatomma; **Location:** country: Madagascar; stateProvince: Antsiranana; locality: Makirovana forest; verbatimElevation: 415; decimalLatitude: -14.17066; decimalLongitude: 49.95409; georeferenceRemarks: coordinates obtained from GPS; **Event:** samplingProtocol: 10 MaxiWinks, mixed samples; eventDate: 04/28/2011; habitat: rainforest; fieldNumber: BLF26523; eventRemarks: sifted litter (leaf mold, rotten wood); **Record Level:** institutionCode: CASC
- **Type status:** Other material. **Occurrence:** catalogNumber: casent0163904; recordedBy: B.L.Fisher et al.; sex: 1Q; preparations: pin; associatedMedia: http://www.antweb.org/specimen/casent0163904; **Taxon:** scientificName: Stigmatomma tsyhady; genus: Stigmatomma; **Location:** country: Madagascar; stateProvince: Toamasina; locality: Réserve Spéciale Ambatovaky, Sandrangato river; verbatimElevation: 520; decimalLatitude: -16.7633; decimalLongitude: 49.26692; georeferenceRemarks: coordinates obtained from GPS; **Event:** samplingProtocol: 10 MaxiWinks, mixed samples; eventDate: 02/22/2010; habitat: rainforest; fieldNumber: BLF24600; eventRemarks: sifted litter (leaf mold, rotten wood), along crête; **Record Level:** institutionCode: CASC
- **Type status:** Other material. **Occurrence:** catalogNumber: casent0162247; recordedBy: B.L.Fisher et al.; sex: 1w; preparations: pin; associatedMedia: http://www.antweb.org/specimen/casent0162247; **Taxon:** scientificName: Stigmatomma tsyhady; genus: Stigmatomma; **Location:** country: Madagascar; stateProvince: Toamasina; locality: Réserve Spéciale Ambatovaky, Sandrangato river; verbatimElevation: 450; decimalLatitude: -16.77274; decimalLongitude: 49.26551; georeferenceRemarks: coordinates obtained from GPS; **Event:** samplingProtocol: 10 MaxiWinks, mixed samples; eventDate: 02/20/2010; habitat: rainforest; fieldNumber: BLF24310; eventRemarks: sifted litter (leaf mold, rotten wood); **Record Level:** institutionCode: CASC
- **Type status:** Other material. **Occurrence:** catalogNumber: casent0162250; recordedBy: B.L.Fisher et al.; sex: 1w; preparations: pin; associatedMedia: http://www.antweb.org/specimen/casent0162250; **Taxon:** scientificName: Stigmatomma tsyhady; genus: Stigmatomma; **Location:** country: Madagascar; stateProvince: Toamasina; locality: Réserve Spéciale Ambatovaky, Sandrangato river; verbatimElevation: 450; decimalLatitude: -16.77274; decimalLongitude: 49.26551; georeferenceRemarks: coordinates obtained from GPS; **Event:** samplingProtocol: 10 MaxiWinks, mixed samples; eventDate: 02/20/2010; habitat: rainforest; fieldNumber: BLF24310; eventRemarks: sifted litter (leaf mold, rotten wood); **Record Level:** institutionCode: CASC
- **Type status:** Other material. **Occurrence:** catalogNumber: casent0163903; recordedBy: B.L.Fisher et al.; sex: 1w; preparations: pin; associatedMedia: http://www.antweb.org/specimen/casent0163903; **Taxon:** scientificName: Stigmatomma tsyhady; genus: Stigmatomma; **Location:** country: Madagascar; stateProvince: Toamasina; locality: Réserve Spéciale Ambatovaky, Sandrangato river; verbatimElevation: 520; decimalLatitude: -16.7633; decimalLongitude: 49.26692; georeferenceRemarks: coordinates obtained from GPS; **Event:** samplingProtocol: 10 MaxiWinks, mixed samples; eventDate: 02/22/2010; habitat: rainforest; fieldNumber: BLF24600; eventRemarks: sifted litter (leaf mold, rotten wood), along crête; **Record Level:** institutionCode: CASC
- **Type status:** Other material. **Occurrence:** catalogNumber: casent0163908; recordedBy: B.L.Fisher et al.; sex: 1w; preparations: pin; associatedMedia: http://www.antweb.org/specimen/casent0163908; **Taxon:** scientificName: Stigmatomma tsyhady; genus: Stigmatomma; **Location:** country: Madagascar; stateProvince: Toamasina; locality: Réserve Spéciale Ambatovaky, Sandrangato river; verbatimElevation: 520; decimalLatitude: -16.7633; decimalLongitude: 49.26692; georeferenceRemarks: coordinates obtained from GPS; **Event:** samplingProtocol: 10 MaxiWinks, mixed samples; eventDate: 02/22/2010; habitat: rainforest; fieldNumber: BLF24600; eventRemarks: sifted litter (leaf mold, rotten wood), along crête; **Record Level:** institutionCode: CASC
- **Type status:** Other material. **Occurrence:** catalogNumber: casent0163926; recordedBy: B.L.Fisher et al.; sex: 1w; preparations: pin; associatedMedia: http://www.antweb.org/specimen/casent0163926; **Taxon:** scientificName: Stigmatomma tsyhady; genus: Stigmatomma; **Location:** country: Madagascar; stateProvince: Toamasina; locality: Réserve Spéciale Ambatovaky, Sandrangato river; verbatimElevation: 520; decimalLatitude: -16.7633; decimalLongitude: 49.26692; georeferenceRemarks: coordinates obtained from GPS; **Event:** samplingProtocol: 10 MaxiWinks, mixed samples; eventDate: 02/22/2010; habitat: rainforest; fieldNumber: BLF24600; eventRemarks: sifted litter (leaf mold, rotten wood), along crête; **Record Level:** institutionCode: CASC
- **Type status:** Other material. **Occurrence:** catalogNumber: casent0163961; recordedBy: B.L.Fisher et al.; sex: 1w; preparations: pin; associatedMedia: http://www.antweb.org/specimen/casent0163961; **Taxon:** scientificName: Stigmatomma tsyhady; genus: Stigmatomma; **Location:** country: Madagascar; stateProvince: Toamasina; locality: Réserve Spéciale Ambatovaky, Sandrangato river; verbatimElevation: 520; decimalLatitude: -16.7633; decimalLongitude: 49.26692; georeferenceRemarks: coordinates obtained from GPS; **Event:** samplingProtocol: 10 MaxiWinks, mixed samples; eventDate: 02/22/2010; habitat: rainforest; fieldNumber: BLF24600; eventRemarks: sifted litter (leaf mold, rotten wood), along crête; **Record Level:** institutionCode: CASC
- **Type status:** Other material. **Occurrence:** catalogNumber: casent0704854; recordedBy: B.L.Fisher, F.A.Esteves et al.; sex: 1w; preparations: pin; associatedMedia: http://www.antweb.org/specimen/casent0704854; **Taxon:** scientificName: Stigmatomma tsyhady; genus: Stigmatomma; **Location:** country: Madagascar; stateProvince: Toliara; locality: Anosy Region, Anosyenne Mts, 31.2 km NW Manantenina; verbatimElevation: 1125; decimalLatitude: -24.13401; decimalLongitude: 47.05675; georeferenceRemarks: coordinates obtained from GPS, +-50m; **Event:** samplingProtocol: 10 MaxiWinks, mixed samples; eventDate: 02/25/2015; habitat: montane rainforest; fieldNumber: BLF36450; eventRemarks: sifted litter (leaf mold, rotten wood); **Record Level:** institutionCode: CASC
- **Type status:** Other material. **Occurrence:** catalogNumber: casent0704856; recordedBy: B.L.Fisher, F.A.Esteves et al.; sex: 1w; preparations: pin; associatedMedia: http://www.antweb.org/specimen/casent0704856; **Taxon:** scientificName: Stigmatomma tsyhady; genus: Stigmatomma; **Location:** country: Madagascar; stateProvince: Toliara; locality: Anosy Region, Anosyenne Mts, 31.2 km NW Manantenina; verbatimElevation: 1125; decimalLatitude: -24.13401; decimalLongitude: 47.05675; georeferenceRemarks: coordinates obtained from GPS, +-50m; **Event:** samplingProtocol: 10 MaxiWinks, mixed samples; eventDate: 02/25/2015; habitat: montane rainforest; fieldNumber: BLF36450; eventRemarks: sifted litter (leaf mold, rotten wood); **Record Level:** institutionCode: CASC
- **Type status:** Other material. **Occurrence:** catalogNumber: casent0721031; recordedBy: B.L.Fisher, F.A.Esteves et al.; sex: 1w; preparations: pin; associatedMedia: http://www.antweb.org/specimen/casent0721031; **Taxon:** scientificName: Stigmatomma tsyhady; genus: Stigmatomma; **Location:** country: Madagascar; stateProvince: Toliara; locality: Anosy Region, Anosyenne Mts, 31.2 km NW Manantenina; verbatimElevation: 1125; decimalLatitude: -24.13401; decimalLongitude: 47.05675; georeferenceRemarks: coordinates obtained from GPS, +-50m; **Event:** samplingProtocol: 10 MaxiWinks, mixed samples; eventDate: 02/25/2015; habitat: montane rainforest; fieldNumber: BLF36450; eventRemarks: sifted litter (leaf mold, rotten wood); **Record Level:** institutionCode: CASC
- **Type status:** Other material. **Occurrence:** catalogNumber: casent0721033; recordedBy: B.L.Fisher, F.A.Esteves et al.; sex: 1w; preparations: pin; associatedMedia: http://www.antweb.org/specimen/casent0721033; **Taxon:** scientificName: Stigmatomma tsyhady; genus: Stigmatomma; **Location:** country: Madagascar; stateProvince: Toliara; locality: Anosy Region, Anosyenne Mts, 31.2 km NW Manantenina; verbatimElevation: 1125; decimalLatitude: -24.13401; decimalLongitude: 47.05675; georeferenceRemarks: coordinates obtained from GPS, +-50m; **Event:** samplingProtocol: 10 MaxiWinks, mixed samples; eventDate: 02/25/2015; habitat: montane rainforest; fieldNumber: BLF36450; eventRemarks: sifted litter (leaf mold, rotten wood); **Record Level:** institutionCode: CASC
- **Type status:** Other material. **Occurrence:** catalogNumber: casent0721038; recordedBy: B.L.Fisher, F.A.Esteves et al.; sex: 1w; preparations: pin; associatedMedia: http://www.antweb.org/specimen/casent0721038; **Taxon:** scientificName: Stigmatomma tsyhady; genus: Stigmatomma; **Location:** country: Madagascar; stateProvince: Toliara; locality: Anosy Region, Anosyenne Mts, 31.2 km NW Manantenina; verbatimElevation: 1125; decimalLatitude: -24.13401; decimalLongitude: 47.05675; georeferenceRemarks: coordinates obtained from GPS, +-50m; **Event:** samplingProtocol: 10 MaxiWinks, mixed samples; eventDate: 02/25/2015; habitat: montane rainforest; fieldNumber: BLF36450; eventRemarks: sifted litter (leaf mold, rotten wood); **Record Level:** institutionCode: CASC
- **Type status:** Other material. **Occurrence:** catalogNumber: casent0721040; recordedBy: B.L.Fisher, F.A.Esteves et al.; sex: 1dq; preparations: pin; associatedMedia: http://www.antweb.org/specimen/casent0721040; **Taxon:** scientificName: Stigmatomma tsyhady; genus: Stigmatomma; **Location:** country: Madagascar; stateProvince: Toliara; locality: Anosy Region, Anosyenne Mts, 31.2 km NW Manantenina; verbatimElevation: 1125; decimalLatitude: -24.13401; decimalLongitude: 47.05675; georeferenceRemarks: coordinates obtained from GPS, +-50m; **Event:** samplingProtocol: 10 MaxiWinks, mixed samples; eventDate: 02/25/2015; habitat: montane rainforest; fieldNumber: BLF36450; eventRemarks: sifted litter (leaf mold, rotten wood); **Record Level:** institutionCode: CASC
- **Type status:** Other material. **Occurrence:** catalogNumber: casent0721048; recordedBy: B.L.Fisher, F.A.Esteves et al.; sex: 1w; preparations: pin; associatedMedia: http://www.antweb.org/specimen/casent0721048; **Taxon:** scientificName: Stigmatomma tsyhady; genus: Stigmatomma; **Location:** country: Madagascar; stateProvince: Toliara; locality: Anosy Region, Anosyenne Mts, 31.2 km NW Manantenina; verbatimElevation: 1125; decimalLatitude: -24.13401; decimalLongitude: 47.05675; georeferenceRemarks: coordinates obtained from GPS, +-50m; **Event:** samplingProtocol: 10 MaxiWinks, mixed samples; eventDate: 02/25/2015; habitat: montane rainforest; fieldNumber: BLF36450; eventRemarks: sifted litter (leaf mold, rotten wood); **Record Level:** institutionCode: CASC
- **Type status:** Other material. **Occurrence:** catalogNumber: casent0366973; recordedBy: B.L.Fisher et al.; sex: 1w; preparations: pin; associatedMedia: http://www.antweb.org/specimen/casent0366973; **Taxon:** scientificName: Stigmatomma tsyhady; genus: Stigmatomma; **Location:** country: Madagascar; stateProvince: Antsiranana; locality: Galoko chain, Mont Kalabenono; verbatimElevation: 643; decimalLatitude: -13.64179; decimalLongitude: 48.67282; georeferenceRemarks: ±500m; **Event:** samplingProtocol: 3 MaxiWinks, mixed samples; eventDate: 10/10/2013; habitat: rainforest; fieldNumber: BLF31887; eventRemarks: sifted litter; **Record Level:** institutionCode: CASC
- **Type status:** Other material. **Occurrence:** catalogNumber: casent0366976; recordedBy: B.L.Fisher et al.; sex: 1w; preparations: pin; associatedMedia: http://www.antweb.org/specimen/casent0366976; **Taxon:** scientificName: Stigmatomma tsyhady; genus: Stigmatomma; **Location:** country: Madagascar; stateProvince: Antsiranana; locality: Galoko chain, Mont Kalabenono; verbatimElevation: 643; decimalLatitude: -13.64179; decimalLongitude: 48.67282; georeferenceRemarks: ±500m; **Event:** samplingProtocol: 3 MaxiWinks, mixed samples; eventDate: 10/10/2013; habitat: rainforest; fieldNumber: BLF31887; eventRemarks: sifted litter; **Record Level:** institutionCode: CASC
- **Type status:** Other material. **Occurrence:** catalogNumber: casent0369172; recordedBy: B.L.Fisher et al.; sex: 1w; preparations: pin; associatedMedia: http://www.antweb.org/specimen/casent0369172; **Taxon:** scientificName: Stigmatomma tsyhady; genus: Stigmatomma; **Location:** country: Madagascar; stateProvince: Antsiranana; locality: Galoko chain, Mont Kalabenono; verbatimElevation: 937; decimalLatitude: -13.64609; decimalLongitude: 48.67732; georeferenceRemarks: ±500m; **Event:** samplingProtocol: 3 MaxiWinks, mixed samples; eventDate: 10/10/2013; habitat: rainforest; fieldNumber: BLF31888; eventRemarks: sifted litter; **Record Level:** institutionCode: CASC
- **Type status:** Other material. **Occurrence:** catalogNumber: casent0128409; recordedBy: B.L.Fisher et al.; sex: 1w; preparations: pin; associatedMedia: http://www.antweb.org/specimen/casent0128409; **Taxon:** scientificName: Stigmatomma tsyhady; genus: Stigmatomma; **Location:** country: Madagascar; stateProvince: Toliara; locality: Manatantely, 8.9km NW Tolagnaro; verbatimElevation: 100; decimalLatitude: -24.9815; decimalLongitude: 46.92567; georeferenceRemarks: coordinates obtained from GPS; **Event:** samplingProtocol: 4 MaxiWinks, mixed samples; eventDate: 11/27/2006; habitat: rainforest; fieldNumber: BLF15358; eventRemarks: sifted litter (leaf mold, rotten wood), 24 hours; **Record Level:** institutionCode: CASC
- **Type status:** Other material. **Occurrence:** catalogNumber: casent0068608; recordedBy: B.L.Fisher et al.; sex: 1dQ; preparations: pin; associatedMedia: http://www.antweb.org/specimen/casent0068608; **Taxon:** scientificName: Stigmatomma tsyhady; genus: Stigmatomma; **Location:** country: Madagascar; stateProvince: Toamasina; locality: Parc National Mananara-Nord, 7.1 km 261° Antanambe; verbatimElevation: 225; decimalLatitude: -16.455; decimalLongitude: 49.7875; georeferenceRemarks: coordinates obtained from GPS; **Event:** samplingProtocol: 8 maxi winks; eventDate: 11/14/2005; habitat: rainforest; fieldNumber: BLF12556; eventRemarks: sifted litter (leaf mold, rotten wood); **Record Level:** institutionCode: CASC
- **Type status:** Other material. **Occurrence:** catalogNumber: casent0068609; recordedBy: B.L.Fisher et al.; sex: 1w; preparations: pin; associatedMedia: http://www.antweb.org/specimen/casent0068609; **Taxon:** scientificName: Stigmatomma tsyhady; genus: Stigmatomma; **Location:** country: Madagascar; stateProvince: Toamasina; locality: Parc National Mananara-Nord, 7.1 km 261° Antanambe; verbatimElevation: 225; decimalLatitude: -16.455; decimalLongitude: 49.7875; georeferenceRemarks: coordinates obtained from GPS; **Event:** samplingProtocol: 8 maxi winks; eventDate: 11/14/2005; habitat: rainforest; fieldNumber: BLF12556; eventRemarks: sifted litter (leaf mold, rotten wood); **Record Level:** institutionCode: CASC
- **Type status:** Other material. **Occurrence:** catalogNumber: casent0068627; recordedBy: B.L.Fisher et al.; sex: 1w; preparations: pin; associatedMedia: http://www.antweb.org/specimen/casent0068627; **Taxon:** scientificName: Stigmatomma tsyhady; genus: Stigmatomma; **Location:** country: Madagascar; stateProvince: Toamasina; locality: Parc National Mananara-Nord, 7.1 km 261° Antanambe; verbatimElevation: 225; decimalLatitude: -16.455; decimalLongitude: 49.7875; georeferenceRemarks: coordinates obtained from GPS; **Event:** samplingProtocol: 8 maxi winks; eventDate: 11/14/2005; habitat: rainforest; fieldNumber: BLF12556; eventRemarks: sifted litter (leaf mold, rotten wood); **Record Level:** institutionCode: CASC
- **Type status:** Other material. **Occurrence:** catalogNumber: casent0069939; recordedBy: B.L.Fisher et al.; sex: 1w; preparations: pin; associatedMedia: http://www.antweb.org/specimen/casent0069939; **Taxon:** scientificName: Stigmatomma tsyhady; genus: Stigmatomma; **Location:** country: Madagascar; stateProvince: Toamasina; locality: Parc National Mananara-Nord, 7.1 km 261° Antanambe; verbatimElevation: 225; decimalLatitude: -16.455; decimalLongitude: 49.7875; georeferenceRemarks: coordinates obtained from GPS; **Event:** samplingProtocol: 8 maxi winks; eventDate: 11/14/2005; habitat: rainforest; fieldNumber: BLF12556; eventRemarks: sifted litter (leaf mold, rotten wood); **Record Level:** institutionCode: CASC
- **Type status:** Other material. **Occurrence:** catalogNumber: casent0067258; recordedBy: B.L. Fisher et al.; sex: 1w; preparations: pin; associatedMedia: http://www.antweb.org/specimen/casent0067258; **Taxon:** scientificName: Stigmatomma tsyhady; genus: Stigmatomma; **Location:** country: Madagascar; stateProvince: Fianarantsoa; locality: Forêt de Vevembe, 66.6 km 293° Farafangana; verbatimElevation: 600; decimalLatitude: -22.791; decimalLongitude: 47.18183; georeferenceRemarks: coordinates obtained from GPS; **Event:** samplingProtocol: 9 Maxi winklers; eventDate: 04/23/2006; habitat: rainforest, transition to montane forest; fieldNumber: BLF14120; **Record Level:** institutionCode: CASC
- **Type status:** Other material. **Occurrence:** catalogNumber: casent0067260; recordedBy: B.L. Fisher et al.; sex: 1w; preparations: pin; associatedMedia: http://www.antweb.org/specimen/casent0067260; **Taxon:** scientificName: Stigmatomma tsyhady; genus: Stigmatomma; **Location:** country: Madagascar; stateProvince: Fianarantsoa; locality: Forêt de Vevembe, 66.6 km 293° Farafangana; verbatimElevation: 600; decimalLatitude: -22.791; decimalLongitude: 47.18183; georeferenceRemarks: coordinates obtained from GPS; **Event:** samplingProtocol: 9 Maxi winklers; eventDate: 04/23/2006; habitat: rainforest, transition to montane forest; fieldNumber: BLF14120; **Record Level:** institutionCode: CASC
- **Type status:** Other material. **Occurrence:** catalogNumber: casent0067262; recordedBy: B.L. Fisher et al.; sex: 1w; preparations: pin; associatedMedia: http://www.antweb.org/specimen/casent0067262; **Taxon:** scientificName: Stigmatomma tsyhady; genus: Stigmatomma; **Location:** country: Madagascar; stateProvince: Fianarantsoa; locality: Forêt de Vevembe, 66.6 km 293° Farafangana; verbatimElevation: 600; decimalLatitude: -22.791; decimalLongitude: 47.18183; georeferenceRemarks: coordinates obtained from GPS; **Event:** samplingProtocol: 9 Maxi winklers; eventDate: 04/23/2006; habitat: rainforest, transition to montane forest; fieldNumber: BLF14120; **Record Level:** institutionCode: CASC
- **Type status:** Other material. **Occurrence:** catalogNumber: casent0072900; recordedBy: B.L. Fisher et al.; sex: 1w; preparations: pin; associatedMedia: http://www.antweb.org/specimen/casent0072900; **Taxon:** scientificName: Stigmatomma tsyhady; genus: Stigmatomma; **Location:** country: Madagascar; stateProvince: Fianarantsoa; locality: Réserve Speciale Manombo 24.5 km 228° Farafangana; verbatimElevation: 30; decimalLatitude: -23.01583; decimalLongitude: 47.719; georeferenceRemarks: coordinates obtained from GPS; **Event:** samplingProtocol: 9 Maxi winklers; eventDate: 04/20/2006; habitat: rainforest; fieldNumber: BLF13963; **Record Level:** institutionCode: CASC
- **Type status:** Paratype. **Occurrence:** catalogNumber: casent0121349; recordedBy: B.L.Fisher et al.; sex: 1w; preparations: pin; associatedMedia: http://www.antweb.org/specimen/casent0121349; **Taxon:** scientificName: Stigmatomma tsyhady; genus: Stigmatomma; **Location:** country: Madagascar; stateProvince: Toamasina; locality: Ambatovy, 12.4 km NE Moramanga; verbatimElevation: 1080; decimalLatitude: -18.83937; decimalLongitude: 48.30842; georeferenceRemarks: coordinates obtained from GPS; **Event:** samplingProtocol: General collecting; eventDate: 03/08/2007; habitat: montane rainforest; fieldNumber: BLF16939; eventRemarks: ground forager(s); **Record Level:** institutionCode: MHNG
- **Type status:** Other material. **Occurrence:** catalogNumber: casent0208784; recordedBy: B.L.Fisher et al.; sex: 1w; preparations: pin; associatedMedia: http://www.antweb.org/specimen/casent0208784; **Taxon:** scientificName: Stigmatomma tsyhady; genus: Stigmatomma; **Location:** country: Madagascar; stateProvince: Fianarantsoa; locality: Anja Reserve; verbatimElevation: 990; decimalLatitude: -21.85241; decimalLongitude: 46.84579; georeferenceRemarks: coordinates obtained from GPS; **Event:** samplingProtocol: General collecting; eventDate: 12/14/2010; habitat: Degraded forest below granite out crop; fieldNumber: BLF25963; eventRemarks: under rootmat, litter on rock; **Record Level:** institutionCode: CASC
- **Type status:** Other material. **Occurrence:** catalogNumber: casent0367488; recordedBy: B.L.Fisher et al.; sex: 1w; preparations: pin; associatedMedia: http://www.antweb.org/specimen/casent0367488; **Taxon:** scientificName: Stigmatomma tsyhady; genus: Stigmatomma; **Location:** country: Madagascar; stateProvince: Antsiranana; locality: Galoko chain, Mont Kalabenono; verbatimElevation: 937; decimalLatitude: -13.64609; decimalLongitude: 48.67732; georeferenceRemarks: ±500m; **Event:** samplingProtocol: General collecting; eventDate: 10/13/2013; habitat: rainforest; fieldNumber: BLF31941; eventRemarks: ex soil; **Record Level:** institutionCode: CASC
- **Type status:** Other material. **Occurrence:** catalogNumber: casent0370064; recordedBy: B.L.Fisher et al.; sex: 1w; preparations: pin; associatedMedia: http://www.antweb.org/specimen/casent0370064; **Taxon:** scientificName: Stigmatomma tsyhady; genus: Stigmatomma; **Location:** country: Madagascar; stateProvince: Antsiranana; locality: Galoko chain, Mont Kalabenono; verbatimElevation: 937; decimalLatitude: -13.64609; decimalLongitude: 48.67732; georeferenceRemarks: ±500m; **Event:** samplingProtocol: General collecting; eventDate: 10/13/2013; habitat: rainforest; fieldNumber: BLF31940; eventRemarks: ex soil; **Record Level:** institutionCode: CASC
- **Type status:** Other material. **Occurrence:** catalogNumber: casent0230064; recordedBy: B.L.Fisher et al.; sex: 1w; preparations: pin; associatedMedia: http://www.antweb.org/specimen/casent0230064; **Taxon:** scientificName: Stigmatomma tsyhady; genus: Stigmatomma; **Location:** country: Madagascar; stateProvince: Antsiranana; locality: Makirovana forest; verbatimElevation: 415; decimalLatitude: -14.17066; decimalLongitude: 49.95409; georeferenceRemarks: coordinates obtained from GPS; **Event:** samplingProtocol: General collecting; eventDate: 04/28/2011; habitat: rainforest; fieldNumber: BLF26587; eventRemarks: ex rotten log, live colony collected and taken with Christian Peeters; **Record Level:** institutionCode: CASC
- **Type status:** Other material. **Occurrence:** catalogNumber: casent0072579; recordedBy: Val C.; sex: 1w; preparations: pin; associatedMedia: http://www.antweb.org/specimen/casent0072579; **Taxon:** scientificName: Stigmatomma tsyhady; genus: Stigmatomma; **Location:** country: Madagascar; stateProvince: Fianarantsoa; locality: Parc National de Ranomafana, Vatoharanana; verbatimElevation: 1100; decimalLatitude: -21.29067; decimalLongitude: 47.42617; **Event:** samplingProtocol: General collecting; eventDate: 03/31/2003; habitat: old-growth primary forest; fieldNumber: VCR004; **Record Level:** institutionCode: CASC
- **Type status:** Other material. **Occurrence:** catalogNumber: casent0070558; recordedBy: Val C. et al.; sex: 1w; preparations: pin; associatedMedia: http://www.antweb.org/specimen/casent0070558; **Taxon:** scientificName: Stigmatomma tsyhady; genus: Stigmatomma; **Location:** country: Madagascar; stateProvince: Fianarantsoa; locality: Parc National de Ranomafana, Voniparara; verbatimElevation: 1100; decimalLatitude: -21.22633; decimalLongitude: 47.36983; **Event:** samplingProtocol: General collecting; eventDate: 03/14/2003; habitat: disturbed roadside forest; fieldNumber: VCR001; **Record Level:** institutionCode: CASC
- **Type status:** Other material. **Occurrence:** catalogNumber: casent0724170; recordedBy: B.L.Fisher, F.A.Esteves et al.; sex: 2w; preparations: pin; associatedMedia: http://www.antweb.org/specimen/casent0724170; **Taxon:** scientificName: Stigmatomma tsyhady; genus: Stigmatomma; **Location:** country: Madagascar; stateProvince: Toliara; locality: Anosy Region, Anosyenne Mts, 29.33 km NW Manantenina; verbatimElevation: 540; decimalLatitude: -24.13993; decimalLongitude: 47.07418; georeferenceRemarks: coordinates obtained from GPS, +-50m; **Event:** samplingProtocol: General collecting; eventDate: 02/21/2015; habitat: rainforest; fieldNumber: BLF36118; eventRemarks: under root mat on rock; **Record Level:** institutionCode: CASC
- **Type status:** Other material. **Occurrence:** catalogNumber: casent0724169; recordedBy: B.L.Fisher, F.A.Esteves et al.; sex: 1w; preparations: pin; associatedMedia: http://www.antweb.org/specimen/casent0724169; **Taxon:** scientificName: Stigmatomma tsyhady; genus: Stigmatomma; **Location:** country: Madagascar; stateProvince: Toliara; locality: Anosy Region, Anosyenne Mts, 29.33 km NW Manantenina; verbatimElevation: 540; decimalLatitude: -24.13993; decimalLongitude: 47.07418; georeferenceRemarks: coordinates obtained from GPS, +-50m; **Event:** samplingProtocol: General collecting; eventDate: 02/21/2015; habitat: rainforest; fieldNumber: BLF36118; eventRemarks: under root mat on rock; **Record Level:** institutionCode: CASC
- **Type status:** Other material. **Occurrence:** catalogNumber: casent0724168; recordedBy: B.L.Fisher, F.A.Esteves et al.; sex: 1w; preparations: pin; associatedMedia: http://www.antweb.org/specimen/casent0724168; **Taxon:** scientificName: Stigmatomma tsyhady; genus: Stigmatomma; **Location:** country: Madagascar; stateProvince: Toliara; locality: Anosy Region, Anosyenne Mts, 29.33 km NW Manantenina; verbatimElevation: 540; decimalLatitude: -24.13993; decimalLongitude: 47.07418; georeferenceRemarks: coordinates obtained from GPS, +-50m; **Event:** samplingProtocol: General collecting; eventDate: 02/21/2015; habitat: rainforest; fieldNumber: BLF36118; eventRemarks: under root mat on rock; **Record Level:** institutionCode: CASC
- **Type status:** Other material. **Occurrence:** catalogNumber: casent0723306; recordedBy: B.L.Fisher, F.A.Esteves et al.; sex: 1w; preparations: pin; associatedMedia: http://www.antweb.org/specimen/casent0723306; **Taxon:** scientificName: Stigmatomma tsyhady; genus: Stigmatomma; **Location:** country: Madagascar; stateProvince: Toliara; locality: Anosy Region, Anosyenne Mts, 31.2 km NW Manantenina; verbatimElevation: 1125; decimalLatitude: -24.13894; decimalLongitude: 47.06804; georeferenceRemarks: coordinates obtained from GPS, +-50m; **Event:** samplingProtocol: General collecting; eventDate: 02/26/2015; habitat: montane rainforest; fieldNumber: BLF36567; eventRemarks: ex root mat on soil; **Record Level:** institutionCode: CASC
- **Type status:** Other material. **Occurrence:** catalogNumber: casent0723305; recordedBy: B.L.Fisher, F.A.Esteves et al.; sex: 1w; preparations: pin; associatedMedia: http://www.antweb.org/specimen/casent0723305; **Taxon:** scientificName: Stigmatomma tsyhady; genus: Stigmatomma; **Location:** country: Madagascar; stateProvince: Toliara; locality: Anosy Region, Anosyenne Mts, 31.2 km NW Manantenina; verbatimElevation: 1125; decimalLatitude: -24.13894; decimalLongitude: 47.06804; georeferenceRemarks: coordinates obtained from GPS, +-50m; **Event:** samplingProtocol: General collecting; eventDate: 02/26/2015; habitat: montane rainforest; fieldNumber: BLF36567; eventRemarks: ex root mat on soil; **Record Level:** institutionCode: CASC
- **Type status:** Other material. **Occurrence:** catalogNumber: casent0723304; recordedBy: B.L.Fisher, F.A.Esteves et al.; sex: 1w; preparations: pin; associatedMedia: http://www.antweb.org/specimen/casent0723304; **Taxon:** scientificName: Stigmatomma tsyhady; genus: Stigmatomma; **Location:** country: Madagascar; stateProvince: Toliara; locality: Anosy Region, Anosyenne Mts, 31.2 km NW Manantenina; verbatimElevation: 1125; decimalLatitude: -24.13894; decimalLongitude: 47.06804; georeferenceRemarks: coordinates obtained from GPS, +-50m; **Event:** samplingProtocol: General collecting; eventDate: 02/26/2015; habitat: montane rainforest; fieldNumber: BLF36567; eventRemarks: ex root mat on soil; **Record Level:** institutionCode: CASC
- **Type status:** Other material. **Occurrence:** catalogNumber: casent0723247; recordedBy: B.L.Fisher, F.A.Esteves et al.; sex: 1w; preparations: pin; associatedMedia: http://www.antweb.org/specimen/casent0723247; **Taxon:** scientificName: Stigmatomma tsyhady; genus: Stigmatomma; **Location:** country: Madagascar; stateProvince: Toliara; locality: Anosy Region, Anosyenne Mts, 31.2 km NW Manantenina; verbatimElevation: 1125; decimalLatitude: -24.13894; decimalLongitude: 47.06804; georeferenceRemarks: coordinates obtained from GPS, +-50m; **Event:** samplingProtocol: General collecting; eventDate: 02/26/2015; habitat: montane rainforest; fieldNumber: BLF36487; eventRemarks: ex root mat; **Record Level:** institutionCode: CASC
- **Type status:** Other material. **Occurrence:** catalogNumber: casent0723248; recordedBy: B.L.Fisher, F.A.Esteves et al.; sex: 1aq; preparations: pin; associatedMedia: http://www.antweb.org/specimen/casent0723248; **Taxon:** scientificName: Stigmatomma tsyhady; genus: Stigmatomma; **Location:** country: Madagascar; stateProvince: Toliara; locality: Anosy Region, Anosyenne Mts, 31.2 km NW Manantenina; verbatimElevation: 1125; decimalLatitude: -24.13894; decimalLongitude: 47.06804; georeferenceRemarks: coordinates obtained from GPS, +-50m; **Event:** samplingProtocol: General collecting; eventDate: 02/26/2015; habitat: montane rainforest; fieldNumber: BLF36487; eventRemarks: ex root mat; **Record Level:** institutionCode: CASC
- **Type status:** Other material. **Occurrence:** catalogNumber: casent0723249; recordedBy: B.L.Fisher, F.A.Esteves et al.; sex: 1w.1m.; preparations: pin; associatedMedia: http://www.antweb.org/specimen/casent0723249; **Taxon:** scientificName: Stigmatomma tsyhady; genus: Stigmatomma; **Location:** country: Madagascar; stateProvince: Toliara; locality: Anosy Region, Anosyenne Mts, 31.2 km NW Manantenina; verbatimElevation: 1125; decimalLatitude: -24.13894; decimalLongitude: 47.06804; georeferenceRemarks: coordinates obtained from GPS, +-50m; **Event:** samplingProtocol: General collecting; eventDate: 02/26/2015; habitat: montane rainforest; fieldNumber: BLF36487; eventRemarks: ex root mat; **Record Level:** institutionCode: CASC
- **Type status:** Other material. **Occurrence:** catalogNumber: casent0723250; recordedBy: B.L.Fisher, F.A.Esteves et al.; sex: 1w; preparations: pin; associatedMedia: http://www.antweb.org/specimen/casent0723250; **Taxon:** scientificName: Stigmatomma tsyhady; genus: Stigmatomma; **Location:** country: Madagascar; stateProvince: Toliara; locality: Anosy Region, Anosyenne Mts, 31.2 km NW Manantenina; verbatimElevation: 1125; decimalLatitude: -24.13894; decimalLongitude: 47.06804; georeferenceRemarks: coordinates obtained from GPS, +-50m; **Event:** samplingProtocol: General collecting; eventDate: 02/26/2015; habitat: montane rainforest; fieldNumber: BLF36487; eventRemarks: ex root mat; **Record Level:** institutionCode: CASC
- **Type status:** Other material. **Occurrence:** catalogNumber: casent0723251; recordedBy: B.L.Fisher, F.A.Esteves et al.; sex: 1w.1m.; preparations: pin; associatedMedia: http://www.antweb.org/specimen/casent0723251; **Taxon:** scientificName: Stigmatomma tsyhady; genus: Stigmatomma; **Location:** country: Madagascar; stateProvince: Toliara; locality: Anosy Region, Anosyenne Mts, 31.2 km NW Manantenina; verbatimElevation: 1125; decimalLatitude: -24.13894; decimalLongitude: 47.06804; georeferenceRemarks: coordinates obtained from GPS, +-50m; **Event:** samplingProtocol: General collecting; eventDate: 02/26/2015; habitat: montane rainforest; fieldNumber: BLF36487; eventRemarks: ex root mat; **Record Level:** institutionCode: CASC
- **Type status:** Other material. **Occurrence:** catalogNumber: casent0723252; recordedBy: B.L.Fisher, F.A.Esteves et al.; sex: 1w; preparations: pin; associatedMedia: http://www.antweb.org/specimen/casent0723252; **Taxon:** scientificName: Stigmatomma tsyhady; genus: Stigmatomma; **Location:** country: Madagascar; stateProvince: Toliara; locality: Anosy Region, Anosyenne Mts, 31.2 km NW Manantenina; verbatimElevation: 1125; decimalLatitude: -24.13894; decimalLongitude: 47.06804; georeferenceRemarks: coordinates obtained from GPS, +-50m; **Event:** samplingProtocol: General collecting; eventDate: 02/26/2015; habitat: montane rainforest; fieldNumber: BLF36487; eventRemarks: ex root mat; **Record Level:** institutionCode: CASC
- **Type status:** Other material. **Occurrence:** catalogNumber: casent0724165; recordedBy: B.L.Fisher, F.A.Esteves et al.; sex: 1w; preparations: pin; associatedMedia: http://www.antweb.org/specimen/casent0724165; **Taxon:** scientificName: Stigmatomma tsyhady; genus: Stigmatomma; **Location:** country: Madagascar; stateProvince: Toliara; locality: Anosy Region, Anosyenne Mts, 31.2 km NW Manantenina; verbatimElevation: 1125; decimalLatitude: -24.13894; decimalLongitude: 47.06804; georeferenceRemarks: coordinates obtained from GPS, +-50m; **Event:** samplingProtocol: General collecting; eventDate: 02/26/2015; habitat: montane rainforest; fieldNumber: BLF36574; eventRemarks: ex root mat; **Record Level:** institutionCode: CASC
- **Type status:** Other material. **Occurrence:** catalogNumber: casent0209263; recordedBy: B.L.Fisher et al.; sex: 1aQ; preparations: pin; associatedMedia: http://www.antweb.org/specimen/casent0209263; **Taxon:** scientificName: Stigmatomma tsyhady; genus: Stigmatomma; **Location:** country: Madagascar; stateProvince: Toliara; locality: Makay Mts.; verbatimElevation: 500; decimalLatitude: -21.21985; decimalLongitude: 45.32396; georeferenceRemarks: coordinates obtained from GPS; **Event:** samplingProtocol: Malaise trap; eventDate: 11/24/2010; habitat: gallery forest on sandy soil; fieldNumber: BLF25357; **Record Level:** institutionCode: CASC
- **Type status:** Other material. **Occurrence:** catalogNumber: casent0120702; recordedBy: B.L.Fisher et al.; sex: 1w; preparations: pin; associatedMedia: http://www.antweb.org/specimen/casent0120702; **Taxon:** scientificName: Stigmatomma tsyhady; genus: Stigmatomma; **Location:** country: Madagascar; stateProvince: Toamasina; locality: Ambatovy, 12.4 km NE Moramanga; verbatimElevation: 1010; decimalLatitude: -18.84963; decimalLongitude: 48.2947; georeferenceRemarks: coordinates obtained from GPS; **Event:** samplingProtocol: MW 25 sample transect, 5m; eventDate: 03/03/2007; habitat: montane rainforest; fieldNumber: BLF16914; eventRemarks: sifted litter (leaf mold, rotten wood); **Record Level:** institutionCode: CASC
- **Type status:** Other material. **Occurrence:** catalogNumber: casent0120744; recordedBy: B.L.Fisher et al.; sex: 1w; preparations: pin; associatedMedia: http://www.antweb.org/specimen/casent0120744; **Taxon:** scientificName: Stigmatomma tsyhady; genus: Stigmatomma; **Location:** country: Madagascar; stateProvince: Toamasina; locality: Ambatovy, 12.4 km NE Moramanga; verbatimElevation: 1010; decimalLatitude: -18.84963; decimalLongitude: 48.2947; georeferenceRemarks: coordinates obtained from GPS; **Event:** samplingProtocol: MW 25 sample transect, 5m; eventDate: 03/03/2007; habitat: montane rainforest; fieldNumber: BLF16914; eventRemarks: sifted litter (leaf mold, rotten wood); **Record Level:** institutionCode: CASC
- **Type status:** Other material. **Occurrence:** catalogNumber: hjr121(12)-6; recordedBy: H.J.Ratsirarson; sex: 1dQ; preparations: pin; associatedMedia: http://www.antweb.org/specimen/hjr121(12)-6; **Taxon:** scientificName: Stigmatomma tsyhady; genus: Stigmatomma; **Location:** country: Madagascar; stateProvince: Toamasina; locality: F.C. Andriantantely; verbatimElevation: 530; decimalLatitude: -18.695; decimalLongitude: 48.81333; **Event:** samplingProtocol: MW 25 sample transect, 5m; eventDate: 12/04/1998; habitat: rainforest; fieldNumber: HJR121; eventRemarks: sifted litter (leaf mold, rotten wood); **Record Level:** institutionCode: CASC
- **Type status:** Other material. **Occurrence:** catalogNumber: hjr121(02)-5; recordedBy: H.J.Ratsirarson; sex: 1w; preparations: pin; associatedMedia: http://www.antweb.org/specimen/hjr121(02)-5; **Taxon:** scientificName: Stigmatomma tsyhady; genus: Stigmatomma; **Location:** country: Madagascar; stateProvince: Toamasina; locality: F.C. Andriantantely; verbatimElevation: 530; decimalLatitude: -18.695; decimalLongitude: 48.81333; **Event:** samplingProtocol: MW 25 sample transect, 5m; eventDate: 12/04/1998; habitat: rainforest; fieldNumber: HJR121; eventRemarks: sifted litter (leaf mold, rotten wood); **Record Level:** institutionCode: CASC
- **Type status:** Other material. **Occurrence:** catalogNumber: hjr121(03)-8; recordedBy: H.J.Ratsirarson; sex: 1w; preparations: pin; associatedMedia: http://www.antweb.org/specimen/hjr121(03)-8; **Taxon:** scientificName: Stigmatomma tsyhady; genus: Stigmatomma; **Location:** country: Madagascar; stateProvince: Toamasina; locality: F.C. Andriantantely; verbatimElevation: 530; decimalLatitude: -18.695; decimalLongitude: 48.81333; **Event:** samplingProtocol: MW 25 sample transect, 5m; eventDate: 12/04/1998; habitat: rainforest; fieldNumber: HJR121; eventRemarks: sifted litter (leaf mold, rotten wood); **Record Level:** institutionCode: CASC
- **Type status:** Other material. **Occurrence:** catalogNumber: hjr121(04)-4; recordedBy: H.J.Ratsirarson; sex: 1w; preparations: pin; associatedMedia: http://www.antweb.org/specimen/hjr121(04)-4; **Taxon:** scientificName: Stigmatomma tsyhady; genus: Stigmatomma; **Location:** country: Madagascar; stateProvince: Toamasina; locality: F.C. Andriantantely; verbatimElevation: 530; decimalLatitude: -18.695; decimalLongitude: 48.81333; **Event:** samplingProtocol: MW 25 sample transect, 5m; eventDate: 12/04/1998; habitat: rainforest; fieldNumber: HJR121; eventRemarks: sifted litter (leaf mold, rotten wood); **Record Level:** institutionCode: CASC
- **Type status:** Other material. **Occurrence:** catalogNumber: hjr121(06)-6; recordedBy: H.J.Ratsirarson; sex: 1w; preparations: pin; associatedMedia: http://www.antweb.org/specimen/hjr121(06)-6; **Taxon:** scientificName: Stigmatomma tsyhady; genus: Stigmatomma; **Location:** country: Madagascar; stateProvince: Toamasina; locality: F.C. Andriantantely; verbatimElevation: 530; decimalLatitude: -18.695; decimalLongitude: 48.81333; **Event:** samplingProtocol: MW 25 sample transect, 5m; eventDate: 12/04/1998; habitat: rainforest; fieldNumber: HJR121; eventRemarks: sifted litter (leaf mold, rotten wood); **Record Level:** institutionCode: CASC
- **Type status:** Other material. **Occurrence:** catalogNumber: hjr121(23)-4; recordedBy: H.J.Ratsirarson; sex: 1w; preparations: pin; associatedMedia: http://www.antweb.org/specimen/hjr121(23)-4; **Taxon:** scientificName: Stigmatomma tsyhady; genus: Stigmatomma; **Location:** country: Madagascar; stateProvince: Toamasina; locality: F.C. Andriantantely; verbatimElevation: 530; decimalLatitude: -18.695; decimalLongitude: 48.81333; **Event:** samplingProtocol: MW 25 sample transect, 5m; eventDate: 12/04/1998; habitat: rainforest; fieldNumber: HJR121; eventRemarks: sifted litter (leaf mold, rotten wood); **Record Level:** institutionCode: CASC
- **Type status:** Other material. **Occurrence:** catalogNumber: hjr122(32)-3; recordedBy: H.J.Ratsirarson; sex: 1w; preparations: pin; associatedMedia: http://www.antweb.org/specimen/hjr122(32)-3; **Taxon:** scientificName: Stigmatomma tsyhady; genus: Stigmatomma; **Location:** country: Madagascar; stateProvince: Toamasina; locality: F.C. Andriantantely; verbatimElevation: 530; decimalLatitude: -18.695; decimalLongitude: 48.81333; **Event:** samplingProtocol: MW 25 sample transect, 5m; eventDate: 12/07/1998; habitat: rainforest; fieldNumber: HJR122; eventRemarks: sifted litter (leaf mold, rotten wood); **Record Level:** institutionCode: CASC
- **Type status:** Other material. **Occurrence:** catalogNumber: hjr122(35)-3; recordedBy: H.J.Ratsirarson; sex: 1w; preparations: pin; associatedMedia: http://www.antweb.org/specimen/hjr122(35)-3; **Taxon:** scientificName: Stigmatomma tsyhady; genus: Stigmatomma; **Location:** country: Madagascar; stateProvince: Toamasina; locality: F.C. Andriantantely; verbatimElevation: 530; decimalLatitude: -18.695; decimalLongitude: 48.81333; **Event:** samplingProtocol: MW 25 sample transect, 5m; eventDate: 12/07/1998; habitat: rainforest; fieldNumber: HJR122; eventRemarks: sifted litter (leaf mold, rotten wood); **Record Level:** institutionCode: CASC
- **Type status:** Other material. **Occurrence:** catalogNumber: hjr122(36)-3; recordedBy: H.J.Ratsirarson; sex: 1w; preparations: pin; associatedMedia: http://www.antweb.org/specimen/hjr122(36)-3; **Taxon:** scientificName: Stigmatomma tsyhady; genus: Stigmatomma; **Location:** country: Madagascar; stateProvince: Toamasina; locality: F.C. Andriantantely; verbatimElevation: 530; decimalLatitude: -18.695; decimalLongitude: 48.81333; **Event:** samplingProtocol: MW 25 sample transect, 5m; eventDate: 12/07/1998; habitat: rainforest; fieldNumber: HJR122; eventRemarks: sifted litter (leaf mold, rotten wood); **Record Level:** institutionCode: CASC
- **Type status:** Other material. **Occurrence:** catalogNumber: casent0318419; recordedBy: H.J.Ratsirarson; sex: 1w; preparations: pin; associatedMedia: http://www.antweb.org/specimen/casent0318419; **Taxon:** scientificName: Stigmatomma tsyhady; genus: Stigmatomma; **Location:** country: Madagascar; stateProvince: Toamasina; locality: F.C. Sandranantitra; verbatimElevation: 450; decimalLatitude: -18.04833; decimalLongitude: 49.09167; **Event:** samplingProtocol: MW 25 sample transect, 5m; eventDate: 01/21/1999; habitat: rainforest; fieldNumber: HJR102; eventRemarks: sifted litter (leaf mold, rotten wood); **Record Level:** institutionCode: CASC
- **Type status:** Other material. **Occurrence:** catalogNumber: hjr101(15)-3; recordedBy: H.J.Ratsirarson; sex: 1w; preparations: pin; associatedMedia: http://www.antweb.org/specimen/hjr101(15)-3; **Taxon:** scientificName: Stigmatomma tsyhady; genus: Stigmatomma; **Location:** country: Madagascar; stateProvince: Toamasina; locality: F.C. Sandranantitra; verbatimElevation: 450; decimalLatitude: -18.04833; decimalLongitude: 49.09167; **Event:** samplingProtocol: MW 25 sample transect, 5m; eventDate: 01/18/1999; habitat: rainforest; fieldNumber: HJR101; eventRemarks: sifted litter (leaf mold, rotten wood); **Record Level:** institutionCode: CASC
- **Type status:** Other material. **Occurrence:** catalogNumber: hjr101(25)-5; recordedBy: H.J.Ratsirarson; sex: 1w; preparations: pin; associatedMedia: http://www.antweb.org/specimen/hjr101(25)-5; **Taxon:** scientificName: Stigmatomma tsyhady; genus: Stigmatomma; **Location:** country: Madagascar; stateProvince: Toamasina; locality: F.C. Sandranantitra; verbatimElevation: 450; decimalLatitude: -18.04833; decimalLongitude: 49.09167; **Event:** samplingProtocol: MW 25 sample transect, 5m; eventDate: 01/18/1999; habitat: rainforest; fieldNumber: HJR101; eventRemarks: sifted litter (leaf mold, rotten wood); **Record Level:** institutionCode: CASC
- **Type status:** Other material. **Occurrence:** catalogNumber: hjr102(45)-4; recordedBy: H.J.Ratsirarson; sex: 1w; preparations: pin; associatedMedia: http://www.antweb.org/specimen/hjr102(45)-4; **Taxon:** scientificName: Stigmatomma tsyhady; genus: Stigmatomma; **Location:** country: Madagascar; stateProvince: Toamasina; locality: F.C. Sandranantitra; verbatimElevation: 450; decimalLatitude: -18.04833; decimalLongitude: 49.09167; **Event:** samplingProtocol: MW 25 sample transect, 5m; eventDate: 01/21/1999; habitat: rainforest; fieldNumber: HJR102; eventRemarks: sifted litter (leaf mold, rotten wood); **Record Level:** institutionCode: CASC
- **Type status:** Other material. **Occurrence:** catalogNumber: casent0179498; recordedBy: H.J.Ratsirarson; sex: 2aQ; preparations: pin; associatedMedia: http://www.antweb.org/specimen/casent0179498; **Taxon:** scientificName: Stigmatomma tsyhady; genus: Stigmatomma; **Location:** country: Madagascar; stateProvince: Toamasina; locality: F.C. Sandranantitra; verbatimElevation: 450; decimalLatitude: -18.04833; decimalLongitude: 49.09167; **Event:** samplingProtocol: MW 25 sample transect, 5m; eventDate: 01/21/1999; habitat: rainforest; fieldNumber: HJR102; eventRemarks: sifted litter (leaf mold, rotten wood); **Record Level:** institutionCode: CASC
- **Type status:** Other material. **Occurrence:** catalogNumber: hjr102(32)-4; recordedBy: H.J.Ratsirarson; sex: 2w; preparations: pin; associatedMedia: http://www.antweb.org/specimen/hjr102(32)-4; **Taxon:** scientificName: Stigmatomma tsyhady; genus: Stigmatomma; **Location:** country: Madagascar; stateProvince: Toamasina; locality: F.C. Sandranantitra; verbatimElevation: 450; decimalLatitude: -18.04833; decimalLongitude: 49.09167; **Event:** samplingProtocol: MW 25 sample transect, 5m; eventDate: 01/21/1999; habitat: rainforest; fieldNumber: HJR102; eventRemarks: sifted litter (leaf mold, rotten wood); **Record Level:** institutionCode: CASC
- **Type status:** Other material. **Occurrence:** catalogNumber: hjr102(41)-2; recordedBy: H.J.Ratsirarson; sex: 2w; preparations: pin; associatedMedia: http://www.antweb.org/specimen/hjr102(41)-2; **Taxon:** scientificName: Stigmatomma tsyhady; genus: Stigmatomma; **Location:** country: Madagascar; stateProvince: Toamasina; locality: F.C. Sandranantitra; verbatimElevation: 450; decimalLatitude: -18.04833; decimalLongitude: 49.09167; **Event:** samplingProtocol: MW 25 sample transect, 5m; eventDate: 01/21/1999; habitat: rainforest; fieldNumber: HJR102; eventRemarks: sifted litter (leaf mold, rotten wood); **Record Level:** institutionCode: CASC
- **Type status:** Other material. **Occurrence:** catalogNumber: casent0053287; recordedBy: B.L.Fisher; sex: 1w; preparations: pin; associatedMedia: http://www.antweb.org/specimen/casent0053287; **Taxon:** scientificName: Stigmatomma tsyhady; genus: Stigmatomma; **Location:** country: Madagascar; stateProvince: Antsiranana; locality: Forêt Ambanitaza, 26.1 km 347° Antalaha; verbatimElevation: 240; decimalLatitude: -14.67933; decimalLongitude: 50.18367; georeferenceRemarks: coordinates obtained from GPS; **Event:** samplingProtocol: MW 25 sample transect, 5m; eventDate: 11/26/2004; habitat: rainforest; fieldNumber: BLF10997; eventRemarks: sifted litter (leaf mold, rotten wood); **Record Level:** institutionCode: CASC
- **Type status:** Other material. **Occurrence:** catalogNumber: casent0054671; recordedBy: B.L.Fisher; sex: 1w; preparations: pin; associatedMedia: http://www.antweb.org/specimen/casent0054671; **Taxon:** scientificName: Stigmatomma tsyhady; genus: Stigmatomma; **Location:** country: Madagascar; stateProvince: Antsiranana; locality: Forêt Ambanitaza, 26.1 km 347° Antalaha; verbatimElevation: 240; decimalLatitude: -14.67933; decimalLongitude: 50.18367; georeferenceRemarks: coordinates obtained from GPS; **Event:** samplingProtocol: MW 25 sample transect, 5m; eventDate: 11/26/2004; habitat: rainforest; fieldNumber: BLF10997; eventRemarks: sifted litter (leaf mold, rotten wood); **Record Level:** institutionCode: CASC
- **Type status:** Other material. **Occurrence:** catalogNumber: casent0054672; recordedBy: B.L.Fisher; sex: 1w; preparations: pin; associatedMedia: http://www.antweb.org/specimen/casent0054672; **Taxon:** scientificName: Stigmatomma tsyhady; genus: Stigmatomma; **Location:** country: Madagascar; stateProvince: Antsiranana; locality: Forêt Ambanitaza, 26.1 km 347° Antalaha; verbatimElevation: 240; decimalLatitude: -14.67933; decimalLongitude: 50.18367; georeferenceRemarks: coordinates obtained from GPS; **Event:** samplingProtocol: MW 25 sample transect, 5m; eventDate: 11/26/2004; habitat: rainforest; fieldNumber: BLF10997; eventRemarks: sifted litter (leaf mold, rotten wood); **Record Level:** institutionCode: CASC
- **Type status:** Other material. **Occurrence:** catalogNumber: casent0007088; recordedBy: Fisher, Griswold et al.; sex: 1w; preparations: pin; associatedMedia: http://www.antweb.org/specimen/casent0007088; **Taxon:** scientificName: Stigmatomma tsyhady; genus: Stigmatomma; **Location:** country: Madagascar; stateProvince: Toliara; locality: Forêt Classée d'Analavelona, 29.4 km 343° NNW Mahaboboka; verbatimElevation: 1050; decimalLatitude: -22.675; decimalLongitude: 44.18667; georeferenceRemarks: coordinates obtained from GPS; **Event:** samplingProtocol: MW 25 sample transect, 5m; eventDate: 02/21/2003; habitat: montane rainforest; fieldNumber: BLF07893; eventRemarks: sifted litter (leaf mold, rotten wood); **Record Level:** institutionCode: CASC
- **Type status:** Other material. **Occurrence:** catalogNumber: casent0007097; recordedBy: Fisher, Griswold et al.; sex: 1w; preparations: pin; associatedMedia: http://www.antweb.org/specimen/casent0007097; **Taxon:** scientificName: Stigmatomma tsyhady; genus: Stigmatomma; **Location:** country: Madagascar; stateProvince: Toliara; locality: Forêt Classée d'Analavelona, 29.4 km 343° NNW Mahaboboka; verbatimElevation: 1050; decimalLatitude: -22.675; decimalLongitude: 44.18667; georeferenceRemarks: coordinates obtained from GPS; **Event:** samplingProtocol: MW 25 sample transect, 5m; eventDate: 02/21/2003; habitat: montane rainforest; fieldNumber: BLF07893; eventRemarks: sifted litter (leaf mold, rotten wood); **Record Level:** institutionCode: CASC
- **Type status:** Other material. **Occurrence:** catalogNumber: casent0007098; recordedBy: Fisher, Griswold et al.; sex: 1w; preparations: pin; associatedMedia: http://www.antweb.org/specimen/casent0007098; **Taxon:** scientificName: Stigmatomma tsyhady; genus: Stigmatomma; **Location:** country: Madagascar; stateProvince: Toliara; locality: Forêt Classée d'Analavelona, 29.4 km 343° NNW Mahaboboka; verbatimElevation: 1050; decimalLatitude: -22.675; decimalLongitude: 44.18667; georeferenceRemarks: coordinates obtained from GPS; **Event:** samplingProtocol: MW 25 sample transect, 5m; eventDate: 02/21/2003; habitat: montane rainforest; fieldNumber: BLF07893; eventRemarks: sifted litter (leaf mold, rotten wood); **Record Level:** institutionCode: CASC
- **Type status:** Other material. **Occurrence:** catalogNumber: casent0042788; recordedBy: B.L.Fisher; sex: 1w; preparations: pin; associatedMedia: http://www.antweb.org/specimen/casent0042788; **Taxon:** scientificName: Stigmatomma tsyhady; genus: Stigmatomma; **Location:** country: Madagascar; stateProvince: Antsiranana; locality: Forêt de Binara, 9.1km 233° SW Daraina; verbatimElevation: 800; decimalLatitude: -13.26333; decimalLongitude: 49.60333; georeferenceRemarks: coordinates obtained from GPS; **Event:** samplingProtocol: MW 25 sample transect, 5m; eventDate: 12/03/2003; habitat: rainforest; fieldNumber: BLF09656; eventRemarks: sifted litter (leaf mold, rotten wood); **Record Level:** institutionCode: CASC
- **Type status:** Other material. **Occurrence:** catalogNumber: hjr111(05)-3; recordedBy: H.J.Ratsirarson; sex: 1dQ; preparations: pin; associatedMedia: http://www.antweb.org/specimen/hjr111(05)-3; **Taxon:** scientificName: Stigmatomma tsyhady; genus: Stigmatomma; **Location:** country: Madagascar; stateProvince: Toamasina; locality: P.N. Mantadia; verbatimElevation: 895; decimalLatitude: -18.79167; decimalLongitude: 48.42667; **Event:** samplingProtocol: MW 25 sample transect, 5m; eventDate: 11/25/1998; habitat: rainforest; fieldNumber: HJR111; eventRemarks: sifted litter (leaf mold, rotten wood); **Record Level:** institutionCode: CASC
- **Type status:** Other material. **Occurrence:** catalogNumber: casent0044919; recordedBy: B.L.Fisher et al.; sex: 1w; preparations: pin; associatedMedia: http://www.antweb.org/specimen/casent0044919; **Taxon:** scientificName: Stigmatomma tsyhady; genus: Stigmatomma; **Location:** country: Madagascar; stateProvince: Antsiranana; locality: Parc National de Marojejy, Manantenina River, 28.0 km 38° NE Andapa, 8.2 km 333° NNW Manantenina; verbatimElevation: 450; decimalLatitude: -14.43667; decimalLongitude: 49.775; georeferenceRemarks: coordinates obtained from GPS; **Event:** samplingProtocol: MW 25 sample transect, 5m; eventDate: 11/12/2003; habitat: rainforest; fieldNumber: BLF08722; eventRemarks: sifted litter (leaf mold, rotten wood); **Record Level:** institutionCode: CASC
- **Type status:** Other material. **Occurrence:** catalogNumber: casent0044920; recordedBy: B.L.Fisher et al.; sex: 1w; preparations: pin; associatedMedia: http://www.antweb.org/specimen/casent0044920; **Taxon:** scientificName: Stigmatomma tsyhady; genus: Stigmatomma; **Location:** country: Madagascar; stateProvince: Antsiranana; locality: Parc National de Marojejy, Manantenina River, 28.0 km 38° NE Andapa, 8.2 km 333° NNW Manantenina; verbatimElevation: 450; decimalLatitude: -14.43667; decimalLongitude: 49.775; georeferenceRemarks: coordinates obtained from GPS; **Event:** samplingProtocol: MW 25 sample transect, 5m; eventDate: 11/12/2003; habitat: rainforest; fieldNumber: BLF08722; eventRemarks: sifted litter (leaf mold, rotten wood); **Record Level:** institutionCode: CASC
- **Type status:** Other material. **Occurrence:** catalogNumber: casent0044921; recordedBy: B.L.Fisher et al.; sex: 1w; preparations: pin; associatedMedia: http://www.antweb.org/specimen/casent0044921; **Taxon:** scientificName: Stigmatomma tsyhady; genus: Stigmatomma; **Location:** country: Madagascar; stateProvince: Antsiranana; locality: Parc National de Marojejy, Manantenina River, 28.0 km 38° NE Andapa, 8.2 km 333° NNW Manantenina; verbatimElevation: 450; decimalLatitude: -14.43667; decimalLongitude: 49.775; georeferenceRemarks: coordinates obtained from GPS; **Event:** samplingProtocol: MW 25 sample transect, 5m; eventDate: 11/12/2003; habitat: rainforest; fieldNumber: BLF08722; eventRemarks: sifted litter (leaf mold, rotten wood); **Record Level:** institutionCode: CASC
- **Type status:** Other material. **Occurrence:** catalogNumber: casent0044922; recordedBy: B.L.Fisher et al.; sex: 1w; preparations: pin; associatedMedia: http://www.antweb.org/specimen/casent0044922; **Taxon:** scientificName: Stigmatomma tsyhady; genus: Stigmatomma; **Location:** country: Madagascar; stateProvince: Antsiranana; locality: Parc National de Marojejy, Manantenina River, 28.0 km 38° NE Andapa, 8.2 km 333° NNW Manantenina; verbatimElevation: 450; decimalLatitude: -14.43667; decimalLongitude: 49.775; georeferenceRemarks: coordinates obtained from GPS; **Event:** samplingProtocol: MW 25 sample transect, 5m; eventDate: 11/12/2003; habitat: rainforest; fieldNumber: BLF08722; eventRemarks: sifted litter (leaf mold, rotten wood); **Record Level:** institutionCode: CASC
- **Type status:** Other material. **Occurrence:** catalogNumber: casent0044924; recordedBy: B.L.Fisher et al.; sex: 1w; preparations: pin; associatedMedia: http://www.antweb.org/specimen/casent0044924; **Taxon:** scientificName: Stigmatomma tsyhady; genus: Stigmatomma; **Location:** country: Madagascar; stateProvince: Antsiranana; locality: Parc National de Marojejy, Manantenina River, 28.0 km 38° NE Andapa, 8.2 km 333° NNW Manantenina; verbatimElevation: 450; decimalLatitude: -14.43667; decimalLongitude: 49.775; georeferenceRemarks: coordinates obtained from GPS; **Event:** samplingProtocol: MW 25 sample transect, 5m; eventDate: 11/12/2003; habitat: rainforest; fieldNumber: BLF08722; eventRemarks: sifted litter (leaf mold, rotten wood); **Record Level:** institutionCode: CASC
- **Type status:** Other material. **Occurrence:** catalogNumber: blf0760(02)-1; recordedBy: B.L.Fisher; sex: 3w; preparations: pin; associatedMedia: http://www.antweb.org/specimen/blf0760(02)-1; **Taxon:** scientificName: Stigmatomma tsyhady; genus: Stigmatomma; **Location:** country: Madagascar; stateProvince: Fianarantsoa; locality: 43 km S Ambalavao, Rés. Andringitra; verbatimElevation: 825; decimalLatitude: -22.23333; decimalLongitude: 47; **Event:** samplingProtocol: MW 3 sample transect, 5m; eventDate: 10/09/1993; habitat: rainforest; fieldNumber: BLF00760; eventRemarks: sifted litter (leaf mold, rotten wood); **Record Level:** institutionCode: CASC
- **Type status:** Other material. **Occurrence:** catalogNumber: blf0492(24)-1; recordedBy: B.L.Fisher; sex: 1dQ, 2w; preparations: pin; associatedMedia: http://www.antweb.org/specimen/blf0492(24)-1; **Taxon:** scientificName: Stigmatomma tsyhady; genus: Stigmatomma; **Location:** country: Madagascar; stateProvince: Toliara; locality: 11 km NW Enakara, Rés. Andohahela; verbatimElevation: 800; decimalLatitude: -24.56667; decimalLongitude: 46.83333; **Event:** samplingProtocol: MW 50 sample transect, 5m; eventDate: 11/17/1992; habitat: rainforest; fieldNumber: BLF00492; eventRemarks: sifted litter (leaf mold, rotten wood); **Record Level:** institutionCode: CASC
- **Type status:** Other material. **Occurrence:** catalogNumber: blf0492(12)-1; recordedBy: B.L.Fisher; sex: 1w; preparations: pin; associatedMedia: http://www.antweb.org/specimen/blf0492(12)-1; **Taxon:** scientificName: Stigmatomma tsyhady; genus: Stigmatomma; **Location:** country: Madagascar; stateProvince: Toliara; locality: 11 km NW Enakara, Rés. Andohahela; verbatimElevation: 800; decimalLatitude: -24.56667; decimalLongitude: 46.83333; **Event:** samplingProtocol: MW 50 sample transect, 5m; eventDate: 11/17/1992; habitat: rainforest; fieldNumber: BLF00492; eventRemarks: sifted litter (leaf mold, rotten wood); **Record Level:** institutionCode: CASC
- **Type status:** Other material. **Occurrence:** catalogNumber: blf0492(17)-1; recordedBy: B.L.Fisher; sex: 2w; preparations: pin; associatedMedia: http://www.antweb.org/specimen/blf0492(17)-1; **Taxon:** scientificName: Stigmatomma tsyhady; genus: Stigmatomma; **Location:** country: Madagascar; stateProvince: Toliara; locality: 11 km NW Enakara, Rés. Andohahela; verbatimElevation: 800; decimalLatitude: -24.56667; decimalLongitude: 46.83333; **Event:** samplingProtocol: MW 50 sample transect, 5m; eventDate: 11/17/1992; habitat: rainforest; fieldNumber: BLF00492; eventRemarks: sifted litter (leaf mold, rotten wood); **Record Level:** institutionCode: CASC
- **Type status:** Other material. **Occurrence:** catalogNumber: blf2102(48)-2; recordedBy: B.L.Fisher (J.-Baptiste); sex: 1dQ; preparations: pin; associatedMedia: http://www.antweb.org/specimen/blf2102(48)-2; **Taxon:** scientificName: Stigmatomma tsyhady; genus: Stigmatomma; **Location:** country: Madagascar; stateProvince: Toliara; locality: 2.7 km WNW 302° Ste. Luce; verbatimElevation: 20; decimalLatitude: -24.77167; decimalLongitude: 47.17167; **Event:** samplingProtocol: MW 50 sample transect, 5m; eventDate: 12/09/1998; habitat: littoral rainforest; fieldNumber: BLF02102; eventRemarks: sifted litter (leaf mold, rotten wood); **Record Level:** institutionCode: CASC
- **Type status:** Other material. **Occurrence:** catalogNumber: blf2102(09)-1; recordedBy: B.L.Fisher (J.-Baptiste); sex: 1w; preparations: pin; associatedMedia: http://www.antweb.org/specimen/blf2102(09)-1; **Taxon:** scientificName: Stigmatomma tsyhady; genus: Stigmatomma; **Location:** country: Madagascar; stateProvince: Toliara; locality: 2.7 km WNW 302° Ste. Luce; verbatimElevation: 20; decimalLatitude: -24.77167; decimalLongitude: 47.17167; **Event:** samplingProtocol: MW 50 sample transect, 5m; eventDate: 12/09/1998; habitat: littoral rainforest; fieldNumber: BLF02102; eventRemarks: sifted litter (leaf mold, rotten wood); **Record Level:** institutionCode: CASC
- **Type status:** Other material. **Occurrence:** catalogNumber: blf2102(30)-01; recordedBy: B.L.Fisher (J.-Baptiste); sex: 1w; preparations: pin; associatedMedia: http://www.antweb.org/specimen/blf2102(30)-01; **Taxon:** scientificName: Stigmatomma tsyhady; genus: Stigmatomma; **Location:** country: Madagascar; stateProvince: Toliara; locality: 2.7 km WNW 302° Ste. Luce; verbatimElevation: 20; decimalLatitude: -24.77167; decimalLongitude: 47.17167; **Event:** samplingProtocol: MW 50 sample transect, 5m; eventDate: 12/09/1998; habitat: littoral rainforest; fieldNumber: BLF02102; eventRemarks: sifted litter (leaf mold, rotten wood); **Record Level:** institutionCode: CASC
- **Type status:** Other material. **Occurrence:** catalogNumber: casent0318420; recordedBy: B.L.Fisher (J.-Baptiste); sex: 1w; preparations: pin; associatedMedia: http://www.antweb.org/specimen/casent0318420; **Taxon:** scientificName: Stigmatomma tsyhady; genus: Stigmatomma; **Location:** country: Madagascar; stateProvince: Toliara; locality: 2.7 km WNW 302° Ste. Luce; verbatimElevation: 20; decimalLatitude: -24.77167; decimalLongitude: 47.17167; **Event:** samplingProtocol: MW 50 sample transect, 5m; eventDate: 12/09/1998; habitat: littoral rainforest; fieldNumber: BLF02102; eventRemarks: sifted litter (leaf mold, rotten wood); **Record Level:** institutionCode: CASC
- **Type status:** Other material. **Occurrence:** catalogNumber: blf0926(41)-3; recordedBy: B.L.Fisher; sex: 1w; preparations: pin; associatedMedia: http://www.antweb.org/specimen/blf0926(41)-3; **Taxon:** scientificName: Stigmatomma tsyhady; genus: Stigmatomma; **Location:** country: Madagascar; stateProvince: Toamasina; locality: 5.3 km SSE Ambanizana, Andranobe; verbatimElevation: 425; decimalLatitude: -15.67133; decimalLongitude: 49.97395; **Event:** samplingProtocol: MW 50 sample transect, 5m; eventDate: 11/21/1993; habitat: rainforest; fieldNumber: BLF00926; eventRemarks: sifted litter (leaf mold, rotten wood); **Record Level:** institutionCode: CASC
- **Type status:** Other material. **Occurrence:** catalogNumber: casent0318412; recordedBy: B.L.Fisher; sex: 1w; preparations: pin; associatedMedia: http://www.antweb.org/specimen/casent0318412; **Taxon:** scientificName: Stigmatomma tsyhady; genus: Stigmatomma; **Location:** country: Madagascar; stateProvince: Toamasina; locality: 5.3 km SSE Ambanizana, Andranobe; verbatimElevation: 425; decimalLatitude: -15.67133; decimalLongitude: 49.97395; **Event:** samplingProtocol: MW 50 sample transect, 5m; eventDate: 11/21/1993; habitat: rainforest; fieldNumber: BLF00926; eventRemarks: sifted litter (leaf mold, rotten wood); **Record Level:** institutionCode: CASC
- **Type status:** Other material. **Occurrence:** catalogNumber: blf1757(01)-7; recordedBy: B.L.Fisher (Sylvain); sex: 1w; preparations: pin; associatedMedia: http://www.antweb.org/specimen/blf1757(01)-7; **Taxon:** scientificName: Stigmatomma tsyhady; genus: Stigmatomma; **Location:** country: Madagascar; stateProvince: Fianarantsoa; locality: 9.0 km NE Ivohibe; verbatimElevation: 900; decimalLatitude: -22.42667; decimalLongitude: 46.93833; **Event:** samplingProtocol: MW 50 sample transect, 5m; eventDate: 11/12/1997; habitat: rainforest; fieldNumber: BLF01757; eventRemarks: sifted litter (leaf mold, rotten wood); **Record Level:** institutionCode: CASC
- **Type status:** Other material. **Occurrence:** catalogNumber: blf1757(13)-7; recordedBy: B.L.Fisher (Sylvain); sex: 1w; preparations: pin; associatedMedia: http://www.antweb.org/specimen/blf1757(13)-7; **Taxon:** scientificName: Stigmatomma tsyhady; genus: Stigmatomma; **Location:** country: Madagascar; stateProvince: Fianarantsoa; locality: 9.0 km NE Ivohibe; verbatimElevation: 900; decimalLatitude: -22.42667; decimalLongitude: 46.93833; **Event:** samplingProtocol: MW 50 sample transect, 5m; eventDate: 11/12/1997; habitat: rainforest; fieldNumber: BLF01757; eventRemarks: sifted litter (leaf mold, rotten wood); **Record Level:** institutionCode: CASC
- **Type status:** Other material. **Occurrence:** catalogNumber: blf1757(24)-9; recordedBy: B.L.Fisher (Sylvain); sex: 1w; preparations: pin; associatedMedia: http://www.antweb.org/specimen/blf1757(24)-9; **Taxon:** scientificName: Stigmatomma tsyhady; genus: Stigmatomma; **Location:** country: Madagascar; stateProvince: Fianarantsoa; locality: 9.0 km NE Ivohibe; verbatimElevation: 900; decimalLatitude: -22.42667; decimalLongitude: 46.93833; **Event:** samplingProtocol: MW 50 sample transect, 5m; eventDate: 11/12/1997; habitat: rainforest; fieldNumber: BLF01757; eventRemarks: sifted litter (leaf mold, rotten wood); **Record Level:** institutionCode: CASC
- **Type status:** Other material. **Occurrence:** catalogNumber: blf1757(31)-1; recordedBy: B.L.Fisher (Sylvain); sex: 1w; preparations: pin; associatedMedia: http://www.antweb.org/specimen/blf1757(31)-1; **Taxon:** scientificName: Stigmatomma tsyhady; genus: Stigmatomma; **Location:** country: Madagascar; stateProvince: Fianarantsoa; locality: 9.0 km NE Ivohibe; verbatimElevation: 900; decimalLatitude: -22.42667; decimalLongitude: 46.93833; **Event:** samplingProtocol: MW 50 sample transect, 5m; eventDate: 11/12/1997; habitat: rainforest; fieldNumber: BLF01757; eventRemarks: sifted litter (leaf mold, rotten wood); **Record Level:** institutionCode: CASC
- **Type status:** Other material. **Occurrence:** catalogNumber: casent0030797; recordedBy: Fisher, Griswold et al.; sex: 1w; preparations: pin; associatedMedia: http://www.antweb.org/specimen/casent0030797; **Taxon:** scientificName: Stigmatomma tsyhady; genus: Stigmatomma; **Location:** country: Madagascar; stateProvince: Toliara; locality: Forêt Classée d'Analavelona, 29.2 km 343° NNW Mahaboboka; verbatimElevation: 1100; decimalLatitude: -22.675; decimalLongitude: 44.19; georeferenceRemarks: coordinates obtained from GPS; **Event:** samplingProtocol: MW 50 sample transect, 5m; eventDate: 02/18/2003; habitat: montane rainforest; fieldNumber: BLF07820; eventRemarks: sifted litter (leaf mold, rotten wood); **Record Level:** institutionCode: CASC
- **Type status:** Other material. **Occurrence:** catalogNumber: blf2072(35)-50; recordedBy: B.L.Fisher; sex: 2w; preparations: pin; associatedMedia: http://www.antweb.org/specimen/blf2072(35)-50; **Taxon:** scientificName: Stigmatomma tsyhady; genus: Stigmatomma; **Location:** country: Madagascar; stateProvince: Toliara; locality: Forêt de Petriky, 12.5 km W 272° Tolagnaro; verbatimElevation: 10; decimalLatitude: -25.06167; decimalLongitude: 46.87; **Event:** samplingProtocol: MW 50 sample transect, 5m; eventDate: 11/22/1998; habitat: littoral rainforest; fieldNumber: BLF02072; eventRemarks: sifted litter (leaf mold, rotten wood); **Record Level:** institutionCode: CASC
- **Type status:** Other material. **Occurrence:** catalogNumber: casent0170204; recordedBy: B.L.Fisher; sex: 2w; preparations: pin; associatedMedia: http://www.antweb.org/specimen/casent0170204; **Taxon:** scientificName: Stigmatomma tsyhady; genus: Stigmatomma; **Location:** country: Madagascar; stateProvince: Toliara; locality: Forêt de Petriky, 12.5 km W 272° Tolagnaro; verbatimElevation: 10; decimalLatitude: -25.06167; decimalLongitude: 46.87; **Event:** samplingProtocol: MW 50 sample transect, 5m; eventDate: 11/22/1998; habitat: littoral rainforest; fieldNumber: BLF02072; eventRemarks: sifted litter (leaf mold, rotten wood); **Record Level:** institutionCode: CASC
- **Type status:** Other material. **Occurrence:** catalogNumber: casent0034840; recordedBy: Fisher, Griswold et al.; sex: 1w; preparations: pin; associatedMedia: http://www.antweb.org/specimen/casent0034840; **Taxon:** scientificName: Stigmatomma tsyhady; genus: Stigmatomma; **Location:** country: Madagascar; stateProvince: Fianarantsoa; locality: Parc National de Ranomafana, Vatoharanana River, 4.1 km 231° SW Ranomafana; verbatimElevation: 1100; decimalLatitude: -21.29; decimalLongitude: 47.43333; georeferenceRemarks: coordinates obtained from GPS; **Event:** samplingProtocol: MW 50 sample transect, 5m; eventDate: 03/27/2003; habitat: montane rainforest; fieldNumber: BLF08400; eventRemarks: sifted litter (leaf mold, rotten wood); **Record Level:** institutionCode: CASC
- **Type status:** Other material. **Occurrence:** catalogNumber: casent0030364; recordedBy: Fisher, Griswold et al.; sex: 1w; preparations: pin; associatedMedia: http://www.antweb.org/specimen/casent0030364; **Taxon:** scientificName: Stigmatomma tsyhady; genus: Stigmatomma; **Location:** country: Madagascar; stateProvince: Fianarantsoa; locality: Parc National d'Isalo, Sahanafa River, 29.2 km 351° N Ranohira; verbatimElevation: 500; decimalLatitude: -22.31333; decimalLongitude: 45.29167; georeferenceRemarks: coordinates obtained from GPS; **Event:** samplingProtocol: MW 50 sample transect, 5m; eventDate: 02/10/2003; habitat: gallery forest; fieldNumber: BLF07651; eventRemarks: sifted litter (leaf mold, rotten wood); **Record Level:** institutionCode: CASC
- **Type status:** Other material. **Occurrence:** catalogNumber: casent0030365; recordedBy: Fisher, Griswold et al.; sex: 1w; preparations: pin; associatedMedia: http://www.antweb.org/specimen/casent0030365; **Taxon:** scientificName: Stigmatomma tsyhady; genus: Stigmatomma; **Location:** country: Madagascar; stateProvince: Fianarantsoa; locality: Parc National d'Isalo, Sahanafa River, 29.2 km 351° N Ranohira; verbatimElevation: 500; decimalLatitude: -22.31333; decimalLongitude: 45.29167; georeferenceRemarks: coordinates obtained from GPS; **Event:** samplingProtocol: MW 50 sample transect, 5m; eventDate: 02/10/2003; habitat: gallery forest; fieldNumber: BLF07651; eventRemarks: sifted litter (leaf mold, rotten wood); **Record Level:** institutionCode: CASC
- **Type status:** Other material. **Occurrence:** catalogNumber: casent0030366; recordedBy: Fisher, Griswold et al.; sex: 1w; preparations: pin; associatedMedia: http://www.antweb.org/specimen/casent0030366; **Taxon:** scientificName: Stigmatomma tsyhady; genus: Stigmatomma; **Location:** country: Madagascar; stateProvince: Fianarantsoa; locality: Parc National d'Isalo, Sahanafa River, 29.2 km 351° N Ranohira; verbatimElevation: 500; decimalLatitude: -22.31333; decimalLongitude: 45.29167; georeferenceRemarks: coordinates obtained from GPS; **Event:** samplingProtocol: MW 50 sample transect, 5m; eventDate: 02/10/2003; habitat: gallery forest; fieldNumber: BLF07651; eventRemarks: sifted litter (leaf mold, rotten wood); **Record Level:** institutionCode: CASC
- **Type status:** Other material. **Occurrence:** catalogNumber: casent0030367; recordedBy: Fisher, Griswold et al.; sex: 1w; preparations: pin; associatedMedia: http://www.antweb.org/specimen/casent0030367; **Taxon:** scientificName: Stigmatomma tsyhady; genus: Stigmatomma; **Location:** country: Madagascar; stateProvince: Fianarantsoa; locality: Parc National d'Isalo, Sahanafa River, 29.2 km 351° N Ranohira; verbatimElevation: 500; decimalLatitude: -22.31333; decimalLongitude: 45.29167; georeferenceRemarks: coordinates obtained from GPS; **Event:** samplingProtocol: MW 50 sample transect, 5m; eventDate: 02/10/2003; habitat: gallery forest; fieldNumber: BLF07651; eventRemarks: sifted litter (leaf mold, rotten wood); **Record Level:** institutionCode: CASC
- **Type status:** Other material. **Occurrence:** catalogNumber: casent0030368; recordedBy: Fisher, Griswold et al.; sex: 1w; preparations: pin; associatedMedia: http://www.antweb.org/specimen/casent0030368; **Taxon:** scientificName: Stigmatomma tsyhady; genus: Stigmatomma; **Location:** country: Madagascar; stateProvince: Fianarantsoa; locality: Parc National d'Isalo, Sahanafa River, 29.2 km 351° N Ranohira; verbatimElevation: 500; decimalLatitude: -22.31333; decimalLongitude: 45.29167; georeferenceRemarks: coordinates obtained from GPS; **Event:** samplingProtocol: MW 50 sample transect, 5m; eventDate: 02/10/2003; habitat: gallery forest; fieldNumber: BLF07651; eventRemarks: sifted litter (leaf mold, rotten wood); **Record Level:** institutionCode: CASC
- **Type status:** Other material. **Occurrence:** catalogNumber: casent0030369; recordedBy: Fisher, Griswold et al.; sex: 1w; preparations: pin; associatedMedia: http://www.antweb.org/specimen/casent0030369; **Taxon:** scientificName: Stigmatomma tsyhady; genus: Stigmatomma; **Location:** country: Madagascar; stateProvince: Fianarantsoa; locality: Parc National d'Isalo, Sahanafa River, 29.2 km 351° N Ranohira; verbatimElevation: 500; decimalLatitude: -22.31333; decimalLongitude: 45.29167; georeferenceRemarks: coordinates obtained from GPS; **Event:** samplingProtocol: MW 50 sample transect, 5m; eventDate: 02/10/2003; habitat: gallery forest; fieldNumber: BLF07651; eventRemarks: sifted litter (leaf mold, rotten wood); **Record Level:** institutionCode: CASC
- **Type status:** Other material. **Occurrence:** catalogNumber: casent0030370; recordedBy: Fisher, Griswold et al.; sex: 1w; preparations: pin; associatedMedia: http://www.antweb.org/specimen/casent0030370; **Taxon:** scientificName: Stigmatomma tsyhady; genus: Stigmatomma; **Location:** country: Madagascar; stateProvince: Fianarantsoa; locality: Parc National d'Isalo, Sahanafa River, 29.2 km 351° N Ranohira; verbatimElevation: 500; decimalLatitude: -22.31333; decimalLongitude: 45.29167; georeferenceRemarks: coordinates obtained from GPS; **Event:** samplingProtocol: MW 50 sample transect, 5m; eventDate: 02/10/2003; habitat: gallery forest; fieldNumber: BLF07651; eventRemarks: sifted litter (leaf mold, rotten wood); **Record Level:** institutionCode: CASC
- **Type status:** Other material. **Occurrence:** catalogNumber: casent0030371; recordedBy: Fisher, Griswold et al.; sex: 1w; preparations: pin; associatedMedia: http://www.antweb.org/specimen/casent0030371; **Taxon:** scientificName: Stigmatomma tsyhady; genus: Stigmatomma; **Location:** country: Madagascar; stateProvince: Fianarantsoa; locality: Parc National d'Isalo, Sahanafa River, 29.2 km 351° N Ranohira; verbatimElevation: 500; decimalLatitude: -22.31333; decimalLongitude: 45.29167; georeferenceRemarks: coordinates obtained from GPS; **Event:** samplingProtocol: MW 50 sample transect, 5m; eventDate: 02/10/2003; habitat: gallery forest; fieldNumber: BLF07651; eventRemarks: sifted litter (leaf mold, rotten wood); **Record Level:** institutionCode: CASC
- **Type status:** Other material. **Occurrence:** catalogNumber: casent0030372; recordedBy: Fisher, Griswold et al.; sex: 1w; preparations: pin; associatedMedia: http://www.antweb.org/specimen/casent0030372; **Taxon:** scientificName: Stigmatomma tsyhady; genus: Stigmatomma; **Location:** country: Madagascar; stateProvince: Fianarantsoa; locality: Parc National d'Isalo, Sahanafa River, 29.2 km 351° N Ranohira; verbatimElevation: 500; decimalLatitude: -22.31333; decimalLongitude: 45.29167; georeferenceRemarks: coordinates obtained from GPS; **Event:** samplingProtocol: MW 50 sample transect, 5m; eventDate: 02/10/2003; habitat: gallery forest; fieldNumber: BLF07651; eventRemarks: sifted litter (leaf mold, rotten wood); **Record Level:** institutionCode: CASC
- **Type status:** Other material. **Occurrence:** catalogNumber: casent0030373; recordedBy: Fisher, Griswold et al.; sex: 1w; preparations: pin; associatedMedia: http://www.antweb.org/specimen/casent0030373; **Taxon:** scientificName: Stigmatomma tsyhady; genus: Stigmatomma; **Location:** country: Madagascar; stateProvince: Fianarantsoa; locality: Parc National d'Isalo, Sahanafa River, 29.2 km 351° N Ranohira; verbatimElevation: 500; decimalLatitude: -22.31333; decimalLongitude: 45.29167; georeferenceRemarks: coordinates obtained from GPS; **Event:** samplingProtocol: MW 50 sample transect, 5m; eventDate: 02/10/2003; habitat: gallery forest; fieldNumber: BLF07651; eventRemarks: sifted litter (leaf mold, rotten wood); **Record Level:** institutionCode: CASC
- **Type status:** Other material. **Occurrence:** catalogNumber: casent0031371; recordedBy: Fisher, Griswold et al.; sex: 1w; preparations: pin; associatedMedia: http://www.antweb.org/specimen/casent0031371; **Taxon:** scientificName: Stigmatomma tsyhady; genus: Stigmatomma; **Location:** country: Madagascar; stateProvince: Fianarantsoa; locality: Parc National d'Isalo, Sahanafa River, 29.2 km 351° N Ranohira; verbatimElevation: 500; decimalLatitude: -22.31333; decimalLongitude: 45.29167; georeferenceRemarks: coordinates obtained from GPS; **Event:** samplingProtocol: MW 50 sample transect, 5m; eventDate: 02/10/2003; habitat: gallery forest; fieldNumber: BLF07651; eventRemarks: sifted litter (leaf mold, rotten wood); **Record Level:** institutionCode: CASC
- **Type status:** Other material. **Occurrence:** catalogNumber: casent0031592; recordedBy: Fisher, Griswold et al.; sex: 1w; preparations: pin; associatedMedia: http://www.antweb.org/specimen/casent0031592; **Taxon:** scientificName: Stigmatomma tsyhady; genus: Stigmatomma; **Location:** country: Madagascar; stateProvince: Fianarantsoa; locality: Parc National d'Isalo, Sahanafa River, 29.2 km 351° N Ranohira; verbatimElevation: 500; decimalLatitude: -22.31333; decimalLongitude: 45.29167; georeferenceRemarks: coordinates obtained from GPS; **Event:** samplingProtocol: MW 50 sample transect, 5m; eventDate: 02/10/2003; habitat: gallery forest; fieldNumber: BLF07651; eventRemarks: sifted litter (leaf mold, rotten wood); **Record Level:** institutionCode: CASC
- **Type status:** Other material. **Occurrence:** catalogNumber: casent0031593; recordedBy: Fisher, Griswold et al.; sex: 1w; preparations: pin; associatedMedia: http://www.antweb.org/specimen/casent0031593; **Taxon:** scientificName: Stigmatomma tsyhady; genus: Stigmatomma; **Location:** country: Madagascar; stateProvince: Fianarantsoa; locality: Parc National d'Isalo, Sahanafa River, 29.2 km 351° N Ranohira; verbatimElevation: 500; decimalLatitude: -22.31333; decimalLongitude: 45.29167; georeferenceRemarks: coordinates obtained from GPS; **Event:** samplingProtocol: MW 50 sample transect, 5m; eventDate: 02/10/2003; habitat: gallery forest; fieldNumber: BLF07651; eventRemarks: sifted litter (leaf mold, rotten wood); **Record Level:** institutionCode: CASC
- **Type status:** Other material. **Occurrence:** catalogNumber: casent0031594; recordedBy: Fisher, Griswold et al.; sex: 2w; preparations: pin; associatedMedia: http://www.antweb.org/specimen/casent0031594; **Taxon:** scientificName: Stigmatomma tsyhady; genus: Stigmatomma; **Location:** country: Madagascar; stateProvince: Fianarantsoa; locality: Parc National d'Isalo, Sahanafa River, 29.2 km 351° N Ranohira; verbatimElevation: 500; decimalLatitude: -22.31333; decimalLongitude: 45.29167; georeferenceRemarks: coordinates obtained from GPS; **Event:** samplingProtocol: MW 50 sample transect, 5m; eventDate: 02/10/2003; habitat: gallery forest; fieldNumber: BLF07651; eventRemarks: sifted litter (leaf mold, rotten wood); **Record Level:** institutionCode: CASC
- **Type status:** Other material. **Occurrence:** catalogNumber: blf1745(07)-7; recordedBy: B.L.Fisher (Sylvain); sex: 1w; preparations: pin; associatedMedia: http://www.antweb.org/specimen/blf1745(07)-7; **Taxon:** scientificName: Stigmatomma tsyhady; genus: Stigmatomma; **Location:** country: Madagascar; stateProvince: Fianarantsoa; locality: R.S. Ivohibe, 7.5 km ENE Ivohibe; verbatimElevation: 900; decimalLatitude: -22.47; decimalLongitude: 46.96; **Event:** samplingProtocol: MW 50 sample transect, 5m; eventDate: 10/07/1997; habitat: rainforest; fieldNumber: BLF01745; eventRemarks: sifted litter (leaf mold, rotten wood); **Record Level:** institutionCode: CASC
- **Type status:** Other material. **Occurrence:** catalogNumber: blf1745(22)-1; recordedBy: B.L.Fisher (Sylvain); sex: 1w; preparations: pin; associatedMedia: http://www.antweb.org/specimen/blf1745(22)-1; **Taxon:** scientificName: Stigmatomma tsyhady; genus: Stigmatomma; **Location:** country: Madagascar; stateProvince: Fianarantsoa; locality: R.S. Ivohibe, 7.5 km ENE Ivohibe; verbatimElevation: 900; decimalLatitude: -22.47; decimalLongitude: 46.96; **Event:** samplingProtocol: MW 50 sample transect, 5m; eventDate: 10/07/1997; habitat: rainforest; fieldNumber: BLF01745; eventRemarks: sifted litter (leaf mold, rotten wood); **Record Level:** institutionCode: CASC
- **Type status:** Other material. **Occurrence:** catalogNumber: casent0008712; recordedBy: B.L.Fisher (Sylvain); sex: 1w; preparations: pin; associatedMedia: http://www.antweb.org/specimen/casent0008712; **Taxon:** scientificName: Stigmatomma tsyhady; genus: Stigmatomma; **Location:** country: Madagascar; stateProvince: Fianarantsoa; locality: R.S. Ivohibe, 7.5 km ENE Ivohibe; verbatimElevation: 900; decimalLatitude: -22.47; decimalLongitude: 46.96; **Event:** samplingProtocol: MW 50 sample transect, 5m; eventDate: 10/07/1997; habitat: rainforest; fieldNumber: BLF01745; eventRemarks: sifted litter (leaf mold, rotten wood); **Record Level:** institutionCode: CASC
- **Type status:** Other material. **Occurrence:** catalogNumber: casent0028767; recordedBy: Fisher, Griswold et al.; sex: 1w; preparations: pin; associatedMedia: http://www.antweb.org/specimen/casent0028767; **Taxon:** scientificName: Stigmatomma tsyhady; genus: Stigmatomma; **Location:** country: Madagascar; stateProvince: Toliara; locality: Réserve Spéciale d'Ambohijanahary, Forêt d'Ankazotsihitafototra, 34.6 km 314° NW Ambaravaranala; verbatimElevation: 1100; decimalLatitude: -18.26; decimalLongitude: 45.41833; georeferenceRemarks: coordinates obtained from GPS; **Event:** samplingProtocol: MW 50 sample transect, 5m; eventDate: 01/16/2003; habitat: montane rainforest; fieldNumber: BLF07086; eventRemarks: sifted litter (leaf mold, rotten wood); **Record Level:** institutionCode: CASC
- **Type status:** Other material. **Occurrence:** catalogNumber: casent0028768; recordedBy: Fisher, Griswold et al.; sex: 1w; preparations: pin; associatedMedia: http://www.antweb.org/specimen/casent0028768; **Taxon:** scientificName: Stigmatomma tsyhady; genus: Stigmatomma; **Location:** country: Madagascar; stateProvince: Toliara; locality: Réserve Spéciale d'Ambohijanahary, Forêt d'Ankazotsihitafototra, 34.6 km 314° NW Ambaravaranala; verbatimElevation: 1100; decimalLatitude: -18.26; decimalLongitude: 45.41833; georeferenceRemarks: coordinates obtained from GPS; **Event:** samplingProtocol: MW 50 sample transect, 5m; eventDate: 01/16/2003; habitat: montane rainforest; fieldNumber: BLF07086; eventRemarks: sifted litter (leaf mold, rotten wood); **Record Level:** institutionCode: CASC
- **Type status:** Other material. **Occurrence:** catalogNumber: casent0027537; recordedBy: Fisher, Griswold et al.; sex: 1w; preparations: pin; associatedMedia: http://www.antweb.org/specimen/casent0027537; **Taxon:** scientificName: Stigmatomma tsyhady; genus: Stigmatomma; **Location:** country: Madagascar; stateProvince: Toliara; locality: Réserve Spéciale d'Ambohijanahary, Forêt d'Ankazotsihitafototra, 35.2 km 312° NW Ambaravaranala; verbatimElevation: 1050; decimalLatitude: -18.26667; decimalLongitude: 45.40667; georeferenceRemarks: coordinates obtained from GPS; **Event:** samplingProtocol: MW 50 sample transect, 5m; eventDate: 01/13/2003; habitat: montane rainforest; fieldNumber: BLF07020; eventRemarks: sifted litter (leaf mold, rotten wood); **Record Level:** institutionCode: CASC
- **Type status:** Other material. **Occurrence:** catalogNumber: casent0027538; recordedBy: Fisher, Griswold et al.; sex: 1w; preparations: pin; associatedMedia: http://www.antweb.org/specimen/casent0027538; **Taxon:** scientificName: Stigmatomma tsyhady; genus: Stigmatomma; **Location:** country: Madagascar; stateProvince: Toliara; locality: Réserve Spéciale d'Ambohijanahary, Forêt d'Ankazotsihitafototra, 35.2 km 312° NW Ambaravaranala; verbatimElevation: 1050; decimalLatitude: -18.26667; decimalLongitude: 45.40667; georeferenceRemarks: coordinates obtained from GPS; **Event:** samplingProtocol: MW 50 sample transect, 5m; eventDate: 01/13/2003; habitat: montane rainforest; fieldNumber: BLF07020; eventRemarks: sifted litter (leaf mold, rotten wood); **Record Level:** institutionCode: CASC
- **Type status:** Other material. **Occurrence:** catalogNumber: casent0027539; recordedBy: Fisher, Griswold et al.; sex: 1w; preparations: pin; associatedMedia: http://www.antweb.org/specimen/casent0027539; **Taxon:** scientificName: Stigmatomma tsyhady; genus: Stigmatomma; **Location:** country: Madagascar; stateProvince: Toliara; locality: Réserve Spéciale d'Ambohijanahary, Forêt d'Ankazotsihitafototra, 35.2 km 312° NW Ambaravaranala; verbatimElevation: 1050; decimalLatitude: -18.26667; decimalLongitude: 45.40667; georeferenceRemarks: coordinates obtained from GPS; **Event:** samplingProtocol: MW 50 sample transect, 5m; eventDate: 01/13/2003; habitat: montane rainforest; fieldNumber: BLF07020; eventRemarks: sifted litter (leaf mold, rotten wood); **Record Level:** institutionCode: CASC
- **Type status:** Other material. **Occurrence:** catalogNumber: casent0074427; recordedBy: Malagasy ant team; sex: 1w; preparations: pin; associatedMedia: http://www.antweb.org/specimen/casent0074427; **Taxon:** scientificName: Stigmatomma tsyhady; genus: Stigmatomma; **Location:** country: Madagascar; stateProvince: Toamasina; locality: Analamay; verbatimElevation: 1068; decimalLatitude: -18.80623; decimalLongitude: 48.33707; georeferenceRemarks: coordinates obtained from GPS; **Event:** samplingProtocol: MW, 25 sifted litter; eventDate: 03/21/2004; habitat: montane rainforest; fieldNumber: BLF10502; eventRemarks: sifted litter (leaf mold, rotten wood); **Record Level:** institutionCode: CASC
- **Type status:** Other material. **Occurrence:** catalogNumber: casent0074428; recordedBy: Malagasy ant team; sex: 1w; preparations: pin; associatedMedia: http://www.antweb.org/specimen/casent0074428; **Taxon:** scientificName: Stigmatomma tsyhady; genus: Stigmatomma; **Location:** country: Madagascar; stateProvince: Toamasina; locality: Analamay; verbatimElevation: 1068; decimalLatitude: -18.80623; decimalLongitude: 48.33707; georeferenceRemarks: coordinates obtained from GPS; **Event:** samplingProtocol: MW, 25 sifted litter; eventDate: 03/21/2004; habitat: montane rainforest; fieldNumber: BLF10502; eventRemarks: sifted litter (leaf mold, rotten wood); **Record Level:** institutionCode: CASC
- **Type status:** Other material. **Occurrence:** catalogNumber: casent0074430; recordedBy: Malagasy ant team; sex: 1w; preparations: pin; associatedMedia: http://www.antweb.org/specimen/casent0074430; **Taxon:** scientificName: Stigmatomma tsyhady; genus: Stigmatomma; **Location:** country: Madagascar; stateProvince: Toamasina; locality: Analamay; verbatimElevation: 1068; decimalLatitude: -18.80623; decimalLongitude: 48.33707; georeferenceRemarks: coordinates obtained from GPS; **Event:** samplingProtocol: MW, 25 sifted litter; eventDate: 03/21/2004; habitat: montane rainforest; fieldNumber: BLF10502; eventRemarks: sifted litter (leaf mold, rotten wood); **Record Level:** institutionCode: CASC
- **Type status:** Other material. **Occurrence:** catalogNumber: casent0074431; recordedBy: Malagasy ant team; sex: 1w; preparations: pin; associatedMedia: http://www.antweb.org/specimen/casent0074431; **Taxon:** scientificName: Stigmatomma tsyhady; genus: Stigmatomma; **Location:** country: Madagascar; stateProvince: Toamasina; locality: Analamay; verbatimElevation: 1068; decimalLatitude: -18.80623; decimalLongitude: 48.33707; georeferenceRemarks: coordinates obtained from GPS; **Event:** samplingProtocol: MW, 25 sifted litter; eventDate: 03/21/2004; habitat: montane rainforest; fieldNumber: BLF10502; eventRemarks: sifted litter (leaf mold, rotten wood); **Record Level:** institutionCode: CASC
- **Type status:** Other material. **Occurrence:** catalogNumber: casent0073991; recordedBy: Malagasy ant team; sex: 1w; preparations: pin; associatedMedia: http://www.antweb.org/specimen/casent0073991; **Taxon:** scientificName: Stigmatomma tsyhady; genus: Stigmatomma; **Location:** country: Madagascar; stateProvince: Toamasina; locality: Forêt Ambatovy, 14.3 km 57° Moramanga; verbatimElevation: 1075; decimalLatitude: -18.85083; decimalLongitude: 48.32; georeferenceRemarks: coordinates obtained from GPS; **Event:** samplingProtocol: MW, 25 sifted litter; eventDate: 03/21/2004; habitat: montane rainforest; fieldNumber: BLF10501; eventRemarks: sifted litter (leaf mold, rotten wood); **Record Level:** institutionCode: CASC
- **Type status:** Other material. **Occurrence:** catalogNumber: casent0074179; recordedBy: Malagasy ant team; sex: 1w; preparations: pin; associatedMedia: http://www.antweb.org/specimen/casent0074179; **Taxon:** scientificName: Stigmatomma tsyhady; genus: Stigmatomma; **Location:** country: Madagascar; stateProvince: Toamasina; locality: Forêt Ambatovy, 14.3 km 57° Moramanga; verbatimElevation: 1075; decimalLatitude: -18.85083; decimalLongitude: 48.32; georeferenceRemarks: coordinates obtained from GPS; **Event:** samplingProtocol: MW, 25 sifted litter; eventDate: 03/21/2004; habitat: montane rainforest; fieldNumber: BLF10501; eventRemarks: sifted litter (leaf mold, rotten wood); **Record Level:** institutionCode: CASC
- **Type status:** Other material. **Occurrence:** catalogNumber: casent0074290; recordedBy: Malagasy ant team; sex: 1w; preparations: pin; associatedMedia: http://www.antweb.org/specimen/casent0074290; **Taxon:** scientificName: Stigmatomma tsyhady; genus: Stigmatomma; **Location:** country: Madagascar; stateProvince: Toamasina; locality: Forêt Ambatovy, 14.3 km 57° Moramanga; verbatimElevation: 1075; decimalLatitude: -18.85083; decimalLongitude: 48.32; georeferenceRemarks: coordinates obtained from GPS; **Event:** samplingProtocol: MW, 25 sifted litter; eventDate: 03/21/2004; habitat: montane rainforest; fieldNumber: BLF10501; eventRemarks: sifted litter (leaf mold, rotten wood); **Record Level:** institutionCode: CASC
- **Type status:** Other material. **Occurrence:** catalogNumber: casent0074291; recordedBy: Malagasy ant team; sex: 1w; preparations: pin; associatedMedia: http://www.antweb.org/specimen/casent0074291; **Taxon:** scientificName: Stigmatomma tsyhady; genus: Stigmatomma; **Location:** country: Madagascar; stateProvince: Toamasina; locality: Forêt Ambatovy, 14.3 km 57° Moramanga; verbatimElevation: 1075; decimalLatitude: -18.85083; decimalLongitude: 48.32; georeferenceRemarks: coordinates obtained from GPS; **Event:** samplingProtocol: MW, 25 sifted litter; eventDate: 03/21/2004; habitat: montane rainforest; fieldNumber: BLF10501; eventRemarks: sifted litter (leaf mold, rotten wood); **Record Level:** institutionCode: CASC
- **Type status:** Other material. **Occurrence:** catalogNumber: casent0074309; recordedBy: Malagasy ant team; sex: 1w; preparations: pin; associatedMedia: http://www.antweb.org/specimen/casent0074309; **Taxon:** scientificName: Stigmatomma tsyhady; genus: Stigmatomma; **Location:** country: Madagascar; stateProvince: Toamasina; locality: Forêt Ambatovy, 14.3 km 57° Moramanga; verbatimElevation: 1075; decimalLatitude: -18.85083; decimalLongitude: 48.32; georeferenceRemarks: coordinates obtained from GPS; **Event:** samplingProtocol: MW, 25 sifted litter; eventDate: 03/21/2004; habitat: montane rainforest; fieldNumber: BLF10501; eventRemarks: sifted litter (leaf mold, rotten wood); **Record Level:** institutionCode: CASC
- **Type status:** Other material. **Occurrence:** catalogNumber: casent0208068; recordedBy: R. Harin'Hala; sex: 1dQ; preparations: pin; associatedMedia: http://www.antweb.org/specimen/casent0208068; **Taxon:** scientificName: Stigmatomma tsyhady; genus: Stigmatomma; **Location:** country: Madagascar; stateProvince: Fianarantsoa; locality: 1 km E of Isalo National Park Interpretative Center; verbatimElevation: 885; decimalLatitude: -22.62667; decimalLongitude: 45.35817; georeferenceRemarks: coordinates obtained from GPS; **Event:** samplingProtocol: Malaise trap; eventDate: 06/12/2002; habitat: dry wash; fieldNumber: MA-02-11B-32; **Record Level:** institutionCode: CASC

#### Description

Worker (Fig. 101; holotype values within parentheses): **HL**: 1.04-1.33 (1.31); **HW**: 0.95-1.24 (1.13); **HW2**: 0.84-1.08 (1.03); **SL**: 0.59-0.73 (0.73); **ML**: 0.67-0.94 (0.87); **WL**: 1.36-1.72 (1.70); **PPW**: 0.60-0.79 (0.70); **PtL**: 0.63-0.83 (0.78); **PtW**: 0.64-0.85 (0.77); **CI**: 84-93 (86); **SI**: 55-57 (56); **MI**: 64-70 (66); **PtI**: 98-104 (102). 

*Head*:

Mandibular baso-masticatory margin skirted dorsally by row of filiform setae; ventrally, by spatular setae (Fig. 102a). Mandibular dentition arrangement, from base to apex: single larger tooth; much smaller single tooth (absent or much reduced in length in some specimens); five pairs of teeth (each tooth pair with same dimensions, fused basally; pairs of teeth similar in length along basoapical axis of mandible); single preapical tooth; apical tooth (Fig. 102a​). Anterior clypeal margin with eight to nine tubercle-like cuticular processes, arranged in a single row, armed anteriorly with an asymmetrical, mucronate, dentiform seta (Fig. 102a​). Lateral-most clypeal cuticular process with row of smaller conical setae anterolaterally, continuing laterally along clypeal anterior margin, arising from flat cuticle (Fig. 102a​). Median clypeal cuticular processes around 2x the length of associated dentiform setae (Fig. 102a​). Pair of long, filiform setae on anterior clypeal margin bordering the central-most cuticular processes (Fig. 102a​). Median area of clypeus extending posteriorly between antennal sockets; frontoclypeal sulcus round (Fig. 102a, b). Supraclypeal area an oval concavity (Fig. 102a, b​). Twelve antennomeres. Genal teeth present (Fig. 101a). Widest diameter of compound eyes: two to three ommatidia (Fig. 102c). Palpal formula: 4:3 (four maxillary, three labial; Fig. 102d​). 

*Mesosoma*:

In dorsal view, lateral margins of mesonotum continuous with posterior remainder of mesosoma, or somewhat expanded laterally (Fig. 103a). Metanotal suture absent or weakly impressed (Fig. 103a​). Sulcus dividing mesepisternum into anepisternum and katepisternum (Fig. 103b). Metathoracic spiracle slit­-like (Fig. 103b​). Propodeal spiracle round, with swollen margin (Fig. 103b​). Face of propodeal declivity slightly concave (Fig. 103a​).

*Legs*:

Baso­ventral half of calcar of strigil lamellar (Fig. 104b). Anterior face of calcar of strigil with strap­-like microtrichia (Fig. 104a); posterior face with lanceolate microtrichia (Fig. 104b). Multiple paddle-like setae on antero­ventral face of protibial apex, next to calcar of strigil (Fig. 104a). Multiple paddle­-like setae on anterior face of probasitarsus (Fig. 104a); row of stout setae on posterior face, parallel to comb of strigil (Fig. 104b). Two mesotibial spurs (Fig. 104c); simple anterior spur covered with lanceolate microtrichia, posterior spur somewhat falcate (with round baso­ventral projection) and covered with lanceolate microtrichia. Ventral margin of posterior mesotibial spur with digitiform cuticular projections, restricted to the basal-most region or along entire ventral margin. Absence of a longitudinal sulcus on the mesobasitarsus (Fig. 104d). Stout filiform setae along inner face of mesobasitarsus. Two metatibial spurs (Fig. 105a): simple anterior spur, with lanceolate microtrichia; posterior spur pectinate. Anterior face of posterior spur almost glabrous, with few lanceolate microtrichia (Fig. 105a); posterior face glabrous (Fig. 105b). Absence of a longitudinal sulcus on metabasitarsus (Fig. 105c). Row of few stout, paddle­-like setae on the baso-inner face of metabasitarsus. Stout filiform setae on the remainder of inner face of metabasitarsus (Fig. 105c​). Arolium on pro-, meso-, and metapretarsus.

*Metasoma*:

Petiole sessile (Fig. 106a). Ventroanterior margin of petiolar tergite anterior dorso-latero-ventral carina (Ward 1990) slightly shorter than anterior margin of subpetiolar process, in lateral view (Fig. 106a). Ventral margin of subpetiolar process running posteriorly in a somewhat continuous line (Fig. 106a). Presence of a fenestra on the lateral face of subpetiolar process (Fig. 106a). Petiolar proprioceptor zone a large, round concavity with numerous sensilla (Fig. 106b). Prora present (Fig. 106a). Scrobiculate sulcus between pretergite and postergite of abdominal segment III and presclerites and postsclerites of abdominal segment IV. Absence of stout setae on hypopygium (Fig. 106c).

**Figure 107.**
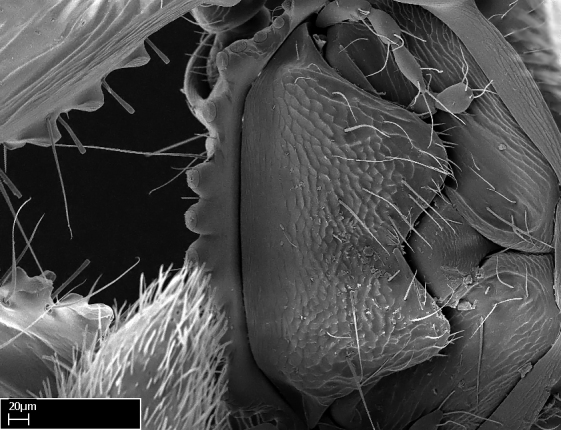
*Stigmatomma tsyhady* ​**sp. n.** worker (CASENT0074309): ventral view of the mouth parts. Image by F. A. Esteves; available at AntWeb.org.

*Sculpture*:

Mandibular dorsal face mostly costate, except for smooth apical portion (Fig. 102a). Clypeal median area costate (Fig. 102a). Supraclypeal area mostly smooth (Fig. 102a). First third of the head, in dorsal view, mostly costulate­-foveolate, grading into foveolate posteriorly (Fig. 102b). Area posterior to tentorial pit carinate concentrically (Fig. 102b​). Labrum imbricate (Fig. 107). Pronotum and dorsal face of mesosoma foveolate (Fig. 103). Katepisternum mostly costulate (Fig. 103b). Metapleuron costate dorsally and posteriorly (Fig. 103b). Remainder of the lateral face of mesosoma smooth (Fig. 103b). Face of propodeal declivity foveolate (Fig. 103a​). Petiolar tergite smooth anteriorly, grading into punctate/foveolate-rugulose laterally, and into punctate/foveolate dorsally (Fig. 106a); laterotergite somewhat imbricate; poststernite alveolate (Fig. 106b). Abdominal segments III and IV weakly punctate/foveolate; tergites of abdominal segments VI and VII weakly imbricate (Fig. 106c).

*Pilosity and color*:

Erect to subdecumbent pilosity on head, dorsal face of mesosoma, petiolar tergite, and abdominal segments III, IV, and V. Erect to suberect pilosity on anterior half of petiolar poststernite. Longer pilosity on abdominal segments VI and VII. Body color brown to black; gaster yellow­-brown to brown (sometimes sclerites are darkish, grading into yellow­-brown to orange towards anterior and posterior margins); appendages yellow­-brown to orange.

##### Comments on character variation

No geographic pattern is seen in the variation seen on *Stigmatomma tsyhady*, and characters such as body size, the position and size of the smaller basal single tooth on the mandibles, number of dentiform setae on the anterior margin of the clypeus, degree of mesonotum expansion, metanotal suture presence and its degree of development, amount of cuticular projections on the ventral margin of the posterior mesotibial spur, sculpture, and color fluctuate even among specimens collected at the same locality.

##### Other castes

Gyne (Fig. 108); alate when virgin: similar to the worker caste but for the greater body length, larger compound eyes, presence of ocelli (Fig. 108a), and differences on the mesosoma due to the presence of wings. Parapsidal lines on the mesoscutum; scuto-scutellar suture narrow, without apparent sculpture on its midsection, but scrobiculate on its apexes (Fig. 109a). Mesepisternum somewhat divided into anepisternum and katepisternum; anepisternum dispersed foveate; katepisternum costate; mesepimeral lobe not distinct; metapleuron divided into upper and lower sections; upper metapleuron separated from propodeum by wide, costate-dispersed foveate sulcus; lower metapleuron separated from the propodeum by a carina, followed dorsally by a strigate sulcus (Fig. 109b). Forewing (Fig. 109c): pterostigma well developed; Rs.f2-3 present; Rs.f5 present and reaching R.f3; 1r-rs absent; 2r-rs, M.f4, 2rs-m, Cu.f2, 1m-cu, and A.f2 present; cu-a intercepting M+Cu anteriorly to the separation point between Cu.f1and M.f1. Hindwing (Fig. 109d): C indistinct; R present, but indistinct when reaching costal margin; Rs.f2 and 1rs-m present; M.f2 present, but indistinct; Cu, cu-A, and A.f2 present.

Male (Fig. 110); alate: Mandibles falcate, with sharp, single apical tooth (Fig. 110a​). Anterior clypeal margin armed with dentiform setae (Fig. 110a​). Compound eyes with sparse setae present among ommatidia (Fig. 110a). Palpal formula 4:3 (Fig. 111a). Notauli distinct and scrobiculate; parapsidal lines present; scuto-scutellar suture scrobiculate (Fig. 112a​). Mesepisternum partially divided into anepisternum and katepisternum; posterior oblique sulcus short, not well developed; epimeral lobe well developed; metapleuron divided into upper and lower sections by a pit; scrobiculate sulcus separating upper metapleuron from propodeum; narrower scrobiculate sulcus separating lower metapleuron from propodeum (Fig. 112b). Forewing (Fig. 113a​): pterostigma well developed; Rs.f2-3 present; Rs.f5 present and reaching R.f3; 1r-rs absent; 2r-rs, M.f4, 2rs-m, Cu.f2, 1m-cu, and A.f2 present; cu-a intercepting M+Cu at the separation point between M.f1 and Cu.f1. Hindwing (Fig. 113b): C present; R seemingly absent; Rs.f2, M.f2, and Cu present as not well-distinct stubs; 1rs-m and cu-a present; A.f2 distinct, but short. Pygostyles present (Fig. 112c). Posterior margin of abdominal segment IX convex (Fig. 111d). Paramere not visibly divided into telomere and basimere. Digitus tongue-plier-shaped: presence of a comparatively enlarged, but thin basal projection on the digitus; cuspis shorter than digitus (Fig. 111c). Entire ventral margin of the penisvalva comparatively finely serrate; dorsal portion of the penisvalva sclerotized (Fig. 111b).

##### Specimens used in prior studies

*Stigmatomma tsyhady* was referenced as *Amblyopone* sp.1 (specimen CASENT0500011) in Saux et al. (2004).

#### Diagnosis

Worker

With characters of the *tsyhady* species­-group and the *tsyhady* species­-complex as described above, and the following characters (asterisks flag unique characters within the genus in the Malagasy bioregion):

1. Integument brown to black (Fig. 101); medium- to large­-sized ant (HL: 1.04-1.33 , WL: 1.36-1.72).
2. Pairs of teeth along baso­-masticatory margin of mandible have the same length along basoapical axis (Fig. 102a).
3. Spatular setae ventrally skirting baso­-masticatory margin of mandible (Fig. 102a).
4. Dorsal face of the head mostly costulate­-foveolate, grading into foveolate posteriorly (Fig. 102b).
5. Palpal formula 4:3 (Fig. 102d​).
6. Pronotum and the dorsal face of remainder mesosoma foveolate; lateral face of remainder mesosoma mostly smooth; propodeal declivitous face foveolate (Fig. 103).
7. Mesepisternum divided into anepisternum and katepisternum (Fig. 103b).
8. Baso­ventral half of calcar of strigil lamellar (Fig. 104b).
9. Anterior face of calcar of strigil with strap­-like microtrichia (Fig. 104a).
10. Two mesotibial spurs (Fig. 104c).
* Absence of a longitudinal sulcus on mesobasitarsus (Fig. 104d).
12. Two metatibial spurs (Fig. 105a).
13. Anterior face of posterior spur almost glabrous, with few lanceolate microtrichia (Fig. 105a); posterior face glabrous (Fig. 105b).
14. Row of of few stout, paddle-like setae present on the baso-inner face of metabasitarsus.
15. Absence of a longitudinal sulcus on metabasitarsus (Fig. 105c).
16. Subpetiolar process runs continuously posteriorly, not forming a fin (Fig. 106a).

*Stigmatomma tsyhady*, *S. irayhady*, *S. roahady*, and *S. liebe* share the presence of genal teeth, palpal formula, presence of fenestra on the subpetiolar process, shape of microtrichia on posterior face of posterior metatibial spur, and absence of stout setae on the apex of hypopygium. Color, size, and presence of two mesotibial spurs make *S. irayhady*, *S. roahady* and *S. tsyhady* even more similar. However, *S. tsyhady* is distinguished by the absence of a sulcus on the antero­dorsal face of its mesobasitarsus, and on the anterior face of its metabasitarsus.

*Stigmatomma tsyhady* occurs in sympatry with *S. irayhady* at the Binara Forest, with *S. roahady* in twelve localities (Ambatovy Forest, Analamay Forest, Vevembe Forest, Mantadia National Park, Marojejy National Park, Ranomafana National Park, Zahamena National Park, Andohahela National Park, Andringitra Reserve, Ivohibe special reserve, Anosyenne Mountains, and at the Binara Forest), and with *S. liebe* in four localities (Andohahela National Park, Andringitra Reserve, Ivohibe Special Reserve, and at the Anosyenne Mountains).

#### Etymology

Combines the Malagasy preposition *tsy*, meaning absence, and the Malagasy noun *hady*, meaning sulcus, ditch, or trench. The name *tsyhady* refers to the absence of longitudinal sulci on the mesobasitarsus and metabasitarsus of this species; such absences together are unique among *Stigmatomma* species in the Malagasy bioregion.

#### Distribution

**Figure 114.**
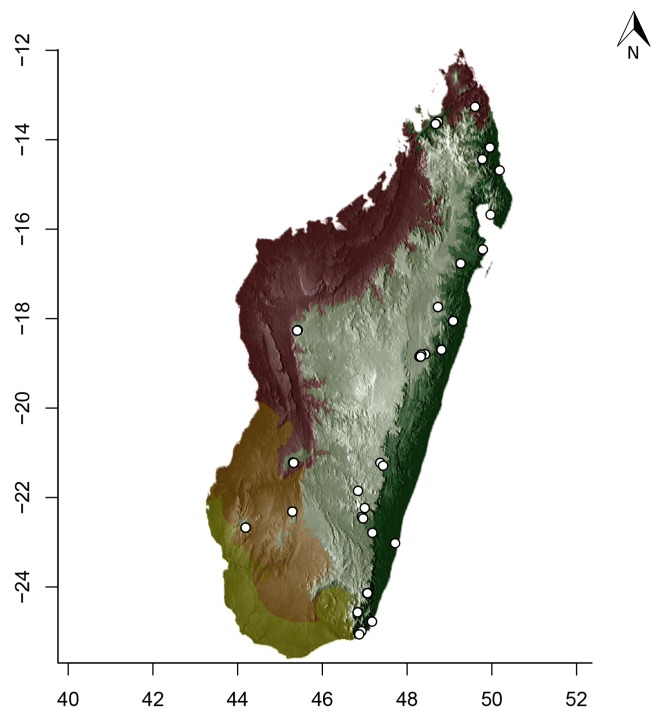
Distribution map of *Stigmatomma tsyhady* ​**sp. n.** in the Malagasy bioregion. Collection localities are mapped over the outlines of five simplified ecoregion zones of Madagascar: humid forests (dark green), subhumid forests (light green), dry deciduous forests (brown), succulent woodlands (orange), and spiny thickets (yellow).

*Stigmatomma tsyhady* has been collected in gallery forests, littoral forests, montane forests, and rainforests within the limits of the humid forests and subhumid forests ecoregions (sensu Schatz 2000) in the eastern biome of Madagascar (*sensu* Burgess et al. 2004; Fig. 114). Specimens were distributed at elevations ranging from 10 to 1125 m, of which 72 collection events occurred at an elevation lower than 800 m, out of a total of 131 collection events.

Specimens were recorded from sifted leaf mold and rotten wood (119 collection events), Malaise trap (one event), ground foraging (two events), nesting or foraging in the root mat on the ground or on rock (three events and two events, respectively), in a rotten log (one event), and in the soil (two events).

## Identification Keys

### Identification key for *Stigmatomma* workers and gynes in the Malagasy bioregion

**Table:** 

1	Ten antennomeres (Fig. 115a, A1). In fullface view, genal teeth absent (Fig. 115a, B1). Dorsal face of the head densely taeniate catenate (Fig. 116a, b). Clypeus narrowly inserted between frontal lobes (Fig. 115a, C1). Mesepisternum divided into anepisternum and katepisternum (Fig. 115c) [workers only]. Subpetiolar process without a fenestra (Fig. 115e). Color yellow (Fig. 117a). Small sized ant (HL= 0.38-0.40; WL= 0.41-0.43). (Seychelles).	*** besucheti ***
–	Twelve antennomeres (Fig. 115b, A2). In fullface view, genal teeth absent or present (Fig. 115b, B2). Dorsal sculpture of the head variable, but not taeniate-catenate (Figs 116c, d, e, f, 118). Clypeus narrowly or broadly inserted between frontal lobes (Fig. 115b, C2). Mesepisternum divided or not into anepisternum and katepisternum (Fig. 115d) [workers only]. Subpetiolar process with or without a fenestra (Fig. 115f). Color and size variable (Figs 117b, c, d, e, f, 119).	2
2	Subpetiolar process with a fenestra (Fig. 115f). In lateral view, stout setae absent from the apex of hypopygium (Fig. 120b) [requires high magnification; differences in light/shade may enhance setae visualization]. Genal teeth present in fullface view (Fig. 115b, B2). Mesepisternum divided into anepisternum and katepisternum (Fig. 120e) [workers only].	3
–	Subpetiolar process without fenestra (Fig. 120a). In lateral view, stout spiniform setae present on the apex of hypopygium (Fig. 120c) [requires high magnification; differences in light/shade may enhance setae visualization]. Genal teeth present or absent in fullface view (for the absence of genal teeth see Fig. 120d). Mesepisternum divided or not into anepisternum and katepisternum (for an undivided mesepisternum, see Fig. 115d) [workers only].	6
3	Longitudinal sulcus present on the anterior face of metabasitarsus (Fig. 121a) [differences in light/shade may enhance this character]. Longitudinal sulcus present on the anterodorsal face of mesobasitarsus (Fig. 121c) [differences in light/shade may enhance this character]. Dorsal face of the head costulate-punctate/foveolate, grading into punctate/foveolate posteriorly (Fig. 118a). Body color orange-brown to black; gaster orange to black with slightly lighter apex; appendages yellow-brown to orange (Fig. 117f). Large-sized ant (HL= 1.32-1.60; WL= 1.68-2.03). (Madagascar)	***roahady* sp. n.**
–	Longitudinal sulcus absent from metabasitarsus (Fig. 121b) [differences in light/shade may enhance this character]. Longitudinal sulcus on mesobasitarsus present or absent (for the absence of sulcus from mesobasitarsus see Fig. 121d) [differences in light/shade may enhance this character]. Dorsal sculpture of the head variable (Figs 116d, f, 118). Color variable (Figs 117c, e, 119b). Body size variable.	4
4	Longitudinal sulcus absent from mesobasitarsus (Fig. 121d) [differences in light/shade may enhance the absence of this character]. Dorsal face of the head costulate-foveolate, grading into foveolate posteriorly (Fig. 118c). Body color brown to black; gaster yellow­-brown to brown (sometimes sclerites are darkish, grading into yellow­-brown to orange towards anterior and posterior margins); appendages yellow­-brown to orange (Fig. 119b). Medium-sized ant (HL= 1.04-1.33; WL= 1.36-1.72). (Madagascar)	***tsyhady* sp. n.**
–	Longitudinal sulcus present on the anterodorsal face of mesobasitarsus (Fig. 122) [differences in light/shade may enhance this character]. Dorsal sculpture of the head variable (Fig. 116d, f). Color variable (Fig. 117c, e). Body size variable.	5
5	Body color dark brown to blackish; apex of the gaster orange; yellow-brown appendages (Fig. 117c). Anterior and posterior metatibial spurs always visible (anterior spur longer than half the length of the posterior metatibial spur; Fig. 123a). Anterior and posterior mesotibial spurs always visible (Fig. 123c). Dorsal face of the head mostly areolate-rugose, grading into foveolate/foveate posteriorly (Fig. 116d​). Large-sized ant (HL= 1.07-1.14; WL= 1.37-1.55). (Madagascar)	***irayhady* sp. n.**
–	Body color dark yellow to orange; yellow appendages (Fig. 117e). Anterior metatibial spur generally not visible, but if visible, extremely reduced in length (less than one-third the length of the posterior metatibial spur; Fig. 123b). Anterior mesotibial spur generally not visible (Fig. 123d). Dorsal face of the head mostly costate-slightly catenate-foveolate, grading into foveolate posteriorly and laterally (Fig. 116f). Medium-sized ant (HL= 0.90-0.96; WL= 1.16-1.34). (Madagascar)	***liebe* sp. n.**
6	Genal teeth present in fullface view (Fig. 124a). Mesepisternum not divided into anepisternum and katepisternum (Fig. 115d) [workers only]. Brush of filiform setae on the lateral-most clypeal area in fullface view (Fig. 124c) [if setae are removed, the region will present dense punctuations under higher magnification]. Lamella on the ventral margin of the calcar of strigil not visible (Fig. 124e). Body color orange-brown; light orange appendages (Fig. 117d​). Dorsal face of the head areolate (Fig. 116e). Medium-sized ant (HL= 0.74-0.79; WL= 0.87-0.93). (Seychelles)	***janovitsika* sp. n.**
–	Genal teeth absent in fullface view (Fig. 120d). Mesepisternum divided into anepisternum and katepisternum (Fig. 124b) [workers only]. No brush of setae or densely arranged punctuations on the lateral-most clypeal area in fullface view (Fig. 124d). Lamella on the ventral margin of the calcar of strigil visible (Fig. 124f). Sculpture of dorsal face of the head foveate to densely foveate/foveate-reticulate (Figs 116c, 118b​). Color variable (Figs 117b, 119a). Body size variable.	7
7	In dorsal view, declivitous face of the propodeum strigate (​Fig. 125a). Dorsal face of the head mostly densely foveate/foveate-reticulate (Fig. 116c). Mesosoma lateral face costate-foveate (Fig. 124b). Body color red-brown; apex of gaster and appendages orange-yellow (Fig. 117b). Medium-sized ant (HL= 0.76; WL= 0.92). (Madagascar)	***bolabola* sp. n.**
–	In dorsal view, face of propodeal declivity smooth or weakly alveolate (Fig. 125b). Dorsal face of the head foveate (Fig. 118b). Katepisternum and metapleuron alveolate; lateral face of the propodeum dispersed costulate-weakly alveolate, or mostly alveolate (Fig. 125c). Body color orange-brown; appendages yellow to light orange (Fig. 119a). Medium-sized ant (HL= 0.74-0.76; WL= 0.89-0.94). (Madagascar)	***sakalava* sp. n.**

Aid for character visualization and its applicability is placed within brackets.

## Discussion

### Distribution

**Figure 126.**
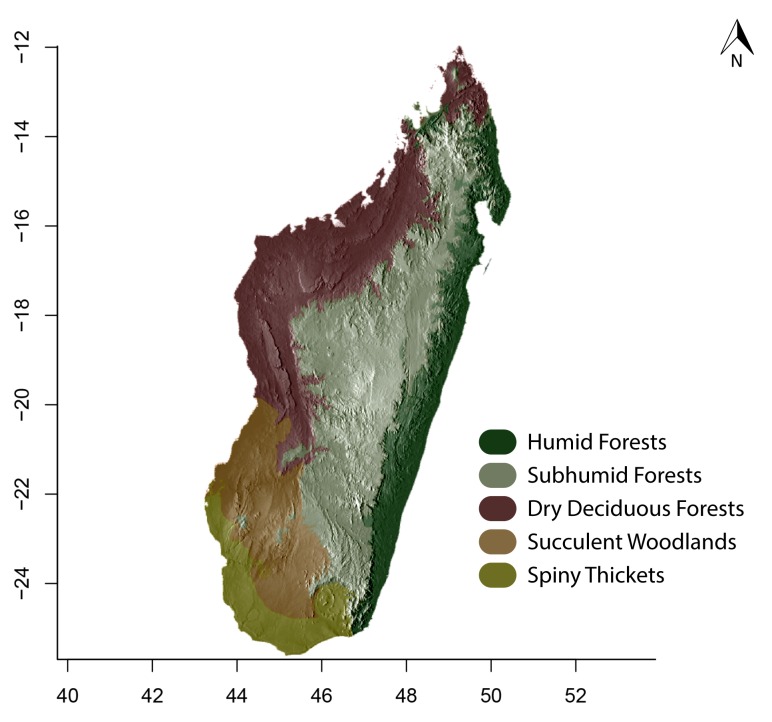
The outlines of five simplified ecoregions of Madagascar are mapped over the shaded relief of the island. Humid forests are highlighted in dark green, subhumid forests in light green, dry deciduous forests in brown, succulent woodlands in orange, and spiny thickets in yellow.

Madagascar is covered by three biomes, each of which shares a similar plant composition. Based on bioclimatic variables, the biomes may be divided into smaller subunits called ecoregions (Fig. 126). In general, the distribution of *Stigmatomma* species reflects the division of the island into those units. Below, we provide a brief description of each biome and nested ecoregions, and summarize the distribution pattern of each *Stigmatomma* species occurring in Madagascar.

**Tropical and Subtropical Moist Broadleaf Forests Biome (herein called Eastern biome)** – found in the center and east of the island, extending over the coastal plain, eastern escarpment, and over the central highlands of Madagascar (Burgess et al. 2004). All *Stigmatomma* species distributed in the island occur in the eastern biome, excluding *S. sakalava*. Based mostly on the annual amount of rainfall and the length of the dry season, this biome is currently subcategorized in two ecoregions: humid forests and subhumid forests.

Humid Forests – ranges from the coastal plain in the east to the limits of the subhumid ecoregion in the west (Burgess et al. 2004). Rainfall exceeds 2000 mm per year, and the dry season lasts less than two months (Burgess et al. 2004). Fauna and flora composition shifts with elevation and with latitude; for some vertebrates, major rivers play a significant role as dispersion barriers along the eastern latitudinal range of Madagascar (Burgess et al. 2004).

Some studies place the border between humid and subhumid ecoregions around an elevation of 600–900 m (Du Puy and Moat 1996, Faramalala 1995, Faramalala 1988, Koechlin et al. 1974, Humbert 1955, Humbert 1965, Perrier de la Bâthie 1921), while Burgess et al. (2004) lay it at the crest of the eastern escarpment (around 1200–1600 m). We prefer the boundaries established by Burgess et al. (2004)​, for it is based on the differences of dry season length and annual rainfall, which is consistent with the methodology used to divide the island into ecoregions. Also, as seen below, the distribution patterns of *Stigmatomma* species that occur in the area corroborate their hypothesis.

Three species of *Stigmatomma* were only recorded within the boundaries of this ecoregion. *S. liebe* was found between 1125 and 1315 m at the south portion of the eastern escarpment (Fig. 72). *S. bolabola* was found only at the rainforests and montane forests of the Makira Forest Protected Area in the northeast of the island (Fig. 34). *S. roahady* ranges along the eastern escarpment from 400 to 1400 m elevation, and in the northwest region known as Sambirano (Fig. 87). As described below, the Sambirano is considered by some studies as part of the humid forests ecoregion for its phytophysiognomy and bioclimate, and the distribution of *S. roahady* agrees with that.

Subhumid Forests – occupies central Madagascar, and merges into the humid forests to the east, and the western biome to the west; it is replaced by the spiny thickets and succulent woodlands in the southwest (Burgess et al. 2004). It also includes disjunct forest blocks to the southwest (e.g. Makay, Isalo, Analavelona; Burgess et al. 2004). This ecoregion includes montane forest, woodland (tapia forest), forest–grassland mosaic, and sclerophyllous forest. Annual rainfall is around 1500 mm, and the dry season lasts three to seven months (Burgess et al. 2004).

There is some dispute about the boundaries of this ecoregion with regards to the northwestern region known as Sambirano. The Sambirano possesses high local endemism, and has been considered a distinct ecoregion (Faramalala 1995, Faramalala 1988, Koechlin et al. 1974), as part of the subhumid forests (Burgess et al. 2004, Cornet and Guillaumet 1976), and as a disjunct part of the humid forests (Du Puy and Moat 1998, Koechlin et al. 1974, Humbert 1965). The latter is based on phytophysiognomy and bioclimatic data (Lowry II et al. 1997).

*Stigmatomma irayhady* was the only species strictly found in this ecoregion, recorded at elevations ranging from 1100 to 1620 m at the central/northern portion of the central plateau of Madagascar (Fig. 43). 

*Stigmatomma tsyhady* is widespread within the limits of the eastern biome, occupying habitats within the humid and subhumid forests ecoregions (Fig. 114). It has been collected in gallery forests, littoral forests, rainforests, and montane forests, at elevations ranging from 10 to 1125 m. 

**Tropical and Subtropical Dry and Broadleaf Forests Biome (herein mentioned as Western biome)** – covers a great portion of western Madagascar, transitioning into the succulent woodland to the south, and into the subhumid forest to the west (Burgess et al. 2004). The Sambirano region splits this ecoregion into two different geographic blocks. The dry season lasts approximately seven months, and annual rainfall is around 1500 mm in the north and 1000 mm in the south (Burgess et al. 2004). The vegetation is represented primarily by dry, deciduous forests, or by a deciduous forest/grassland mosaic (Burgess et al. 2004).

The southern boundary of the western biome has been under discussion, for its fauna and flora overlaps with those of the succulent woodland. Hence, some authors have lumped the succulent woodlands in the western biome (Du Puy and Moat 1998, Lowry II et al. 1997, Guillaumet 1984, Humbert 1965).

**Deserts and Xeric Shrublands (herein called Southern biome)** – covers the south and southwest of Madagascar, in areas with strong seasonality (Burgess et al. 2004). It may be further divided into two bioregions:

Succulent Woodlands – located at the southwestern and central western Madagascar, bordering the western biome to the north, the subhumid forests to the west, and the spiny thickets to the south. The dry season extends from May to October, and annual rainfall reaches 750 mm. Vegetation is similar to that of the western biome, but includes more xerophilous species. Fauna overlaps with that of the western biome to the north, and with that of the spiny thickets to the south.

Spiny Thickets – covers southern/southwestern Madagascar. It merges into the succulent woodlands to the north, and borders the subhumid forests to the east. Dry season may last for nine to eleven months, and annual rainfall is around 500 mm. Vegetation is primarily deciduous thicket, but also includes thicket/grassland mosaic, coastal scrub, and gallery forests around major rivers. 

*Stigmatomma sakalava* spreads across the western and southern biomes, and is the only species of the genus recorded for those areas (Fig. 100). However, in the southern biome, this species was only collected in gallery forests. Those forests are considered floristically/physiognomically similar to the vegetation of the western biome (WWF 2015, Cornet and Guillaumet 1976, Koechlin et al. 1974, Humbert 1965).

### Morphometry

**Figure 127.**
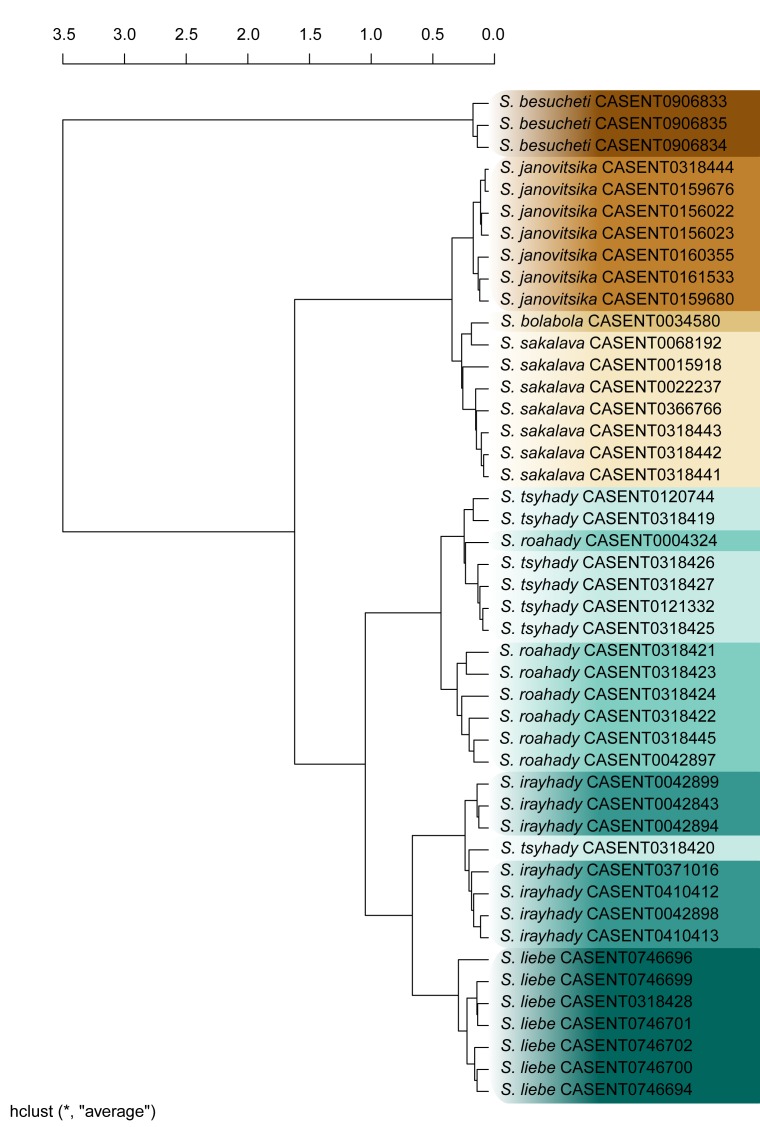
UPGMA hierarchical clustering of *Stigmatomma* specimens. It classified specimens into groups according to the dissimilarity of distances between log-normalized measurement values. The cophenetic correlation coefficient, which measures how well the cluster represented the distances between specimens, was 0.889.

We measured 46 specimens, comprising all *Stigmatomma* species present in the Malagasy region. Specimen clustering based on measurement data matched our species hypothesis fairly well (Fig. 127; cophenetic correlation coefficient: 0.889), with three exceptions: one specimen of *S. roahady* clustered with *S. tsyhady* (specimen CASENT0004324), one specimen of *S. tsyhady* clustered with *S. irayhady* (specimen CASENT0318420), and *S. bolabola* is nested within *S. sakalava*. The Principal Component Analysis (PCA) offers some hints on why the clustering did not perfectly fit our species hypothesis.

**Figure 128.**
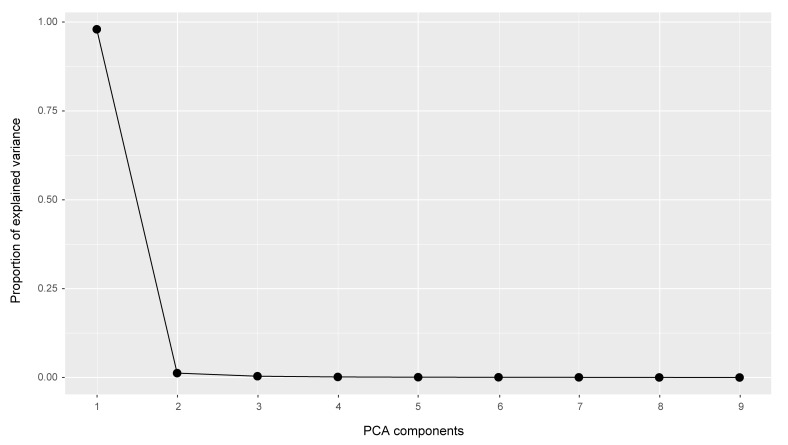
Scree plot of the proportion of total variance explained by each PCA component. See also Table 2.

PCA first two components accounts for more than 99% of the morphometric variance among specimens, as original variables were highly correlated (Table 1, Table 2, Fig. 128). The first component (PCA1) alone expresses 97.9% of the variance (Fig. 128, Table 2), and seems to represent size as it is equally related to all measurements (Table 3). In fact, PCA1 represents general size for almost every morphometric dataset that is not normalized for size (Zelditch et al. 2012). 

**Figure 129.**
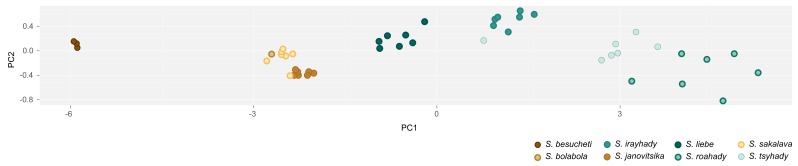
Principal Componente Analysis (PCA) resulting graphic. Specimens positions are projected onto the space defined by the two PCA components that represent most of the measurements variance (PCA1 and PCA2). Specimens are colored according to the species they were assigned *a priori* to this analysis: *Stigmatomma besucheti* in dark brown, *S. bolabola* in light brown outlined in dark brown, *S. irayhady* in teal blue, *S. janovitsika* in brown, *S. liebe* in dark teal blue, *S. roahady* in teal blue outlined in dark teal blue, *S. sakalava* in yellow outlined in dark yellow, and *S. tsyhady* in light teal blue/light turquoise (see legend for disambiguation).

Fig. 129 illustrates how size variation configures specimens in the ordination space. Samples in the right of the figure are larger than samples in the left. *Stigmatomma roahady* is the largest species, followed and somewhat overlapping in size with *S. tsyhady*, of which all specimens are larger than *S. irayhady*, but one. *S. liebe* is the smallest species of the *tsyhady* species-complex. Within *sakalava* species-complex, *S. janovitsika* is the largest species, and its smallest specimens overlap in size with the largest specimens of *S. sakalava*. The size range of the latter encompasses *S. bolabola*. *S. besucheti* is the smallest species we evaluated. PCA2 represents the difference of proportions between head and posterior remainder of the body (Table 3). Specimens located at the upper part of Fig. 129 have the posterior part of the body proportionally larger than the head, and the opposite happens with specimens at the lower part of the figure. 

PCA and cluster analysis results are consistent, and so, it seems that: (1) clustering basically reflected differences in size among specimens evaluated, and (2) intraspecific size variation explains the mismatches between clustering and our species hypothesis. However, PCA components do not clarify why size variation is discontinuous in the specimens of *Stigmatomma tsyhady* we evaluated. This may be just a reflection of sampling design bias.

Sampling design is crucial in morphometric analysis, for the analysis will better assess the variability among species if most of the variation within and among populations of species is represented in the samples (Marhold 2011). That was not the case in this study. We delimited species based mainly on qualitative characters, and only used morphometrics to quantify size and to provide means of comparison with other *Stigmatomma* species. In addition to the holotype and some paratypes, we selected specimens that seemed to represent maximum, minimum, and average values of the morphometric spectrum of a given species. Therefore, and depending on intraspecific variation, the number of selected specimens may not had been enough to capture the entire range of variation, as happened with *S. tsyhady*.

## Supplementary Material

Supplementary material 1 Linear morphometry of Stigmatomma species in the Malagasy bioregion – raw data set of measurements and indices Data type: morphological
File: oo_71176.xls Esteves, F.A.; Fisher, B.L.

Supplementary material 2 R script for clustering specimens based on measurement data Data type: R code
File: oo_87292.pdf Esteves, F.A.

Supplementary material 3 R script for Principal Component Analysis (PCA): specimens on a morphometric ordination space Data type: R code
File: oo_87291.pdf Esteves, F.A.

Supplementary material 4 R script for mapping the distribution of Stigmatomma species in Madagascar and Seychelles. Data type: R code
File: oo_87293.pdf Esteves, F.A.

XML Treatment for Stigmatomma

XML Treatment for Stigmatomma besucheti

XML Treatment for Stigmatomma bolabola

XML Treatment for Stigmatomma irayhady

XML Treatment for Stigmatomma janovitsika

XML Treatment for Stigmatomma liebe

XML Treatment for Stigmatomma roahady

XML Treatment for Stigmatomma sakalava

XML Treatment for Stigmatomma tsyhady

## Figures and Tables

**Figure 1a.**
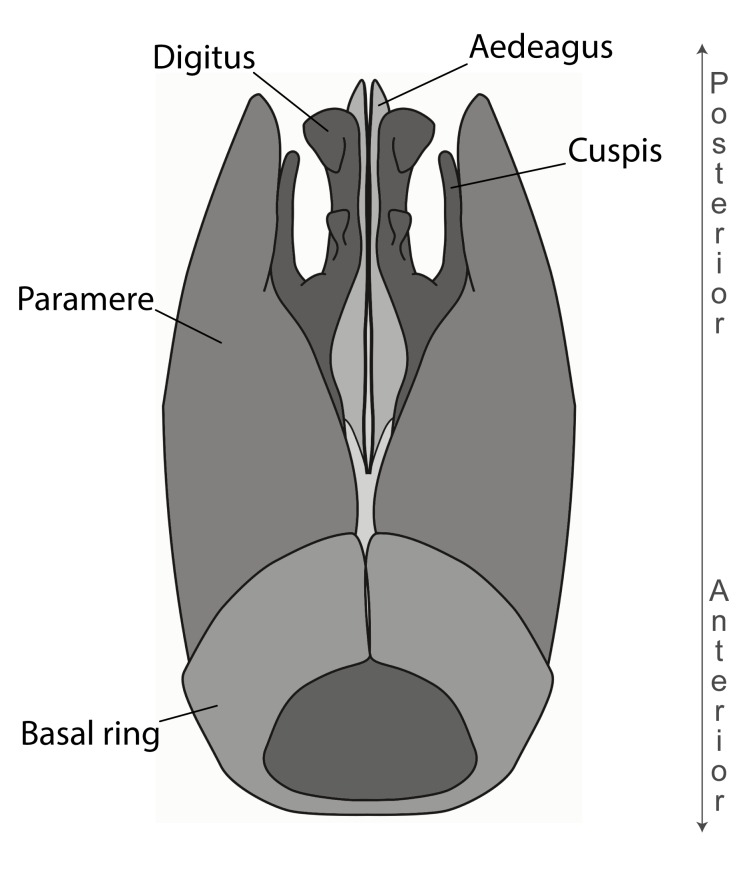
Genital capsule, ventral view. Illustration by F. A. Esteves.

**Figure 1b.**
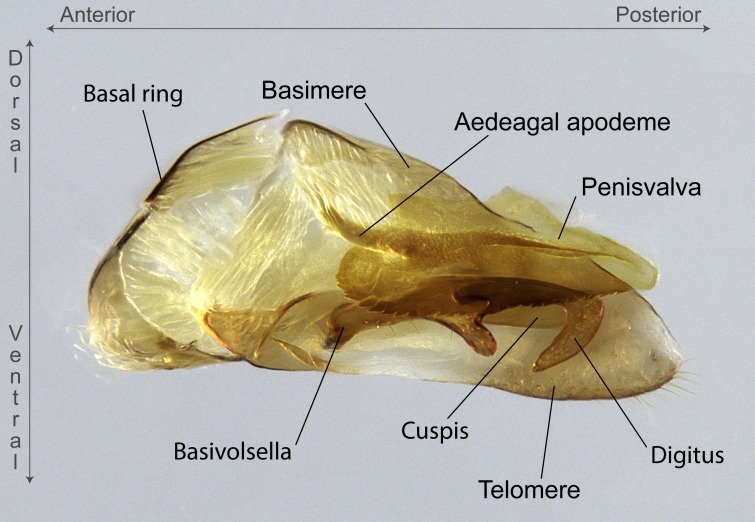
Genital capsule (CASENT0148201); longitudinal section, inner face. The aedeagal sclerite is darkened to enhance visibility. Image by F. A. Esteves.

**Figure 1c.**
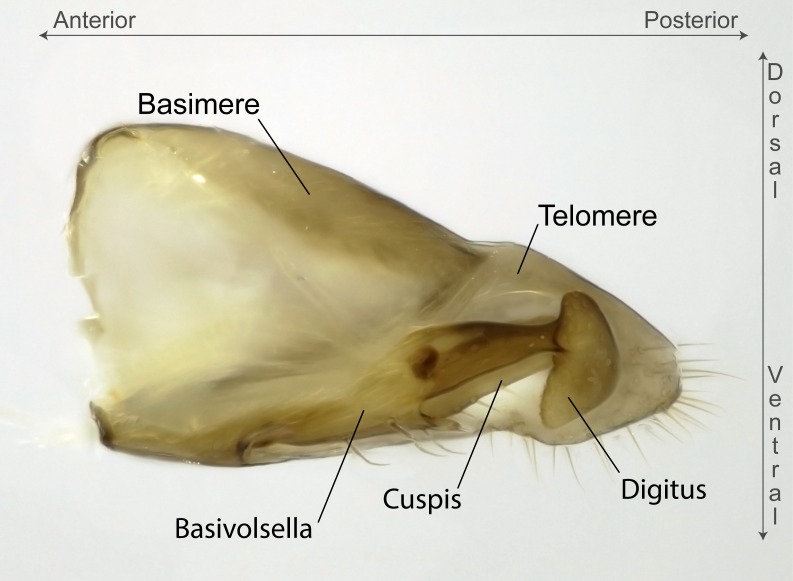
Genital capsule of *Stigmatomma janovitsika* **sp. n.** (CASENT0318446); longitudinal section, inner face. The basal ring and the aedeagus were removed from the capsule. Image by F. A. Esteves.

**Figure 1d.**
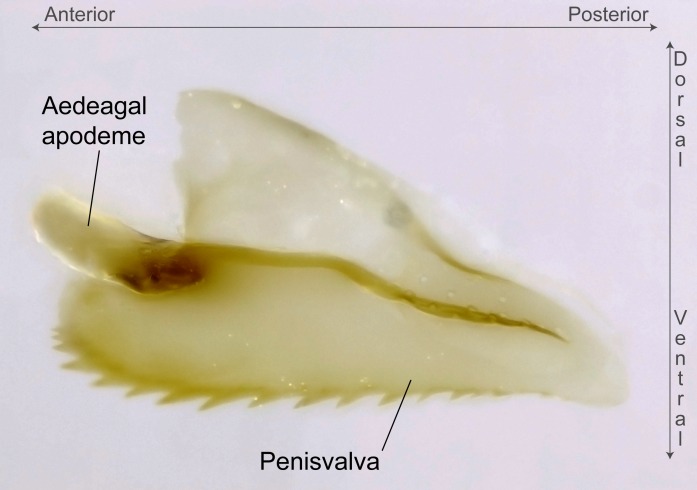
Right sclerite of the aedeagus of *Stigmatomma liebe* **sp. n.** (CASENT0724171); lateral view. Image by F. A. Esteves.

**Figure 2a.**
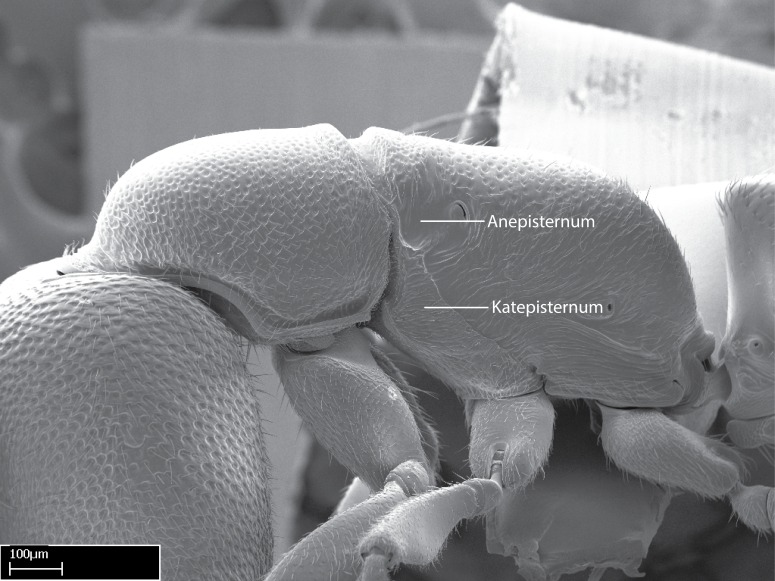
*Stigmatomma liebe* **sp. n.**, worker (CASENT0009102). The mesepisternum is divided in anepisternum and katepisternum by the median mesepisternal sulcus. Image by F. A. Esteves.

**Figure 2b.**
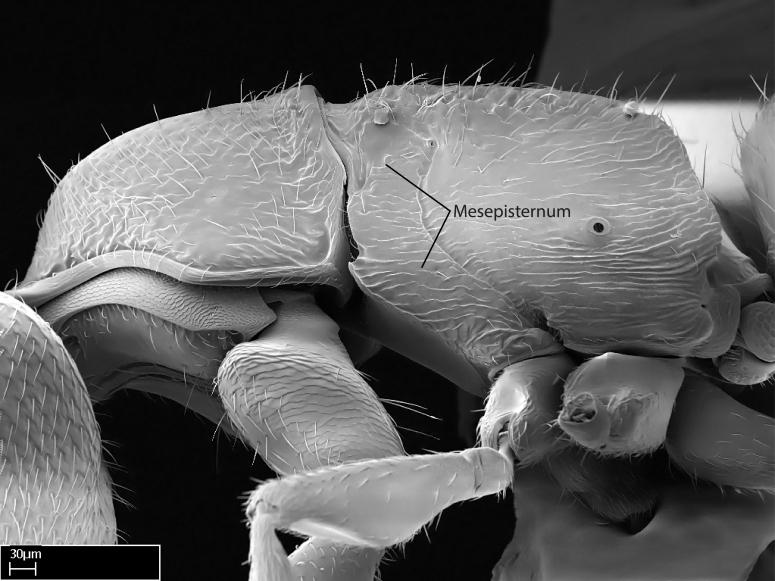
*Stigmatomma janovitsika* **sp. n.**, worker (CASENT0145426). The mesepisternum is not divided into anepisternum and katepisternum by the median mesepisternal sulcus. Image by F. A. Esteves.

**Figure 3a.**
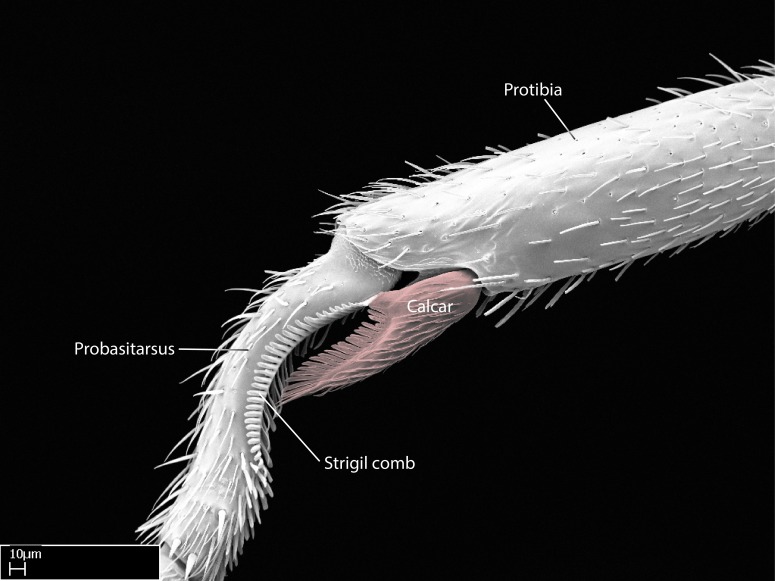
Foreleg of *Stigmatomma bolabola* **sp. n.** (CASENT0034744); posterior face. Calcar highlighted in red. Image and illustration by F. A. Esteves.

**Figure 3b.**
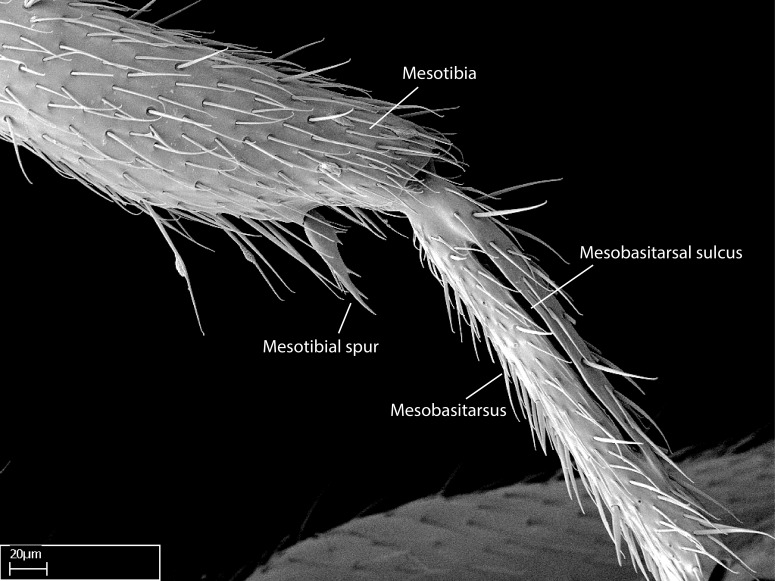
Midleg of *Stigmatomma sakalava* **sp. n.** (CASENT0438262); anterior face. Image by F. A. Esteves.

**Figure 3c.**
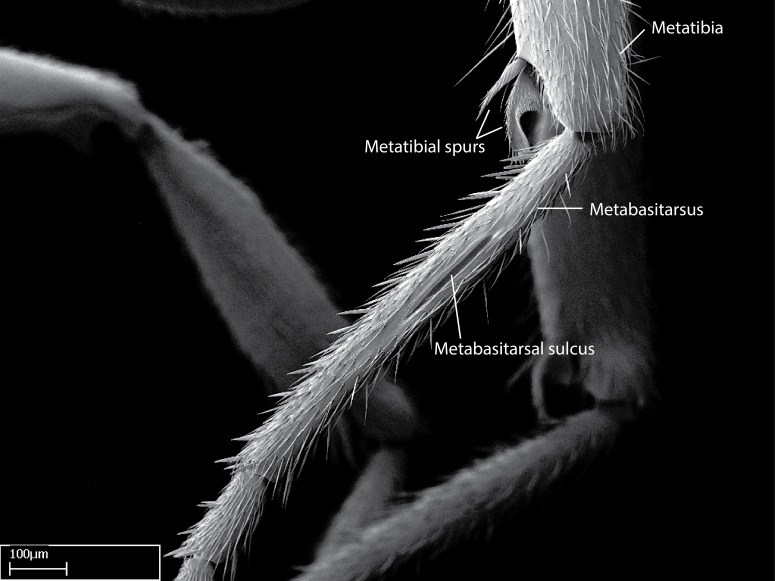
Hindleg of *Stigmatomma roahady* ​**sp. n.** (CASENT0056916); anterior face. Image by F. A. Esteves.

**Figure 3d.**
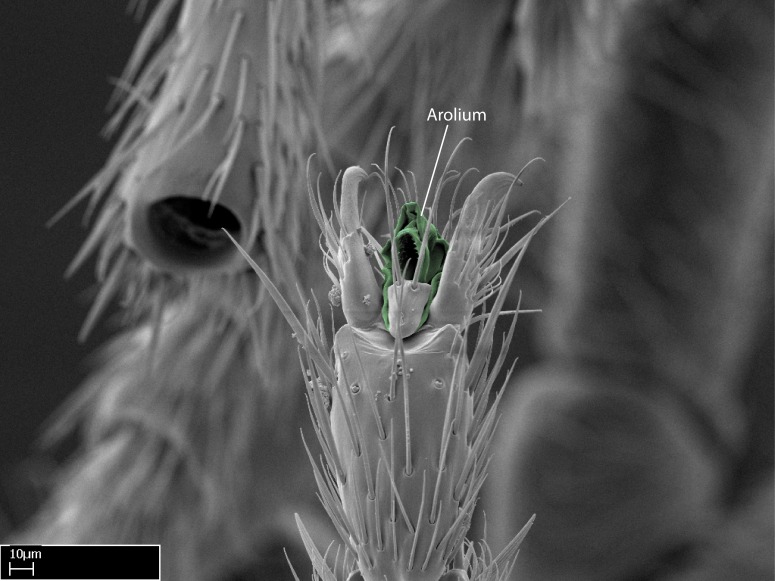
Midleg of *Stigmatomma roahady* **sp. n.** (CASENT0002078); dorsal view: fifth tarsomere and pretarsal claw. Arolium highlighted in green. Image and illustration by F. A. Esteves.

**Figure 4a.**
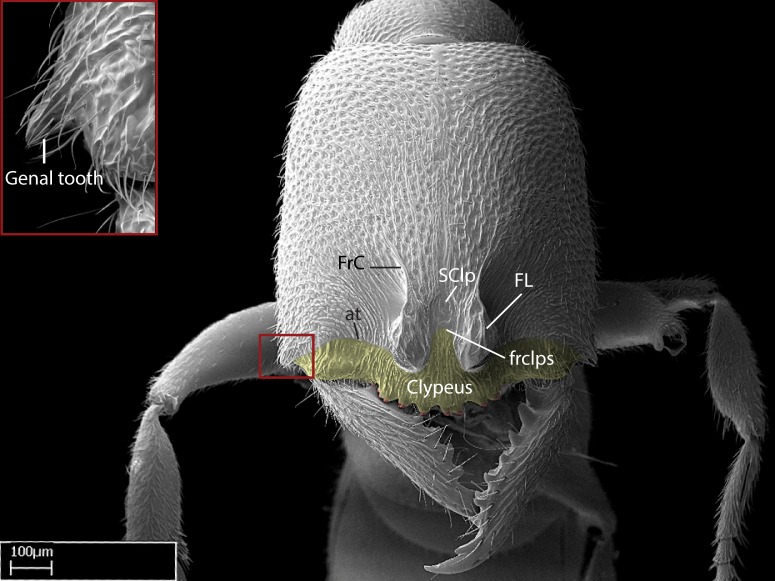
Head of *Stigmatomma liebe* **sp. n.** worker (CASENT0009102); fullface view. Clypeus highlighted in yellow. Abbreviations: at, anterior tentorial pit; FL, frontal lobe; FrC, frontal carina; frclps, frontoclypeal sulcus; SClp, supraclypeal area. Image and illustration by F. A. Esteves.

**Figure 4b.**
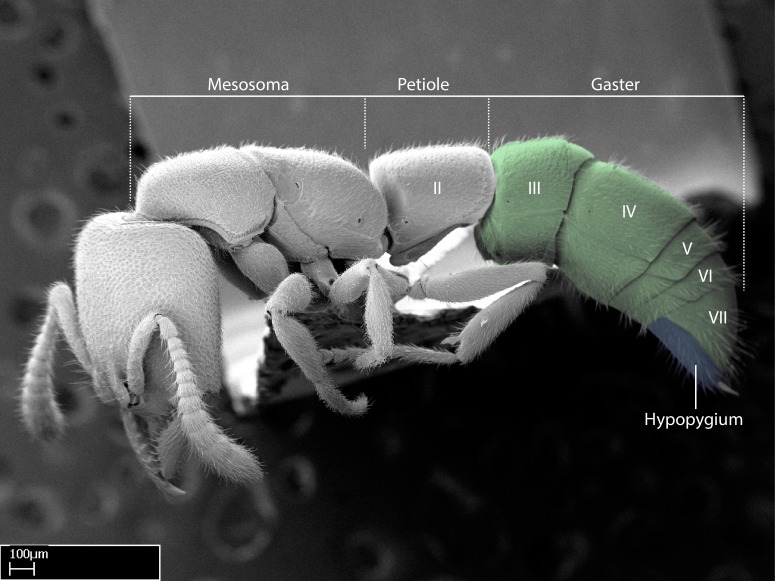
*Stigmatomma liebe* **sp. n.** worker (CASENT0318414); lateral view. Gaster highlighted in green and blue; hypopygium in blue. Abdominal segments are labeled by corresponding roman numeral. Image and illustration by F. A. Esteves.

**Figure 5a.**
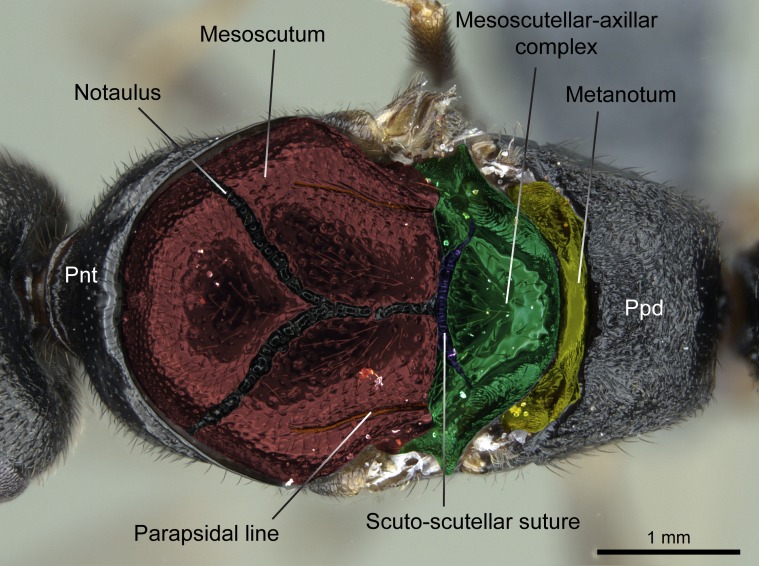
Mesosoma of *Stigmatomma tsyhady* **sp. n.** (CASENT0723249); male; dorsal face. Mesoscutum highlighted in red, mesoscutellar-axillar complex in green, scuto-scutellar suture in purple, and metanotum in yellow. Wings were removed for better illustration. Abbreviations: Pnt, pronotum; Ppd, propodeum. Image and illustration by F. A. Esteves.

**Figure 5b.**
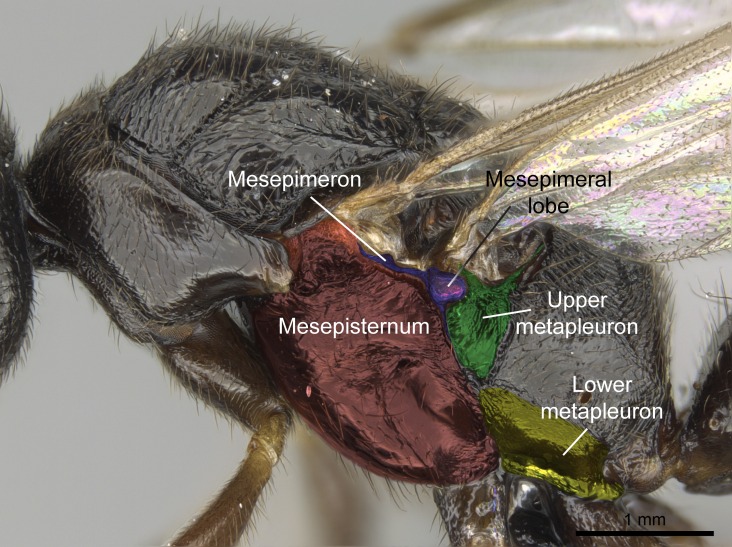
Mesosoma of *Stigmatomma roahady* **sp. n.** (CASENT0107483); male; lateral face. Mesepisternum highlighted in red, mesepimeron in purple, lower metapleuron in yellow, and upper metapleuron in green. Image and illustration by F. A. Esteves.

**Figure 6a.**
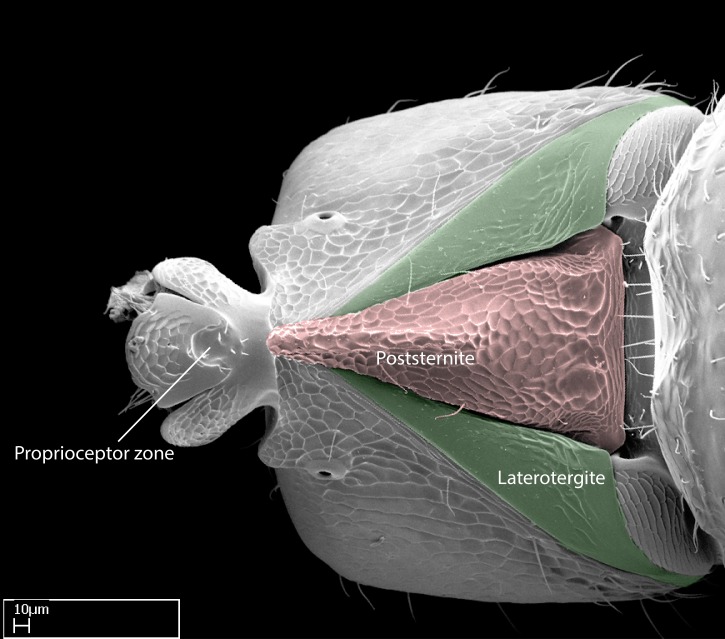
Petiole of *Stigmatomma sakalava* **n. sp.**, ventral view (CASENT0022146). Petiolar laterotergite is highlighted in red. Image and illustration by F. A. Esteves.

**Figure 6b.**
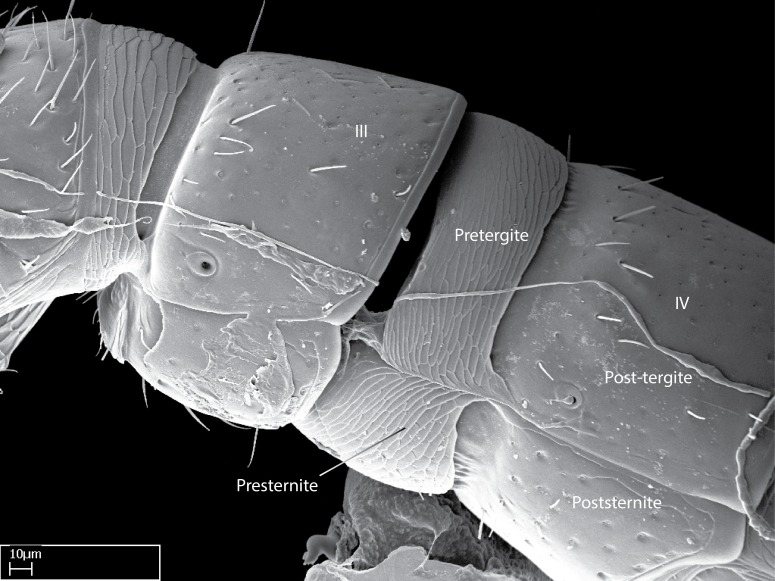
Gaster of *Stigmatomma besucheti*, lateral view (CASENT0101970). Abdominal segments are labeled by corresponding roman numeral. Image by F. A. Esteves.

**Figure 7a.**
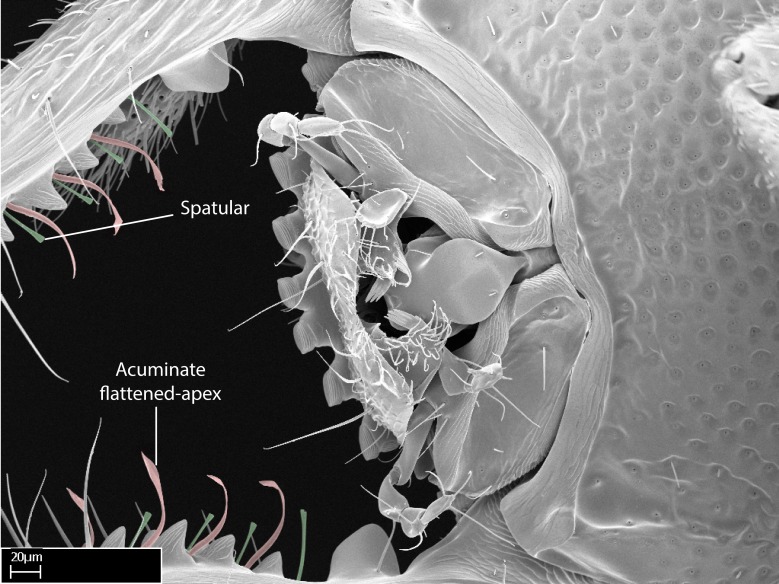
Head of *Stigmatomma janovitsika* **sp. n.** worker (CASENT0318418): ventral view of mandibles and mouth parts. Acuminate flattened-apex setae are highlighted in red. Statular setae are highlighted in green. Image and illustration by F. A. Esteves.

**Figure 7b.**
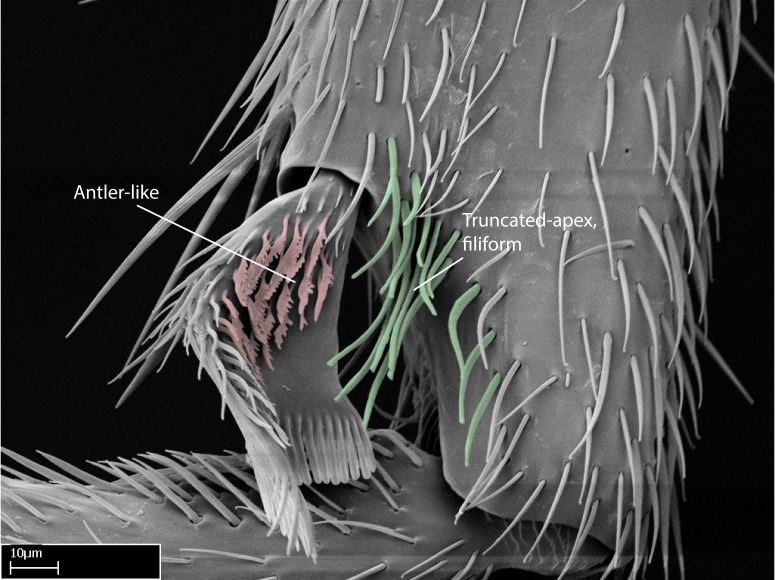
Hindleg of *Stigmatomma janovitsika* **sp. n.** worker (CASENT0145426): posterior face of posterior metatibial spur. Antler-like microtrichia are highlighted in red. Truncated-apex filiform setae are highlighted in green. Image and illustration by F. A. Esteves.

**Figure 7c.**
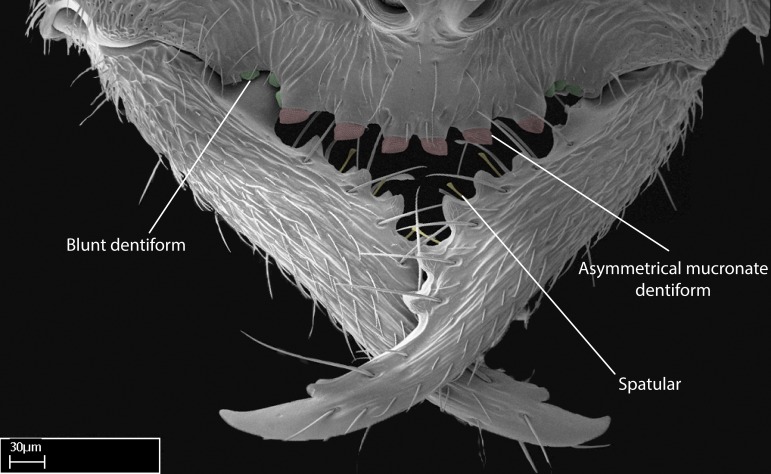
Head of *Stigmatomma janovitsika* **sp. n.** worker (CASENT0145426): dorsal face of mandibles and clypeal area. Blunt dentiform setae are highlighted in green. Asymmetrical mucronate dentiform setae are highlighted in red. Spatular setae are seen in yellow. Image and illustration by F. A. Esteves.

**Figure 7d.**
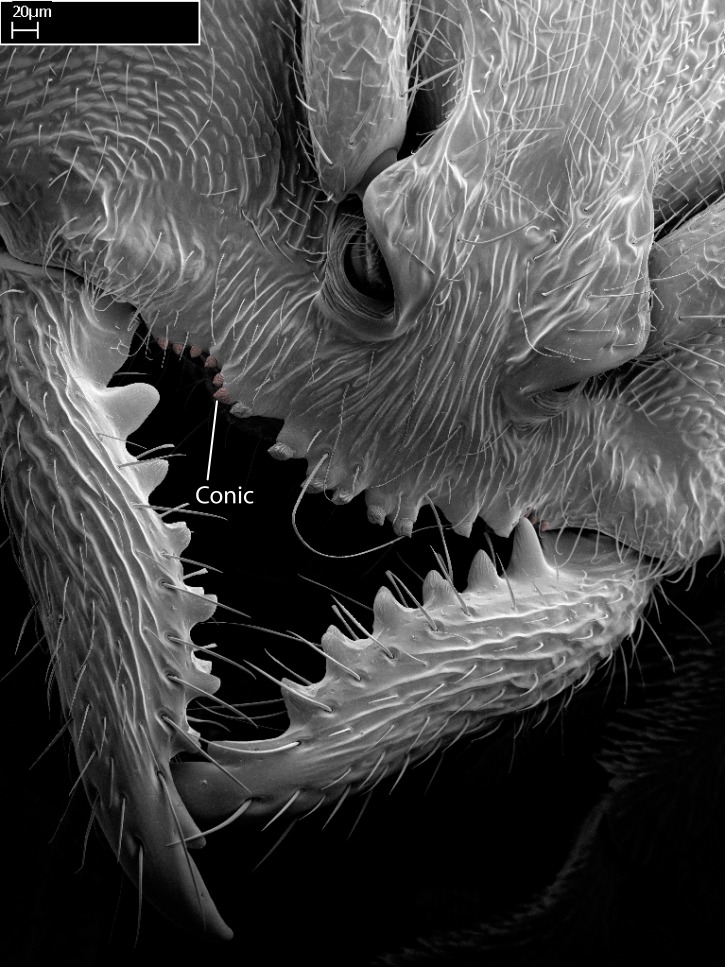
Head of *Stigmatomma liebe* **sp. n.** worker (CASENT0318414): dorsal face of mandibles and anterior part of the head. Conic setae are highlighted in red. Image and illustration by F. A. Esteves.

**Figure 7e.**
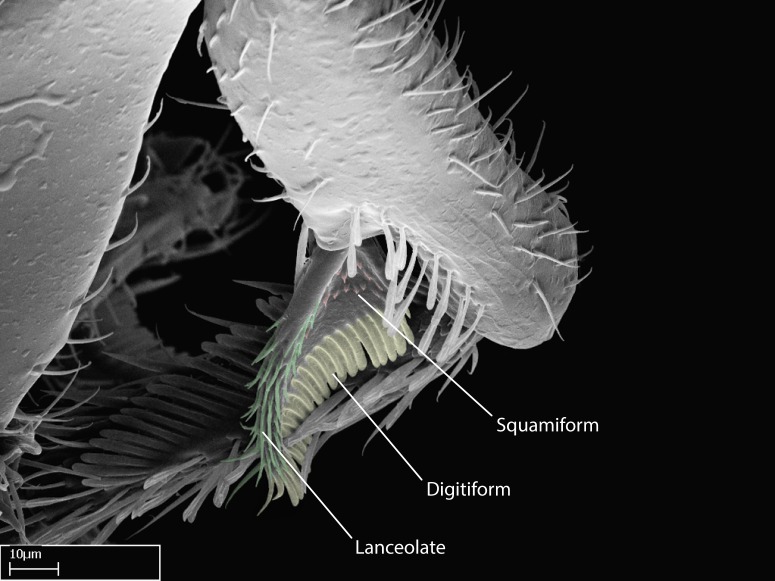
Foreleg of *Stigmatomma besucheti* worker (CASENT0101970): anterior face of calcar of strigil. Digitiform cuticular projections are highlighted in yellow. Squamiform microtrichia are highlighted in red. Lanceolate microtrichia are seen in green. Image and illustration by F. A. Esteves.

**Figure 7f.**
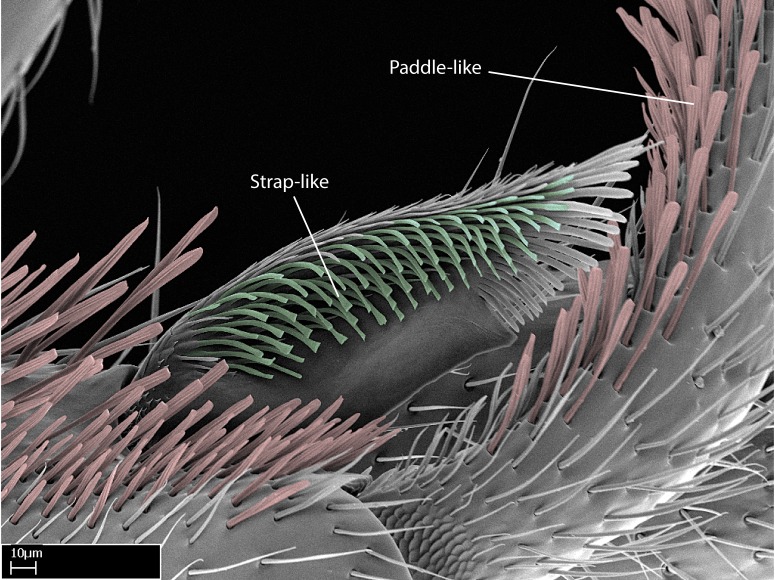
Foreleg of *Stigmatomma roahady* **sp. n.** worker (CASENT0002078): anterior face of calcar of strigil. Paddle-like setae are highlighted in red, and strap-like setae in green. Image and illustration by F. A. Esteves.

**Figure 8a.**
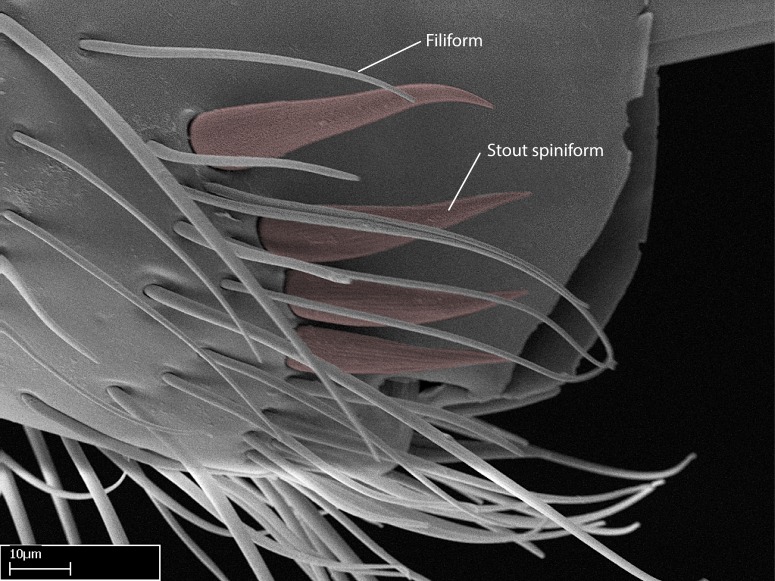
Seventh abdominal segment of *Stigmatomma janovitsika* **sp. n.** worker (CASENT0145426): lateral view of hypopygium. Stout spiniform setae are highlighted in red, while filiform setae appear in gray. Image and illustration by F. A. Esteves.

**Figure 8b.**
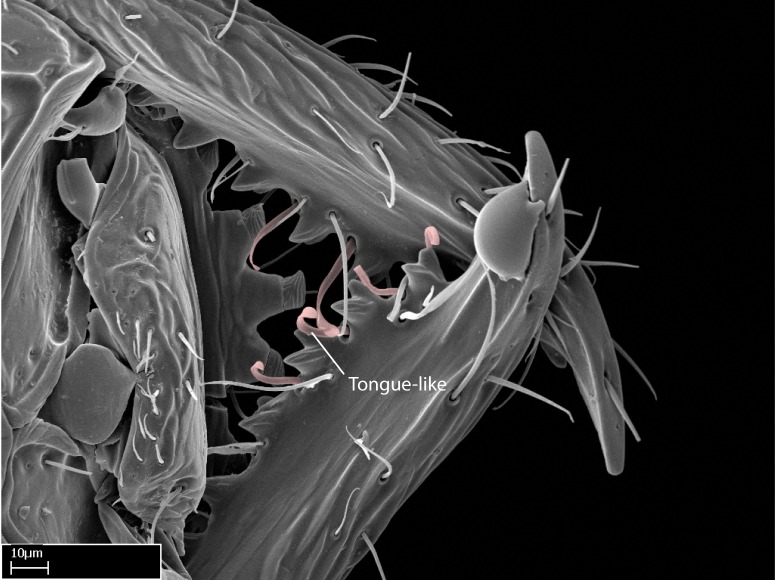
Head of *Stigmatomma besucheti* worker (CASENT0101970): ventral view of mandibles and mouth parts. Tongue-like setae are highlighted in red. Image and illustration by F. A. Esteves.

**Figure 8c.**
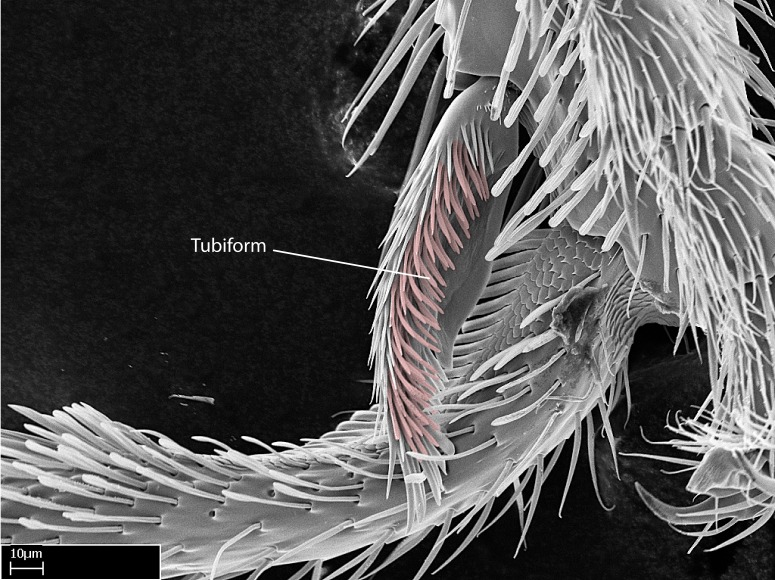
Foreleg of *Stigmatomma sakalava* **sp. n.** worker (CASENT0017556): anterior face of calcar of strigil and mesobasitarsus. Tubiform setae are highlighted in red. Image and illustration by F. A. Esteves.

**Figure 12a.**
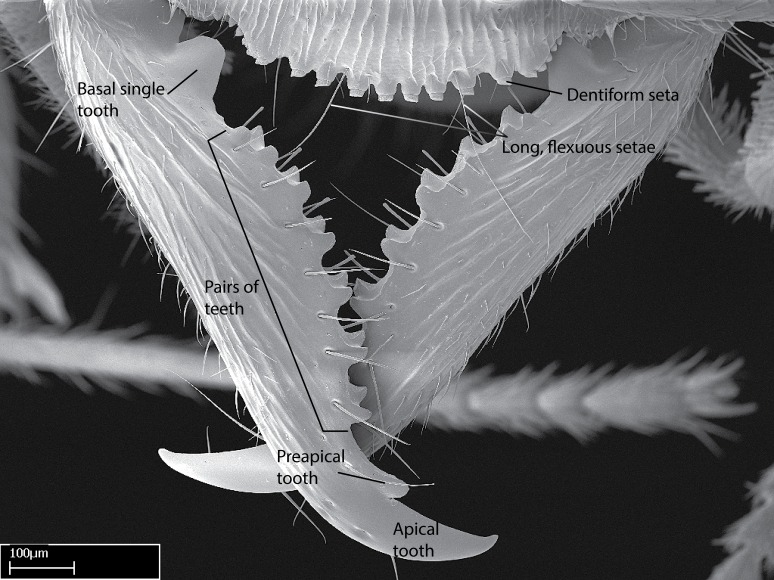
Mandibles of *Stigmatomma roahady* **sp. n.** worker; dorsal view (CASENT0004339). Teeth distribution layout indicated in the figure. Note the enlarged most basal tooth. Image by F. A. Esteves; available at AntWeb.org

**Figure 12b.**
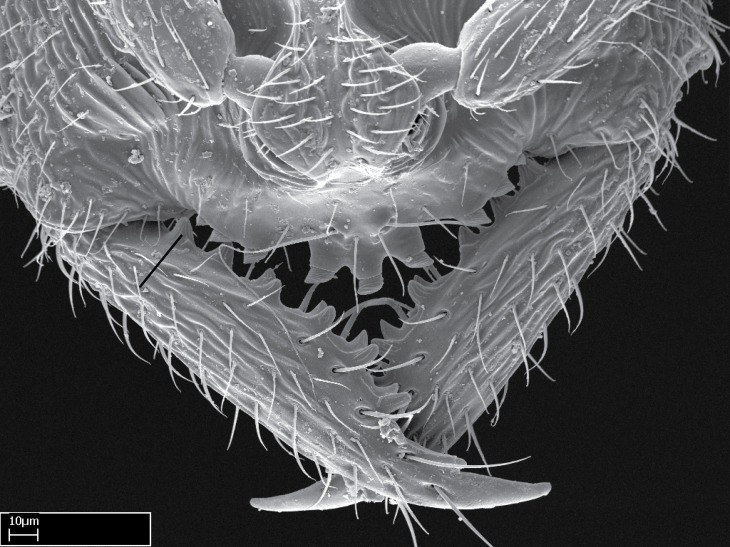
Mandibles of *Stigmatomma besucheti* Baroni-Urbani worker; dorsal view (CASENT0906833). The arrow highlights the most basal tooth, which is similar in size with the more apical teeth. Image by F. A. Esteves; available at AntWeb.org

**Figure 16a.**
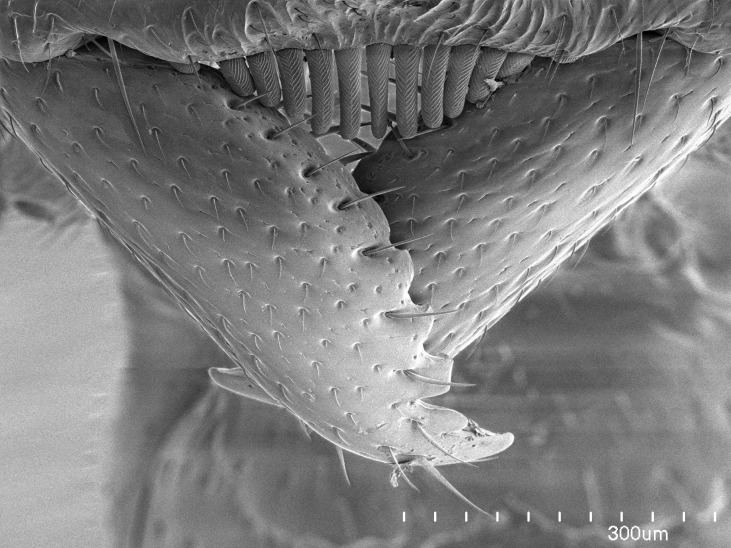
Dorsal view of the mandibles illustrates the absence of pairs of teeth. Image by Roberto Keller; available at AntWeb.org

**Figure 16b.**
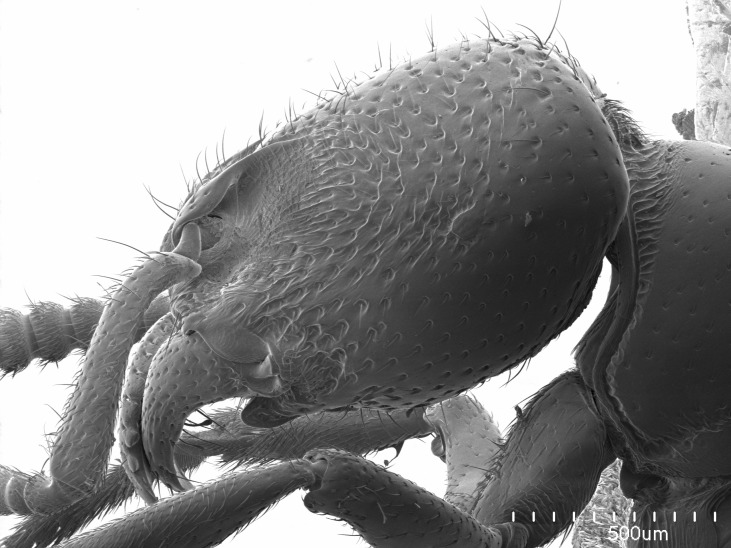
Lateral view of the head, which confirms that mandibles have no basal teeth paired with mandibular teeth. Image by Roberto Keller; available at AntWeb.org

**Figure 17a.**
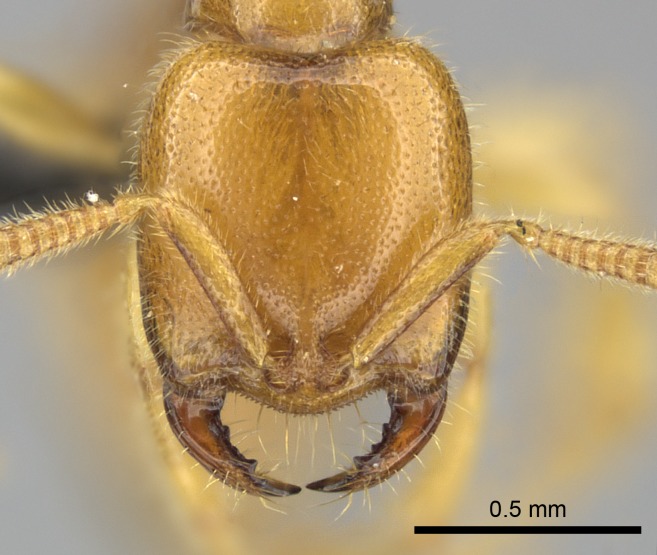
Fullface view of *Adetomyrma bressleri* worker (CASENT0205995). Image by Ryan Perry; available at AntWeb.org

**Figure 17b.**
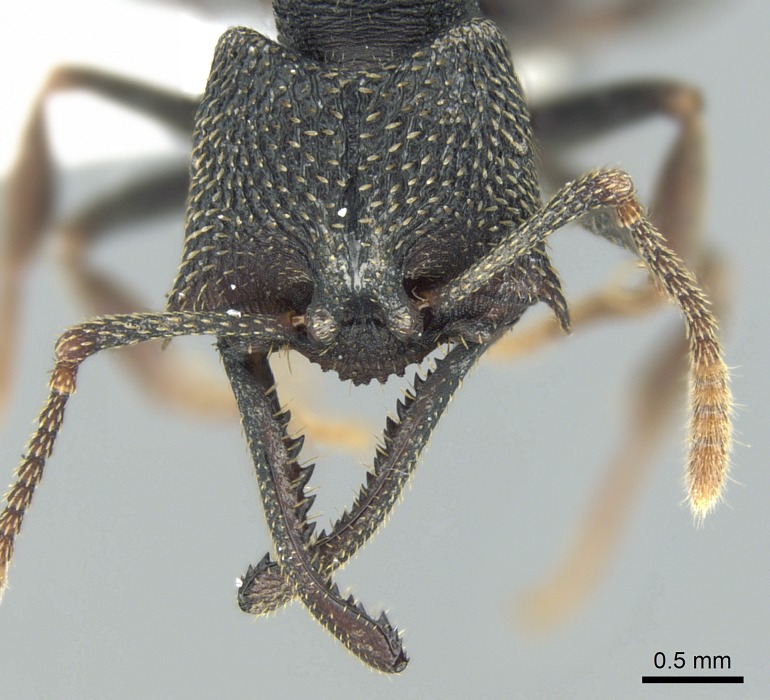
Fullface view of *Mystrium eques* worker (CASENT0317390). Image by Estella Ortega; available at AntWeb.org

**Figure 17c.**
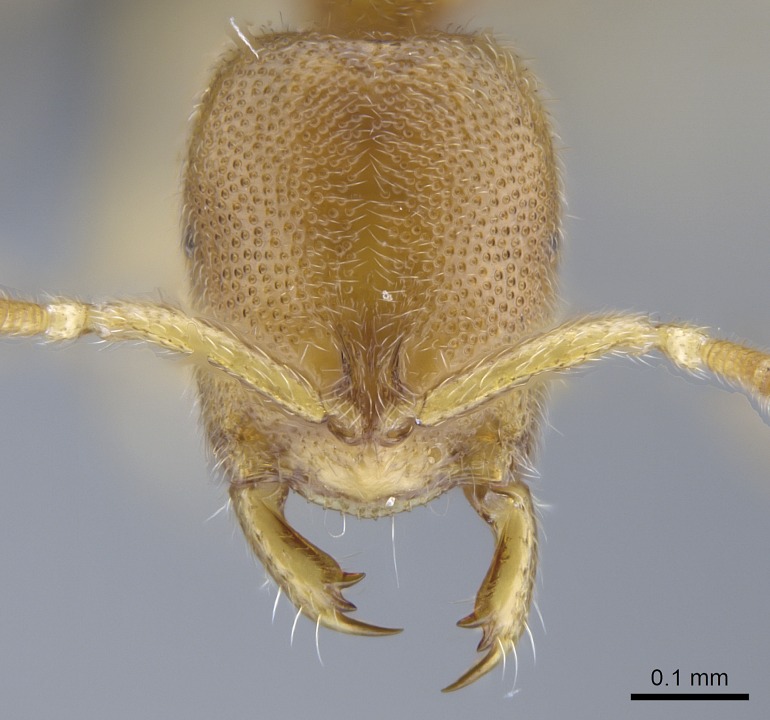
Fullface view of *Prionopelta descarpentriesi* worker (CASENT0034837). Image by Rick Overson; available at AntWeb.org

**Figure 17d.**
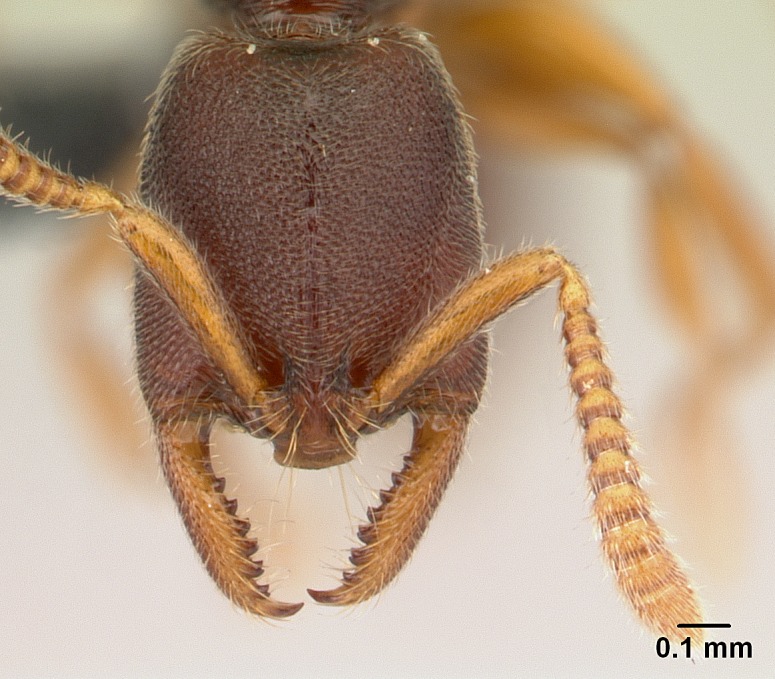
Fullface view of *Xymmer* mg04 worker (CASENT0151732). Image by Erin Prado; available at AntWeb.org

**Figure 18a.**
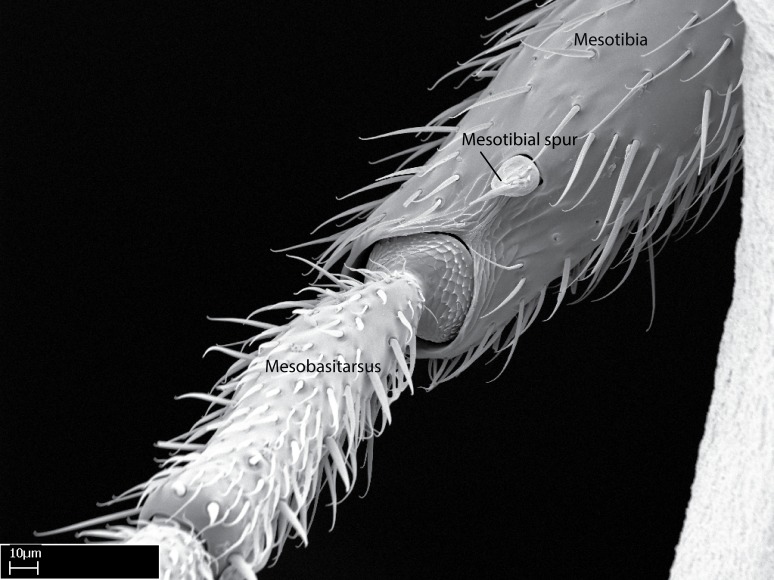
Mesotibia of *Stigmatomma bolabola* **sp. n.** worker (CASENT0034744). Inner face of the apical portion featuring a single spur. Image by F. A. Esteves; available at AntWeb.org

**Figure 18b.**
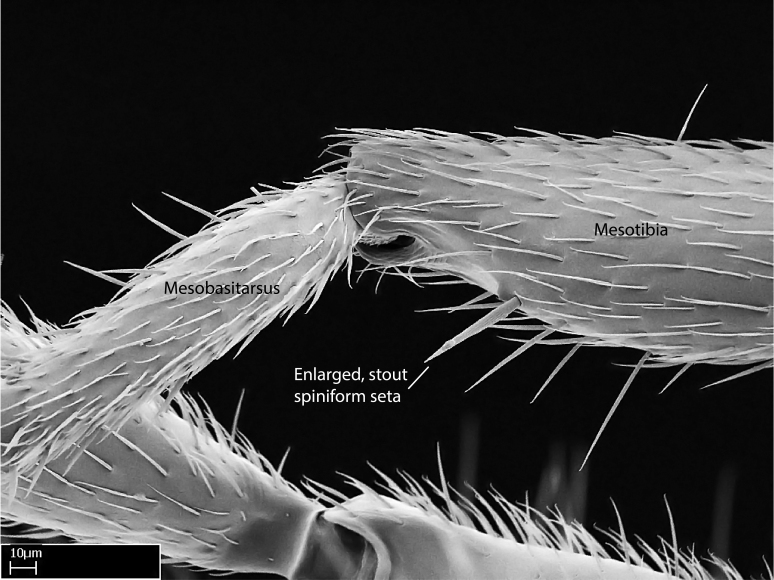
Mesotibia of *Stigmatomma janovitsika* **sp. n.** worker (CASENT0145426). Posterior face of the apical portion featuring an enlarged seta. Image by F. A. Esteves; available at AntWeb.org

**Figure 19a.**
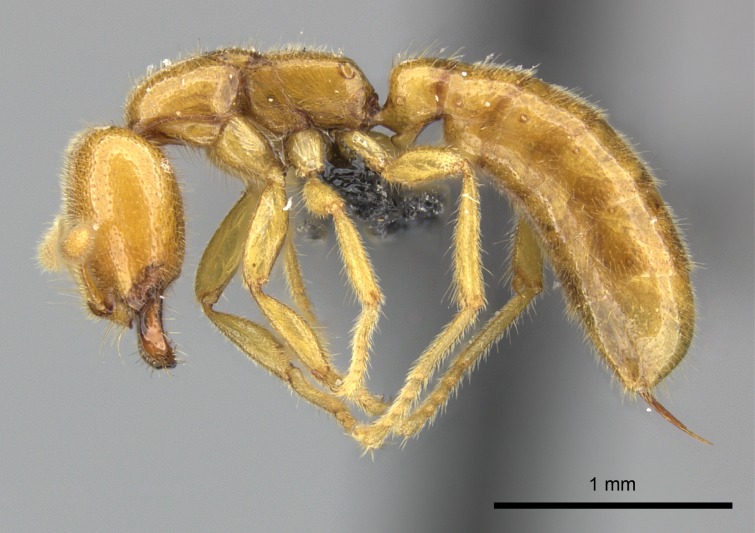
*Adetomyrma barrybressleri* worker, lateral view (CASENT0205995). Image by Ryan Perry; available at AntWeb.org

**Figure 19b.**
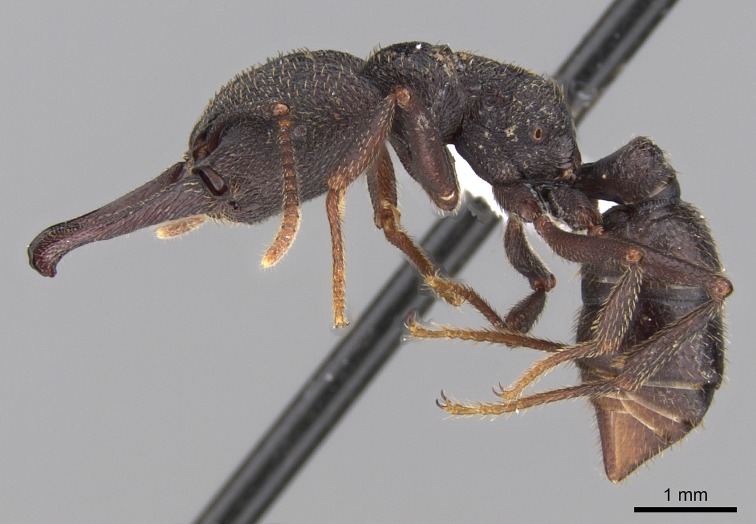
*Mystrium mysticum* worker, lateral view (CASENT0429959). Image by Cerise Chen; available at AntWeb.org

**Figure 19c.**
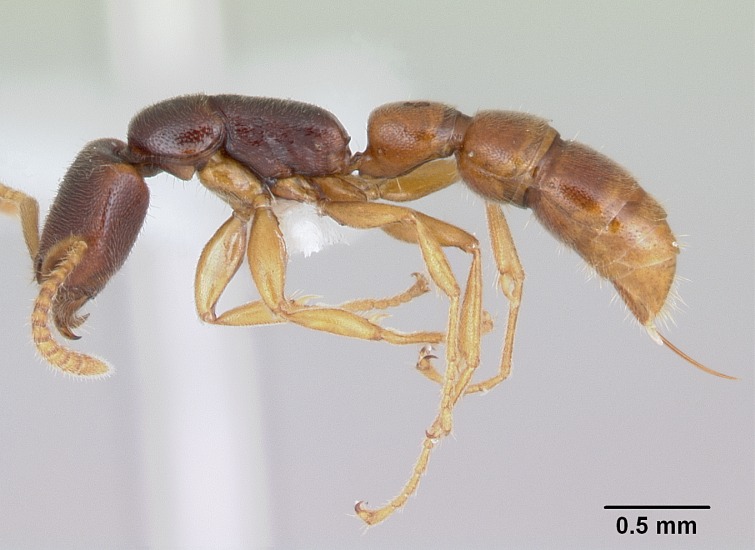
*Xymmer* mg01 worker, lateral view (CASENT0004310). Image by April Nobile; available at AntWeb.org

**Figure 19d.**
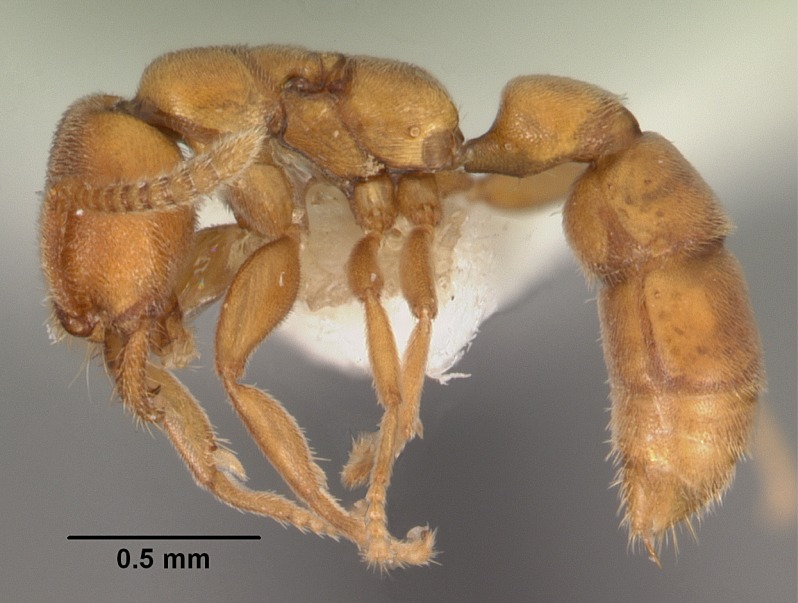
*Xymmer muticus* dealated queen, lateral view (Afrotropical bioregion; CASENT0102213). Image by April Nobile; available at AntWeb.org

**Figure 20a.**
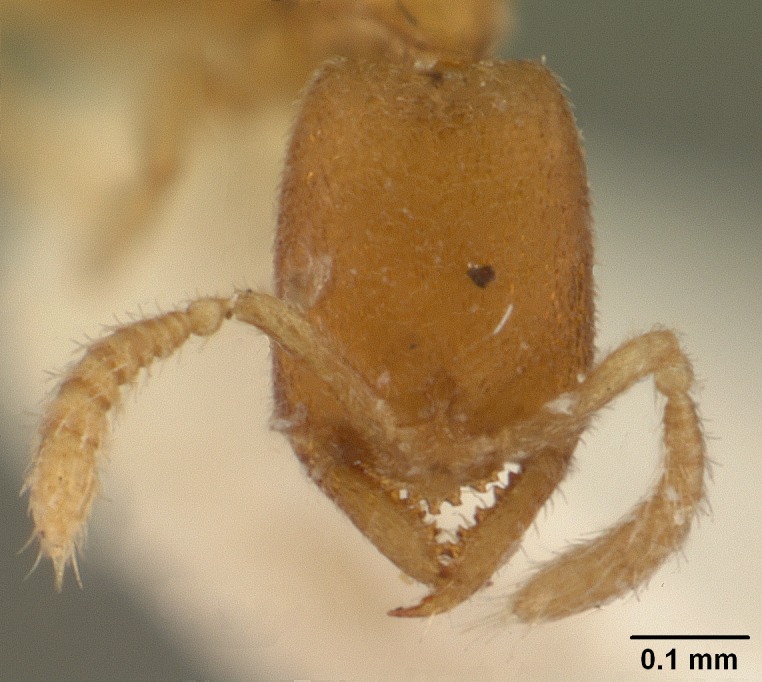
Fullface view.

**Figure 20b.**
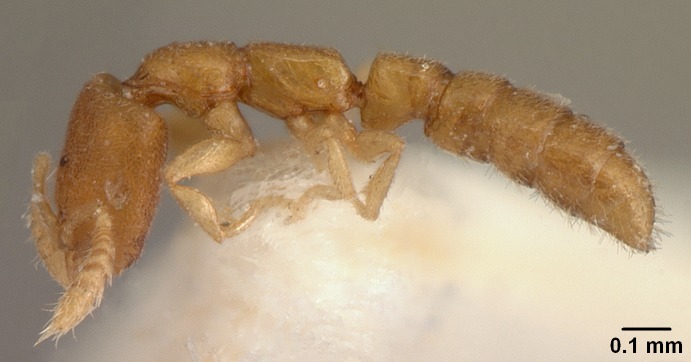
Lateral view.

**Figure 20c.**
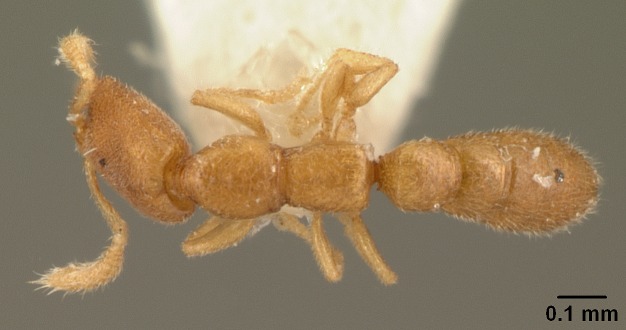
Dorsal view.

**Figure 21a.**
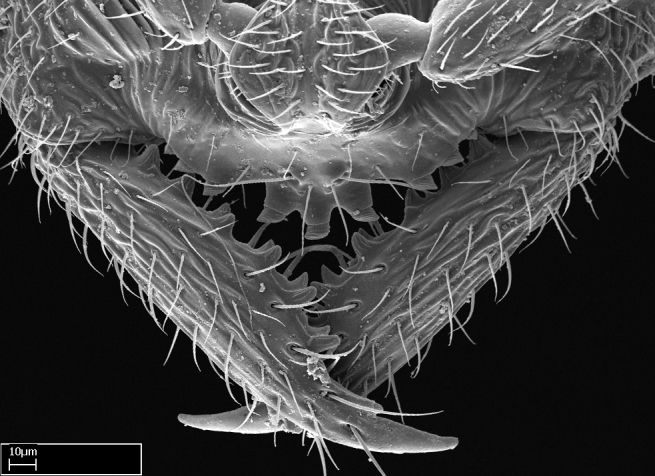
Dorsal view of mandibles and anterior part of the head (CASENT0906833). Image by F. A. Esteves; available at AntWeb.org

**Figure 21b.**
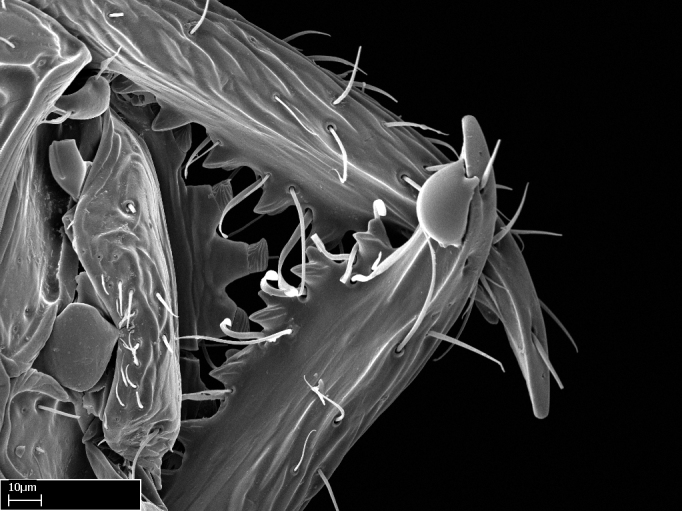
Ventral view of mandibles and mouth parts (CASENT0101970). Image by F. A. Esteves; available at AntWeb.org

**Figure 21c.**
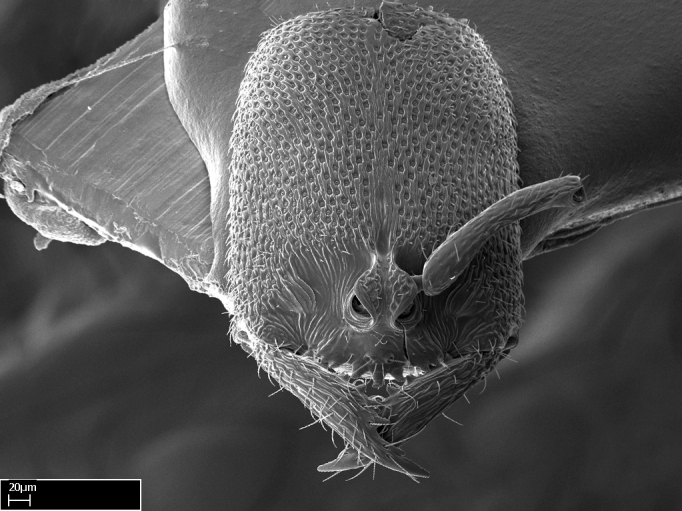
Fullface view (CASENT0101970). Image by F. A. Esteves; available at AntWeb.org

**Figure 21d.**
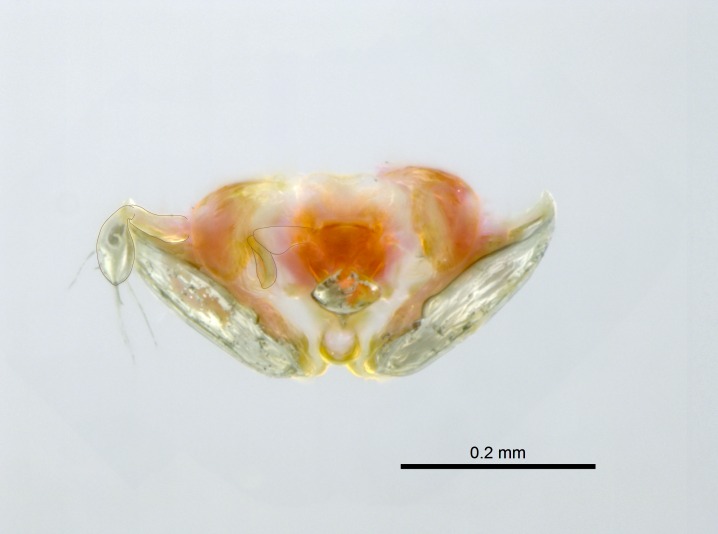
Anteroventral view of mouth parts (CASENT0101970). Left maxillary palp is missing. Right maxillary and labial palps are outlined in gray to enhance visibility. Slide and image by F. A. Esteves; available at AntWeb.org

**Figure 22a.**
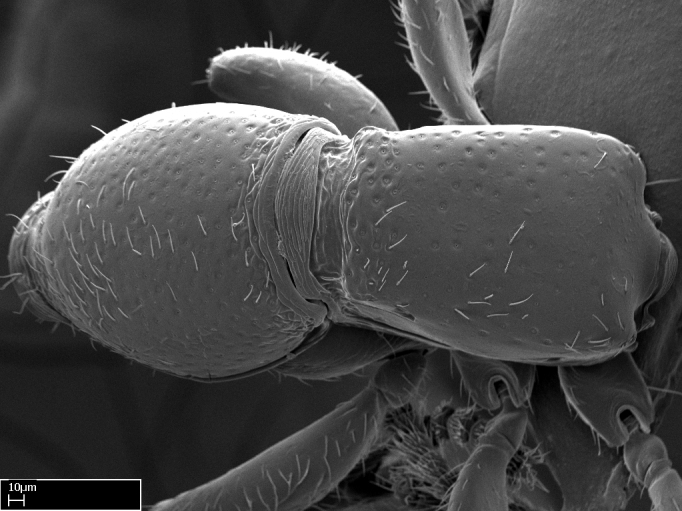
Dorsal view.

**Figure 22b.**
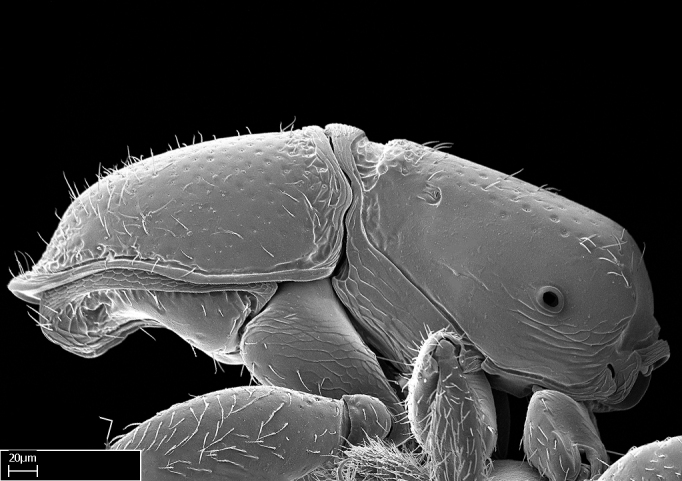
Lateral view.

**Figure 23a.**
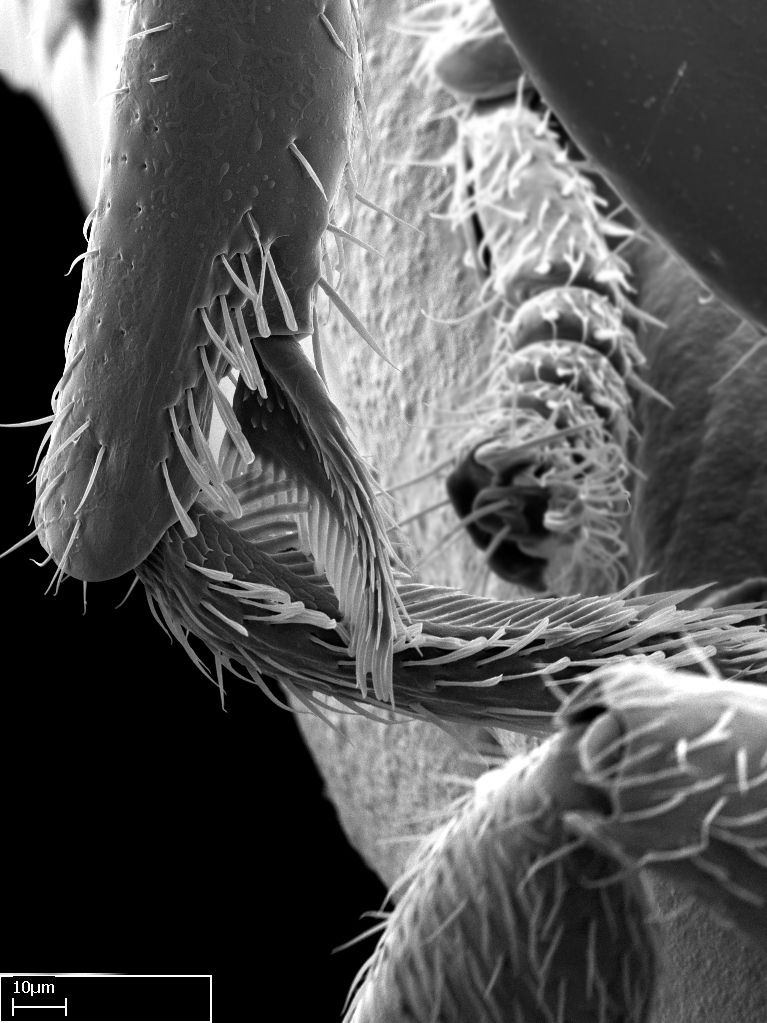
Foreleg (CASENT0101970): anterior face of protibia, bearing the calcar of strigil, and probasitarsus.

**Figure 23b.**
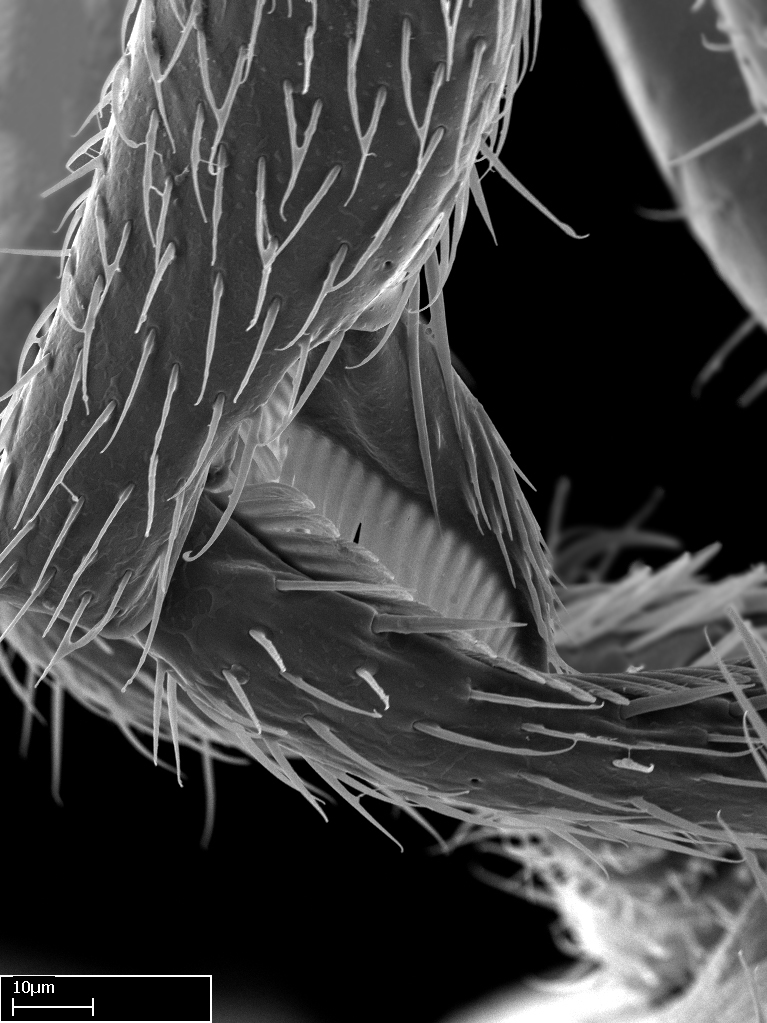
Foreleg (CASENT0101970): posterior face of protibia, bearing the calcar of strigil, and probasitarsus.

**Figure 23c.**
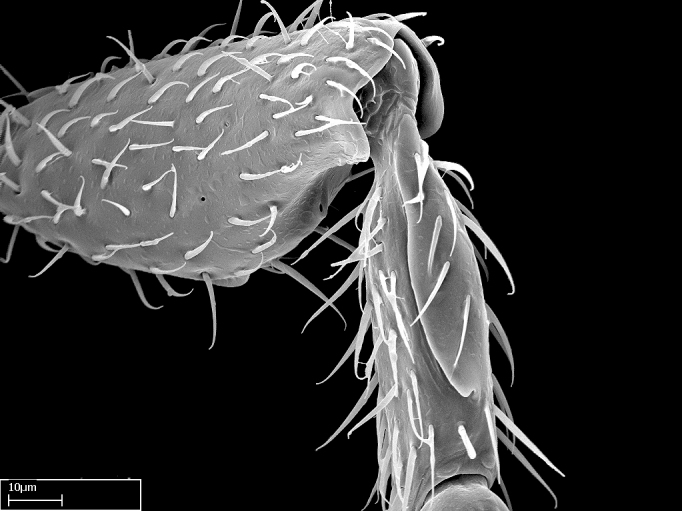
Midleg (CASENT0101970): anteroventral face of mesotibia and dorsoposterior face of mesobasitarsus, which possesses a longitudinal slit-like sulcus.

**Figure 23d.**
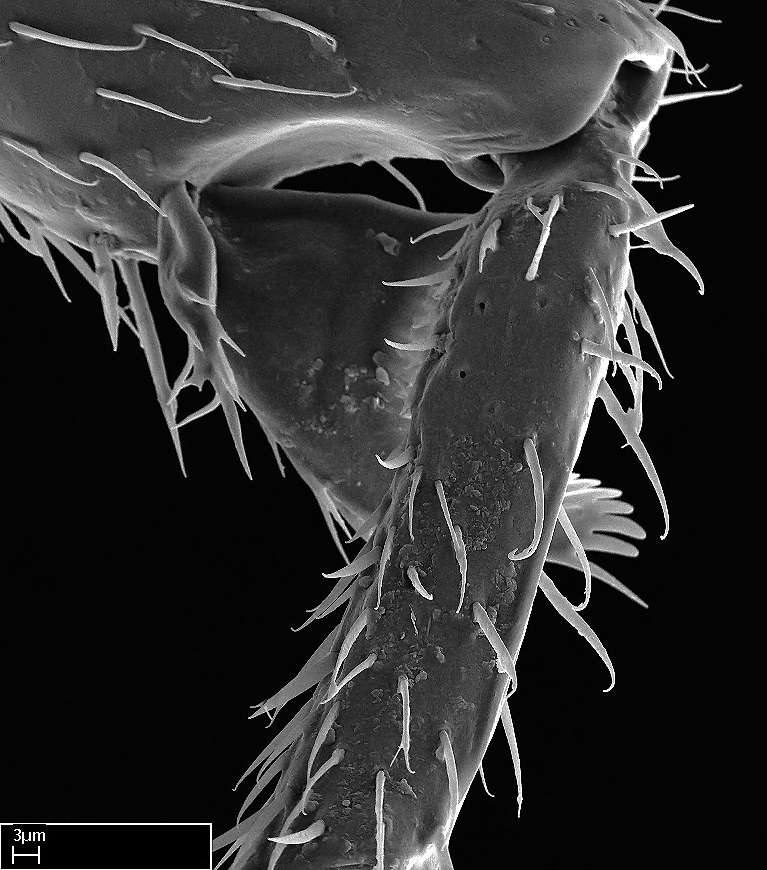
Hindleg (CASENT0101970): anterior face of metatibia, bearing two spurs apically, and metabasitarsus.

**Figure 23e.**
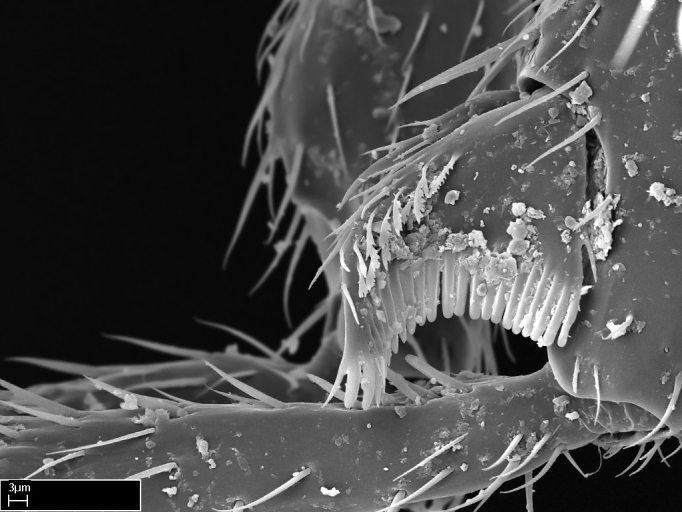
Hindleg (CASENT0906833): posterior face of metatibia (apical part), associated posterior spur, and metabasitarsus.

**Figure 23f.**
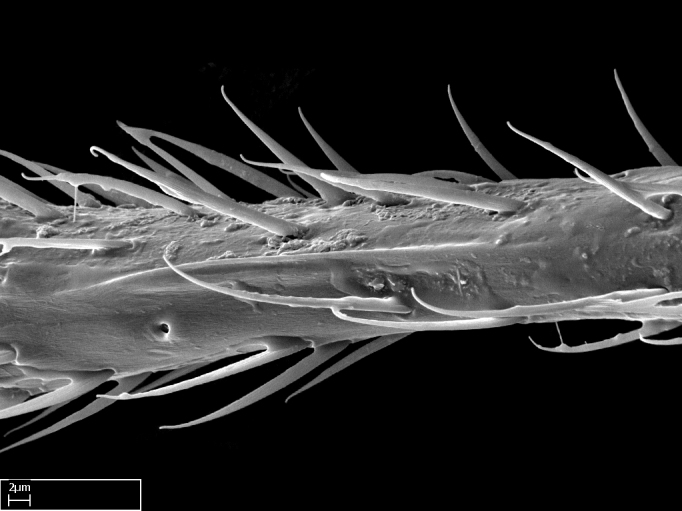
Hindleg (CASENT0101970); dorsal face of metatibia: closeup of its two parallel carinae with convergent apexes.

**Figure 24a.**
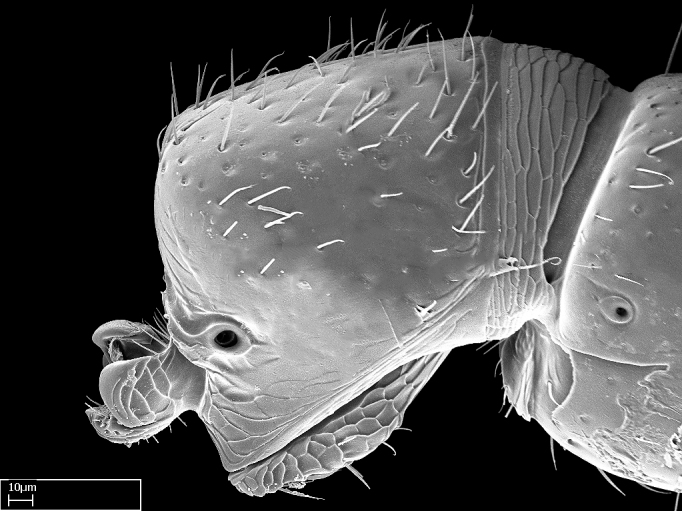
Petiole; lateral view.

**Figure 24b.**
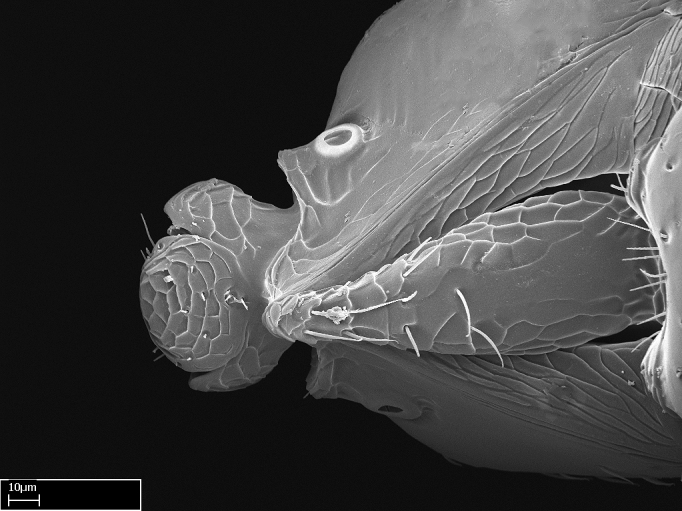
Petiole, ventral view. Left part of the image: petiolar anterior articular end, bearing reduced proprioceptor zone.

**Figure 24c.**
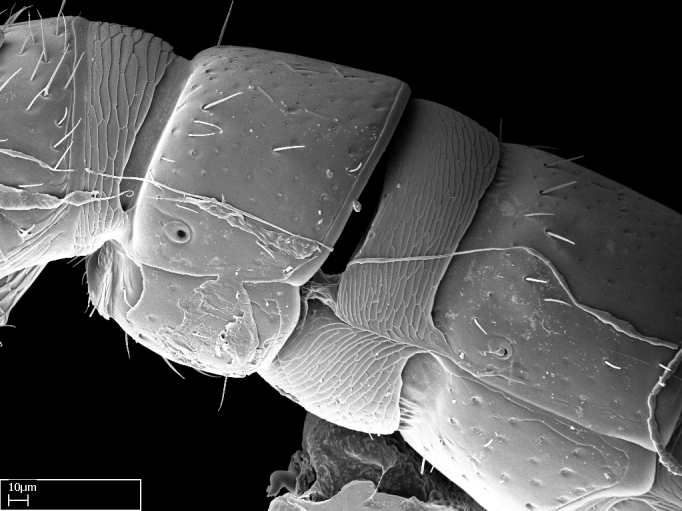
Posterior part of the petiole, abdominal segment III, and abdominal segment IV; lateral view.

**Figure 24d.**
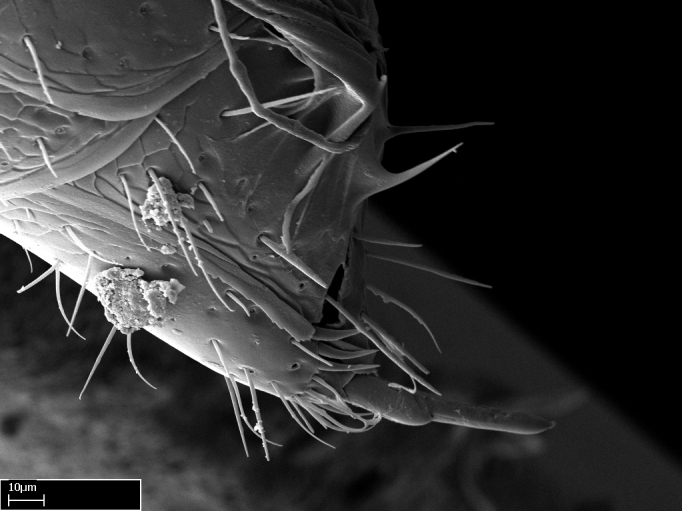
Abdominal segment VII (including hypopygium and associated stout spiniform setae); lateral view.

**Figure 25a.**
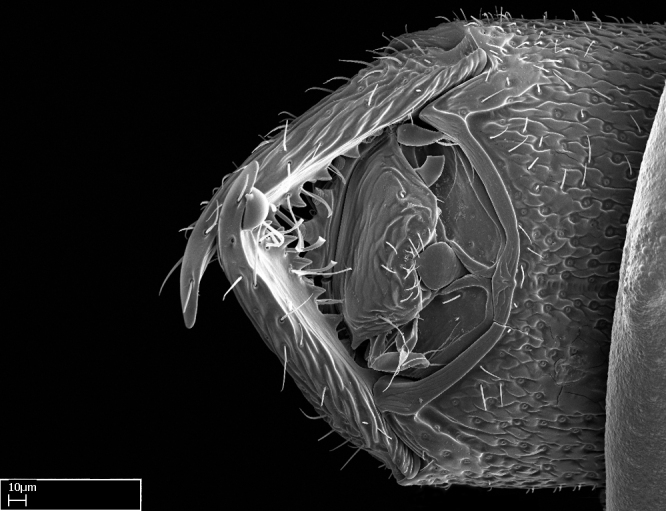
Ventral view of mandibles, mouth parts, and anterior part of the head.

**Figure 25b.**
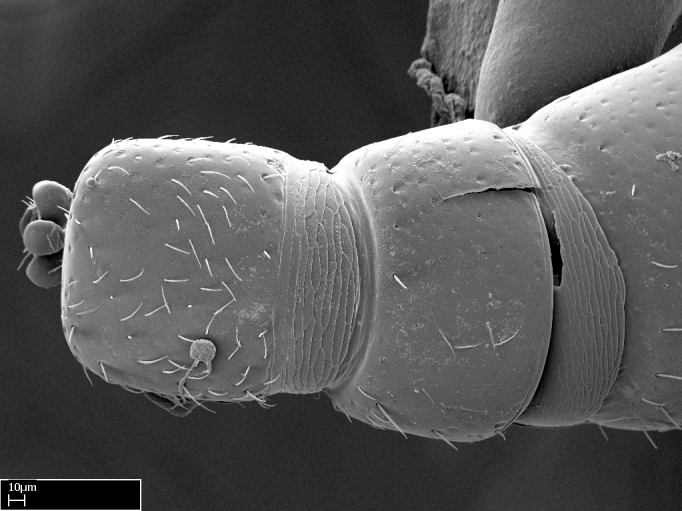
Dorsal view of petiole, Abdominal tergite III, and anterior part of abdominal tergite IV.

**Figure 25c.**
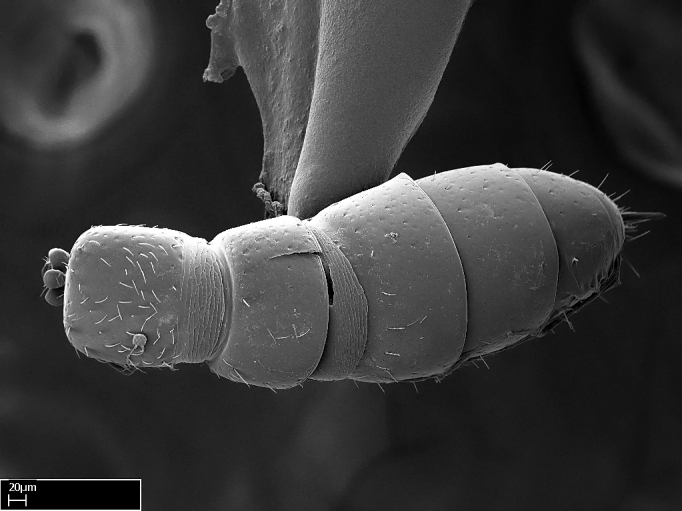
Dorsal view of petiole and gaster.

**Figure 27a.**
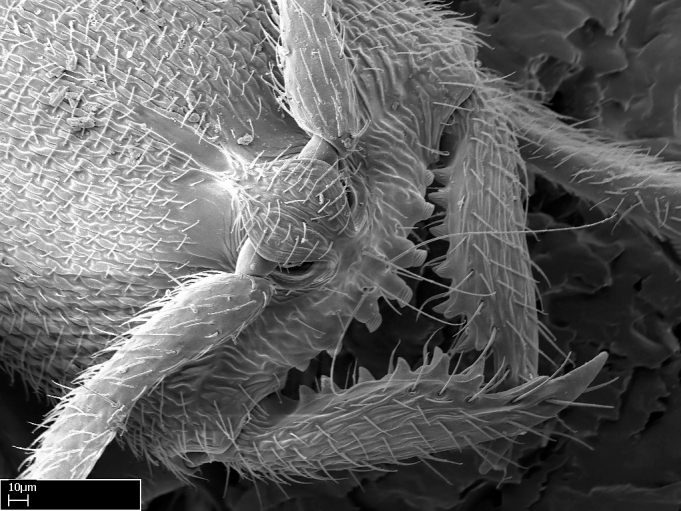
Dorsal view of the anterior part of the head.

**Figure 27b.**
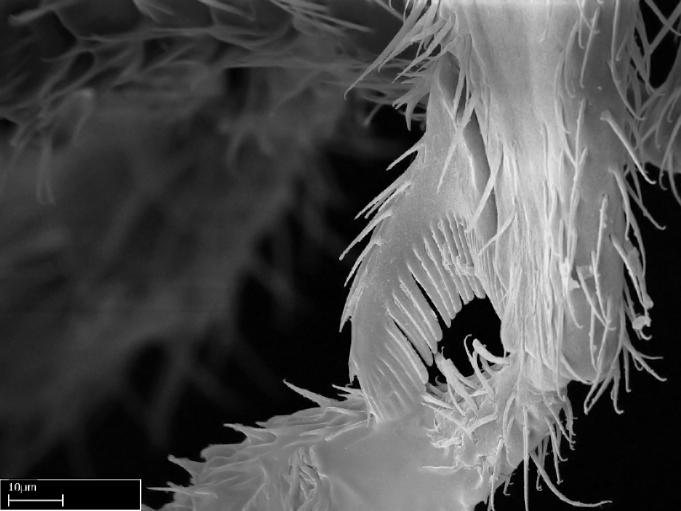
Close-up view of the posterior face of the metatibial apex, posterior metatibial spur, and basal region of metabasitarsus.

**Figure 28a.**
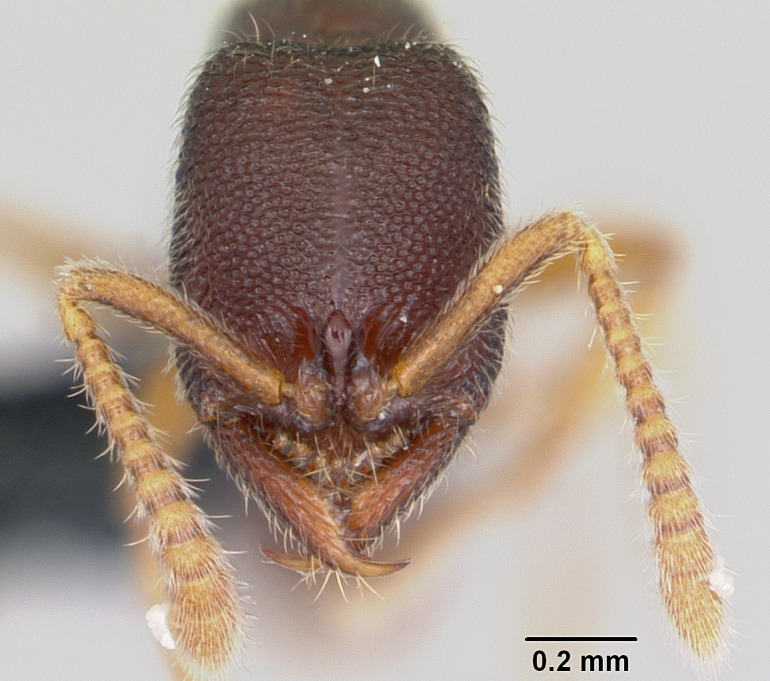
Fullface view.

**Figure 28b.**
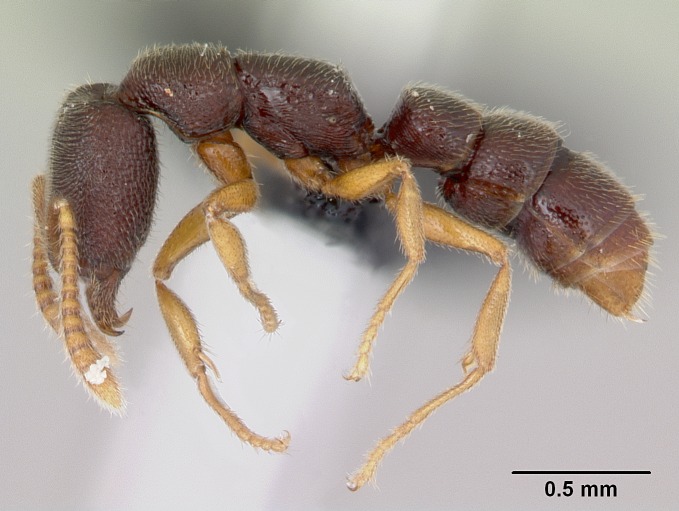
Lateral view.

**Figure 28c.**
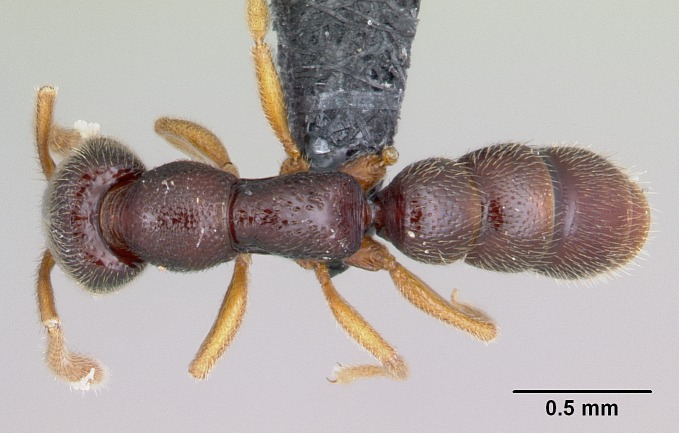
Dorsal view.

**Figure 29a.**
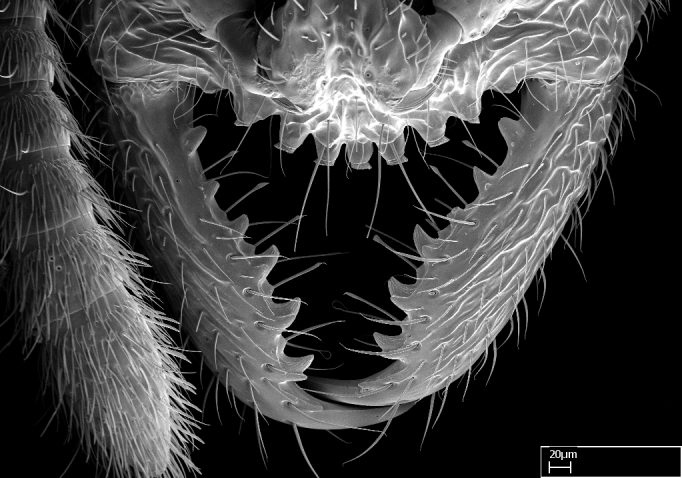
Dorsal view of mandibles and anterior portion of the head.

**Figure 29b.**
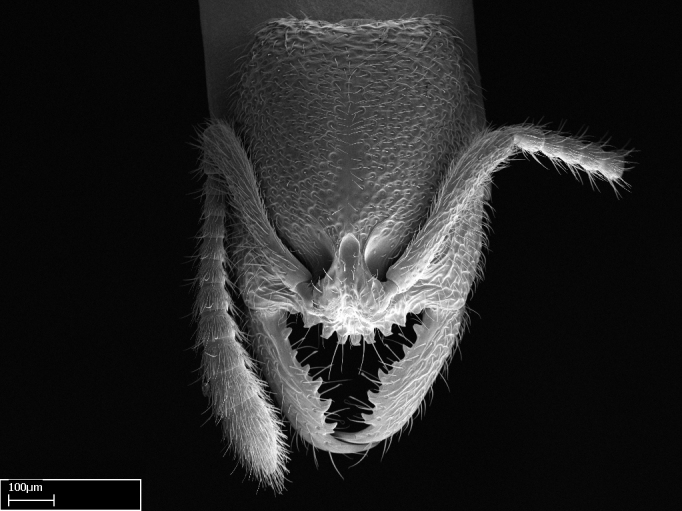
Fullface view.

**Figure 29c.**
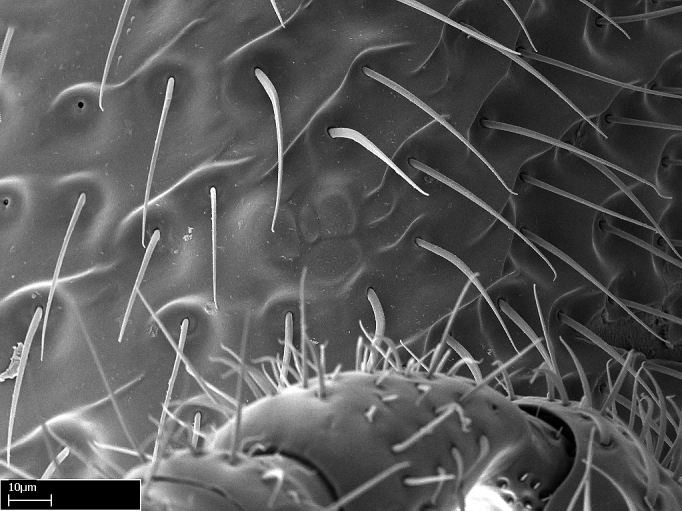
Close up of the eyes.

**Figure 29d.**
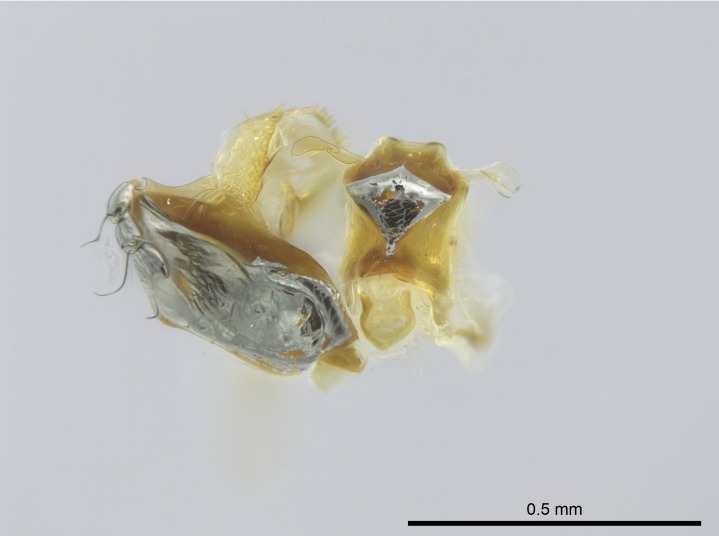
Ventral view of the mouthparts. Left stipe and left maxillary palp are missing, and the mouthparts are partially coated in gold-palladium. Right maxillary and labial palps are outlined in gray and darkened to enhance visibility. Slide by F. A. Esteves

**Figure 30a.**
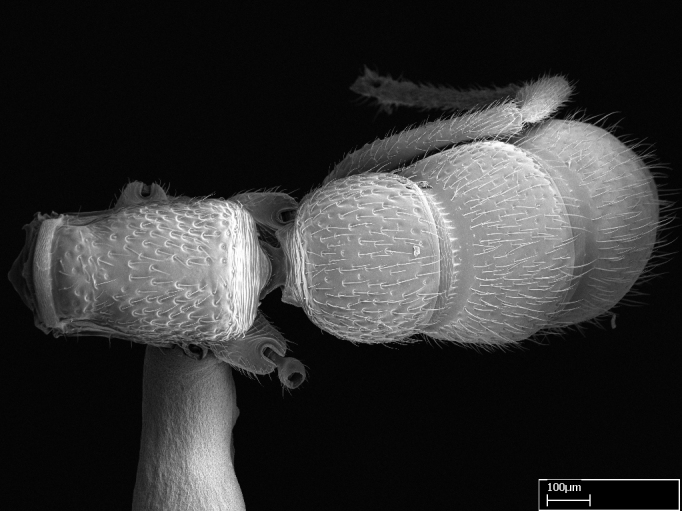
Dorsal view. Pronotum is disarticulated and is missing in the image.

**Figure 30b.**
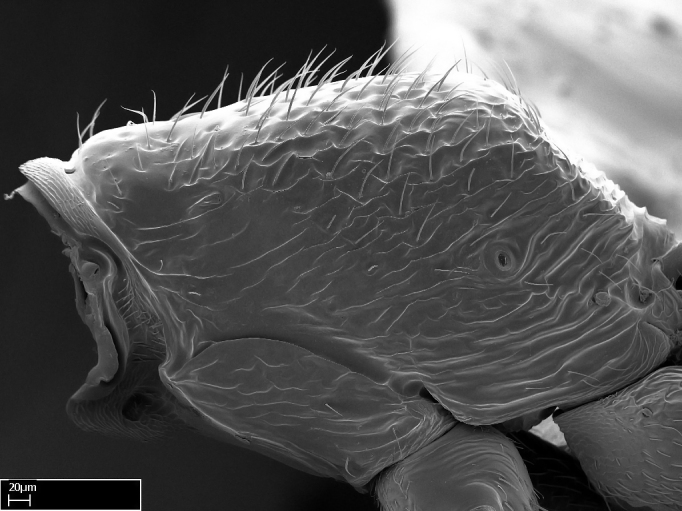
Lateral view. Pronotum is disarticulated and is missing in the image.

**Figure 31a.**
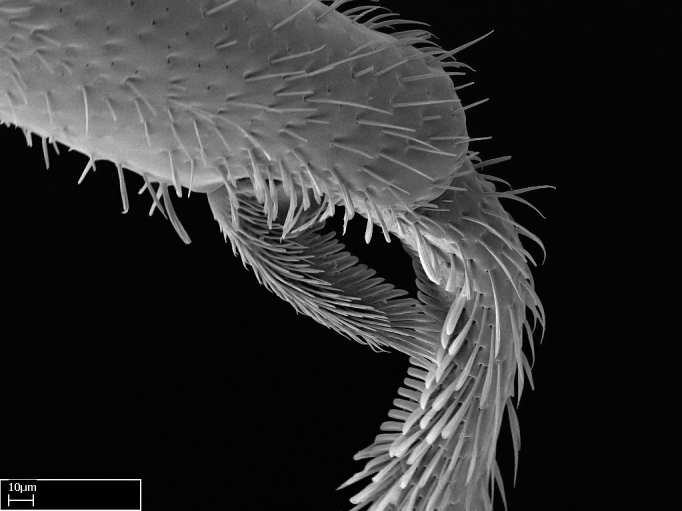
Anterior face of foreleg; close up of apex of protibia, including the calcar of strigil, and basal portion of probasitarsus.

**Figure 31b.**
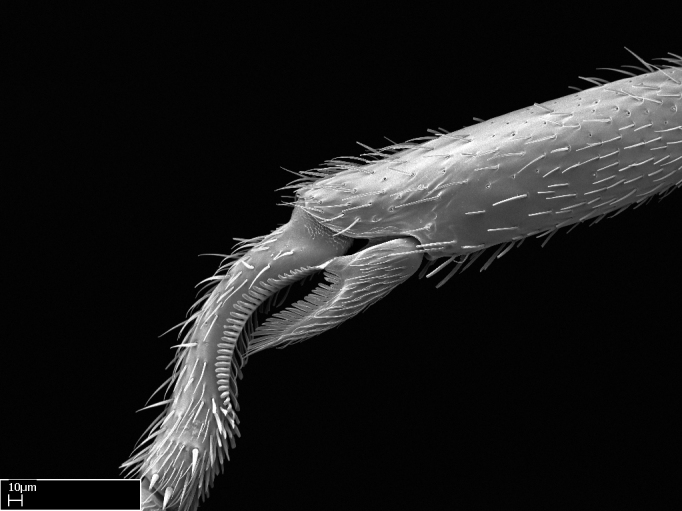
Posterior face of foreleg; close up of protibia, including the calcar of strigil, and probasitarsus.

**Figure 31c.**
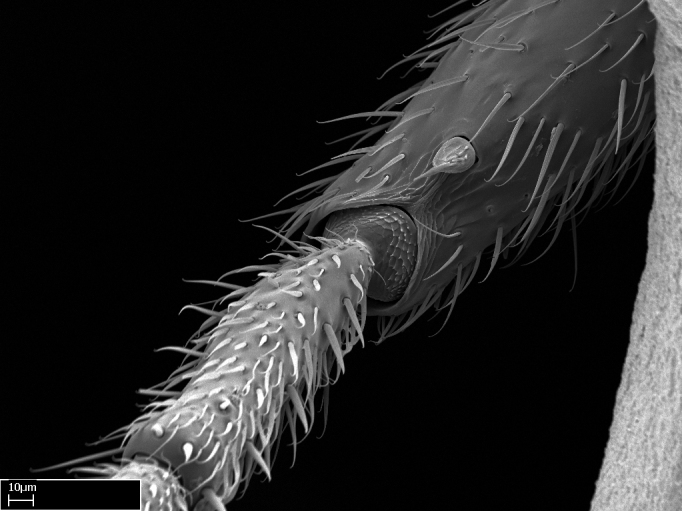
Inner face of midleg; close up of apex of mesotibia, including the mesotibial spur, and mesobasitarsus.

**Figure 31d.**
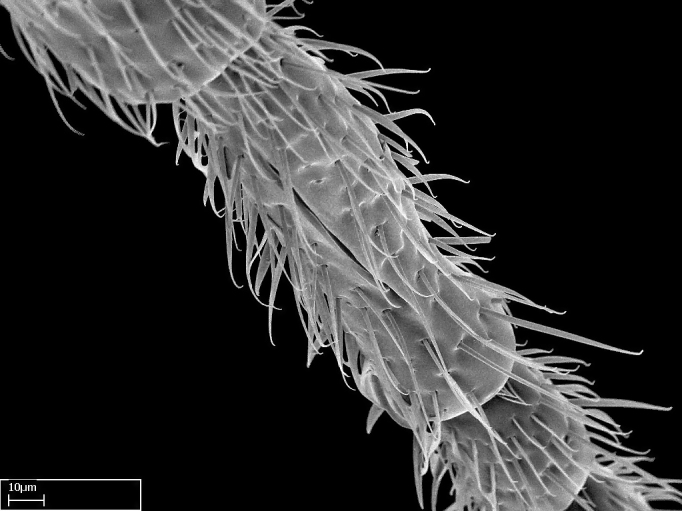
Dorsoanterior face of mesobasitarsus, including its longitudinal slit-like sulcus.

**Figure 31e.**
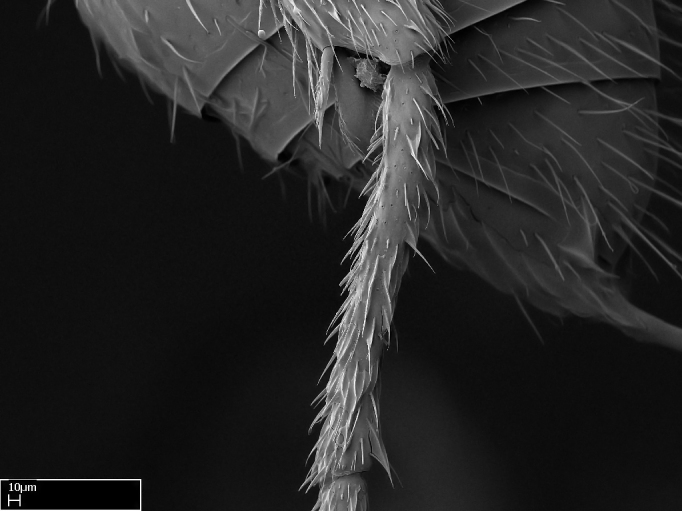
Anterior face of hindleg; close up of apex of metatibia, including metatibial spurs, and metabasitarsus.

**Figure 32a.**
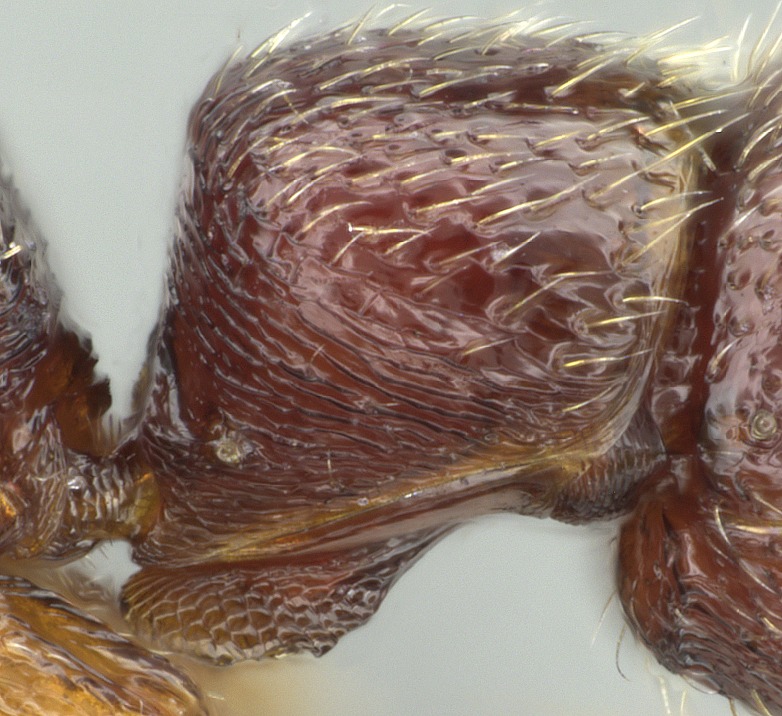
Petiole, lateral view (CASENT0034580).

**Figure 32b.**
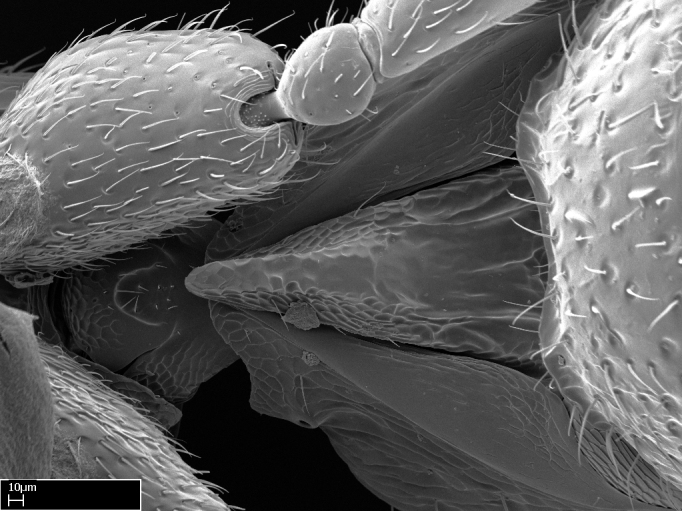
Petiole, ventral view (CASENT0034744).

**Figure 32c.**
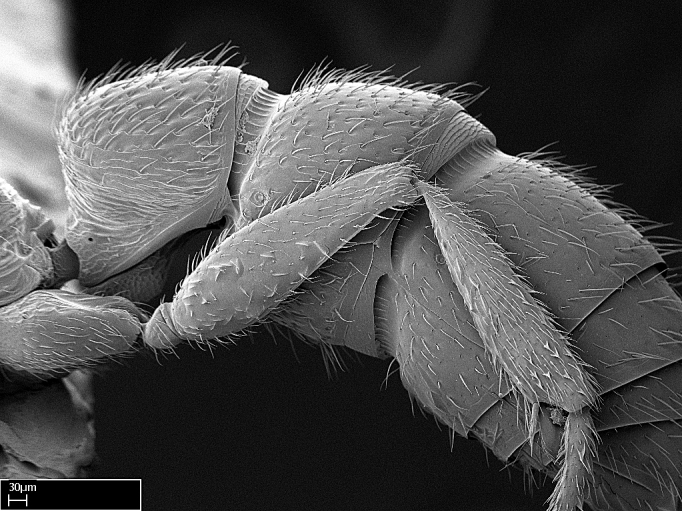
Petiole and gaster, lateral view (CASENT0034744).

**Figure 32d.**
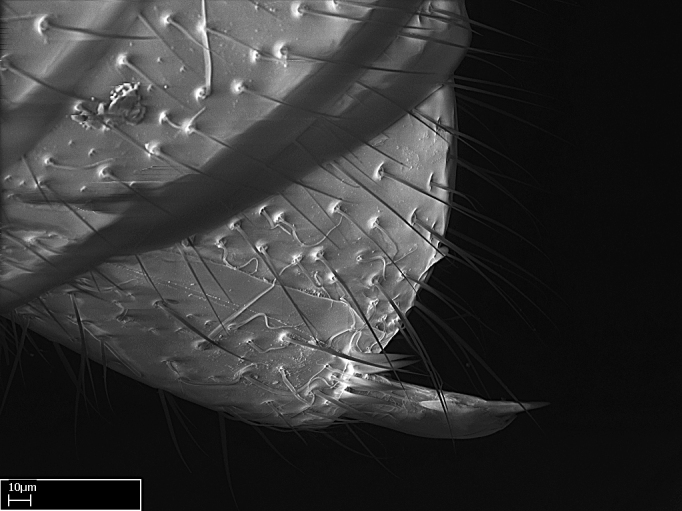
Close up of the gastral apical region, lateral view (CASENT0034580). Note stout spiniform setae on the apex of hypopygium, surrounding the stinger.

**Figure 33a.**
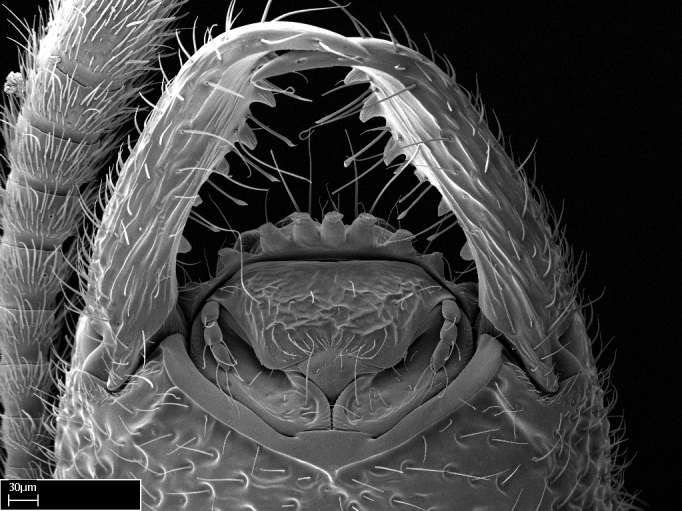
Ventral view of mandibles and mouthparts.

**Figure 33b.**
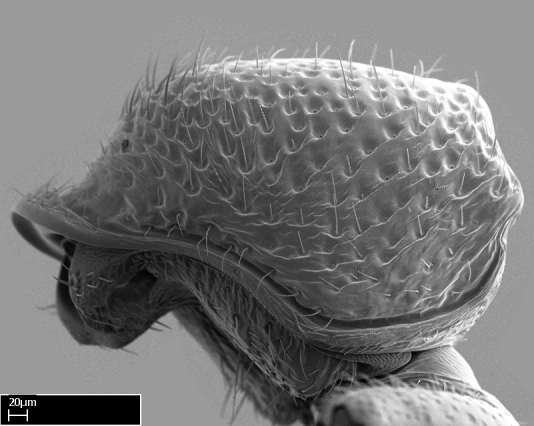
Lateral view of pronotum, which is disarticulated from head and remainder mesosoma.

**Figure 33c.**
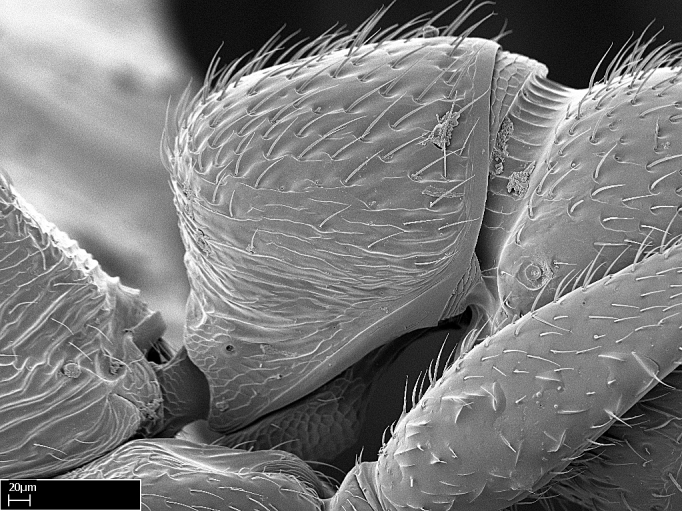
Lateral view of petiole.

**Figure 35a.**
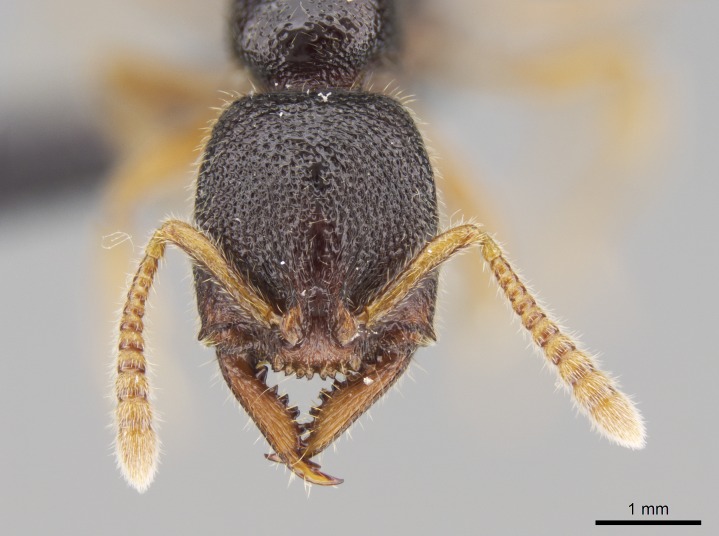
Fullface view.

**Figure 35b.**
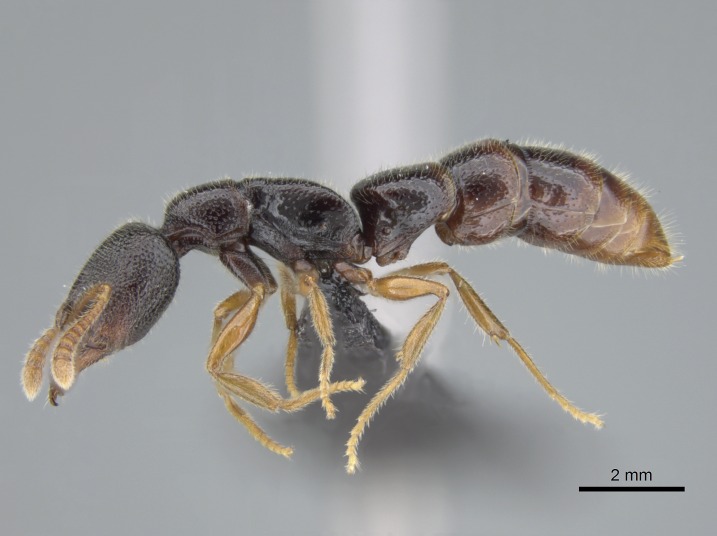
Lateral view.

**Figure 35c.**
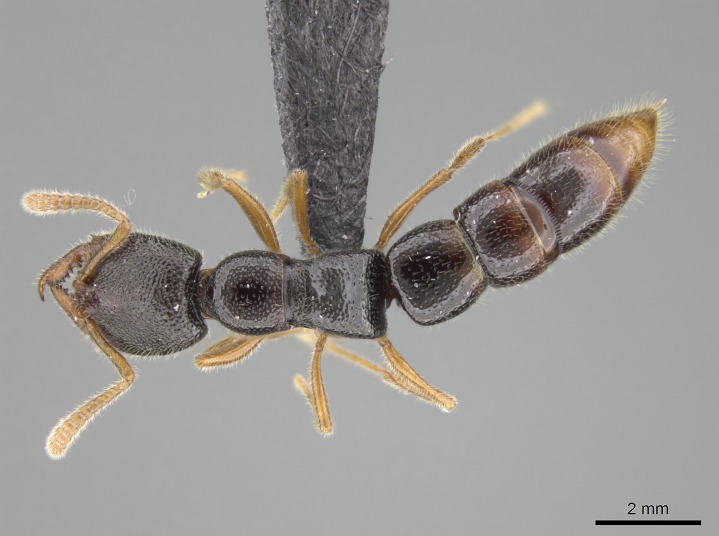
Dorsal view.

**Figure 36a.**
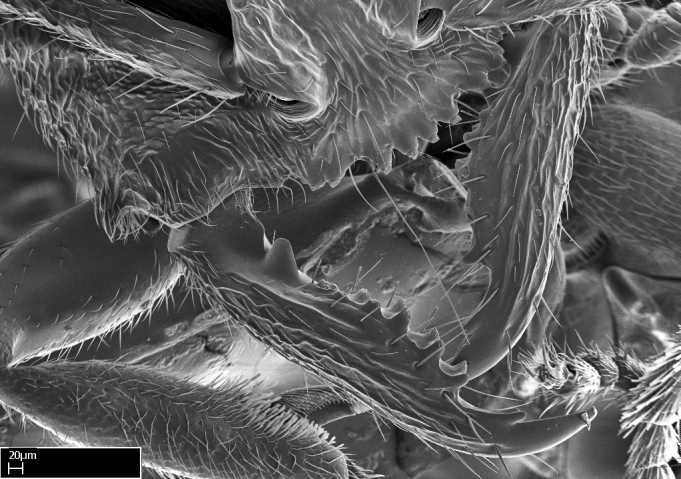
Dorsal view of the mandibles and anterior part of the head (CASENT0458591).

**Figure 36b.**
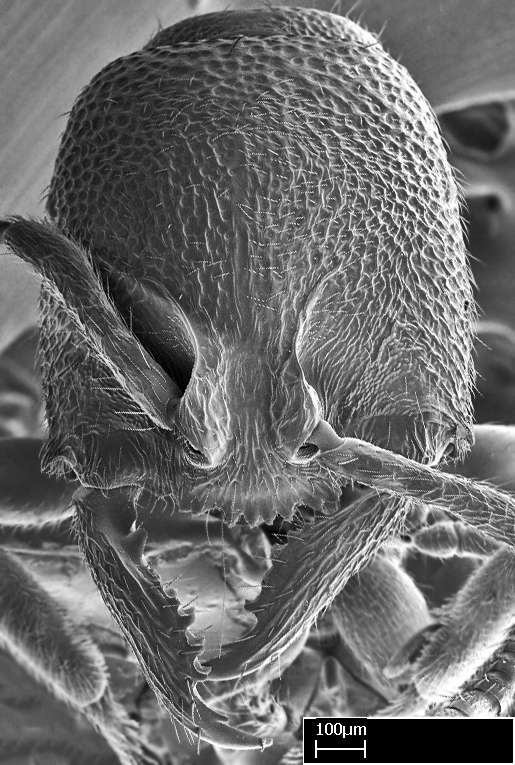
Fullface view (CASENT0458591).

**Figure 36c.**
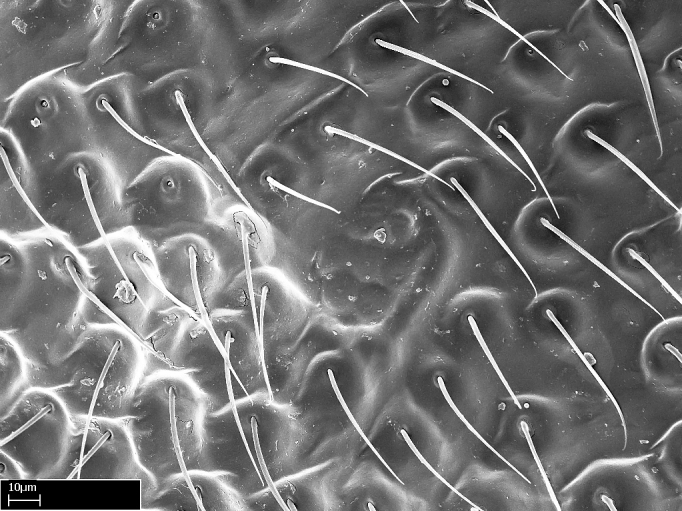
Close-up of the eyes, dorsolateral view (CASENT0458591).

**Figure 36d.**
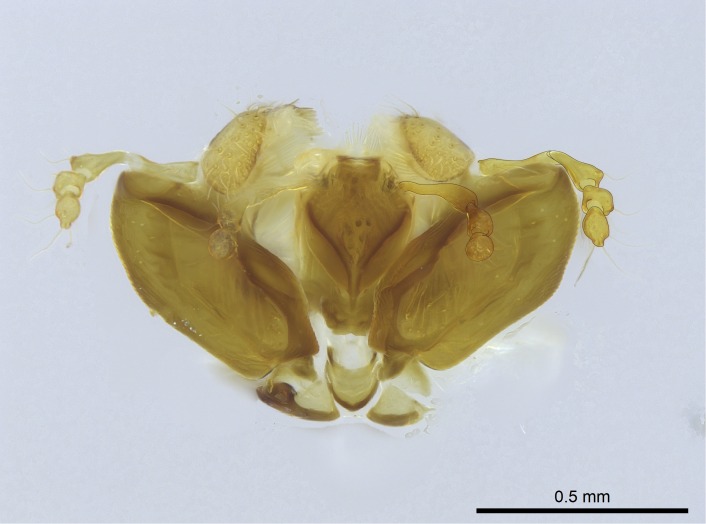
Mouth parts, ventral view (CASENT0458588). Left maxillary and labial palps are outlined in black and darkened to enhance visibility. Slide by F. A. Esteves.

**Figure 37a.**
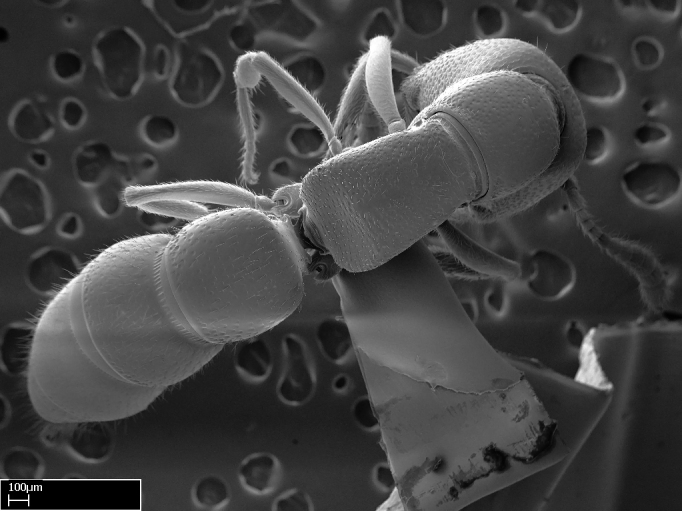
Dorsal view.

**Figure 37b.**
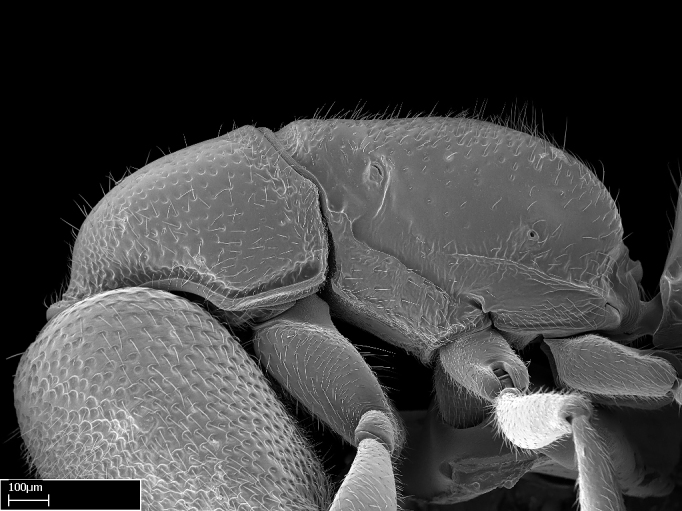
Lateral view

**Figure 38a.**
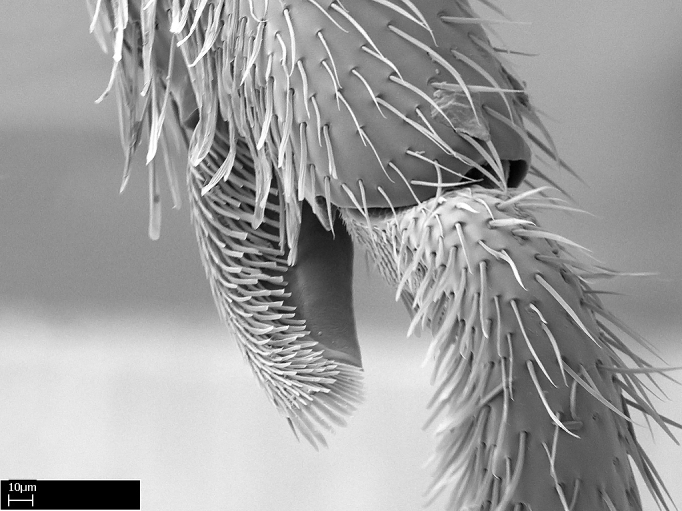
Foreleg, anterior face: close-up of apex of protibia, calcar of strigil, and base of probasitarsus.

**Figure 38b.**
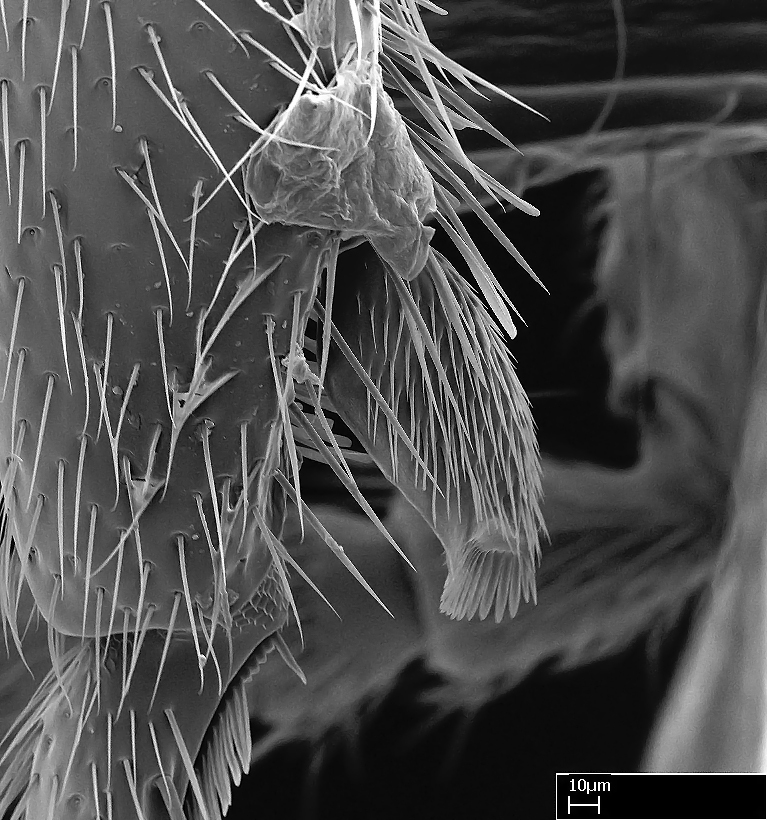
Foreleg, posterior face: close-up of apex of protibia, calcar of strigil, and base of probasitarsus.

**Figure 38c.**
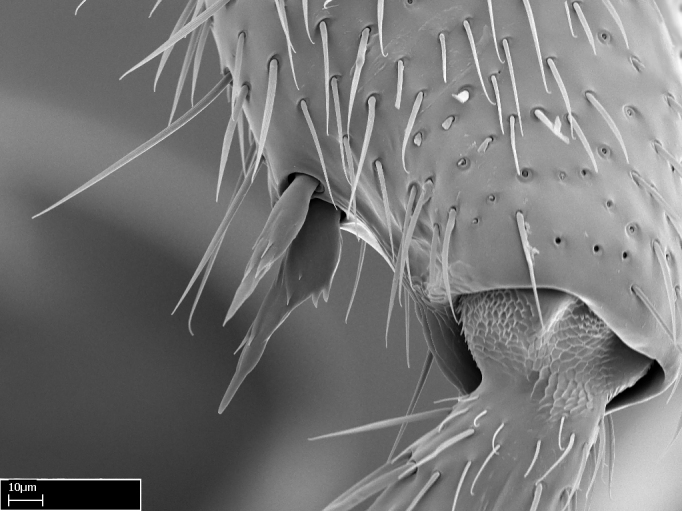
Midleg, anterior face: close-up of apex of mesotibia, mesotibial spurs, and base of mesobasitarsus.

**Figure 38d.**
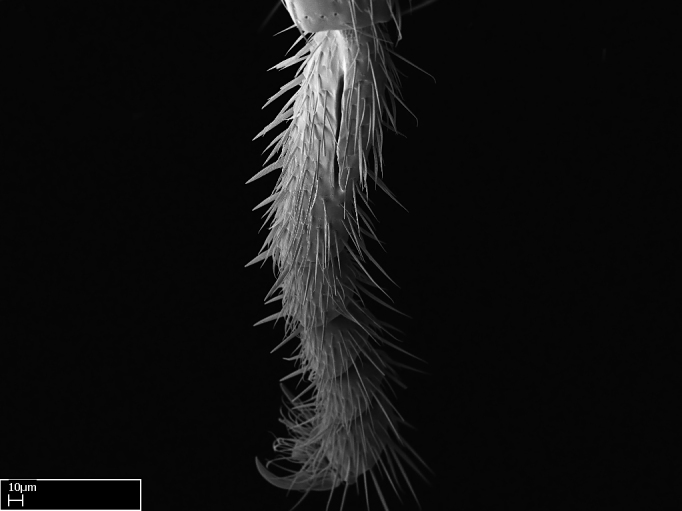
Midleg, anterodorsal face: close-up of mesobasitarsus bearing longitudinal slit-like sulcus, and tarsi.

**Figure 38e.**
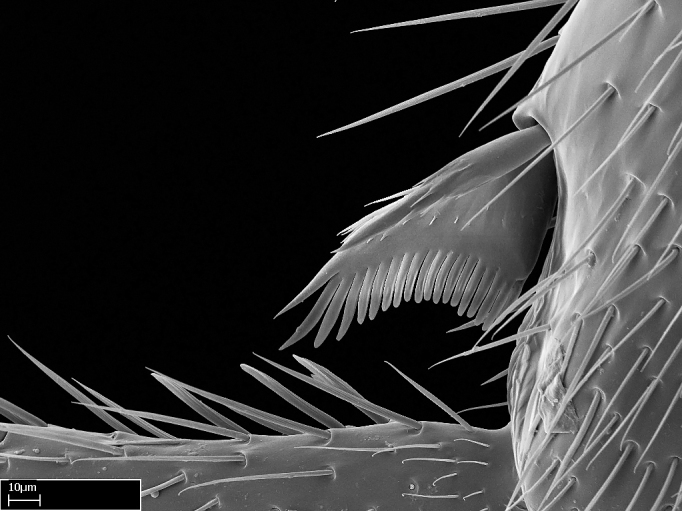
Hindleg, anterior face: close-up of apex of metatibia, metatibial spurs, and base of metabasitarsus.

**Figure 38f.**
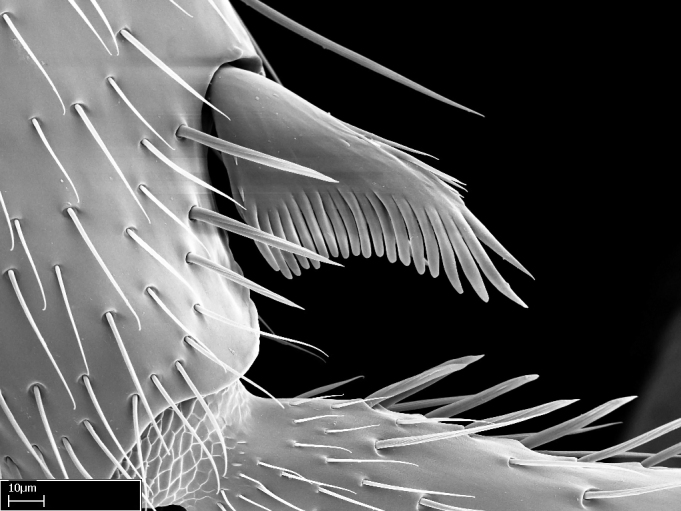
Hindleg, posterior face: close-up of apex of metatibia, metatibial spurs, and base of metabasitarsus.

**Figure 39a.**
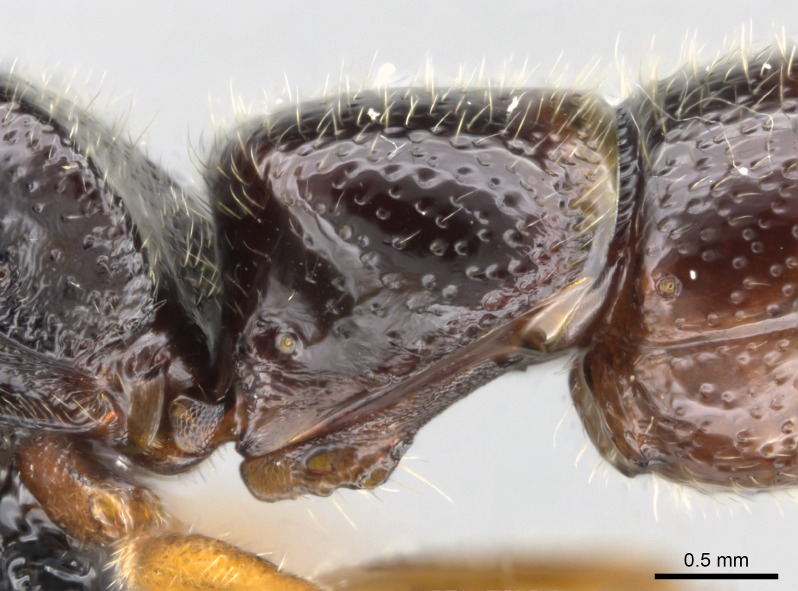
Petiole, lateral view (CASENT0042899).

**Figure 39b.**
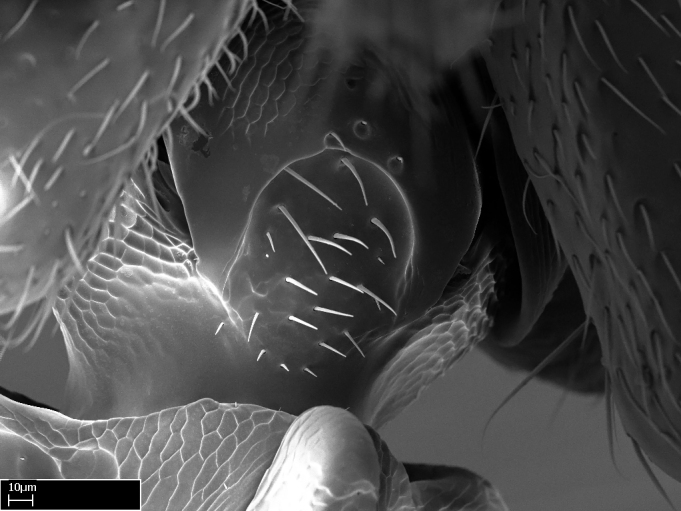
Petiole, ventral view: close-up of proprioceptor zone (CASENT0458591).

**Figure 39c.**
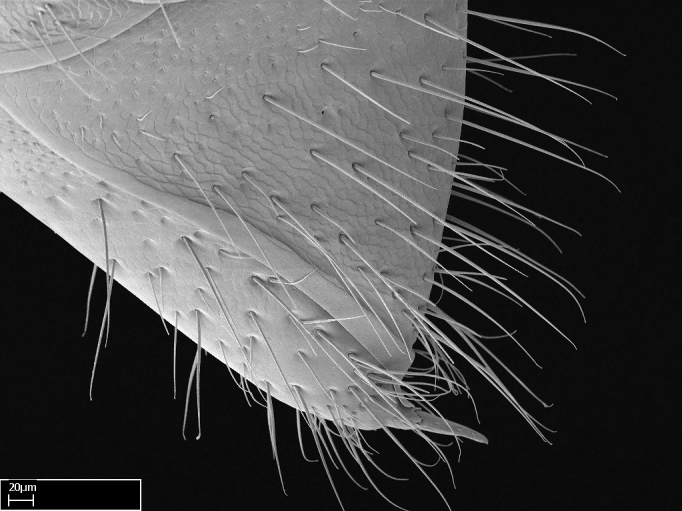
Gater, lateral view: close-up of abdominal segment VII (CASENT0458591).

**Figure 41a.**
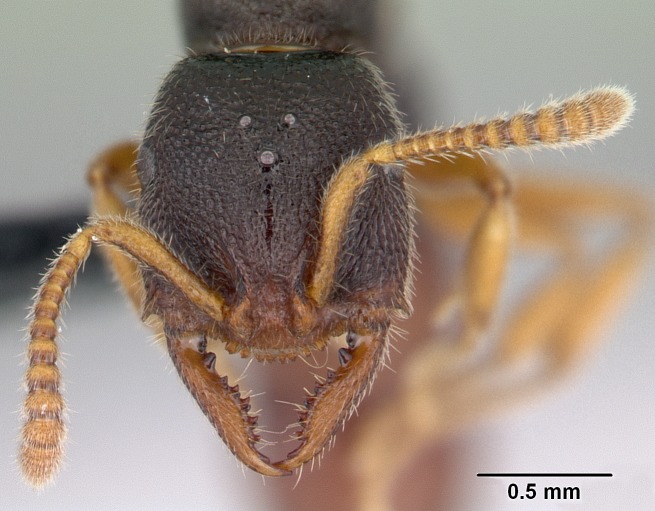
Fullface view.

**Figure 41b.**
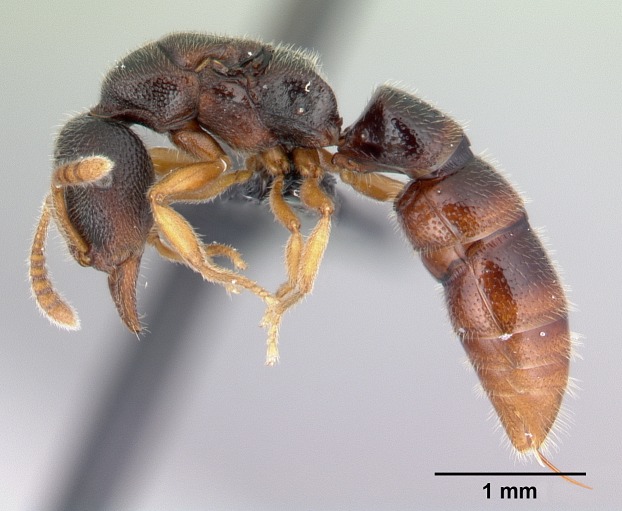
Lateral view.

**Figure 41c.**
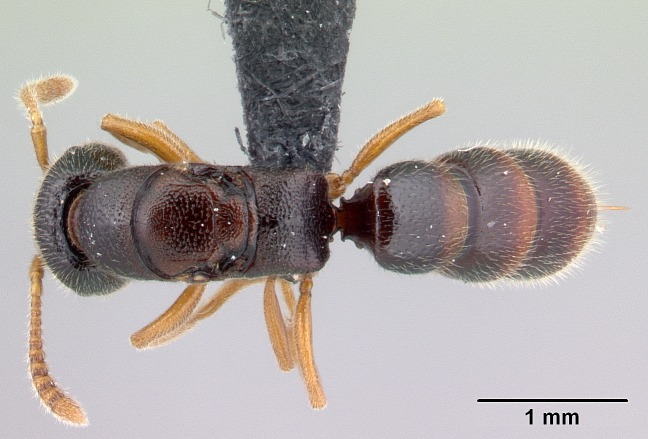
Dorsal view.

**Figure 42a.**
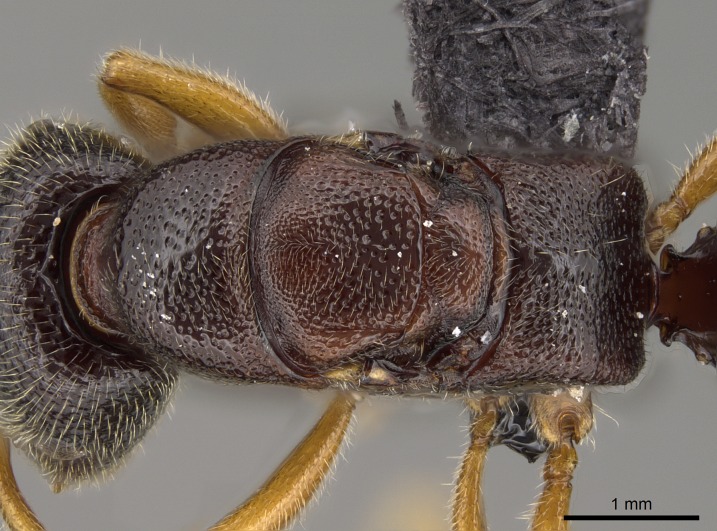
Mesosoma, dorsal view.

**Figure 42b.**
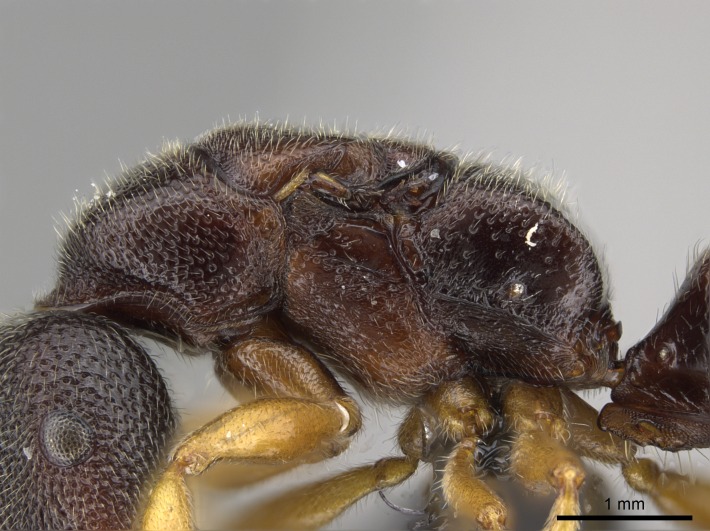
Mesosoma, lateral view.

**Figure 44a.**
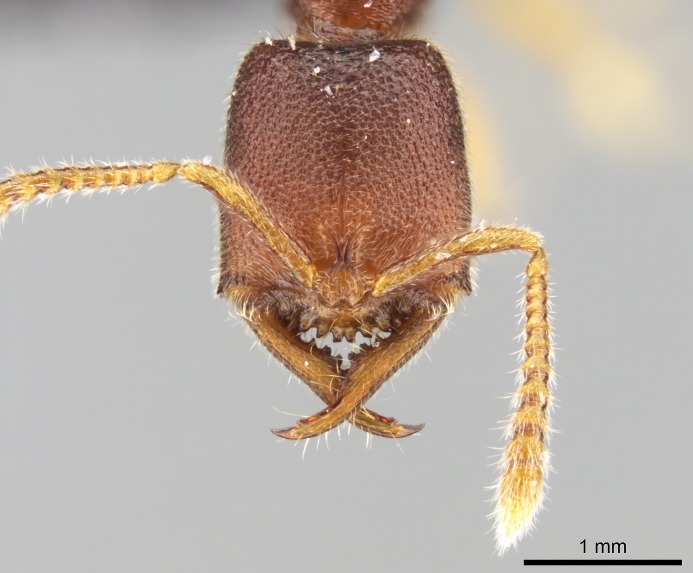
Fullface view.

**Figure 44b.**
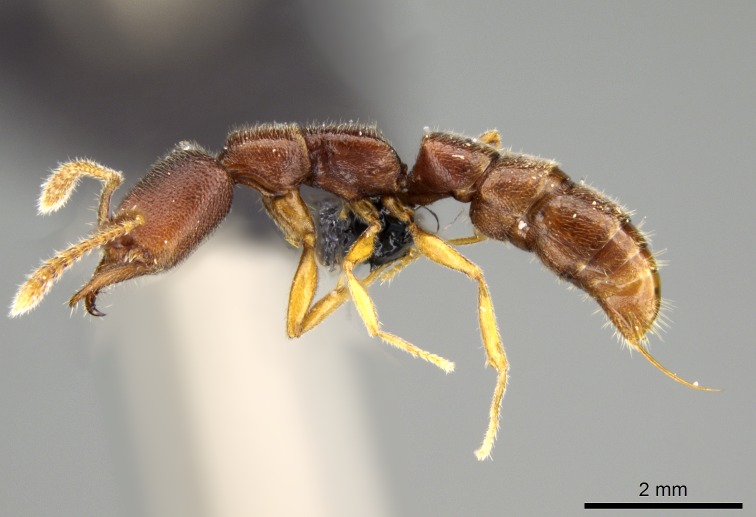
Lateral view.

**Figure 44c.**
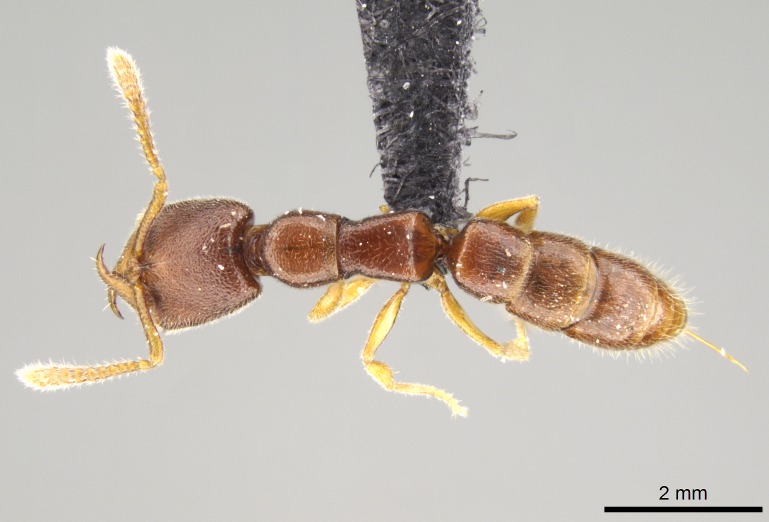
Dorsal view.

**Figure 45a.**
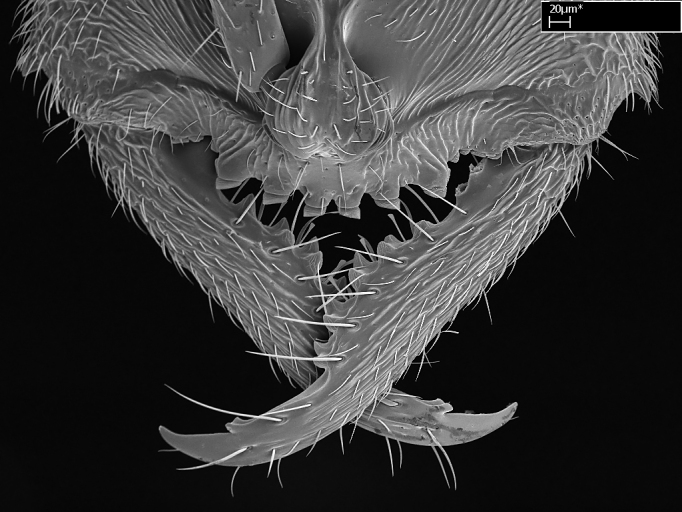
Dorsal view of the mandibles and anterior part of the head (CASENT0145426).

**Figure 45b.**
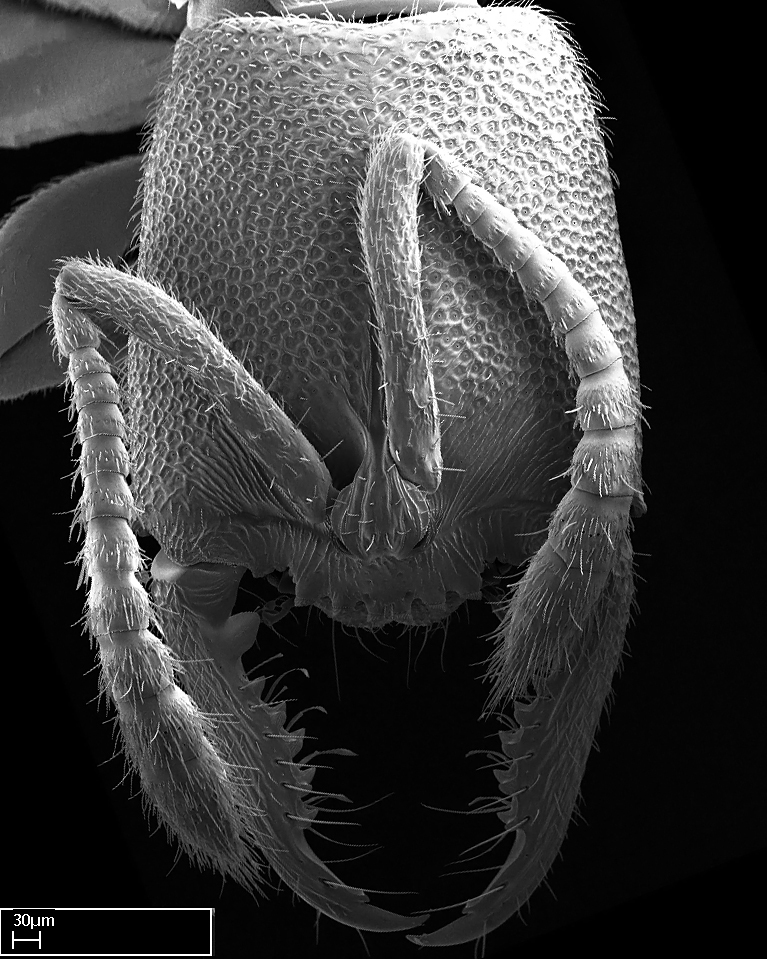
Fullface view (CASENT0318418).

**Figure 45c.**
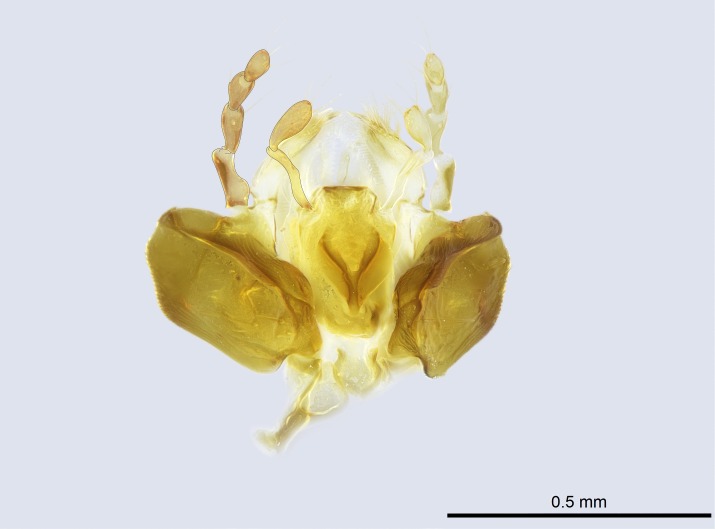
Mouthparts, ventral view (CASENT0159679). Left maxillary and labial palps are outlined in black and darkened to enhance visibility. Slide by F. A. Esteves.

**Figure 46a.**
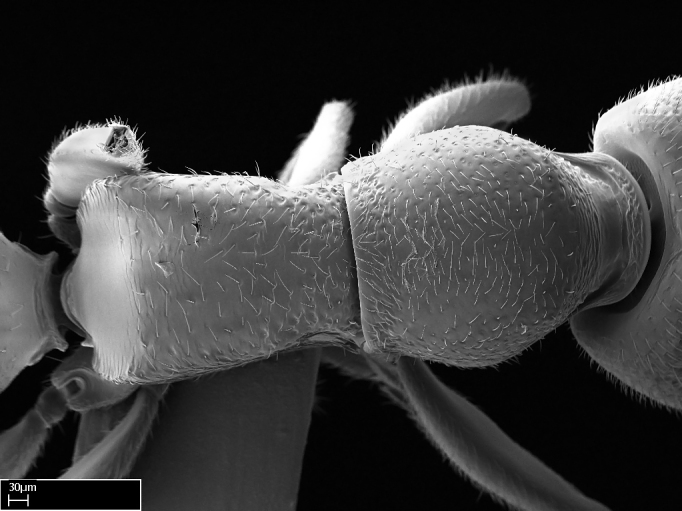
Dorsal view (CASENT0318418).

**Figure 46b.**
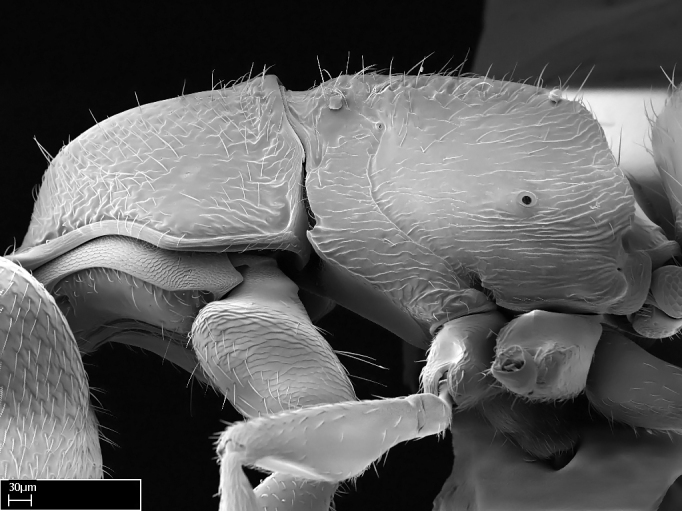
Lateral view (CASENT0145426).

**Figure 47a.**
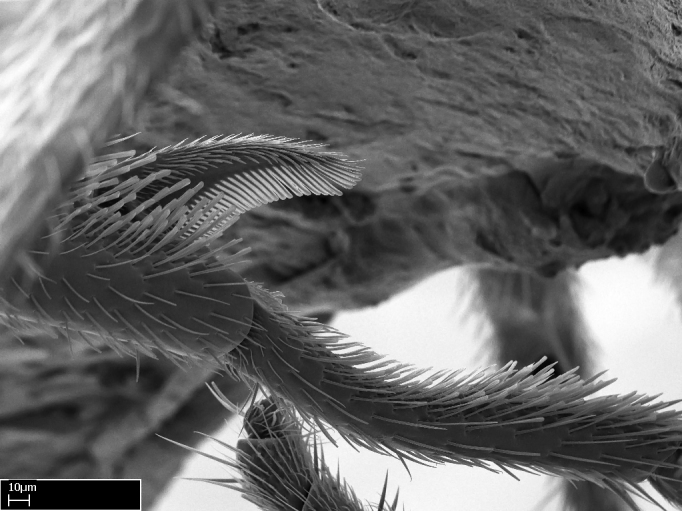
Foreleg, anterior face (CASENT0318418): close-up of the protibial apex, including the calcar of strigil and probasitarsus.

**Figure 47b.**
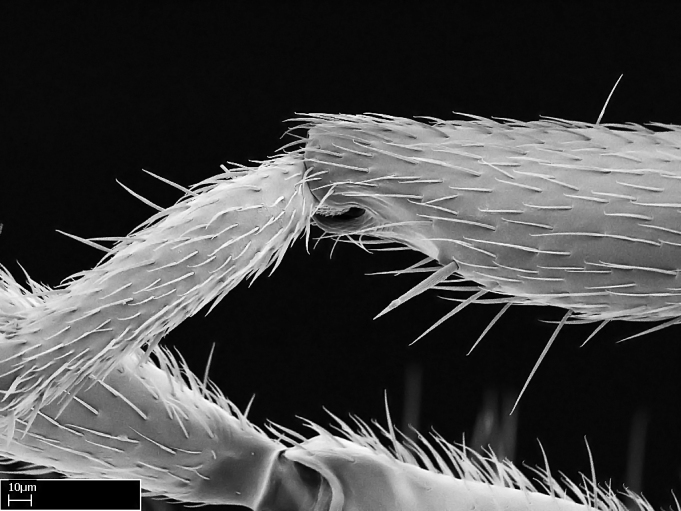
Midleg, posterior face (CASENT0145426): close-up of apical portion of mesotibia, including an enlarged, stout seta, and mesobasitarsus.

**Figure 47c.**
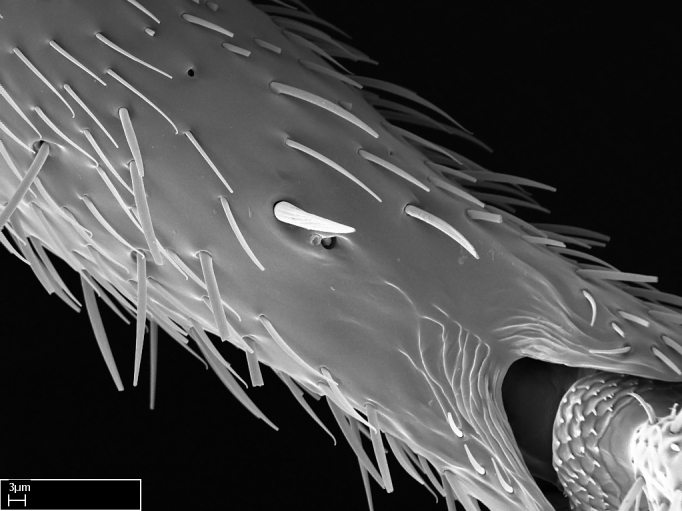
Midleg, inner face (CASENT0318418): close-up of the mesotibia apex, which includes an enlarged, stout seta, followed by a deep fovea concealing a small, stout, truncated seta.

**Figure 47d.**
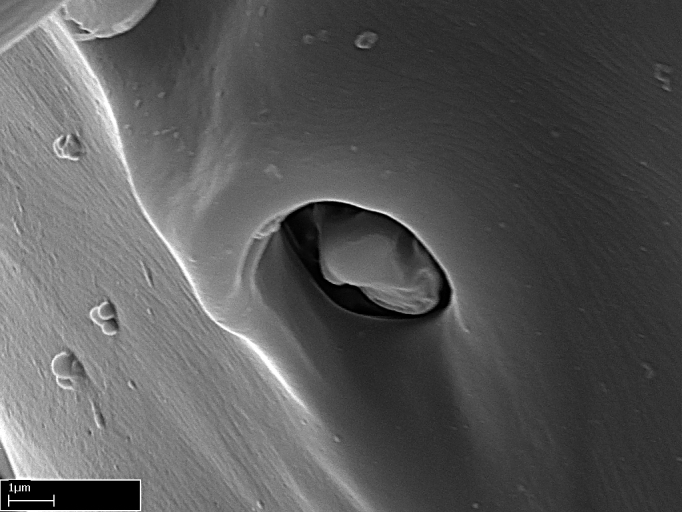
Midleg, inner face (CASENT0318418): close-up of the deep fovea concealing a small, stout, truncated seta (present at the mesotibia's apex).

**Figure 47e.**
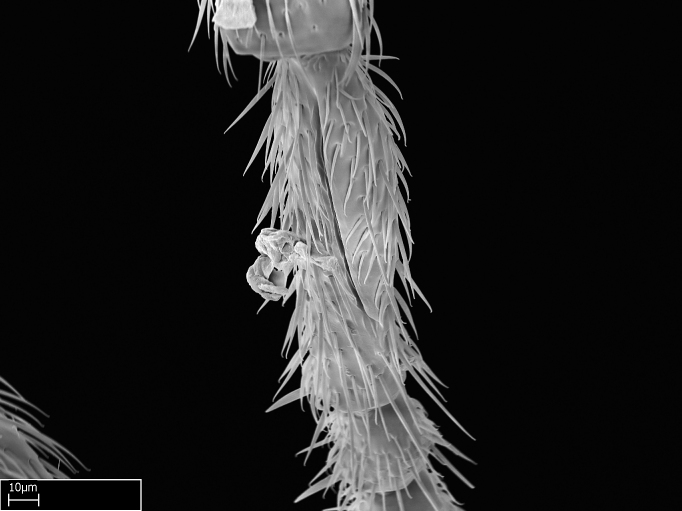
Midleg, anterior face (CASENT0145426): close up of the mesobasitarsus, including its slit-like longitudinal sulcus.

**Figure 48a.**
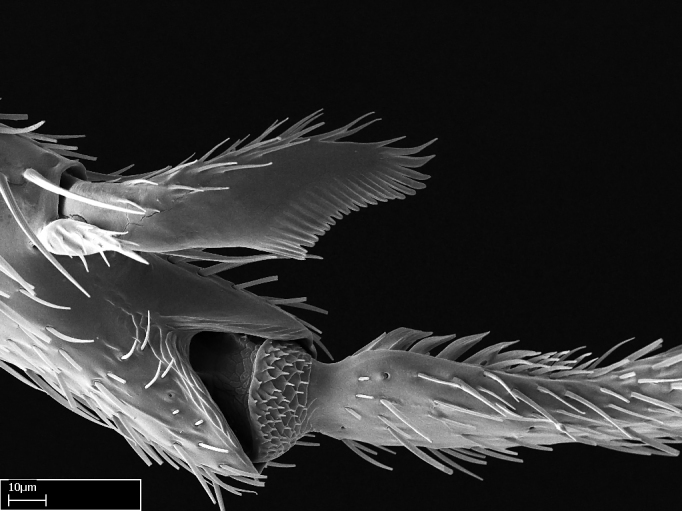
Hindleg: close-up of the inner face of the metatibial apex, which includes the metatibial spurs, and anterior face of metabasitarsus.

**Figure 48b.**
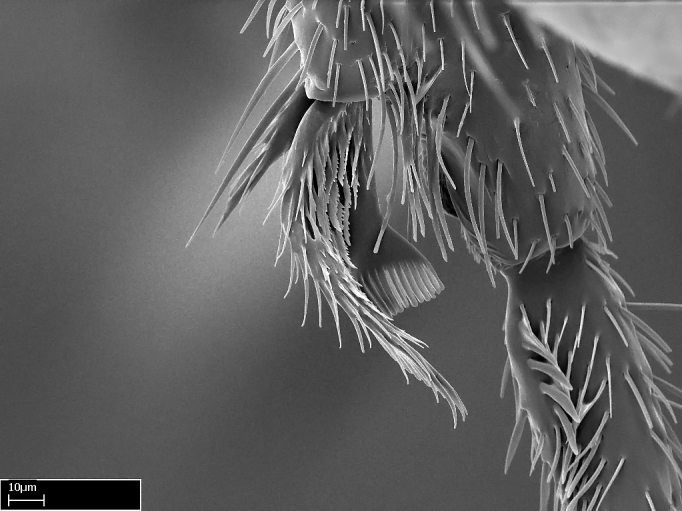
Hindleg, posterior face: close up of metatibial apex, which includes the metatibial spurs, and basal portion of metabasitarsus.

**Figure 48c.**
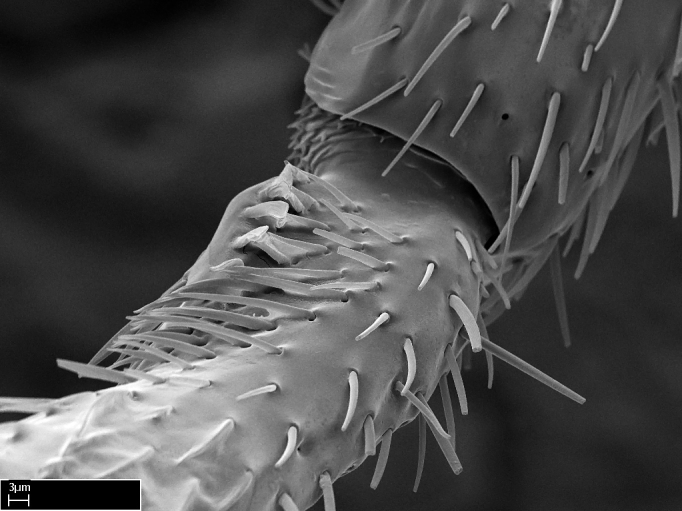
Hindleg: inner face of basal portion of metabasitarsus.

**Figure 49a.**
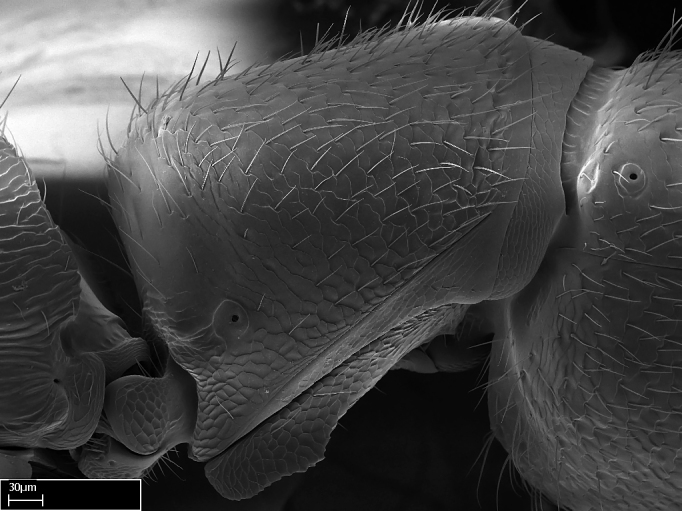
Petiole, lateral view.

**Figure 49b.**
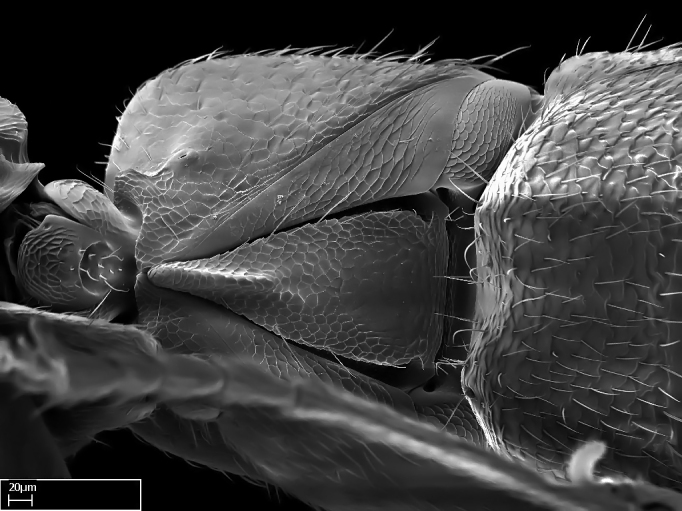
Petiole, ventral view.

**Figure 49c.**
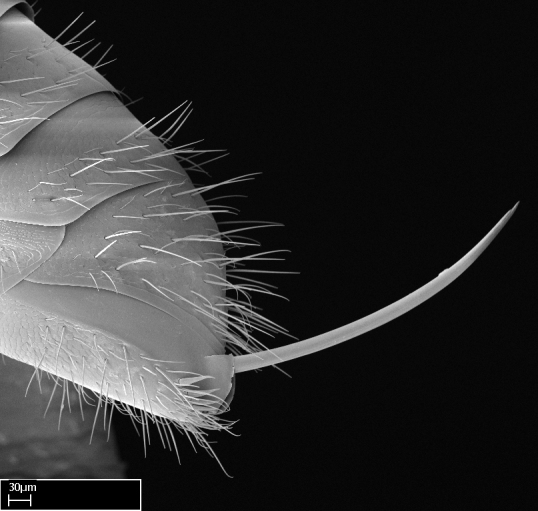
Apex of the gaster and stinger, lateral view. Note the stout spiniform setae on the apex of the hypopygium, surrounding the stinger.

**Figure 51a.**
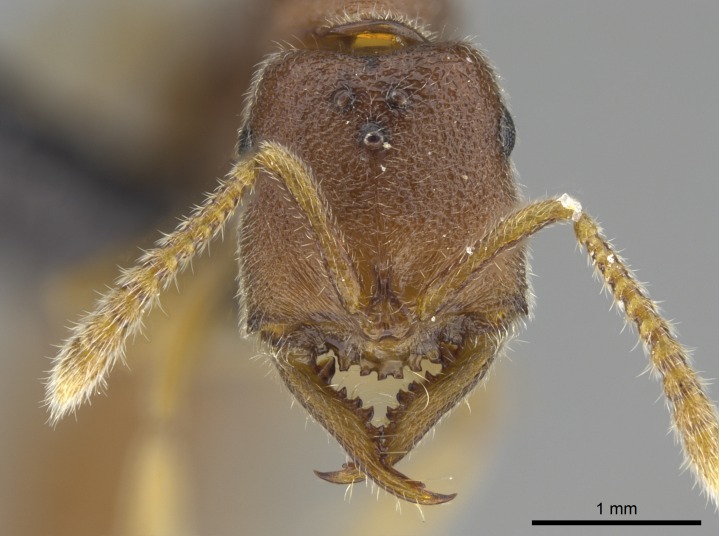
Fullface view.

**Figure 51b.**
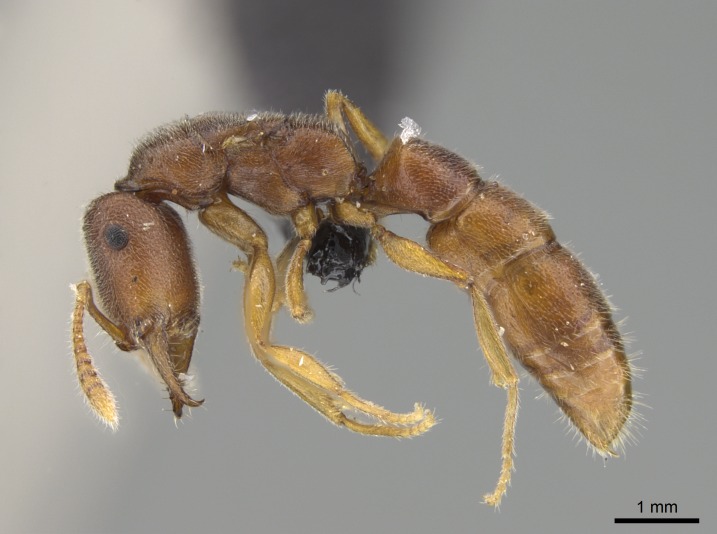
Lateral view.

**Figure 51c.**
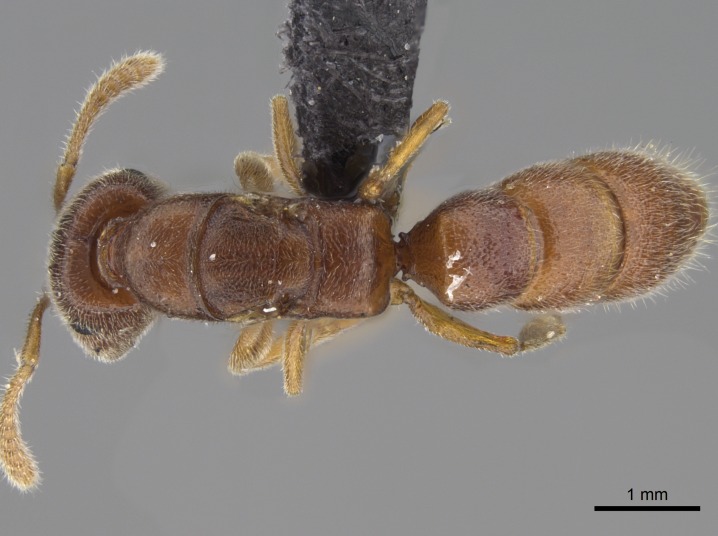
Dorsal view.

**Figure 52a.**
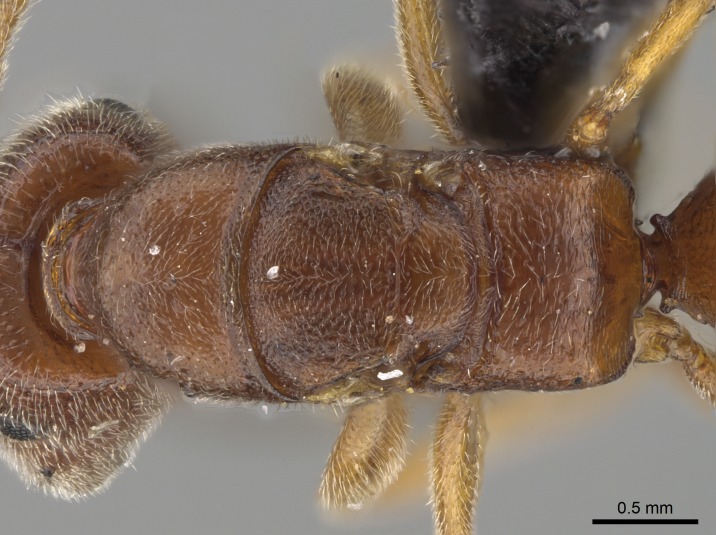
Mesosoma, dorsal view.

**Figure 52b.**
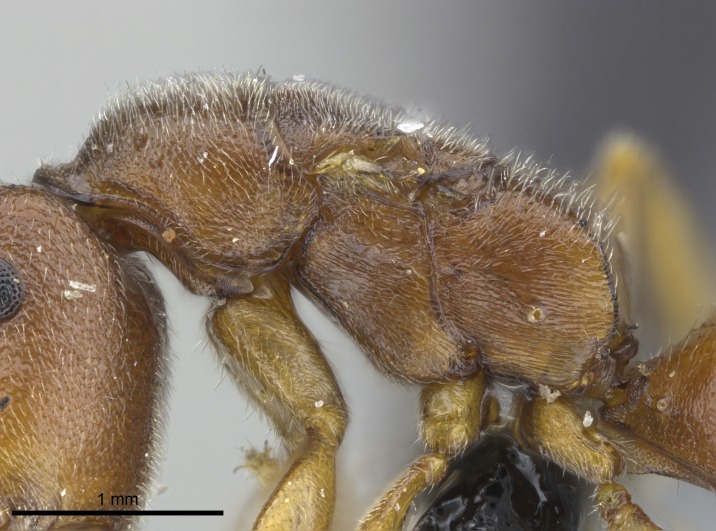
Mesosoma, lateral view.

**Figure 53a.**
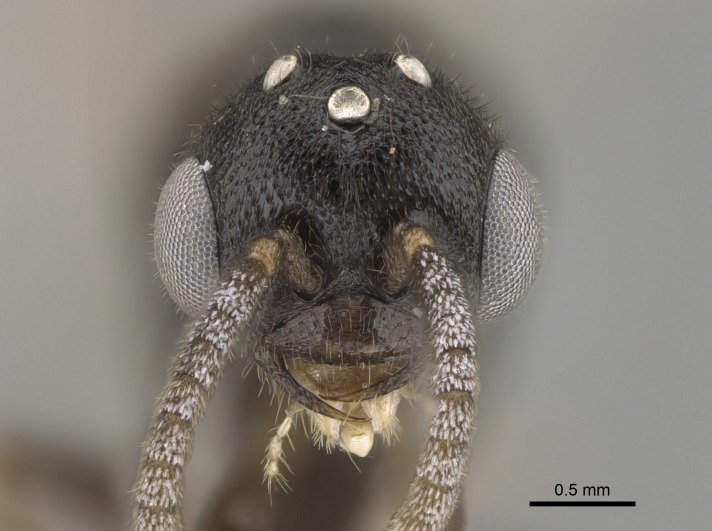
Fullface view.

**Figure 53b.**
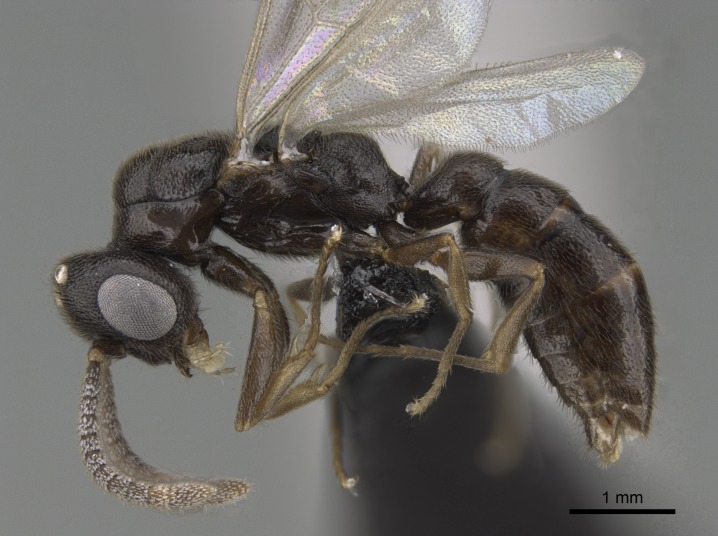
Lateral view.

**Figure 53c.**
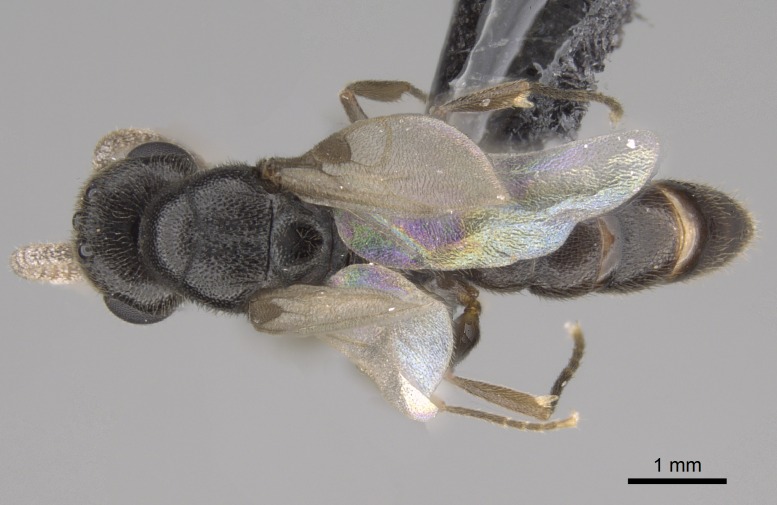
Dorsal view.

**Figure 53d.**
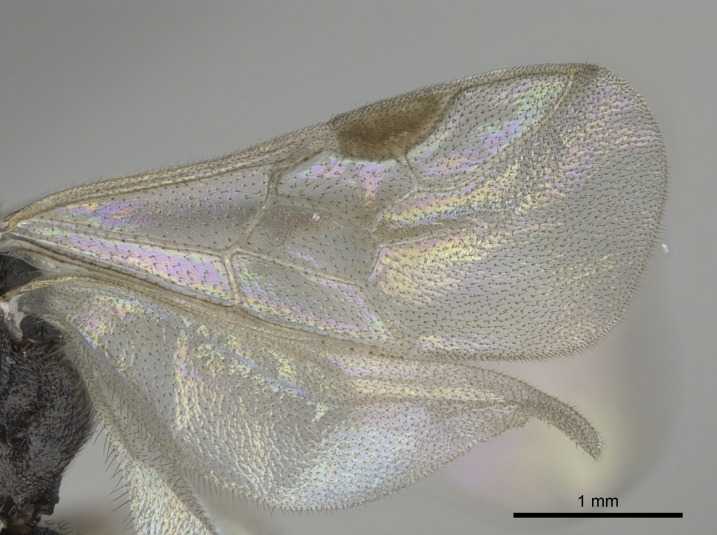
Left fore- and hindwing.

**Figure 54a.**
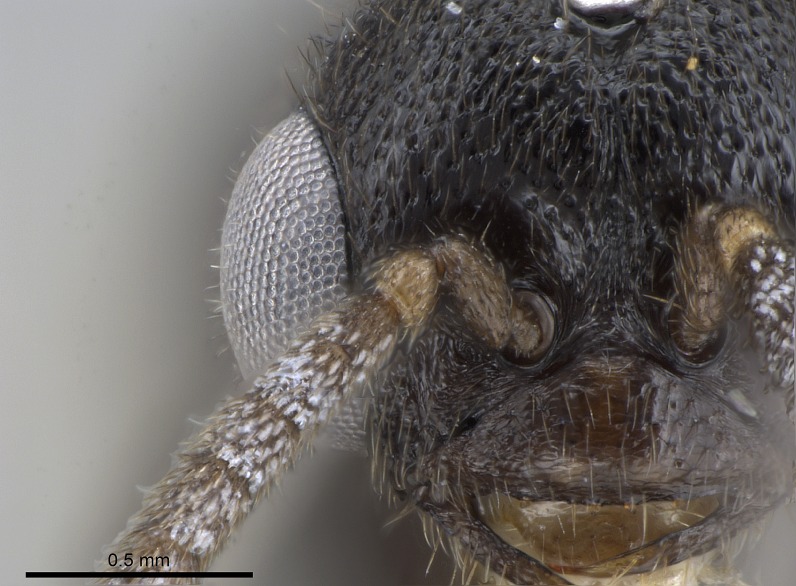
Right compound eye ,dorsal view.

**Figure 54b.**
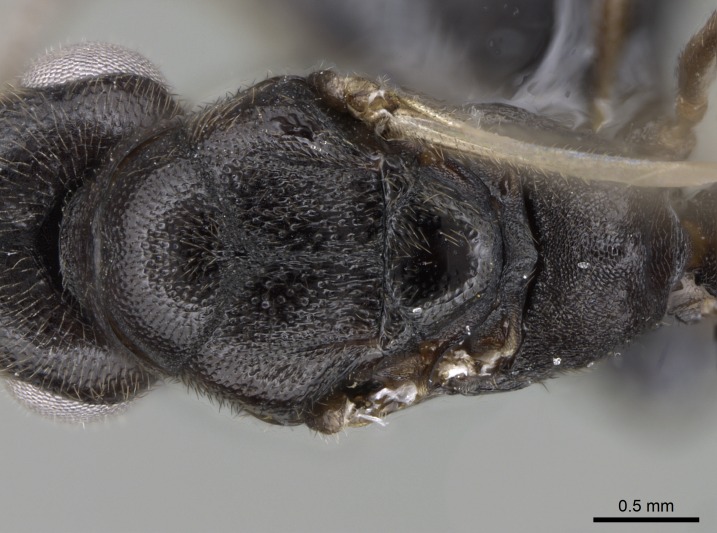
Mesosoma, dorsal view. Left wings were removed for better illustration.

**Figure 54c.**
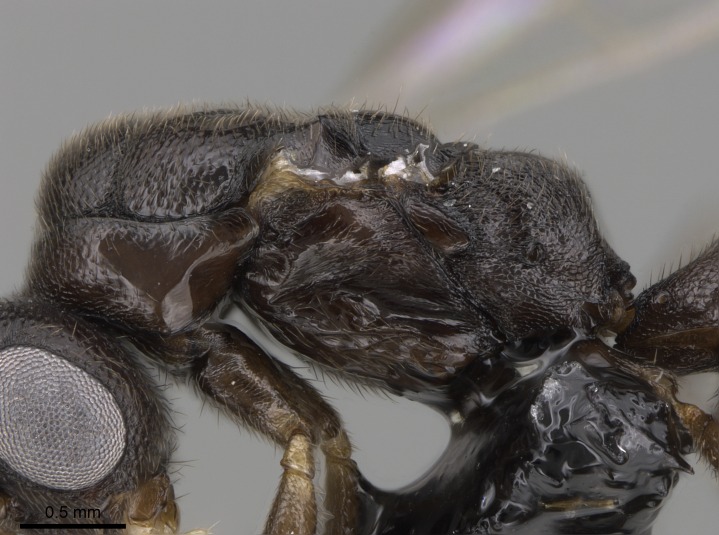
Mesosoma, lateral view. Left wings were removed for better illustration.

**Figure 54d.**
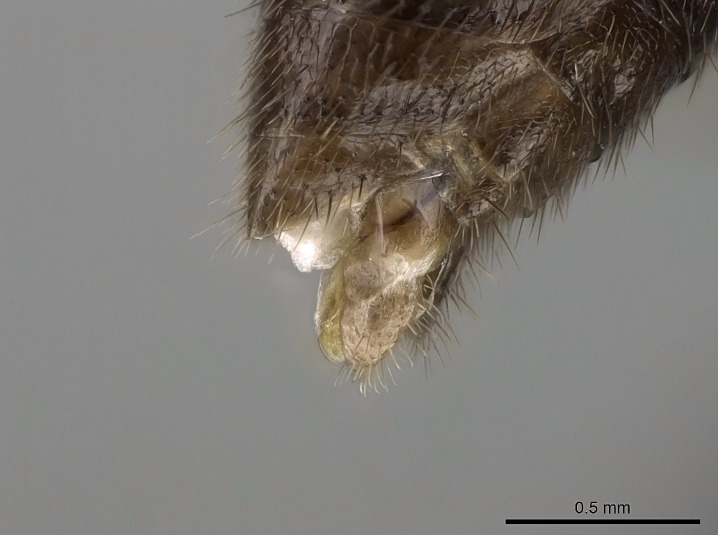
Apex of the gaster, lateral view.

**Figure 55a.**
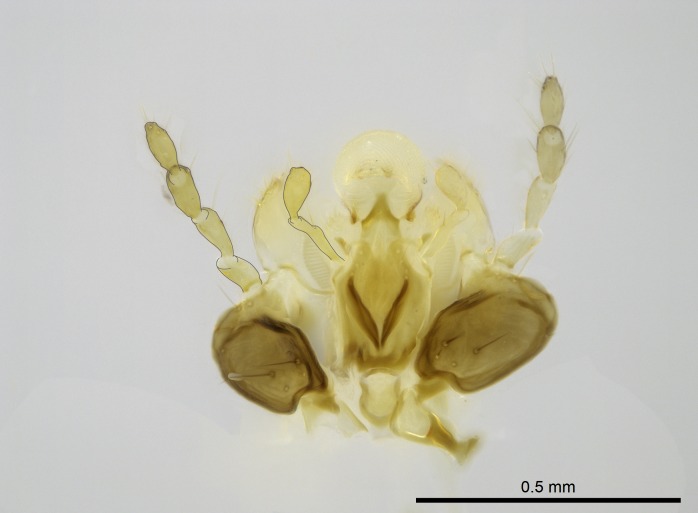
Mouthparts, ventral view. Right maxillary and labial palps are outlined in black and darkened to enhance visibility. Slide by F. A. Esteves.

**Figure 55b.**
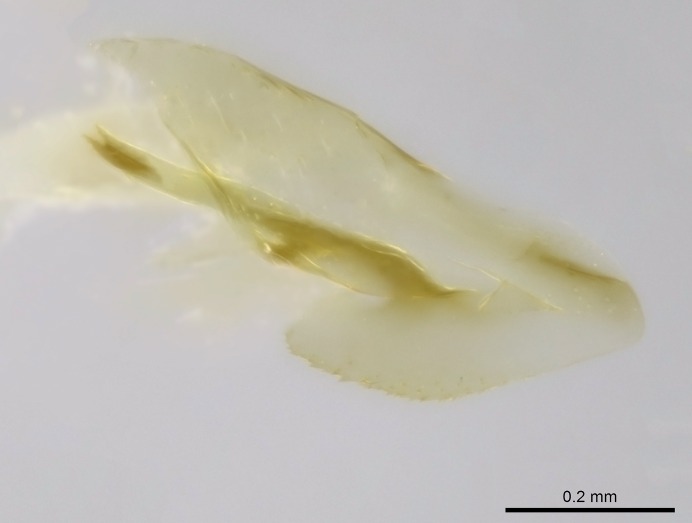
Aedeagus, lateral view. Slide by F. A. Esteves.

**Figure 55c.**
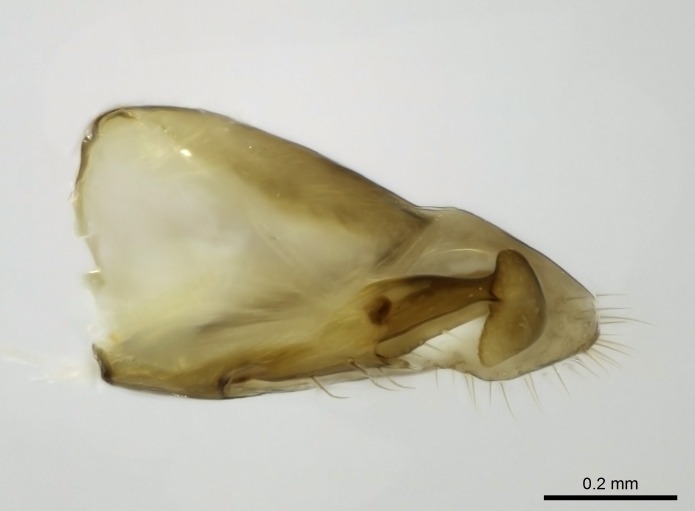
Longitudinal section of the genital capsule; inner face, lateral view. The basal ring was removed from the specimen. Slide by F. A. Esteves.

**Figure 55d.**
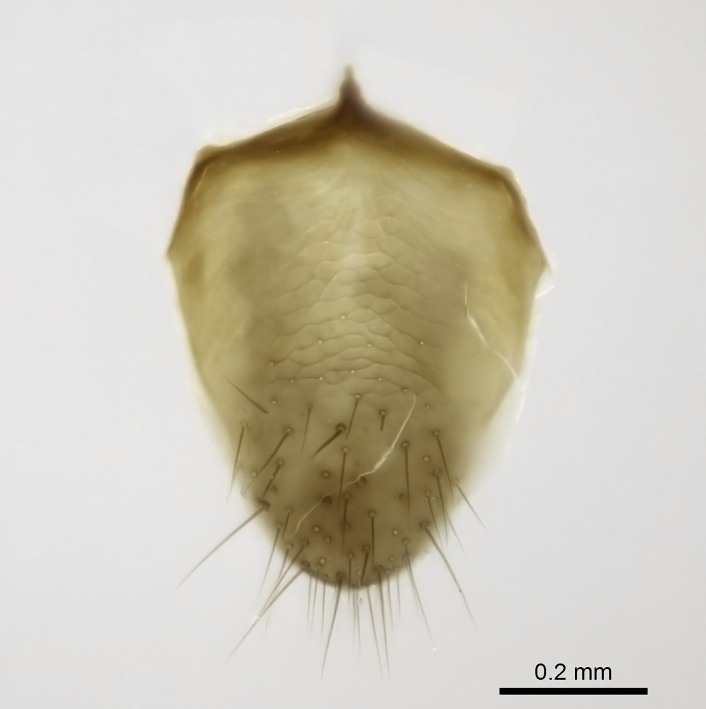
Abdominal sternun IX. Slide by F. A. Esteves.

**Figure 56a.**
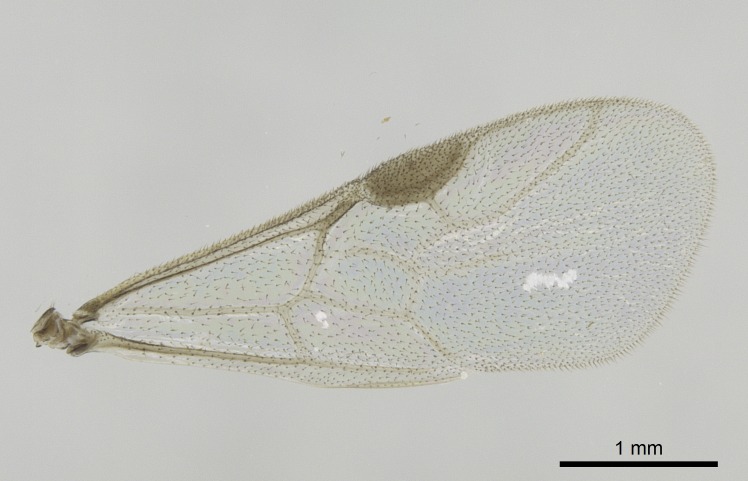
Forewing. Slide by F. A. Esteves.

**Figure 56b.**
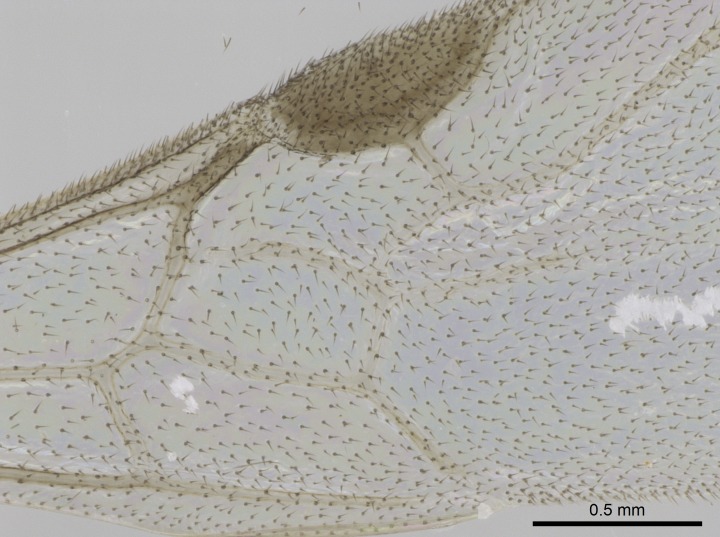
Close up of the venation of the forewing. Slide by F. A. Esteves.

**Figure 56c.**
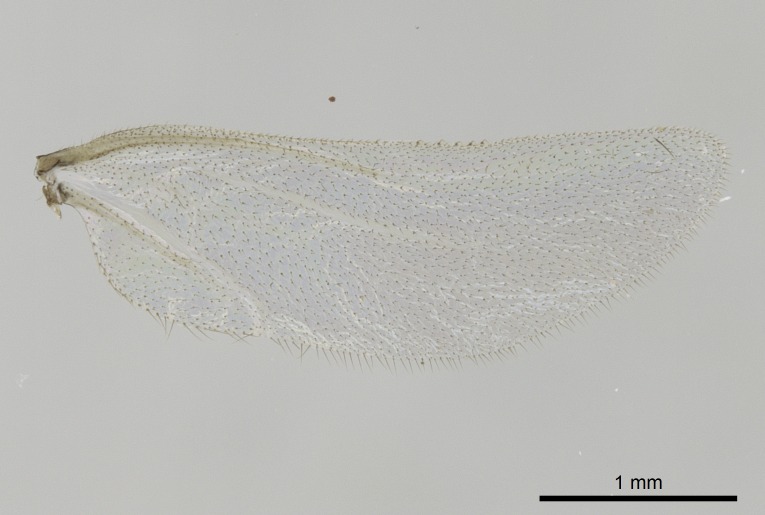
Hindwing. Slide by F. A. Esteves.

**Figure 56d.**
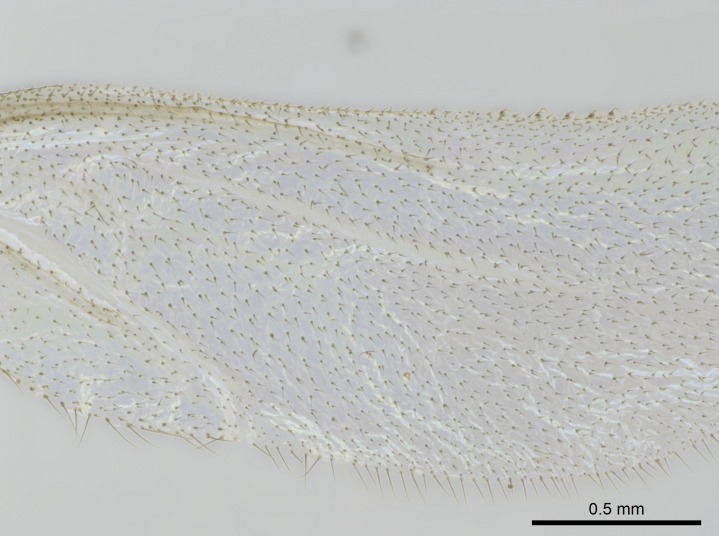
Close-up of the venation of the hindwing. Slide by F. A. Esteves.

**Figure 57a.**
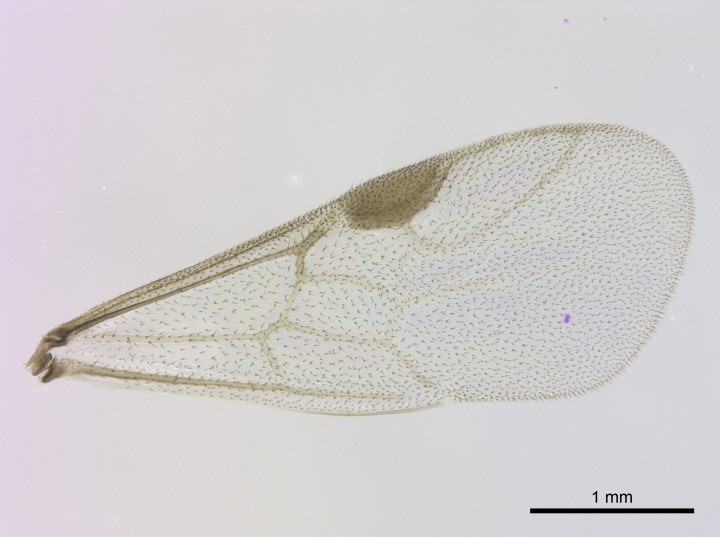
Forewing. Slide by F. A. Esteves.

**Figure 57b.**
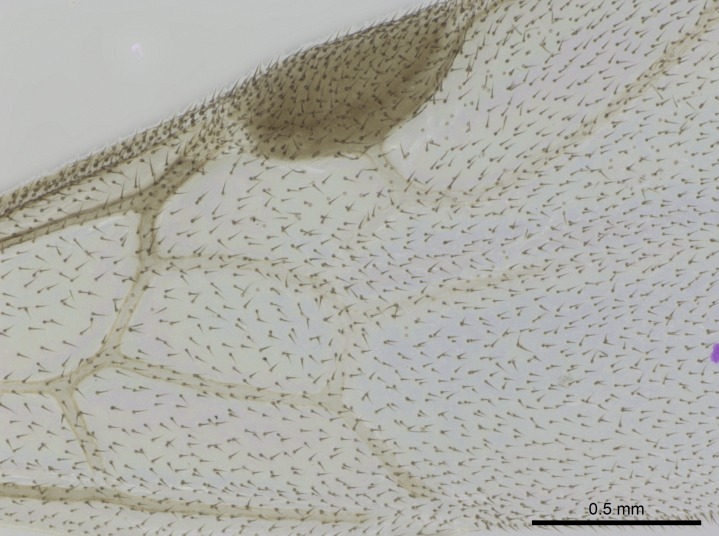
Close-up of the venation of the forewing. Slide by F. A. Esteves.

**Figure 57c.**
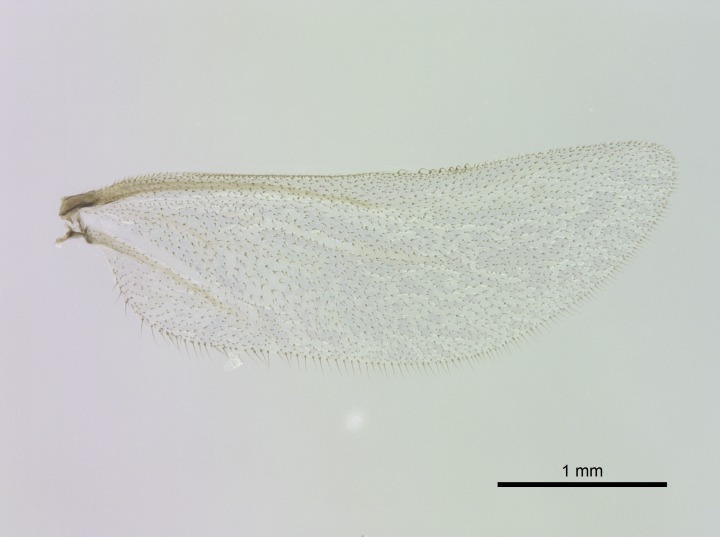
Hindwing. Slide by F. A. Esteves.

**Figure 57d.**
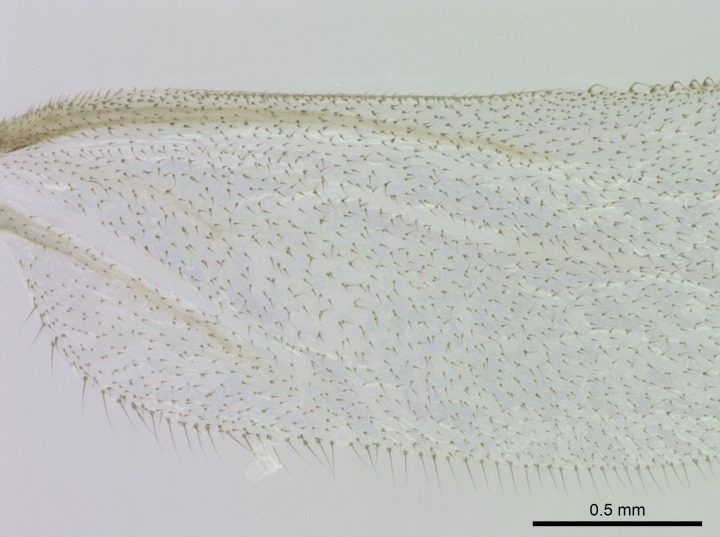
Close-up of the venation of the hindwing. Slide by F. A. Esteves.

**Figure 59a.**
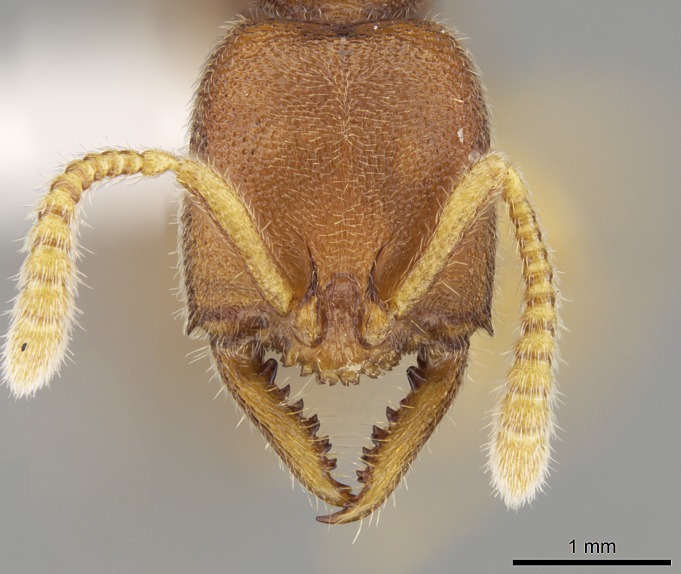
Fullface view.

**Figure 59b.**
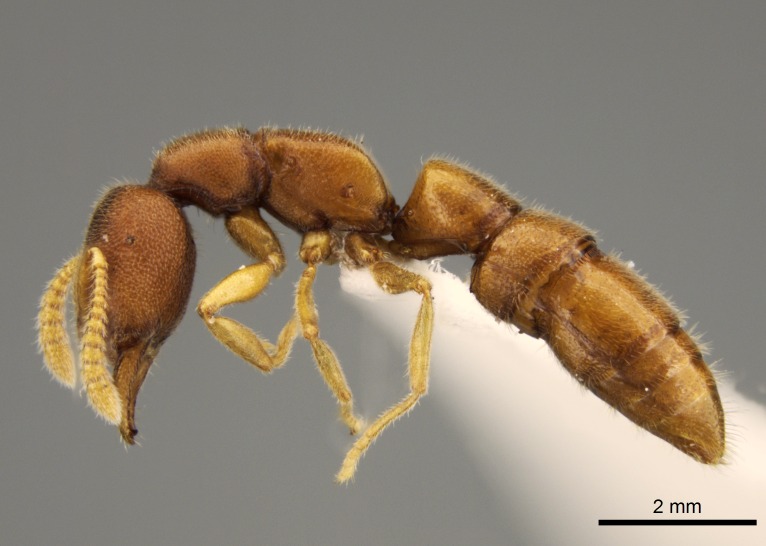
Lateral view.

**Figure 59c.**
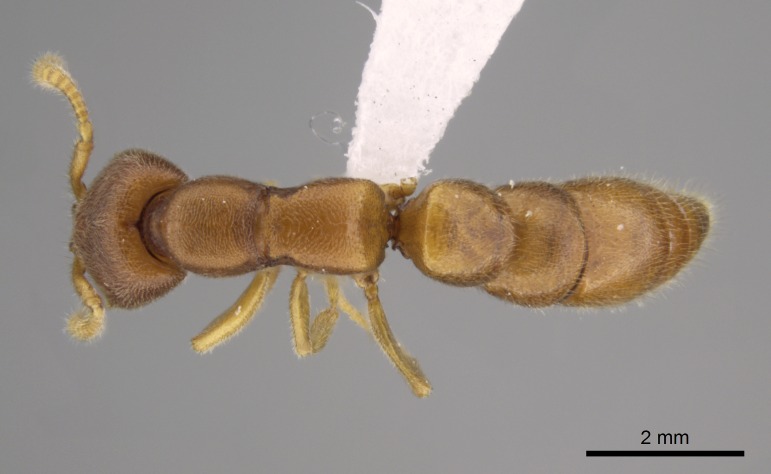
Dorsal view.

**Figure 60a.**
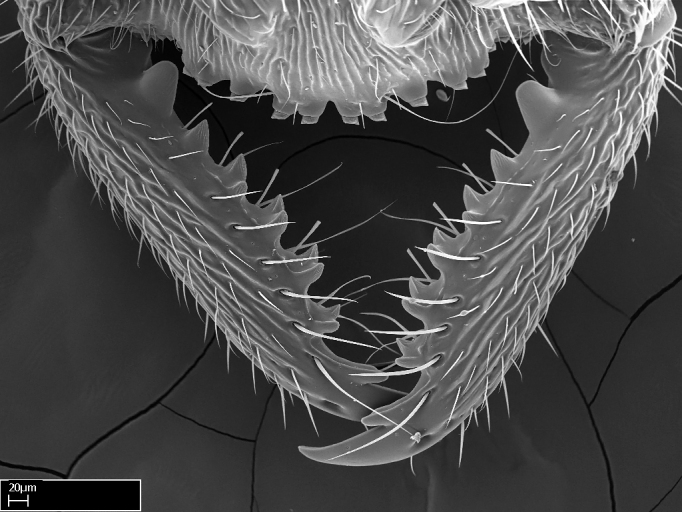
Dorsal view of the mandibles and anterior part of the head (CASENT0724172).

**Figure 60b.**
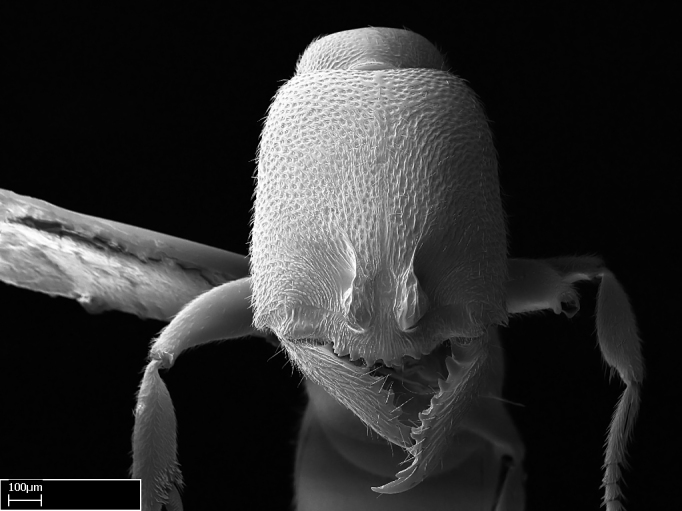
Fullface view (CASENT0009102).

**Figure 60c.**
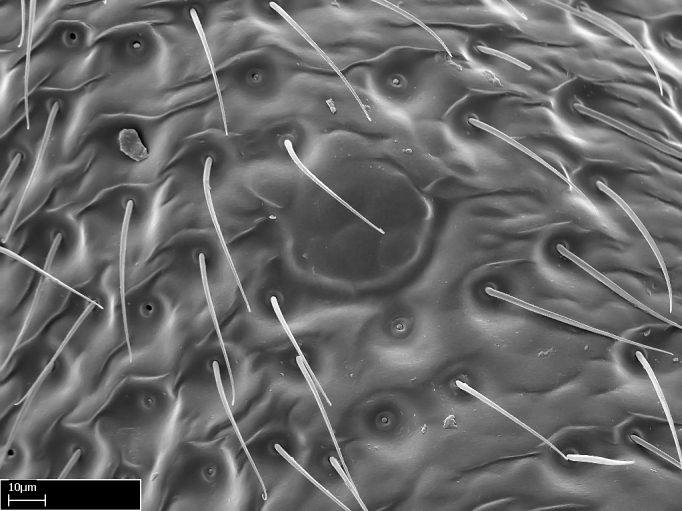
Close-up of the eyes, dorsolateral view (CASENT0009102).

**Figure 60d.**
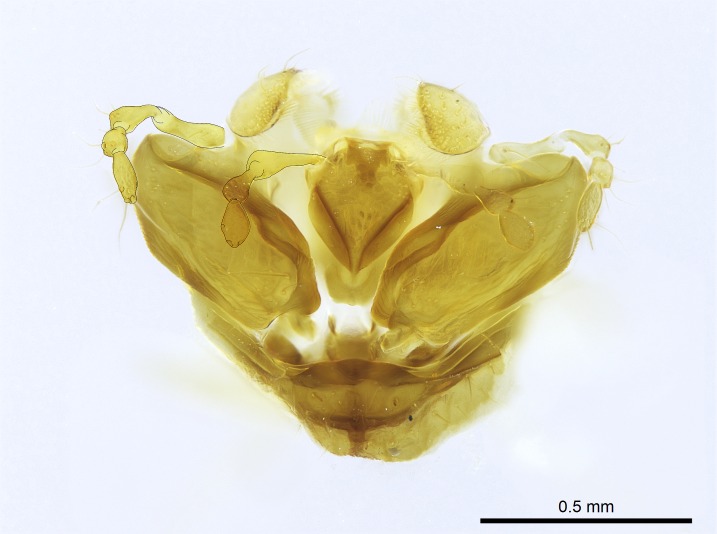
Mouthparts, ventral view (BLF0561(L.O.)-03). Right maxillary and labial palps are outlined in black and darkened to enhance visibility. Slide by F. A. Esteves.

**Figure 61a.**
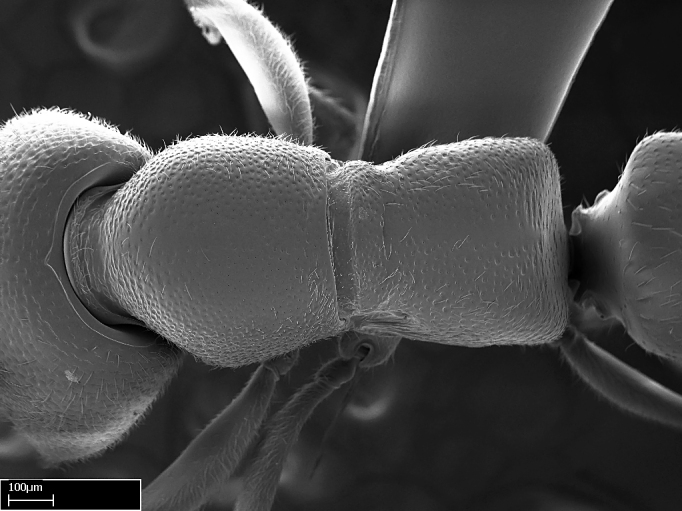
Dorsal view (CASENT0009102).

**Figure 61b.**
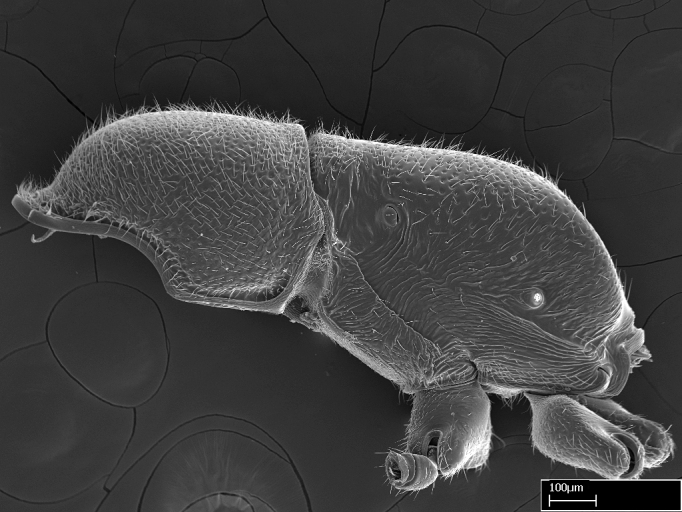
Lateral view (CASENT0724172). The head and metasoma were disarticulated from the mesosoma.

**Figure 62a.**
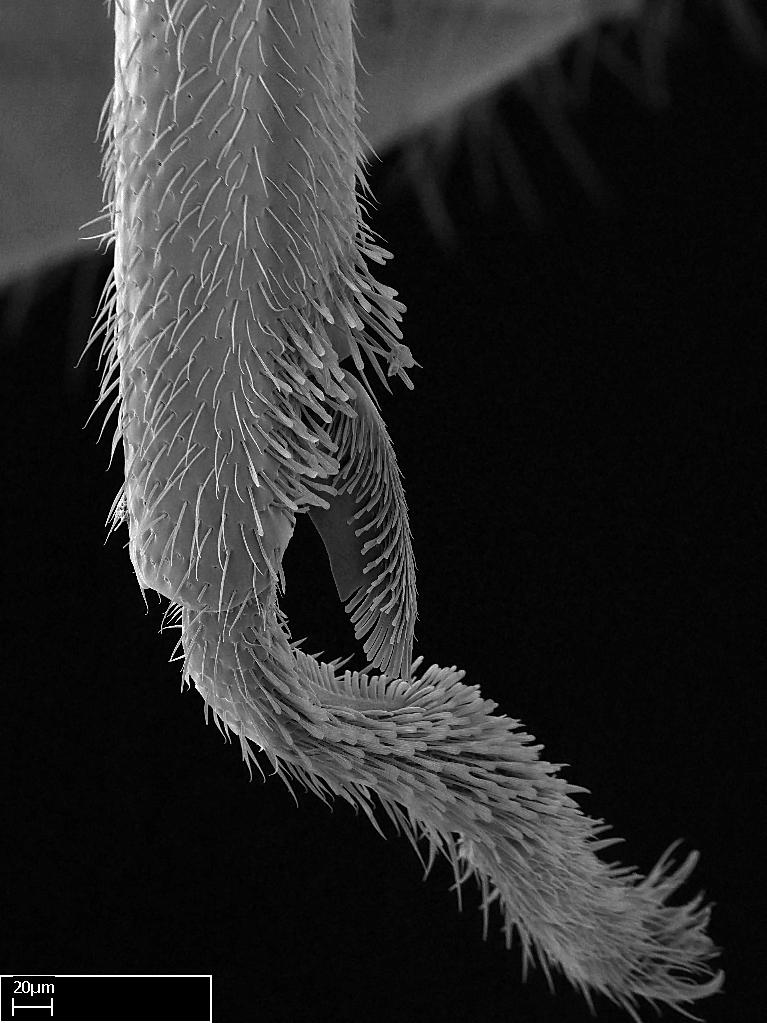
Foreleg (CASENT0009102), anterior face: apical portion of tibia, its associated calcar of strigil, and remainder of apical part of the leg.

**Figure 62b.**
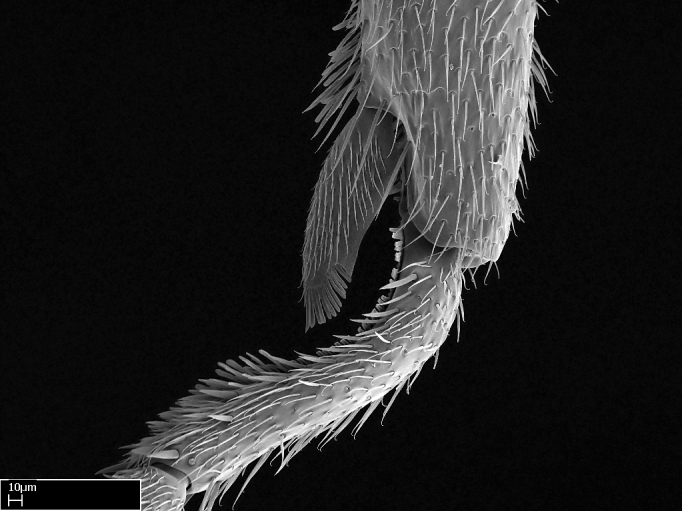
Foreleg (CASENT0009102), posterior face: apical portion of tibia, its associated calcar of strigil, and basitarsus.

**Figure 62c.**
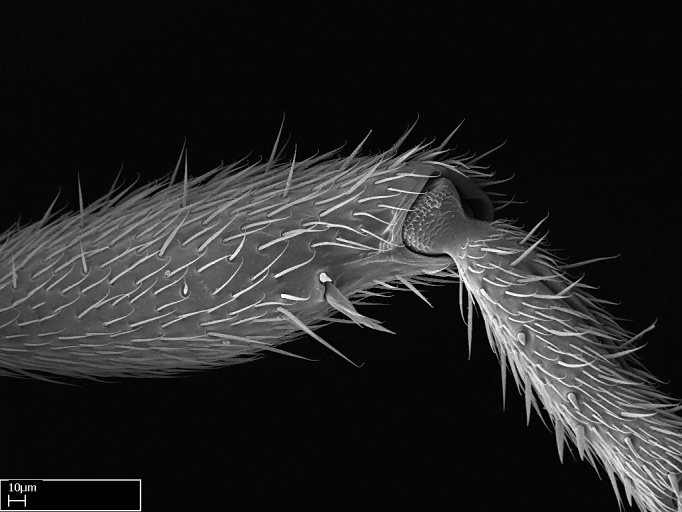
Midleg (CASENT0318413): antero-inner face of apical portion of the tibia and basitarsus.

**Figure 62d.**
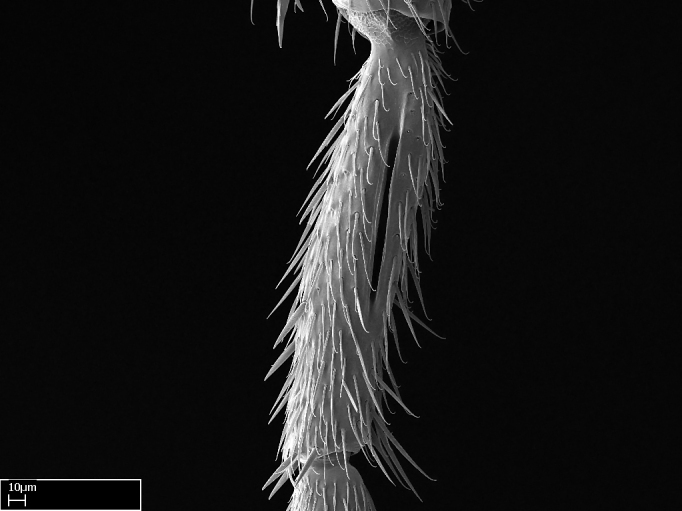
Midleg (CASENT0009102): anterior face of the basitarsus.

**Figure 63a.**
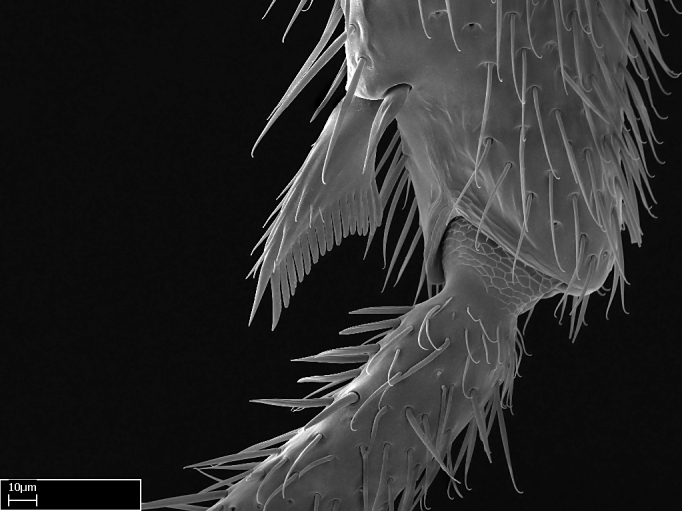
Hindleg, antero-inner face: apical portion of the tibia, its associated spurs, and basal portion of the basitarsus.

**Figure 63b.**
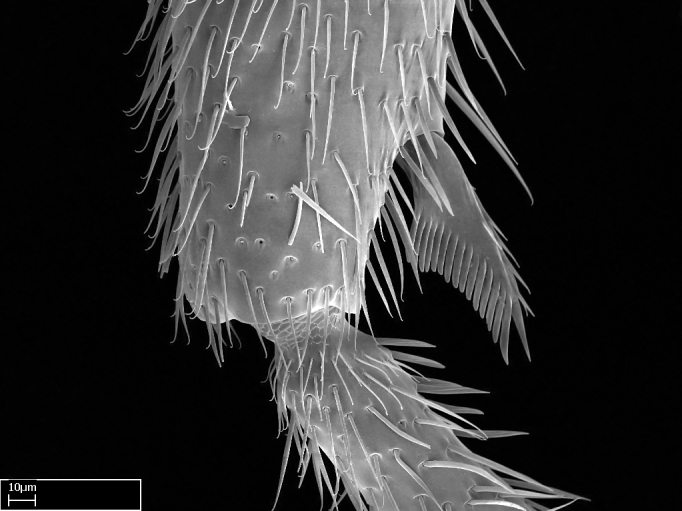
Hindleg, posterior face: apical portion of the tibia, its associated posterior spur, and basal portion of the basitarsus.

**Figure 63c.**
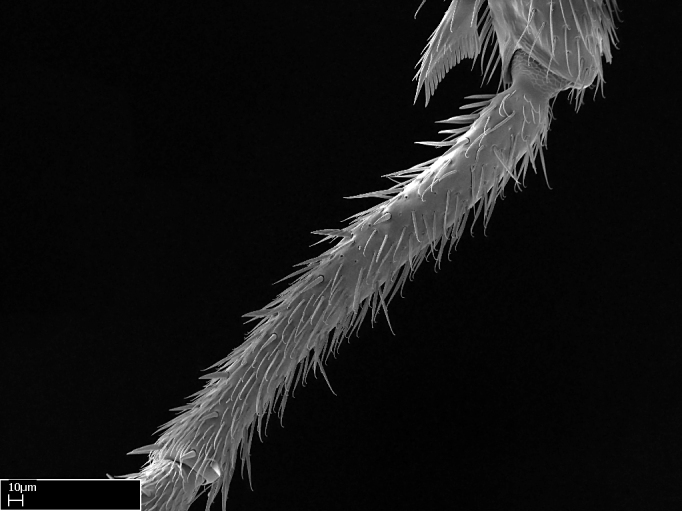
Hindleg, anterior face: apical portion of the tibia and associated posterior spur, and basitarsus.

**Figure 64a.**
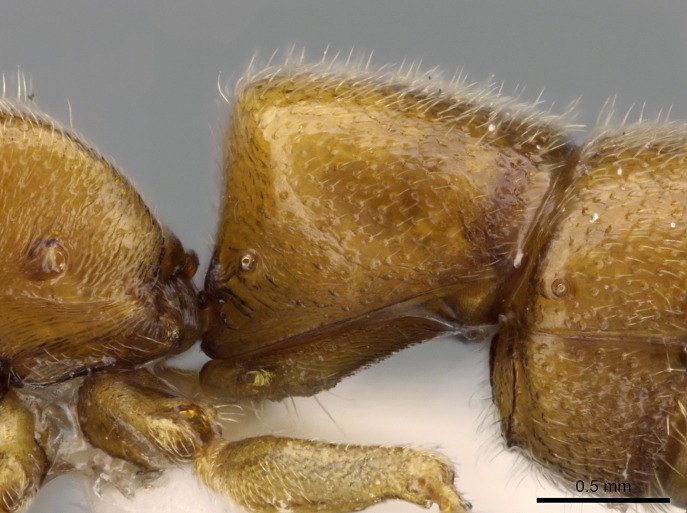
Petiole, lateral view (CASENT0318428).

**Figure 64b.**
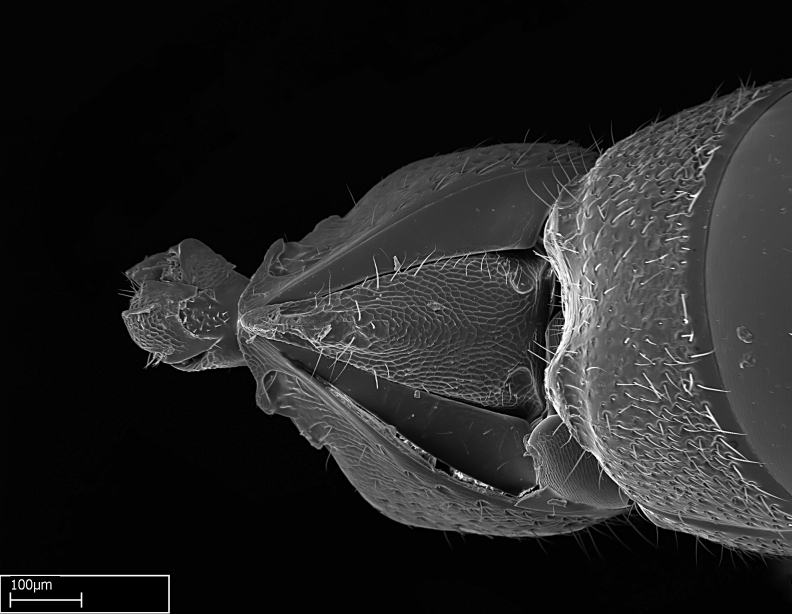
Petiole, ventral view (CASENT0724172).

**Figure 64c.**
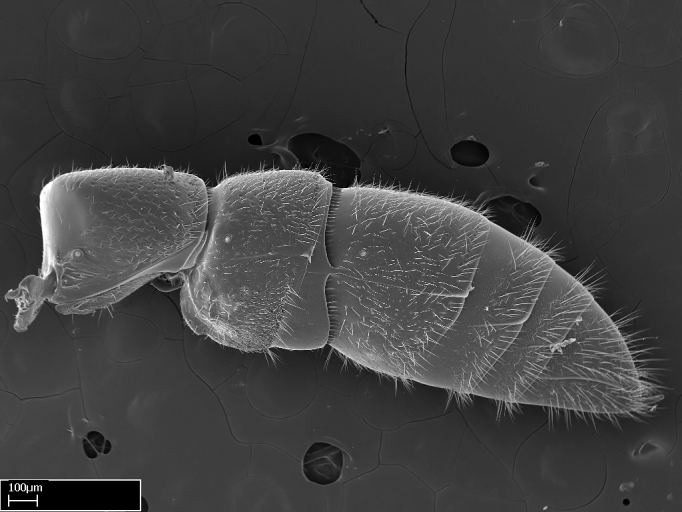
Metasoma, lateral view (CASENT0724172). The mesosoma was disarticulated from the metasoma.

**Figure 64d.**
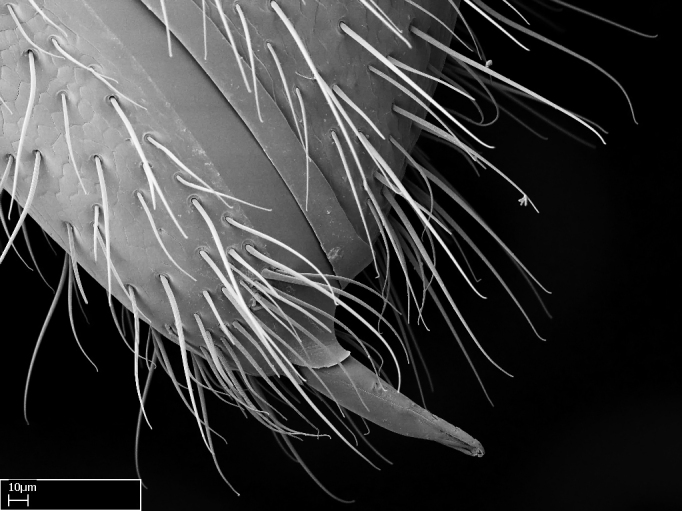
Abdominal segment VII and stinger, lateral view (CASENT0318414).

**Figure 66a.**
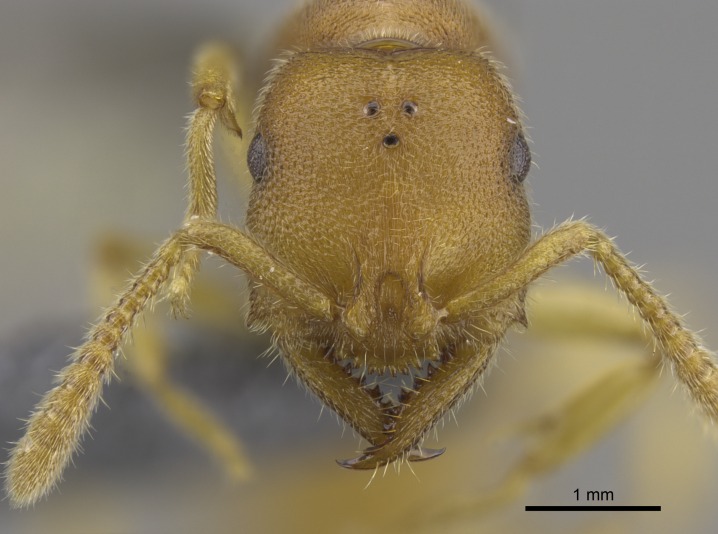
Fullface view.

**Figure 66b.**
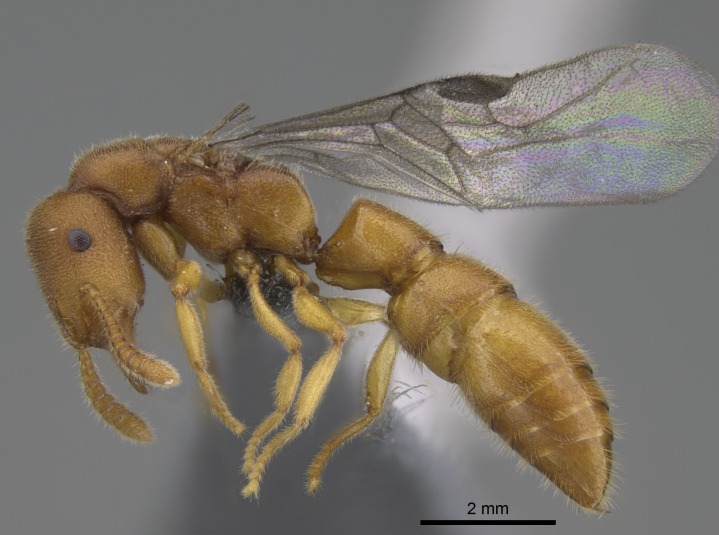
Lateral view. Left wings were removed for better illustration.

**Figure 66c.**
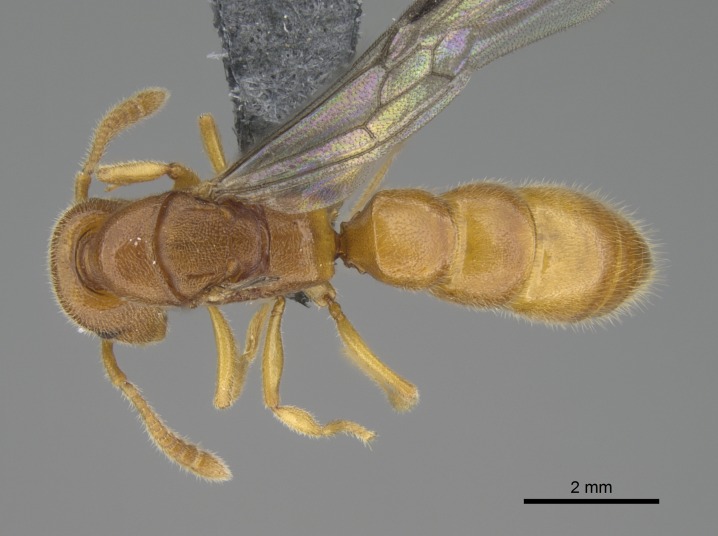
Dorsal view. Left wings were removed for better illustration.

**Figure 66d.**
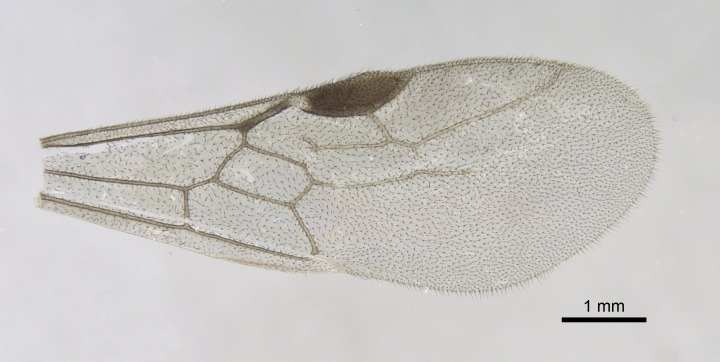
Left forewing. Slide by F. A. Esteves.

**Figure 66e.**
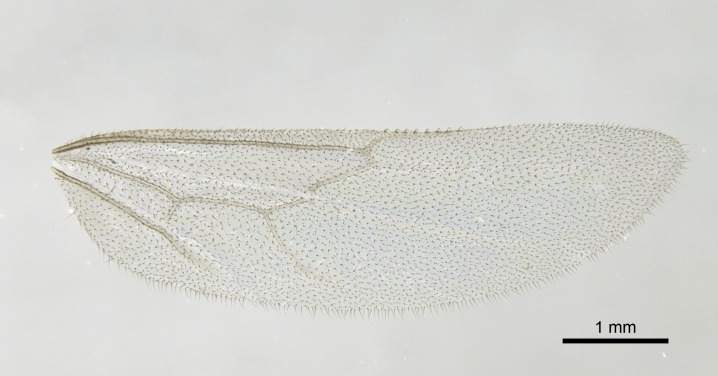
Left hindwing. Slide by F. A. Esteves.

**Figure 67a.**
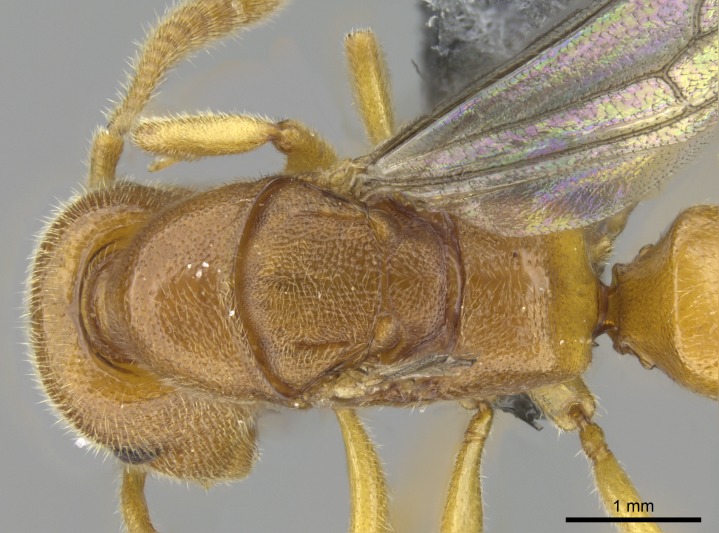
Mesosoma, dorsal view. Left wings were removed for better illustration.

**Figure 67b.**
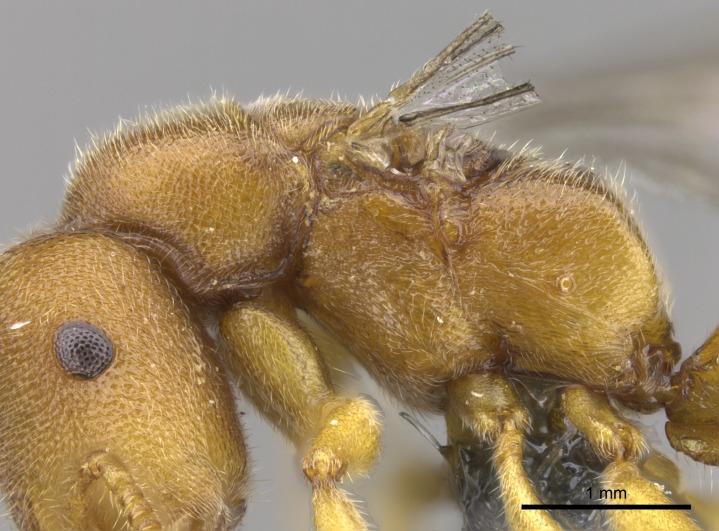
Mesosoma, lateral view. Left wings were removed for better illustration.

**Figure 68a.**
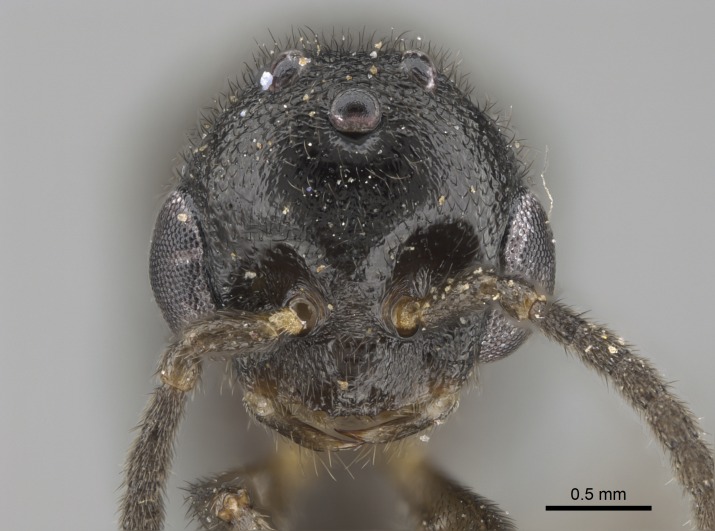
Fullface view.

**Figure 68b.**
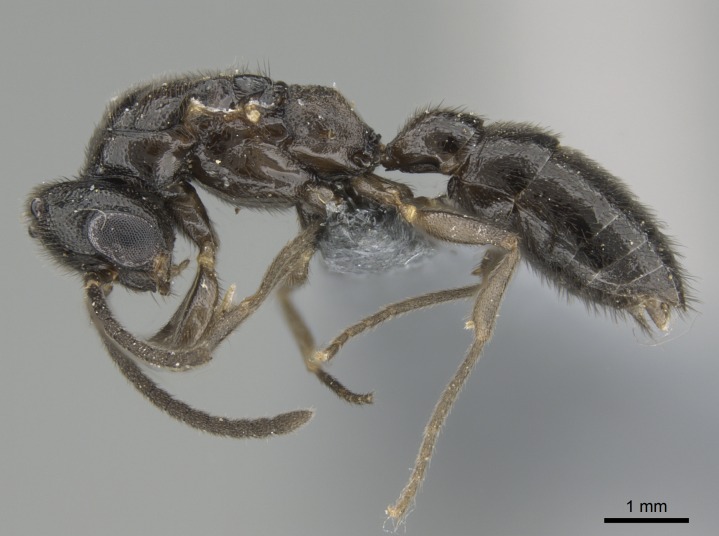
Lateral view. Wings were removed for better illustration.

**Figure 68c.**
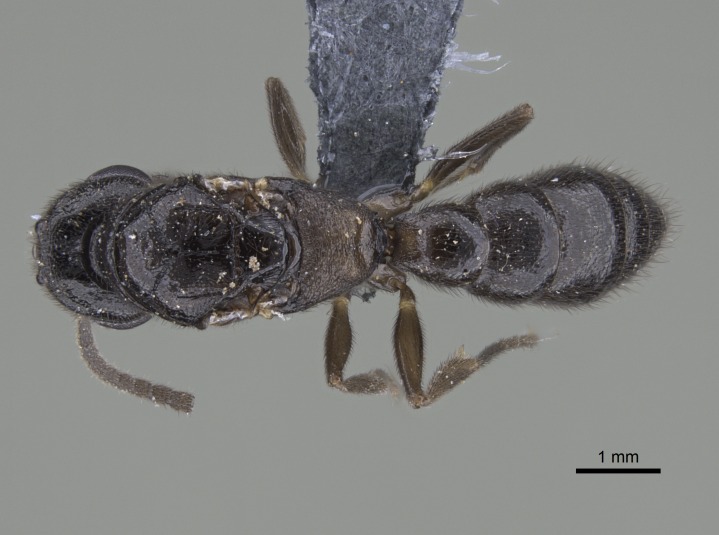
Dorsal view. Wings were removed for better illustration.

**Figure 69a.**
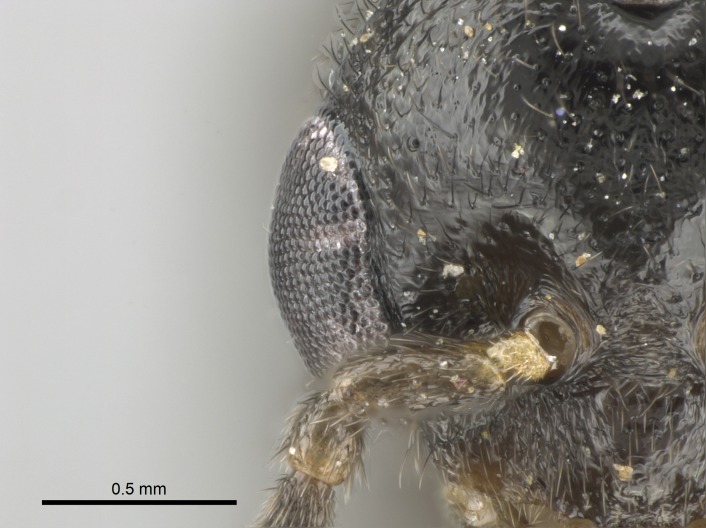
Right compound eye, dorsal view.

**Figure 69b.**
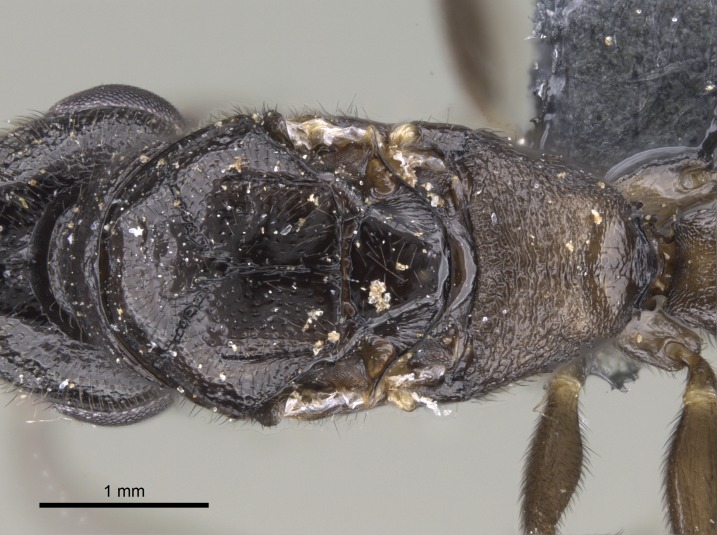
Mesosoma, dorsal view. Wings were removed for better illustration.

**Figure 69c.**
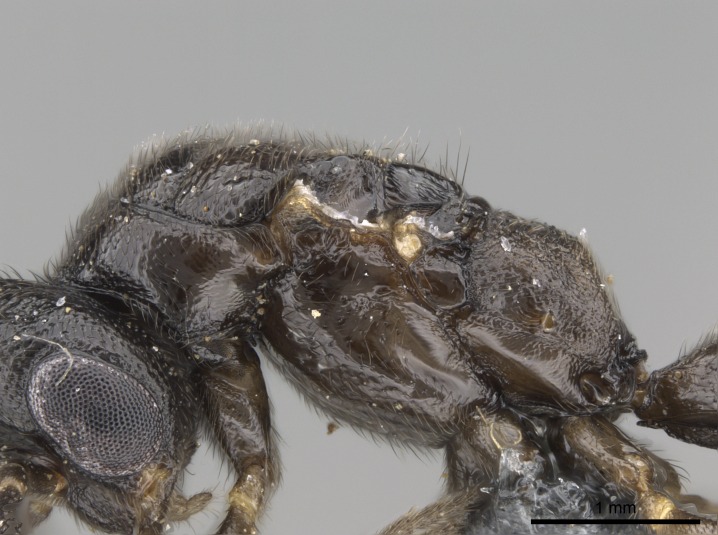
Mesosoma, lateral view. Wings were removed for better illustration.

**Figure 69d.**
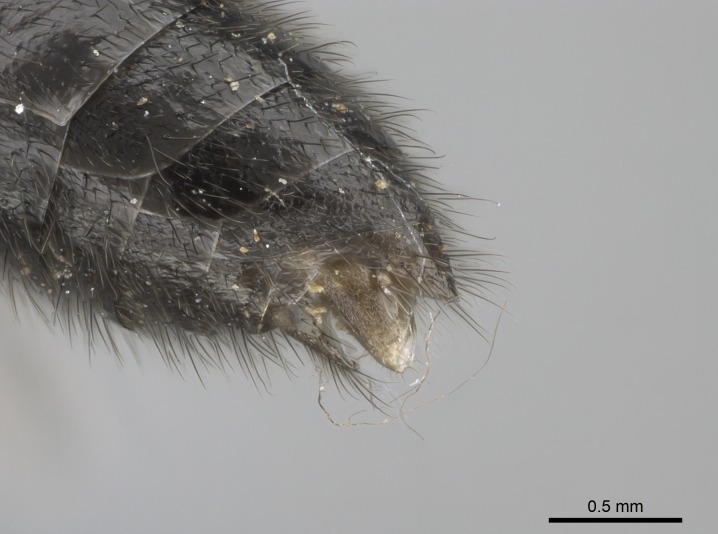
Apex of the gaster, lateral view.

**Figure 70a.**
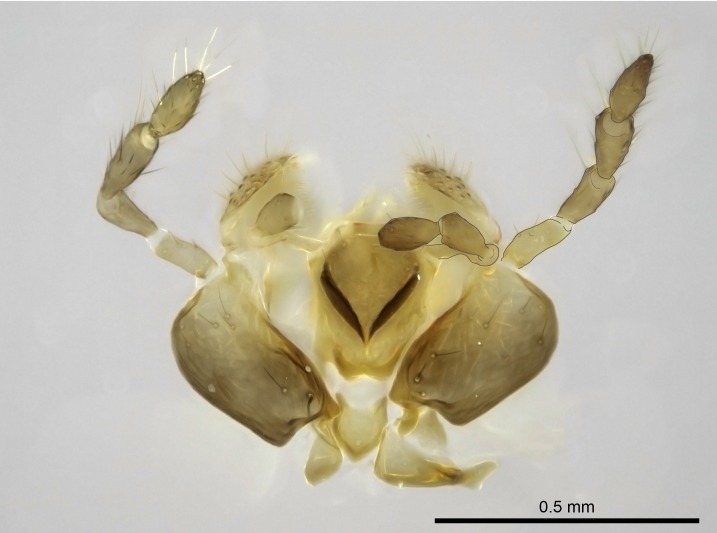
Mouthparts, ventral view. Left maxillary and labial palps are outlined in gray and darkened to enhance visibility. Slide by F. A. Esteves.

**Figure 70b.**
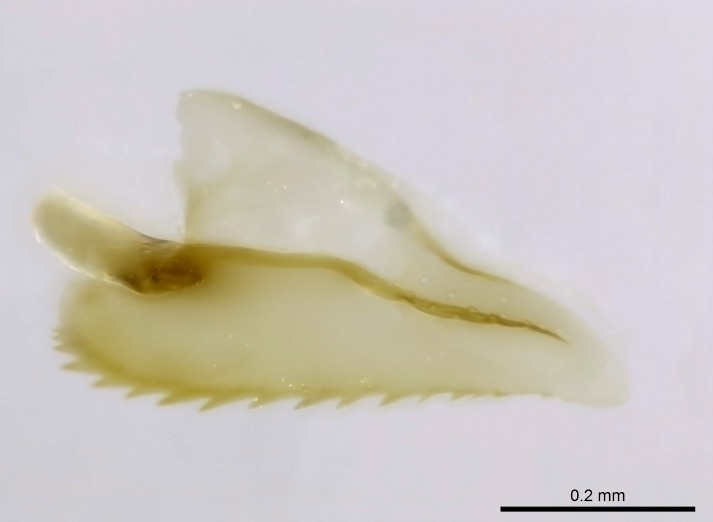
Aedeagus, lateral view. Slide by F. A. Esteves.

**Figure 70c.**
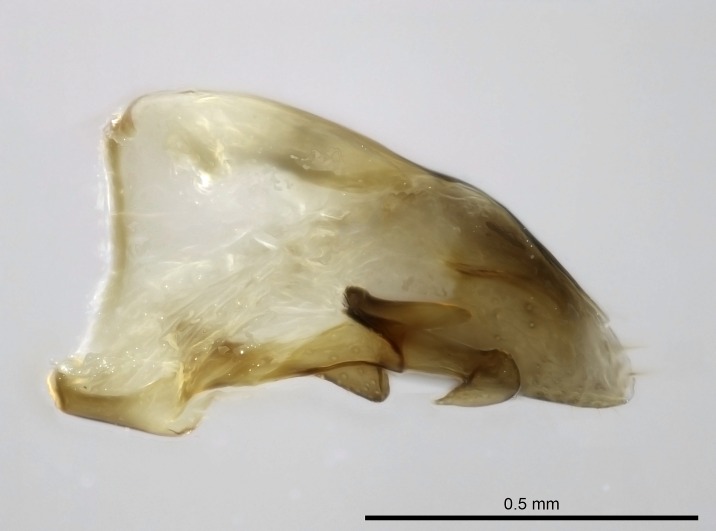
Longitudinal section of the genital capsule; inner face, lateral view. The basal ring was removed from the specimen. Slide by F. A. Esteves.

**Figure 70d.**
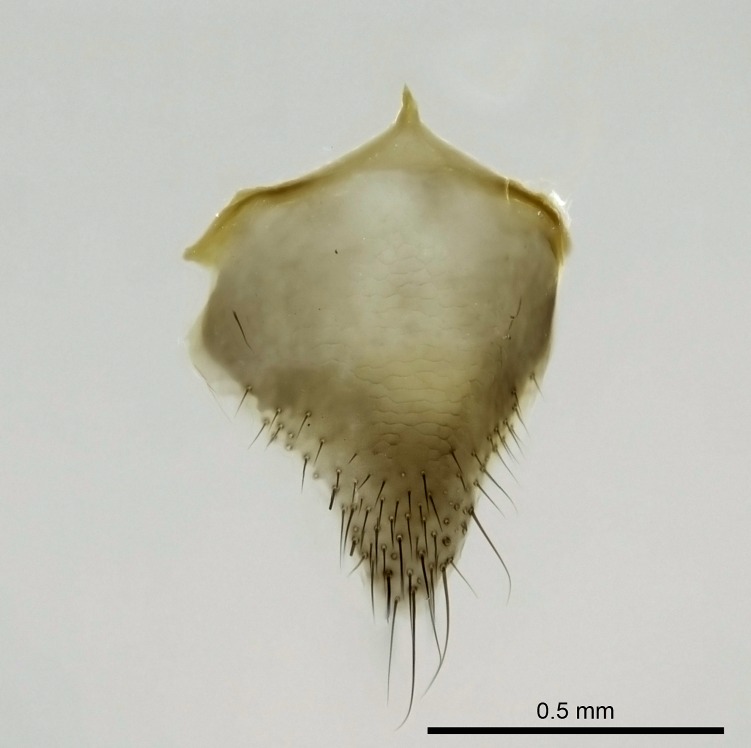
Abdominal sternum IX, ventral view. Slide by F. A. Esteves.

**Figure 71a.**
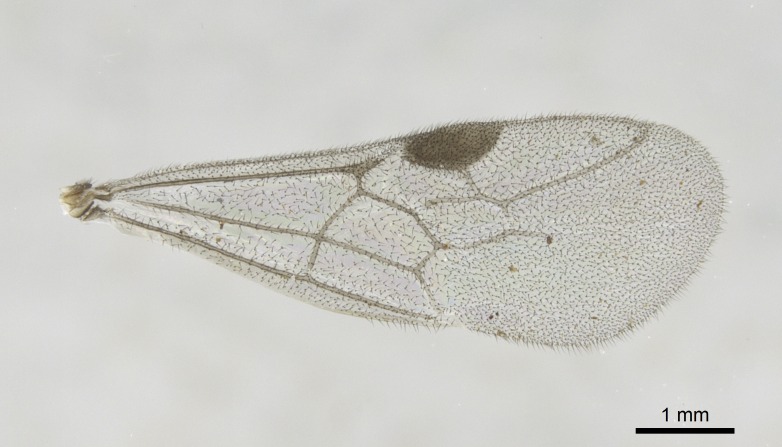
Right forewing. Slide by F. A. Esteves.

**Figure 71b.**
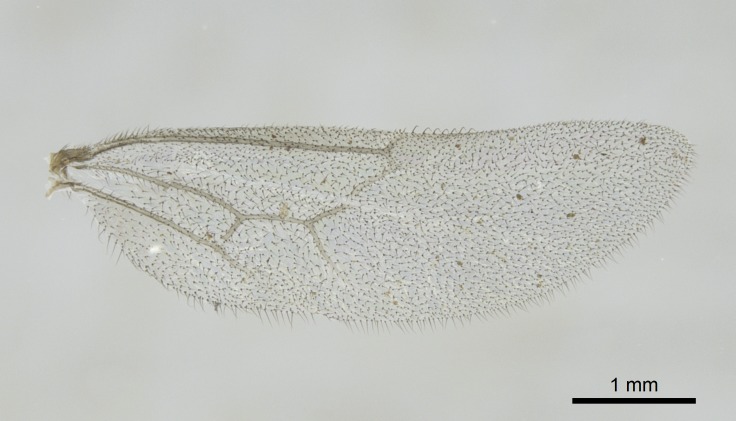
Right hindwing. Slide by F. A. Esteves.

**Figure 73a.**
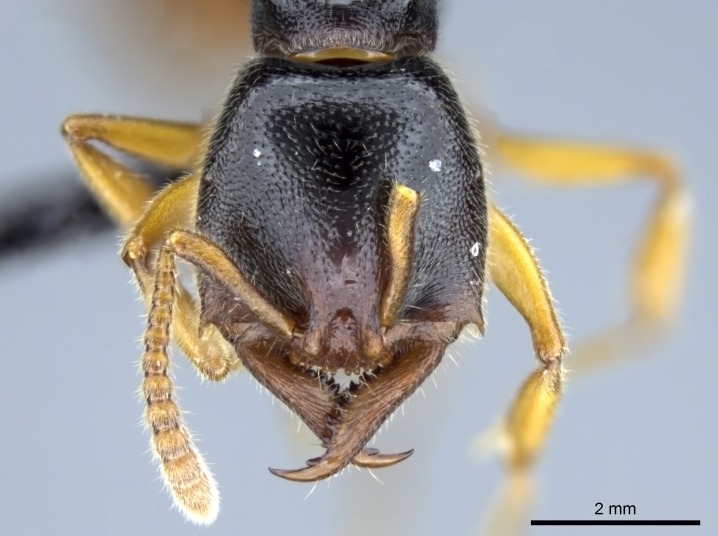
Fullface view.

**Figure 73b.**
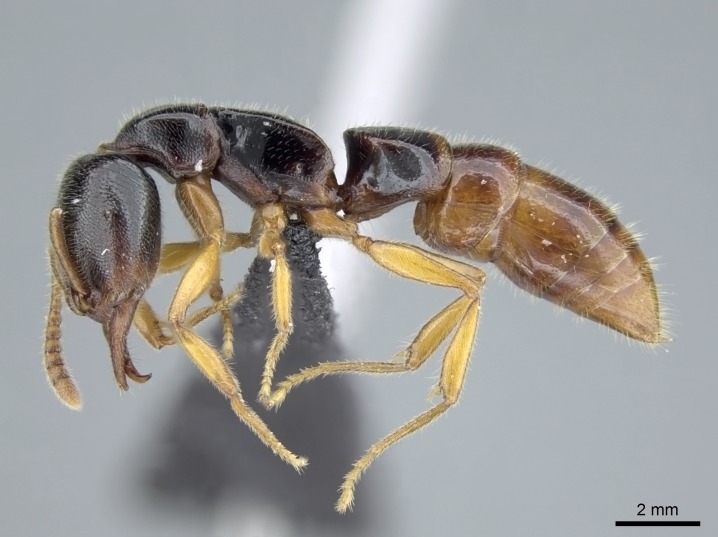
Lateral view.

**Figure 73c.**
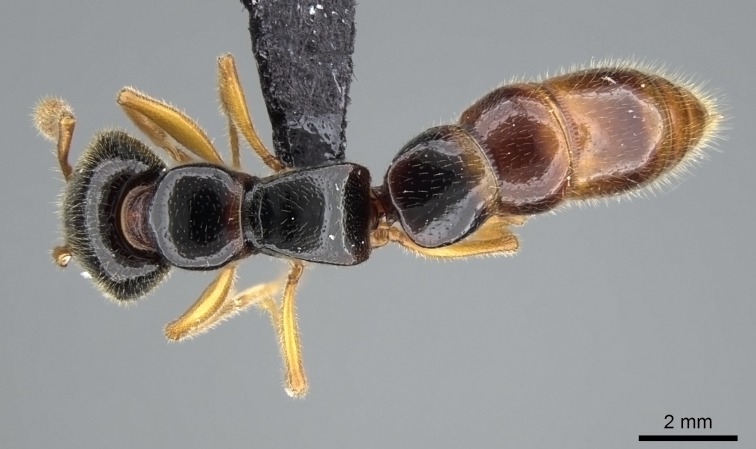
Dorsal view.

**Figure 74a.**
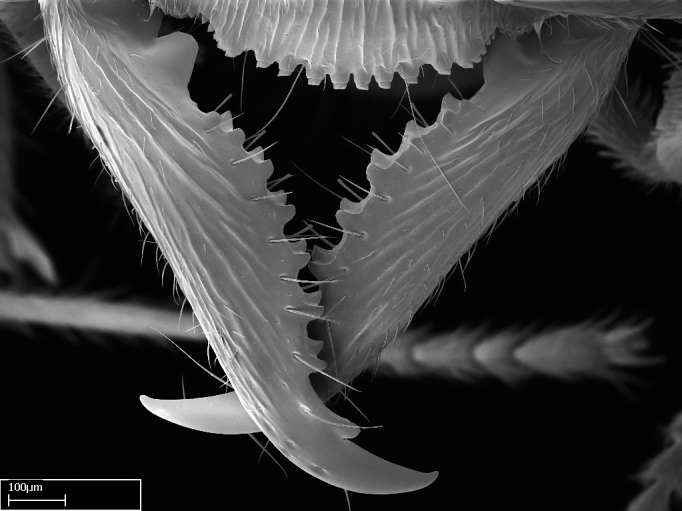
Dorsal view of the mandibles and anterior part of the head (CASENT0004339).

**Figure 74b.**
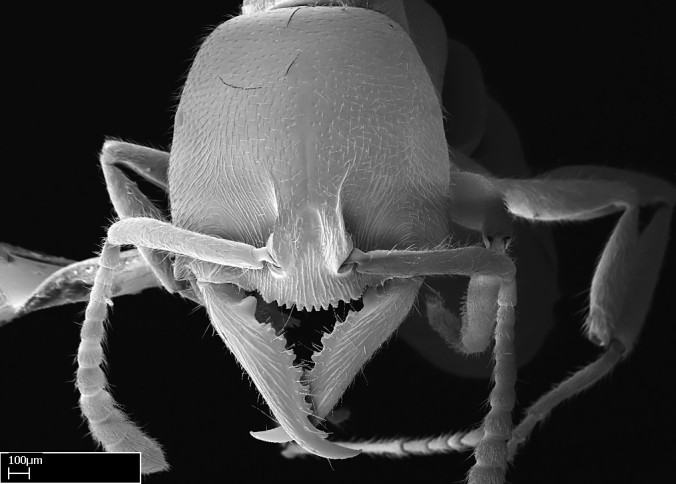
Fullface view (CASENT0004339).

**Figure 74c.**
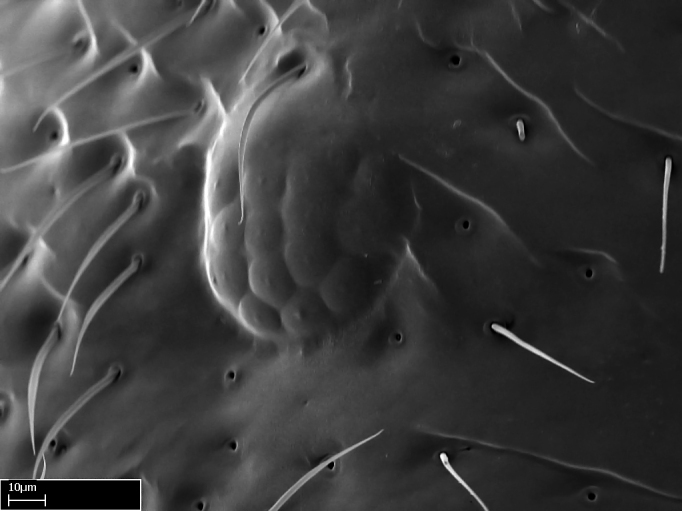
Close-up of the eyes, dorsolateral view (CASENT0056916).

**Figure 74d.**
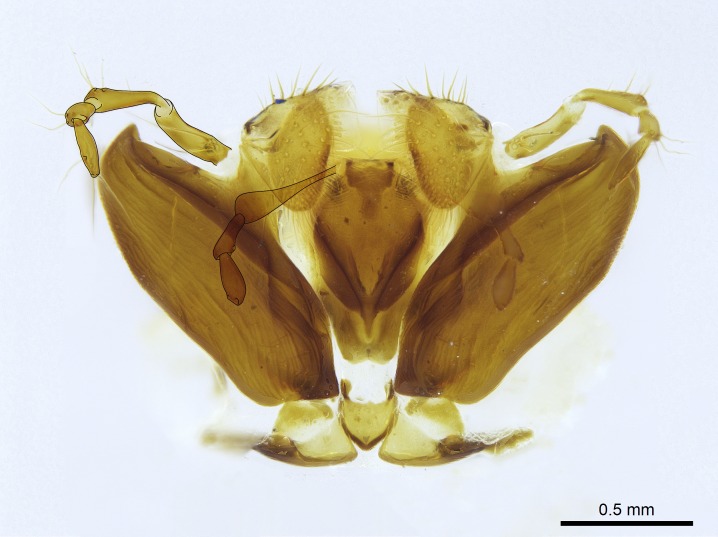
Mouthparts, ventral view (CASENT0318480). Right maxillary and labial palps are outlined in black and darkened to enhance visibility. Slide by F. A. Esteves.

**Figure 75a.**
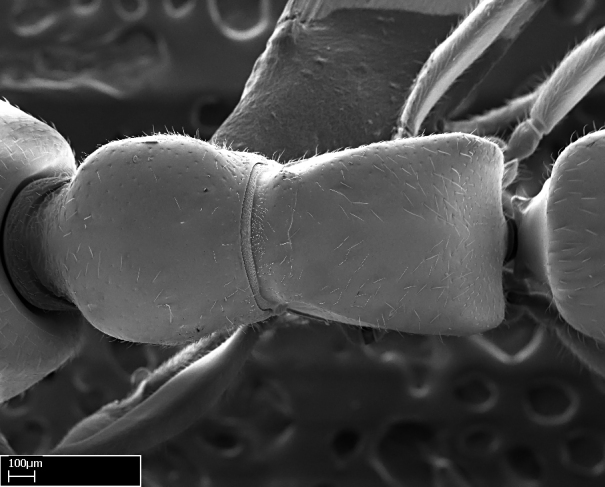
Dorsal view (CASENT0056916).

**Figure 75b.**
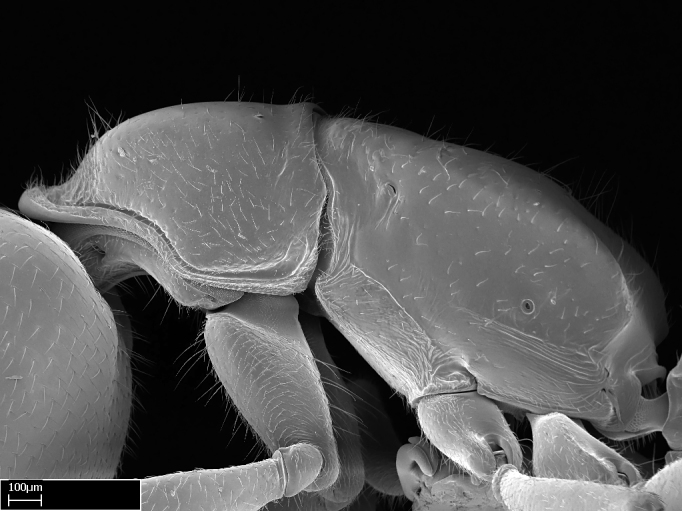
Lateral view (CASENT0004339).

**Figure 76a.**
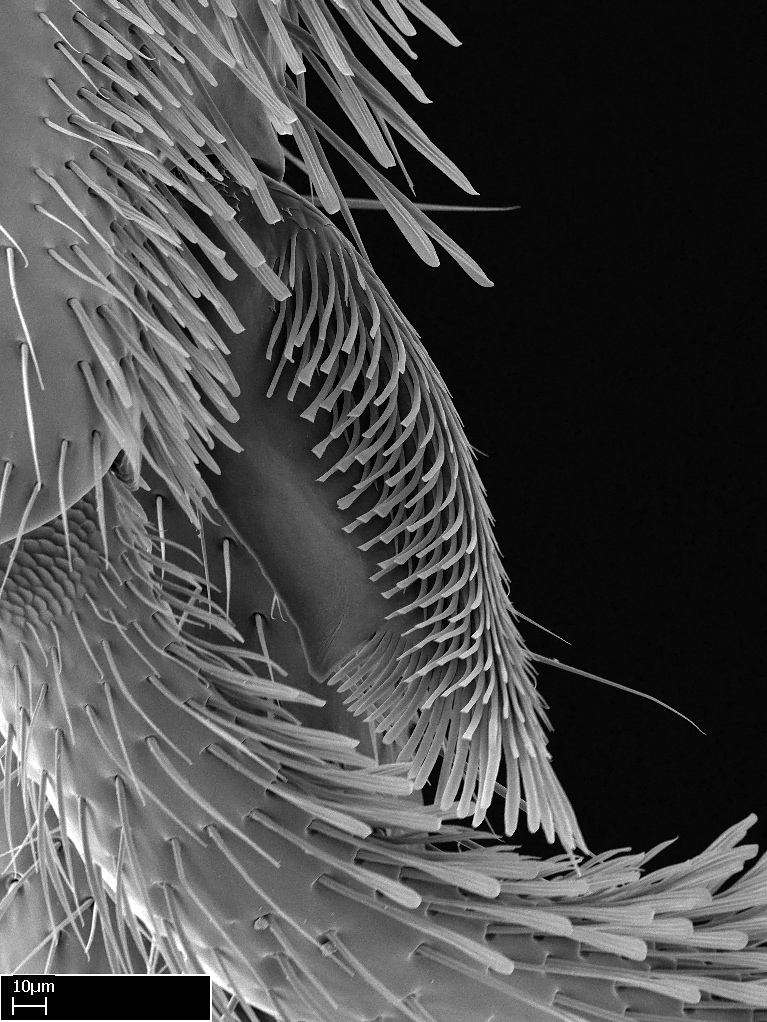
Foreleg (CASENT0002078), anterior face: apical portion of the tibia, its associated calcar of strigil, and basal portion of the basitarsus.

**Figure 76b.**
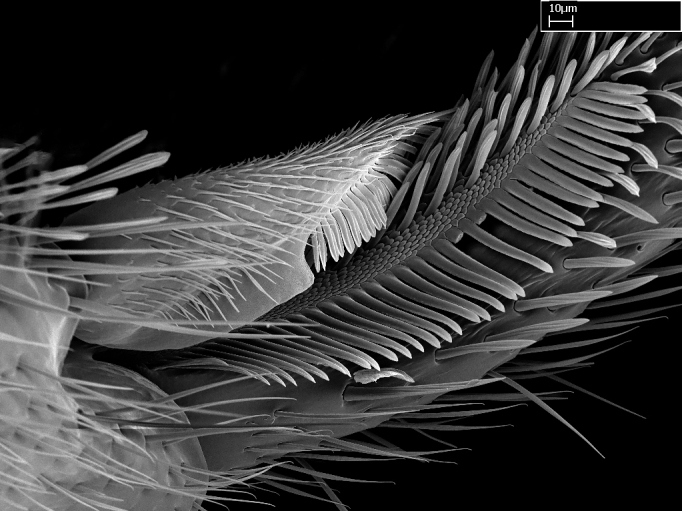
Foreleg (CASENT0056916), posterior face: apical portion of tibia, its associated calcar of strigil, and basal portion of the basitarsus.

**Figure 76c.**
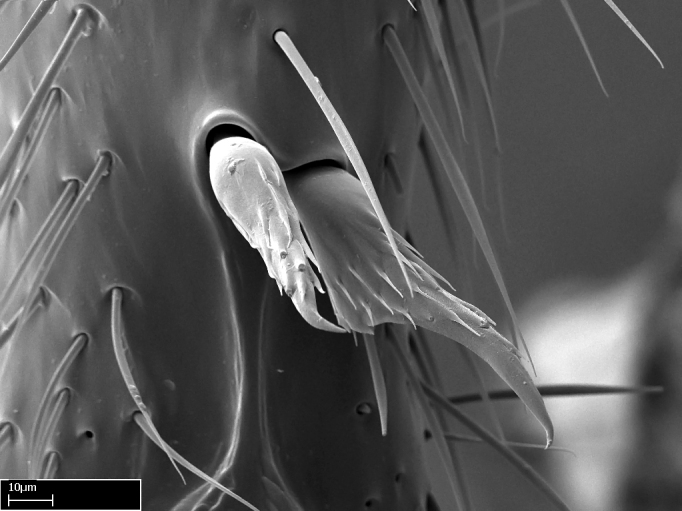
Midleg (CASENT0004339), ventral view: apical portion of the tibia, and its associated spurs. Anterior spur on the left; posterior spur on the right.

**Figure 76d.**
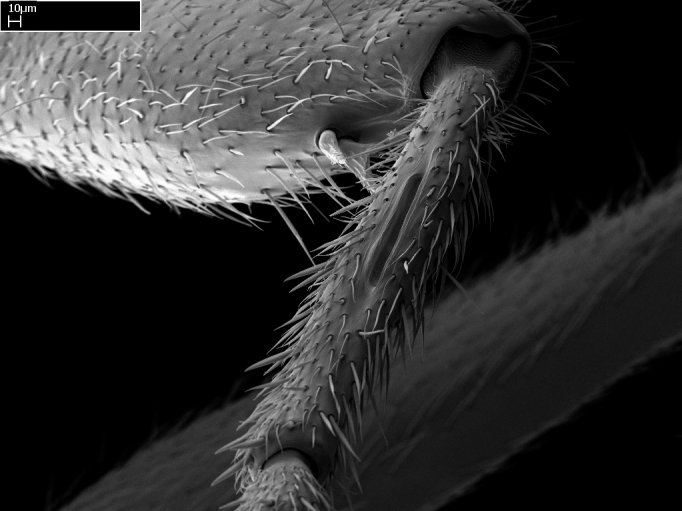
Midleg (CASENT0056916), anteroventral view: apical portion of the tibia, its associated spurs, and basitarsus.

**Figure 77a.**
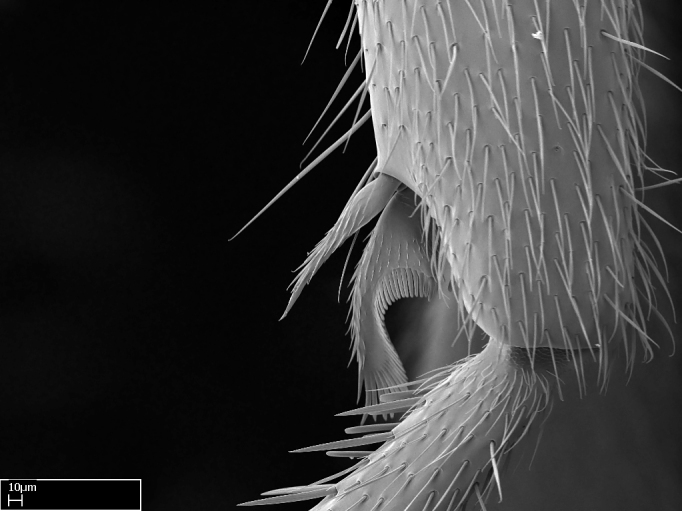
Hindleg (CASENT0056916), anterior face: apical portion of the tibia, its associated spurs, and basal portion of the basitarsus.

**Figure 77b.**
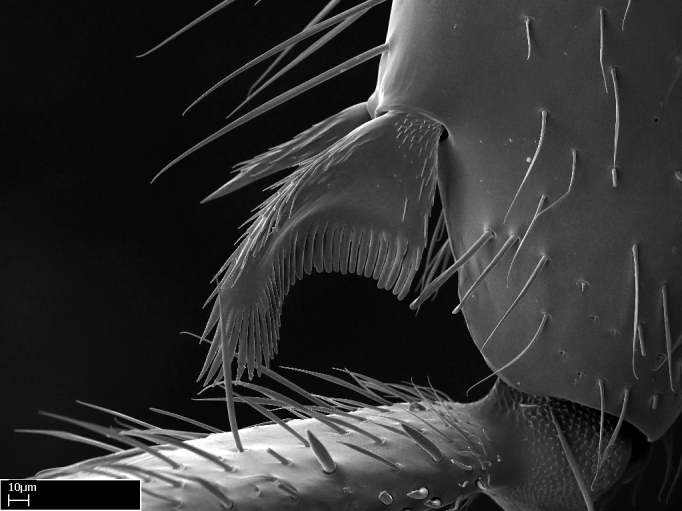
Hindleg (CASENT0004339), posterior face: apical portion of the tibia, its associated spurs, and basal portion of the basitarsus.

**Figure 77c.**
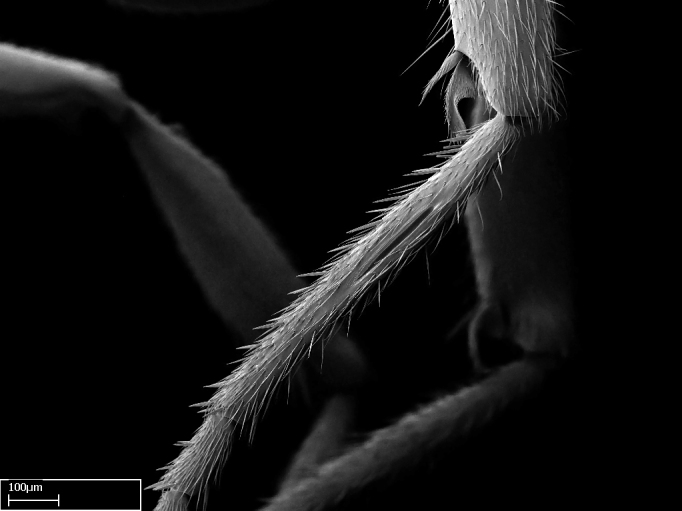
Hindleg (CASENT0056916), anterior face: apical portion of the tibia, its associated spurs, and basitarsus.

**Figure 78a.**
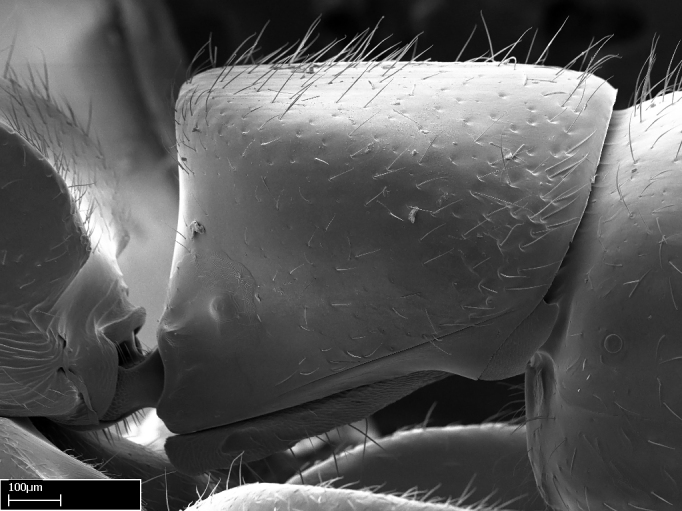
Petiole (CASENT0004339), lateral view.

**Figure 78b.**
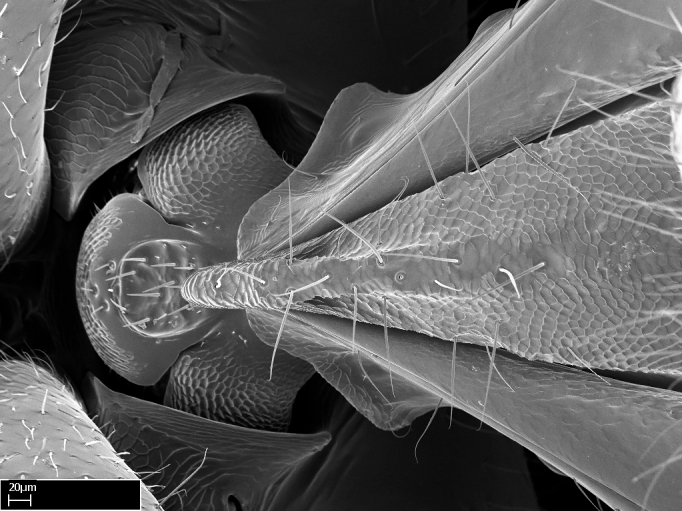
Petiole (CASENT0004339), ventral view.

**Figure 78c.**
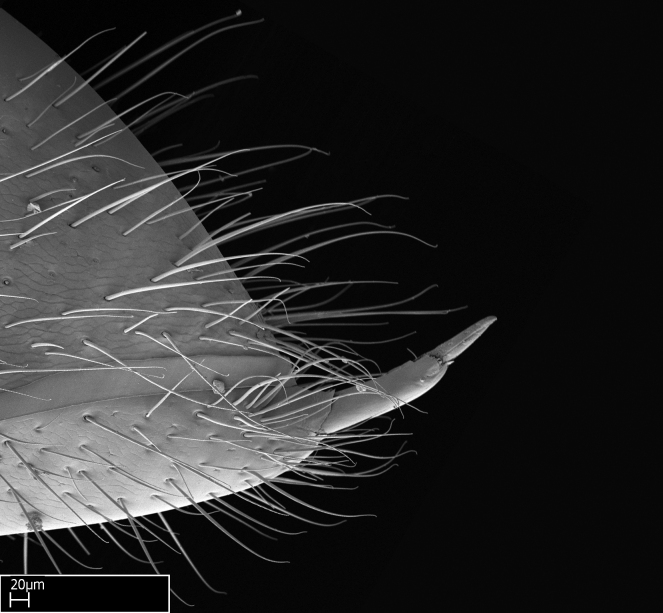
Abdominal segment VII and stinger (CASENT0002078), lateral view.

**Figure 80a.**
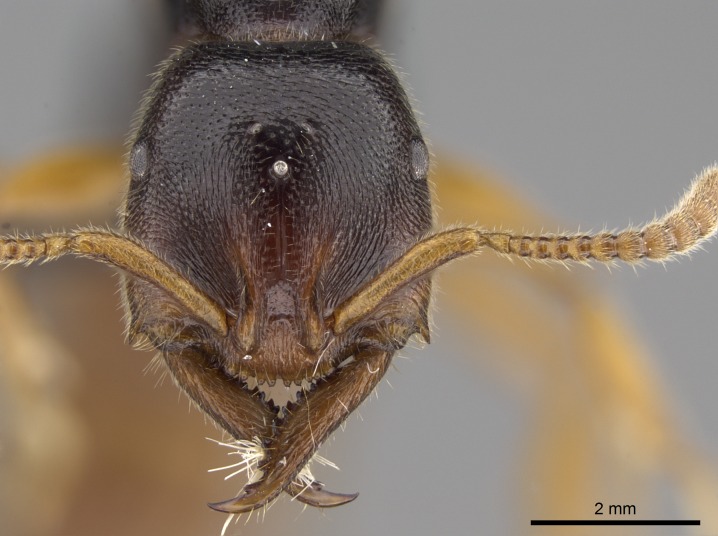
Fullface view.

**Figure 80b.**
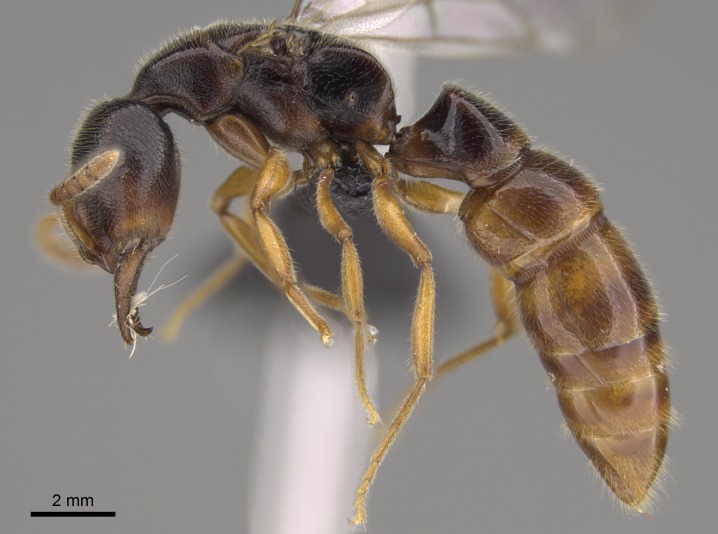
Lateral view. Left wings were removed for better illustration.

**Figure 80c.**
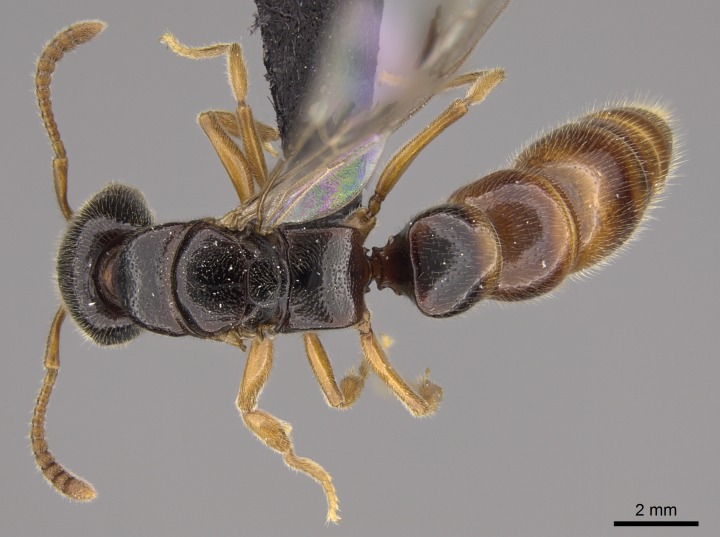
Dorsal view. Left wings were removed for better illustration.

**Figure 81a.**
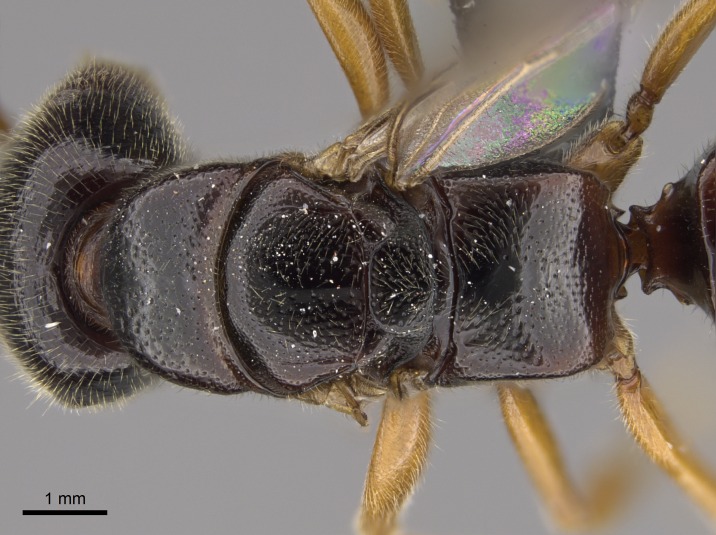
Mesosoma, dorsal view. Left wings were removed for better illustration.

**Figure 81b.**
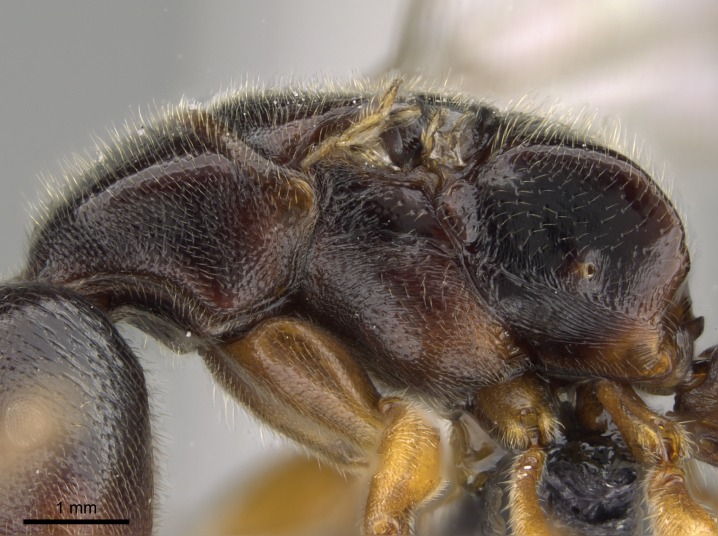
Mesosoma, lateral view. Left wings were removed for better illustration.

**Figure 81c.**
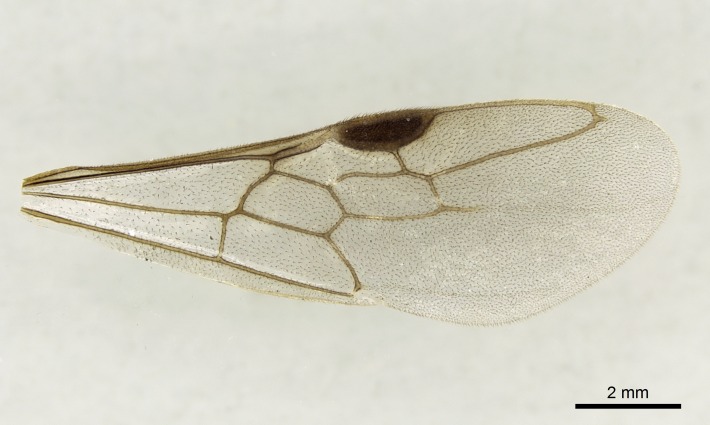
Left forewing.

**Figure 81d.**
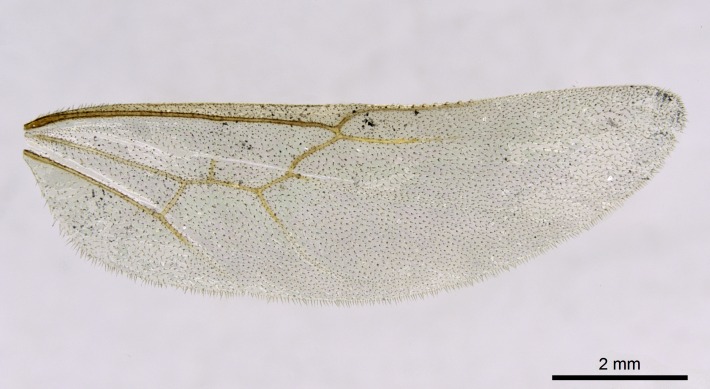
left hindwing.

**Figure 82a.**
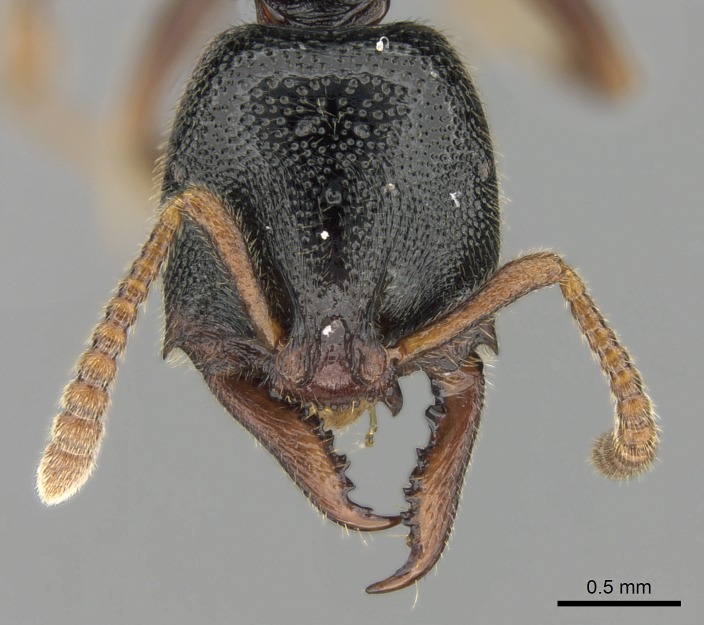
Fullface view.

**Figure 82b.**
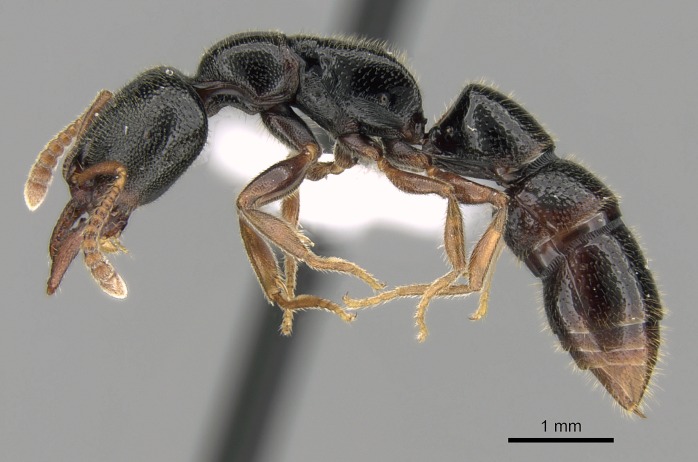
Lateral view.

**Figure 82c.**
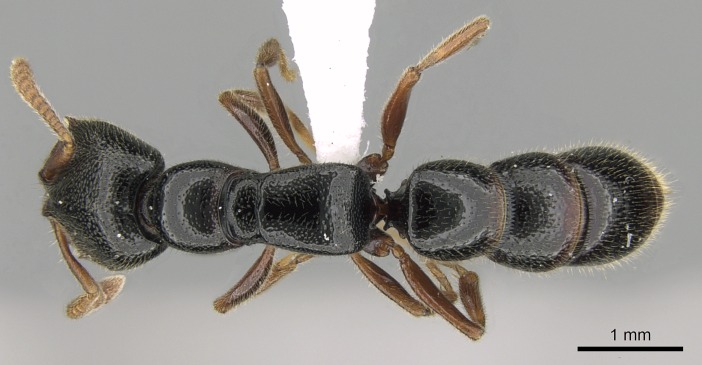
Dorsal view.

**Figure 83a.**
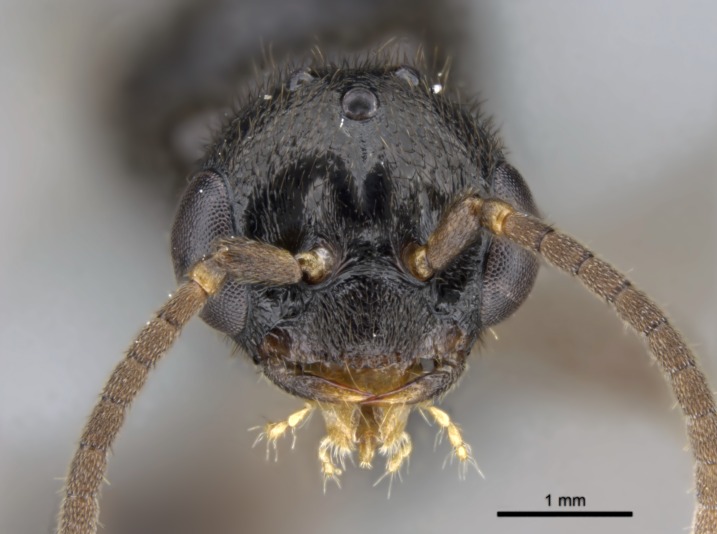
Fullface view.

**Figure 83b.**
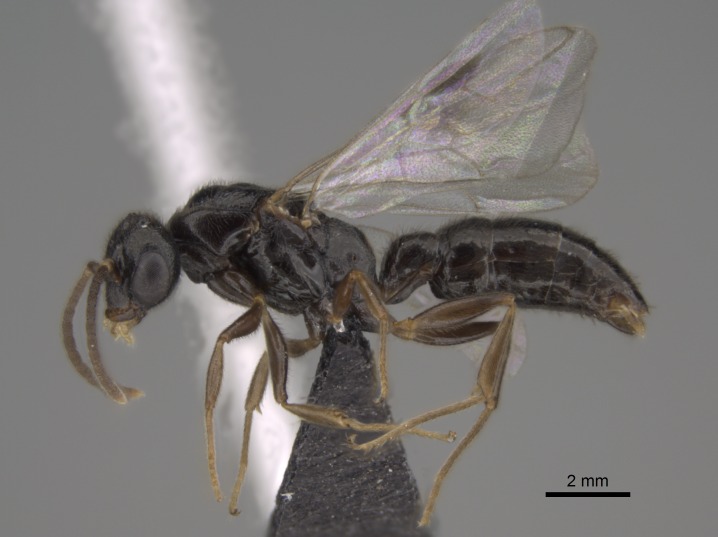
Lateral view.

**Figure 83c.**
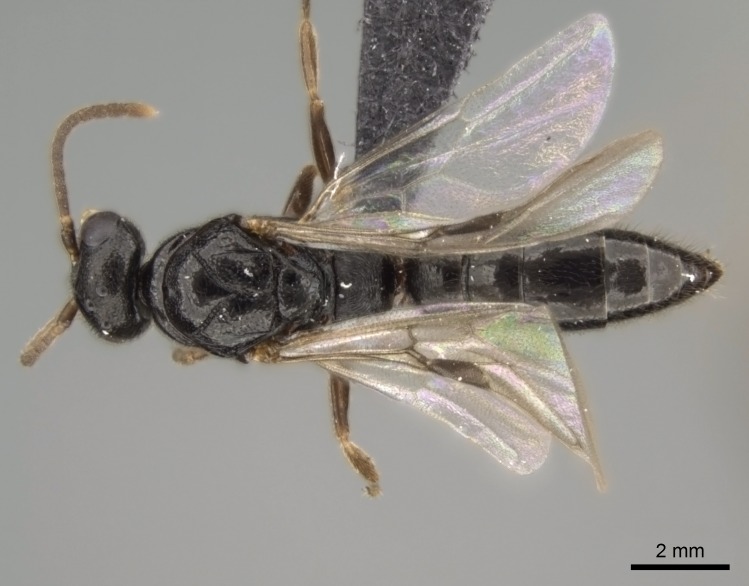
Dorsal view.

**Figure 84a.**
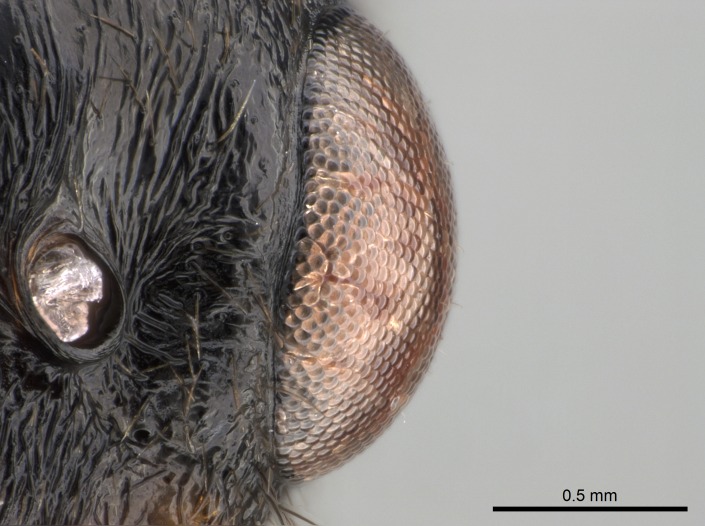
Left compound eye, dorsal view (CASENT0318450).

**Figure 84b.**
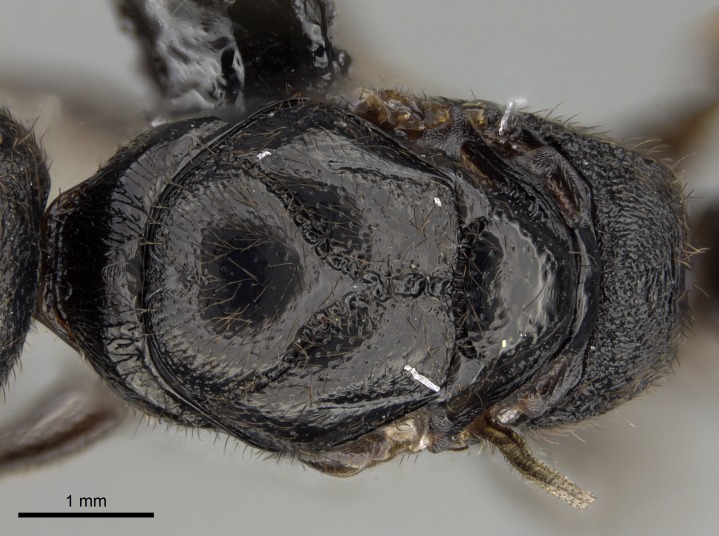
Mesosoma, dorsal view (CASENT0318450). Wings were removed for better illustration.

**Figure 84c.**
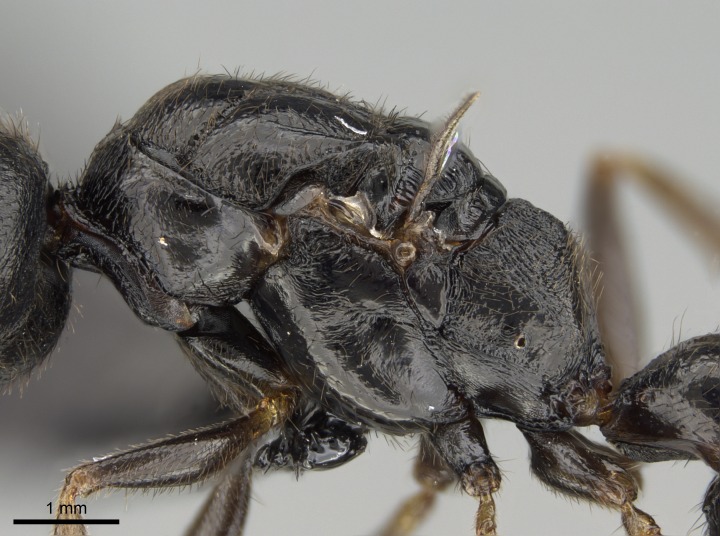
Mesosoma, lateral view (CASENT0318450). Wings were removed for better illustration.

**Figure 84d.**
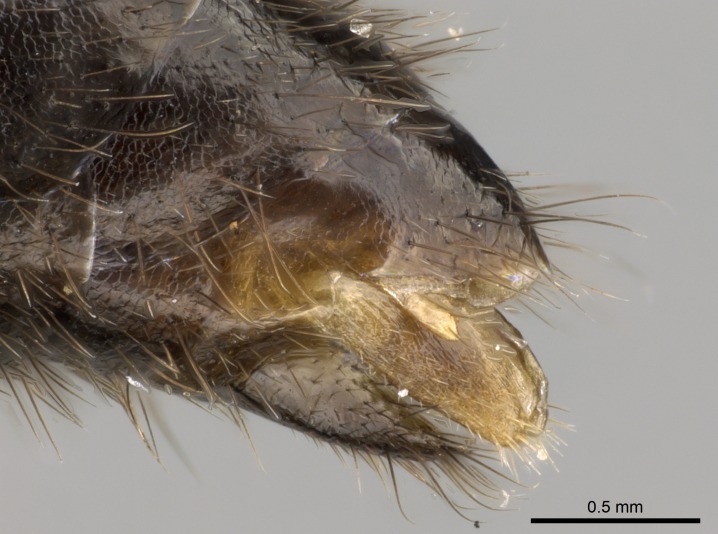
Apex of the gaster, lateral view (CASENT0107483).

**Figure 85a.**
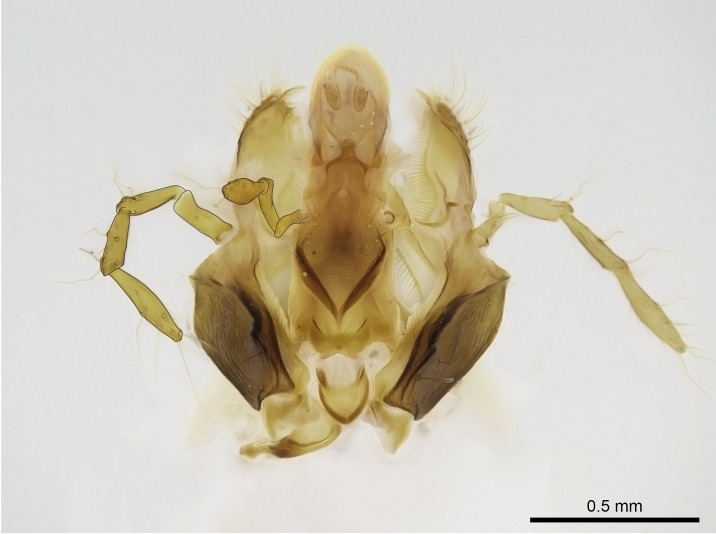
Mouthparts, ventral view. Right maxillary and labial palps are outlined in gray and darkened to enhance visibility. Slide by F. A. Esteves.

**Figure 85b.**
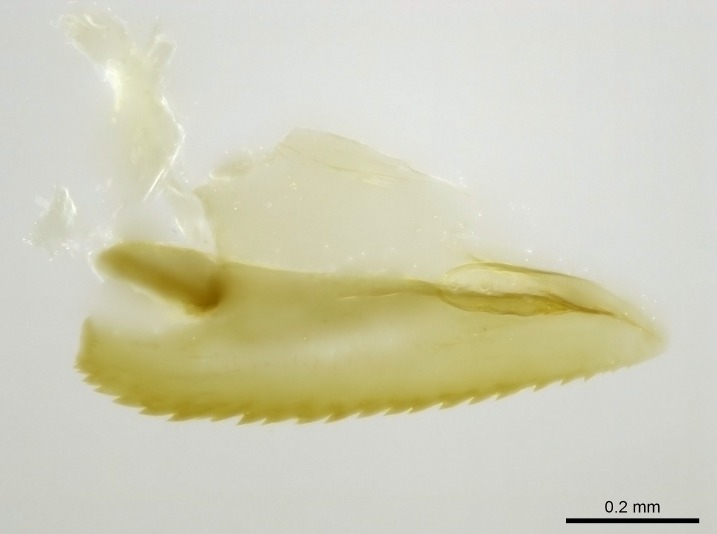
Aedeagus, lateral view. Slide by F. A. Esteves.

**Figure 85c.**
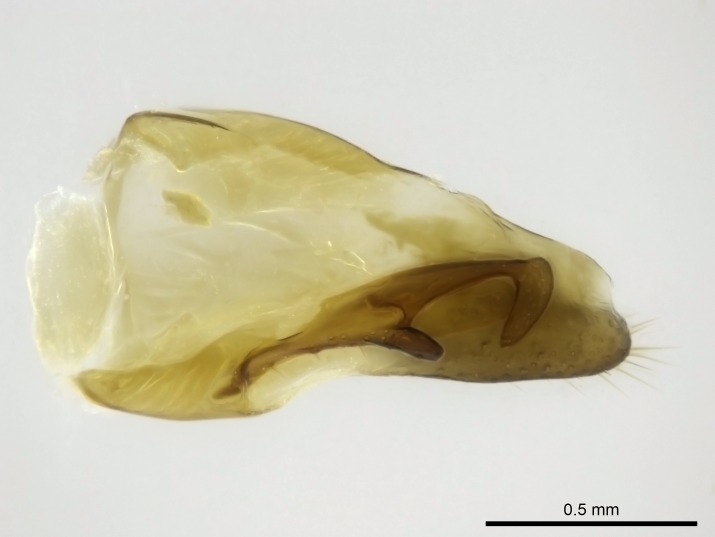
Longitudinal section of the genital capsule; inner face, lateral view. The basal ring was removed from the specimen. Slide by F. A. Esteves.

**Figure 85d.**
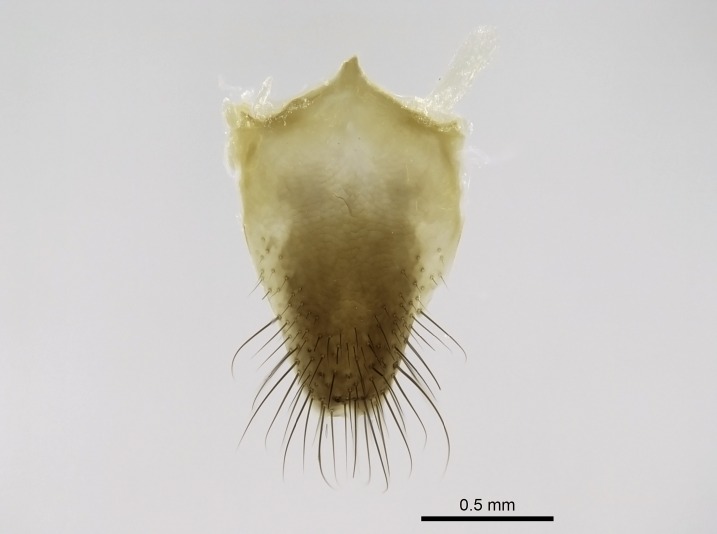
Abdominal sternun IX, ventral view. Slide by F. A. Esteves.

**Figure 86a.**
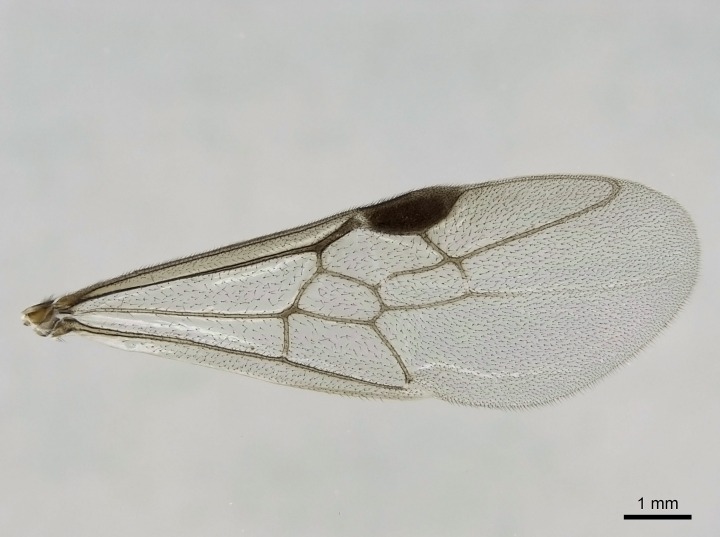
Right forewing. Slide by F. A. Esteves

**Figure 86b.**
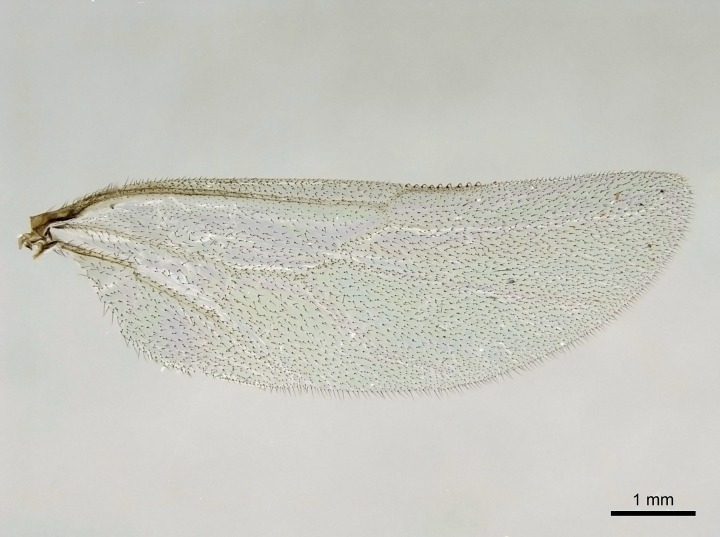
Right hindwing. Slide by F. A. Esteves

**Figure 88a.**
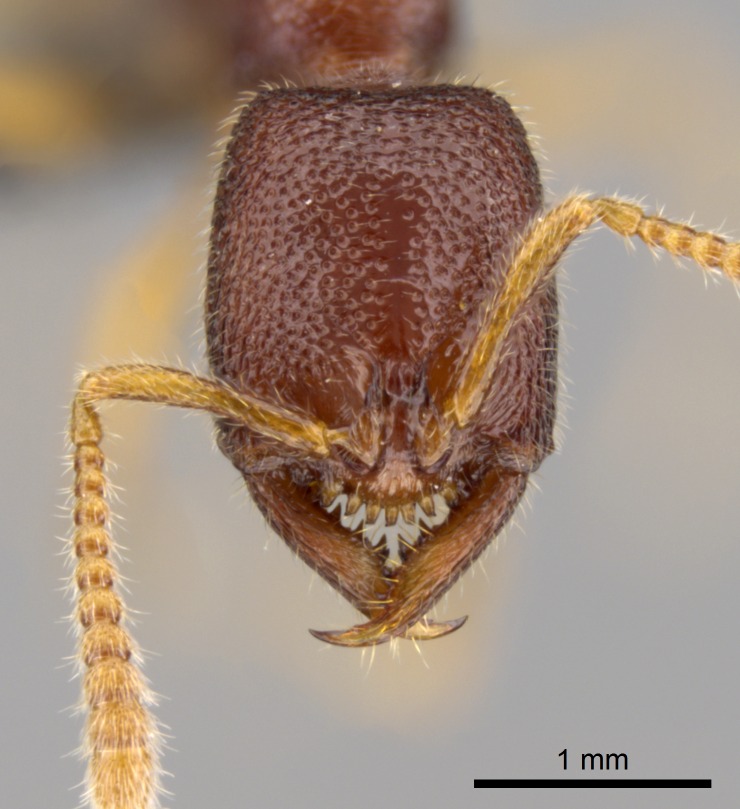
Fullface view.

**Figure 88b.**
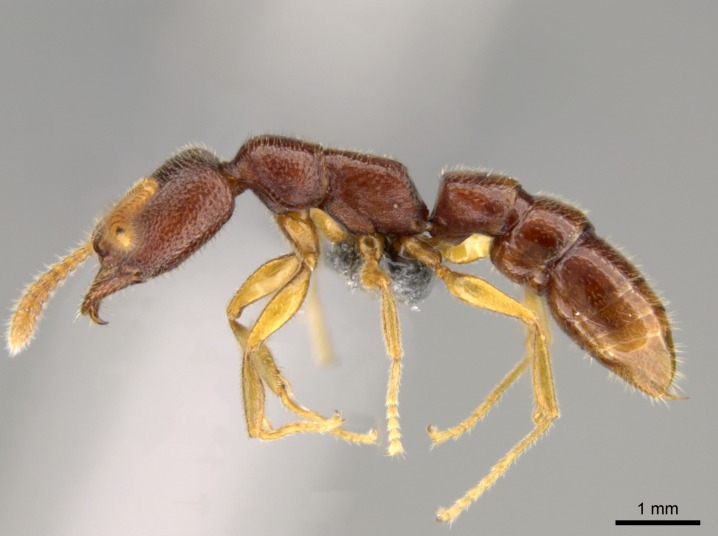
Lateral view.

**Figure 88c.**
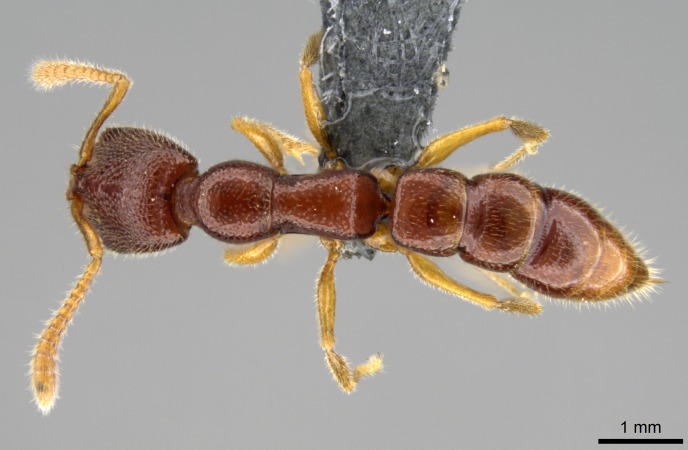
Dorsal view.

**Figure 89a.**
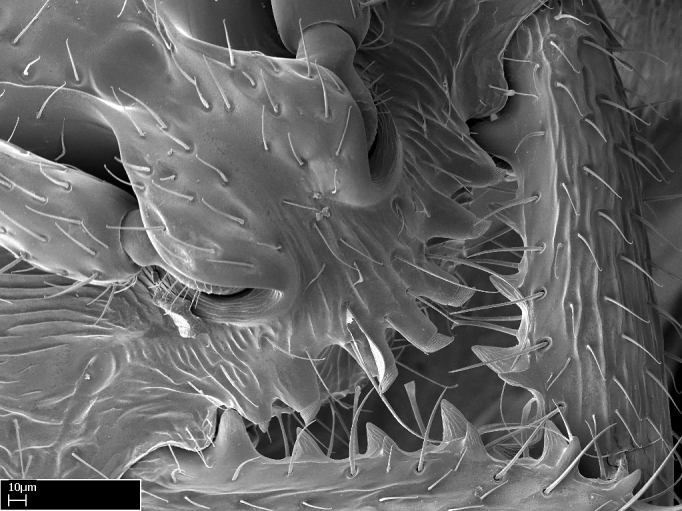
Partial dorsal view of the mandibles and anterior part of the head (CASENT0022146).

**Figure 89b.**
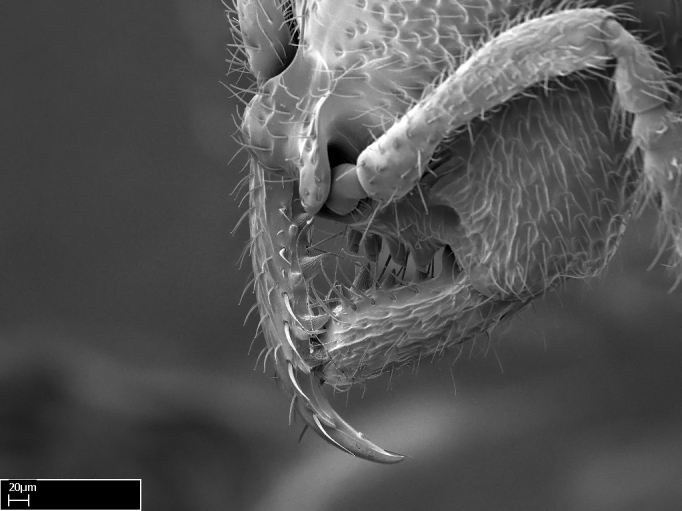
Dorsolateral view of the mandibles and anterior part of the head (CASENT0022146).

**Figure 89c.**
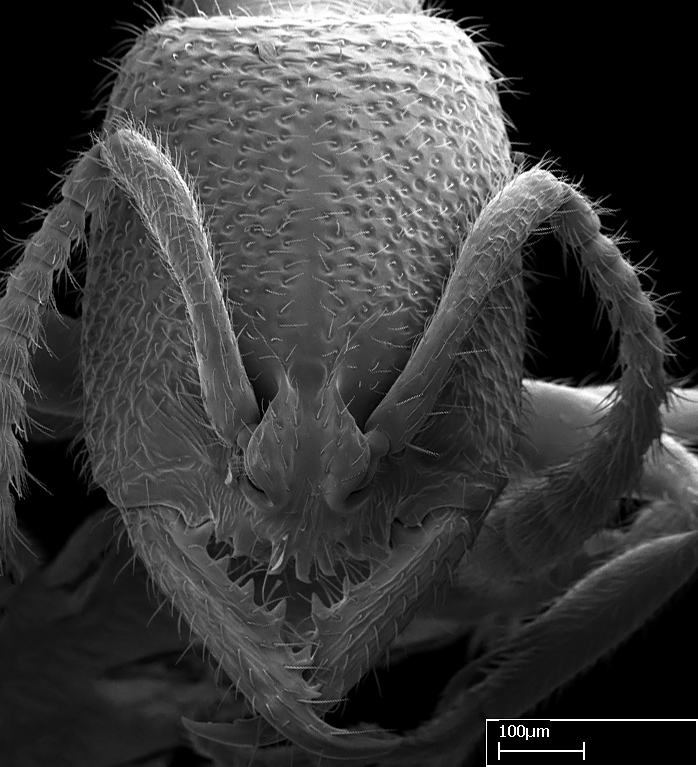
Fullface view (CASENT0022146).

**Figure 89d.**
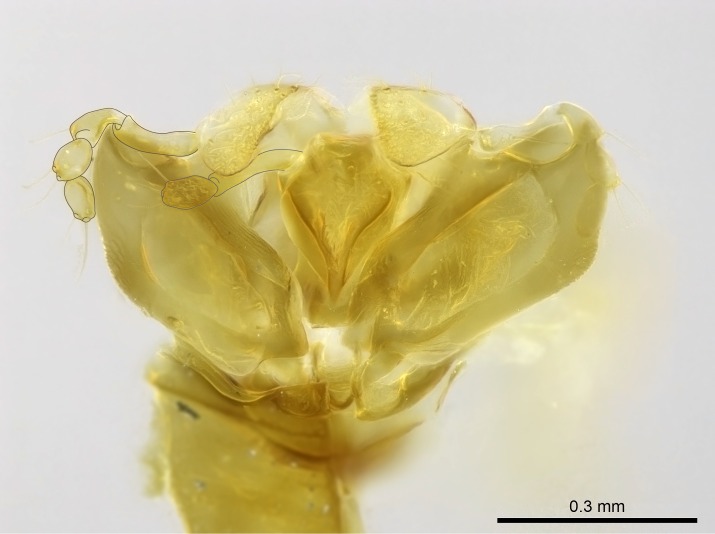
Mouthparts, ventral view (CASENT0017557). Right maxillary and labial palps are outlined in grey and darkened to enhance visibility. Slide by F. A. Esteves.

**Figure 90a.**
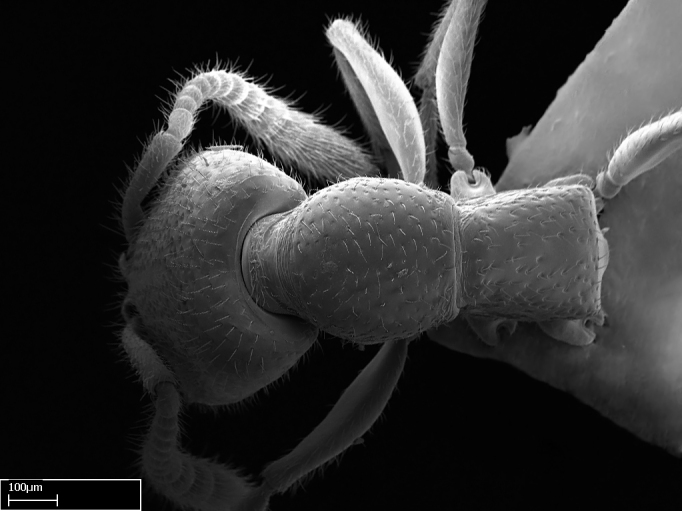
Dorsal view.

**Figure 90b.**
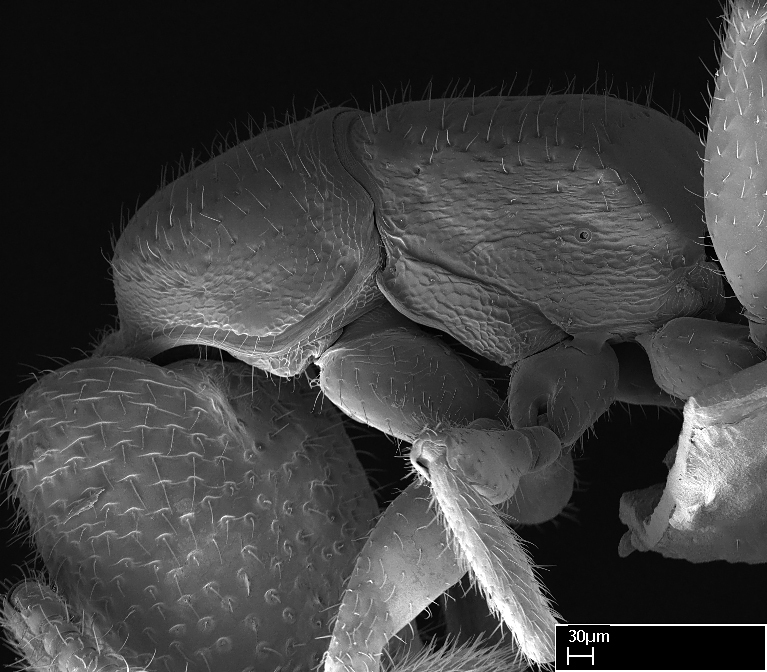
Lateral view.

**Figure 91a.**
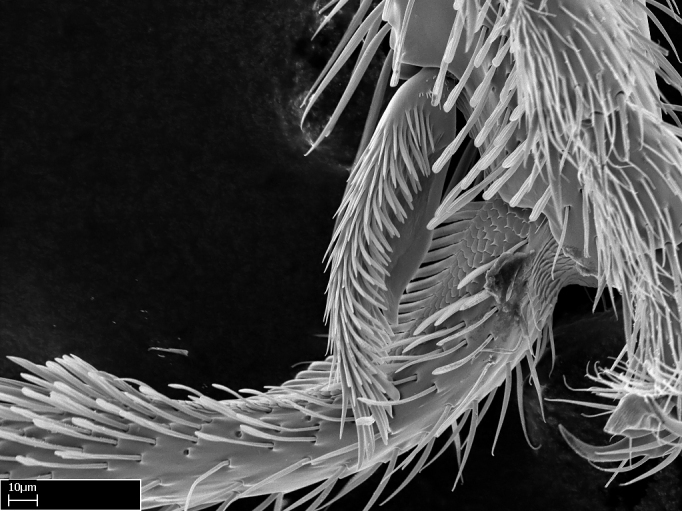
Foreleg (CASENT0017556), anterior face: apical portion of the tibia, its associated calcar of strigil, and the basitarsus.

**Figure 91b.**
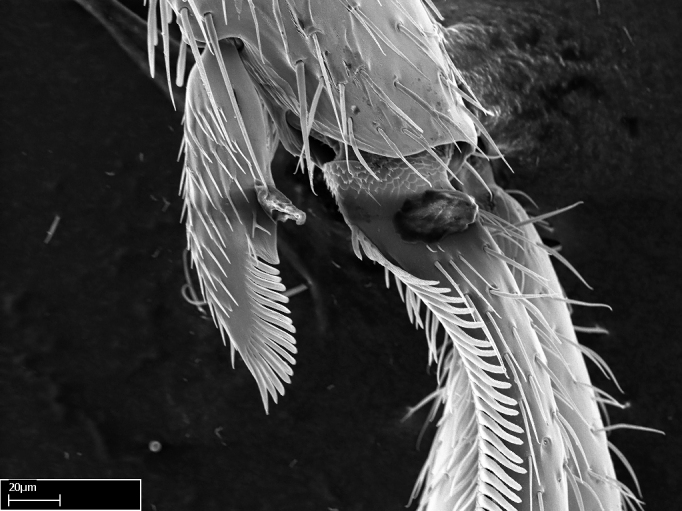
Foreleg (CASENT0017556), posterior face: apical portion of tibia, its associated calcar of strigil, and basal portion of the basitarsus.

**Figure 91c.**
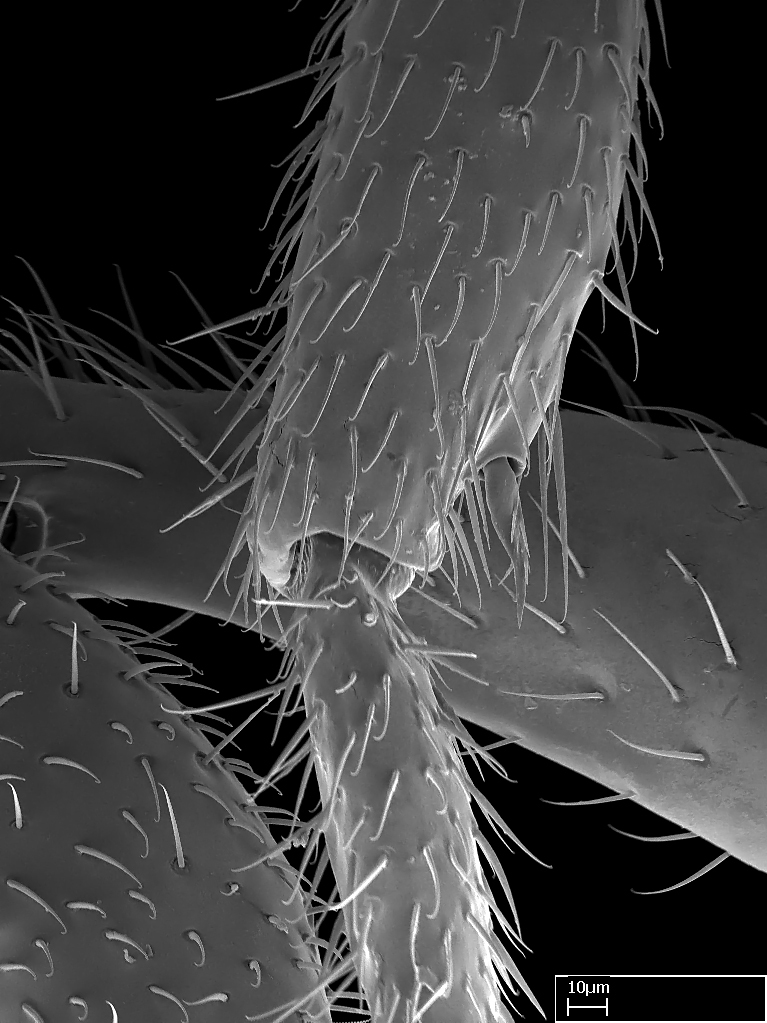
Midleg (CASENT0022146), posterior face: apical portion of the tibia, and its associated spur; basal portion of the basitarsus.

**Figure 91d.**
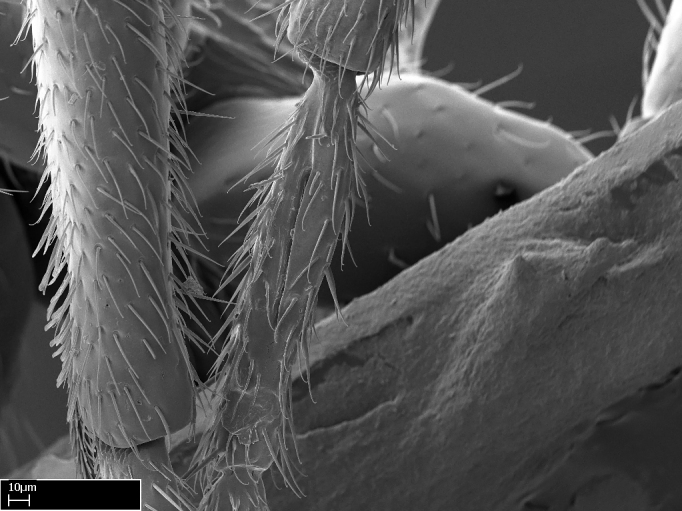
Midleg (CASENT0022146), anterior face: basitarsus with longitudinal sulcus.

**Figure 92a.**
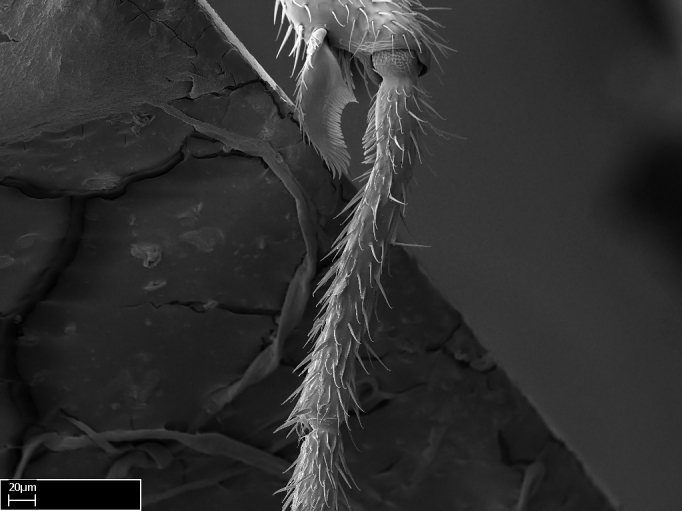
Hindleg, anterior face: apical portion of the tibia, its associated spurs, and the basitarsus.

**Figure 92b.**
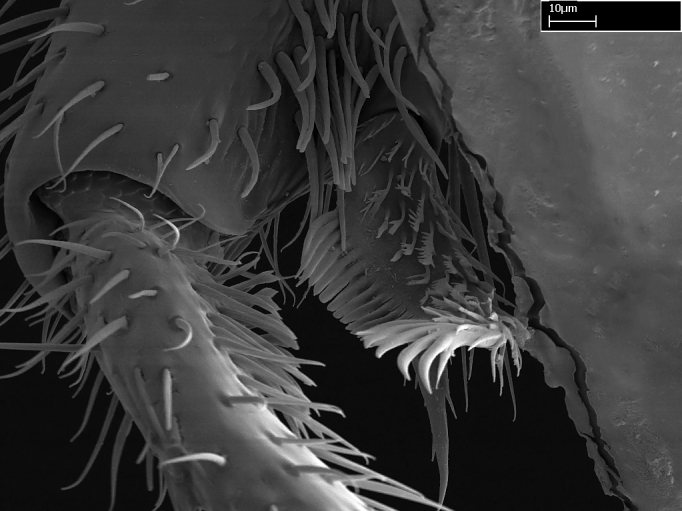
Hindleg, posterior face: apical portion of the tibia, its associated spur, and basal portion of the basitarsus.

**Figure 93a.**
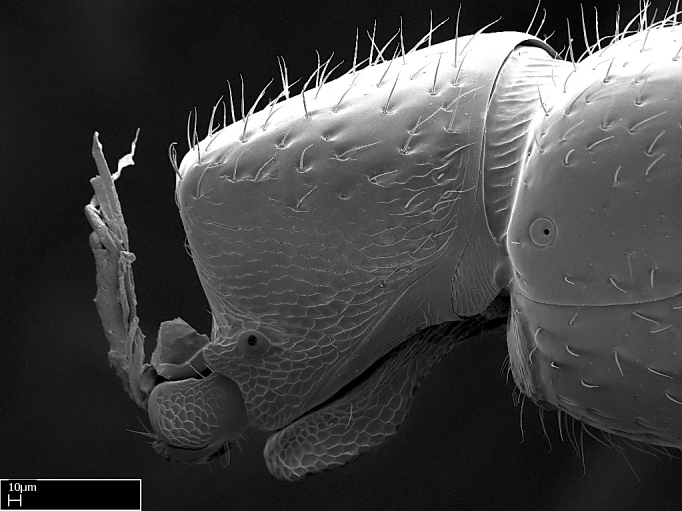
Petiole, lateral view.

**Figure 93b.**
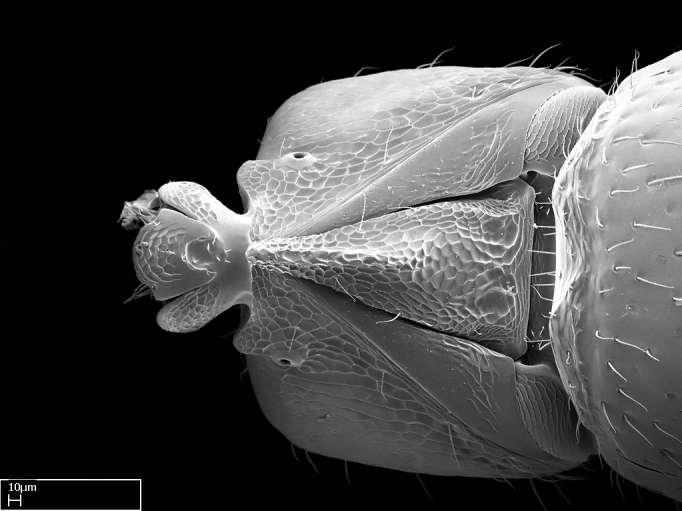
Petiole, ventral view.

**Figure 93c.**
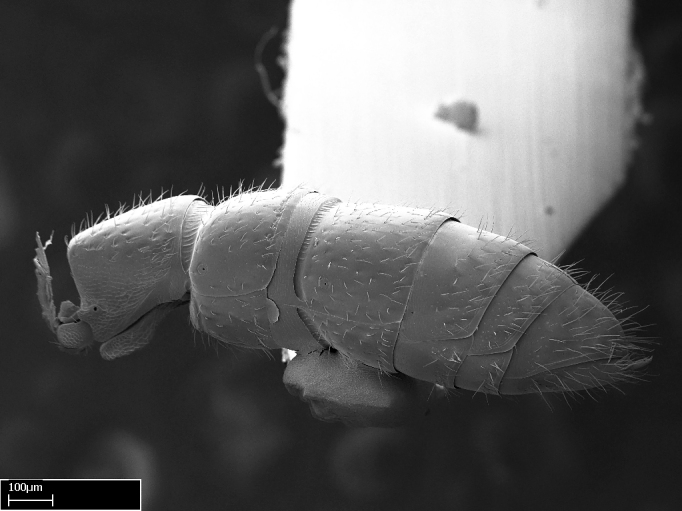
Petiole and gaster, lateral view.

**Figure 93d.**
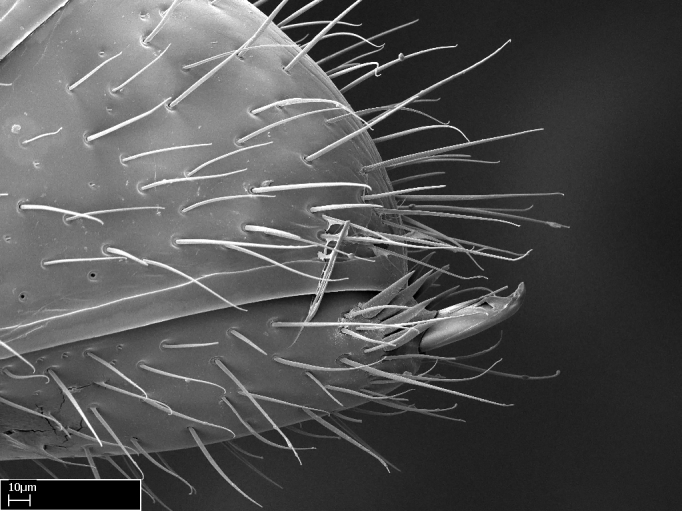
Abdominal segment VII and stinger; lateral view.

**Figure 95a.**
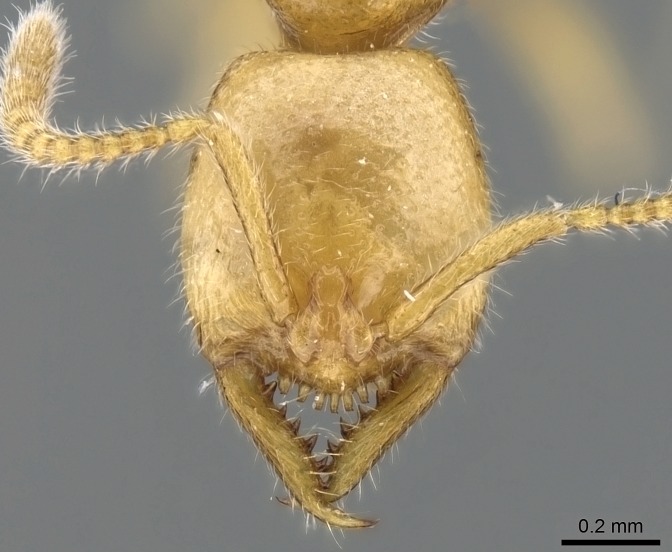
Fullface view.

**Figure 95b.**
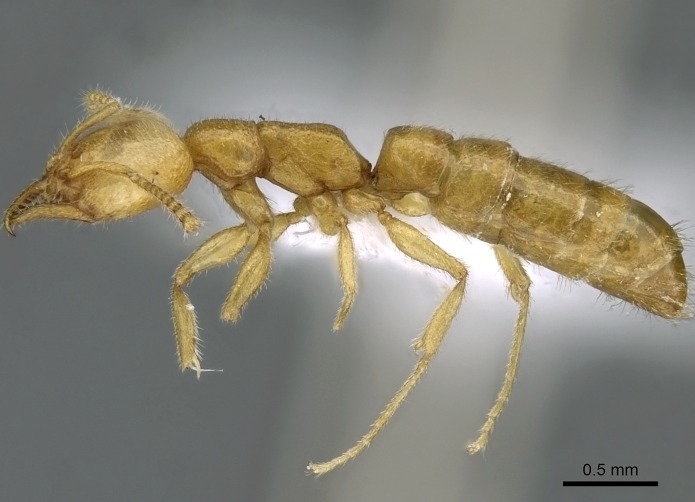
Lateral view.

**Figure 95c.**
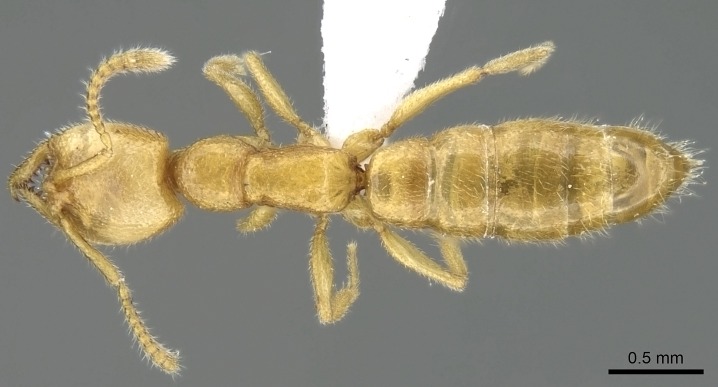
Dorsal view.

**Figure 96a.**
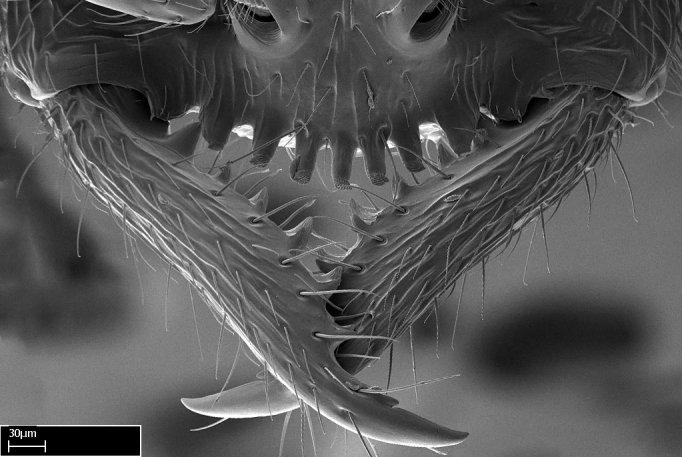
Dorsal view of the mandibles and anterior part of the head.

**Figure 96b.**
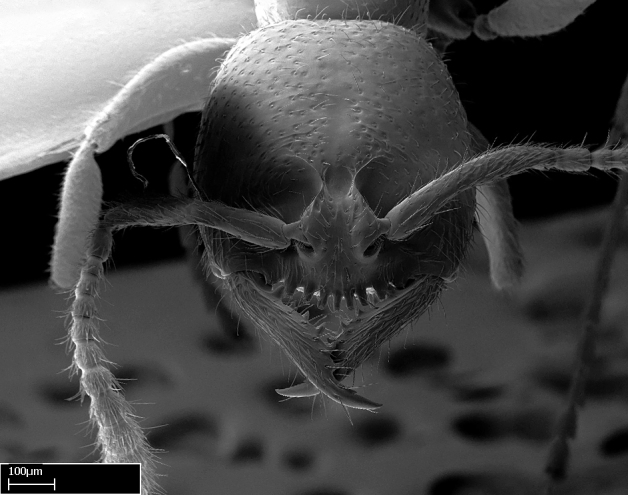
Fullface view.

**Figure 96c.**
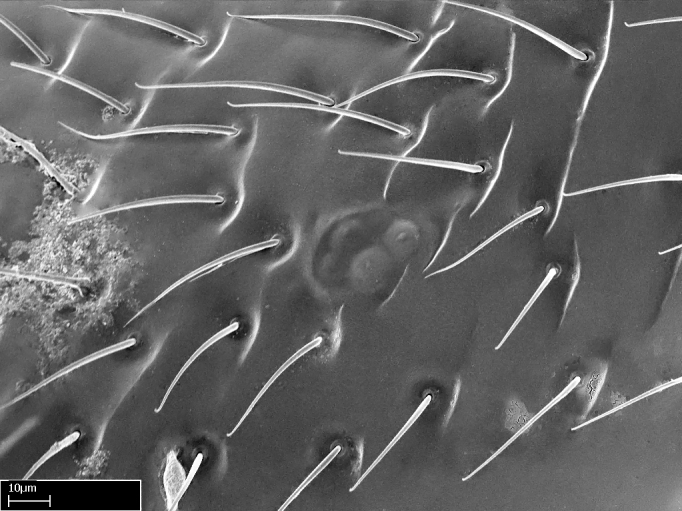
Close-up of the eyes, dorsolateral view.

**Figure 97a.**
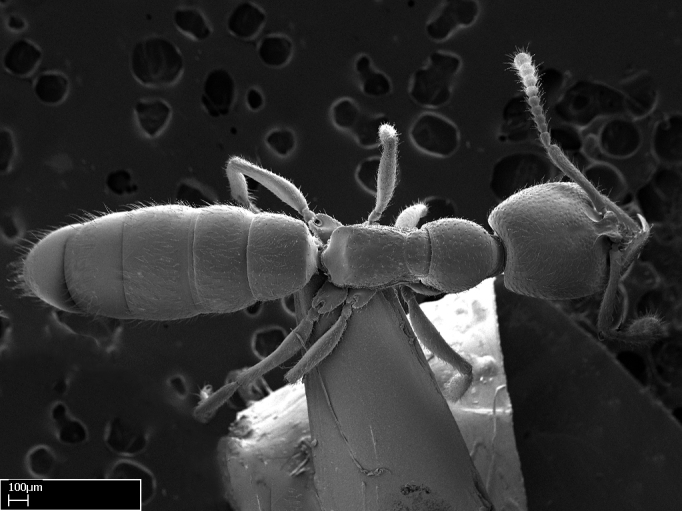
Dorsal view of the body.

**Figure 97b.**
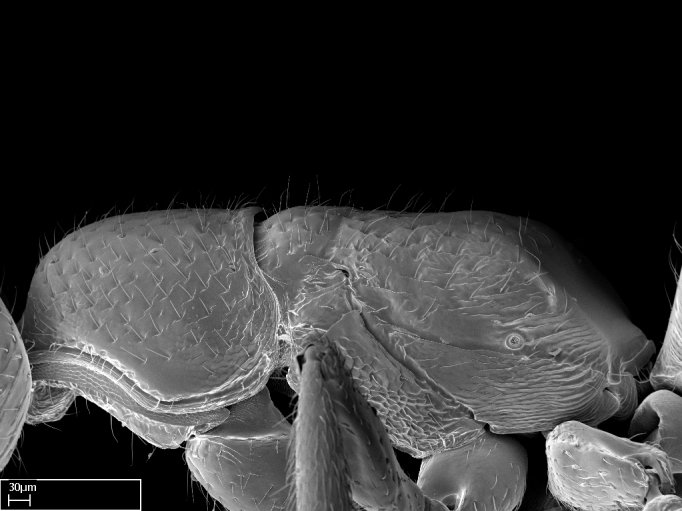
Mesosoma, lateral view.

**Figure 97c.**
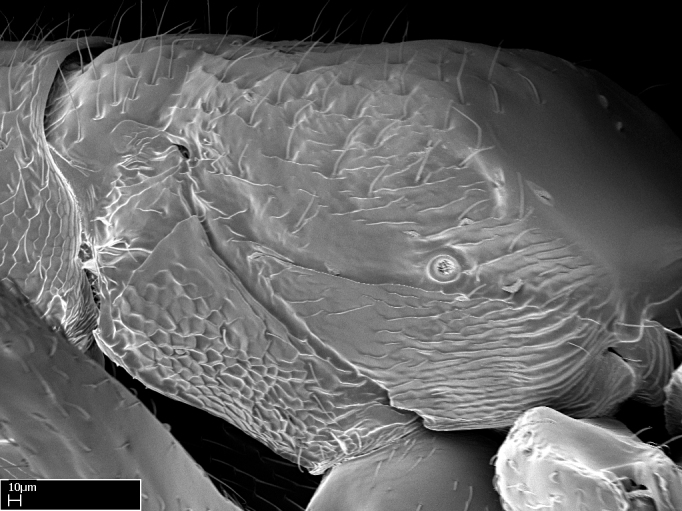
Apical portion of the pronotum, and remainder of mesosoma, in lateral view.

**Figure 98a.**
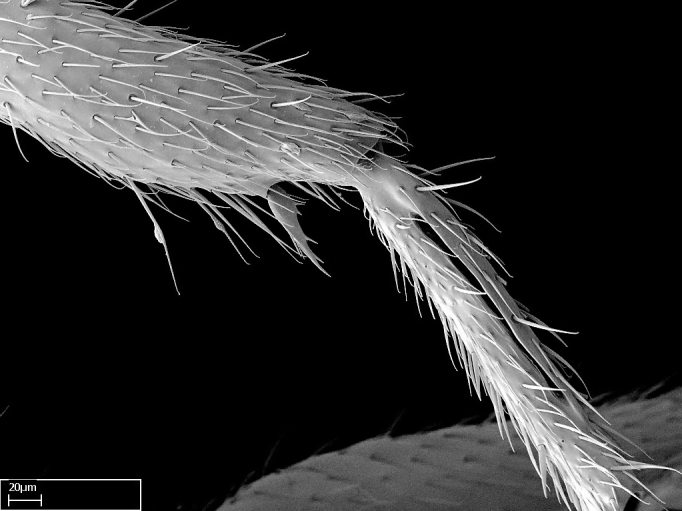
Midleg, anterior view: apical portion of the tibia, with associated spur, and basitarsus.

**Figure 98b.**
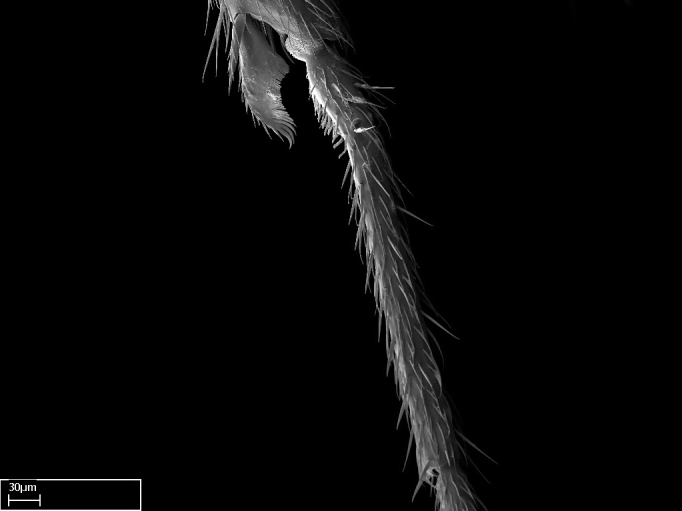
Hindleg, anterior view: apical portion of the tibia, with associated spurs, and basitarsus.

**Figure 98c.**
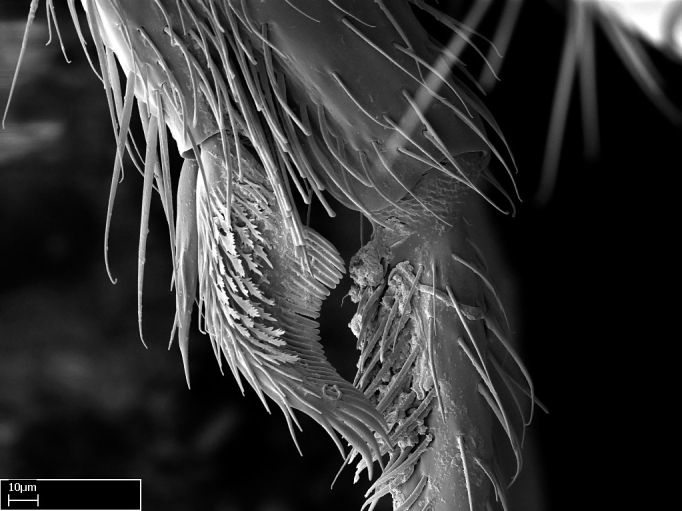
Hindleg, posterior view: apical portion of the tibia, with associated spurs, and basal portion of the basitarsus.

**Figure 99a.**
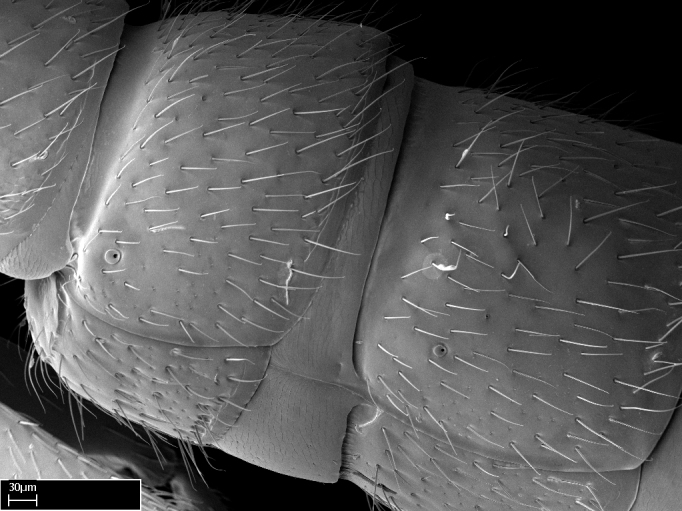
From left to right: apical portion of the petiole, abdominal segment III, and abdominal segment IV, lateral view.

**Figure 99b.**
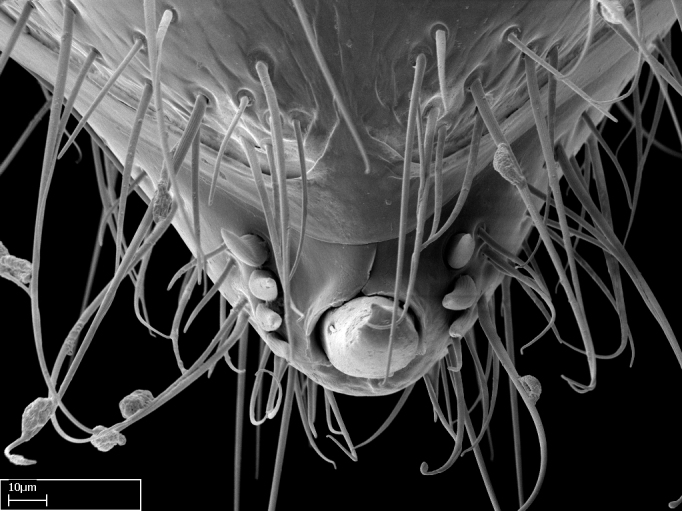
Apical portion of the abdominal segment VII, dorsal view.

**Figure 101a.**
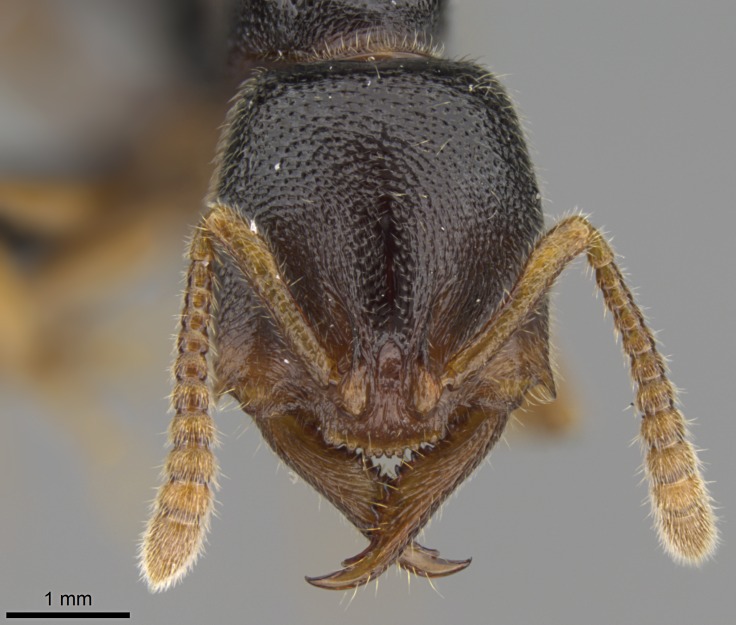
Fullface view.

**Figure 101b.**
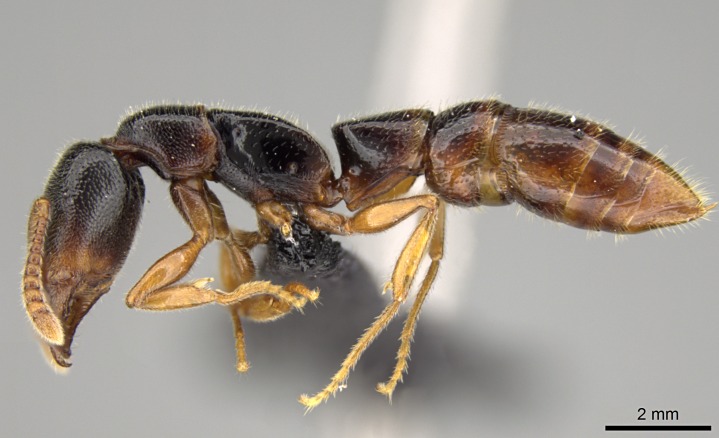
Lateral view.

**Figure 101c.**
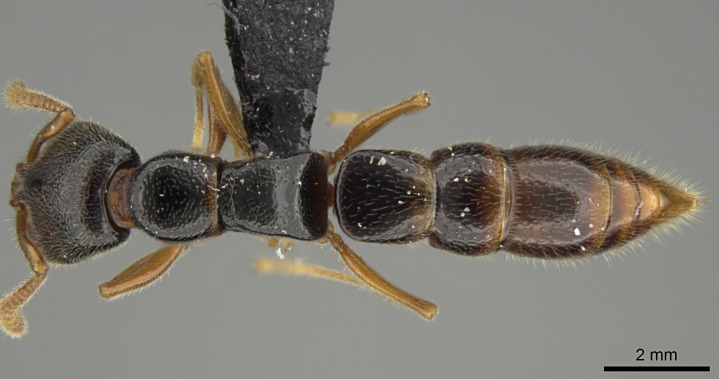
Dorsal view.

**Figure 102a.**
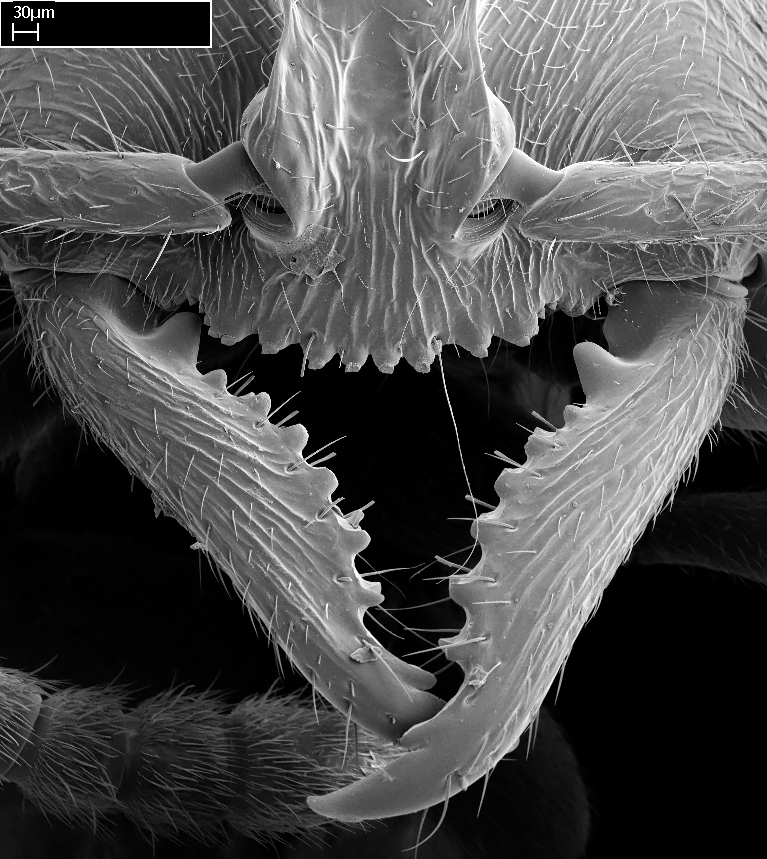
Dorsal view of the mandibles and anterior part of the head (CASENT0074309).

**Figure 102b.**
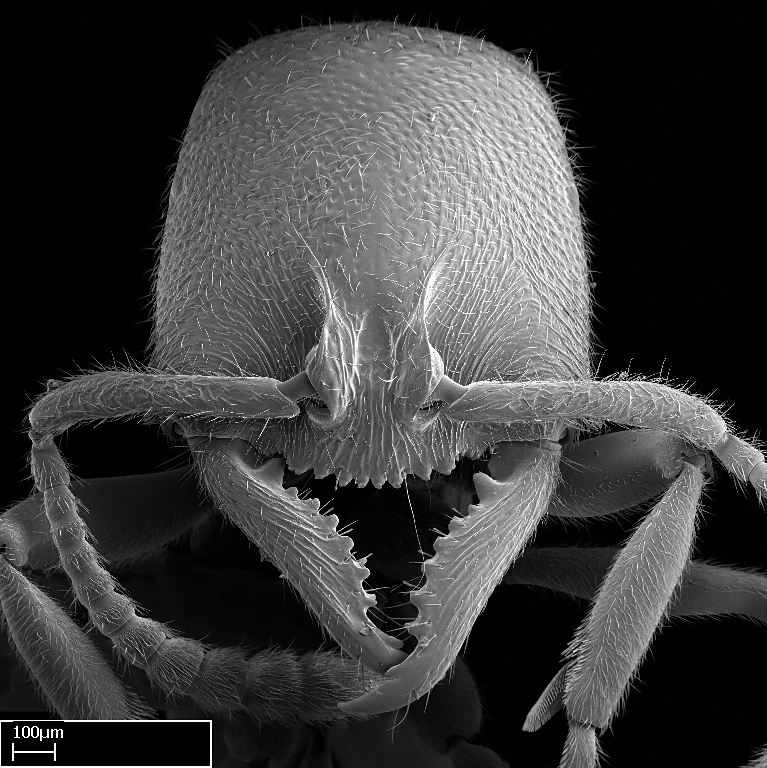
Fullface view (CASENT0074309).

**Figure 102c.**
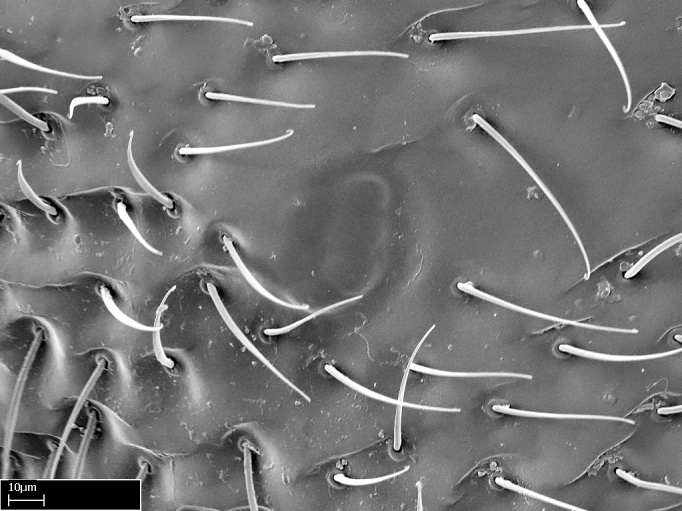
Close-up of the eyes, dorsolateral view (CASENT0074309).

**Figure 102d.**
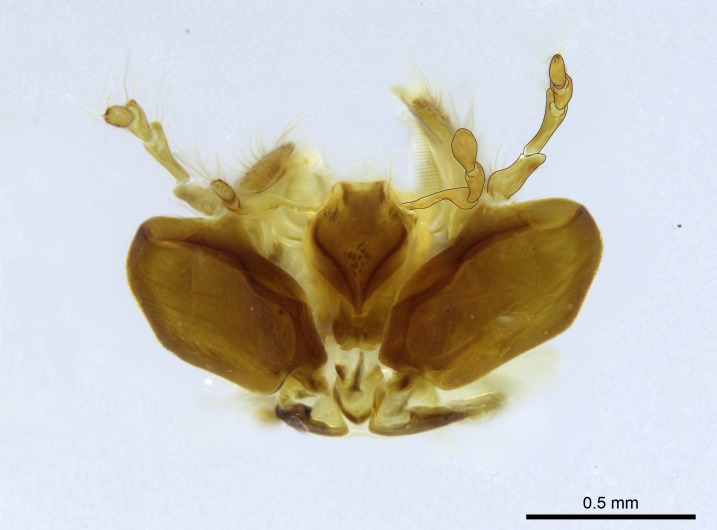
Mouthparts, ventral view [HJR102(41)]. Left maxillary and labial palps are outlined in black and darkened to enhance visibility. Slide by F. A. Esteves

**Figure 103a.**
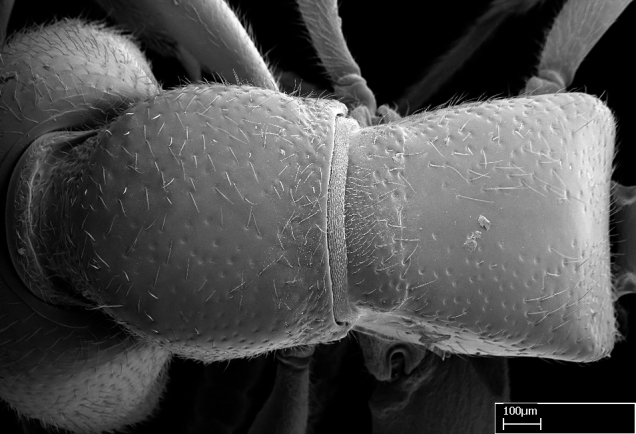
Dorsal view.

**Figure 103b.**
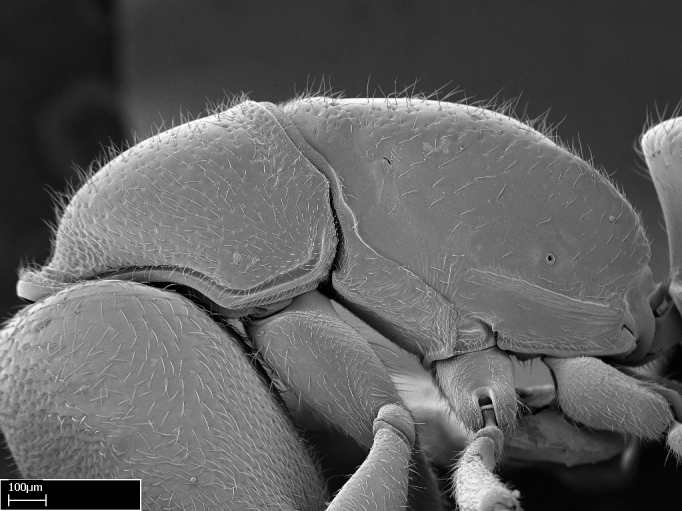
Lateral view.

**Figure 104a.**
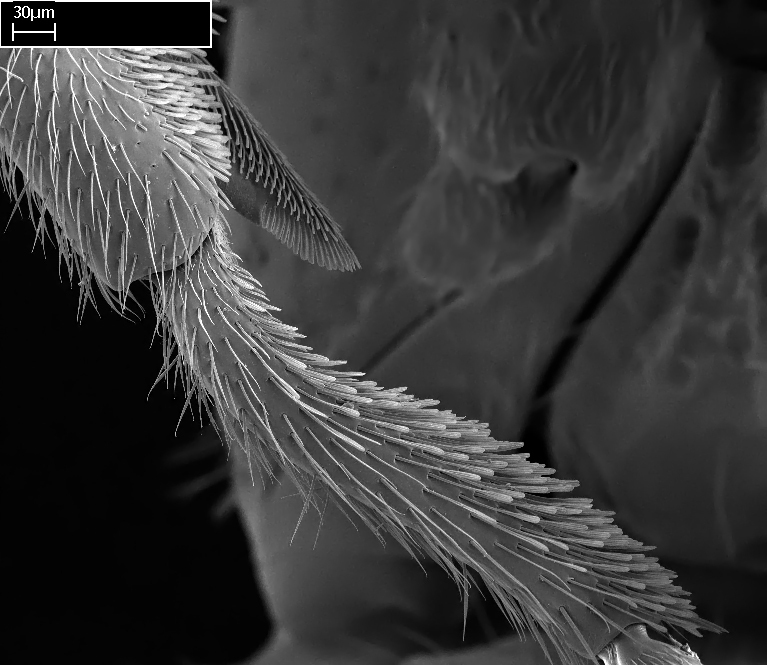
Foreleg, anterior face: apical portion of the tibia, its associated calcar of strigil, and basal portion of the basitarsus

**Figure 104b.**
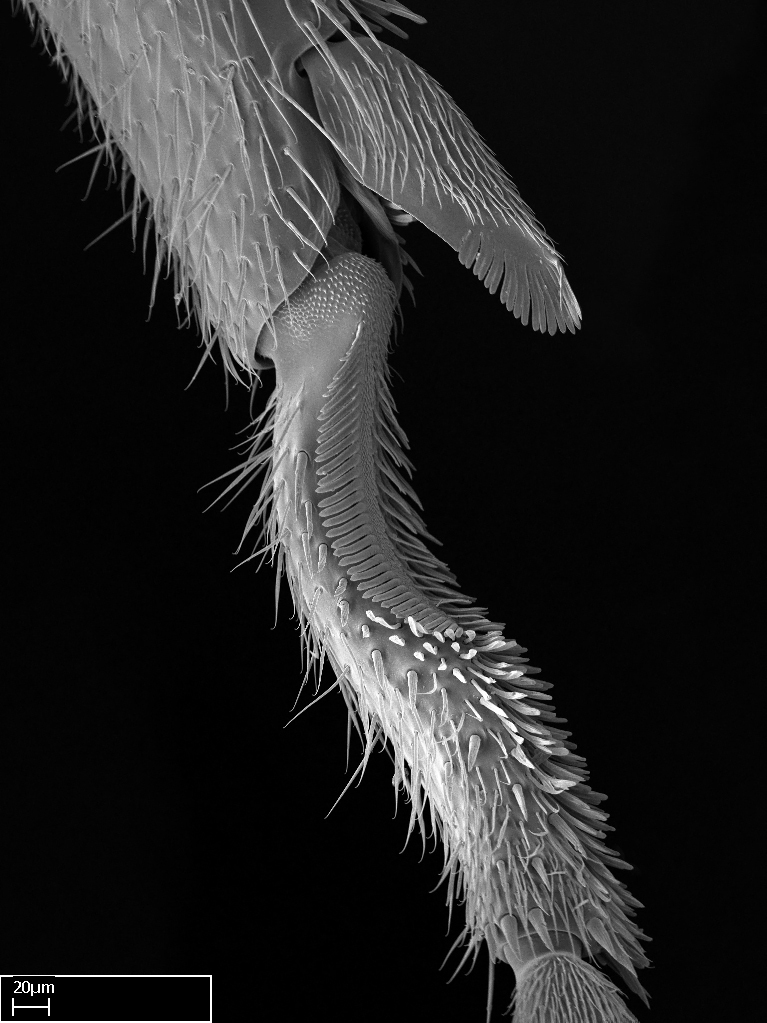
Foreleg, postero-inner face: apical portion of tibia, its associated calcar of strigil, and basitarsus.

**Figure 104c.**
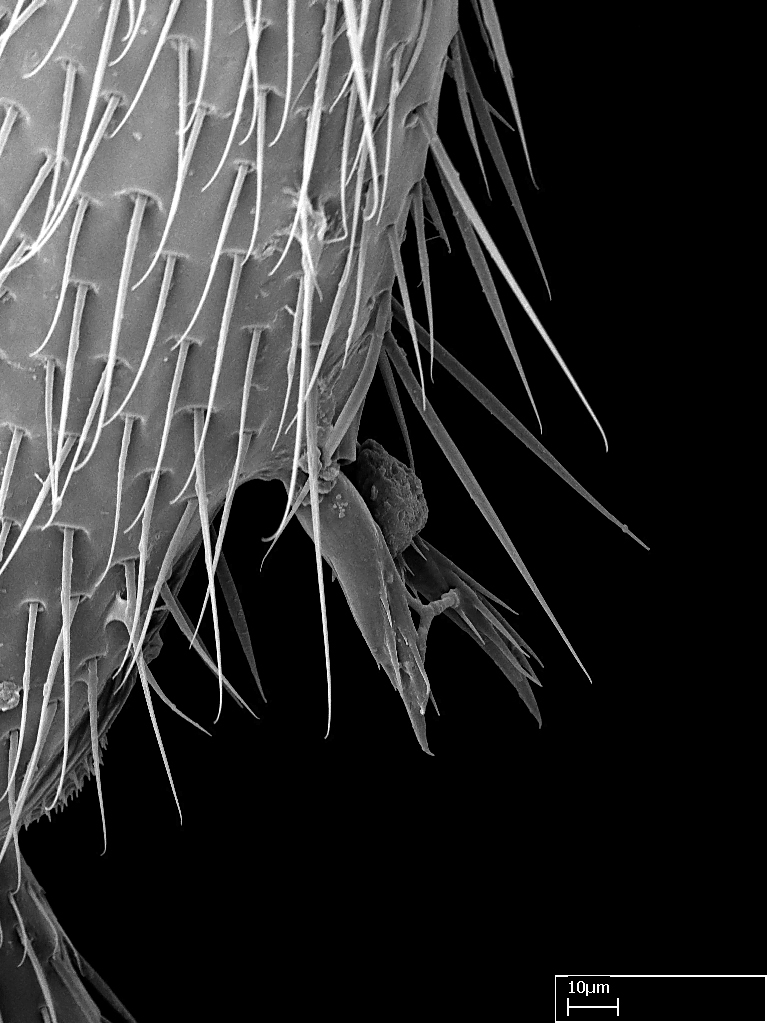
Midleg, anterior face: apical portion of the tibia, and its associated spurs.

**Figure 104d.**
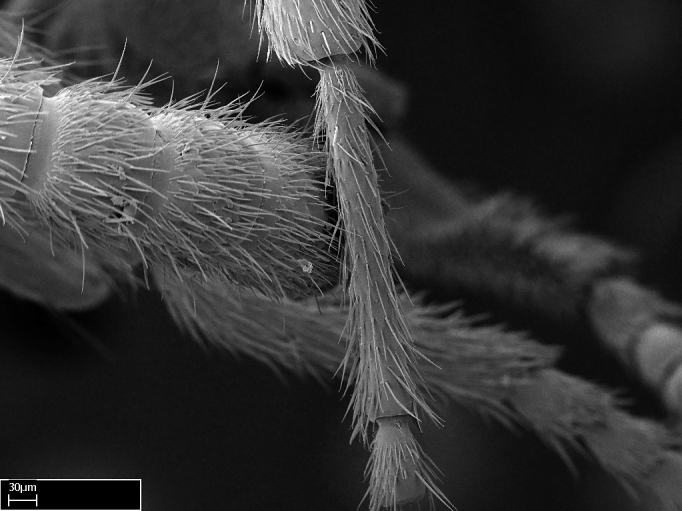
Midleg, anterodorsal view: apical portion of the tibia, and basitarsus.

**Figure 105a.**
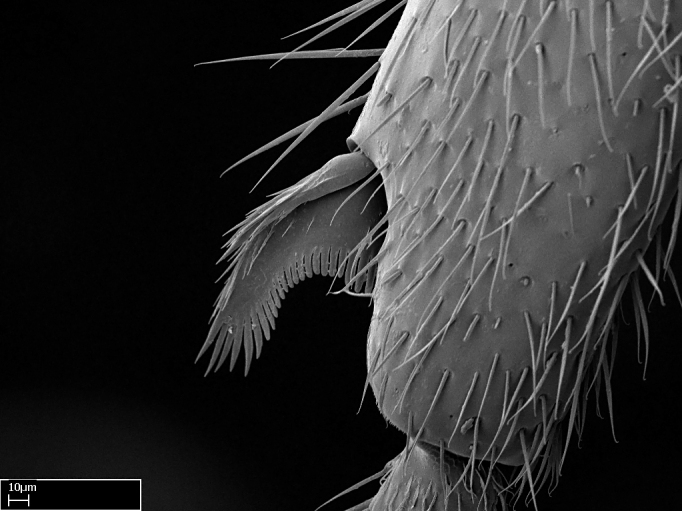
Hindleg, anterior face: apical portion of the tibia, and its associated spurs.

**Figure 105b.**
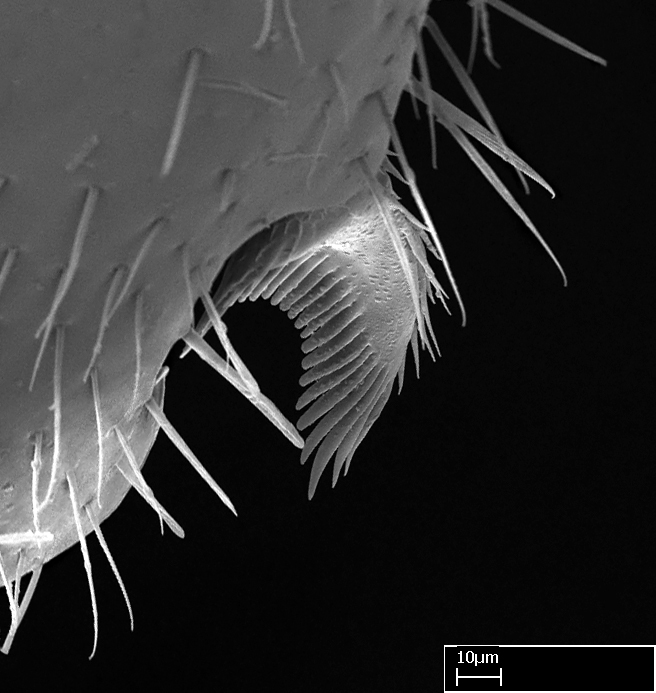
Hindleg, posterior face: apical portion of the tibia, and its associated posterior spur.

**Figure 105c.**
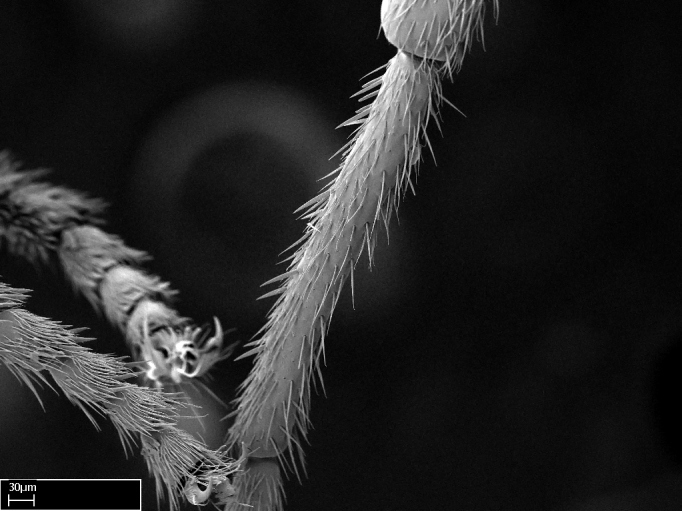
Hindleg, anterior face: apical portion of the tibia, and basitarsus.

**Figure 106a.**
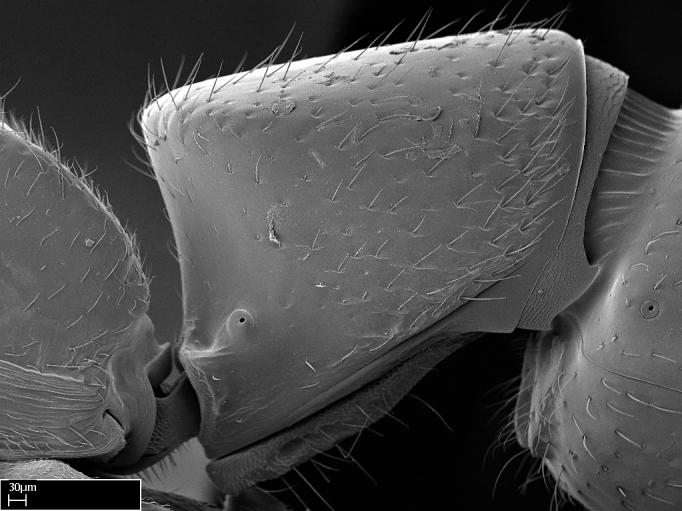
Petiole, lateral view.

**Figure 106b.**
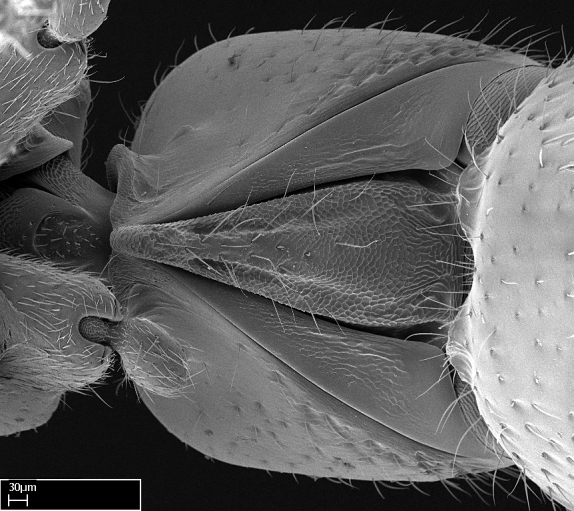
Petiole, ventral view

**Figure 106c.**
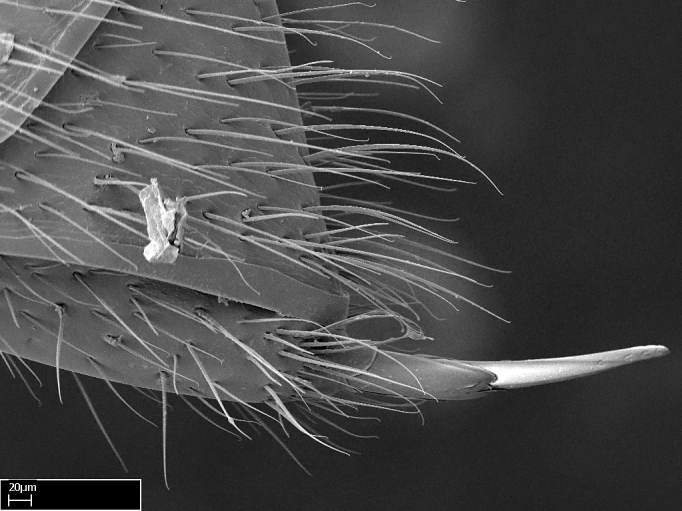
Abdominal segment VII and stinger, lateral view

**Figure 108a.**
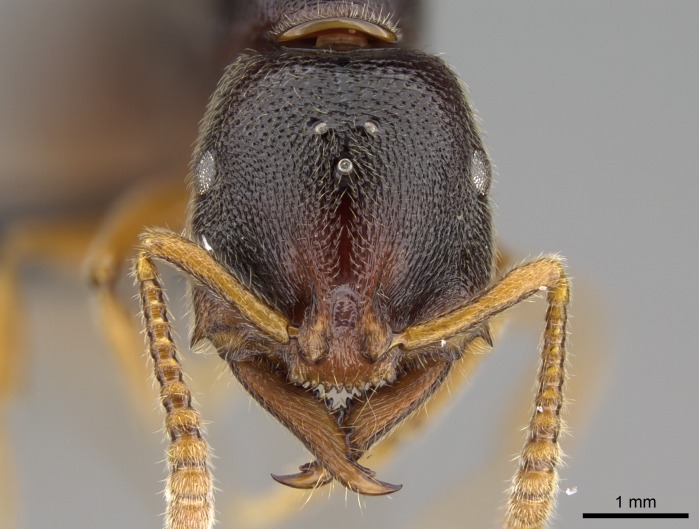
Fullface view.

**Figure 108b.**
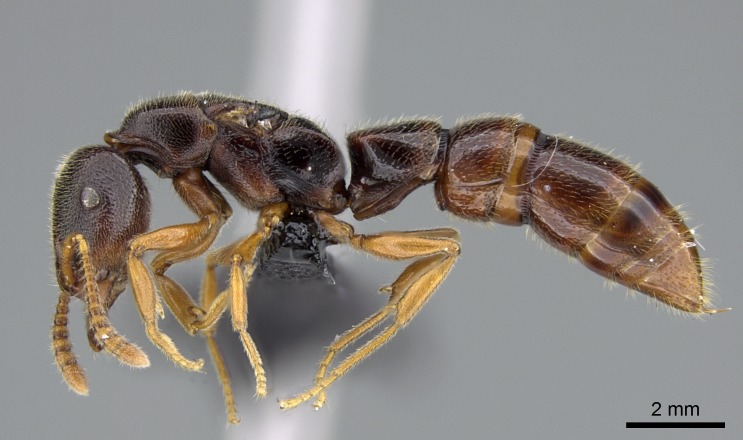
Lateral view. Wings were removed for better illustration.

**Figure 108c.**
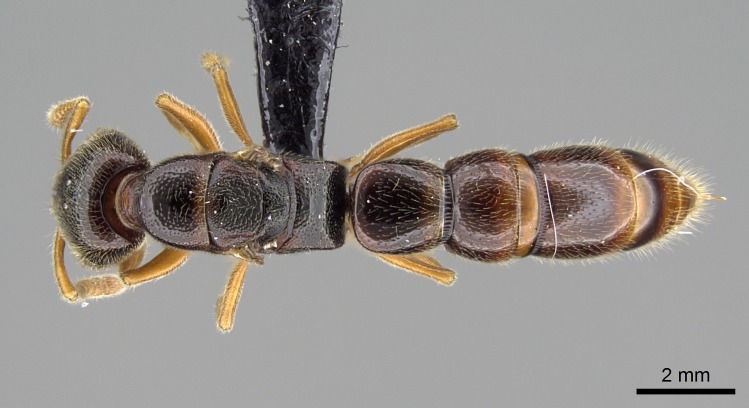
Dorsal view. Wings were removed for better illustration.

**Figure 109a.**
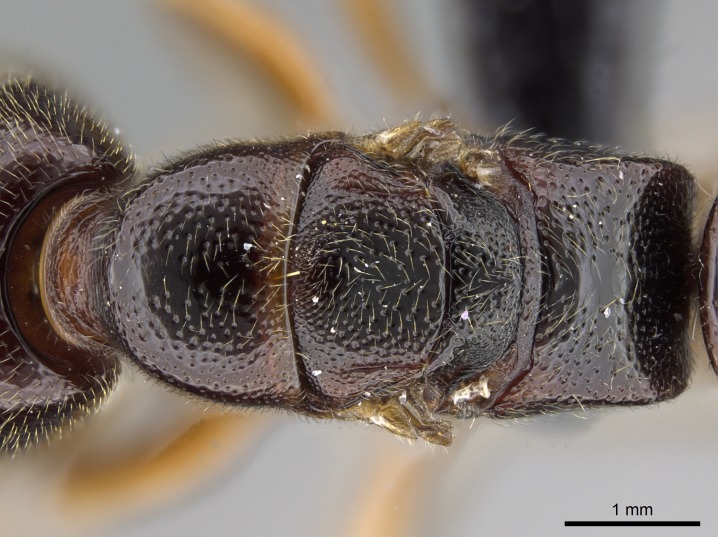
Mesosoma, dorsal view. Wings were removed for better illustration.

**Figure 109b.**
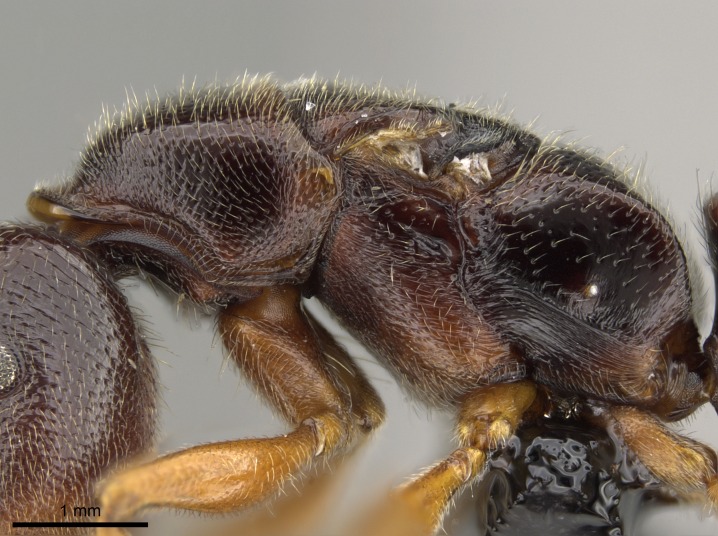
Mesosoma, lateral view. Wings were removed for better illustration.

**Figure 109c.**
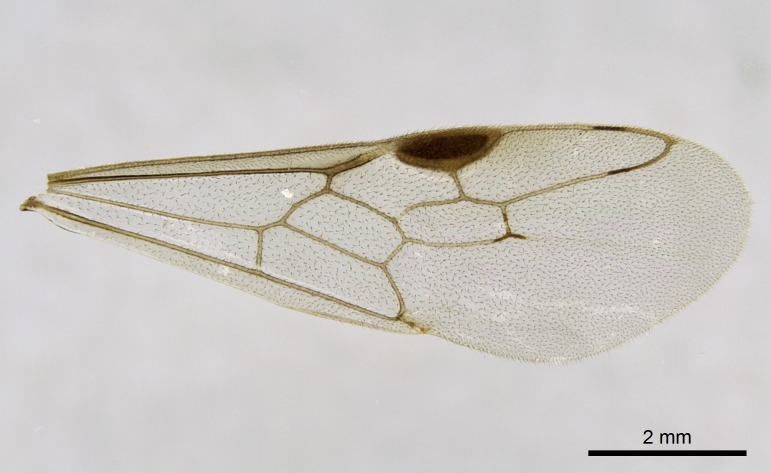
Right forewing.

**Figure 109d.**
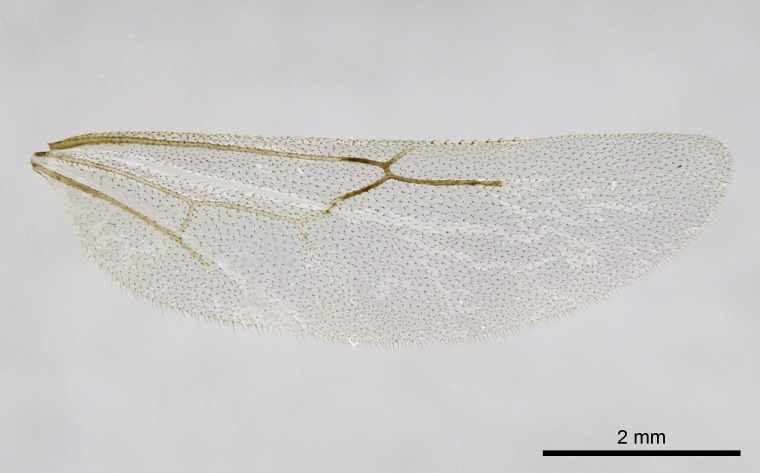
Right hindwing.

**Figure 110a.**
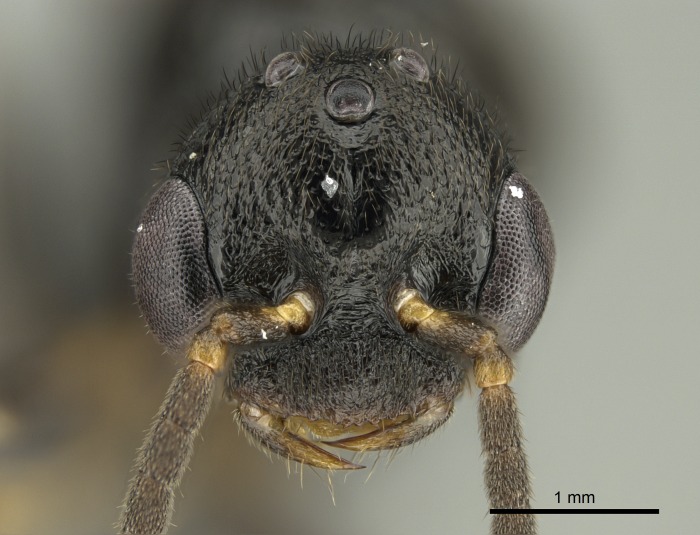
Fullface view.

**Figure 110b.**
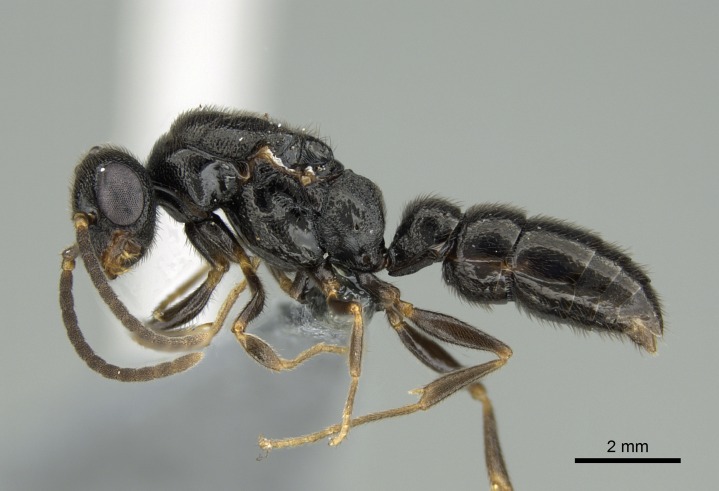
Lateral view. Wings were removed for better illustration.

**Figure 110c.**
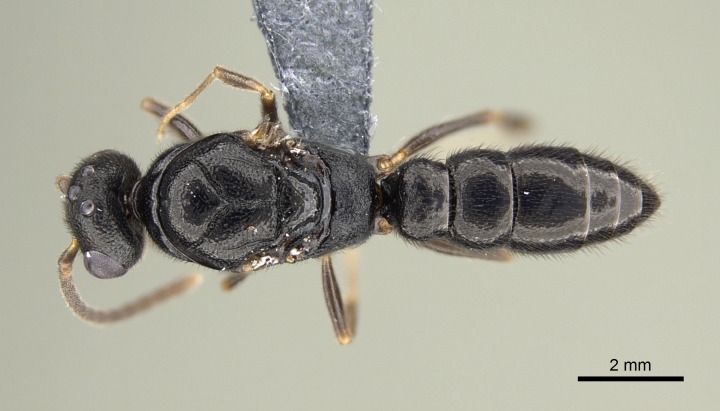
Dorsal view. Wings were removed for better illustration.

**Figure 111a.**
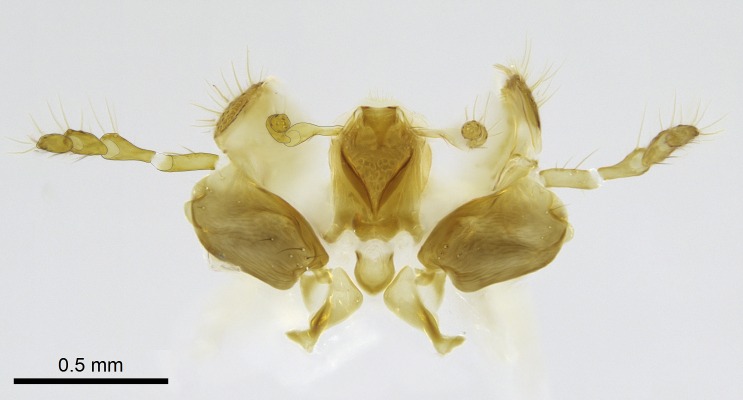
Mouthparts (CASENT0723249), ventral view. Right maxillary and labial palps are outlined in gray and darkened to enhance visibility. Slide by F. A. Esteves.

**Figure 111b.**
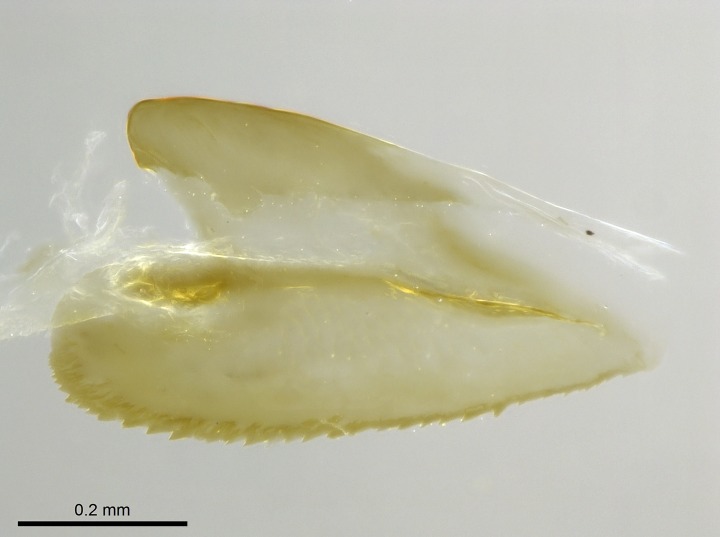
Aedeagus (CASENT0723249), lateral view. Slide by F. A. Esteves.

**Figure 111c.**
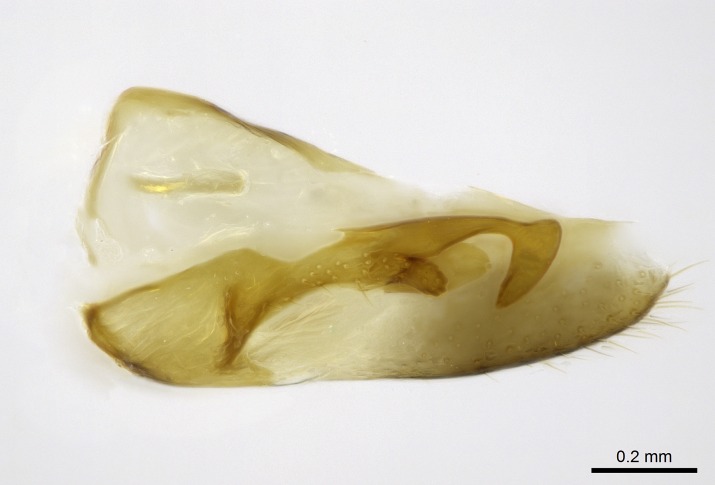
Longitudinal section of the genital capsule (CASENT0723251); inner face, lateral view. The basal ring was removed from the specimen. Slide by F. A. Esteves.

**Figure 111d.**
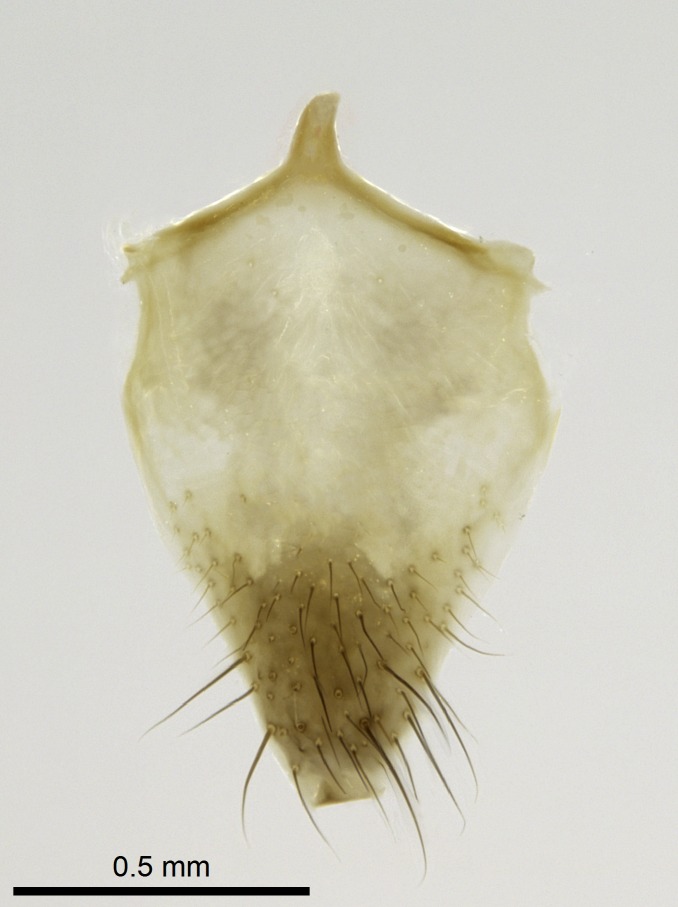
Abdominal sternun IX (CASENT0723249), ventral view. Slide by F. A. Esteves.

**Figure 112a.**
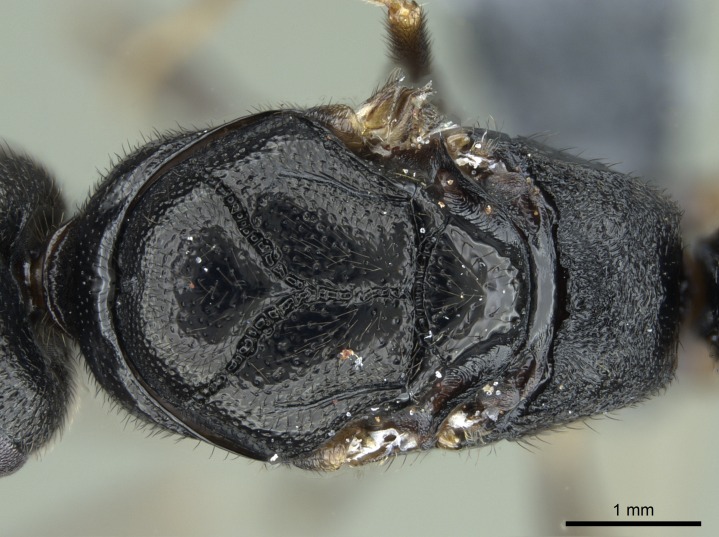
Mesosoma; dorsal view. Wings were removed for better illustration.

**Figure 112b.**
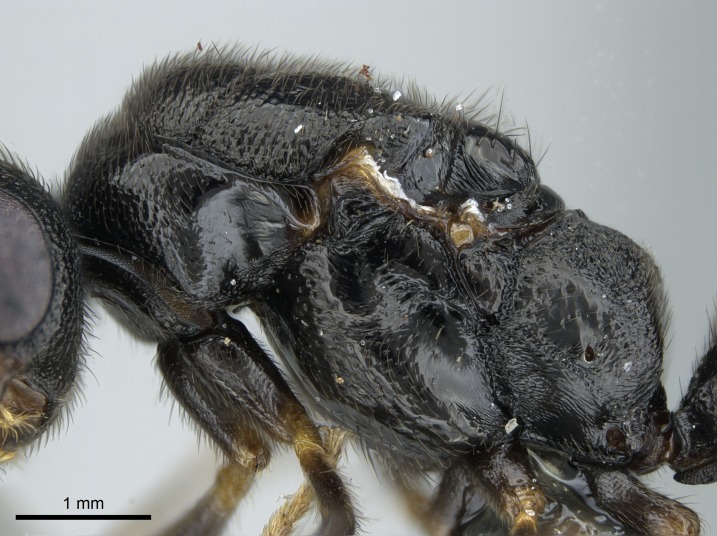
Mesosoma; lateral view. Wings were removed for better illustration.

**Figure 112c.**
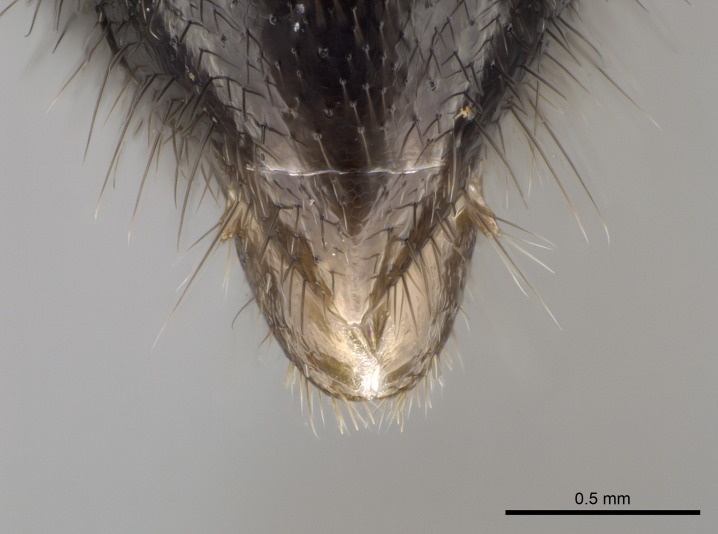
Apex of the gaster; dorsal view.

**Figure 113a.**
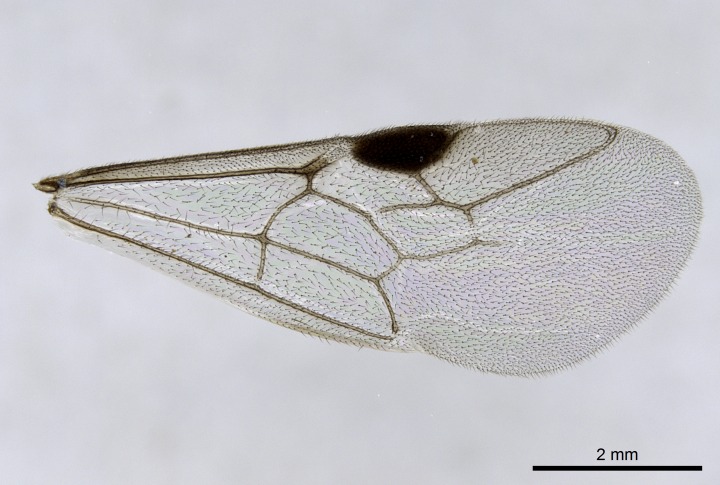
Right forewing.

**Figure 113b.**
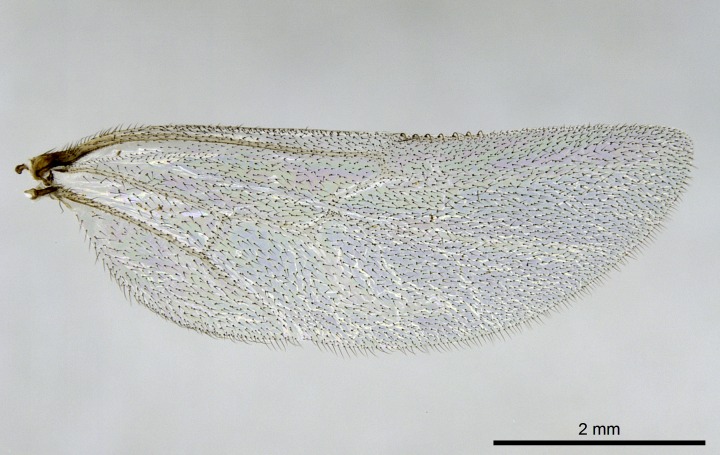
Right hindwing.

**Figure 115a.**
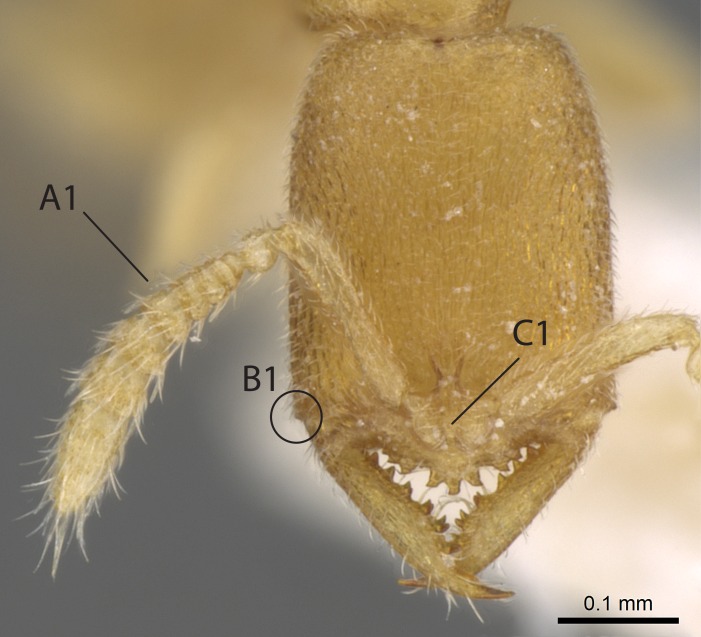
Paratype of *Stigmatomma besucheti* (CASENT0906833); worker; dorsal face of the head. **A1**: antennomeres; **B1**: absence of genal tooth; **C1**: clypeus narrowly inserted between frontal lobes. Image by Michele Esposito; available at AntWeb.org

**Figure 115b.**
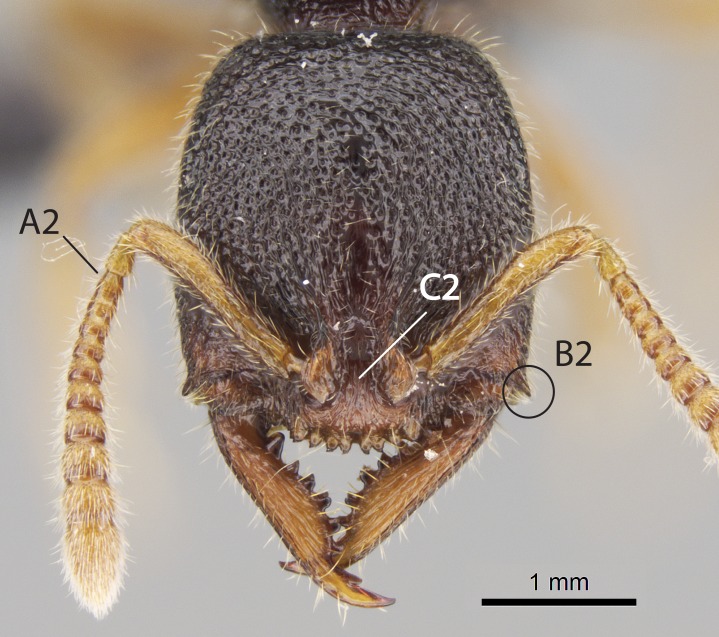
Holotype of *Stigmatomma irayhady* **sp. n.** (CASENT0042899); worker; dorsal face of the head. **A2**: antennomeres; **B2**: presence of genal tooth; **C2**: clypeus broadly inserted between frontal lobes. Image by F. A. Esteves; available at AntWeb.org

**Figure 115c.**
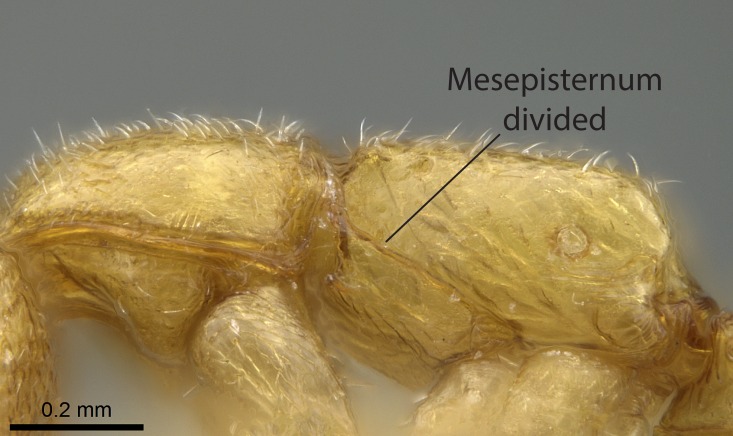
Paratype of *Stigmatomma besucheti* (CASENT0906835); worker; lateral face of the mesosoma. Note that the mesepisternum is divided into anepisternum and katepisternum. Image by F. A. Esteves; available at AntWeb.org

**Figure 115d.**
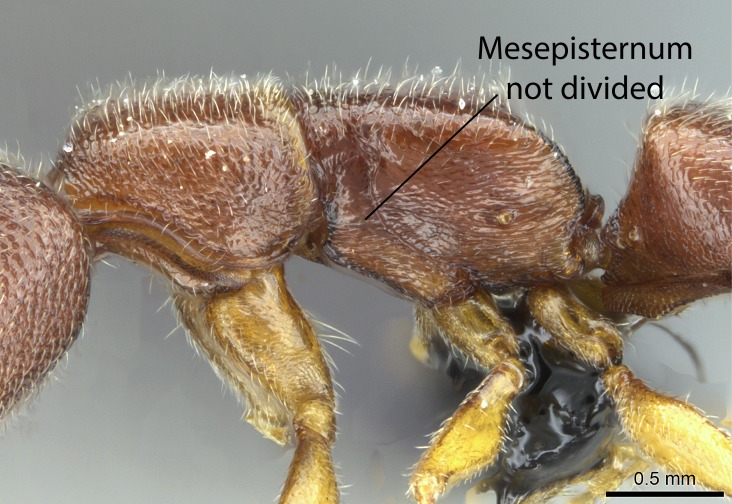
Holotype of *Stigmatomma janovitsika* **sp. n.** (CASENT0161533); worker; lateral face of the mesosoma. Note that the mesepisternum is not divided into anepisternum and katepisternum. Image by F. A. Esteves; available at AntWeb.org

**Figure 115e.**
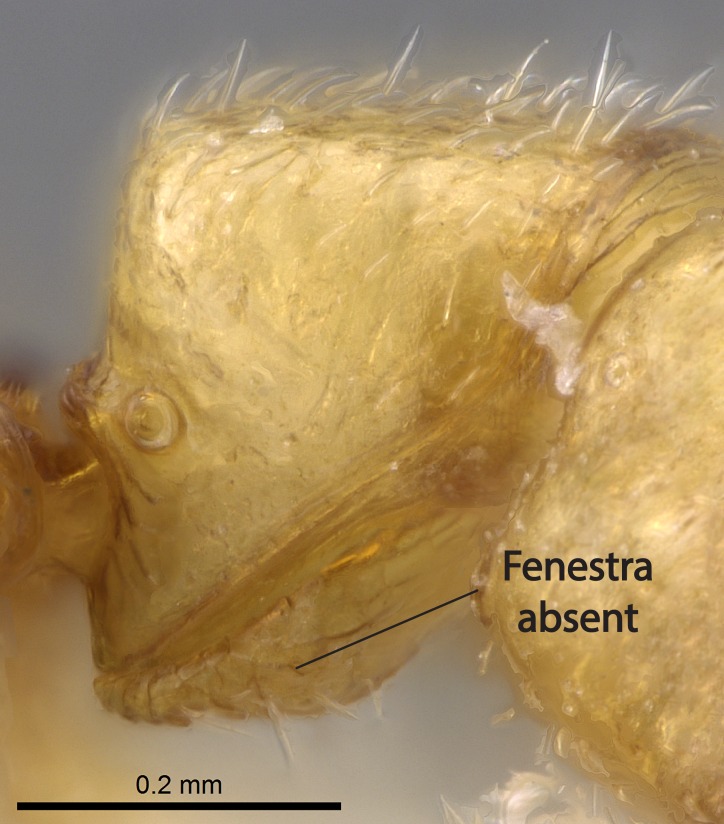
Paratype of *Stigmatomma besucheti* (CASENT0906835); worker; lateral face of the petiole. Note the absence of a fenestra on the lateral face of the subpetiolar process. Image by F. A. Esteves ; available at AntWeb.org

**Figure 115f.**
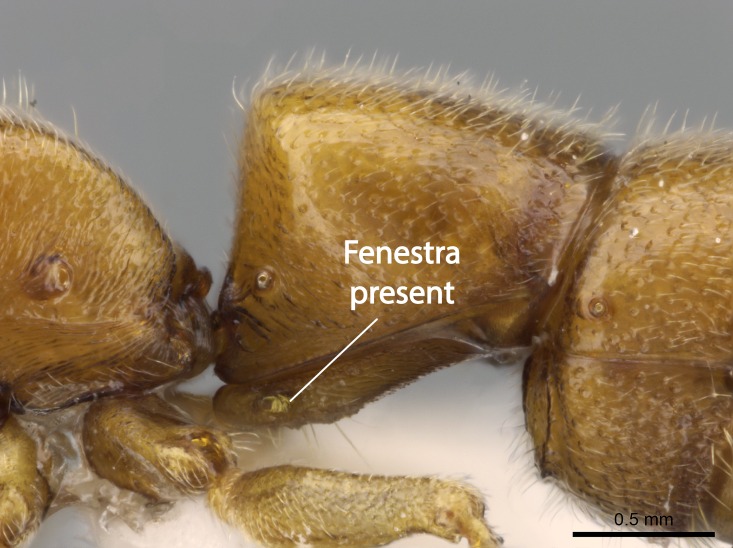
Holotype of *Stigmatomma liebe* **sp. n.** (CASENT0318428); worker; lateral face of the petiole. Note the presence of a fenestra on the lateral face of the subpetiolar process. Image by F. A. Esteves; available at AntWeb.org

**Figure 116a.**
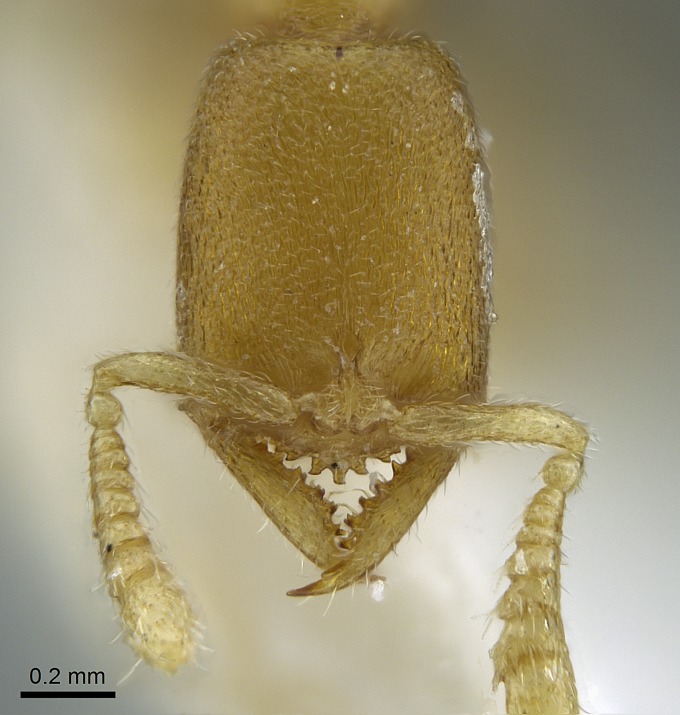
Paratype of *Stigmatomma besucheti* (CASENT0906835); worker. Image by F. A. Esteves; available at AntWeb.org

**Figure 116b.**
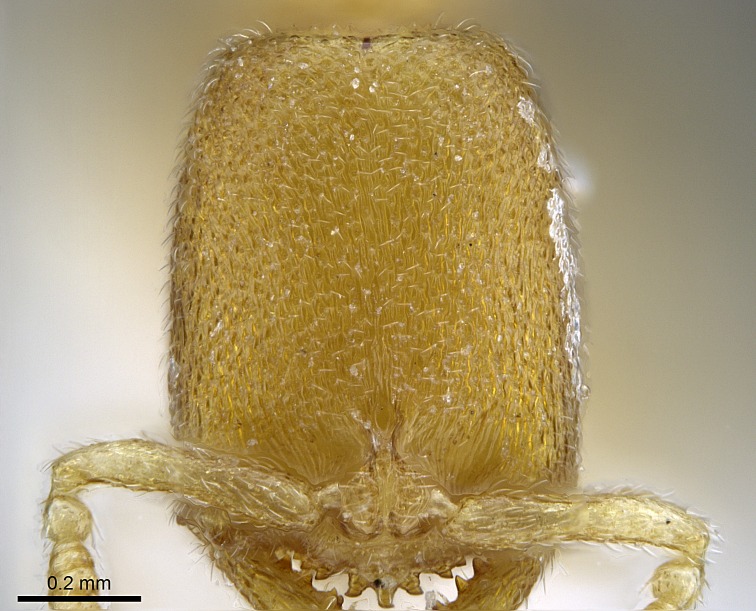
Paratype of *Stigmatomma besucheti* (CASENT0906835); worker: close-up of the head sculpture. Images by F. A. Esteve; available at AntWeb.org

**Figure 116c.**
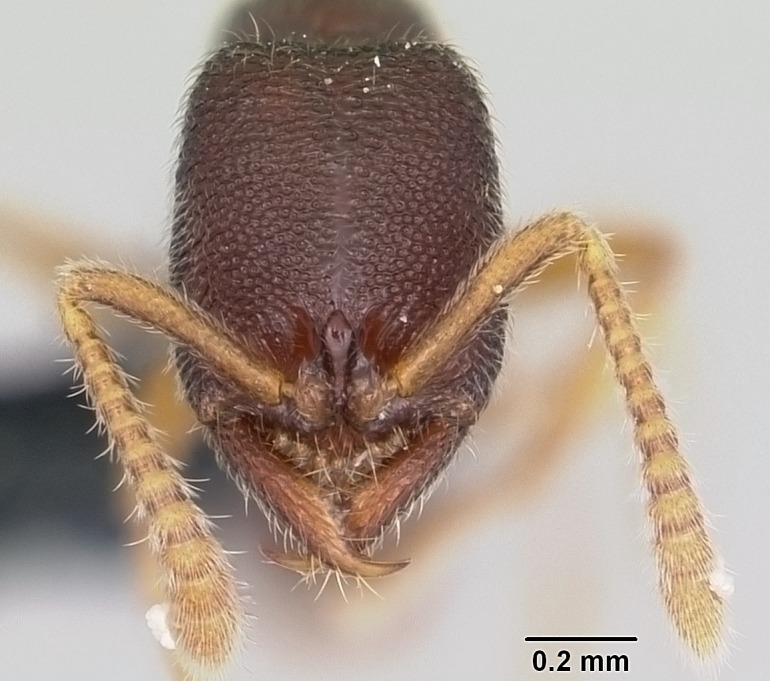
Holotype of *Stigmatomma bolabola* **sp. n.** (CASENT0034580); worker. Image by April Nobile; available at AntWeb.org

**Figure 116d.**
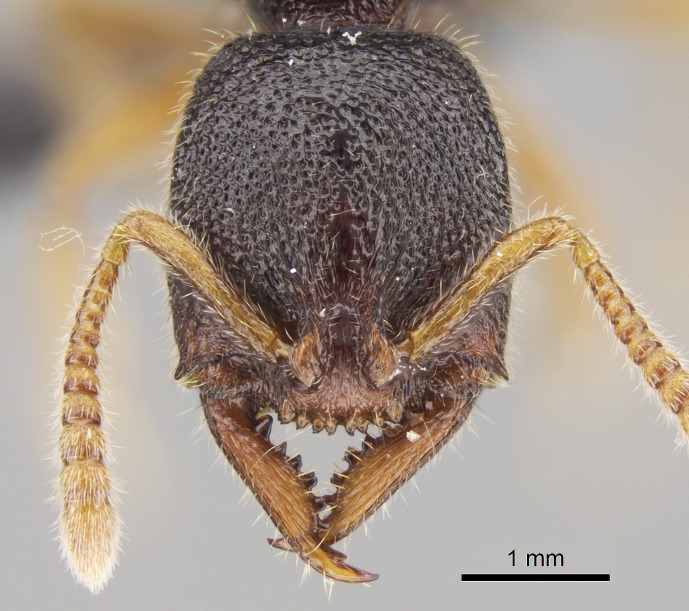
Holotype of *Stigmatomma irayhady* **sp. n.** (CASENT0042899); worker. Image by F. A. Esteves; available at AntWeb.org

**Figure 116e.**
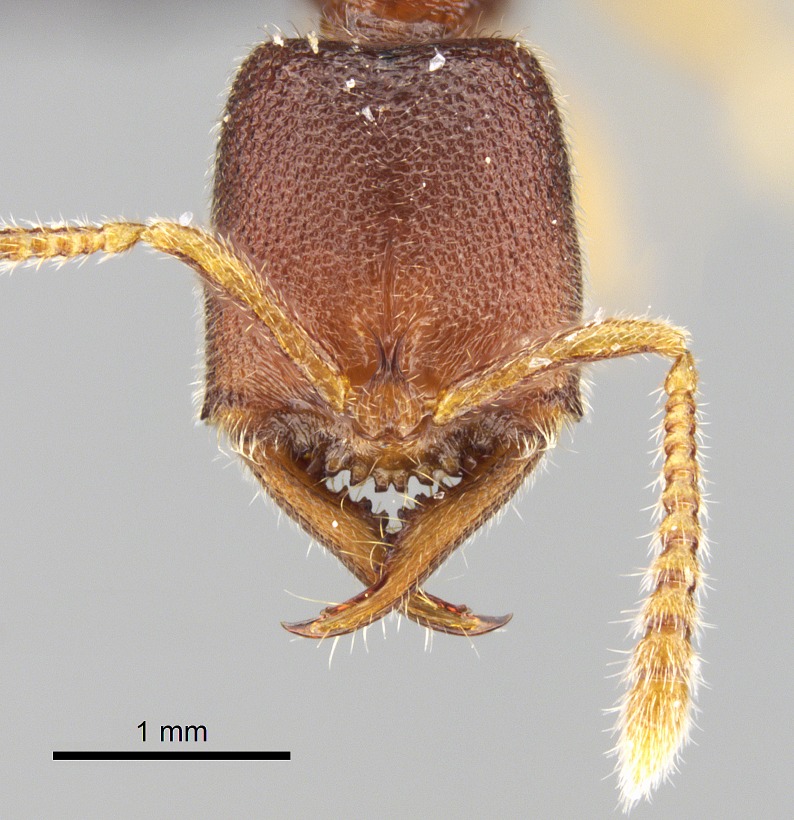
Holotype of *Stigmatomma janovitsika* **sp. n.** (CASENT0161533); worker. Image by F. A. Esteves; available at AntWeb.org

**Figure 116f.**
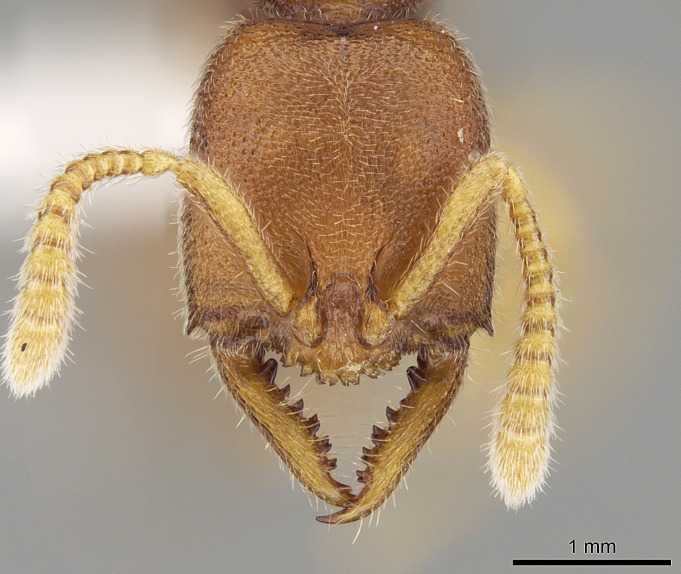
Holotype of *Stigmatomma liebe* **sp. n.** (CASENT0318428); worker. Image by F. A. Esteves; available at AntWeb.org

**Figure 117a.**
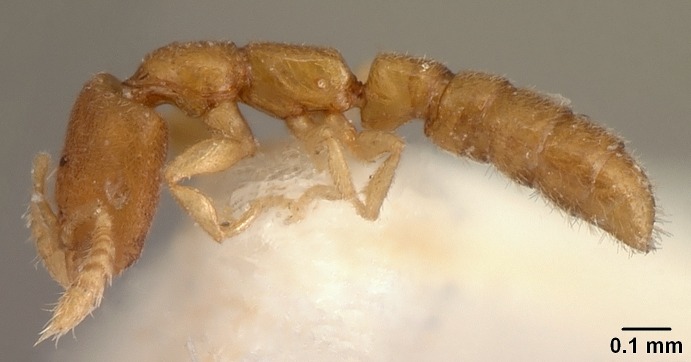
Holotype of *Stigmatomma besucheti* (CASENT0101816); worker. Image by April Nobile; available at AntWeb.org

**Figure 117b.**
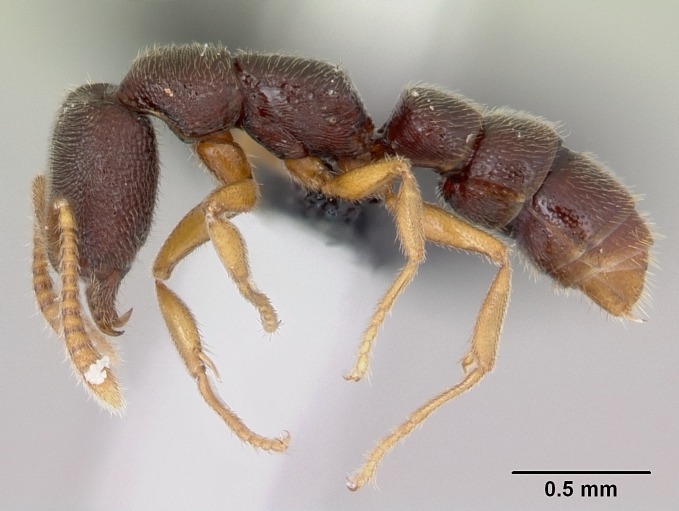
Holotype of *Stigmatomma bolabola* **sp. n.** (CASENT0034580); worker. Image by April Nobile; available at AntWeb.org

**Figure 117c.**
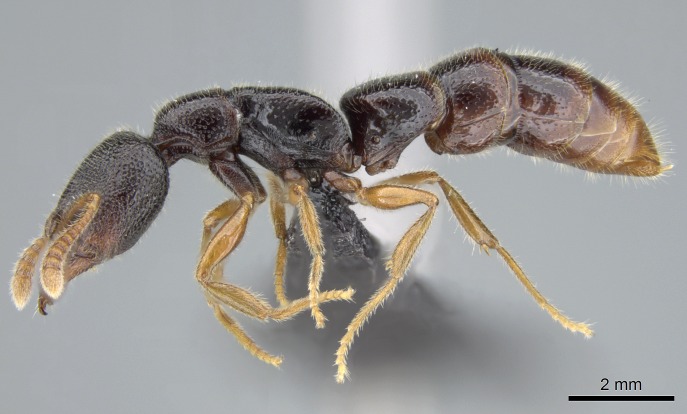
Holotype of *Stigmatomma irayhady* **sp. n.** (CASENT0042899); worker. Image by F. A. Esteves; available at AntWeb.org

**Figure 117d.**
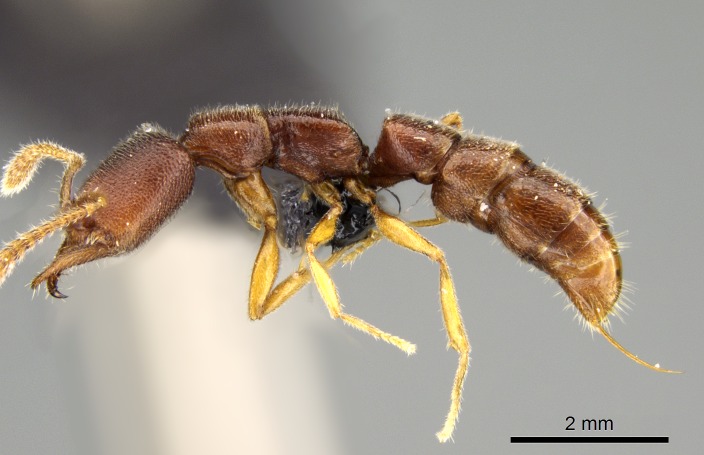
Holotype of *Stigmatomma janovitsika* **sp. n.** (CASENT0161533); worker. Image by F. A. Esteves; available at AntWeb.org

**Figure 117e.**
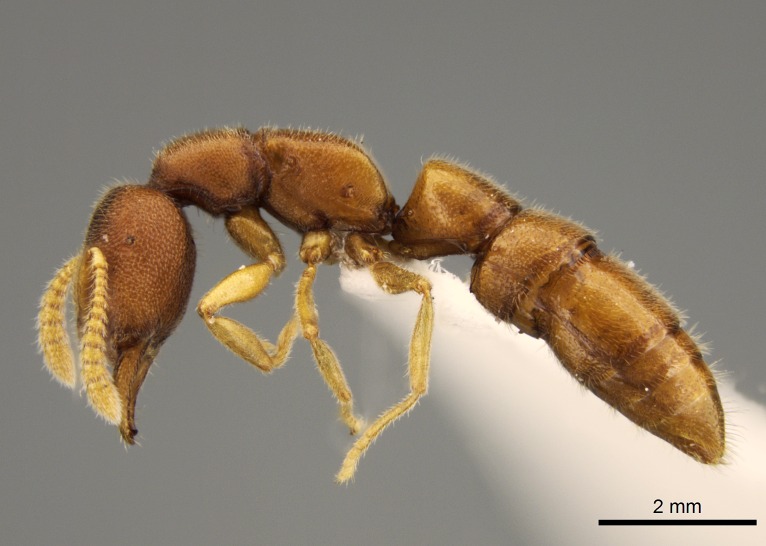
Holotype of *Stigmatomma liebe* **sp. n.** (CASENT0318428); worker. Image by F. A. Esteves; available at AntWeb.org

**Figure 117f.**
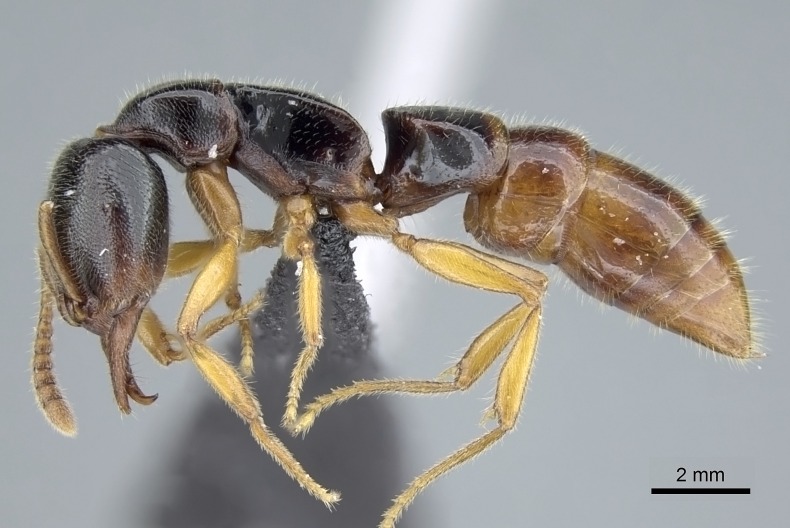
Holotype of *Stigmatomma roahady* **sp. n.** (CASENT0318421); worker. Image by F. A. Esteves; available at AntWeb.org

**Figure 118a.**
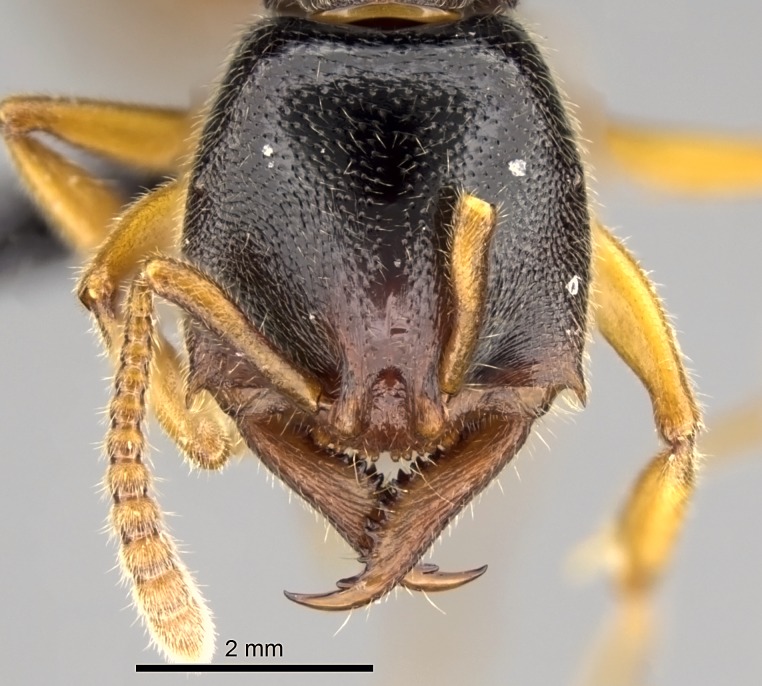
Holotype of *Stigmatomma roahady* **sp. n.** (CASENT0318421); worker. Image by F. A. Esteves; available at AntWeb.org

**Figure 118b.**
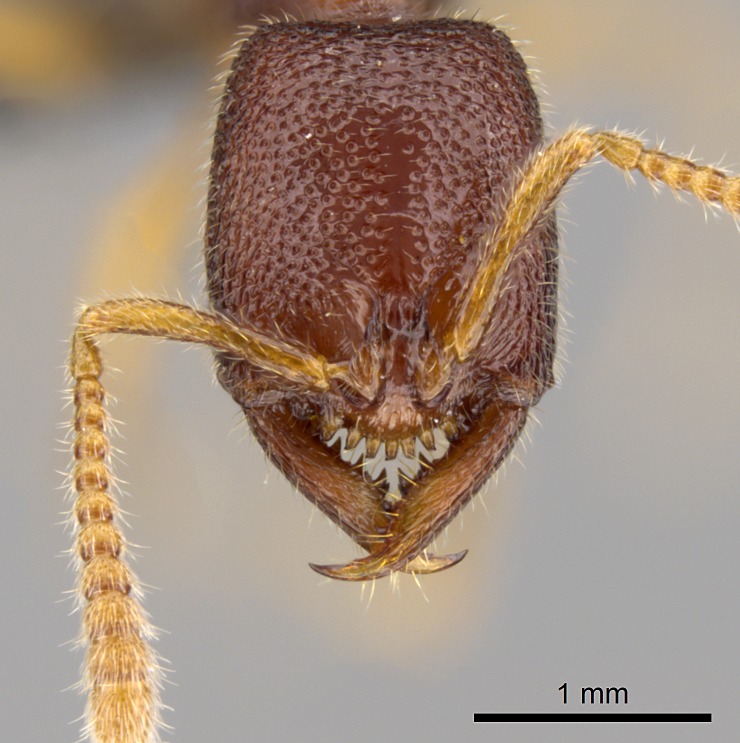
Holotype of *Stigmatomma sakalava* **sp. n.** (CASENT0366766); worker. Image by F. A. Esteves; available at AntWeb.org

**Figure 118c.**
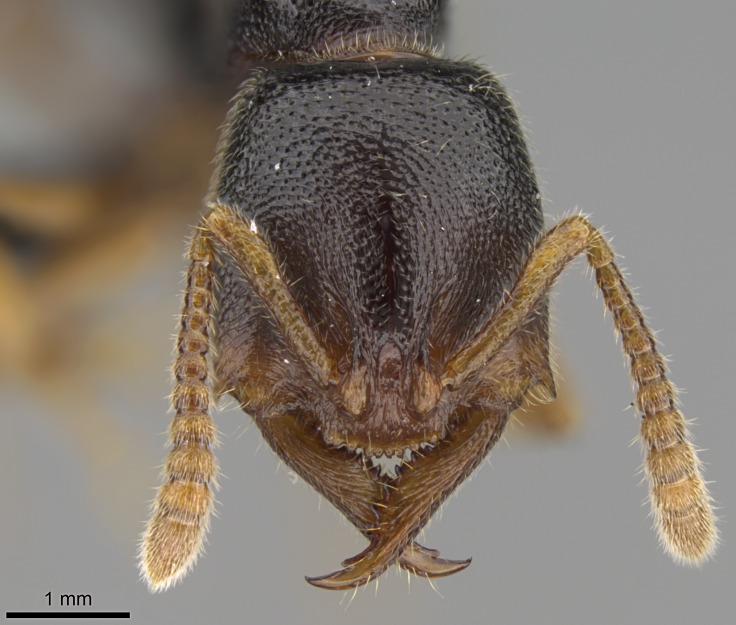
Holotype of *Stigmatomma tsyhady* **sp. n.** (CASENT0121332); worker. Image by F. A. Esteves; available at AntWeb.org

**Figure 119a.**
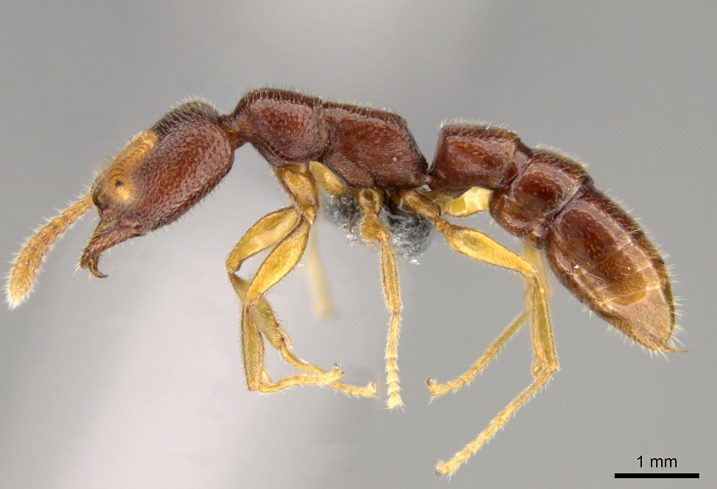
Holotype of *Stigmatomma sakalava* **sp. n.** (CASENT0366766); worker. Image by F. A. Esteves; available at AntWeb.org

**Figure 119b.**
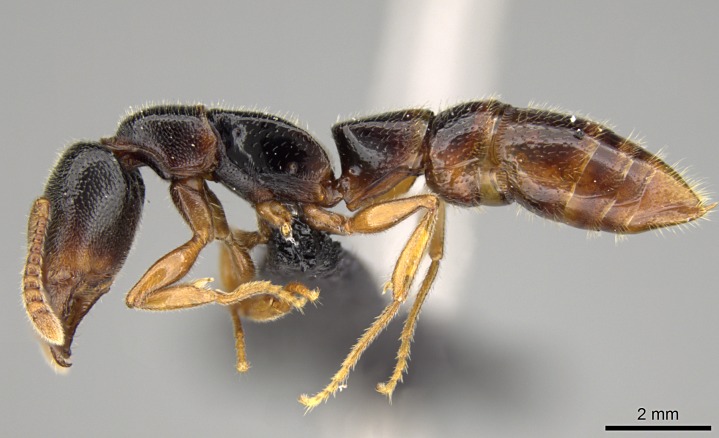
Holotype of *Stigmatomma tsyhady* **sp. n.** (CASENT0121332); worker. Image by F. A. Esteves; available at AntWeb.org

**Figure 120a.**
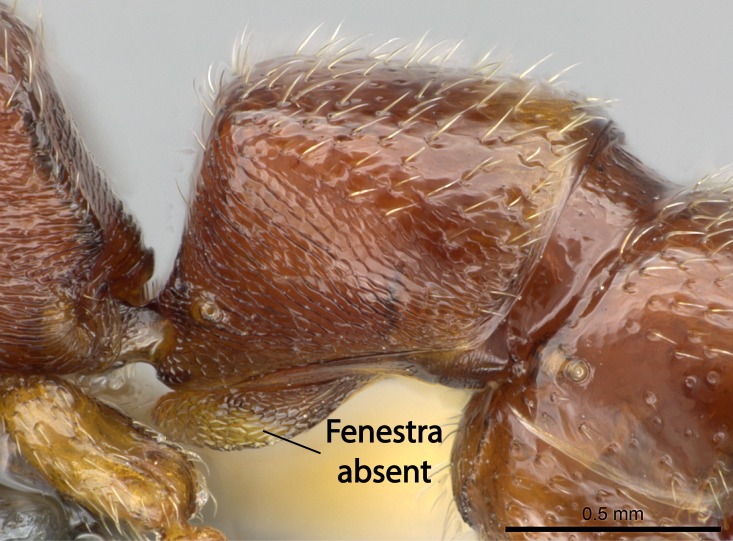
Holotype of *Stigmatomma sakalava* **sp. n.** (CASENT0366766); worker; lateral face of the petiole. Note the absence of a fenestra on the lateral face of the subpetiolar process. Image by F. A. Esteves; available at AntWeb.org

**Figure 120b.**
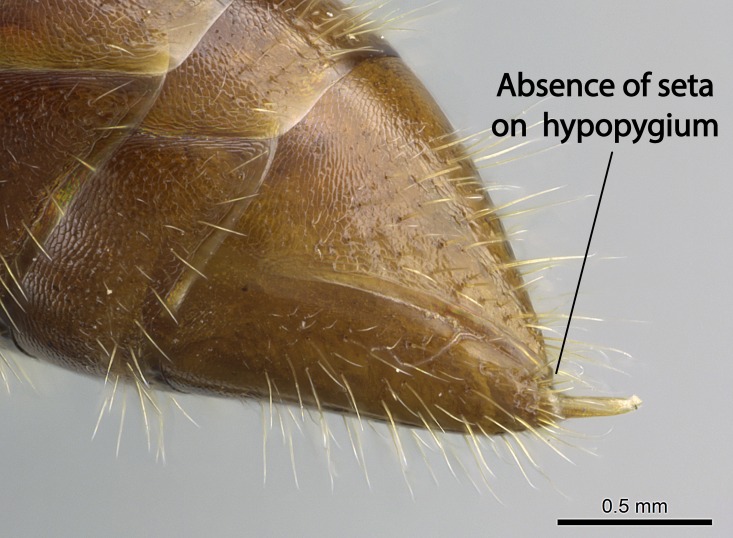
Holotype of *Stigmatomma irayhady* **sp. n.** (CASENT0042899); worker; lateral face of the apex of the gaster. Note the absence of stout setae on the apex of the hypopygium. Image by F. A. Esteves; available at AntWeb.org

**Figure 120c.**
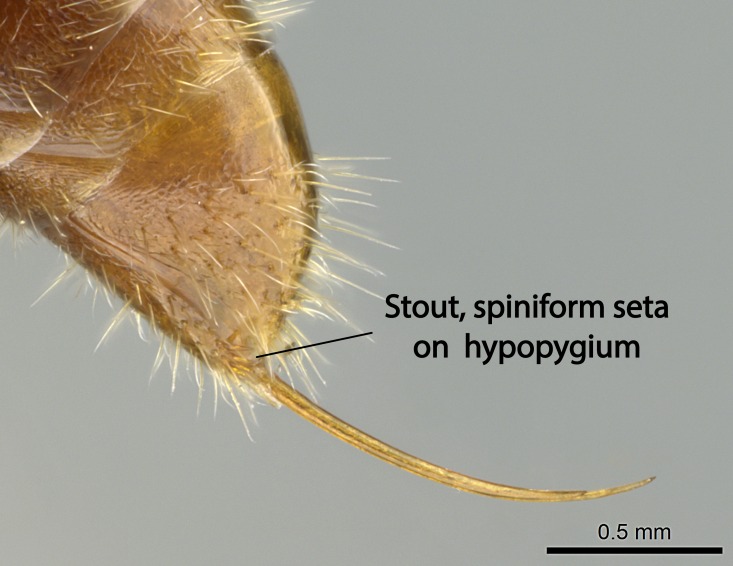
Holotype of *Stigmatomma janovitsika* **sp. n.** (CASENT0161533); worker; lateral face of the apex of the gaster. Note the presence of stout, spiniform setae on the apex of the hypopygium. Image by F. A. Esteves; available at AntWeb.org

**Figure 120d.**
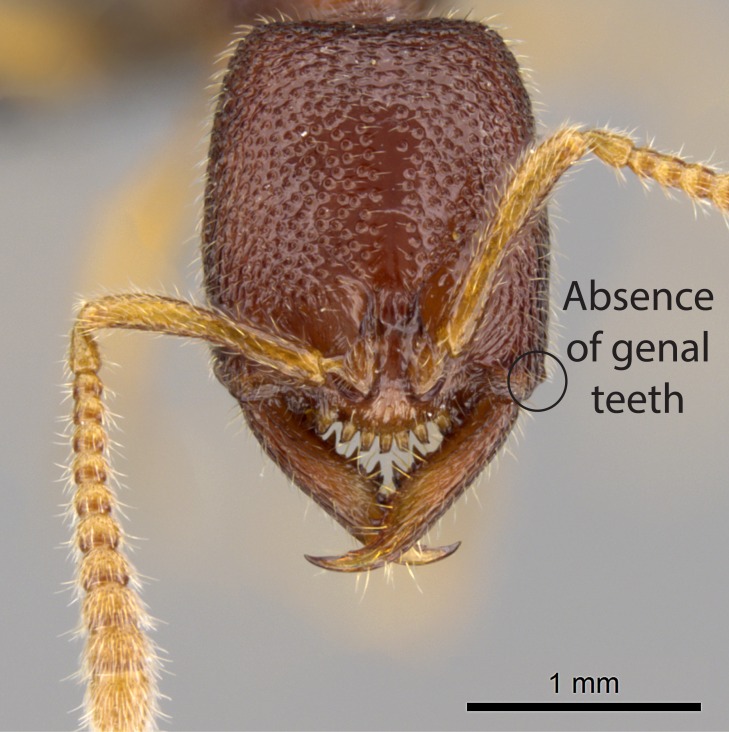
Holotype of *Stigmatomma sakalava* **sp. n.** (CASENT0366766); worker; dorsal face of the head. Note the absence of genal teeth. Image by F. A. Esteves; available at AntWeb.org

**Figure 120e.**
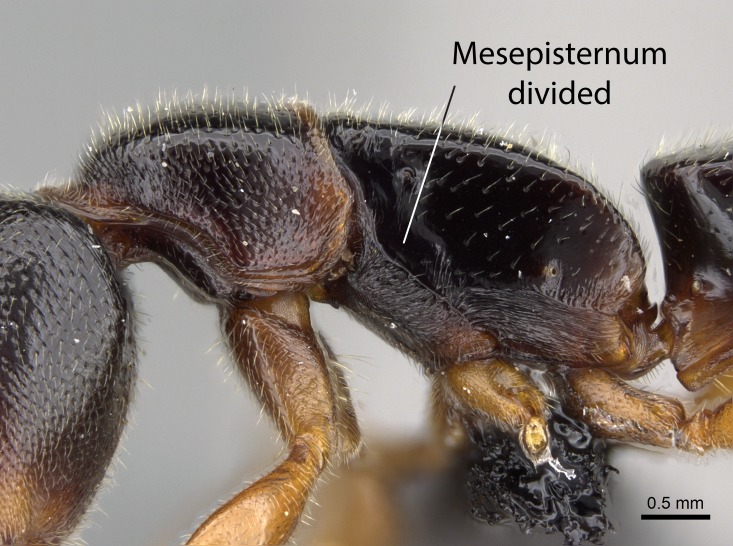
Holotype of *Stigmatomma tsyhady* **sp. n.** (CASENT0121332); worker; lateral face of the mesosoma. Note that the mesepisternum is divided into anepisternum and katepisternum. Image by F. A. Esteves; available at AntWeb.org

**Figure 121a.**
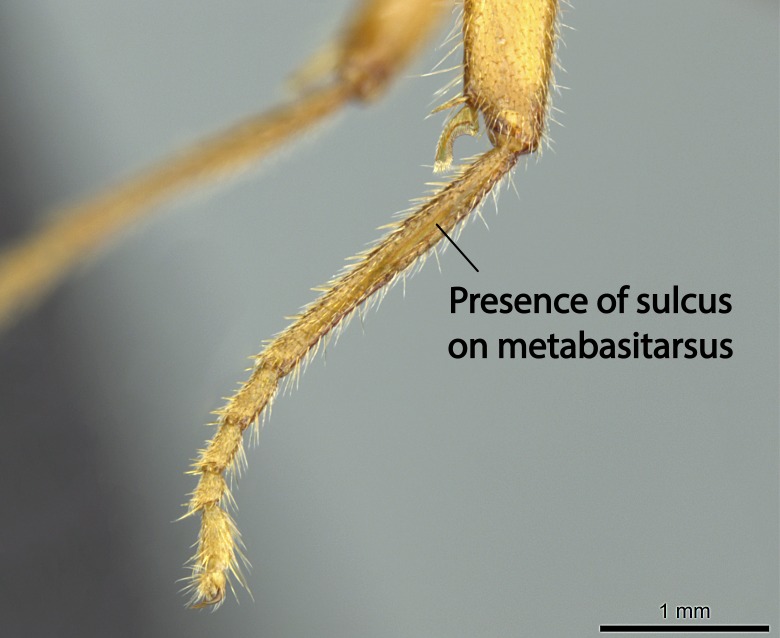
Holotype of *Stigmatomma roahady* **sp. n.** (CASENT0318421); worker; anterior face of the hindleg: close-up of the apical portion of tibia, and tarsi. Note the presence of a sulcus on the metabasitarsus. Image by F. A. Esteves ; available at AntWeb.org

**Figure 121b.**
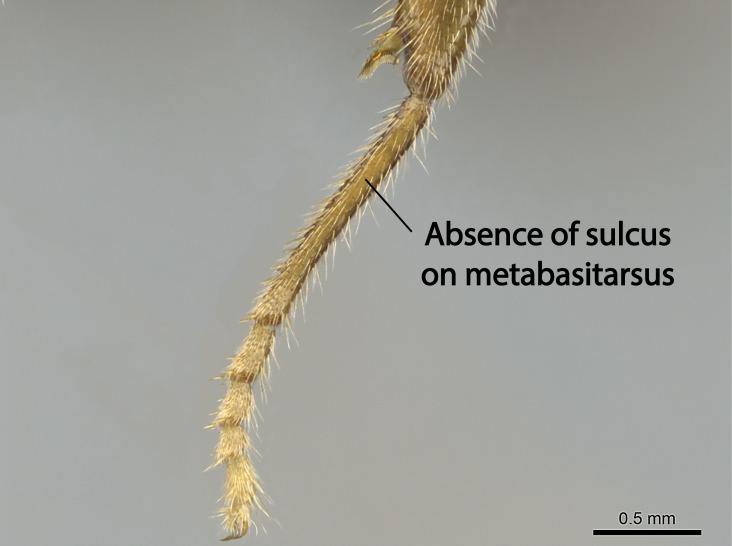
Holotype of *Stigmatomma irayhady* **sp. n.** (CASENT0042899); worker; anterior face of the hindleg: close-up of the apical portion of tibia, and tarsi. Note the absence of a sulcus on the metabasitarsus. Image by F. A. Esteves; available at AntWeb.org

**Figure 121c.**
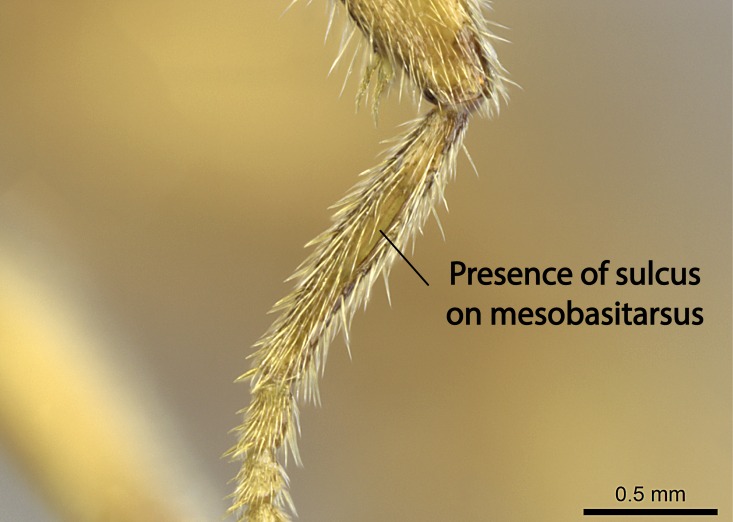
Holotype of *Stigmatomma roahady* **sp. n.** (CASENT0318421); worker; anterior face of the midleg: close-up of the apical portion of tibia, and basal tarsi. Note the presence of a sulcus on the mesobasitarsus. Image by F. A. Esteves; available at AntWeb.org

**Figure 121d.**
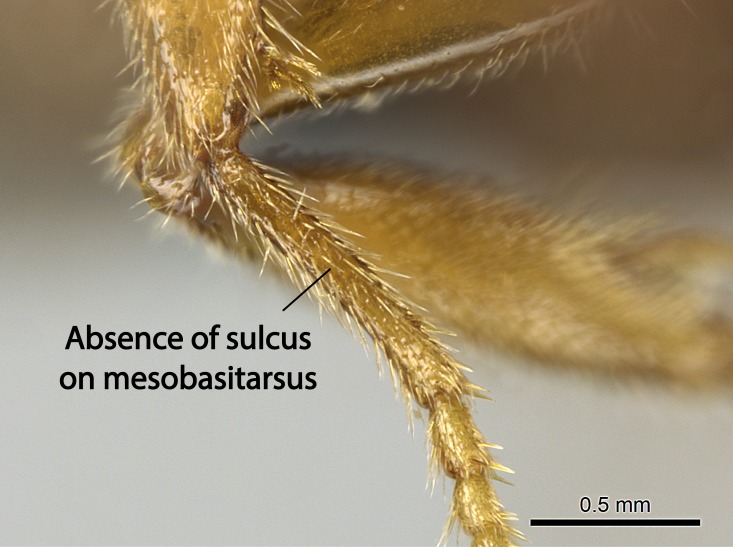
Holotype of *Stigmatomma tsyhady* **sp. n.** (CASENT0121332); worker; anterior face of the midleg: close-up of the apical portion of tibia, and basal tarsi. Note the absence of a sulcus on the mesobasitarsus. Image by F. A. Esteves; available at AntWeb.org

**Figure 122.**
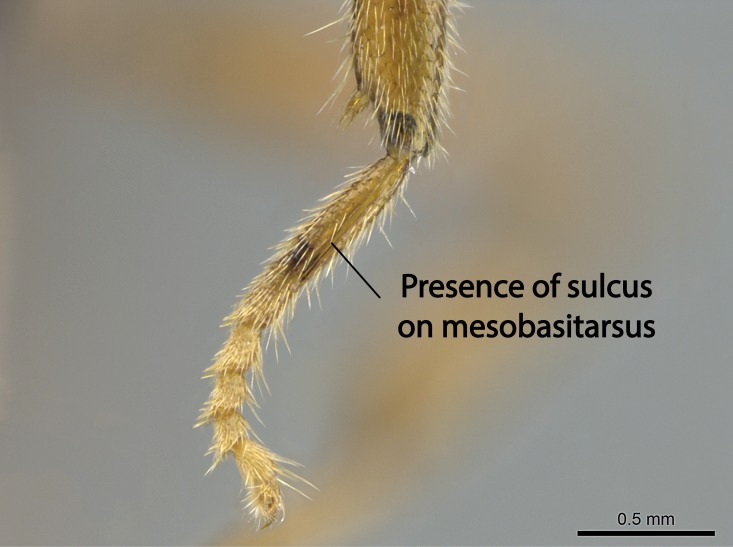
Holotype of *Stigmatomma irayhady* **sp. n.** (CASENT0042899); worker; anterior face of the midleg: close-up of the apical portion of tibia, and tarsi. Note the presence of a sulcus on the mesobasitarsus. Image by F. A. Esteves ; available at AntWeb.org

**Figure 123a.**
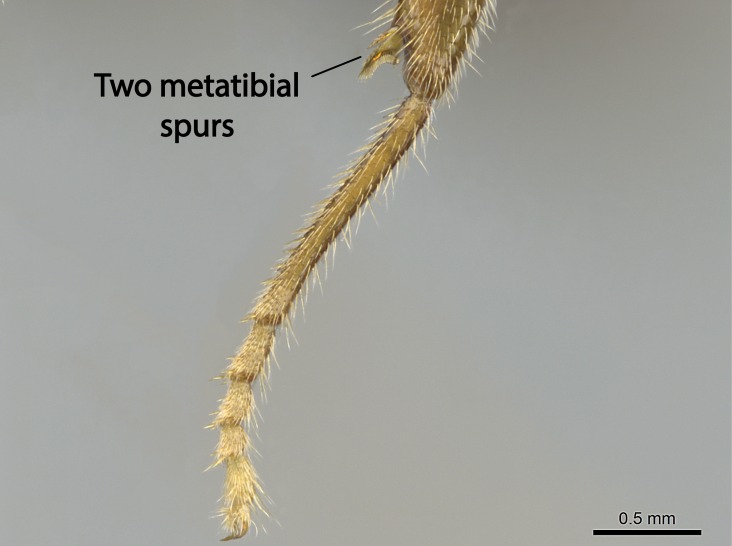
Holotype of *Stigmatomma irayhady* **sp. n.** (CASENT0042899); worker; anterior face of the hindleg: close up of the apical portion of tibia, and tarsi. Note that two metatibial spurs are visible. Image by F. A. Esteves ; available at AntWeb.org

**Figure 123b.**
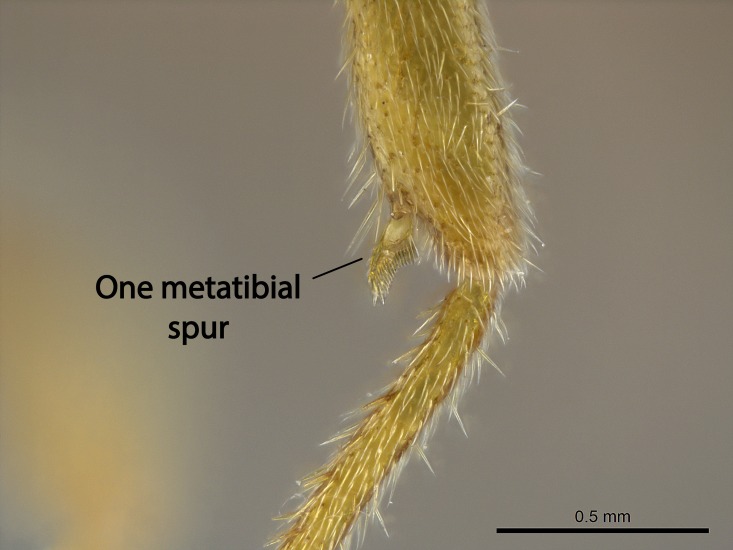
Holotype of *Stigmatomma liebe* **sp. n.** (CASENT0318428); worker; anterior face of the hindleg: close up of the apical portion of tibia, and basitarsus. Note that only the posterior metatibial spur is visible. Image by F. A. Esteves; available at AntWeb.org

**Figure 123c.**
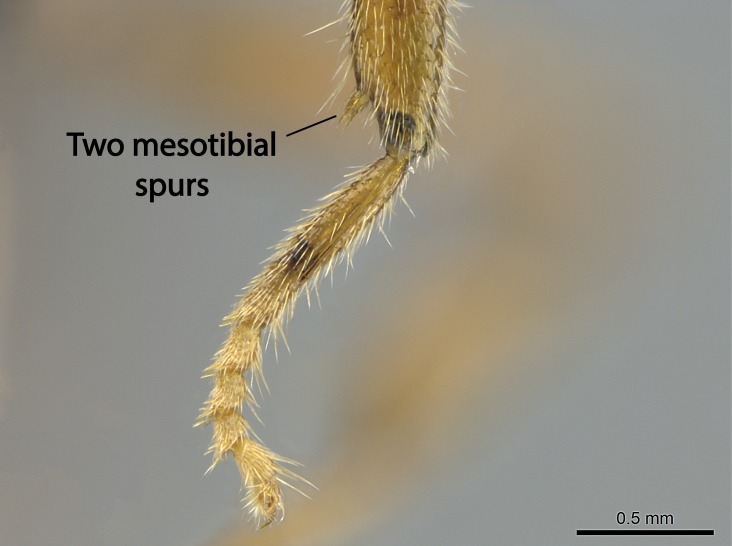
Holotype of *Stigmatomma irayhady* **sp. n.** (CASENT0042899); worker; anterior face of the midleg: close up of the apical portion of tibia, and tarsi. Note that two mesotibial spurs are visible. Image by F. A. Esteves; available at AntWeb.org

**Figure 123d.**
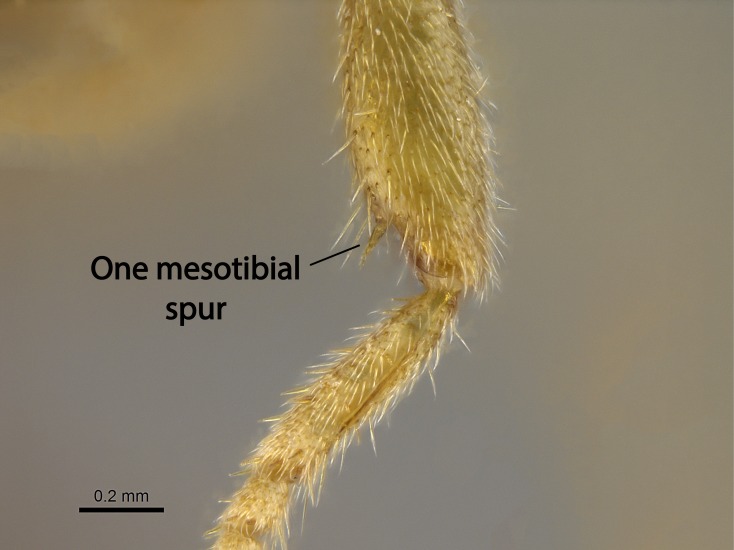
Holotype of *Stigmatomma liebe* **sp. n.** (CASENT0318428); worker; anterior face of the midleg: close up of the apical portion of tibia, and basal tarsi. Note that only the posterior mesotibial spur is visible. Image by F. A. Esteves; available at AntWeb.org

**Figure 124a.**
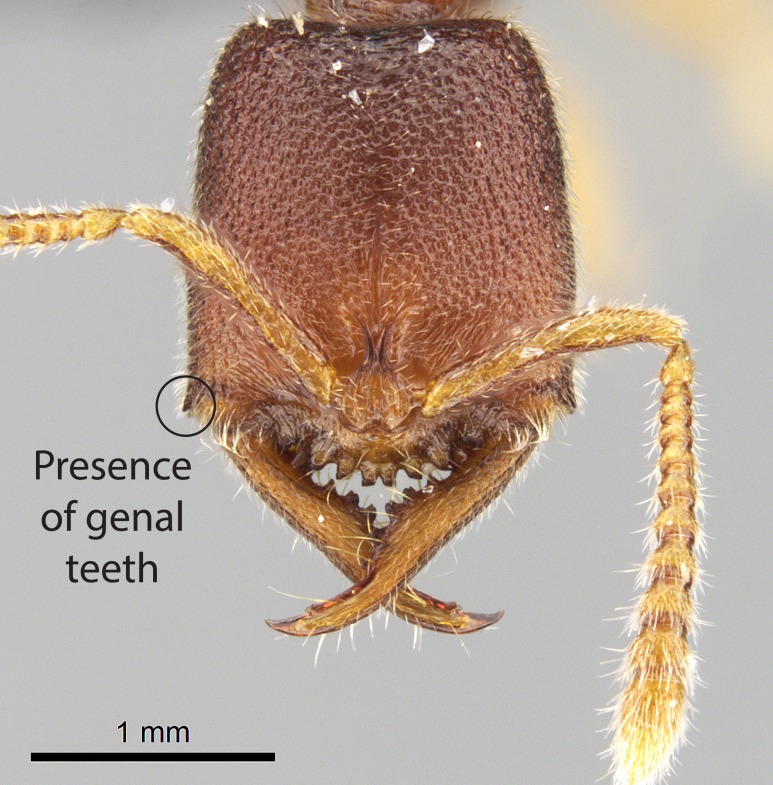
Holotype of *Stigmatomma janovitsika* **sp. n.** (CASENT0161533); worker; dorsal face of the head. Note the presence of genal teeth. Image by F. A. Esteves; available at AntWeb.org

**Figure 124b.**
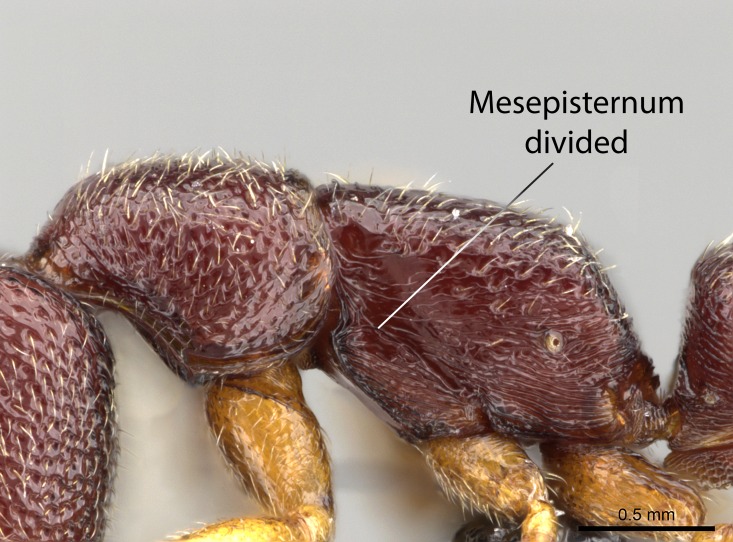
Holotype of *Stigmatomma bolabola* **sp. n.** (CASENT0034580); worker; lateral face of the mesosoma. Note that the mesepisternum is divided into anepisternum and katerpisternum. Image by F. A. Esteves; available at AntWeb.org

**Figure 124c.**
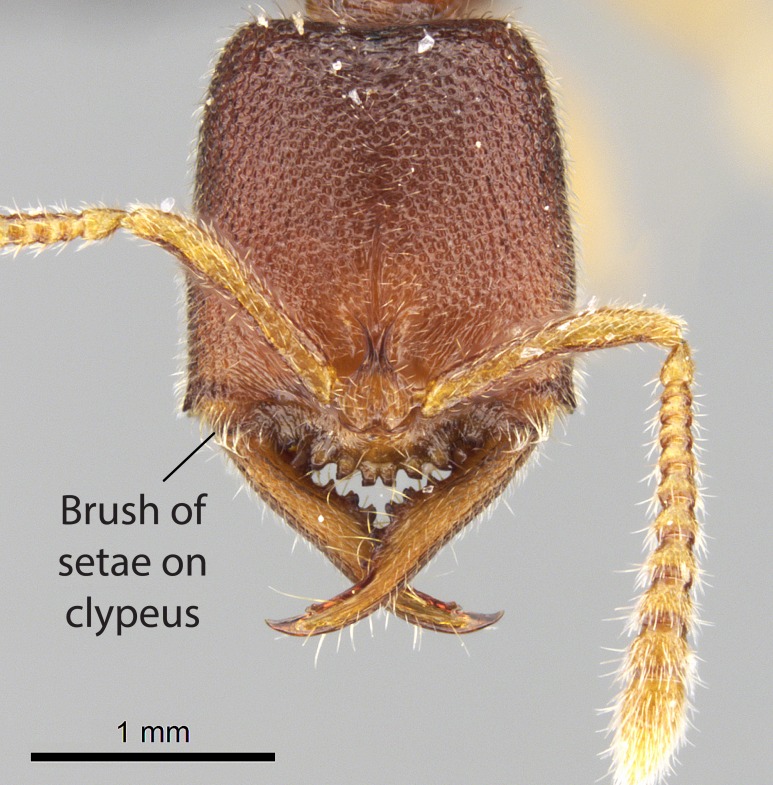
Holotype of *Stigmatomma janovitsika* **sp. n.** (CASENT0161533); worker; dorsal face of the head. Note the presence of a brush of setae on the lateral-most area of the clypeus. Image by F. A. Esteves; available at AntWeb.org

**Figure 124d.**
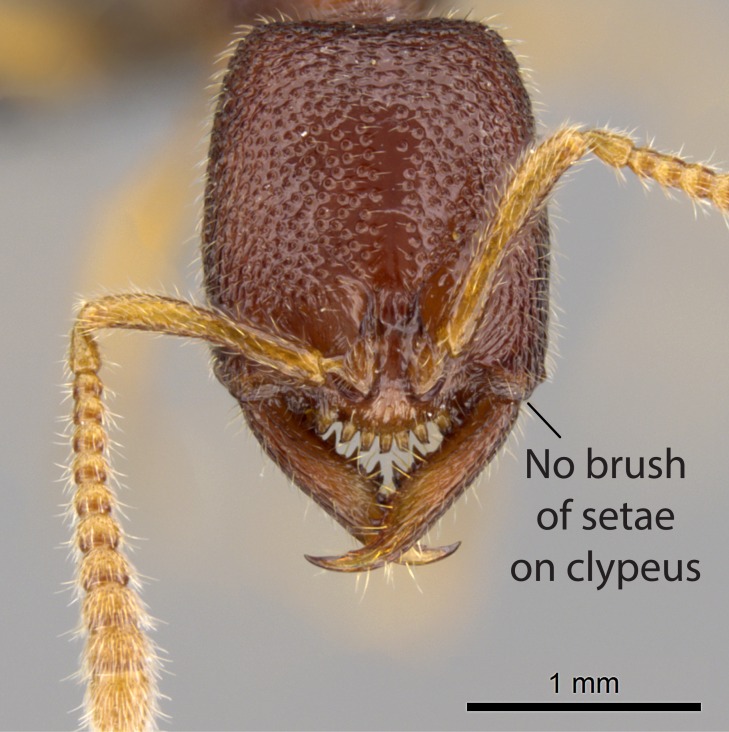
Holotype of *Stigmatomma sakalava* **sp. n.** (CASENT0366766); worker; dorsal face of the head. Note the absence of a brush of setae on the lateral-most area of the clypeus. Image by F. A. Esteves; available at AntWeb.org

**Figure 124e.**
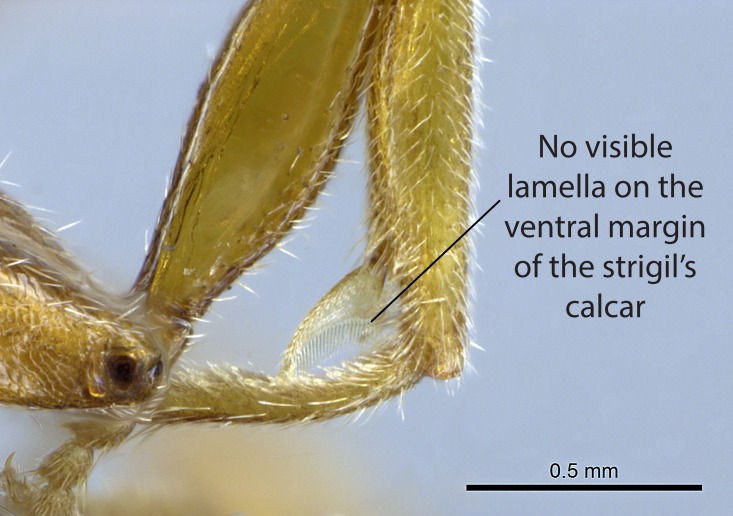
Paratype of *Stigmatomma janovitsika* **sp. n.** (CASENT0156022); worker; anterior face of the foreleg: basal portion of the femur, apical portion of the tibia, and tarsi. Note that a basal lamella is not visible on the ventral margin of the calcar of strigil. Image by F. A. Esteves; available at AntWeb.org

**Figure 124f.**
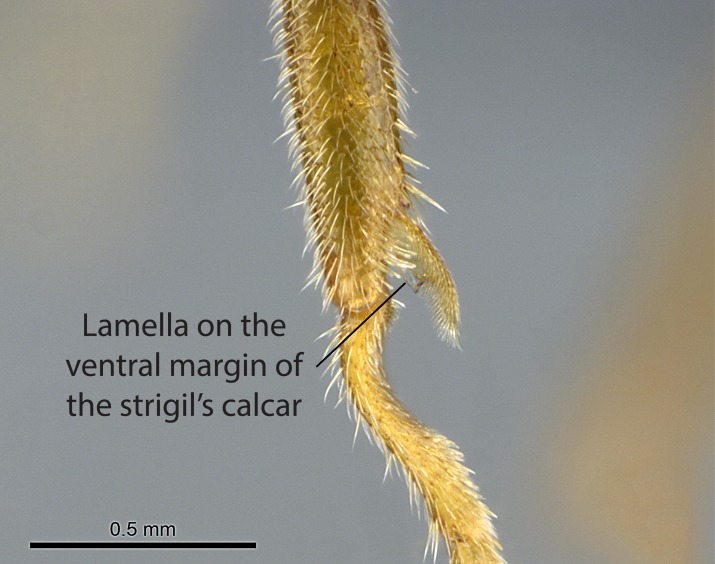
Holotype of *Stigmatomma sakalava* **sp. n.** (CASENT0366766); worker; anterior face of the foreleg: apical portion of the tibia, and basitarsus. Note the basal lamella on the ventral margin of the calcar of strigil. Image by F. A. Esteves; available at AntWeb.org

**Figure 125a.**
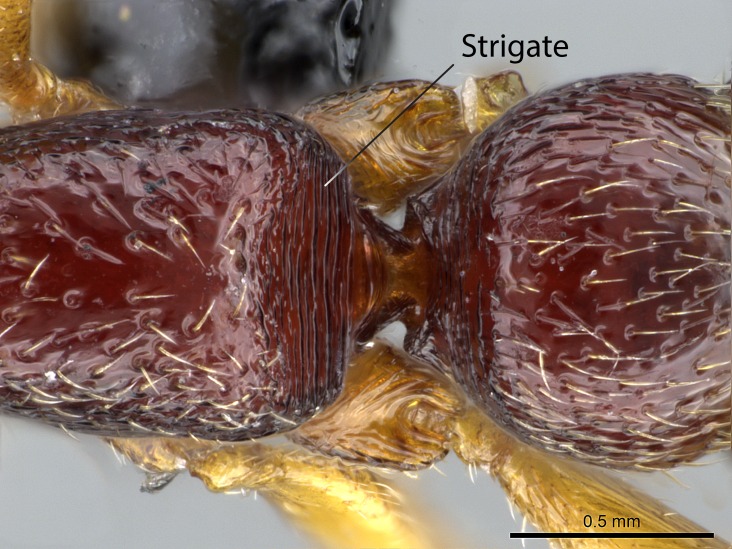
Holotype of *Stigmatomma bolabola* **sp. n.** (CASENT0034580); worker; dosal view of the propodeum and petiole. Note the strigate sculpture on the face of the propodeal declivity. Image by F. A. Esteves; available at AntWeb.org

**Figure 125b.**
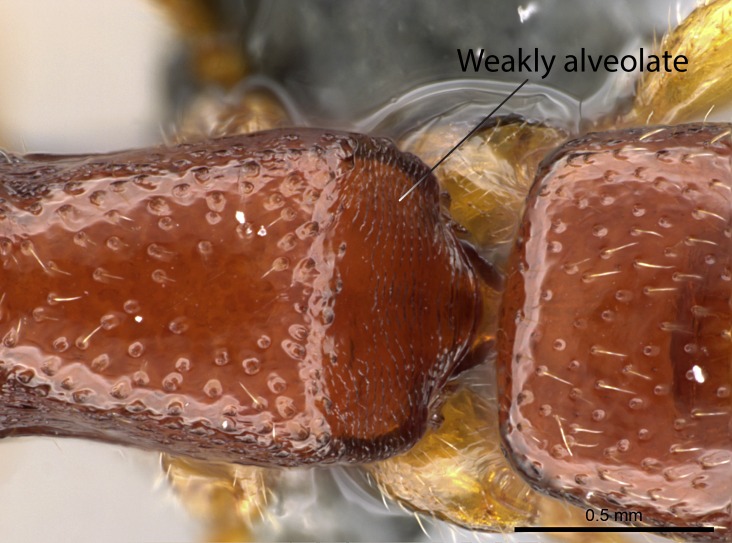
Holotype of *Stigmatomma sakalava* **sp. n.** (CASENT0366766); worker; dosal view of the propodeum and petiole. Note the strigate sculpture on the face of the propodeal declivity. Image by F. A. Esteves; available at AntWeb.org

**Figure 125c.**
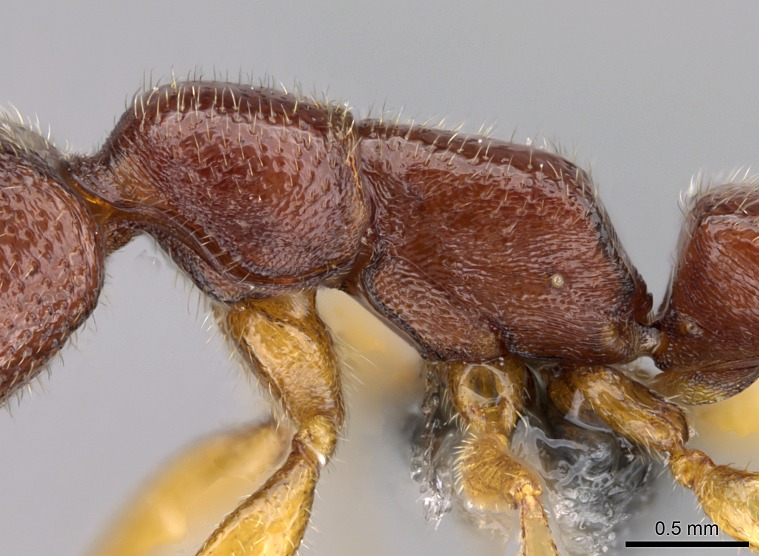
Holotype of *Stigmatomma sakalava* **sp. n.** (CASENT0366766); worker; lateral face of the mesosoma. Image by F. A. Esteves; available at AntWeb.org

**Table 1.** Matrix of correlation coefficients between morphometric variables. Positive coefficients are positive correlations, and negative coefficients, negative correlations; values equalling one indicate that the pair of variables is completely correlated, while zero means no correlation. This table shows that all variables are highly and positively correlated.

	**HL**	**HW**	**HW2**	**SL**	**ML**	**WL**	**PPW**	**PtL**	**PtW**
**HL**	1	0.996	0.994	0.981	0.983	0.987	0.979	0.973	0.979
**HW**	0.996	1	0.997	0.977	0.987	0.981	0.978	0.971	0.975
**HW2**	0.994	0.997	1	0.981	0.991	0.982	0.975	0.974	0.972
**SL**	0.981	0.977	0.981	1	0.974	0.975	0.961	0.951	0.961
**ML**	0.983	0.987	0.991	0.974	1	0.959	0.949	0.945	0.944
**WL**	0.987	0.981	0.982	0.975	0.959	1	0.991	0.988	0.991
**PPW**	0.979	0.978	0.975	0.961	0.949	0.991	1	0.989	0.994
**PtL**	0.973	0.971	0.974	0.951	0.945	0.988	0.989	1	0.99
**PtW**	0.979	0.975	0.972	0.961	0.944	0.991	0.994	0.99	1

**Table 2.** Proportion of the total variance and the proportion of cumulative variance encompassed by each PCA component.

	**PC1**	**PC2**	**PC3**	**PC4**	**PC5**	**PC6**	**PC7**	**PC8**	**PC9**
**Standard deviation**	2.9694	0.3341	0.1826	0.12122	0.09115	0.0828	0.06838	0.04687	0.03187
**Proportion of Variance**	0.9797	0.0124	0.0037	0.00163	0.00092	0.00076	0.00052	0.00024	0.00011
**Cumulative Proportion**	0.9797	0.9921	0.9958	0.99744	0.99836	0.99912	0.99964	0.99989	1

**Table 3.** Table containing eigenvectors values for each morphometric variable. Eigenvectors are the location of original measurements on each PCA component/axis, and represent the contribution of each variable to a given component—the larger the absolute value, the more important the variable.

	**PC1**	**PC2**	**PC3**	**PC4**	**PC5**	**PC6**	**PC7**	**PC8**	PC9
**HL**	0.3354	-0.1394	0.0640	-0.4269	0.4909	-0.0357	0.0441	-0.6569	-0.0777
**HW**	0.3350	-0.1945	0.2713	-0.3528	0.0288	0.2758	0.3343	0.4351	0.5234
**HW2**	0.3352	-0.2314	0.2059	0.1872	0.0529	0.0791	0.3272	0.2652	-0.7551
**SL**	0.3312	-0.2903	-0.8218	0.1941	-0.1033	0.2298	0.1183	-0.0695	0.1038
**ML**	0.3301	-0.5427	0.2984	0.2214	-0.2929	-0.2230	-0.5437	-0.0907	0.1378
**WL**	0.3347	0.2215	-0.2215	-0.0013	0.3403	-0.7187	-0.0744	0.3832	0.0709
**PPW**	0.3333	0.3584	0.0142	-0.2703	-0.7184	-0.2325	0.2441	-0.2279	-0.0741
**PtL**	0.3319	0.4104	0.2496	0.6725	0.1607	0.1612	0.1582	-0.2401	0.2688
**PtW**	0.3329	0.4054	-0.0635	-0.2149	0.0331	0.4661	-0.6161	0.1983	-0.1932
