# Supplementary material for: Taxonomic revision of Stigmatomma Roger (Hymenoptera: Formicidae) in the Malagasy region
Source: Biodivers Data J. 2016 Jun 13;(4):e8032. doi: 10.3897/BDJ.4.e8032 (PMC4934140; doi:10.3897/BDJ.4.e8032)
Supplement: Supplementary material 2 — R script for clustering specimens based on measurement data [file biodiversity_data_journal-4-e8032-s002.pdf]

Supplementary Materials for

Taxonomic revision of *Stigmatomma* Roger (Hymenoptera: Formicidae) in the Malagasy region

Flavia A. Esteves*, Brian L. Fisher

*Corresponding author. E-mail: flaviaesteves@gmail.com

This PDF file includes:

R script for UPGMA analysis
Author: Flavia A. Esteves

R script for UPGMA analysis

```
## Setting working directory ##
setwd("write the address of the directory that contains the measurements data file")

## Reading original dataset ## ## Download available in the Suppl. Material 1 ##
ant = read.table("Supplementary material_measurements.txt",header=TRUE)

## Creating data-frame for analysis ##
dataset = data.frame(ant[,c(5,6,7,8,9,10,11,12,13)])
rownames(dataset) = paste(ant[,2], ant[,3], sep=' ')

## Normalizing the dataset ##
comlog = log(dataset)

##Creating dissimilarity matrix ##
dist = dist(comlog, method="euclidean")

## Clustering ##
plot.cluster = hclust(dist,"average")

## Calculating cophenetic distances ##
coph = cophenetic (plot.cluster)

## Calculating linear correlation between cophenetic and original distances ##
cor(dist,coph)

## Creating figure ##
plot(plot.cluster,hang=-1)
```
