# Supplementary material for: Taxonomic revision of Stigmatomma Roger (Hymenoptera: Formicidae) in the Malagasy region
Source: Biodivers Data J. 2016 Jun 13;(4):e8032. doi: 10.3897/BDJ.4.e8032 (PMC4934140; doi:10.3897/BDJ.4.e8032)
Supplement: Supplementary material 3 — R script for Principal Component Analysis (PCA): specimens on a morphometric ordination space [file biodiversity_data_journal-4-e8032-s003.pdf]

Supplementary Materials for

Taxonomic revision of *Stigmatomma* Roger (Hymenoptera: Formicidae) in the Malagasy region

Flavia A. Esteves*, Brian L. Fisher

*Corresponding author. E-mail: flaviaesteves@gmail.com

This PDF file includes:

R script for Principal Component Analysis (PCA)
Author: Flavia A. Esteves

R script for Principal Component Analysis (PCA)

```
## Setting working directory ##
setwd ("directory that contains the measurements data file")

## Reading original dataset ## ## Download available in the Suppl. Material 1 ##
ant = read.table("Supplementary material_measurements.txt",header=TRUE)

## Creating data-frame for analysis ##
dataset = data.frame(ant[,c(5,6,7,8,9,10,11,12,13)])
rownames(dataset) = paste(ant[,3])

## Creating object containing grouping factors ##
species = factor(ant[,2])

## Checking variable correlations ##
round(cor(dataset[,1:9]),3)

##Performing PCA ##
pca = prcomp(dataset,scale=T)
summary(pca)

## Scree plot of the proportion of explained variance of each PCA component ##
require(ggbiplot)
ggscreeplot(pca)

## Obtaining eigenvector values ##
pca$rotation

## Creating PCA graphic ##
g =
ggbiplot(pca,choices=c(1,2),obs.scale=1,var.scale=0,groups=species,ellipse=F,ci
rcle=F,var.axes= F)
```

```
g =
g+scale_colour_manual(values=c("#543005","#EFA30C","#80cdc1","#8C5D26","
#003c30","#0BD1AA","#F6E8C3","#f5f5f5"))
g = g+theme(legend.direction="horizontal",legend.position="bottom")
print(g)
```
