# Supplementary material for: Taxonomic revision of Stigmatomma Roger (Hymenoptera: Formicidae) in the Malagasy region
Source: Biodivers Data J. 2016 Jun 13;(4):e8032. doi: 10.3897/BDJ.4.e8032 (PMC4934140; doi:10.3897/BDJ.4.e8032)
Supplement: Supplementary material 4 — R script for mapping the distribution of Stigmatomma species in Madagascar and Seychelles. [file biodiversity_data_journal-4-e8032-s004.pdf]

Supplementary Materials for

Taxonomic revision of *Stigmatomma* Roger (Hymenoptera: Formicidae) in the Malagasy region

Flavia A. Esteves*, Brian L. Fisher

*Corresponding author. E-mail: flaviaesteves@gmail.com

This PDF file includes:

R script for mapping species distribution
Author: Flavia A. Esteves

```
## Script for mapping species distribution in Madagascar ##

## Reading species distribution data ##
setwd("directory that contains the distribution data ")
samps = read.csv("distribution data.csv")

## Downloading altitude data for Madagascar – raster layer##
require(raster)
alt = getData("alt",country="MDG")

## Reading ecoregion polygons – vector layers ##
require(rgdal)
ned = readOGR("directory that contains the shape file","Dry Forests")
ned2 = readOGR("directory that contains the shape file","Subhumid Forests")
ned3 = readOGR("directory that contains the shape file","Humid forests")
ned4 = readOGR("directory that contains the shape file","Succulent Woodlands")
ned5 = readOGR("directory that contains the shape file","Spiny Thickets")

## Standardizing projections of raster and vector layers ##
proj4string(ned5)
crs.geo =
CRS("+proj=longlat+datum=WGS84+no_defs+ellps=WGS84+towgs84=0,0,0")
proj4string(alt) = crs.geo

## Obtaining the shaded-relief of Madagascar ##
slope = terrain(alt,opt ="slope")
aspect = terrain(alt,opt ="aspect")
hill = hillShade(slope,aspect,40,270)

## Mapping shaded-relief, altitude, ecoregions, and distribution points ##
require(scales)
plot(hill,col=grey(0:100/100),legend=FALSE)
plot(alt,col=grey(0:100/100,alpha=0.30),legend=FALSE,add=TRUE)
plot(ned,col=alpha("brown",0.30),border=FALSE,add=TRUE)
```

```
plot(ned2,col=alpha("limegreen",0.30),border=FALSE,add=TRUE)
plot(ned4,col=alpha("green4", 0.30),border=FALSE,add=TRUE)
plot(ned3,col=alpha("orange",0.30),border=FALSE,add=TRUE)
plot(ned5,col=alpha("yellow",0.30),border=FALSE,add=TRUE)
points(samps$LocLongitude,samps$LocLatitude,pch=21,col="black",bg="white",
cex=1)
```

## Script for mapping species distribution in Seychelles ##

```
## Reading species distribution data ##
setwd("directory that contains the distribution data ")
samps = read.csv("distribution data.csv")

## Reading altitude data for Seychelles ##
require(rgdal)
alt = readGDAL("altitude file.tif")

## Setting projection of altitude data ##
require(raster)
crs.geo = CRS("+proj=longlat +datum=WGS84 +no_defs +ellps=WGS84
+towgs84=0,0,0")
proj4string(alt) = crs.geo

## Rasterizing elevation data ##
altraster = raster(alt)

## Obtaining the shaded-relief of Seychelles ##
slope = terrain(alt,opt="slope")
aspect = terrain(alt,opt="aspect")
hill = hillShade(slope,aspect,40,270)

## Mapping shaded-relief, altitude, and distribution points ##
require(scales)
plot(hill,col=grey(0:100/100),legend=FALSE)
plot(altraster,col=terrain.colors(6,alpha=0.30),legend=FALSE,add=TRUE)
points(samps$LocLongitude,samps$LocLatitude,pch=21,col="black",bg="yellow",
cex=1)
```
